# Water electrolysis: from textbook knowledge to the latest scientific strategies and industrial developments†

**DOI:** 10.1039/d0cs01079k

**Published:** 2022-05-16

**Authors:** Marian Chatenet, Bruno G. Pollet, Dario R. Dekel, Fabio Dionigi, Jonathan Deseure, Pierre Millet, Richard D. Braatz, Martin Z. Bazant, Michael Eikerling, Iain Staffell, Paul Balcombe, Yang Shao-Horn, Helmut Schäfer

**Affiliations:** University Grenoble Alpes, University Savoie Mont Blanc, CNRS, Grenoble INP (Institute of Engineering and Management University Grenoble Alpes) LEPMI, 38000 Grenoble France; Hydrogen Energy and Sonochemistry Research group, Department of Energy and Process Engineering, Faculty of Engineering, Norwegian University of Science and Technology (NTNU) NO-7491 Trondheim Norway; Green Hydrogen Lab, Institute for Hydrogen Research (IHR), Université du Québec à Trois-Rivières (UQTR) 3351 Boulevard des Forges Trois-Rivières Québec G9A 5H7 Canada; The Wolfson Department of Chemical Engineering, Technion – Israel Institute of Technology Haifa 3200003 Israel; The Nancy & Stephen Grand Technion Energy Program (GTEP), Technion – Israel Institute of Technology Haifa 3200003 Israel; Department of Chemistry, Chemical Engineering Division, Technical University Berlin 10623 Berlin Germany; Paris-Saclay University, ICMMO (UMR 8182) 91400 Orsay France; Elogen, 8 avenue du Parana 91940 Les Ulis France; Department of Chemical Engineering, Massachusetts Institute of Technology Cambridge Massachusetts 02139 USA; Department of Mathematics, Massachusetts Institute of Technology 77 Massachusetts Avenue Cambridge Massachusetts 02139 USA; Chair of Theory and Computation of Energy Materials, Division of Materials Science and Engineering, RWTH Aachen University Intzestraße 5 52072 Aachen Germany; Institute of Energy and Climate Research, IEK-13: Modelling and Simulation of Materials in Energy Technology Forschungszentrum Jülich GmbH 52425 Jülich Germany; Centre for Environmental Policy, Imperial College London London UK; Division of Chemical Engineering and Renewable Energy, School of Engineering and Material Science, Queen Mary University of London London UK; Research Laboratory of Electronics and Department of Mechanical Engineering, Massachusetts Institute of Technology Cambridge Massachusetts 02139 USA; Institute of Chemistry of New Materials, The Electrochemical Energy and Catalysis Group, University of Osnabrück Barbarastrasse 7 49076 Osnabrück Germany helmut.schaefer@uos.de

## Abstract

Replacing fossil fuels with energy sources and carriers that are sustainable, environmentally benign, and affordable is amongst the most pressing challenges for future socio-economic development. To that goal, hydrogen is presumed to be the most promising energy carrier. Electrocatalytic water splitting, if driven by green electricity, would provide hydrogen with minimal CO_2_ footprint. The viability of water electrolysis still hinges on the availability of durable earth-abundant electrocatalyst materials and the overall process efficiency. This review spans from the fundamentals of electrocatalytically initiated water splitting to the very latest scientific findings from university and institutional research, also covering specifications and special features of the current industrial processes and those processes currently being tested in large-scale applications. Recently developed strategies are described for the optimisation and discovery of active and durable materials for electrodes that ever-increasingly harness first-principles calculations and machine learning. In addition, a technoeconomic analysis of water electrolysis is included that allows an assessment of the extent to which a large-scale implementation of water splitting can help to combat climate change. This review article is intended to cross-pollinate and strengthen efforts from fundamental understanding to technical implementation and to improve the ‘junctions’ between the field's physical chemists, materials scientists and engineers, as well as stimulate much-needed exchange among these groups on challenges encountered in the different domains.

## Abstract 2

Replacing fossil fuels with energy sources and carriers that are sustainable, environmentally benign, and affordable is amongst the most pressing challenges for future socio-economic development.

## Graphical abstract

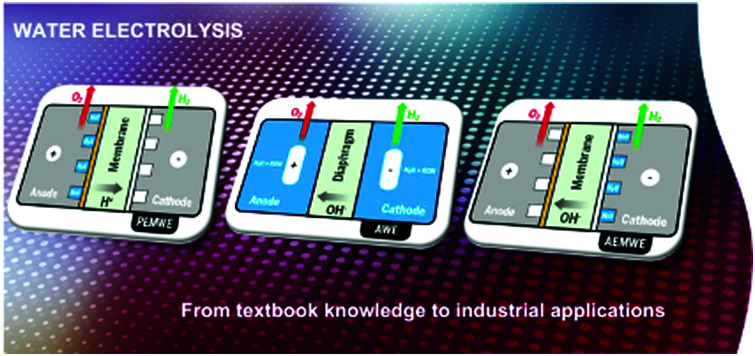

## 1 Introduction

**Fig. 1:**
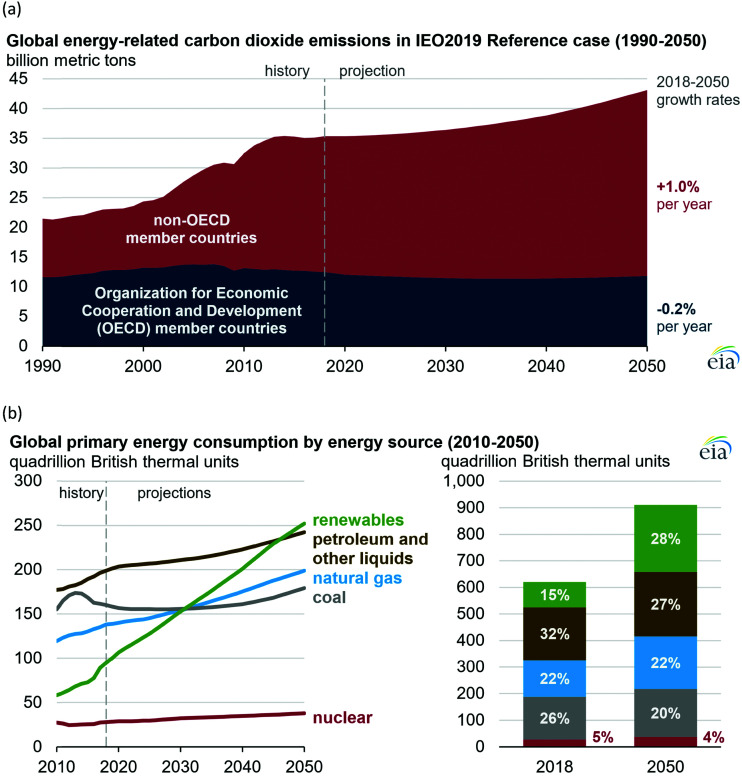
(a) Global carbon dioxide emissions; (b) global primary energy consumption by energy source. Source: ref. 19.

All our environmental problems are compounded with a growing population.^1,2^ Population increases the greenhouse gas production due to increasing livestock husbandry and the gigantic hunger of the population for electrical energy, the production of which releases carbon dioxide (Fig. 1a).^3^ The world energy demand is predicted to double by 2050 and triple by the end of the 21st century.^4^ The accelerated depletion of fossil fuels and ecological consequences associated with their use are a major concern of both policy makers and the public. Thus, the global energy consumption by energy source will have to change drastically in the next decades (Fig. 1b) and scientists and engineers are forced to search for green energy carriers, *i.e.*, produced using zero-carbon renewable energy resources like wind, solar, hydropower or geothermal.^5,6^ Solar energy however suffers from intermittent availability due to regional or seasonal factors – a drawback that makes it difficult to adapt to the demands of a modern society.^7,8^ Energy conversion, and in particular energy storage, will therefore be an essential pillar in allowing energy to be harvested where and when needed. Compared to electrochemical storage (*e.g.*, in Li-ion batteries), storing energy in the bonds of molecules such as hydrogen does not suffer from self-discharge (energy loss) during the storage period. Hydrogen (H_2_) has future potential as an energy carrier due to its high energy content and harmless burning products. The energy can be subsequently regenerated by fuel cells. In addition, H_2_ could be easily integrated to existing distribution systems for gas and oil.^9^

However, hydrogen can only be seen as a green energy carrier when its generation is not fraught with the release of greenhouse gases. Hydrogen is currently produced almost entirely from fossil fuels, with 6% of global natural gas and 2% of global coal being used for hydrogen, and therefore it is responsible for CO_2_ emissions of around 830 million tonnes of carbon dioxide per year.^10^

A sustainable energy industry based on hydrogen is currently only being implemented slowly by society. National and international efforts are necessary and are already ongoing to pave the way for hydrogen as the main energy carrier of the future.^10^ Several countries and regions now have ambitious targets for the share of electricity coming from low-carbon sources, with South Australia aiming for 100% by 2025, Fukushima Prefecture by 2040, Sweden by 2040, California by 2045, and Denmark by 2050.^10^

Splitting of water into hydrogen and oxygen by exploiting solar energy transforms water into an inexhaustible and environmentally friendly fuel source.^11–16^ Among the known strategies, water electrolysis is the easiest technology to be transferred to large-scale industry.^17,18^

**Fig. 2:**
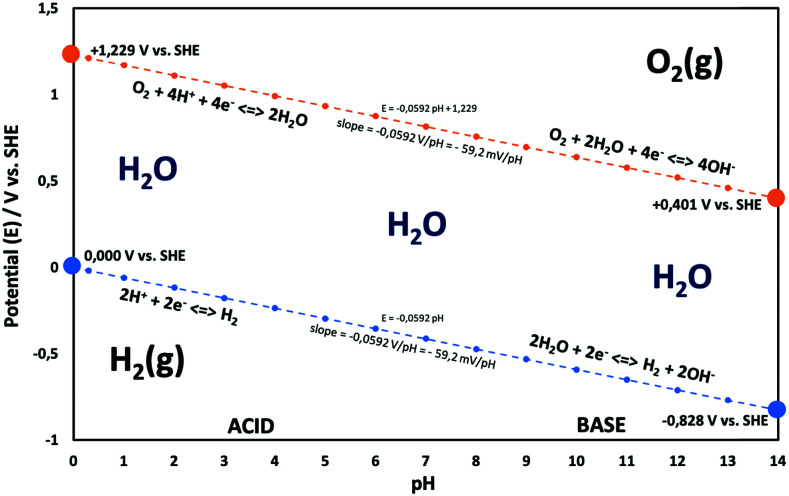
Water electrolysis electrode potentials with pH at standard conditions. Reproduced with permission from ref. 86. Wiley 2020.

Electricity-driven water splitting comprises two half-cell reactions, the hydrogen evolution reaction (HER) and the oxygen (O_2_) evolution reaction (OER). Oxygen-evolving electrodes contribute mainly to the surplus of cell voltage which must be applied in addition to the theoretical decomposition voltage (1.229 V in standard conditions) of water electrolysis. Fig. 2 shows the Pourbaix diagram of water (potential pH diagram at standard conditions). HER and OER fundamentals are discussed in Section 2 of this review.

Besides alkaline water electrolyser (AWE),^17^ proton-exchange membrane (PEM) water electrolysers^20^ (PEMWE) and most recently anion-exchange membrane (AEM) water electrolysers^21^ (AEMWE) are currently well-developed and commercially available. Section 3 gives an overview of the water electrolyser technologies. Unlike AWE, PEMWE is compatible with frequent changes of the current load, a crucial characteristic when converting energy from a renewable source of electricity. All these technologies have their advantages and disadvantages, and the challenges for reducing the costs of produced hydrogen really are technology-depending. The membrane material represents an enormous cost driver for PEM technology;however, for PEMWE developing earth-abundant, durable electrode materials capable of replacing noble electrodes is currently the most effective way to reduce capital costs (capital expenditure, CAPEX). For AEMWE electrolysers, the maintenance costs caused by the poor stability of the membranes are the main cost factor. To bring clarity here, the different approaches are compared based on a (in-depth) techno-economic and SWOT (Strengths, Weakness Opportunities, and Threats) analysis (Sections 4 and 14), while Section 5 focuses on the materials of these water electrolysers’ technologies.

The usefulness of electrocatalytically-driven H_2_/O_2_ production stands and falls with complementary properties that must be met by the electrolyzer system. The efficiency, the rate and the stability of the system and its core materials are pivotal to its practical implementation. From an engineering standpoint, a system operated at a low overpotential (and low rate) would exhibit a high efficiency (low operating cost), combined with a low productivity (little hydrogen production in comparison to the total cost of the construction: high capital cost), maintenance and operation of the system, so that it will not always be economically competitive. On the contrary, a system running at higher rate (and a lower efficiency), could be more economically-viable per produced kg of hydrogen. In addition, the system stability must be considered, as long-lasting electrodes would enable to lower the maintainence/replacement costs. So, it is not only the electrodes/electrocatalysts’ efficiency which drive the electrolyser's practicability. However, one can admit that more efficient electrodes/electrocatalysts are still desperately needed; the electrocatalytic efficiency is directly determined by the overpotentials (*η*) occurring on both half-cell sides.^22,23^ It is therefore not surprising that optimisation of hydrogen-evolving and oxygen-evolving electrodes remains a hard-fought battlefield on which scientists and engineers currently cavort.

Especially of interest is the development of OER electrodes that consist of cheap, non-noble earth-abundant elements capable of replacing the noble, rarely occurring components such as iridium (Ir), platinum (Pt), or ruthenium (Ru) known to be highly active oxygen evolving electrodes. We would like to point out here that the periphery of the as-prepared electrode, *i.e.*, the as prepared catalyst, is usually not identical with the active catalytic surface that is formed under operation. Today's materials discovery strategies based on first-principles calculations (*e.g.*, DFT), machine learning, and optimisation approaches (*aka* the materials-by-design approach) for reducing the overpotential for metal-based and metal-free OER and HER electrocatalysts are evaluated in Sections 6–8.

For a general assessment of the quality of water electrolysis electrodes, it is not enough to consider only the pure electrocatalytic performance of the materials from which the electrodes are made. The number of active sites and the activity of the active site (the latter being defined as the intrinsic catalyst activity^24^) of the exploited materials play a major role in terms of the overall catalyst's performance and are influenced by particle size,^25^ by engineering catalyst morphology,^26^ and by surface reconstruction into more active site species.^27–31^ For tailored electrocatalytic properties and advantageous mass-transfer behaviour, optimised electrode preparation techniques and options for post-treatment of electrode materials and ready-to-use electrodes are essential (Section 9).

Water-splitting approaches that can be classified as being heterogenous catalysis are the most promising. Molecular catalysts originally intended to support photocatalytic water-splitting are also gradually implemented in water electrocatalysis (heterogenisation of molecular catalysts). Metal complexes can help not only in the understanding of the sequential steps of water oxidation but also have promise for their putative integration in functional devices, particularly for the hydrogen production reaction^32^ (Section 10).

A knowledge-based optimisation of electrodes would have been impossible without the development of ever finer characterisation methods, some of which being applied under potential control (*i.e. in situ* or even *operando*). X-ray photoelectron spectroscopy (XPS), Extended X-Ray Absorption Fine Structure (EXAFS), and X-ray Absorption Near Edge Structure (XANES) analysis helped to understand the characteristics that affect OER activity and are therefore vital for determining the OER mechanism and developing OER electrocatalysts. Often catalysts that appeared to be initially promising have failed when used at conditions approaching normal industrial operation. To evaluate the value of electrode materials or complete water-splitting devices in terms of practical application, intensive *ex situ*/*in situ* testing and durability (long-term) testing under conditions ranging from classical laboratory operating settings (current density loads, temperature, load change behaviour) to industrial settings are an indispensable prerequisite. It is widely agreed that dynamic conditions (*e.g.*, cycling the electrode potential or current density) accelerate the degradation relative to galvanostatic testing which led to the so-called accelerated durability tests (ADT).^33,34^ In terms of the durability of fuel cells, steady progress has been made towards the Department of Energy (DOE) MYRD&D 2020 target of 5000 hours with less than 10% loss of performance (with an ultimate target of 8000 hours at 10% loss of performance).^35^ The challenge today is to have PEMFCs (proton exchange membrane fuel cells) for heavy-duty vehicles with 40 000–50 000 hours of service.^36^ PEM (proton exchange membrane) electrolyser components also degrade upon usage, but this is less of a concern as ∼60 000 hours lifetime has been reported in commercial stacks without any detected voltage decay.^37^ To provide evidence-based scientific support to the European policymaking process, EU harmonised test protocols have been developed.^38,39^ The characterisation methods of water electrolysers and their constitutive materials are addressed in Section 11.

Thinking outside the box can be worthwhile if the problems of classical approaches that have existed for years cannot be completely or not satisfactorily solved. Non-classical water-splitting approaches such as ultrasound and magnetic field-assisted water electrolysis^29^ are reviewed in Section 12.

In order to avoid expensive pre-treatment of the water (depending on country specifications), the electrolyser technology must be adaptable to the water that is directly available in nature. The savings originating from not using a purification step could however be counterbalanced by the depreciated performances of the water electrolyzer when fed with impure water. Problematic ingredients of water from the sea, lakes, and rivers as well as wastewater pose major challenges for electrodes and membranes. This research field is addressed in Section 13, while market and cost issues are focused on in Section 14.

Water splitting is a research field of activity that is developing at breath-taking speed. Consequently, the number of papers that can be assigned to water splitting published per time has increased dramatically. This area of research must not lose sight of a critical review of the research approaches. The authors try at every point in the article to identify opportunities in approaches – including around basic research such as electrode development, approaches to developing theoretical explanations, and the technical implementation of newer research approaches – and perhaps even to uncover possible wrong turns.

## 2 Basic concepts in OER and HER electrocatalysis

A typical water electrolyser comprises three (main) components: an electrolyte, a cathode, and an anode. Energy supplied with an externally generated voltage that must exceed the equilibrium voltage of water splitting, decomposes water molecules into hydrogen gas in the hydrogen evolution reaction (HER) at the cathode and oxygen gas in the oxygen evolution reaction (OER) at the anode. The net reaction of water electrolysis is 2H_2_O → 2H_2_ + O_2_. The standard equilibrium voltage of the water electrolysis cell is *U*_0_ = 1.229 V (at *T* = 298 K, *P* = 1 atm and pH 0). It is related to the standard reaction Gibbs energy by the well-known relation Δ*G*^0^_R_ = −*nFU*_0_, with the Faraday constant *F* = 96 485 C mol^−1^ and the number of electrons converted per H_2_ molecule, *n* = 2. Here, *U*_0_ = *E*_0,OER_ − *E*_0,HER_ is the difference between standard electrode potentials at anode, *E*_0,OER_, and cathode, *E*_0,HER_, that would be measured under standard conditions, if the net reaction rate and the corresponding cell current density were exactly equal to zero. When conditions deviate from the standard conditions, the equilibrium voltage, *U*_eq_ is determined by *U*_0_ and an additional term that generally depends on the temperature as well concentrations, activities or partial pressures of reactant and product species, as described by the Nernst equation. In order to achieve a certain decomposition rate (current density) the cell voltage *U* should exceed the equilibrium voltage (*U* > *U*_eq_). Because of the sluggishness of the OER, a significant departure (*U* − *U*_eq_ > 0.5 V) is required in order to attain technically relevant current densities on the order of 0.3 to 10 A cm^−2^, depending on the water electrolysis technology employed.

The electrode potential values required to achieve a certain net rate or current density of the water decomposition reaction depends strongly on the pH value. Oxygen-evolving electrodes, in particular, incur a significantly higher overpotential (*η*_OER_ = *E*_OER_ − *E*_eq,OER_) under neutral or acidic conditions than under alkaline conditions.^40,41^ The overpotential at hydrogen-evolving electrodes (*η*_HER_ = *E*_HER_ − *E*_eq,HER_) is higher in neutral and alkaline environments.^42^ The overall water decomposition reaction is the reverse process of the water production reaction in a hydrogen fuel cell, in which H_2_ flows around the anode to be oxidised in the hydrogen oxidation reaction (HOR) and O_2_ flows around the cathode to be reduced in the oxygen reduction reaction (ORR). The maximal terminal voltage *U*_t_ (under equilibrium condition at zero current) or equilibrium voltage of an oxyhydrogen fuel cell is identical to the minimal decomposition voltage of water electrolysis (*U*_t_ = *U*_0_ = 1.229 V under standard conditions). Depending upon the solution pH, different OER and HER water electrolysis half-cell reactions and different HOR and ORR fuel-cell half-cell reactions can be defined.^43^

Under acidic conditions for the OER, two water molecules are converted into four protons (H^+^) and one oxygen molecule. In neutral and alkaline media, the OER involves the oxidation of four hydroxide ions to water. The direct oxidation of hydroxide anions on the electrode might be favoured over that of neutral water molecules, due to attractive interactions between anions and the positive anode – an effect that depends on the surface charging relation of the (supported) electrocatalyst material and the corresponding local reaction environment established.^44–46^

The HER takes place at the negatively-charged cathode. When hydrated extra protons (hydronium ions) are available in significant concentrations in acidic electrolytes, they are the preferred reactant and are reduced, eventually leading to the formation of a hydrogen molecule from two protons and two electrons. However, in neutral and alkaline media, the concentration of protons is negligible compared to that of water, and the reduction of H_2_O molecules prevails. The HER requires two-electron transfer steps, whereas the OER comprises at least four steps, typically proton-coupled electron transfer (PCET) steps, and three reaction intermediates. The more complex reaction pathway of the OER causes a higher overall activation energy, thus resulting in the more sluggish reaction kinetics. Rationalising the complex reaction behaviour of the OER requires detailed mechanistic models and analytical concepts that will be discussed below.^47,48^

The energy efficiency of the water splitting reaction is defined as the ratio of the thermodynamic equilibrium cell voltage, *U*_eq_ = 1.229 V in standard conditions, to the real cell voltage, *U*_cell_, measured at *T*,*P*,*j* operating conditions. *U*_cell_ is the sum of the thermodynamic equilibrium cell voltage, the overpotentials that stem from charge transfer reactions on anode and cathode sides, and ohmic losses due to ion migration in the electrolyte phase, and from other parasitic losses, *e.g.*, *via* convection or diffusion or other metallic cell components, *i.e.*, overall 
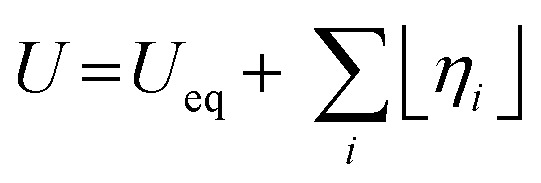
.^49^ For example, when *U*_cell_ = 1.8 V, this yield an efficiency *e* = 1.229 V/1.8 V × 100 = 68.3% at the operating conditions of interest (assuming that the effect of operating temperature and pressure on *U*_eq_ can be neglected). Note: The specific energy consumption at *U*_0_ (under standard equilibrium conditions) is equal to 2.94 kW h m^−3^ H_2′_, and the one at *U*_cell_ = 1.8 V (a usual PEMWE cell voltage at beginning of life; *j* = 1 A cm^−2^, 60 °C)), is equal to 4.31 kW h m^−3^ H_2_. The efficiency can also be defined as *ε* = 2.94/4.31 × 100 = 68.3%.^23,50^ Although the assessment of energy consumption and efficiency of an electrolyser cell can be quite easily determined by the overvoltage beyond the theoretical equilibrium voltage (assuming 100% faradaic efficiency), knowing the overpotential losses from different cell components (anode, cathode, electrolyte) and energy loss processes (HER, OER, ion migration, diffusion) is a more precise methodology, that would enable to isolate/mitigate the cell limitations.

In the following, we will briefly discuss basic concepts and important parameters that determine the response functions between the electrode potentials, *E*_OER_ (anode) or *E*_HER_ (cathode) or total electrode overpotential, *η*_OER/HER_ = *E*_OER/HER_ − *E*_eq,OER/HER_, and the cell current density, *j*. This response function, also referred to as polarisation curve, is the characteristic function of an electrochemical cell.

The condition of electrochemical equilibrium for individual electrode configurations or electrochemical cells can be developed from the very basic concepts of electrochemical thermodynamics that are well-covered in numerous textbooks.^43,51,52^ The interested reader could find a concise treatment of the equilibrium thermodynamics of electrochemical cells in the chapter “Basic Concepts” of ref. 53 and recent extensions for nonequilibrium thermodynamics at high currents in ref. 54.

The flow of electric current influences the electrode potentials at anode and cathode for three reasons. Firstly, the kinetics of charge transfer at electrochemical interfaces is kinetically hindered and thus proceeds at a finite rate. A sufficient overvoltage must be applied to accelerate the charge transfer rate to the value required for achieving the target current density. Secondly, in order to supply electroactive species to the interface at the rate, at which they are being consumed, mass-transport limitations or resistances must be overcome. In water electrolysis, relevant transport processes involve charged ionic species, *i.e.*, hydronium ions or hydroxide anions, and the voltage loss incurred by their transport requirements in liquid electrolyte, or polymer electrolyte membranes (AEM or PEM) and ionomer-impregnated electrodes, is described by Ohm's law that implies a linear relation between voltage loss and current density. Thirdly, the electronic conductors present on both sides of the interface cause further ohmic potential losses.

Depending on the value of the electrode potential relative to the equilibrium electrode potential, either the forward reaction or the reverse reaction of each electrode reaction is slowed down or accelerated. In this way, at the anode, the oxidation half-reaction will be accelerated by an electrode potential that exceeds the equilibrium potential of the OER, *η*_OER_ = *E*_OER_ − *E*_eq,OER_ > 0, and at the cathode the reduction half-reaction will be accelerated by an electrode potential that is smaller than the equilibrium electrode potential of the HER, *η*_HER_ = *E*_HER_ − *E*_eq,HER_ < 0. For an electrolysis cell, the terminal cell voltage is increased relative to the equilibrium cell voltage, *U*_t_(*I*) > *U*_eq_, by a sum that includes the absolute values of the electrode overpotentials, terms due to ohmic transport of ions and electrons, and other transport losses.

At practically-relevant current densities of water electrolysis, the evolution of oxygen and hydrogen involve the nucleation, growth, detachment and transport of gas bubbles. These processes cause further increases in the voltage losses associated with the reaction kinetics and ion transport. Bubbles that are attached to the catalyst surface diminish the effective activity and bubbles present in the electrolyte increase the ohmic losses associated with ionic transport in the electrolyte.

As stated above, the overpotential is connected to both the kinetics of charge-transfer and mass-transport. In the most rudimentary form, overvoltage's associated with the electrode kinetics can be related to the current density at an electrode by the Butler–Volmer equation,1
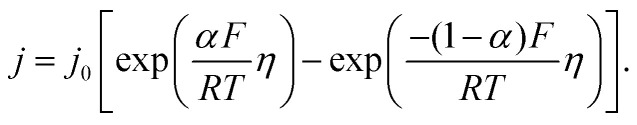
This equation, even though hugely oversimplified, serves to introduce the two crucial parameters that, at a level of phenomenological theory, define the electrochemical properties of an electrocatalyst material: the intrinsic exchange current density, *j*_0_, and the electron transfer coefficient *α*. Here, *R* = 8.31 J (K mol)^−1^ is the ideal gas constant.

It should be noted, that albeit being well-known and widely used, the form of the BV equation provided above, is valid only for single outer-sphere electron transfer processes with complete elimination of any mass-transport effects – conditions that are hardly ever encountered in any technogically relevant electrochemical cell. In the more general case that applies to complex multistep reactions and to conditions with significant mass transport effects, which come into play when the absolute value of the overpotential *η* is large (*η* ≫ *RT*/*F*), the form of the BV equation could be – in principle – retained, but only the term with positive argument of the exponential function needs to be considered at the particular electrode considered (corresponding to the so-called Tafel behaviour). Moreover, due to mass transport effects, local concentrations of reactants (electroactive species) at the electrode surface must be accounted for, which depart significantly from the bulk values or the concentrations provided in external reservoirs. The relations between current density and overpotential in these general cases are:2
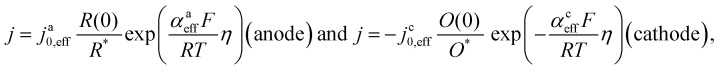
where *R*(0) is the local (meaning: at the electrode surface) concentration of the reduced electroactive species and *O*(0) the local concentration of the oxidised electroactive species, with R* and O* being the corresponding bulk or reference values. While the form of these equations resembles that of the BV equation, they will be the results of detailed derivations based on the microkinetic modelling of reaction mechanisms that accounts for the full complexity of relevant reaction mechanisms and pathways.

Looking deceptively simple in the form of eqn (2), the multistep character of reactions of interest in water electrolysis, especially the OER, will be hidden in two effective parameters (only considering the anode side here): the effective exchange current density, *j*^a^_0,eff_, and the effective transfer coefficient, *α*^a^_eff_. For the latter parameter, we may also introduce the Tafel slope, 
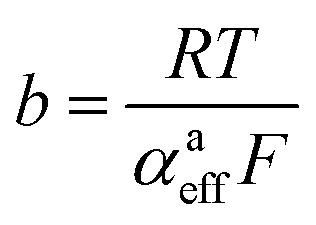
. A microkinetic model of the ORR was solved in ref. 48 and the solution was cast into the form of eqn (2). The formalism was generalised in ref. 47, where the concept of a rate-determining term was presented and applied to the case of the OER. The detailed analyses provided in these recent works unravel the impact of the multistep character of ORR and OER and they reveal the price paid by casting the relation between current density and overpotential into the form of an “effective BV” equation: the two effective parameters *j*^a/c^_0,eff_ and 
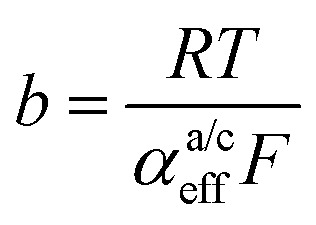
 exhibit strong dependencies on electrode potential (or overpotential), *cf.*Fig. 6 in ref. 48. In the case of *j*^c^_0,eff_ for the ORR, this dependence amounts to a variation by 10 orders of magnitude over the potential range relevant for the ORR. The Tafel slopes needed in eqn (2) vary in the range between 24 mV dec^−1^ at small overpotential and 120 mV dec^−1^ at large overpotential, as revealed by the analyses based on microkinetic modelling and also found in good agreement with experimental observations for ORR^48^ and OER.^47^ Any student or scholar who is beginning to scrutinise the vast experimental literature on the ORR (or OER) is likely to make a confusing experience: reported values for the exchange current density for this reaction seem to be inconsistent and varying by large factors across the literature screened. Ultimately, the multistep nature of the reaction and the oversimplification involved in forcing the complex kinetics of such a process into the form of eqn (2) is responsible for this frustrating experience. Given the strong dependence on the potential of the effective exchange current density and Tafel slope, it is expected that the values found from a Tafel-analysis will be highly sensitive to the range of electrode potentials considered for the fitting of experimental data.

To summarise, the HER and, to a very certain degree, OER are defined by charge transfer kinetics more than by thermodynamic restrictions and contribute mainly to the surplus of cell voltage which must be supplied by an external power source in addition to the theoretical decomposition voltage.^55^ The reactions are not severely mass-transport limited in a well-designed cell, except if bubbles are poorly managed, in particular in AWE.^56,57^ Besides compensation of activation barriers at the anode and cathode side caused by charge-transfer limitations, the overall overpotential results from solution and contact resistances. Activation barriers can be reduced by exploiting improved electrocatalysts suitable for OER and HER, whereas a clever cell design can substantially reduce Ohmic and mass-transport resistances.

Several reviews are discussing mechanisms of OER and HER.^58–61^ Different preparation methods for the generation of the same metal oxide may lead to different metal oxide structures, leading to other pathways for the OER and HER. The following section discusses reaction pathways and mechanistic details of OER and HER for heterogeneous water electrocatalysis. They are not easily transferable to homogeneous catalysts (molecular systems)^62^ or atomically-dispersed catalysts.^63^

### 2.1 Basic mechanisms of the oxygen evolution reaction

Pioneering studies by the groups of Hoare, Bard, Bockris, Conway, and several others^64–68^ showed that the voltage necessary to produce oxygen on a metal surface is related to the redox potential of the metal/metal oxide couple. In other words, even in the case of noble metals, no oxygen can be released from the surface if the corresponding metal oxide is not formed. As was confirmed by recent studies, the OER generally occurs on the hydroxide, oxyhydoxide or oxide layer formed *in situ* on the surface of the electrocatalyst.^69^

**Scheme 1:**
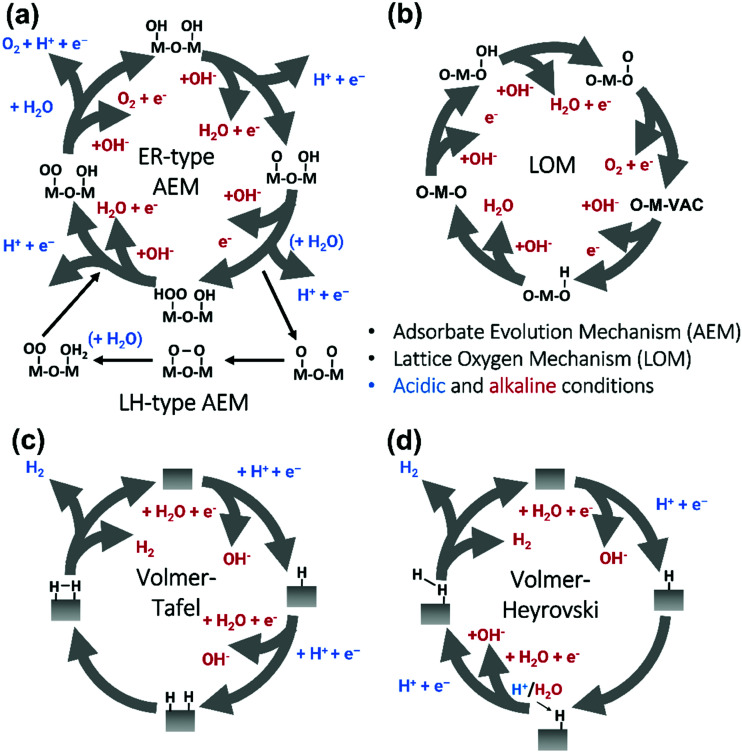
(a) The acid–base and direct coupling adsorbate evolution reaction mechanisms of OER in the acidic (blue) or alkaline (red) medium. (b) The lattice oxygen mechanism of OER in alkaline medium. (c) The Volmer–Tafel HER mechanism on the electrode surface in acidic (blue) or alkaline (red) conditions. (d) Volmer–Heyrovsky mechanism of the HER.

The two generally accepted pathways for the OER in acidic conditions are the Eley–Rideal (ER)-type and the Langmuir–Hinshelwood (LH)-type adsorbate evolution reaction (AEM) mechanisms, illustrated in Scheme 1(a). The difference between the former (*aka* acid–base OER) and the latter (*aka* direct coupling OER) is in the O–O bond formation step.^70,71^ The OER reaction sequence is in all aqueous media initiated by the formation of metal hydroxide intermediates (MOH) subsequently converted to metal oxide species (MO). The formation of dioxygen starting from MO can occur through two different pathways. Either two MO centers are involved, directly splitting off dioxygen, or one MO intermediate reacts with water (acidic condition) or with OH^−^ (alkaline or neutral condition) to give a hydroperoxide species that decompose under release of dioxygen.^72^ The nature of the OER mechanism strongly depends on the nature and structure of the catalyst at stake and any “easy generalisation” appears awkward, the same holding (if not more so) for the kinetics of the reaction. Both ER- and LH-type OER mechanisms involve four steps starting from the transformation of adsorbed OH (OH*) to O*, which results in the oxidation of the metal site.

The ER-type AEM mechanism assumes single metal cation active sites; thus, in the second step, O* undergoes the nucleophilic attack of the active first water molecule, resulting in the formation of OOH*. In the third step, OOH* further oxidises to OO*, which is released in the last step in the form of O_2_, providing the free surface site for the next cycle, starting with the adsorption of another water molecule. The LH-type AEM mechanism, on the other hand, assumes two adjacent metal cation active sites. Therefore, in the second step, OO* is formed between two O* species *via* the direct coupling of two neighbouring oxidised surface metal sites. Likewise, in alkaline conditions, the ER-type AEM involves the evolution from OH^−^ reactant to OH*, O*, OOH*, OO* intermediates to O_2_ product on a single active metal site, while the LH-type AEM assumes that two adjacent metal sites are involved.^73^ As reviewed in ref. 71 the ER-type mechanism is reported for Ru-based catalysts,^71,74^ while there have been reports on LH-type mechanisms for Co-based catalysts,^71,75^

Pathways in AEM assume proton-coupled electron transfer (PCET) for all steps. For catalysts favouring these routes, the OER overpotential becomes pH-independent in the RHE scale, the case reported for Ir oxide catalysts.^76^ In contrast, the lattice oxygen mechanism (LOM) proceeds *via* non-concerted proton–electron transfer steps involving both the metal cation active site and the lattice oxygen. One proposed path for LOM involves five intermediates, *viz.* M–**O**H, M–**O**, M–**O**OH, M–**O**O, and M–ϒ (**O** represents the lattice oxygen), as illustrated in Scheme 1(b). In this picture, LOM is like LH-type AEM because both bypass the OOH* formation step. However, it differs from AEM in generating a vacant oxygen site upon the desorption of molecular oxygen from the surface. The non-concerted proton–electron transfer in LOM gives rise to pH-dependent OER kinetics, the phenomena observed in certain perovskite electrocatalysts^77^ as well as Ni oxyhydroxides.^78,79^ On the other hand, for RuO_2_ (110), the lattice oxygen is not involved in the OER.^80^

### 2.2 Basic mechanisms of the hydrogen evolution reaction

The HER is one of the most extensively studied electrochemical reactions due to its relative simplicity and its direct industrial relevance, not only in water electrolysis but also in chlor-alkali operations. In contrast to the sluggish kinetics of the OER and ORR,^81,82^ the kinetics of the HER on noble metal (platinum group metals, PGM) electrodes are much faster so that practical current densities (>1 A cm^−2^) are possible at a few tens of millivolts overpotential.^83–85^ The only exception is HER in an alkaline media (even on PGM surfaces^42,86^). The first investigations that aimed to clarify the mechanism of the HER on metal-based surfaces focused on nickel and date back to the early 1950s.^87^ The reaction sequence of the HER begins with the adsorption of a proton in case of acidic conditions (M–H^+^) or a water molecule in neutral or alkaline environment (M–HOH), followed by reduction of adsorbed water molecule/proton to form M–H* (and release OH^−^ in case of the reduction of chemisorbed water). From this point onwards, two possible follow-up steps can be distinguished:^88^ (1) the combination of the chemisorbed H_ad_ with another chemisorbed H*, referred to as the Tafel step, which leads to the chemical desorption of H_2,ad_, or (2) electrochemical reaction of the chemisorbed proton with another proton or water molecule from solution, referred to as the Heyrovsky step, followed by further electrochemical discharge and desorption of H_2_. The former sequence of steps corresponds to the Volmer–Tafel mechanism^89^ whereas the latter is known as the Volmer–Heyrovsky mechanism.^90,91^

Scheme 1(c) and (d) illustrates the two pathways for the HER under acidic and alkaline conditions, *i.e.*, the Volmer–Tafel and the Volmer–Heyrovsky mechanisms, respectively.^92,93^ Both pathways start with the Volmer step, in which an electron transfer from the electrode is coupled with proton adsorption on the catalyst site to form an adsorbed H atom,

3 H^+^ + e^−^ → H* (in acidic electrolyte)

4 H_2_O + e^−^ → OH^−^ + H* (in alkaline electrolyte)

Hydronium ions (H_3_O^+^) and water molecules are the source of protons in acidic and alkaline electrolytes, respectively. Next, in the Volmer–Tafel mechanism, the Tafel step combines two H* on adjacent sites to form H_2_, *i.e.*,

5 H* + H* → H_2_ (in acidic and alkaline electrolytes)

In the Heyrovsky step of the Volmer–Heyrovsky mechanism, H_2_ is formed *via* direct interaction of the H* atoms with protons (in acidic) and water molecules (in an alkaline environment),

6 H* + H^+^ + e^−^ → H_2_ (in acidic electrolyte)

7 H_2_O + e^−^ → H_2_ + OH^−^ (in alkaline electrolyte)

**Scheme 2:**
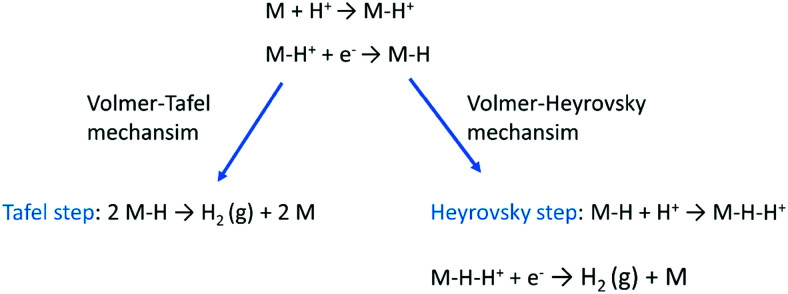
The mechanism of the hydrogen evolution reaction in an acidic medium.

Scheme 2 displays the reaction steps according to both mechanisms for the hydrogen evolution carried out in the acidic regime. The occurrence of one or the other HER mechanism depends on operating parameters, including the pH, the electrode potential, and the nature and structure of the electrode considered.^94^ To date, nickel remains the most popular base metal for HER (and HOR) in an alkaline environment and is under extensive focus by the research community.^95–97^

**Fig. 3:**
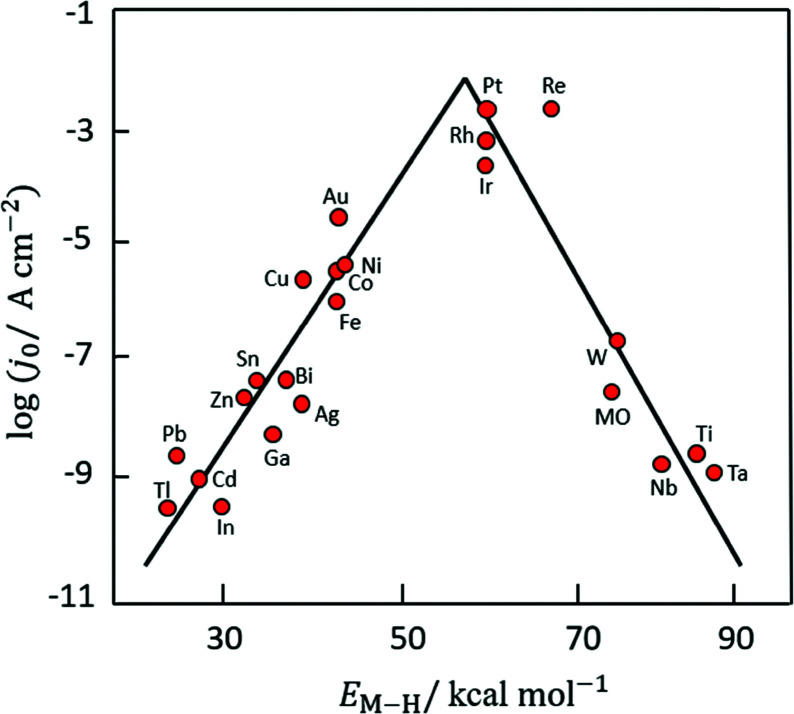
A common phenomenon in chemical catalysis is the volcano relationship between the catalytic activity of a particular reaction on the ordinate (on a log scale) and an activity descriptor on the abscissa. It is found that for a given reaction carried out on a variety of catalysts, the rates on each catalyst can be plotted so that they pass through a maximum. What is plotted on the abscissa varies, but it is always a function that includes a property of the catalyst (*e.g.*, heat of sublimation, bonding strength of a reaction intermediate to the catalyst material). The volcano behaviour of the exchange current density of the hydrogen oxidation reaction *vs.* M–H bonding strength is generally valid for pure metals in acidic solution and was first determined by Trasatti 97. The noble metals Pt and Pd demonstrate exceptionally high activity, with Ni as the most active non-precious metal. Reproduced with permission from ref. 98 Copyright 1972 Elsevier.

Based on Scheme 2, one can easily understand that the M–H* bond strength will influence the catalytic activity of metal towards the HER. On the one hand, a substantial strength is required to support the formation of the M–H* bond, the first step that initiates the reaction sequence (Volmer). On the other hand, too strong M–H bonding is counterproductive, as chemisorbed intermediates or product species will not be easily released from the surface, thereby causing a surface blocking effect. This is the case for reduced Ni surfaces, which bind H_ad_ too strongly,^91^ whereas oxidised Ni surfaces present an intermediate and thus more “optimised” Ni–H bond strength that is beneficial for fast HER/HOR. Investigations confirmed that the catalytic activity toward the HER is correlated with the strength of the interaction between the catalyst surface and adsorbed hydrogen. At low overpotentials (at which HER usually occurs), the slope of the current–voltage curve is proportional to the exchange current density *j*_0_. Exchange current densities for the HER on pure metals in acidic media have been reported in a plethora of experimental studies, as collected and famously reported by Trasatti in ref. 98. Plotting these values against the metal-hydrogen bond strength revealed a characteristic behaviour that is known as the “volcano” curve (Fig. 3) and expected based on the Sabatier principle:^99,100^ the HER activity increases to a peak value obtained at medium bond strengths (Pt, Rh, Ir) then decreases again towards higher bond strengths. It should be mentioned at this point that the *Trasatti volcano* is only applicable to acidic media and requires an exchange current density correction for Pt.^101^

Pt group metals (PGM) are the most effective materials to catalyse the HER in acidic and alkaline conditions. Among the non-PGM class, sulphides, phosphides, carbides, and borides have shown promising HER activity, and these will be reviewed in the forthcoming sections.

In alkaline electrolysers, non-precious-transition metal oxides such as Co-, Ni-, and Fe-based materials are stable and active towards the OER (see Section 7), with Ni-based oxyhydroxides (NiO_*x*_H_*y*_) being among the best-performing OER catalysts under alkaline conditions. *In situ* surface spectroscopy study by Diaz-Morales *et al.* suggests a pH-dependency of the OER at NiO_*x*_H_*y*_ materials, based upon which a mechanism was proposed that involves a non-concerted proton–electron transfer step. pH-Dependence at RHE scale is linked to surface deprotonation and formation of negatively-charged surface oxygen species, NiOO–, that are involved in the OER. DFT studies of the OER mechanism on β-NiOOH(0001) revealed the involvement of lattice oxygen in the mechanism; however, despite the experimental observation, PCET was assumed for all steps. A more recent experimental investigation by Koper *et al.* reported the effect of electrolyte alkali metal cations on the OER activity of these materials:^79^ the interaction of cations with negatively-charged surface oxygen species (NiOO^−^) stabilises cations on the surface. A thorough modelling investigation by Huang *et al.* explains the decrease in OER activity with the increasing effective size of electrolyte cations by a cation overcrowding-effect near the negatively-charged electrode surface.^102^

Incorporating Fe into NiO_*x*_H_*y*_ materials, either intentionally *via* doping or incidentally due to iron ions formed during fabrication or operation of the electrochemical cell and entering the catalyst layer as impurities, significantly increase their OER activity.^103,104^ Controversial explanations were proposed to understand the role of Fe. From the simulation point of view, the controversy can originate at different levels. As an example, the well-known experimental-theoretical investigation by Friebel *et al.* observed the OER dependence on the Fe content and proposed Fe^3+^ as an active site in γ-FeNiOOH(011̄2).^105^ First, the choice of surface termination for the DFT study was explained based on its high activity; however, the (0001) facet is known to be the thermodynamically most stable facet; unlike on the high index facet, the mechanism of OER on the (0001) facet involves lattice oxygen.^106^ The calculations were performed in the gas phase; it is, however, known from studies using the DFT+*U* approach that water strongly interacts with NiOOH surfaces.^107,108^ The calculations in ref. 106, were performed at the PBE+*U* level, although it was shown that the PBE+*U* does not correctly describe the electronic structure of NiOOH and significantly underestimates the bandgap of the material.^109^ The γ-phase of FeNiOOH under OER conditions involves intercalated water and ionic species; Friebel *et al.* approximated the γ-phase with 50% dehydrogenated β-phase to obtain an average oxidation state of +3.5 consistent with γ-phase. Most importantly, the computational hydrogen electrode scheme was used to generate the OER energy diagram assuming that all steps involve PCET. However, there is experimental evidence against this assumption for this material. For example, similar to the conclusion by Koper *et al.*,^78^ Görlin *et al.* proposed a decoupled proton transfer–electron transfer scheme involving negatively-charged oxygenate ligands generated at Fe centers.^110^ In another study, Trotochaud *et al.* explored the activity-dependence of FeNiOOH on the film thickness. They proposed that Fe induces a partial charge on Ni activating it for the OER.^104^ At variance, Xiao *et al.* presented that O–O coupling at Ni-sites is involved, which requires the synergy from the mixed Ni–Fe site.^111^ Mössbauer spectroscopy study indicated the formation of Fe^4+^, but its role on OER is not clear.^112^ Finally, Qiu *et al.* suggested that Fe in NiFe LDHs acts as an agent that creates higher valence Ni in the created oxyhydroxides under OER conditions, resulting in enhanced OER properties.^113^ These studies suggest that the phenomenon driving the enhancement of the activity of FeNiOOH are probably linked to the interplay between Fe and Ni moieties, even though complete understanding is not fully reached yet.

In addition to NiO_*x*_-based electrocatalysts, bimetallic cobaltite oxy-/thio-spinels^114,115^ as well as perovskite oxides with tuneable electronic structure properties, have recently attracted interest due to their promising OER activities.^27^ For the latter class, the OER proceeds *via* the LOM, and the structural changes under OER conditions lead to the formation of an oxy(hydroxide) surface layer that is highly OER-active.^77^ In contrast to the conventional explanations of OER activity based on the correlations in adsorption energies of intermediates, understanding the LOM mechanism on perovskites requires identifying correlations with surface reconstruction phenomena.

### 2.3 Challenges for theory and computation

In the context of PEMWE technology, the key practical question being asked is: how can the precious metal loading, concerning mainly Ir as a key component, and the corresponding cost of this scarce material in anodes for the OER be drastically reduced while meeting or exceeding performance, durability and lifetime targets?^116–119^ This specific problem, entails two general, closely-intertwined challenges: (i) to find a catalyst material with an ideal combination of high intrinsic electrocatalytic activity and chemical stability that is also inexpensive and environmentally-benign,^20^ and (ii) to optimise the design of the porous composite electrode that accommodates the catalyst^120^ to maximise the statistical utilisation (on a per-atom basis) of the catalyst and ensure uniform reaction conditions over the entire catalyst surface dispersed inside of this medium. Using experimental and modelling-based analyses of electrocatalytic performance and stability, candidate materials to be used as electrocatalyst and support can be identified. These pre-selected materials can be passed on for in-device testing and fabrication scale-up.

There is thus an intricate interplay of intrinsic catalytic activity and multicomponent transport that is controlled by the selection and specifically tuned properties of catalyst and support materials and the electrode design. Theory and computation are needed to contribute fundamental understanding as well as modelling-based analytical tools to deconvolute and quantify different voltage loss contributions caused by ohmic transport of ions (hydronium or hydroxide ions), electrocatalytic activation, and gas removal from active catalyst surface sites. Complicating matters, all of these processes and associated voltage losses are affected by the dynamics of gas bubble nucleation, growth, coalescence, detachment, and transport.^121,122^ In particular, the latter aspect calls for game-changing progress in the rational design of gas-evolving electrodes with rapid gas bubble detachment and removal, as emphasised by Zeradjanin^123,124^ and Bernt *et al.*^20^

This section of the review article is not intended as a detailed review of the field of theory and computation in electrolysis research. Recent reviews and perspectives with a strong emphasis on atomic-scale simulations exist.^125–133^ The Sabatier principle and the volcano-type relationships that result from it, are concepts borrowed from the field of heterogeneous catalysis (*i.e.*, dealing with solid-gas interfaces). Early atomistic simulations in the field of electrocatalysis (*i.e.*, dealing with solid-liquid electrolyte interfaces) have essentially transferred these concepts over from heterogeneous catalysis. Such approaches have been remarkably successful^126,127,129,134^ considering the fact that they neglected essential physics of electrochemical interfaces. Aspects of surface morphology, *i.e.*, addressing differences between idealized flat surfaces and those that have terraces and kinks are similar for heterogeneous catalysis and electrocatalysis. However, in the latter field a detailed theoretical understanding of the (sub-)nanoscale structure and properties of the electrochemical interface is needed.^45,46,148^ To evaluate, compare and select electrocatalyst materials for the OER (or the HER), it is of utmost importance to understand the local reaction environment that prevails at the interface (reaction plane) when the electrolysis cell is operated at a certain voltage. This local reaction environment is affected by the atomic-scale surface configuration of the catalyst, by the potential and pH-dependent formation of surface oxides (as rationalized in the form of Pourbaix diagrams),^108^ by the surface charging relation^46^ and by specific ionic effects.^102,108^

This section provides a perspective on what this field currently can or cannot contribute and along which directions it is advancing. It will survey efforts to devise a theoretical-computational framework that comprehensively rationalises potential-induced surface charging phenomena, local reaction conditions, and microkinetic mechanisms at heterogeneous electrochemical interfaces and links such efforts with the modelling of transport and reaction in porous composite electrodes.

### 2.4 What to expect from theory and computation in the field of water electrolysis

Theory and computation can support the development of highly-performing and durable electrocatalyst materials and electrode media for water electrolysis in the following three areas: (i) devise a set of theory-based activity and stability descriptors to steer efforts in materials discovery and inverse design,^134–137^ (ii) employ efficient computational tools based on artificial intelligence to rapidly search the complex parameter space^138–144^ in conjunction with advances in autonomous or self-driving laboratories^144–146^ and (iii) implement smart approaches in electrode design and fabrication based on knowledge of reaction mechanisms, pathways and local reaction conditions.^147,148^

The local reaction environment (LRE) that prevails at the catalyst's surface under real operating conditions plays a central role in this endeavour. On the one hand, it is crucial to understand how the LRE depends on the operating regime, *i.e.*, cell current density or cell voltage, and the externally-controlled parameters such as pressure and temperature – this is the challenge that porous electrode theory and modelling must address. On the other hand, the impact of the LRE on electrocatalytic reaction mechanism and pathways as well as kinetic rate constants must be rationalised – this task calls for concerted efforts in interface theory, microkinetic modelling, and quantum-mechanical (DFT-based) calculations of energy and interactions parameters that control surface adsorption states as well as reactive transformations between them.

Once the optimal LRE has been determined by connecting these aspects, electrode design and fabrication will aim to provide these conditions uniformly over all available catalyst surface sites dispersed in a porous composite electrode. The departure from optimally-uniform conditions can be quantified by calculating the effectiveness factor of catalyst utilisation, as demonstrated for cathode catalyst layers (CCLs) in PEM fuel cells.^149–153^ For CCLs in PEM fuel cells, well-established hierarchical models describe the interplay of transport and reaction at different structural levels, *viz.* (i) single pore, (ii) mesoscopic agglomerate of Pt nanoparticles, carbon-based support and dispersed ionomer aggregates, and (iii) macroscopic porous composite layer. This interplay determines distributions of reaction conditions and rates and the net activity of the CCL for the ORR.^53,154^ Using these approaches, the effectiveness factor of catalyst utilisation was found to lie in the range of 5 to 10%; it decreases with increasing current density of operation, corresponding to higher non-uniformity of reaction conditions and rate distributions.

An overall effectiveness factor of Pt utilisation in PEM fuel cells that accounts for statistical utilisation effects was determined to be even smaller, lying in the range of 1 to 4%.^153^ For PEMWE, a similar model-based calculation and assessment of effectiveness factors in Ir-based anodes have not been made, as electrode models that account for a hierarchy of transport and electrokinetic effects in porous electrodes have not been developed to a sufficient level of sophistication. However, it can be expected that the overall effectiveness factor of Ir-utilisation will be about as small, most likely even smaller, due to the less extensive efforts in CL design for PEMWE and to the fact that (at least present) IrO_2_ OER catalysts are unsupported and of larger particle size than present PtM/C-based ORR catalysts in PEMFCs.

The OER activity in the PEMWE anode is highly dependent on electronic interactions between the electrode material and reaction intermediates. Binding energies of reaction intermediates can thus be employed as viable descriptors for the comparative assessment or “screening” of electrocatalyst materials in terms of their activity for the OER. These energies can be calculated with quantum-mechanical simulations based on density functional theory (DFT).^155–158^

However, other effects related to the electrolyte composition, *i.e.*, the type of solvent and the types and concentrations of ions, must be factored in when attempting to rationalise or predict catalytic activities computationally. These effects determine the local surface state and the near-surface conditions in the electrolyte and thereby exert crucial impacts on electrocatalytic activities of OER and HER.^79,159–165^ DFT-based studies rationalised the importance of cation effects on the HER activity of transition metal electrodes,^166^ and more recently for the OER activity of oxide electrodes.^102,108^

### 2.5 Understanding of the local reaction environment

In electrochemistry, theory and simulation of the structure and dynamics at electrified interfaces between a solid electrode and an electrolyte are of central importance.^167^ The main challenges are concerned with understanding how the metal-based electrode material, the water-based electrolyte, and the complex boundary region in-between these two media impact the energetics and dynamics of adsorption and charge-transfer processes, as considered in a recent review.^195^ Specific questions in this context focus on the following aspects: (i) how do adsorbed intermediates determine or affect pathways of multistep reactions and reactivity^125^ (ii) How do solvent species and ions in the near-surface region modulate interfacial properties and local reaction environment?^148^

The theory of electrified interfaces^168–170^ is closely interwoven with theoretical electrocatalysis and charge-transfer theory.^171–173^ It draws upon large inventories of condensed matter physics, surface science, heterogeneous catalysis, and chemical kinetics.

First-principles computational methods in electrochemistry, with density functional theory (DFT) at their core, strive to decipher the complex relations among the atomic structure and composition of an electrocatalyst material, the energetics, and the reaction kinetics of electrochemical processes. Important steps reveal how surface impurities and chemisorbed species, including reaction intermediates, affect the pathways of multistep reactions and how solvent molecules and ions modulate interfacial properties and the LRE. Theoretical and computational approaches are required to provide distributions of the electric potential, ion concentrations, and solvent orientation or alignment in the near-surface region of the electrolyte that is termed the electrochemical double layer. The key response function or fingerprint of a particular interface configuration is the surface-charging relation, *i.e.*, the relation between the excess surface charge density at the metal denoted *σ*_M_, and the metal phase potential, *ϕ*_M_, as explored in ref. 44–46.

Various innovative catalyst designs have helped improve Ir-based catalysts, the most important element for PEMWEs. Ir–Ir oxide core–shell concepts,^174^ alloys/bimetallic mixed oxides^175^ and inexpensive support materials^176,177,1428^ that prolong the lifetime^178^ have been explored. The crucial idea is to enhance the IrOx nanoparticle dispersion and the ratio of the active surface to the total mass of catalyst.^179^

For supported catalysts, the mechanism and strength of bond formation between nanoparticle and support material must be investigated. The bond strength between these subsystems can be tuned by support doping. Electrochemical conditions at the interface are modulated by the size, shape, and density of nanoparticles on the support. For systems of IrO_2_ nanoparticles deposited on antimony-doped tin oxide (ATO), a significant increase in OER activity has been observed.^176,178,1428^ This gain in OER activity cannot simply be explained as a geometric surface area enhancement effect achieved with the nanoparticle dispersion of the catalyst.^179–181,1420^ Understanding the impact of the oxide support's physical properties on the nanoparticles’ electrocatalytic activity is of crucial importance in this context. Explanations found in the literature often invoke a so-called metal support “interaction”.^178,182–185^ Charge transfer properties at the junction between active catalyst particle and electronic support may also be affected by a Schottky-type barrier. This resistive effect could exert a significant impact on the electrocatalytic activity.^186^

The origin of this MSI effect has remained poorly understood and thus controversial.^178,183^ Electronic equilibration in the catalyst-support system is supposed to play an important role.^183–185^ However, a consistent explanation should also account for simultaneous electrochemical equilibria at interfaces between metal, support material, and electrolyte.^187^ To date, the complex problem of the coupled electronic and electrochemical equilibria at the heterogeneous particle-support surface has not been solved.

### 2.6 Theoretical-computational workflow to decipher the OER

Over the last two decades, the DFT-based method, known as the Computational Hydrogen Electrode (CHE),^82^ has found wide application in the electrocatalysis community as a convenient tool to identify activity trends within a certain class of catalyst materials, including those for transition metals, alloys, or oxides for the OER,^135,189,421^ and the HER.^190–192^ Despite its assumptions and drastic simplifications to the real electrocatalytic system, this scheme has also been the standard approach to determine the stable interface structure under varying electrochemical environments, *i.e.*, for generating surface Pourbaix diagrams under the OER/HER conditions,^108,193,194^ as well for the identification of active sites^105^ and the mechanistic understanding of reaction mechanisms.^125^

**Fig. 4:**
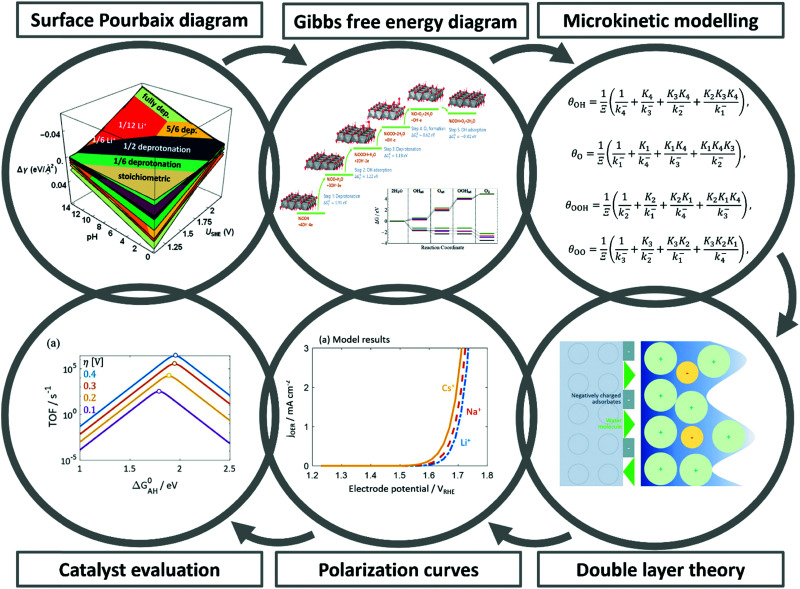
A theoretical-computational workflow to decipher the OER. Step 1. A DFT-based grand-canonical approach is developed to identify the surface adsorption state under relevant electrochemical conditions, i.e., through computing the surface Pourbaix diagram.^108^ Step 2. The OER reaction mechanism is identified and the Gibbs free energy diagram is generated using periodic DFT calculations.^102^ Step 3. A microkinetic model is formulated to obtain an expression for the net reaction rate.^102^ Step 4. The electrochemical interface model is solved to obtain the metal charging relation.^46^ The fully parameterised approach provides as output mechanistic insights as in Step 5 and 6, *e.g.*, the rate-determining term in the net reaction rate;^47^ a descriptor-based activity assessment for materials screening; and effective parameters like Tafel-slope or exchange current density to use in porous electrode models.^188^

However, the main challenge in theoretical and computational electrocatalysis is to move beyond commonly-made simplifying assumptions of the interface problem, such as those made in the CHE scheme. A systematic workflow to proceed in studying OER/HER is illustrated and described in Fig. 4.^195^ The approach combines DFT calculations with microkinetic modelling and the electric double layer theory to address the major complexities at the interface, including the nonlinear solvent polarisation and ion size effects, chemisorption and induced surface dipole effect and surface charging relation.^46,48,102^ The microkinetic modeling explicitly treats all elementary steps of the reaction and it rationalizes the effects of reaction intermediates and their surface coverages on the effective kinetic rate of the overall reaction, as worked out in detail in ref. 47 and 48.

Pinpointing the most stable interface structure under relevant reaction conditions is an essential prerequisite to unraveling the local reaction environment for OER or HER and a requirement in connection with the studies on the kinetic processes involved in surface reactions. Therefore, the first step of the workflow combines the surface slab calculations in periodic DFT with thermodynamics to generate surface Pourbaix diagrams.^193^ Here, the Gibbs energy change associated with the formation of a specific surface configuration is given by, 
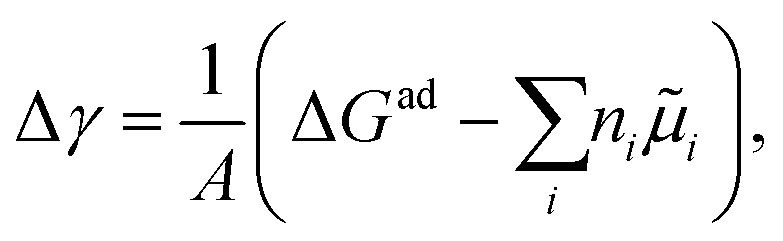
 where, Δ*G*^ad^ is the change in the Gibbs free energy due to adsorption, 
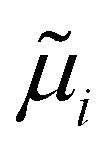
 is the electrochemical potential of ions in the electrolyte, and *n*_*i*_ is the number of adsorbed species of type, *i.* In this picture, the change in the adsorption Gibbs free energy is approximated by, Δ*G*^ad^ ≈ Δ*E*^ad^ + ΔZPE − *T*Δ*S*. Here, Δ*E*^ad^ is the adsorbate binding energy calculated from DFT; ΔZPE, and *T*Δ*S* are the zero-point energy entropy correction terms. ZPE is calculated from the harmonic oscillator approximation of adsorbates, and the total entropies for solvent are typically adopted from standard thermodynamic tables, while only the vibrational entropy contributions are accounted for the adsorbates.^196^

The grand-canonical variant of the CHE assumes that the electrode and the electrolyte are thermodynamic reservoirs for electrons and ions, respectively, whereas the reference system typically corresponds to the standard hydrogen electrode (SHE). At standard conditions, molecular hydrogen in the gas phase is in equilibrium with the solvated proton and the electron, 
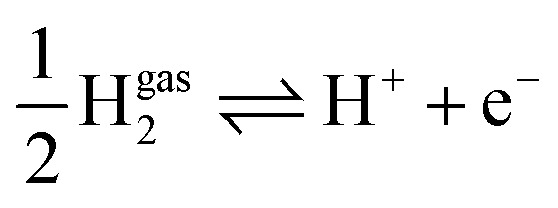
.

Therefore, for a proton-coupled electron transfer (PCET) step in thermodynamic equilibrium, the corresponding chemical potential of hydrogen in the gas phase is equal to that of a proton–electron pair.^82^ This way, one could refer the potential to the SHE or RHE scale and use the calculated gas-phase energy of molecular hydrogen to avoid the calculation of the proton solvation energy in water,
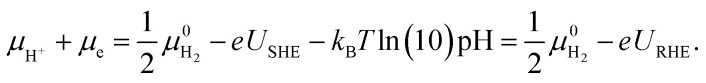
Therefore, the electrode potential, *U*, and pH enter the equilibrium expression of Δ*γ* to account for deviations from the standard conditions. In thermal equilibrium, the most stable adsorbate structure is determined from the lowest Δ*γ* at a given potential and pH; hence the Pourbaix diagram is constructed as shown in the first step in Fig. 4. Besides applying the CHE in the PCET processes, it can be applied to any solvated ionic species for which the standard potential exists.^197^

The second step of the above scheme entails calculating the Gibbs free energy change of each elementary step of the OER/HER and constructing the most favourable reaction pathway under standard conditions. The reference surface structure for this step should be obtained from the output of the first step. For quasi-equilibrium conditions at zero overpotential, all reaction intermediates forming under electrochemical conditions should have higher energy than the reference surface.^198^ The theoretical overpotential is then obtained from the step with the highest value of the reaction Gibbs energy,
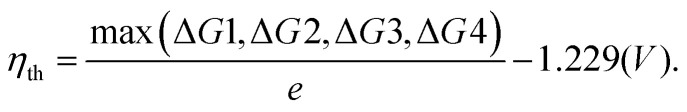


So-called Volcano-type plots, based on the Sabatier principle, can be obtained by plotting *η*_th_ as a function of a simple descriptor like the adsorption energy of the critical intermediate^132,410^ For the OER, the adsorption energy of OOH* and OH* intermediates are linearly correlated, known as the scaling relation. Due to this universal correlation, the binding energy of these intermediates cannot be varied independently on the catalyst surface.^199^

The computational approaches described so far have been employed to rationalise the impact of materials modification strategies that aim to break the scaling relation at the interface and thereby reduce the overpotential. Surface alloying or doping the oxide material with a second metal provide different active sites for optimal binding of key intermediates and thus breaks the scaling relation for an increased catalyst activity. In a recent theory-experiment investigation, the cationic substitution of IrO_2_(100) with Ni was reported to enhance the OER activity of the catalyst.^135^ Rossmeisl and co-workers have shown this effect on Ru by incorporating Ni or Co into the surface.^126^ Buvat *et al.* reported an activity-dependence caused by the orthorhombic distortion of the tetragonal IrO_2_ due to the mismatch between the substrate and the catalyst thin-film at different temperatures.^200^ Other strategies include altering the electrolyte by adding promoters like cations,^79^ engineering the active site by designing novel catalysts like nano-frames,^201^ or applying interfacial nanoconfinement.^202^

The fundamental challenge for first principles studies of electrocatalytically active interfaces is to exert control over the electrode potential, as the crucial parameter controlling structure and dynamics at the interface region between electrode and electrolyte. The electrode potential is not an explicit variable in DFT calculations within the CHE scheme.^82,155^ The electrode potential determines not only the electronic properties of the electrode but also the surface adsorption state and surface charging effects, the orientation of interfacial water (or, generally, solvent) molecules, the local pH, and ion concentration distributions.^46^ The activation energy of elementary steps typically depends on the electrode potential. However, the CHE scheme and its variants do not account for the dependences of the interface properties mentioned above on electrode potential.^203^ Moreover, the adsorbate-induced dipole field interaction, which is neglected in the CHE, is critical for identifying the rate-determining step of reactions that involve intermediates with adsorption energies sensitive to the interfacial electric field.^204^ Additionally, statistical averaging over many electrolyte configurations should also be considered, as proper accounting for the interaction of the adsorbate with solvent is critical in specific reactions.^205^

Other recently presented first-principles schemes to simulate the local reaction condition at electrified interfaces include extrapolation of the unit-cell in periodic slab-type calculations to infinite size to eliminate finite-size effects on activation and reaction energies of charge-transfer reactions,^206,207^ compensating charge and explicit consideration of a reference electrode to simulate the applied potential,^208^ an explicit treatment of electrified interface with *ab initio* molecular dynamics simulations,^205^ effective screening medium combining electronic DFT with mean-field theories and continuum solvation for the electrolyte region,^209–211^ and grand-canonical density functional theory (GC-DFT) that combines electronic and classical DFT for different regions.^212,213^

However, these methods do not account for polarisation effects induced by chemisorbed partially charged adsorbates and charge delocalisation. At potentials that depart significantly from the nominal potential of zero charge, the electrostatic charging and polarisation properties of the boundary region may respond in a non-linear and, possibly, non-monotonic fashion to changes in electrode potential, invalidating approaches based on linear potential extrapolation. The non-linear charging effects modify surface electronic states, short-range electronic interactions with near-surface species, adsorption strength or orientational ordering of polar solvent molecules.^214^ For ionic and molecular species in the near-surface region of the interface, ensemble averaging and the choice of the water model are critical aspects to consider, which may require using *ab initio* molecular dynamics for this specific region.^107,205^

In the past few years, a concerted theoretical-computational framework for modelling interface properties and electrocatalytic reactions has been developed. It combines DFT-based first-principles calculations, a mean-field type model of the double layer, and a microkinetic model for the multistep kinetics of the particular reaction under investigation. DFT calculations are used by this framework to calculate adsorption energies of intermediates or reaction energies of proton-coupled electron transfer steps in the reaction sequence; moreover, DFT studies yield chemisorption-induced surface dipole moments.^215^ The mean-field model of the double layer considers dipolar effects due to chemisorption of oxygen species, solvent orientational polarisation, and ionic effects in the electrolyte.^46^ A more recent, extended theoretical approach explicitly couples the mean-field treatment of electrolyte effects (including solvent, ionic and electronic degrees of freedom) with electronic degrees of freedom in the metal, which are treated at the level of Thomas–Fermi–Dirac–Wigner theory of inhomogeneous electron gas, and it treats the impact of specific ion adsorption at the level of the Anderson–Newns theory.^44^

The mean-field double layer model yields the local reaction environment (LRE) required for the microkinetic model. In the microkinetic model, the reaction rate of each elementary reaction step is formulated using the Frumkin-Butler–Volmer theory. The microkinetic model is parameterised with conditions that define the LRE, *viz.* reactant and ion concentrations, pH, and electrolyte-phase potential. The reaction free energy of each elementary reaction step is obtained from DFT calculations, with proper modifications such as considering lateral interactions between reaction intermediates.

The coupled approach described in the preceding paragraph solves in a self-consistent manner for (i) the coverage variables for the reaction intermediates, which are obtained from the solution of the microkinetic model under the steady-state condition; (ii) the chemisorption-induced surface dipole moment, using coverages of reaction intermediates obtained in the previous step and the value of the elementary dipole properties to be obtained from specific DFT calculations; and (iii) the electrolyte properties (LRE) in the interface region (ion density and potential distribution, solvent density and alignment) using the mean-field double layer model. Closing the self-consistency loop, (iv) the LRE obtained as the output of the double-layer model is used as input for the microkinetic model, which in turn defines the boundary condition for the double-layer model. The resulting surface charge density calculated from the double layer model impacts the binding energies of reaction intermediates, defining another coupling effect that the approach solves self-consistently. Solving the coupled model at a series of electrode potentials, one can build a closed system of relations between the microscopic parameter space of catalyst composition and interfacial properties and the macroscopic parameter space of the effective electrocatalytic activity for the reaction of interest.

The capabilities of the concerted approach that self-consistently integrates DFT-based first-principles calculations for parameterisation of microscopic mechanistic parameters, a mean-field type model of the double layer, and a microkinetic model for the multistep kinetics were demonstrated in ref. 48 for the oxygen reduction reaction (ORR) at Pt(111). In ref. 47 and 102, the approach was applied to the OER.

The approach rationalises contributions of terms consisting of different sequences of elementary steps to the net rate of the reaction. This analysis led to the identification of a rate-determining term (RDT) as a new mechanistic concept to assess and compare the activity of electrocatalyst materials.

The RDT concept incorporates detailed microscopic information about the kinetics and thermodynamics of multistep electrochemical reactions. It represents a generalisation over more widely-known albeit simplified reactivity concepts such as the rate-determining step (RDS),^216,217^ a well-established concept in chemical kinetics, or the potential-determining step (PDS),^82,158,218,219^ specifically developed for the field of electrocatalysis. Both the RDS and PDS concepts, which have been employed in the past to guide comparative materials assessment and screening, start from a premise that a single elementary step could be identified that determines the net rate of the overall reaction. This premise is, however, usually overly reductionist, and it fails to capture vital details of multistep reactions. Using RDS and PDS concepts could, therefore, mislead searches for the most active electrocatalyst material for a particular reaction, as demonstrated in the volcano plots in Fig. 4 and 6 of ref. 47.

The detailed deconvolution of contributions of microscopic elementary steps and reactions pathways to the overall rate of the multistep reaction allows effective kinetic electrode parameters, such as the Tafel slope and the exchange current density, to be calculated as functions of electrode potential. Predictions for potential dependences of the Tafel slope were found to be in agreement with experimental data for the ORR^48^ and the OER.^47^ Exchange current densities calculated from the fully parameterised model in ref. 48 exhibit a variation by more than ten orders of magnitude over the potential range relevant for the ORR.

Lastly, knowledge of the LRE obtained as the self-consistent loop that solves the theoretical-computational framework model should be the basis for comparative assessment or screening of electrocatalysts in terms of their activity for the reaction of interest, *e.g.*, OER or HER. This means that a single descriptor based on the chemisorption energy of a reaction intermediate or the d-band center of the metal, as employed in computational approaches based on the CHE, is not sufficient for catalyst screening. Clearly, the LRE that is related to the charging or capacitive response of the interface must be accounted for. Moreover, it should be noted that knowing the LRE is also an essential prerequisite for assessing catalyst stability, *i.e.*, predicting rates of catalyst degradation.

## 3 Overview of electrolyser technologies

Water electrolysis – literally the decomposition of water under the action of electricity – was first performed by using static electricity by Deiman and van Troostwijk 1789^220^ and then in a “more actual manner” by Nicholson and Carlisle, using a Volta pile, in the early 19th century.^221^ Since then, many electrolysis processes have been discovered, optimised and industrially implemented; for example, the Hall-Héroult process to produce aluminium in molten-salt-based cells^222,223^ or the Castner–Kellner process of alkaline salt electrolysis to produce alkali-hydroxides (*e.g.*, NaOH and KOH).^224^ In this section, the principal water electrolysis technologies are reviewed, covering their main advantages and drawbacks. Special emphasis is given to their critical core materials, which would benefit from further research to make these technologies an industrial reality, or to enhance their present performance (for already-industrialised systems).

**Scheme 3:**
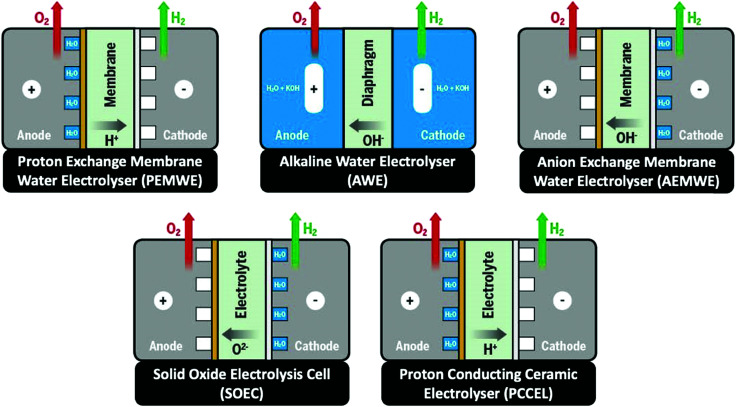
Schematic presentation of the five main types of water electrolysers.

Water electrolysis is the most significant primary electrochemical method for molecular hydrogen, and its importance will increase rapidly with renewable energy production. Depending on the electrolytes, separators, working temperatures and pressures employed, five main types of water electrolysers (summarised in Scheme 3 and Table 1) are encountered, namely:

**Table 1:** Short description of the five types of water electrolysers. Modified from IRENA^225^

	AWE	PEMWE	AEMWE	SOEC	PCCEL
Operating temperature	70–90 °C	50–80 °C	40–60 °C	700–850 °C	300–600 °C
Operating pressure	1–30 bar	<70 bar	<35 bar	1 bar	1 bar
Electrolyte	Potassium hydroxide (KOH) 5–7 mol L^−1^	PFSA membranes	DVB polymer support with KOH or NaHCO_3_ 1 mol L^−1^	Yttria-stabilised zirconia (YSZ)	(Y,Yb)-Doped-Ba(Ce,Zr)O_3−*δ*_
Separator	ZrO_2_ stabilised with PPS mesh	Solid electrolyte (above)	Solid electrolyte (above)	Solid electrolyte (above)	Solid electrolyte (above)
Electrode/catalyst (oxygen side)	Nickel coated perforated stainless steel	Iridium oxide	High surface area nickel or NiFeCo alloys	Perovskite-type (*e.g.*, LSCF, LSM)	Perovskite-type (*e.g.*, LSCF, LSM
Electrode/catalyst (hydrogen side)	Nickel coated perforated stainless steel	Platinum nanoparticles on carbon black	High surface area nickel	Ni/YSZ	Ni/YSZ, Ni-BZY/LSC, BCFYZ
Porous transport layer anode	Nickel mesh (not always present)	Platinum coated sintered porous titanium	Nickel foam	Coarse nickel-mesh or foam	Coarse nickel-mesh or foam
Porous transport layer cathode	Nickel mesh	Sintered porous titanium or carbon cloth	Nickel foam or carbon cloth	None	None
Bipolar plate anode	Nickel-coated stainless steel	Platinum-coated titanium	Nickel-coated stainless steel	None	None
Bipolar plate cathode	Nickel-coated stainless steel	Gold-coated titanium	Nickel-coated stainless steel	Cobalt-coated stainless steel	Cobalt-coated stainless steel
Frames and sealing	PSU, PTFE, EPDM	PTFE, PSU, ETFE	PTFE, silicon	Ceramic glass	Ceramic glass

(1) Alkaline water electrolyser (AWE)

(2) Proton exchange membrane water electrolyser (PEMWE)

(3) Anion exchange membrane water electrolyser (AEMWE)

(4) Solid oxide electrolysis cell (SOEC)

(5) Proton conducting ceramic electrolyser (PCCEL)

### 3.1 Near ambient temperature electrolysers

#### 3.1.1 Alkaline-type electrolysers

##### 3.1.1.1 Alkaline water electrolyser (AWE)

Alkaline water electrolysis is the most mature hydrogen production technology *via* electrochemical water splitting. It is implemented for industrial hydrogen production since several decades^226^ (as a matter of fact, that there were over 400 industrial water electrolyzers in use already in 1902^227^), notably with hydrogen production units coupled to hydroelectric power (dam), *e.g.* in Trail (Canada), Nangaï (India), Aswan (Egypt – it is part of the Aswan Dam project), Norsk (Norway),^228^ and many other plants. In these facilities, the hydrogen produced has a renewable origin (hydroelectric power) and is, therefore, a ‘green’ endeavour for (alkaline) water electrolysis. Hydrogen production uses electricity produced in off-peak (low-demand) times, or at times of large river flows in the spring, enabling electricity storage in the form of chemical bonds (power-to-hydrogen). When the peak electricity generation is needed, hydrogen can be converted back to electricity *via* fuel cells,^229^ although hydrogen is typically used locally as a chemical, notably in plants producing fertilisers (Aswan, Nangaï, Trail), but also in metallurgy and for the production of heavy water. At present, another technology of alkaline electrolysis produces a wealth of pure hydrogen: the brine electrolysis process. In this case, the reaction at the positive electrode is not the evolution of oxygen, but the evolution of chlorine, the hydrogen rarely been utilised as a fuel for fuel cells, but instead as a chemical (*e.g.*, to produce hydrogen peroxide)^230^ or for heat generation. This technology will not be further addressed herein.

The basic architecture of an alkaline water electrolyser is as simple as one can expect for an electrochemical system: 2 electrodes separated by a porous separator impregnated with an alkali electrolyte, usually KOH). It is this inherent simplicity that has enabled the early and consequent deployment of industrial AWE cells worldwide. Alkaline water electrolyser cells consist of two metallic electrodes that are immersed in an aqueous liquid electrolyte (generally 25–40 wt% aqueous solutions of KOH or NaOH); the working temperature range is 70–90 °C in order to provide maximum electrical conductivity: KOH has a specific conductivity of 0.184 S cm^−1^ at 25 °C.^231^ The reduction of water in an AWE (at pH 14) takes place at the cathode (eqn (8)):8
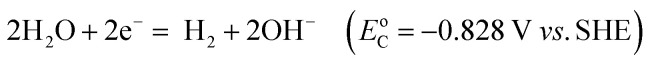
while the hydroxyl ion oxidation occurs at the anode (eqn (9)):9
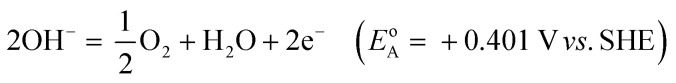
The AWE technology presents several advantages, mostly related to the alkali metal hydroxide aqueous electrolyte, which enables using non-PGM catalysts without compromising the performance and durability in operation.^232^ Electrode materials based on nickel (RANEY® at the negative electrode or oxyhydroxides at the positive electrode),^226,233–235^ cobalt or simply stainless steels^233,234^ are conventionally used in AWE cells.^226^ Some important and recent advances regarding the development of such PGM-free catalysts for AWE will be addressed in Section 7, while Section 6 will detail more classical (and sometimes used in AWE) PGM-based catalysts.

Several designs/constructions are used for industrial alkaline water electrolysers.^236^ Either the individual cells are connected in parallel (monopolar assembly), or in series (bipolar assembly). In the former case, all anodes (resp. cathodes) are connected in parallel, usually on copper (or aluminium) conduction bars to lower Ohmic drop and ensure homogeneous current feeding/collection. In the latter case, the current is collected *via* endplates at the two extremities of the assembly, the cathode and anode of neighbouring unit cells being electrically connected. The monopolar and bipolar assemblies have their own advantages and drawbacks (Table 2), the bipolar configuration being more efficient from an energetic viewpoint.

**Table 2:** Advantages and drawbacks of the monopolar and bipolar configurations of assembly for AWE cells

Configuration	Monopolar assembly	Bipolar assembly
Advantages	• Smaller electrolysis voltage due to the parallel stacking, resulting in larger electrical safety of operation	• More homogeneous current feeding
	• Absence of current leaks	• Gain in voltage due to minored Ohmic drop in connectors/wires
	• Impossibility of electrical shorts between anodes and cathodes	• Smaller current intensity, resulting in less expensive electrical transformer/rectifier
Drawbacks	• Less homogeneous current feeding	• Larger installation voltage, inducing electrical safety issues
	• Larger number of electrical contacts/wires	• Possible current leaks between the inlets/outlets of electrolyte, feeding the cells in parallel (high potential differences applied to the same channel)
	• Larger current intensity, resulting in more expensive electrical transformer/rectifier	• Risk of contact failure between two neighbouring anodes/cathodes

Whatever the configuration, the main drawback of AWE cells is linked to the generation of H_2_ and O_2_ bubbles at the cathode and anode, respectively^17^ Firstly, bubbles in the liquid electrolyte alter its ionic conductivity, hence heightening the cell Ohmic-drop and the operating cost of AWE. Secondly, because the separator is porous, intermixing between H_2_ and O_2_ bubbles is possible if the mass-transport is not well-balanced, which has adverse consequences in terms of safety of operation, but also of gas purity.^237,238^ In practice, AWE cells need several hours to reach their steady-state, in terms of electrolyte flow, temperature and current density (hence of bubbles generated),^57,239^ which means that AWE can usually not be operated in transient regime, making their coupling to renewable sources of solar/wind electricity awkward (although this coupling is being studied).^240^ For the same reasons, operation under pressure is awkward. These drawbacks are not encountered with water electrolysis cells using a dense separator, like a PEM or an AEMs.

##### 3.1.1.2 Alkaline membrane-based water electrolysis

To further decrease the internal resistance of the electrolysers and to operate the cells at high pressure, the possibility of using a non-porous membrane with high anionic conductivity has also been studied. Porous catalyst layers are deposited on each side of the polymeric membrane to form a membrane electrode assembly (MEA) very similar to what is currently used in PEMWE. The main requirements of OH^−^-conducting membranes are as follows:

(1) excellent mechanical and thermal stability in contact with water and during operations;

(2) insulator regarding electronic conductivity;

(3) efficient transfer of OH^−^ ions from one electrode to the other (high ionic conductivity);

(4) very low permeability to gases to minimise or even eliminate gas crossover between the anodic and cathodic compartments;

(5) low cost.

AEM are described in detail in Section 5.2. In AEMWE, the alkaline environment allows a great variety of catalyst material selection, which could permit the use of non-precious metals for the HER and OER. The ability to use cheaper non-platinum or non-precious metal-based catalysts in AEMWEs is the reason why research is actively addressing the issues hindering AEM commercialising for AEMWE.

#### 3.1.2 Proton exchange membrane water electrolyser (PEMWE)

PEMWEs are the most effective water electrolysis technology. Their critical component is the ion-exchange membrane. Anode and cathode form a sandwich against a proton-conducting polymer electrolyte (*e.g.*, Nafion®), the so-called membrane-electrode assembly (MEA). This MEA is then immersed in pure water, and a cell voltage (*V*_cell_) is applied to trigger the O_2_ evolution at the anode (eqn (10)):10
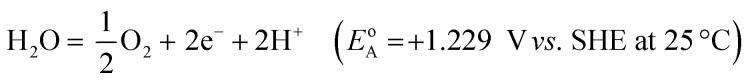
and the H_2_ evolution at the cathode (eqn (11)):11
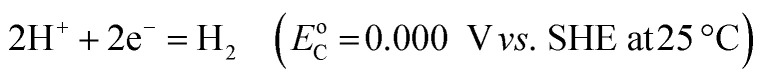
The overall reaction in a PEMWE (as in all WE cells) being (eqn (12)):12
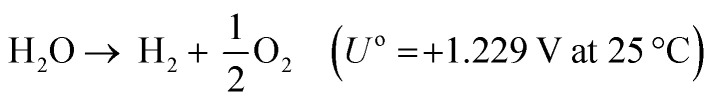


Importantly, there is no net consumption of the electrolyte and only water is consumed. Provided that water is supplied at the rate at which it is consumed, the concentration of the ions remains constant. During the electrolysis, mobile proton species remain confined with the highly-acidic polymer membrane. Due to this, noble metal catalysts that are resistant to such acidity are required at both the cathode and the anode.

Modern PEMWEs contain perfluorinated sulphonic acid copolymer membranes because of their relatively high ionic conductivity (as compared to other membrane materials), high mechanical strength, and fairly strong chemical stability. The most widely used membrane material is Nafion® by DuPont de Nemours Co. (USA). Nafion® membranes are thin, elastic and transparent. However, swelling and dissociation of the ion-exchange groups of the membrane can occur when in contact with water, resulting in the free movement of protons from one electrode to another. The resistivity of perfluorinated sulphonic acid membranes is significantly larger than that of alkali solutions (*i.e.*, 11–12 Ω cm at 20 °C and 5–6 Ω cm at 80–90 °C). Thin membranes having a thickness in the 100–300 μm range are used to reduce ohmic losses. However, using thin membranes increase the permeability of gases through the membrane, reducing the efficiency of the system. Since liquid electrolytes are not used in PEMWEs, the electrodes are pressed tightly against the membrane in a zero-gap configuration. The catalysts used in PEMWEs are deposited on the surface of the ion-conducting membrane (to form a CCM – catalyst coated membrane) to achieve high surface contact between the catalyst and the electrolyte. Porous current collectors are then pressed against these CCMs, adjacent electrolysis cells being stacked together and separated by metallic bipolar plates. The intimate contact between the porous transport layer (PTL) is critical to reach high performance PEMWE. The HER electrode morphology can essentially be kept similar to that of PEMFC anodes (where H_2_ oxidation occurs). Pt-based catalysts supported on high surface area carbon in contact with a conventional gas diffusion layer (GDL) encompassing a microporous layer (mixture of high surface area carbon and PTFE binder) is appropriate,^241,242^ and further refinements are possible (fluorinated carbons improve the performance^243^). On the OER side, the issue is more complex, because carbon is not stable; hence, titanium-based PTL are usually employed as the porous current collector. Because they are complex to nanostructure, Ti-based PTL usually display coarser structures than carbon-based ones, enabling poorer distribution of the electrical contact points to the OER catalyst layer. As a result, the OER preferentially occurs at the regions of the catalyst layer near to good conducting paths (contact points) of the PTL, resulting in very heterogeneous OER within the catalyst layer^119,244^ especially with alteration of the PTL/catalyst layer conductivity, owing to unavoidable increase of the interfacial contact resistance of the Ti PTL upon gradual passivation in OER regime.^245^ PTL with finer structures improve the situation, resulting in more numerous electrical contact points between the PTL and the catalyst layer^246^ opening the way to more tailored designed GDSs for the OER in PEMWE.^247,248^ The catalysts used in PEMWEs are generally platinum group metals (PGMs). Ruthenium (Ru) is one such PGM that has high catalytic activity in the O_2_ evolution reaction when in oxide form. However, it must be noted that Ru-based electrodes can have poor stability in acidic conditions. The most commonly used anode catalyst is iridium (Ir) with loadings of around 1.0–2.0 mg cm^−2^, whereas platinum (Pt) or palladium (Pd) are the main catalysts used at the cathode, with the anode current collectors being constructed of a porous titanium (Ti) material and the cathode current collectors being constructed of carbon material.

When compared to other water electrolysers, the main advantages of a PEMWEs are as follows (see also Table 1):

(1) Possibility of operating at high current densities (high power);

(2) High energy efficiency;

(3) High purity of generated gases; and

(4) A high dynamic range (ideal for use with intermittent renewable energy).

The main drawbacks are:

(1) High initial capital investment; and

(2) Requirement for high-temperature electrolysers

### 3.2 High-temperature electrolysers

#### 3.2.1 Solid oxide electrolyser cell (SOEC)

In solid oxide electrolyser cells (SOEC),^249–252^ oxide-ion conducting ceramics are used both as the solid electrolyte and the cell separator. The operating temperatures for the SOECs are usually in the 800–1000 °C range. The electrolyte used in SOECs is generally zirconia that has been stabilised with yttrium and scandium oxides (“YSZ”), with the main components consisting of stainless-steel bipolar plates and manganite-coated stabilised zirconia as the solid electrolyte. In a SOEC, water vapour is reduced at the cathode (eqn (13)):13
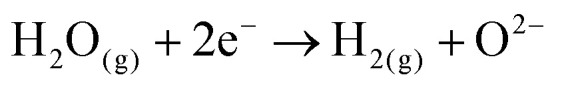
The resulting oxygen ions migrate to the anode, where O_2_ evolves (eqn (14)):14
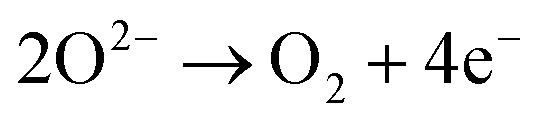
The oxide ions are transported from the cathode to the anode across the zirconia electrolyte by an ionic diffusion process, very thin (*ca.* 30–150 μm thick) ceramic membranes being used to reduce the ohmic losses. The steam cathode is typically composed porous nickel, while the air anode is typically composed of porous perovskite materials, such as lanthanum strontium manganite (“LSM”), with various catalyst blends under development, such as lanthanum strontium cobalt ferrite (LSCF) and samarium-doped ceria^253^ and rare-earth nickelates.^254^ Detailed modelling of heterogeneous electrocatalysis in these systems, supported by impedance and imaging data, has shown that oxygen surface diffusion and de-sorption on the LSM surface from the YSZ triple-phase boundary can be the rate-limiting step, which can be optimised by tailoring the microstructure of the porous composite functional layer at the cathode-electrolyte interface.^255^ Despite the high temperature, multi-component gas diffusion in the porous electrodes can also be rate-limiting, especially at the steam electrode.^256^

SOEC technologies have been driven by the possibility to operate at high current densities (*e.g.*, 3.6 A cm^−2^ at 1.48 V and 950 °C) and efficiencies. In addition, the electrochemical processes are highly efficient and reversible, because SOECs are run at high operating temperatures, which allows a single SOEC unit to operate as either a fuel cell or electrolysis cell. Challenges for current research include understanding and controlling electrochemical degradation and thermo-mechanical stability,^257^ in order to meet the demands of producing H2 from (intermittent) renewable electricity.

#### 3.2.2 Proton conducting ceramic electrolyser (PCCEL)

In a proton-conducting ceramic electrolyser (PCCEL) water vapour is supplied to the oxygen electrode side (anode), and pure H_2_ is generated at the cathode (no dilution by water vapour). The cathode in both a SOEC and a PCCEL is typically a Ni-in-oxide-electrolyte composite (cermet). Since the water vapour is supplied to the anode, it is expected to avoid Ni oxidation and irreversible agglomeration at the cathode. In addition, the intermediate operating temperatures of a PCCEL (around 500 °C) brings economic advantages: (i) the required electrical energy for water electrolysis decreases as the operating temperature increases, since a significant portion of the energy supplied is in the form of thermal energy; (ii) the sluggish kinetic issues at low temperatures are offset by the elevated operating temperatures. Therefore, PCCELs enable water electrolysis with higher efficiency than low-temperature electrolysers. Although the first demonstration of PCCEL technology was reported in 1981, its development has been slow due to the technical difficulty associated with the fabrication of the bilayer structure in the configuration of thin and dense electrolyte/porous electrode support. They also suffer from the same poor thermos-mechanical properties as SOEC.

## 4. Key performance indicators (KPI) and technology targets

According to the International Energy Agency's Net Zero by 2050 report,^258^ achieving global net-zero emissions by 2050 would require to produce around 306 million tonnes of green hydrogen from renewable energy sources each year. This would also require a global electrolyser capacity of *ca.* 3600 GW, up from about 300 MW today, and *ca.* 14 500 TW h of electricity—about 20% of the world's electricity supply (∼71 000 TW h). The IEA predicts that blue hydrogen from natural gas will cost around US$1–2 per kg, with green hydrogen at U$1–2.50 kg^−1^ by 2050. In the same report, the IEA estimates a substantial increase in renewable and other energy sources of installed capacities (Table 3).

**Table 3:** Power capacity to be installed in 2050 for reaching net-zero emissions. Modified from the IEA^258^

Energy sources	2020 installed capacity/GW	2050 projected installed capacity/GW
Solar PV	737	14 458
Wind	737	8265
Hydro	1327	2599
Hydrogen power plants	0	1867
Nuclear	415	812
Bioenergy	171	640
Coal-fire with carbon capture sequestration (CCS)	1	222
Gas-fire with carbon capture sequestration (CCS)	0	171
Concentrating solar power (CSP)	6	426
Geothermal	15	126
Marine (wave and tidal)	1	55
Total	3410	29 641

On 8th July 2020, the European Union (EU) launched “A hydrogen strategy for a climate-neutral Europe”^259^ as part of its Green Deal but also announced two other important initiatives – the Energy System Integration plan and the European Clean Hydrogen Alliance (E2CH2A). The overall objective is to establish a European hydrogen economy and to make of euro the currency of choice on the global market. The industry led E2CH2A intends to promote investments in hydrogen production and application.^259^

All three initiatives offer a unique opportunity for using wind- and PV-sourced renewable hydrogen (RH_2_) to supply fuel and chemical feedstock throughout Europe and to store energy in salt caverns. Clean hydrogen production capacity is projected to grow to 1 million tonnes by 2024 and 10 million by 2030 – meaning 6 and 40 GW by 2024 and 2030, respectively. Adding in 40 GW produced in neighbouring countries, 2030 capacity will reduce CO_2_ emissions by 100 million tonnes.

The total cost of kick-starting a European hydrogen economy is estimated at €430 billion, with initial EU investments coming to €96 billion. The funding will be split between electrolysers (13%), offshore (47%) and onshore wind (25%) and solar PV (15%). The aim is to produce 4.4 million tonnes of RH_2_ in the EU. An additional €91.5 billion will be spent producing 4 million tonnes in Ukraine and North Africa.

The European electrolyser industry will create an estimated 170 000 jobs. As for the hydrogen infrastructure, €120 billion will need to be invested in the EU and North Africa to supply RH_2_ for fuel and materials production, *e.g.*, for producing kerosene and steel, and to fund hydrogen manufacture in the transportation, heat and power markets. Natural gas pipelines and storage systems are expected to serve an important electricity interconnector function across Europe, while hydrogen could provide more grid flexibility.

The European Commission's strategy for rapid market growth involves three stages:^259^

– Stage 1 (2020 to 2024): produce 1 million tonnes of RH_2_ and kick-start electricity generation.

– Stage 2 (2025 to 2030): increase energy production capacity, produce 10 million tonnes, and decarbonise most of Europe's energy markets and industry.

– Stage 3 (2030 to 2050): modernise and transform hard-to-abate sectors, *e.g.*, shipping and aviation.

Overall, an EU-wide market for hydrogen promises significant value-adds within a multi-billion-euro high-tech environment. Hydrogen production, storage and distribution will drive innovation, growth, jobs, trade and transportation throughout the EU. The technology's competitiveness will hinge on the swift delivery of new, innovative and sustainable solutions promising efficient, on-demand power. These solutions will be vital to meet societal demand for reliable, clean and efficient energy generation through smart, green and integrated networks. By 2050, a continent-wide hydrogen market could generate €820 billion in revenues, provide 5.4 million jobs, and avoid 560 million tonnes of CO_2_ a year. Supporting innovative production techniques is thus crucial to facilitate the establishment of a hydrogen economy.

In order to achieve these ambitious economic and production targets, stringent technology targets and key performance indicators (KPIs) have been implemented. Table 4 lists KPIs for the four-electrolysis technologies considered, both for the state-of-the-art in 2020 and as targets for 2050.

**Table 4:** State-of-the-art and future key performance indicators (KPIs) for all electrolyser technologies. Adapted from IRENA^225^

	2022	Target 2050	R&D focus
	PEM electrolysers		
Nominal current density	1–3 A cm^−2^	4–6 A cm^−2^	Design, membrane
Voltage range (limits)	1.4–2.3 V	<1.7 V	Catalyst, membrane
Operating temperature	50–80 °C	80 °C	Effect on durability
Cell pressure	≤50 bar	>70 bar	Membrane, rec. catalysts
Load range	5–130%	5–300%	Membrane
H_2_ purity	99.9–99.9999%	Same	Membrane
Voltage efficiency (LHV)	50–68%	>80%	Catalysts
Electrical efficiency (stack)	47–66 kW h kgH_2_^−1^	<42 kW h kgH_2_^−1^	Catalysts/membrane
Electrical efficiency (system)	50–83 kW h kgH_2_^−1^	<45 kW h kgH_2_^−1^	Balance of plant
Lifetime (stack)	50 000–80 000 h	100 000–120 000 h	Membrane, catalysts, PTLs
Stack unit size	1–2 MW	10 MW	MEA, PTL
Electrode area	≤3000 cm^2^	>10 000 cm^2^	MEA, PTL
Cold start (to nom. load)	<20 min	<5 min	Insulation (design)
Capital costs (stack) min 1 MW	400 USD kW^−1^	<100 USD kW^−1^	MEA, PTLs, BPs
Capital costs (system) min 10 MW	700–1400 USD kW^−1^	<200 USD kW^−1^	Rectifier, water purification
	Alkaline electrolysers		
Nominal current density	0.2–0.8 A cm^−2^	>2 A cm^−2^	Diaphragm
Voltage range (limits)	1.4–3 V	<1.7 V	Catalysts
Operating temperature	70–90 °C	>90 °C	Diaphragm, frames, BoP components
Cell pressure	<30 bar	>70 bar	Diaphragm, cell, frames
Load range	15–100%	5–300%	Diaphragm
H_2_ purity	99.9–99.9998%	>99.9999%	Diaphragm
Voltage efficiency (LHV)	50–68%	>70%	Catalysts, temp.
Electrical efficiency (stack)	47–66 kW h kgH_2_^−1^	<42 kW h kgH_2_^−1^	Diaphragm, catalysts
Electrical efficiency (system)	50–78 kW h kgH_2_^−1^	<45 kW h kgH_2_^−1^	Balance of plant
Lifetime (stack)	60 000 h	100 000 h	Electrodes
Stack unit size	1 MW	10 MW	Electrodes
Electrode area	10 000–30 000 cm^2^	30 000 cm^2^	Electrodes
Cold start (to nom. Load)	<50 min	<30 min	Insulation (design)
Capital costs (stack) min 1 MW	270 USD kW^−1^	<100 USD kW^−1^	Electrodes
Capital costs (system) min 10 MW	500–1000 USD kW^−1^	<200 USD kW^−1^	Balance of plant
	AEM electrolysers		
Nominal current density	0.2–2 A cm^−2^	>2 A cm^−2^	Membrane, rec., catalyst
Voltage range (limits)	1.4–2.0 V	<2 V	Catalyst
Operating temperature	40–60 °C	80 °C	Effect on durability
Cell pressure	<35 bar	>70 bar	Membrane
Load range	5–100%	5–200%	Membrane
H_2_ purity	99.9–99.999%	>99.9999%	Membrane
Voltage efficiency (LHV)	52–67%	>75%	Catalysts
Electrical efficiency (stack)	51.5–66 kW h kgH_2_^−1^	<42 kW h kgH_2_^−1^	Catalysts/membrane
Electrical efficiency (system)	57–69 kW h kgH_2_^−1^	<45 kW h kgH_2_^−1^	Balance of plant
Lifetime (stack)	>5000 h	100 000 h	Membrane, electrodes
Stack unit size	2.5 kW	2 MW	MEA
Electrode area	<300 cm^2^	1000 cm^2^	MEA
Cold start (to nom. Load)	<20 min	<5 min	Insulation (design)
Capital costs (stack) min 1 MW	Unknown	<100 USD kW^−1^	MEA
Capital costs (system) min 1 MW	Unknown	<200 USD kW^−1^	Rectifier
	Solid oxide electrolysers		
Nominal current density	0.3–1 A cm^−2^	>2 A cm^−2^	Electrolyte, electrodes
Voltage range (limits)	1.0–1.5 V	<1.48 V	Catalysts
Operating temperature	700–850 °C	<600 °C	Electrolyte
Cell pressure	1 bar	>20 bar	Electrolyte, electrodes
Load range	30–125%	0–200%	Electrolyte, electrodes
H_2_ purity	99.9%	>99.9999%	Electrolyte, electrodes
Voltage efficiency (LHV)	75–85%	>85%	Catalysts
Electrical efficiency (stack)	35–50 kW h kgH_2_^−1^	<35 kW h kgH_2_^−1^	Electrolyte, electrodes
Electrical efficiency (system)	40–50 kW h kgH_2_^−1^	<40 kW h kgH_2_^−1^	Balance of plant
Lifetime (stack)	<20 000 h	80 000 h	All
Stack unit size	5 kW	200 kW	All
Electrode area	200 cm^2^	500 cm^2^	All
Cold start (to nom. Load)	>600 min	<300 min	Insulation (design)
Capital costs (stack) min 1 MW	>2000 USD kW^−1^	<200 USD kW^−1^	Electrolyte, electrodes
Capital costs (system) min 1 MW	Unknown	<300 USD kW^−1^	All

## 5 Materials: focus, challenges, and solutions

It is clear that green hydrogen production (coupling renewable energy systems with electrolysers) is witnessing an exponential increase. According to the Hydrogen Council, hydrogen could help meet almost a quarter of the global energy demand by 2050, creating a US$10 trillion addressable market. These projections are supported by the recent strong hydrogen-focused national hydrogen strategies, for example in Germany, France, Spain, Portugal, the EU, Japan, South Korea, Australia, New Zealand, Canada, Chile and the USA. Moreover, Aurora Energy Research predicts that a 1000-fold increase in electrolyser units is expected by 2040.^260,261^

Overall, most green hydrogen projects involve the installation of PEMWEs and AWEs as they are well-established technologies, although AEMWE (*e.g.*, Enapter) and SOEC (*e.g.*, Haldor Topsoe) technologies are currently being chosen as potential candidates for large-scale hydrogen production. Water electrolysis is the most significant primary electrochemical method for hydrogen production, and its importance will increase rapidly with renewable energy production.

However, water electrolysis technologies strongly depend upon the materials used *i.e.*, catalysts, electrolytes, separators, working temperatures and pressures. Currently, hydrogen production *via* electrolysis is more expensive than *via* other methods due to the capital costs and dependence on electricity costs. Although the CAPEX and OPEX of electrolysers have been reduced noticeably since 2012, further improvements are required, especially when operated solely on renewable energy sources; limited utilisation increases the impact of the CAPEX and OPEX on commercial viability. A second objective is to improve the electrolyser system's efficiency to reduce cost and the electricity consumed.

As a “rule of thumb” for all electrolysers, new materials which are low-cost, highly performing, and durable with a particular focus on thinner membranes (electrolytes), more active and durable catalysts and less critical raw materials, are required.

### 5.1 Alkaline water electrolyser

AWE is a mature and commercial technology which uses mainly nickel based material although some systems contain platinum (and cobalt). IRENA (the International Renewable Energy Agency) highlights that further R&D in AWE materials is required to drastically improve performance and durability.^225^Table 5 highlights the degree of challenges in material properties development and makes clear that new development in OER and HER catalyst is required.

**Table 5:** Degree of challenges in AWE material properties development. Modified from ref. 225. Abbreviations: E: easy; M: moderate; D: difficult L: low; M: moderate; H: high

AWE component: AWE material properties	Degree of challenges	Degree of improvement
Catalyst: high catalyst surface area > 50 m^2^ g^−1^	E	M
Catalyst: high catalyst utilisation > 80%	M	M
Catalyst: improved kinetics for both OER and HER with novel nickel-based alloys	M	H
Catalyst: mitigate catalyst poisoning/deactivation by foreign elements from electrolyte, and components present in the system	M	M
Catalyst: design, create, and integrate forms of recombination catalysts for gas permeation (crossover)	M	M
Catalyst: mitigate critical degradation of catalysts on the anode side to avoid loss of surface area	D	H
Catalyst: mitigate nickel hydride (NiH) formation on the cathode side	D	L
Catalyst layer: eliminate mechanical degradation of catalyst layers (delamination, dissolution)	D	H
Catalyst layer/porous transport layer: identify and reduce interface resistances from catalyst layer to PTLs	D	H
Diaphragm: identify stable polymer chemistry that can be used as ionomer (OH^−^ transport) to be used to fabricate electrodes for alkaline electrolysers	D	H

### 5.2 Anion exchange membrane water electrolysers

#### 5.2.1 AEM main properties

AEMWE can use the same catalysts than their liquid electrolyte counterpart. Those will be described in great details in the forthcoming sections. The real challenge of AEMWE is their AEM, as described below.

**Fig. 5:**
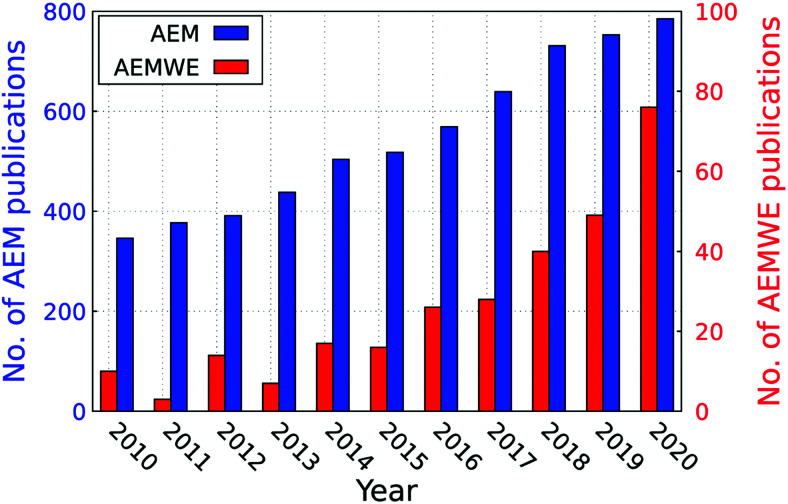
The annual number of publications in the field of AEMs (from Web of Science (access 29.07.2021)). Search terms: “Anion exchange membrane”, “water” and “electrolysis”.

The concept of AEM water electrolysis has been the subject of numerous reports in recent years in the scientific literature. A search of the academic literature (Web of Science) in the field in the past decade shows a remarkable increasing number of publications in AEMs as well as AEMs for water electrolysers (Fig. 5) clearly underpinning a growing interest in the research community, caused by the many advantages of the AEMWE over the PEMWE technology.

**Fig. 6:**
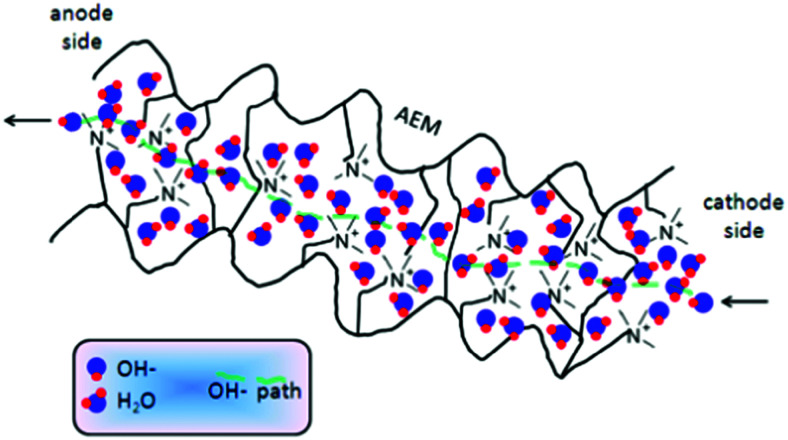
Scheme of hydroxide ion transport through an AEM. Reproduced with permission from ref. 314 Copyright Springer 2014.

AEM electrolysers work with an alkaline environment at the membrane interface provided by an anion-conducting polymeric membrane, called Hydroxide-Exchange Membrane (HEM), or generically, Anion-Exchange Membrane (AEM). Generally, AEMs are formed by a polymer backbone with anchored cationic groups that confer anion conductivity and selectivity (Fig. 6). The most common relevant backbones cited in the literature used for AEMs are: polysulphone type^262–266^ poly(ether ketone) type,^267–270^ poly(ether imide) type,^271–273^ poly(ether oxadiazole) type,^275–277^ and poly(phenylene oxide) type,^274,278–282^ polyphenylene type,^283–285^ fluorinated type,^286–291^ polybenzimidazole type,^292–298^ polyethylene type,^299–306^ and polystyrene type.^264,307–313^

**Fig. 7:**
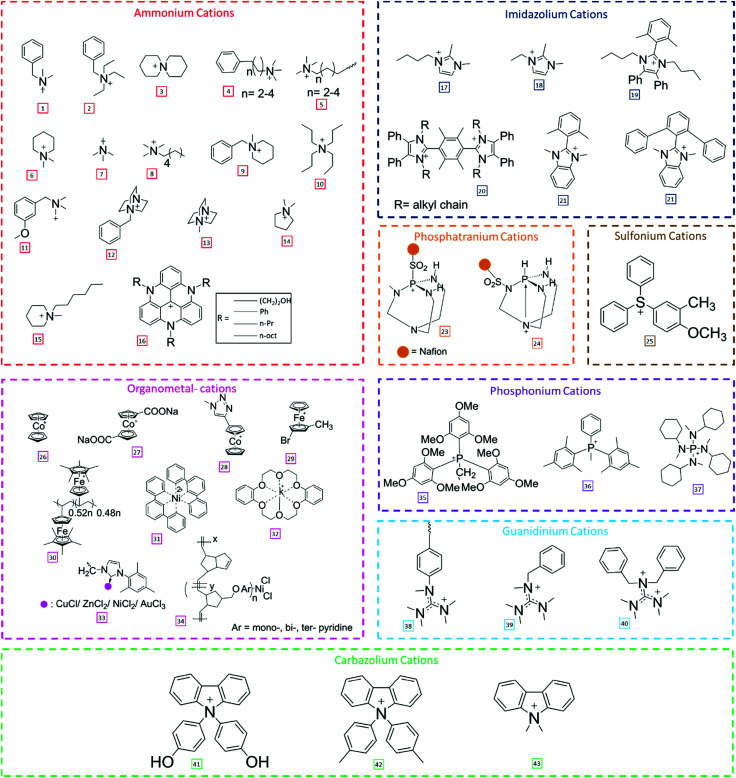
Scheme of representative cationic functional groups used in AEMs.

A few cationic functional group chemistries have been studied (Fig. 7), most of which involve N-based groups^296,315–322^ whereby piperidinium^323^ and spirocyclic^280^ are currently state-of-the-art. Besides non-N-based cationic groups like phosphonium,^269,324–327^ phosphatranium,^328^ S-based functional groups such as sulphonium^329–331^ and metal-containing anion-conducting groups, such as complexes of ruthenium(ii),^332,333^ Cobaltocenium,^334–338^ ferrocenium,^339^ copper(ii),^340^ Nickel(ii)^341,342^ and gold(ii)^343^ have been described (Fig. 7). Alternative anion-conducting groups were also exploited, such as guanidinium.^265,328,344–347^

**Fig. 8:**
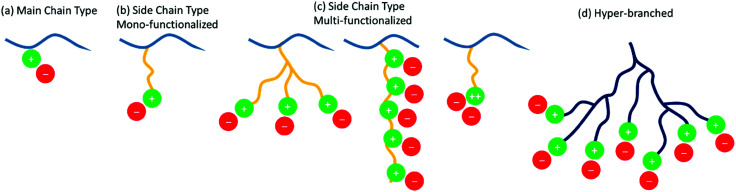
Schematic representation for (a) main chain type, (b) comb-shaped, (c) side chain type with multi-cationic head groups, and (d) hyper-branched AEMs.

These cationic functional groups can be an integral part of the backbone (*e.g.*, polybenzimidazolium-based polymers) or attached to the polymer backbone in different ways (Fig. 8).^348^ The cationic moieties can also consist of mono-cations or multi-cations.^341,349–351^ Besides hyper-branched cations (and pendant groups),^313,352,353^ can also be found.

**Fig. 9:**
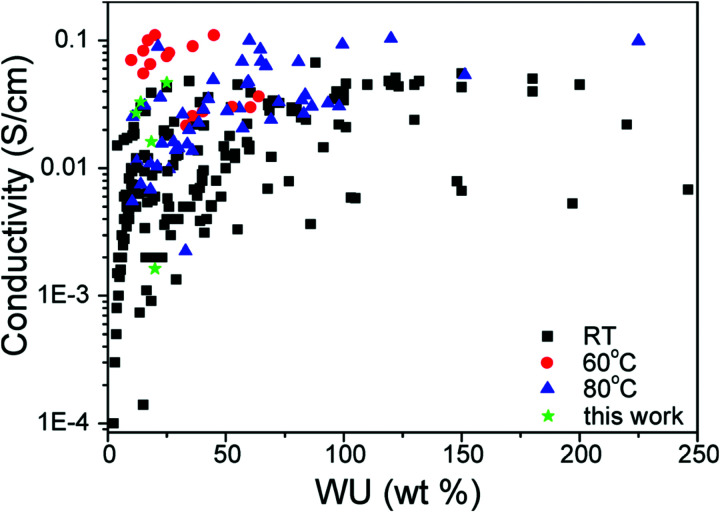
Conductivity as a function of water uptake (WU) from liquid water of AEMs at room temperature (RT, 20–30 °C), 60 °C, and 80 °C. Reproduced with permission from ref. 297. Copyright American Chemical Society 2018.

There are two main synthetic approaches to incorporate the cation functional groups into AEMs for AEMWE (and other electrochemical applications) – the direct polymerisation of cationic monomers and the post-polymerisation functionalisation of the cationic functional groups onto pre-formed polymer backbones.^354,355^ The most important performance characteristics of AEMs for water electrolysis applications are hydroxide conductivity (ideally, >100 mS cm^−1^) and water mobility, both of which are directly linked to each other. Zheng *et al.*^297^ have summarised conductivity and water uptake (WU) data that have been collected on AEMs submerged in liquid water (*e.g.*, not in contact with water vapor) (Fig. 9).

**Fig. 10:**
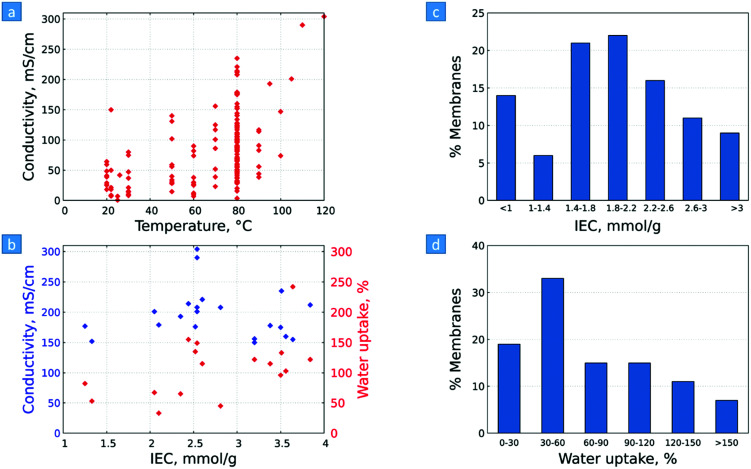
(a) AEM hydroxide conductivity *vs.* temperature, and (b) hydroxide conductivity and water uptake of selected AEMs (with conductivities ≥150 mS cm^−1^). (c) Fraction of AEMs at different levels of (c) IEC range and (d) water uptake range. Water uptake values are given in different temperatures. Represented data and the underlying sources are given in Table S1 (ESI†).

The conductivity, mechanical properties, and the physical dimensions of an AEM are functions of such water content, making this an important parameter for AEM design for water electrolysers.^356^Fig. 10a and b summarise hydroxide conductivity and water uptake of highly conducting AEMs reported in the past few years.

Alkaline AEMs (AAEMs) with hydroxide conductivity exceeding 200 mS cm^−1^ (*e.g.*,^357,358^) and as high as ∼300 mS cm^−1^ working durably at measured at temperatures close, or even above 100 °C were reported,^302,303^ values which only a few years ago seemed far from possible. These recent data show not only the potential of AEMs to be used in AEMWEs, but also suggest that they can be used in high-temperature AEM fuel cells (HT-AEMFC).^303,359,360^

High hydroxide conductivity is primarily enabled by a high density of cationic functional groups, *e.g.*, high ion exchange capacity (IEC). Fig. 10c and d show that most of the lately developed AEMs exhibit a mid-range of IEC of 1.4–2.2 mmol g^−1^, with relatively low water uptake (<60%) making them suitable for their use for AEMWE application.

**Fig. 11:**
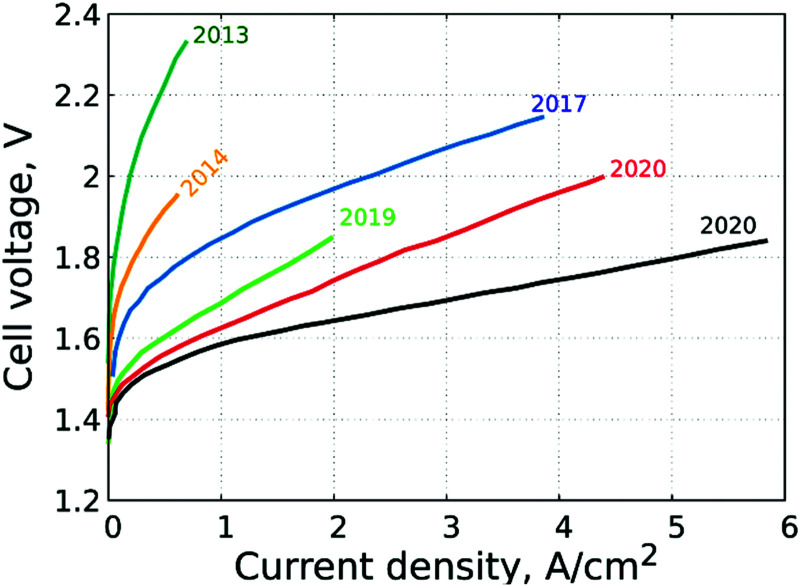
Selected high performance (polarisation curves) of AEMWEs reported in the literature. KOH solutions are fed to the AEMWEs. PGM-catalysts were used in these studies.^307,362–368^

Similar to what has been observed for the case of AEMFCs,^361^ the progress achieved in the AEMWE performance is also remarkable; thus the AEMWE cell performance (mostly achieved with PGM-free catalysts) increased from 0.4 (2012) to 5 A cm^−2^ at 1.8 V reached in 2020 (Fig. 11).

**Fig. 12:**
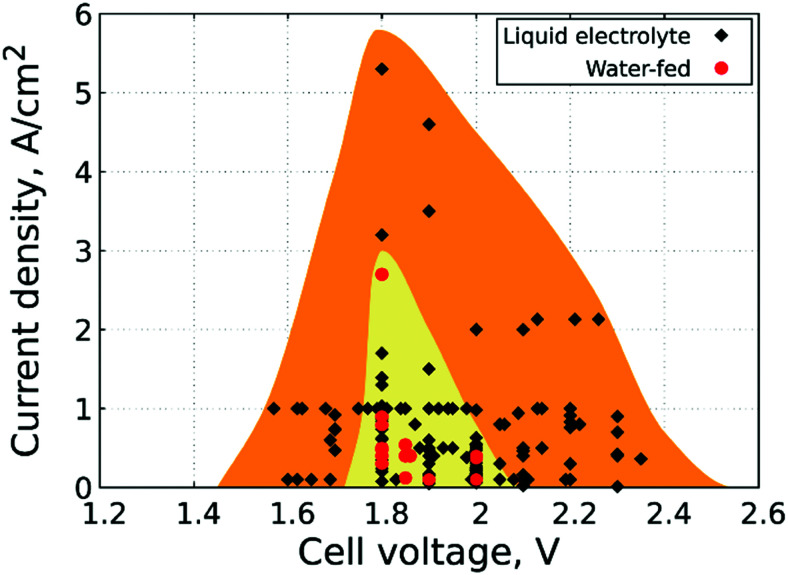
Performance summary of AEMWEs: comparison of current densities achieved at cell voltages in the 1.5–2.4 V range, extracted from different polarisation curves with different feed types. Yellow and orange areas represent AEMWE performance data with (KOH addition) and without liquid electrolyte (pure water). Operating temperature ranges from 22 to 90 °C. Main design parameters, operating conditions and underlying sources are provided in Tables S3 and S4 (ESI†).

Liquid electrolyte (in addition to polymer electrolytes) not only reduces the ohmic resistance of the AEM and the catalyst layer, but also improves the reaction kinetics, increasing in turn, the AEMWE performance^369^ (Fig. 12).

**Fig. 13:**
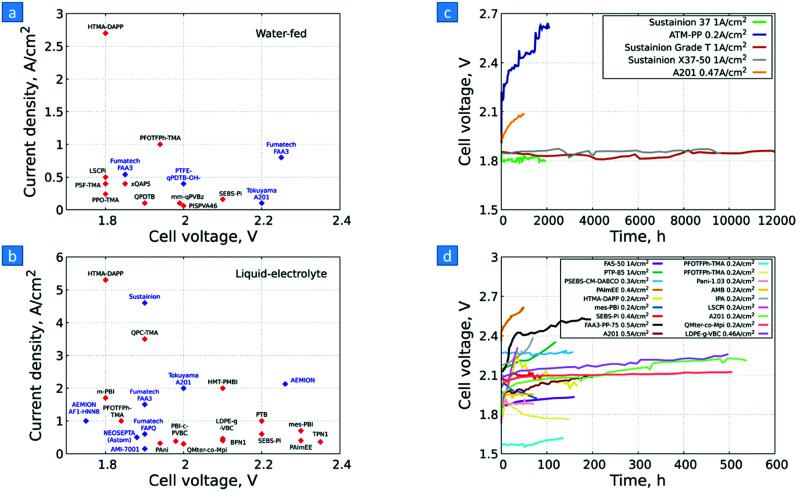
Selected AEMs and their operando performance stability data reported in the literature. AEMs under development (research in universities) are marked in red, and commercially available AEMs are marked in blue, for (a) pure water fed (no liquid electrolyte) and (b) liquid electrolyte. AEMs and their operando performance stability of selected AEMWE cells showing the long-term tests (c) and a zoom in into the 0–600 h range (d). Main design parameters, operating conditions and underlying sources are provided in Tables S3–S5 (ESI†).

Several commercially available AEMs have appeared in recent years.^370^Fig. 13 compares the performance and performance stability of AEMWEs based on AEMs from both research and industrial groups.

Very good performance has been reported with both research and industrial AEMs. Worth noting the outstanding performance of the HTMA-DAPP AEM^46^ and Sustainion® AEM.^371^

Despite the numerous reports presenting AEMWE performance data, studies on cell performance stability remain rare: most of the performance stability tests for AEMWE at constant current density showed a substantial reduction in performance in the first 200 h of operation (Fig. 13d), probably owing to chemical degradation of the anion conducting polymers used both as AEMs (and ionomers) at high pH value. Only a few AEMs relatively withstand performance above 1000 h such as Sustainion.^368,372^ Besides ionomer-catalyst detachment, ionomer poisoning, and catalyst degradation are further issues.^373^

#### 5.2.2 Remaining challenges of AEMs for their use in AEMWE applications

A peculiar characteristic of AEMWE, the high operating pressure, creates a unique operational challenge that requires special attention for the design of AEMs. Mechanical properties of the membrane and other components are almost the same for both PEM and AEM electrolysers, hence-no design modifications of cell components are required when hydrogen pressure at the cathode is limited to less than 10 bars.^374^ However, when hydrogen is pressurised in the cathode compartment, the increase in hydrogen cross-permeation through the membrane needs to be carefully considered. The hydrogen permeability of an AEM (hydrocarbon-based) is usually around one order of magnitude less than that of its counterpart PEM; so, the hydrogen barrier ability of an AEM of ∼28 μm thickness corresponds to that of a ∼175 μm-thick PEM,^375^ and substantially thinner membrane can be used in AEMWEs than in PEMWEs, one of the many advantages of AEMs for electrolysis.

**Fig. 14:**
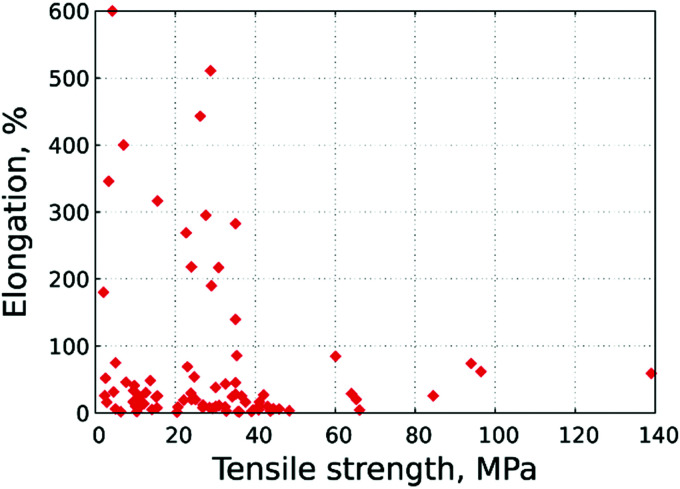
Elongation [%] *vs.* tensile strength [MPa]. In case there is a range of values, the lower value was considered. Represented data and underlying sources are given in Table S1 (ESI†).

Mechanical failure of the membrane can contribute to the failure of the entire device; thus, membrane durability is critical to overall system design. Fig. 14 gives an overview of the mechanical properties of selected AEMs. In general, for AEMs to be used in AEMWEs, high Young's modulus, a high tensile strength, and high elongation at break are desired. These properties are usually reported for AEM in their dry halide form at room temperature, which is, unfortunately, not relevant for AEMWE. Higher tensile strength of the catalyst ionomer improves the electrode-membrane adhesion and reduces electrode crack formation, which positively influences the device performance.^376^ For AEMs, benchmark values of >10 MPa stress at break, >100% elongation at break, and a Young's modulus between 75–400 MPa are proposed as being essential to obtain robust membranes.^377^

**Fig. 15:**
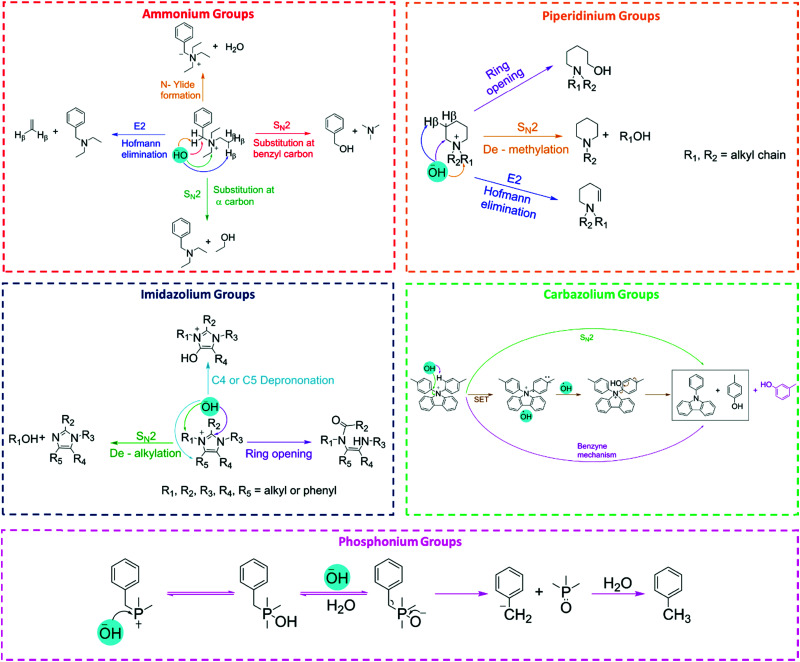
Different degradation mechanisms reported for cationic functional groups in AEMs.

Overcoming degradation caused by the alkaline electrolyte is still challenging. The molecular structure of the anion-conducting polymers (both for AEMs and ionomers in the electrodes) breaks down due to the strong reactivity of the hydroxide ions with the quaternary ammonium (QA) cation leading to a detrimental reduction of the membrane IEC, which, in turn, reduces the anion conductivity (increases cell resistance), causing a rapid decay in the AEMWE performance. Among the different mechanisms of degradation, Hofmann elimination (E2), S_N_2, N-Ylide formation, ring-opening, deprotonation, SET, and benzyne mechanisms were identified for the ammonium,^378^ imidazolium,^296,320^ piperidinium,^317,379^ carbazolium,^321,322^ and phosphonium^378^ groups (Fig. 15).

**Fig. 16:**
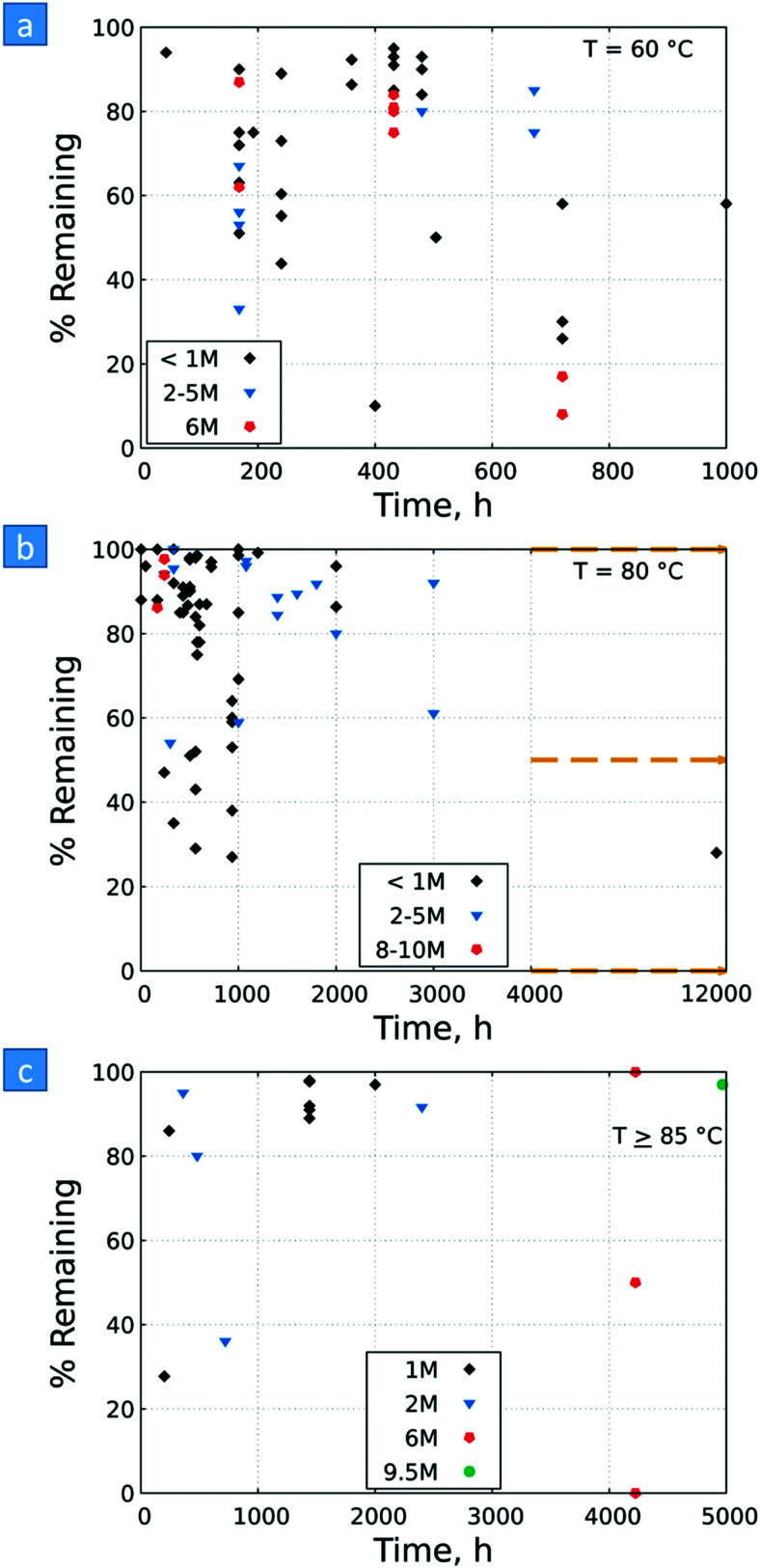
*Ex situ* alkaline stability data of AEMs. The stability is reported as % QA cation remaining *vs.* time of stability test, performed in various base concentrations at constant temperatures of (a) 60 °C, (b) 80 °C, and (c) ≥85 °C. Represented data and underlying sources are given in Table S2 (ESI†).

AEM degradation rate was found to be affected by the concentration of the alkali hydroxide as well as the temperature (Fig. 16). It can be seen that (i) most of the available data is in the 0–2000 h range, significantly lower than the targeted lifetime of the desired AEMWEs; (ii) the degradation rate increases when the temperature increases from 60 to 80 °C or above (Fig. 16). Unfortunately, there are very scarce data published on stability tests longer than 5000 h. Table S1 (ESI†) summarises all details of the stability tests of AEMs.

Despite recent improvements in *ex situ* alkaline stability,^296,320,339,380–382^ AEM *in situ* alkaline stability in *operando* AEMWE is still a major concern, suggesting that maybe more than one single factor should be taken into account.

*Ex situ* long-term tests do not adequately simulate the liquid-electrolyte-free environment of AEMWEs, yielding false or misleading indications of degradation rates. The combination of two effects explains how an anion-conducting ionomer can be ‘stable’ in alkaline solution *ex situ* stability tests, but rapidly degrades during operation.^383^ A new *ex situ* technique to measure AEM degradation in conditions that mimic an *operando* cell environment^384^ as well as new stable cationic groups were recently proposed.^296,321,342^

Concerning the durability of the backbone, Mohanty *et al.* showed that aryl ether bonds in the repeating unit have poor chemical stability in alkaline solutions; backbones without aryl ether bonds [*e.g.*, poly(biphenyl alkylene)s and polystyrene block copolymers] remained stable.^385^ AEM backbone degradation could be triggered by the type of cation functional group, while the cation functional group can be destabilised by the type of backbone used in the AEM.^385–389^ Müller *et al.* recently reported a practical and reproducible *ex situ* method to measure the true alkaline stability of AEMs (interaction between backbone and functional groups)^384^ that simulates the most severe environment inside an *operando* AEM-based device (with combined alkaline, temperature, and controlled hydration environment).

AEMs need to be stable towards dissolved oxygen (DO). DO may indeed promote (through ORR in AEMFCs and OER in AEMWEs, respectively) the reactive oxygen species (ROS) formation,^390^ which in turn, may degrade the AEM polymer.^391,392^ However current methods cannot reasonably mimic operating AEMWE environment, and new methods need to be developed.

### 5.3 Proton exchange membrane water electrolyser

As stated earlier PEMWE uses expensive and scarce materials such as iridium (Ir) and platinum (Pt) at the anode and at the cathode respectively, as well as titanium-based materials in the porous transport layer (PTL). The current PGM loading is 2–5 mg_IrO_2__ cm^−2^ and 1–2 mg_Pt_ cm^−2^ at the anode and cathode respectively, and 100 MW PEMWE would require *ca.* 50 kg of Ir (assuming a typical Ir loading of 2 mg_Ir_ cm^−2^ active area and operation @ 4 W cm^−2^). At today's Ir price of US$203 g^−1^ ^393^ this would correspond to a staggering US$10.15 million for a 100 MW PEMWE, not to speak from the scarcity of Ir and Pt in the Earth's crust.^394^

According to a study from Minke *et al.*^395^ current iridium and platinum production rates are estimated at 7–8 and 200 tonnes per annum respectively,^37^ mined mainly in Canada, Russia, South Africa, United States of America and Zimbabwe, South Africa being the leading producer (70% of the global reserve^396^).

According to Minke *et al.*,^395^ if iridium loading is not significantly reduced and the PGM is not fully recycled (at least 90%), a possible bottleneck in iridium supply is expected as PEMWE installation rates ramp up over a 50 year project (at a linear growth of 2 GW year^−1^ of installed capacity). As an example, if it is assumed that the 2030 EU target of 40 GW of electrolysers are mainly PEMWE with a current loading of 0.50 g_Ir_ kW^−1^ (0.5 kg_Ir_ MW^−1^ or 500 kg_Ir_ GW^−1^), then 20 tonnes of iridium would be required. Table 6 shows Ir and Pt loading, current and power density and electrode area targets for PEMWE.

**Table 6:** Ir and Pt loading, current and power density and electrode area targets for PEMWE. Modified from ref. 225 and 395

Parameter	2020 status	2020 target	2035 target	Future
Ir (mg cm^−2^)	2–5	1	0.2–0.40	0.05–0.2
Ir (g kW^−1^)	<2.5 (0.33/0.5/0.67)	0.40	0.05–0.4	0.01–0.4
Pt (mg cm^−2^)	1–2	1	0.5	0.05
Pt (g kW^−1^)	0.5–1	0.5	0.25	0.1
Current density (A cm^−2^)	2	2	3	5
Power density (W cm^−2^)	3	3	8	10
Electrode area (m^2^)	0.12	—	—	0.50

Due to price volatility and scarcity of PGM's, one major objective is to drastically reduce their loading by a factor of at least 40, in the case of Ir, and in the long-term to replace them with PGM-free catalysts. The former can be achieved by developing (i) non-carbonaceous high surface area (HSA) supported catalyst, (ii) alloy PGMs with other abundant materials (*e.g.*, other transition metals), or increasing the (iii) catalyst surface area by using better manufacturing methods, and the (iii) catalyst utilisation in membrane electrode assemblies by using better dispersion and deposition techniques.^1424^

**Fig. 17:**
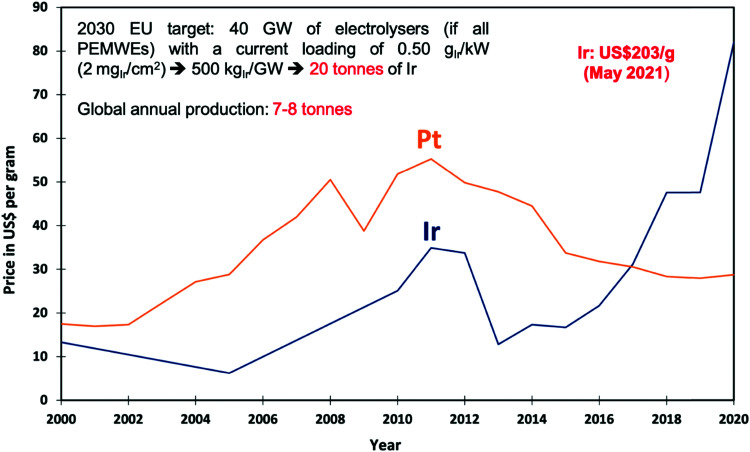
The evolution of platinum and iridium price (US$ g^−1^) in the period of 2000–2020.

Fig. 17 shows the evolution of platinum and iridium cost (US$ g^−1^) in the period of 2000–2020. Historically Pt has been more expensive than iridium. However, since 2017, Ir price surpassed that of Pt (since 2015 the price of Ir has increased by *ca.* 500%; in May 2021, the price of Ir had increased by 20-fold since 2013).

### 5.4 Solid oxide electrolysis cell

SOEC commonly requires high operating temperatures (≥700 °C), because the yttria-stabilised zirconia (YSZ) electrolyte only displays excellent ionic conductivity at these temperatures. However, during long-term operations, YSZ suffers from thermomechanical and thermochemical issues, particularly under shutdown and temperature ramping conditions, which lead to increased degradation rates and shorter stack and system lifetimes. There are also other issues related to SOEC stack degradation *e.g.*, sealing failure at higher differential pressure, electrode contamination originating from external components (*e.g.*, piping), interconnects and sealing. SOECs are today only deployed at the <1 MW scale, although some current demonstration projects have already reached 1 MW. Deploying SOEC at large scale would require larger SOEC cells than currently used *e.g.*, up from 300 cm^2^ to over 1000 cm^2^, which makes them more susceptible to failure.

SOEC is mainly made of abundant and low-cost minerals (*e.g.*, Y, Zr, Sr, La, Mn, Ni) and ceramic materials (no rare metals/critical raw minerals are employed). However, SOEC could experience supply risk, as roughly 95% of the supply for all their materials currently originates from China.^225^ Exact minerals amount per 1 MW cannot be found in the literature but as an example, 1 TW of SOEC would require 1 months and 21 months’ worth of global ZrO_2_ and Y_2_O_3_ production, respectively.

Therefore, the R&D focuses on improving the electrolyte conductivity matching the thermal expansion coefficient of both electrodes, ensuring minimal reactant crossover and optimising chemical and mechanical stability. Decreasing the operating temperatures (≤600 °C) is also an option, opening the way to proton-conducting ceramic electrolysers.

### 5.5 Proton conducting ceramic electrolyser

Proton conducting ceramic electrolysers (PCCEL) exhibit significant proton conductivity at intermediate temperatures in the range of 300–600 °C^397^ which was firstly demonstrated in 1981, by Iwahara *et al.*^398^ Recently, some research groups^399,400^ proved that the technology could be scaled up. Like in SOEC, the electrolyte material is crucial. ABO_3_ perovskites (*e.g.* Y,Yb-doped-Ba(Ce,Zr)O_3−*δ*_) are the most widely-used electrolytes, because they are chemically stable and exhibit high proton conductivity. Examples of perovskites developed include Y and Yb-doped barium zirconate (BZY and BZYb), Y and Yb-doped barium cerate (BCY and BCYb), Y and Yb-doped zirconate-cerate solid solution (BCZY and BCZYb), (iv) Y and Yb-codoped zirconate-cerate solid solution (BCZYYb).^401,402^

In general, PCCELs have similar issues to that of SOECs *i.e.*, problems in cell fabrication and material integrity. As an example, the electrode support structural and compositional homogeneities are critical for large-size cell fabrication; developing novel materials possessing (i) high proton and electronic conductivities, (ii) chemical compatibility and stability with the electrolyte, and (iii) similar thermal expansion coefficients with the electrolyte is the current challenge.^397^

The overall strategy is to decrease both the ohmic and polarisation resistance component, so as to improve (i) the electrolyte conductivity, (ii) the chemical and mechanical stability, (iii) the understanding of material properties at basic levels (in order to achieve “ideal” microstructures), (iv) the manufacturability (at low-cost), (v) match the thermal expansion coefficient of both electrodes, and (vi) optimise the operating conditions.

In summary for this section, key success factors for water electrolysers are as follows: (i) lower costs, (ii) higher performance, (iii) higher efficiency, (iv) higher durability and (v) lower OPEX. High volumes will definitely decrease the cost of electrolysers and governmental support for R&D in supporting the development of new low-cost highly performing and durable materials is key.

## 6. Status of PGM-based HER and OER electrocatalysts and their alloys

Due to the importance of water electrolysis and related electrocatalysis, many in-depth and excellent reviews on PGM-based HER and OER electrocatalysts have been recently published.^133,403–405^ This section will attempt to capture general findings in recent advances in developing strategies to improve HER and OER noble metal-based electrocatalysts.

Generally, one of the most critical barriers for electrochemical water splitting is to use high-performance and durable electrocatalytic material that allow both fast HER and OER reaction kinetics and low overpotentials. The choice of HER and OER catalysts in acidic, neutral and alkaline electrolytes is important as the HER and OER reaction kinetics and overpotential will differ. For example, the HER activity in acidic electrolytes is usually 2 to 3 orders of magnitude higher than in alkaline ones, because the water dissociation step is unnecessary in an acidic environment and the high concentration of H^+^ ions results in faster H–H coupling than at high pH.

### 6.1 PGM-based HER and their alloys

HER activity is related to hydrogen adsorption (H_ad_) in acidic media, which is composed of either Volmer/Heyrovsky (eqn (6)) or Volmer/Tafel (eqn (5)) steps. In alkaline electrolytes, the Heyrovsky (eqn (4)) and Volmer (eqn (7)) steps occur involving hydroxyl adsorption (OH_ad_), and water dissociation, breaking the strong covalent H–O–H bond. In acidic electrolytes, H_3_O^+^ adsorption is much stronger than water adsorption for alkaline conditions. The HER kinetics are highly dependent on various parameters such as the electrode material, the nature of the electrolyte and the crystalline nature and orientation of the electrode (*i.e.*, single-crystal, polycrystalline or amorphous).^406^ Hydrogen adsorption and desorption on the electrode surface are two successive steps in HER electrocatalysis. However, these two steps compete in nature: a catalyst surface with insufficient bonding strength to hydrogen atoms cannot efficiently adsorb the reactant to initiate the HER; whereas a catalyst surface having too high bonding strength would have difficulty in releasing the product toward the completion of the HER. Therefore, the ideal HER electrocatalysts should have well-balanced hydrogen adsorption and desorption properties.^407^ This is entirely in line with the Sabatier principle, which states that to have high catalytic activity, the interaction between reactants and catalysts should neither be too strong nor too weak.^408^ If the interaction is too weak, the catalyst surface will hardly bind the M–H intermediate species, resulting in slow reaction kinetics. If the interaction is too strong, the catalyst active sites will be blocked by intermediate species, leaving no active sites available for new reactant molecules that would continue the reaction.^407,409^ The Sabatier principle usually yields a “volcano” curve when plotting the activity *versus* the M–H bonding energy for different metals.^98^Fig. 3 (Section 2.2) illustrates the relationship between the logarithm of the exchange current density (log(*j*_o_)) and the energy of hydride formation (*E*_M–H_), which was observed by Trasatti^98^ in the form of a “volcano” curve:^409,410^ the HER exchange current density changes by the electrode material, with Pt-group materials on the top of the volcano plot. For alkaline electrolytes, the objective is to increase the M–H_2_O bond energies to help water adsorption and water dissociation, leading to effective HER kinetics. In alkaline electrolytes, the HER kinetics are sluggish when compared to acidic solutions, and four parameters need to be considered when designing the HER catalysts: (a) water adsorption, (b) water dissociation capability, (c) M–H binding energy, and (d) aqueous OH^−^ on the active sites.

#### 6.1.1 HER on Pt

**Fig. 18:**
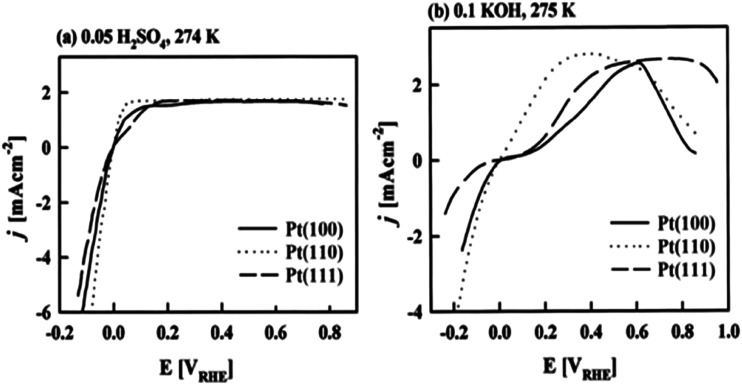
Polarisation curves for hydrogen evolution/oxidation on Pt(*hkl*), with scan rate of 20 mV s^−1^, in (a) acid and (b) alkaline media. Reproduced with permission from ref. 409 (copyright MDPI 2020), 410 (copyright American Chemical Society 1997),^412^ (copyright RSC 1996).

PGM-based catalysts have usually acted as the benchmark for HER, as they exhibit relatively high HER activity. Since the highest |*j*_o_| is exhibited by Pt, atomic-scale studies of the HER rate dependence on the Pt single-crystal surfaces’ atomic-scale morphology have been in the research focus for many years. Marković *et al.*^411^ first illustrated the HER in acid solutions as a surface-sensitive process, suggesting its rate depends on the Pt crystal orientation, as shown in Fig. 18.^409,411^ This might seem intuitively obvious today when *E*_ads(H)_ is commonly used in HER activity studies. This energy depends on the atomic-scale structure of the surface, its orientation, the coordination number of the surface atoms and its reconstruction;^409^ however, early HER studies on Pt did not reveal HER's rate dependence on surface orientation.^409^ Marković *et al.*^411^ observed that the catalytic activity both in acidic and alkaline solutions decreases in the order Pt(110) > Pt(100) > Pt(111). The order is the same for both media; however, the absolute rates are quite different.

The dependency of the HER rate on the crystallographic orientation of Pt in acidic media is also shown in Table 7.^409^ For platinum polycrystalline, experiments in acid solutions show that at low overpotentials the recombination reaction, or Tafel step, is rate-determining following the fast-initial discharge reaction or Volmer step. A Tafel slope *b* ∼30 mV dec^−1^ is measured at this potential range. As the overpotential is increased, the coverage of absorbed hydrogen atoms approaches saturation. This leads to accelerated atom–atom recombination. As a result, the discharge reaction or Volmer step becomes rate-determining with a measured Tafel slope *b* ∼ 120 mV dec^−1^.^407^ HER is also surface-sensitive on Pt in alkaline solutions. This feature has been shown in Fig. 18. According to Marković *et al.*,^412^ Pt(100) exhibits a two-step Tafel slope, starting from 55 mV dec^−1^ shifting to 150 mV dec^−1^, Pt(110) exhibits a slope of 75 mV dec^−1^ that shifts to 140 mV dec^−1^, and Pt(111) is reported to exhibit a Tafel slope of 140–150 mV dec^−1^ with no transition in a 0.1 M KOH solution.^413^

**Table 7:** Hydrogen evolution reaction (HER) on Pt single-crystal surfaces, in acid solutions, and the corresponding available data: Tafel slope (*b*), exchange current density (*j*_o_) at given temperatures (*T*), activation energy (*E*_a_), number of electrons transferred (*z*) and identified mechanism and rate-determining step (rds).^409^ Modified from ref. 263. Copyright MDPI 2020

Single crystal	*b* (mV dec^−1^)	*z*	*j*_o_ (mA cm^−2^) (*T*)	*E*_a_ (kJ mol^−1^)	Mechanism and RDS
Pt(100)	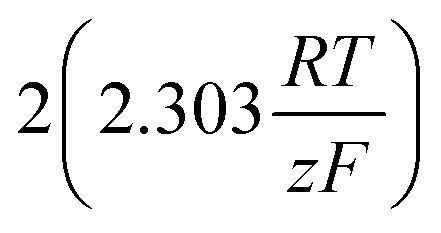	1	0.36 (274 K)	12	Heyrovsky–Volmer
			0.60 (303 K)		
			0.76 (333 K)		
Pt(110)	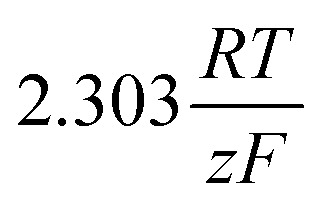	2	0.65 (274 K)	9.5	Tafel–Volmer
			0.98 (303 K)		
			1.35 (333 K)		
Pt(111)	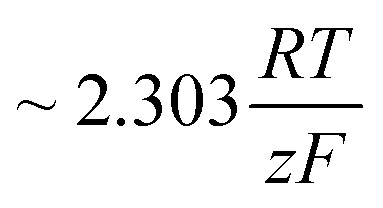	1	0.21 (274 K)	18	Tafel–Volmer, Heyrovsky–Volmer
			0.45 (303 K)		
			0.83 (333 K)		

Conway *et al.*^414^ illustrated that the Tafel slopes and mechanisms of the HER at Pt in acid and alkaline solutions are rationalised in terms of the observed overpotential-deposited (OPD) H coverage behaviour in relation to a parallel pathway of electrochemical and recombination-controlled desorption steps. The best electrocatalytic activity of Pt electrodes for the HER arises in acid solutions where the nominal extent of measured OPD H coverage is found to correspond to the apparent formation of 8 equivalent monolayers; one possible interpretation of that result is in terms of hydride formation in the near-surface region of Pt. As a result, the OPD M–H adsorption bond can be weakened so that the recombination step with low Tafel slope becomes the favoured desorption step and characterises the kinetics at active Pt electrodes. Based on Conway *et al.*,^414^ Tafel slopes of 36–68 mV dec^−1^ at low overpotentials (*η* ≤ 0.05) followed by 125 mV dec^−1^ at high overpotentials ∼*η* > 0.075 V in a 0.5 M H_2_SO_4_ electrolyte for bulk Pt disc electrode have been reported. This indicates the Tafel slope is indeed potential-dependent and, in turn, coverage dependent. Conway *et al.*^414^ also showed that the Pt electrode becomes a poorer electrocatalyst for the HER as the cathodic polarisation time increases. In the same paper, the Tafel plot for the same Pt electrode after 30 min cathodic polarisation at ∼*η* = 0.050 V in 0.5 NaOH is a straight line with a slope of 125 mV dec^−1^ throughout the potential range measured. According to Conway *et al.*,^414^ the decrease of activity of the Pt electrode with time in alkaline solution is appreciably more rapid than in acid solution. Shinagawa *et al.*^413^ confirmed that the Tafel slope measured for Pt electrodes in alkaline solutions is around 120 mV dec^−1^, indicating that the Volmer or the Heyrovsky step is the RDS.

#### 6.1.2 HER on PGM and their alloys

Various strategies have been adopted to reduce the loading of platinum and other PGMs such as Pd, Ru, Ir and Rh as they are expensive and scarce. Examples include alloying them or producing core–shell structures with low cost and abundant metals such as transition metals (TM *e.g.*, Ni, Ti, Zr *etc.*) without compromising on the performance and catalyst utilisation. To boost the HER activity, especially in alkaline solutions, alloying PGMs like Pt with TMs can greatly improve catalyst utilisation by modifying the alloy electronic structure, in turn favouring efficient HER. For example, it was found that the downshift d-band centre of Pt weakens the adsorption energy of hydroxyl species (OH*) on the surface Pt atom.^415^ For alkaline electrolytes, several researchers have worked on improving the M–H_2_O by developing new structures such as PGM nanoparticles on nitrogenated carbon (PGM@C2N).^416^

To improve the slow HER kinetics, dual-active electrocatalytic sites *i.e.*, bifunctional HER catalysts need to be engineered allowing better water adsorption/dissociation, hydrogen adsorption/desorption, and OH^−^ desorption. This is often achieved by developing hybrid structures, such as PGM (Pt/Ir/Ru) on Ni(OH)_2_ surface, PGM (Pt)-decorated Ni_3_/N nanosheets, NiO_*x*_/Pt_3_Ni interfaces, PGM (Ru)@C2N, Co-decorated PGM (Ru) nanosheets, and PGM (Pd)–CN_*x*_ composites.

Strncnik *et al.*^417^ found that Li^+^–Ni(OH)_2_–Pt interface exhibited excellent HER activity in alkaline conditions. It was observed that (i) the edges of the Ni(OH)_2_ cluster promote water dissociation and the Pt surface adsorbs the hydrogen intermediates for recombining into molecular hydrogen, and (ii) the introduction of Li^+^ further strengthens the water dissociation ability because of the distributed HO–H bond. The same research group also found that 3D transition metals (Ni, Ti and V) exhibited similar HER activity in both alkaline and acidic electrolytes due to surface oxides aiding water dissociation.^418^

Another strategy is to dope PGM-based alloy catalyst with N, such as PtNi(N). Xie *et al.*^420^ showed that PtNi(N) exhibited superior kinetics when compared to Pt–Ni and Pt/C with Tafel slopes of *ca.* 29 mV dec^−1^, ∼*η* = 0.013 V (@ 10 mA cm^−2^) and with no potential changes at *j* = 40 mA cm^−2^ for 10 hours in 1.0 M KOH. They attributed the excellent water dissociation kinetics to N decreasing the electron density around Ni site, yielding strong interaction between N and Ni.

### 6.2 PGM-Based OER and their alloys

The OER is the key process that controls the overall efficiency of electrochemical water splitting. This is because the OER is more kinetically sluggish as this reaction is a four-electron transfer process, while the HER needs only two electrons. Binding energy (M–O, M–OH, and M–OOH) is mainly the rudimentary benchmark for OER performance that is usually tuned by electronic and geometric structural “engineering”. Overall, PGM play crucial roles because of their high activities and good selectivity.^419^ Under acidic environments, the PGM catalytic activities decrease in the order of Ru > Ir > Rh > Pd > Pt > Au, while the structural durability follows the order of Pd > Pt > Rh > Ir > Au > Ru. The most efficient OER catalysts are so far combined Ir and Ru electrocatalysts as it possesses excellent dissolution resistance in acidic conditions. Their oxides such as RuO_2_ and IrO_2_, are considered as state-of-the-art electrocatalysts for the OER. To date, many Ir and Ru-based metals, alloys, and oxides have been developed for the OER under acidic environments.^419^

#### 6.2.1 OER on Pt

While Pt is the best oxygen reduction reaction (ORR) catalyst, it does not have good catalytic activity towards the OER, due to the formation of Pt oxides on its surface at high overpotentials.^420,421^ According to the observations from Willsau *et al.*^422^ only two-dimensional Pt surface oxide (O=O), which is a thin oxide layer, participates in the OER, while Pt(ii) and/or Pt(iv) oxide layer does neither take part in the acidic nor alkaline OER. In 1991, Damjanovic *et al.*^423^ proved that the OER activity of Pt strongly depends on the Pt oxide film thickness. They also confirmed the OER Tafel slopes are always greater than 120 mV dec^−1^ in acidic solutions at all thicknesses and potentials. Reier *et al.*^424^ investigated the OER activity of Pt bulk and Pt nanoparticles in 0.10 M HClO_4_ and obtained Tafel slopes of 145 mV dec^−1^ and 210 mV dec^−1^ for Pt bulk and Pt nanoparticles, respectively, results in excellent agreement with Damjanovic *et al.*'s findings. The experimentally observed high Tafel-slope illustrates additional contributions from processes with exponential current–potential dependency, probably related to the formation of Pt oxide layers.^423,424^

In 1992, Damjanovic *et al.*^425^ reported that the OER at Pt in alkaline solutions follows two *E*–log(*j*) relationships (Tafel behaviour). At low current densities, the Tafel slope of Pt is close to 60 mV dec^−1^, and at high current density to 120 mV dec^−1^. In the high current density region where the Tafel slope is 120 mV dec^−1^, the reaction rates are strongly affected by the thickness of the anodically formed oxide film during electrode pre-treatment at high current density or at high electrode potentials.^424^ In contrast, in the low current density region where the slope is 60 mV dec^−1^, the rates are not affected by the film thickness. In alkaline and acid solutions, an 8–15 Å thick anodic oxide or hydroxide film was found to cover the Pt electrode in the potential region of the OER.^424^ These oxide films are electronic insulators^424–426^ and electrons required for the OER are transferred through the films by electron tunnelling process.^424^ They also observed a decrease with the thickness of the oxide film in the rates in alkaline solutions at high current densities.^424^ Experimental parameters at high and low current densities based on Damjanovic *et al.*'s observations are summarised in Table 8.^425^

**Table 8:** Summary of kinetic parameters for the OER at Pt.^425^ Reproduced with permission from ref. 278. Copyright Elsevier 1992

Electrolyte type	*b* (mV dec^−1^)	*z*
Acidic	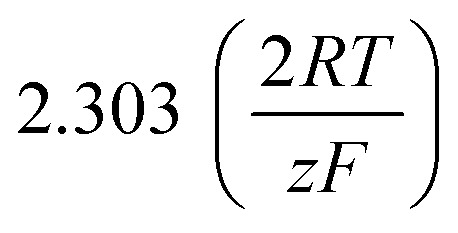	1
Alkaline (at low current densities)	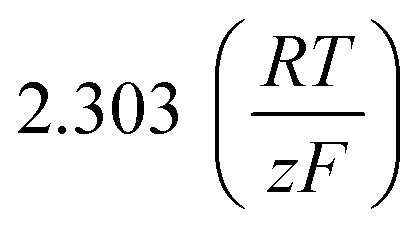	1
Alkaline (at high current densities)	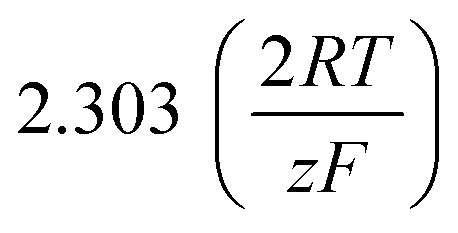	1

**Fig. 19:**
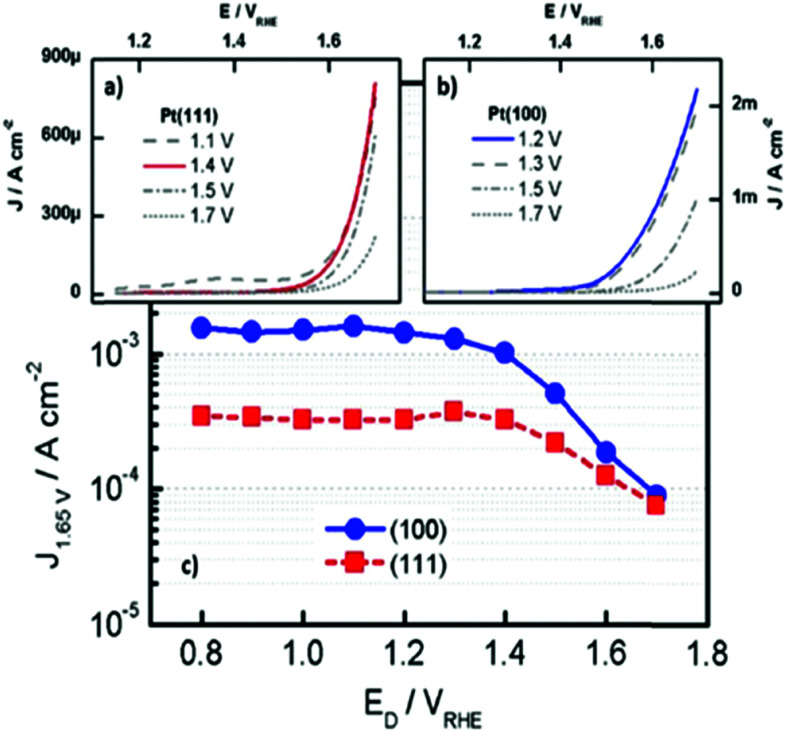
LSV curves of (a) Pt(111), (b) Pt(100) in 0.1 M HClO_4_ with scan rate of 50 mV s^−1^. (c) Plot of the current density at +1.65 V *vs.* RHE (*j*_+1.65V_) in the LSV as a function of the applied potential. Reproduced with permission from ref. 427. Copyright Wiley 2019.

Bizzotto *et al.*^427^ investigated the OER structure sensitivity on Pt(111) and Pt(100) in 0.10 M HClO_4_ solution. According to their findings, Pt is structure-sensitive and Pt(100) is significantly more active than Pt(111) towards OER. In their study, the OER activity was evaluated based upon a series of polarisation curve experiments, and the current density values were monitored at a potential of +1.65 V *vs.* RHE, *i.e.*, *j*_*+*1.65V_. They considered two different potential regions (Fig. 19). In the first potential region ranging from +0.80 V to +1.30 V *vs.* RHE, *j*_*+*1.65V_ was found to be 4 to 5 times larger on Pt(100) than on Pt(111). They estimated the onset potentials of +1.4 V *vs.* RHE and +1.5 V *vs.* RHE for Pt(100) and Pt(111), respectively. Tafel slopes of 116 mV dec^−1^ for Pt(100) and 132 mV dec^−1^ for Pt(111) were calculated in the potential range of +1.40 to +1.60 V *vs.* RHE; the higher slope for Pt(111) was related to additional (overlapping) processes to the OER, possibly due to surface oxidation.^423–427^ They concluded that a potential region exists where the OER is structure sensitive, and no insulating oxide layer is growing. In this potential region, the Pt(100) surface is significantly more active than the Pt(111) surface, although at very high oxidative potentials, *i.e.* +1.70 V *vs.* RHE, the structure sensitivity disappears, and the activity of the two single crystals becomes the same (Fig. 19).^427^

In another study by Lopes *et al.*,^428^ the relationships between atomic level structure and stability/activity of Pt surface atoms in both acidic (0.1 M HClO_4_) and alkaline media (0.1 M KOH) was investigated. They found that the degree of stability of Pt(*hkl*) surfaces (Pt(110) ≪ Pt(100) < Pt(111)) at early stages of oxide formation is proportional to the coordination of surface atoms, as expected from oxophilicity trends. They also investigated functional links between the activity of the OER and the stability of Pt(*hkl*) surface atoms. According to their studies, the amount of dissolution was directly proportional to the OER activity *i.e.* the OER activity increased in the order of Pt(100) > Pt(110) > Pt(111) which is the same order of instability.^428^ These findings were in excellent agreement with those from Bizzotto *et al.*,^427^ as the least stable surface is the most active towards the OER.

#### 6.2.2 OER on PGM and their alloys

As stated earlier, RuO_2_ is the most active OER electrocatalyst, but the dissolution rate of Ru is faster than Ir in acidic environments. IrO_2_ exhibits a higher stability than RuO_2_ and good activity in acidic media, but its cost is 10–15 times higher than that of RuO_2_. Therefore, several strategies have been adopted to enhance the activity and stability of the Ru- and Ir-based catalysts under acidic conditions by either engineering their size, shape, elemental composition or employing stable substrate materials.

**Fig. 20:**
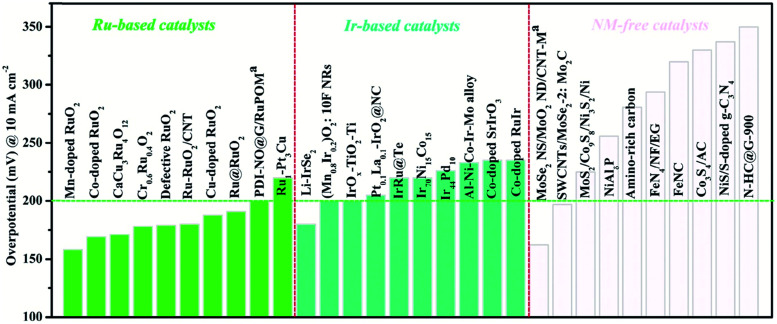
Comparison of OER overpotentials at 10 mA cm^−2^ for the ten most promising catalysts in each category (Ir-, Ru- and PGM-free based catalysts) in acidic media. (a: the overpotential @ 20 mA cm^−2^). Reproduced with permission from ref. 419. Copyright Elsevier 2020.

For example, for acidic media, well-developed structures containing Ir and Ru such as nanosheets, nanotubes and nanoparticles, as well as alloys (containing non-PGMs) and oxides (*e.g.*, as amorphous, perovskite, pyrochlore and hollandite) on carbonaceous/non-carbonaceous substrates have exhibited excellent catalytic activity, catalyst utilisation and durability towards the OER.^419,429–440^ In general, the catalytic activity towards the OER in acidic electrolytes strongly depend on the electrocatalyst size, surface area, porosity, and the crystal and electronic structure arrangements with other elements to create heterostructures. Chen *et al.*^419^ reviewed the current state-of-the art OER catalysts in acidic environments (Fig. 20).

As a matter of fact, the very harsh environment of the OER anode in a PEMWE leaves very little hope to discover catalysts alternative to IrO_2_ that would be durable and active. Implementing iridium oxide (IrOx) nanoparticles at PEMWE anodes requires developing electron-conductive supports, that are stable in OER conditions, and exhibit high specific surface area and porous structure adapted to gas–liquid flows. Of course, in this seek, carbon can play no role as it will be irremediably oxidised into CO_2_. That's why, metal oxides are under intense focus since a decade, more specifically substoechiometric (*e.g.*, Magnely-phases: Ti_4_O_7_, Ti_5_O_9_) or doped metal oxides (*e.g.*, Sb-doped SnO_2_ or Nb-doped TiO_2_). Previously used in PEMFC for ORR applications^441–444^ these metal oxides (*e.g.*, TiO_2_ and SnO_2_) have shown some robustness *versus* oxidation/metal leaching/dissolution. Their Hachille heel however laid in their propensity to passivate at their surface (becoming less electron conductive) and to dissolve/degrade upon incursions to reducing potential. In particular, the doping element was found fairly unstable in operation.

For OER applications, incursions to reducing potentials are avoided, which leaves hope to obtain more stable (doped) metal oxide structures. For example, Claudel *et al.* evaluated several doped SnO_2_ aerogels (IrOx/doped SnO_2_), and assessed their electrocatalytic activity and electrochemical stability towards the OER.^34,445^ Using a flow cell connected to an inductively-coupled mass spectrometer (FC-ICP-MS), they revealed that the corrosion-resistance of the doping element controls the long-term OER activity of the material. In addition, the doping element concentration in the host SnO_2_ matrix controls the electron conductivity of the material, hence its propensity to be practically active. The study further demonstrated that Sb-doped SnO_2_ type supports continuously dissolve in OER operation. On the contrary, Ta-doped or Nb-doped SnO_2_ supports are more stable under acidic OER conditions, provided their doping concentration is appropriate. Although these studies open a door to nanostructured IrO_*x*_ OER catalysts (hence to large depreciation of the materials’ cost of a PEMWE MEA), there is still large room for practical improvements. Developing active and durable nanostructured IrOx catalysts for OER in acidic media is therefore still a mandatory research topic if one wants to deploy PEMWE at large scale.

The previous paragraphs showed that PGM-based catalysts are still the norm in PEMWE (and the (almost) only ones that are active and durable in practical acidic operation). In the search for alternative catalyst materials, the present review will focus specifically on catalysts for alkaline systems, for which the available chemistries are far richer (see following sections). Of course, some of these chemistries can find applications in PEMWE cells, as will be specified as well.

## 7 Status of PGM-free based HER and OER electrocatalysts and their alloys

Due to the low cost and (in most cases) large presence of non-PGM's in the earth's crust, non-PGM-based water-splitting electrocatalysts are of particular interest.^71,446–451^ The essential groups of non-precious metal-based compound classes (transition metal dioxides, spinels, perovskites, transition metal layered double hydroxide, metal-non-metal-based compounds with the main group elements of groups 3, 4, 5 and 6) are discussed in the following subsections. Water splitting promoted on steel surfaces is considered separately (subsection 7.6). Tables 9–11 summarise the OER key data of some recently-developed PGM-free OER electrocatalysts, HER electrocatalysts respectively.

**Table 9:** OER activity of recently reported and highly active NiFe- and CoFe-based LDH and oxyhydroxide catalysts, including trimetallic and multimetallic variants

Catalyst	Substrate	Catalyst loading (mg cm^−2^)	Electrolyte	*η* (mV) @ *J*_geo_ = 10 mA cm^−2^	Ref. (year)
CoFe LDH	GC	0.21	1 M KOH	331 (±3)	823 (2016)
Gelled-FeCoW oxyhydroxide	GC	0.21	1 M KOH	223 (±2)	823 (2016)
Co_5_Fe_3_Cr_2_ (oxy)hydroxide	GC	0.2	1 M KOH	232	895 (2021)
CoCuFeMo (oxy)hydroxides	Cu foil	1	1 M KOH	199	896 (2021)
Ni_6_Fe_2_Cr_1_ LDH	GC	0.2	1 M KOH	280	897 (2018)
Ni_3_Fe_0.5_V_0.5_	CFP (0.2 cm^2^)	—	1 M KOH	200	899 (2018)
Ni–Fe–Mo (oxy)hydroxides	NF	1.6	1 M KOH	238	900 (2018)
NiFeCe-LDH/CNT	GC	0.2	1 M KOH	227	901 (2018)
NiFeMn-LDH	CFP	0.2	1 M KOH	310	902 (2016)
NiFe	CFP	1.67	1 M KOH	248	906 (2020)
NiFeMo	CFP	1.67	1 M KOH	201	906 (2020)
NiFeMoW	CFP	1.67	1 M KOH	205	906 (2020)
FeCo	CFP	1.67	1 M KOH	266	906 (2020)
FeCoMo	CFP	1.67	1 M KOH	233	906 (2020)
FeCoMoW	CFP	1.67	1 M KOH	212	906 (2020)
Pororus monolayer NiFe LDH	GP	0.35	1 M KOH	230	846 (2019)
NiFe LDH	GC	0.1	1 M KOH	270	894 (2019)
Ni_*x*_Fe_1−*x*_Se_2_ derived	NF (0.2 cm^2^)	—	1 M KOH	195	861 (2016)
Ni_3_FeN	GC	0.35	1 M KOH	280	866 (2016)
Fe–Ni–F	GC	0.714	1 M KOH	225	859 (2019)
NiFe LDH	GC	0.1	0.1 M KOH	348	1775 (2020)
CoFe LDH	GC	0.1	0.1 M KOH	404	1775 (2020)
Ni_2.5_Co_0.5_Fe LDH	NF	0.3	0.1 M KOH	275	905 (2016)
NiFeS	GC	0.25	0.1 M KOH	286	858 (2017)
NiFe LDH (pristine)	NF	—	1 M KOH	182	113 (2019)
Aged–NiFe LDH	NF	—	1 M KOH	184	113 (2019)

**Table 10:** Electrochemical characteristics of recently developed steel-based OER electrocatalysts

OER catalyst	Type of activation	Average overpotential at current density (mA cm^−2^)	Tafel slope (pH)	Faraday efficiency at current density (mA cm^−2^)	Ref.
Mild steel	*Ex situ* inco type 123 paint	200 mV (100) in 30 wt% KOH at 80 °C	35–40 mV dec^−1^ (>14)	—	1274
AISI 316L	*In situ*	500 mV (20) in 5 M LiOH	40 mV dec^−1^ (>14)	—	1277
AISI 316L	Unmodified	370 mV (10) at pH 14	30 mV dec^−1^ (14)	96% (10) at pH 14	1238
S235 steel	*Ex situ*	347 mV (2) at pH 13	58.5 mV dec^−1^ (13)	67% (2) at pH 13	1288
	Chem. Oxidation with air/chlorine	462 mV (1) at pH 7	68.7 mV dec^−1^	82% (10) at pH 13	1304
	Phosporisation	326 mV (10) at pH 13			
AISI 302 steel	Unmodified	400 mV (6.3) at pH 14	33 mV dec^−1^ (14)	—	1275
AISI 304 steel	*Ex situ*	500 mV (0.65) at pH 7	—	—	1281
	Chem. Oxidation with air/chlorine	260 mV (1.5) at pH 13			
AISI 304 steel	*Ex situ*	260 mV (10) at pH 14	41 mV dec^−1^ (14)		366
	Chem. Oxidation with KOH/OCl^−^	288 mV (50) at pH 14			
AISI 304 steel	*Ex situ*	269 mV (10) at pH 13	49 mV dec^−1^ (13)	75,5% (10) at pH 13	1286
	Electro-oxidation	212 mV (12) at pH 14			
AISI 304 steel	*Ex situ*	504 mV (10) at pH 6.7–7.3	138 mV dec^−1^	97% (10) at pH	1287
	Electro-oxidation			7	
AISI 304 steel	*Ex situ*	360 mV (100) at pH 14	46 mV dec^−1^ (14)	—	1298
	Etching + electro-oxidation in KRuO_4_				
AISI 316L	*Ex situ*	330 mV (100) at pH >14	35 mV dec^−1^	—	1454
	Electro-oxidation				
AISI 316L	*Ex situ*	270 mV (100) at pH >14	40 mV dec^−1^ (>14)	—	1289
	Electro-oxidation				
AISI 316L	*Ex situ*	290 mV (10) at pH 14	35 mV dec^−1^ (14)	100% (10)	1295
	Electro/chem-oxidation				
AISI 302	*Ex situ*	300 mV (10) at pH 14	35 mV dec^−1^		1222
	Chem-oxidation				
AISI 304	*Ex situ*	293 mV (500) at pH 14	36 mV dec^−1^	98.5%	1229
	Thermoselenisation				
AISI 304 mesh	*Ex situ*	230 mV (20) at pH 14	36 mV dec^−1^	—	1228
	Hydrothermal treatment	173 mV (100) at pH 14	65.7 mV dec^−1^		1308
	Electro-oxidation				
AISI 316 mesh	*Ex situ*	319 mV (100) at pH 14	70 mV dec^−1^ at pH 13	—	1307
	Electrochem.				
X_20_CoCrWMo_10-9_	Electro-oxidation	298 mV (10) at pH 7	141 mV dec^−1^ (7)	75,6% (10) at pH 7	40
	Electro-oxidation	230 mV (10) at pH 13	47 mV dec^−1^ (13)	83% (5) at pH 7	1324
	Oxidation + Li^+^ inter-calation	574 mV (10) at pH 1	36 mV dec^−1^	95,2% (10) at pH 1	
		40 mV (10) at pH 7		88.7% (10) at pH 7	
Ni42 steel	Electro-oxidation	491 mV (4) at pH 7	151 mV dec^−1^ (7)	99,4% (2) at pH 7	1239
		254 mV (10) at pH 13	72 mV dec^−1^ (13)	79% (10) at pH 1	1327
		215 mV (10) at pH 14	127 mV dec^−1^ (1)		
		445 mV (10) at pH 0			
Ni42 steel	Modified in hematite/H_2_SO_4_ suspension	31 mV (30) at pH 0	188.7 mV dec^−1^ (0)	93% (30) at pH 0	1328

**Table 11:** Electrochemical HER characteristics of recently developed electrocatalysts

HER catalyst	Overpotential (*η*) in mV HER (*j* in mA cm^−2^; pH)	Tafel slope (pH)	Faraday efficiency HER (pH)	Ref.
Pt on glassy carbon	65 (20; 0)	—	92 (0)	1257
Commercial Pt/C	40 (20; 14)	—	—	910
	50 (100; 14)			
	100 (15; 9,5)			
NiO/Ni CNT on Ni foam	100 (100; 14)	51 mV dec^−1^ (14)	—	910
NiO/Ni core shell NP on CNT	100 (10; 14)	82 mV dec^−1^ (14)	—	910
	100 (2.5; 9.5)			
Ni_2_P	100 (10; 0)	81 mV dec^−1^ (0)	100 (0)	1259
Fe	360 (10; 13)		105.2 (13)	911
NiSe	185 (50; 14)	64 mV dec^−1^ (14)	100 (14)	912
CoP	110 (10; 0)	64 mV dec^−1^ (14)		1260
		41 mV dec^−1^ (0)		
Co_2_P	110 (10; 0)	52 mV dec^−1^ (14)	100 (14)	1260
		45 mV dec^−1^ (0)		
Pt–MoS_2_	35 (10; 0)	54 mV dec^−1^ (0)		913
NiCo_2_S_2_	305 (100; 14)	89 mV dec^−1^ (14)		914
		141 mV dec^−1^ (14)		
Modified steel Ni42	189 (10; 0)	198 mV dec^−1^ (7)	101.8 (13)	1237
	268.4 (10; 1)	72 mV dec^−1^ (13)		
	333 (10; 13)	118 mV dec^−1^ (0)		
	299 (10; 14)	81 mV dec^−1^ (1)		
	275 (10; 14.6) (at 343.15 K)			
Steel Ni42 anode in hematite/H_2_SO_4_ suspension	370 (30; 0)	—	83.1 (0)	1328
Steel AISI 434	315 (10; 14)	121 mV dec^−1^ (14)	—	1266
Sulphurised steel AISI 316	136 (10; 14)	147 mV dec^−1^ (14)	100 (14)	1262
	280 (50; 14)			
Steel 316L	340 (1.3; 4)	—	91.4 (4)	1249
N,P-Doped AISI 304 steel mesh	230 (12; 14)	36 mV dec^−1^ (14)		1263
N-Doped anodised AISI 304 steel mesh	146 (10; 14)	60.1 mV dec^−1^ (14)		1264
Fe_3_C modified AISI 304	290 (10; 14)	38 mV dec^−1^ (14)	98 (14)	1267
Chem.- + electrochem oxidation	550 (200; 14)	90. mV dec^−1^ (14)		1271
AISI 304	484 (100; 14)			
NiFe LDH (pristine) on NF	204 (10; 14)	78.39 mV dec^−1^ (14)		113
Aged–NiFe LDH on NF	59 (10; 14)	62.30 mV dec^−1^ (14)		113

A detailed discussion of possible reaction pathways through which OER occurs when in particular non-PGM-based electrocatalysts are involved would go beyond the scope of this work and we refer instead to the common articles that have been published on the subject.^133,452–456,880^

A significant technological advance in the development of oxygen evolving electrodes came with H. Beer's 1965 patent on the dimensionally stable anode (DSA), which usually consists of an active metal oxide such as RuO_2_, thermally decomposed on an inert carrier such as Ti. These electrodes are highly active in supporting electrocatalytic oxidation reactions and are also resistant to chemical and electrochemical degradation.^457^

### 7.1 Metal dioxides as OER and HER electrode materials

PbO_2_, MnO_2_, MoO_2_, TiO_2_ (with restrictions) and SnO_2_ were investigated as potential water-splitting electrocatalysts. To somehow keep the amount of literature within manageable limits, we will concentrate on MeO_2_ material in this subsection and hardly consider composite materials containing MeO_2_^458^ species.

#### 7.1.1 PbO_2_ as electrode material for oxygen evolution

The technical application of lead dioxide is not restricted to lead acid batteries. PbO_2_ is inexpensive and combines high conductivity and high corrosion resistance in acids. It is therefore broadly used as an active coating material for applications with an electrochemical background.^459^ PbO_2_ exists in two polymorphic structures, α-PbO_2_ and β-PbO_2_, exhibiting conductivities between 10^3^ S cm^−1^ and 10^4^ S cm^−1^.^460^

The usual method of forming a lead dioxide containing electrode is to oxidise lead first to PbSO_4_ and then to PbO_2_. Oxygen evolution in the lead acid battery occurs as a side reaction on the anode side during charging and as a partial anodic reaction during self-discharge of the positive plate. This is certainly the main reason why the OER properties of PbO_2_ have been intensively studied for decades.^461–465^ In one of the first publications dealing with OER on PbO_2_-based electrodes in sulphuric acid, the OER overpotential to current density relationship was investigated.^461^ A more detailed investigation of the composition and crystallographic phase of the products formed upon electrochemical oxidation of lead metal in 3.5 M H_2_SO_4_ was for the first time reported in 1978 by Pavlov and Rogachev.^463^ Lead oxidation takes place at the lead|oxide interface yielding tetragonal PbO and then either α-PbO_2_ or β-PbO_2_, while the evolution of oxygen (and as we know today not limited to lead metal) takes place at the oxide|solution interface.^463,466,467^ Oxygen (O_2_) evolution on PbO_2_ surfaces follows Tafel behaviour up to current densities of *j* = 50 mA cm^−2^ in sulphuric acid with slope values between 90 and 140 mV dec^−1^,^463–465^ whereas at higher current densities ozone evolves with a Tafel dependence.^464,468–471^ First works that report on a reduction of the OER overpotentials determined for PbO_2_ in H_2_SO_4_ were carried out in the late 1980s:^466^ Sb doped PbO_2_ significantly reduces the oxygen overvoltage.^466^ Investigations related to PbO_2_ concern *e.g.* detailed kinetic experiments,^472,473^ elucidation of the electrochemical reactions occurring whilst OER or ozone evolution reaction,^474^ the influence of ion doping on the ratio of O_3_/O_2_ produced,^469–471,475^ its suitability for wastewater treatment^476,477^ or the properties as an electrode in the lead-acid battery.^478,479^ With respect to lead-acid batteries, oxygen evolution is a parasitic reaction that needs to be suppressed rather than accelerated and reducing the OER overpotential is counterproductive.^480^ Therefore, much effort has been made to increase the overpotential of oxygen evolution of PbO_2_, which acts as an anode in sulphuric acid.^481,482^

In addition, experiments on PbO_2_-based electrode materials aimed rather on lowering the onset potential for ozone formation than for dioxygen formation.^475^ Deviations from the stoichiometry 1 : 2 in the Pb–O system significantly influence its conductivity and thus also its OER activity. The first publication, which reports specifically on overpotential values at a defined current density for the electrocatalytically initiated OER on PbO_2_, appeared in 2000.^483^ Anodes comprise PbO_2_ doped with Co exhibit an overpotential *η* of 535 mV for the OER in 1 M NaOH at a current density of *j* = 100 mA cm^−2^; the tafel slope amounted to 59 mV dec^−1^.^483^ Unfortunately, the study lacks long term polarisation experiments. An enhancement of the OER activity of PbO_2_ electrodes upon Co doping was later on confirmed by Velichenko *et al.*^475^ To the best of the authors' knowledge, the first studies specifically aimed at lowering the OER potential of PbO_2_-based electrode materials in order to make them a more active oxygen evolving electrode for water electrolysis was not published until 2007.^458,484,486,487^

Abaci *et al.* and Cao *et al.* found that the OER activity of PbO_2_ is enhanced considerably for sub-stoichiometric oxides.^485,486^ Besides doping with cobalt, doping with Ce or P enables to obtain PbO_2_ electrodes with increased OER activity.^484,487^ Phosphorous-doped PbO_2_ synthesised by co-deposition was investigated by Li *et al.* The OER activity (*η* = 615 mV at *j* = 0.174 mA cm^−2^) is improved when compared to pure PbO_2_ generated *via* a similar approach but, remains substantially lower compared with PbO_2_ classically-generated *via* Pb metal electrooxidation.^473^

A composite material based on titanium used as conductive substrate modified with TiO_2_ NT/PbO_2_ exhibited a reasonable low overpotential (*η* = 630 mV at *j* = 50 mA cm^−2^ in 1.53 M H_2_SO_4_).^488^

**Fig. 21:**
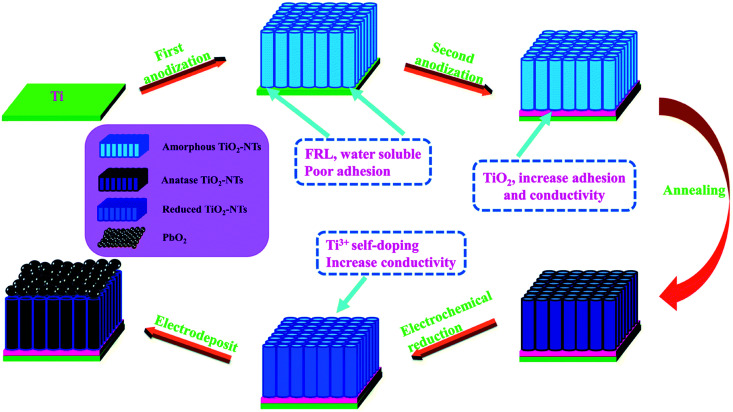
Schematic representation of the generation of Ti/TiO_2_-NTs/PbO_2_. Reproduced with permission from ref. 488. Copyright Royal Society of Chemistry 2021.

Since TiO_2_-NT was created by simple anodisation, the overall process seems straightforward (Fig. 21).

Thus, all in all, one can say that the overpotentials required to result in reasonable OER-based current densities for lead-based electrode materials are in the range of a few hundred millivolts, which is not on benchmark level.

#### 7.1.2 MnO_2_ as electrode material for oxygen evolution

Efforts to investigate manganese oxides as potential water oxidation electrocatalysts have most likely spurred by the presence of manganese in water oxidation cluster in nature's photosystem II. The Pourbaix diagram of Mn reveals that it is stable in the form of MnO_2_ at broad pH range (0–14) between 1.3 to 1.7 V *vs.* RHE.^489^ There are dozens of MnO_2_ polymorphs that crystallise in different crystal structures. The most important ones in terms of electrochemical applications are orthorhombic (cryptomelane), tetragonal β-MnO_2_ (pyrolusite) and layered δ-MnO_2_ (birnesite). The activity of MnO_2_ compounds is highly influenced by the presence of defects and such compounds have been under intense focus for their alkaline ORR properties for two decades (see *e.g.*, the carbon-supported and divalent metal-doped MnO_*x*_/C nanostructures Chatenet *et al.* prepared by a mild hydrothermal procedure^490–493^).

Alpha MnO_2_ is regarded as one of the most promising bifunctional catalysts for use in secondary metal–air batteries due to the advantageous OER^494,495^ and ORR^496,497^ activity. The unique feature of α-MnO_2_ is the large cavities (2V2 tunnel) surrounded by edge and corner-linked MnO_6_ octahedra. The first studies that are dealing with MnO_2_ anodes for application in aqueous solutions were carried out by Kokhanov and Shembel *et al.*^498,499^

Studies that particularly focused on OER on MnO_2_-coated electrodes were carried out in Japan starting in the middle of the 1970s.^500–502^ In their first contribution, Morita *et al.* report on MnO_2_ electrodes evaluated as oxygen-evolving electrode in 0.5 M H_2_SO_4_ and 1 M KOH.^500^ An active zone comprising a mixture of β-MnO_2_ and α-MnO_2_ was achieved on Pt or Ti substrate *via* thermal decomposition of Mn(NO_3_)_2_. The Pt–MnO_2_ samples turned out to be more active than the Ti-MnO_2_ ones (*η* = 650 mV, *j* = 1 mA cm^−2^, 0.5 M H_2_SO_4_; *η* = 480 mV, *j* = 1 mA cm^−2^, 1 M KOH). Manganese oxide-coated electrodes or electrodes which are coated with mixed oxides containing manganese oxide have found particular interest for seawater electrolysis, since it is known that they somewhat suppress the formation of chlorine.^503,504,1853^

**Fig. 22:**
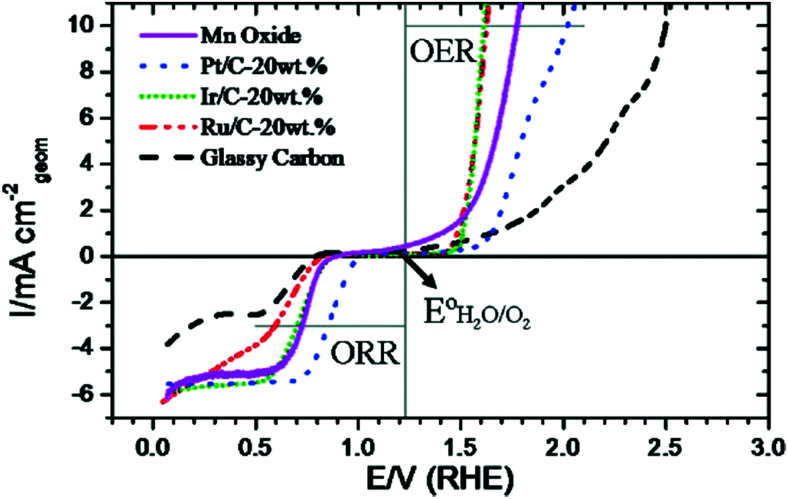
OER activities of the Mn-oxide electrode compared with the ones from Pt, Ir and Ru. Reproduced with permission from ref. 505 Copyright American Chemical Society 2010.

An active bifunctional electrocatalyst for ORR and OER comprising Mn oxide electrodeposited on glassy carbon was introduced by Gorlin *et al.*:^505^ its OER activity (*η* = 520 mV at *j* = 10 mA cm^−2^) in 0.1 M KOH is almost on par with that of Ir or Ru (Fig. 22). XPS results did neither 100% confirm the existence of α-Mn_2_O_3_ nor did they unequivocally rule-out the existence of MnO_2_, though it must be stated that the extreme surface and core of the crystallites may consist of different oxides, especially upon OER.

Fekete *et al.*^506^ deposited upstream-prepared manganese oxide catalysts using screen-printing, a widely used technique for *e.g.*, circuit boards. The screen-printed films although consisting mostly of the less active pyrolusite phase (β-MnO_2_) exhibited promising overall OER efficiency (*η* = 500 mV at *j* = 10 mA cm^−2^ in 1.0 M NaOH), suggesting that even materials traditionally considered less active can be activated by the choice of an appropriate deposition method and/or by an appropriate electrochemical activation. In that regard, the work of Moureaux *et al.* (for the ORR) demonstrated that the activity of nanostructured MnO_*x*_/C compounds can be significantly modulated by the nature of the counter cation of the hydroxide-based electrolyte;^493^ this likely also proceeds for the OER.

α-MnO_2_ exhibits better OER + ORR properties than β-MnO_2_ does.^495^ Selvakumar *et al.*, by hydrothermal procedures, synthesised nano-scaled (wires, tubes, particles) phase-pure α-MnO_2_.;^495^ their OER activity based on CV scans is mediocre (*η* = 570 mV at *j* = 1 mA cm^−2^ in 0.1 M KOH) which might be caused by insufficient catalyst loading (0.14 mg cm^−2^).

MnO_2_, although in general electrochemically stable, dissolves at high OER overpotentials in acidic media and at low potential values (Mn^III^ is non-negligibly soluble, including in base). Frydendal stabilised MnO_2_ upon modification with TiO_2_ or GeO_2_.^507^ By DFT, the authors demonstrated that the termination of undercoordinated sites on MnO_2_ is favourable for guest oxides with lower surface formation energies than MnO_2_. The calculations exhibit that GeO_2_ and TiO_2_ should indeed improve the stability of MnO_2_.

**Fig. 23:**
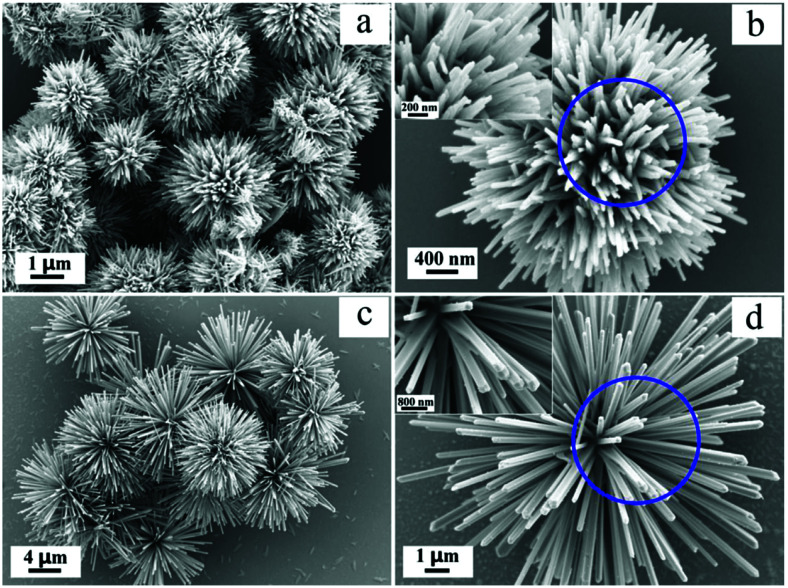
SEM images of dandelion-like- (a and b) and urchin-like α-MnO_2_ (c and d). Reproduced with permission from ref. 509. Copyright Elsevier 2017.

As mentioned, β-MnO_2_ shows lower OER activity owing to inaccessibility to the inner Mn centers, in sharp contrast to alpha MnO_2_. This disadvantage can be overcome *via* a specific synthesis strategy allowing to achieve highly porous β-MnO_2_ nanoplates with surface-bound catalytic Mn sites^508^ (*η* = 450 mV at *j* = 10 mA cm^−2^ in 1.0 M KOH). Zheng *et al.* investigated the influence of the morphology on the electrocatalytic activity of α-MnO_2_.^509^ Two different types of 3D radial a-MnO_2_ (dandelion- and urchin-like) have been synthesised through a hydrothermal route starting from MnSO_4_ upon exploitation of two different oxidants (K_2_S_2_O_8_ → dandelion-like; KClO_3_ → urchin-like)^509^ (Fig. 23).

**Fig. 24:**
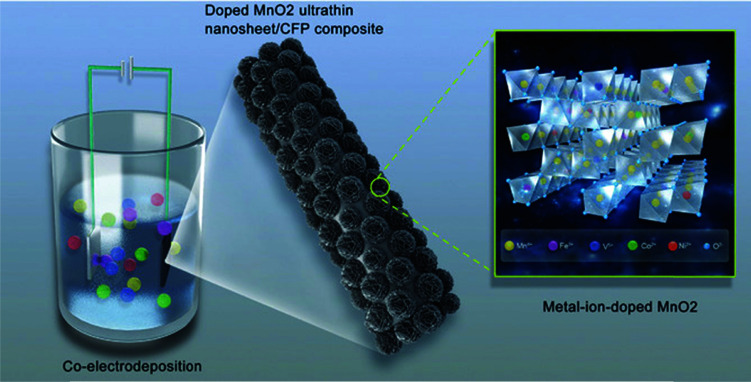
Schematic representation for the preparation of the metal-ion (Fe, V, Co, Ni)-doped MnO_2_ ultrathin nanosheet/CFP composite. Reproduced with permission from ref. 510, Copyright Wiley 2017.

The MnO_2_ based OER electrocatalysts listed so far required an overpotential in the 500 mV range to promote anodic water electrolysis with a current density of 10 mA cm^−2^ in 1 M alkaline. Ye *et al.* studied transition metal-ion doped MnO_2_ ultrathin nanosheets electrodeposited on carbon fiber in 2017.^510^ Transition metal ion doping into MnO_2_ is capable to increase the conductivity of the MnO_*x*_ structures^511^ but also to stabilise the Mn^III^/Mn^IV^ redox shuttle, at least for the ORR.^490,512^ Anodic co-deposition was exploited to prepare a composite electrode comprising multi (Fe, V, Co, Ni) doped MnO_2_ nanosheet/carbon fiber paper (Fig. 24): *η* = 500 mV measured from galvostatic measurements in 1.0 M KOH electrolyte at *j* = 20 mA cm^−2^.

**Fig. 25:**
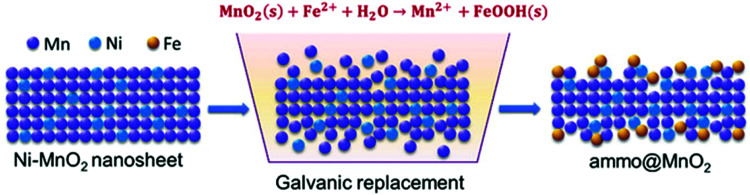
Process of the surface-guided formation of ammo@MnO_2_*via* the galvanic replacement reaction. Reproduced with permission from ref. 514 Copyright Wiley 2017.

Tripkovic *et al.* carried out an in-depth evaluation of (single) doped α-MnO_2_ in terms of structural stability, catalytic activity and electronic conductivity using DFT calculations.^513^ To the author's knowledge, the best OER performance determined for MnO_2_-based electrocatalysts was recently presented by Fang *et al.*^514^ Ni doped δ-MnO_2_ nanosheet array hydrothermally grown on Ni foam (Ni–MnO_2_/NF) and modified with amorphous mixed-metal (oxy)hydroxide overlayers exhibited a large (active) surface area and high electron conductivity. Short-time treatment of Ni–MnO_2_/NF with aqueous FeSO_4_ solution led to the deposition of the mixed metal(oxy)hydroxide layers *via* galvanic replacement leading to ammo@MnO_2_ (Fig. 25). The modified OER catalyst reached *j* = 10 mA cm^−2^ at only *η* = 232 mV overpotential in 1 M KOH. However, it is uncertain whether these properties can be maintained in the long-term.

Recent theoretical studies provide detailed insights into the requirements that must be met for high OER efficiency of MnO_2_-based catalysts. The increased OER activity of MnO_2_ polymorphic OER catalysts is known to be caused by the presence of Mn^3+^ ions whereby the suppression of the Mn^3+^ oxidation to Mn^4+^ by structural constraints was suggested as an important step to enable the accumulation of oxygen holes and the reductive elimination of O_2_.^515^*In situ* electrochemical and X-ray absorption spectroscopic studies revealed that that (i) Mn^3+^ is kinetically stabilised in tetrahedral centers and (ii) its presence strains the oxide lattice, which leads to a favourable arrangement of oxide-based *versus* metal-based energy levels, favoring improved OER.^516^ In general, the overall OER activity (OER-based current per projected area) of MnO_2_-based material depends on many individual factors like crystal structure,^517^ Mn^517,518^ oxidation state, lattice strain,^516^ the existence of structural fragments (μ-oxo-bridges,^815,819^ pseudo-cubane fragments^519^), coordinatively-unsaturated metal cations,^520^ oxygen vacancies,^521^ specific surface area,^522,523^ crystallinity or the electric conductivity of the oxide phase, explaining the richness of the literature about electrochemical properties of MnO_2_ compounds.

In a sense brought into conversation by nature itself MnO_2_ is still a promising water splitting electrocatalyst even if it is not one of the current favorites due to the limited performance and durability.

#### 7.1.3 MoO_2_ as electrode material for oxygen and hydrogen evolution

**Fig. 26:**
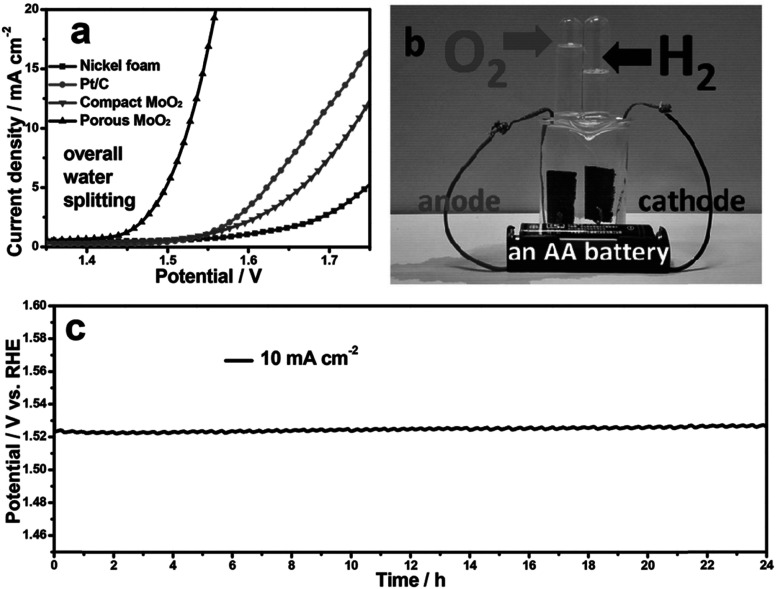
Steady-state polarisation curves for overall water splitting of Ni foam, commercial Pt/C, compact MoO_2_, and porous MoO_2_ in a two-electrode configuration. (b) Demonstration of water-splitting device. (c) Chronopotentiometric curve of water electrolysis for porous MoO_2_. Reproduced with permission from ref. 529 Copyright Wiley 2016.

Molybdenum dioxide exhibits metal-like electrical conductivity and has received considerable attention for exploitation as heterogeneous catalyst^524^ and for water electrolysis,^525^ both for OER^526^ and HER^527,528^ purposes. Pure, binder-free, porous MoO_2_ synthesised on nickel foam was checked for its full water splitting capabilities by Jin *et al.*^529^*Via* a hydrothermal process starting from ammonium molybdate solution, nickel foam activated into a full-water splitting electrocatalyst (1.52 V cell voltage; *j* = 10 mA cm^−2^ in 1.0 M KOH) (Fig. 26).

Oxygen vacancies created in MoO_2_ by post-treatment using N_2_H_4_ solution resulted in good HER (*η* = 200 mV; *j* = 10 mA cm^−2^) and OER (*η* = 371 mV; *j* = 85 mA cm^−2^) properties in 1.0 M KOH.^530^

**Fig. 27:**
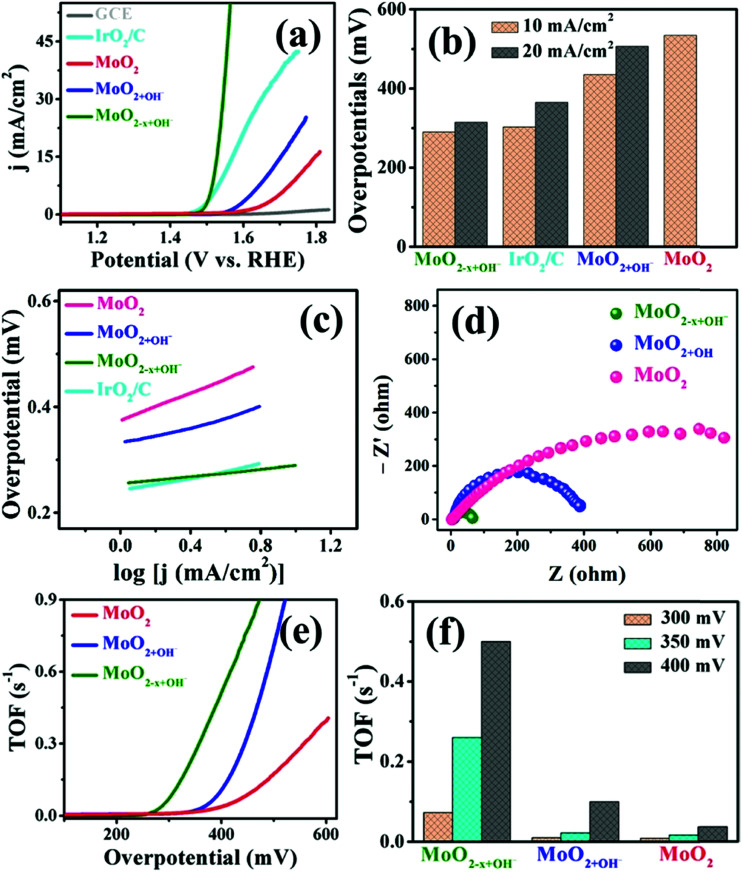
Polarisation curves toward the OER for as-grown MoO_2+OH^−^_ (7 h case), MoO_2–*x*+OH^−^_ (9 h case), commercially available MoO_2_, and IrO_2_/C electrocatalysts on GCE in 1 M KOH electrolyte. Reproduced with permission from ref. 531. Copyright American Chemical Society 2020.

Guha *et al.* synthesised MoO_2_*via* reduction of MoO_3_ upon annealing in hydrogen atmosphere.^531^ The post-grown MoO_2_ has OH^−^ occupancy after 7 h annealing in hydrogen (MoO_2+OH^−^_) and after 9 h of heat-treatment oxygen vacancies have been created (MoO_2−*x*+OH^−^_). With these defects, MoO_2_ was durable and active (*h* = 300 mV; *j* = 20 mA cm^−2^; 1.0 M KOH) for OER (Fig. 27).

#### 7.1.4 TiO_2_ as electrode material for oxygen evolution

**Fig. 28:**
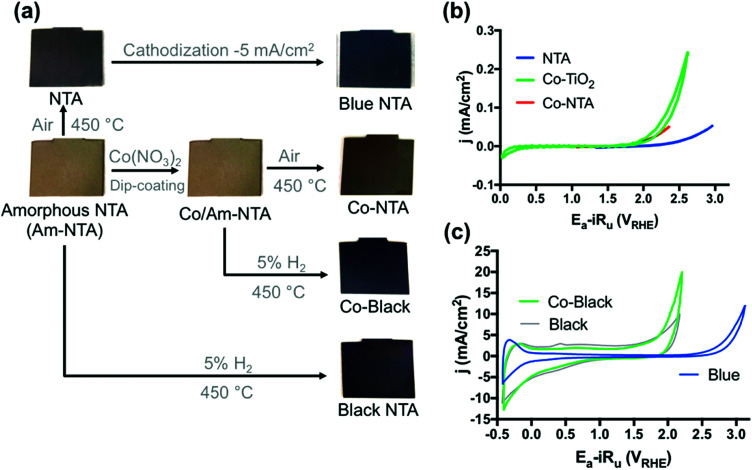
NTA electrode preparation procedures. (b and c) Cyclic voltammograms of NTA electrodes in 100 mM KPi buffer at pH 7.2. Reproduced with permission from ref. 539. Copyright American Chemical Society 2018.

TiO_2_ has long been recognised as a promising photocatalyst for water splitting and wastewater treatment.^532,533^ However, the low electron conductivity of pure TiO_2_ prevents its direct use for electrocatalytic water splitting.^534^ Anatase-structured TiO_2_ can be reduced at high temperatures *via* hydrogen to sub stoichiometric TiO_2−*x*_ which exhibit larger electronic conductivity (the best being reached by magneli phases, Ti_*n*_O_2*n*−1_:Ti_4_O_7_ and Ti_5_O_9_) and showed electrocatalytic water oxidation capabilities.^535,536^ In the meantime, conductive stoichiometric TiO_2_ nanotubes that are either blue or black in appearance, have been produced.^537^ The electronic conductivity and OER activity achieved with pure TiO_2_ without addition of suitable dopants remains insatisfactory for implementation in water electrolysis. TiO_2_ doping is a reasonable strategy to increase its conductivity:^538^ doping trace amounts of cobalt onto black TiO_2_ nanotube array resulted in a substantially lower OER overpotential and increased electrode stability.^539^ The best sample (Co-Black) showed at least a reasonable OER activity in a 0.1 M KPi buffer at pH 7.2: *j* = 10 mA cm^−2^ was measured at *η* = 770 mV^539^ (Fig. 28). TiO_2_ based materials are especially investigated as inert and conductive supports for OER catalysts, as detailed in other sections of the review and illustrated in ref. 248 and 540–542.

#### 7.1.5 SnO_2_ as electrode material for oxygen evolution

Transparent and conductive tin oxide (TCO) thin films are interesting for solar energy conversion, sensors and other various electrode applications.^543^ SnO_2_ is insulating in its bulk form, but, due to deviations in stoichiometry, it becomes semiconducting when manufactured in thin layers. Not only thin films of SnO_2_ but also SnO_2_ nanoparticles exhibit in comparison with their bulk counterparts advantageous electrical-, catalytic- and optical properties.^544–549^ An increase in conductivity can also be reached by increasing the number of free charge carriers realised through doping.^34,550^ The best-known material produced in this way are antimony-doped tin oxide (ATO) and fluorine-doped tin oxide (FTO); both received tremendous attention for their use as conductive and X-ray transparent carriers for electrochemically- or photochemically active compounds.^550–554^

The OER onset potential on pure SnO_2_ electrodes in aqueous solutions is shifted positive compared to PbO_2_-based electrodes^480^ explaining why (pure) SnO_2_ attracted significantly less attention for water electrolysis.

There are however several contributions that report on SnO_2_-based composite materials or doped –SnO_2_ for application in water electrolysis.^550,555–559^ For instance, Sreekanth *et al.*^559^ recently described the synthesis and investigation of SnO_2_ quantum dots decorated on spinel cobalt ferrite nanoparticles to give SnO_2_ QDs@CoFe_2_O_4_ NPs nanocomposites. In combination with Ni foam, this composite gave an average-active water electrolysis anode for alkaline OER (*η* = 290 mV; *j* = 10 mA cm^−2^; 1.0 M KOH).

However, to the best of the authors' knowledge, water electrolysis with reasonably satisfactory efficiency, which is promoted by a solid (pure) SnO_2_ electrode or by undoped SnO_2_ as active species which is only adapted to a conductive carrier, has not yet been described.

### 7.2 Perovskite-based electrode materials for oxygen and hydrogen evolution

Perovskite is a relatively common mineral from the mineral class of “oxides and hydroxides” with the chemical composition CaTiO_3_. Due to flexibility in composition (type of metal ions) and electronic structure, the properties of perovskite materials (commonly referred to as ABO_3_) cover a wide range, explaining their use in various fields.

**Fig. 29:**
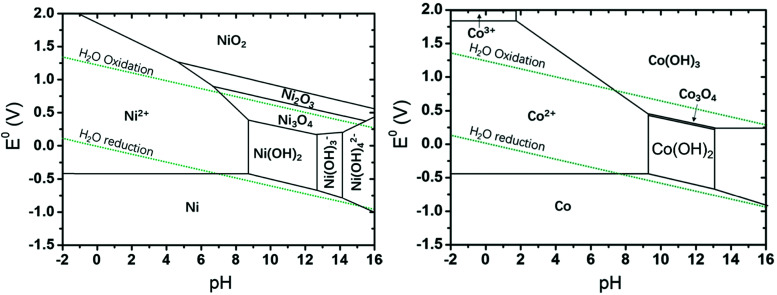
Pourbaix diagrams (potential-pH) calculated for the nickel/water and cobalt/water system. Reproduced with permission from ref. 577. Copyright Wiley 2018.

There are already many brilliant reviews that deal exclusively or partially with perovskite oxide-based materials as water-splitting catalysts. Some of these published articles deal with perovskite oxides for photocatalysis purposes^560–571^ but some of them provide an overview of the knowledge of water electrolysis on perovskite-based materials.^24,86,451,572–589^ Numerous advanced theoretical and experimental studies have been published, leading to a variety of perovskite-type oxides as potential ORR,^590^ OER^591–597,611^ and HER^598–600,619^ electrocatalysts. Because of the severe oxidative conditions experienced at OER anodes, and the highly-reductive conditions at the HER cathode, metal oxides have traditionally been found more adapted for the OER. This is also reflected in the position of the metal oxides within the Pourbaix diagram (Fig. 29): the number of contributions to perovskite-based OER catalysts far exceeds the number that can be ascribed to HER electrocatalysis. Due to the drastically-increased research output of transition metal-based materials for energy applications, we are unable to give a detailed overview of all known structure-activity relationships or all existing articles that mention perovskite-mediated OER or HER electrocatalysis. We shall therefore limit ourselves to investigations which cover the mile stones and the last stage reached.

The first work on perovskite-based OER catalyst dates back to the late 1970s.^601^ The electrocatalytic properties of oxides of 3d TM have been intensively investigated,^602,1309^ and it is known that their OER activity depends on the so-called electronic structure.^603,604^

**Fig. 30:**
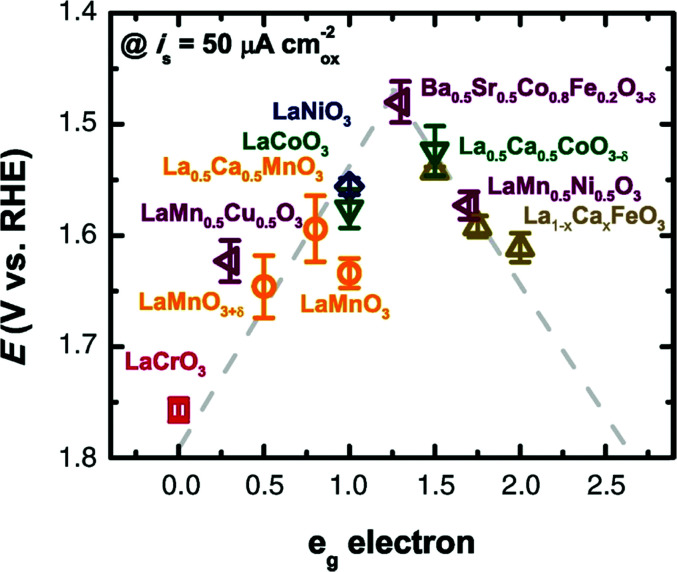
Relation between observed OER activities of perovskites (ABO_3_) and the number of e_g_ symmetry electrons of the transition metal (B in ABO_3_). Reproduced with permission from ref. 611. Copyright AAAS 2011.

Various factors of the electronic structure have been used as descriptors for the OER efficiency, including features (energy, filling and width) of the electronic states,^605^ the M-O coordination state,^606,607^ covalent part of the TM–O bond,^608^ and the number of electrons with specific symmetry.^609^ Such descriptors enable predicting their efficiency. Due to the structural differences of metal oxides, most of the descriptors based on electronic structure are limited to certain specific structural groups. There exist for instance a reliable relation between observed OER activities of perovskites (denoted as ABO_3_) and the number of *e.g.*, symmetry electrons^610,665^ of the transition metal (B in ABO_3_)^611^ as can be derived from the corresponding volcano plot (Fig. 30).

On both sides of the volcano according to perovskites with too little/too much *e.g.*, orbital occupancy, the too strong/weak interaction with oxygen species is responsible for a lower OER activity. At the top of the volcano, perovskites with e_g_ filling close to unity plot exhibit appropriate binding with reaction intermediates and high OER performance.

Moreover, it was shown that the perovskite family with its chemical tunability of various substituting metals can exhibit excellent catalytic performance.^611^

**Fig. 31:**
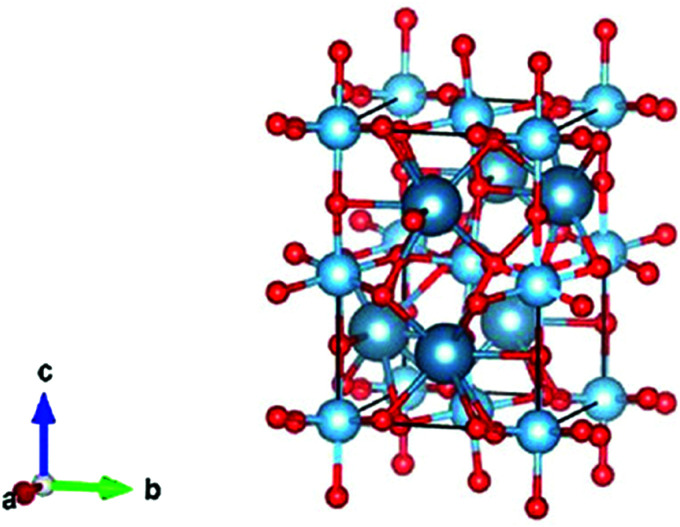
Schematic structure of CaTiO_3_ perovskite. Reproduced with permission from ref. 580. Copyright Wiley 2019.

In perovskite oxides (ABO_3_; Fig. 31), the B site is occupied with smaller transition metal ions octahedral (corner shared) surrounded by oxygen (BO_6_ octaeders). The A position is suitable for larger ions (alkali metal or rare-earth) with 12-fold coordination. On the surface the exposed B sites have BO_5_ coordination with the vertical oxygen removed, *i.e.*, this geometry would bring the orbital splitting of e_g_ and t_2g_ states to distinct energy levels and this surface can be considered as the active site.^612^ Synchronised e_g_ electron filling can be disturbed by strong on-site coulomb repulsive interaction between neighbouring e_g_ orbitals which can be up to some extent controlled by introducing high-valence transition metal- or rare earth ions.^613^ Especially for double perovskite (AA′)B_2_O_6_ or A(BB′)O_6_ the e_g_ orbital filling and thus also the OER properties can be changed in a targeted manner by substitution at certain positions.^591,614^

Up to now a high number of reasonable- and highly active perovskite-based OER electrocatalysts have been developed.^613,615–619^

Typically, perovskites are accessible *via* conventional synthesis methods like for instance high temperature solid state reactions with stoichiometric amounts of solid starting materials synthesis, sol–gel process and high-pressure synthesis. Perovskites synthesised this way are usually characterised by large particle sizes, small surface area (typically below 4 m^2^ g^−1^) combined with good intrinsic OER activity.^620–625^ Suntivic and Shao-Horn^611^ described a strategy for rationally-designing perovskite-based materials for OER: Ba_0.5_Sr_0.5_Co_0.8_Fe_0.2_O_3−*δ*_ (BSCF) catalyses the OER with intrinsic activity that is at least an order of magnitude higher than that of the state-of-the-art iridium oxide catalyst in alkaline media.^611^ The intrinsic OER activity of the investigated binary-, ternary-, quaternary- and pentanary oxides strictly depends on the occupancy of the 3d electron with an e_g_ symmetry of surface transition metal cations in an oxide leading to a volcano-shaped (electronic) structure-activity relationship.^611^ In an update Shao-Horn *et al.* examined the performance of 14 descriptors of the metal-oxygen bond strength using statistical approaches;^626^ they divided these descriptors into five groups and identified electron occupancy and metal-oxygen covalency as the dominant influences on the OER activity. Durability and performance of perovskites upon OER electrocatalysis have been studied in detail: some of the perovskites are leached by either A or B metal cations and surface amorphisation subject to OER conditions.^627,1773,1781^

**Fig. 32:**
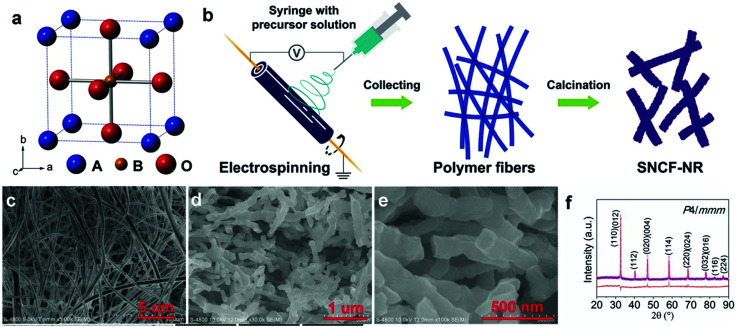
(a) ABO_3_ perovskite structure. (b) Schematic illustration of the preparation process of SNCF-NR by electrospinning. (c) Scanning electron microscopy (SEM) image of as-spun precursory polymer nanofibers before calcination. (d and e) Low/high-magnification SEM images of SNCF-NR. (f) Refined XRD pattern of SNCF-NR. Observed (purple circles), calculated (red solid line), and differences (orange line, bottom) are presented. Reproduced with permission from ref. 619. Copyright Wiley 2017.

A strategy that aims in improving the overall OER activity is based on increasing the specific surface area and the surface-to-volume ratio by reducing the particle size (down to nm dimension) without compromising the morphology (porosity).^628^ Nano-scaled perovskites have been accessible by fine adjustment of synthesis conditions of wet chemical routes (sol–gel processes,^629–632^ hydrothermal procedures.^628^ Nano-scaled perovskites have been accessible by fine adjustment of synthesis conditions of wet chemical routes (sol–gel processes,^629–632^ hydrothermal procedures^633–641^) deposition approaches (chemical precipitation^642–652^ physical-,^653–657^ or chemical vapor deposition,^658–660^ electrodeposition^617,661–663^), electrospinning,^664–667^ and template-based approaches.^668–679^ Nanorods comprising SNCF are also accessible upon a facile electrospinning method (Fig. 32) and showed very good bifunctionality (HER + OER) with respect to water electrolysis (1.68 V cell voltage; *j* = 10 mA cm^−2^; 0.1 M KOH).^619^

In addition to controlling the particle size of the synthesis product while the synthesis is actually being carried out, top-down approaches to generate small particles using mechanical grinding of bulk materials represent an alternative route to small particles.^618,680^

Notably; reducing size dimensions to nm scale does not simply increase the surface to volume ratio but can lead to novel physical properties and make nano-sized perovskite different from their bulk counterparts.^665,681^

**Fig. 33:**
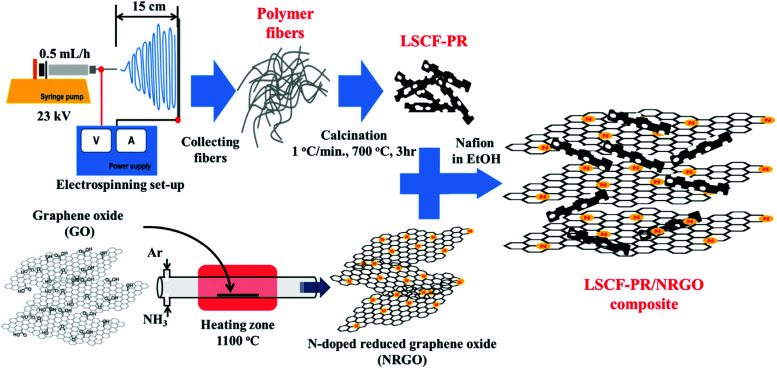
Schematic presentation of the preparation route leading to perovkite-grapheneoxid composite with special morphology. Reproduced with permission from ref. 667. Copyright Elsevier 2014.

Many composite materials developed as potential water-splitting electrocatalysts bear perovskite as the active electrocatalytic phase. Park *et al.* reported on the synthesis and properties of an electrospun graphene oxide-based composite, baring La_0.5_Sr_0.5_Co_0.8_Fe_0.2_O_3_ perovskite nanorods as a catalytically-active phase and exhibiting bifunctional properties for oxygen evolution (*η* = 570 mV at *j* = 15 mA cm^−2^) and oxygen reduction^667^ (Fig. 33).

Non-noble element-containing perovskites need not shy away from a comparison with highly established and highly-active PGM-containing water splitting electrocatalysts like *e.g.*, IrO_2_. Chen *et al.* synthesised nano-scaled oxygen-deficient BaTiO_3−*x*_ perovskites by sol–gel-based chemistry^630^ and obtained a reasonable active bifunctional (OER + ORR) electrocatalyst: at relatively low overpotentials (*η* < 370 mV), it proved more efficient than IrO_2_ for OER in 0.1 M NaOH.^630^

In recent years the OER performance of perovskite-based electrode materials was enormously improved.^596,682–684^ A heterostructured catalyst comprising La_0.5_Sr_0.5_CoO_3−*δ*_ (LSC) perovskite as the OER active part and K^+^ bonded molybdenum diselenide (K-MoSe_2_) as the active HER part was very recently shown by Oh *et al.*^684^

**Fig. 34:**
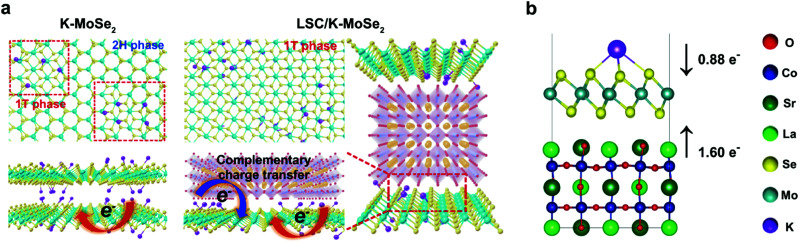
(a) Schematic of the atomic structure and charge transfer effect for K-MoSe_2_ and LSC/K-MoSe_2_. Complementary charge transfer in LSC/K-MoSe_2_ can modulate the electronic structure of MoSe_2_, increasing the 1T-MoSe_2_ ratio in the heterostructure. (b) Charge transfer from K and LSC to MoSe_2_ in the optimised LSC/K-MoSe_2_ heterostructure. Reproduced with permission from ref. 684. Copyright Nature Publishing 2021.

The LSC/K-MoSe_2_ system characterises the multidirectional charge transfer phenomenon, with a two-way charge transfer from K to MoSe_2_ and from LSC to MoSe_2_ (Fig. 34a and b), which is claimed to be responsible for the good (full-) water electrolysis performance (1.75 V cell voltage; *j* = 50 mA cm^−2^; 1 M KOH).

**Fig. 35:**
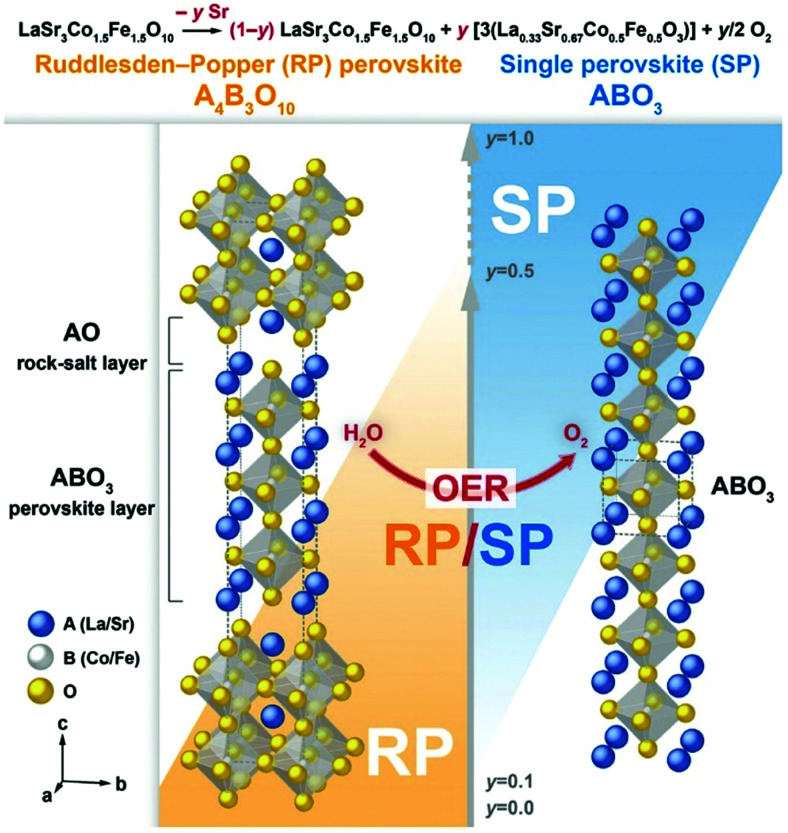
Design of RP/SP composites. Schematic for the RP/SP composites showing RP and SP phase crystal structures. The unit cell of the SP structure is duplicated along the c-axis, to suggest a difference in the material's dimensionality, that is, 2D for RP *versus* 3D for SP. Reproduced with permission from ref. 596. Copyright Wiley 2021.

Many recently-published papers report on composite materials that contain perovskite as an active part of the OER. For instance, dual-phase perovskite oxide composites comprising Ruddlesden-Popper (RP) perovskite and a La_0.33_Sr_0.67_Co_0.5_Fe_0.5_O_3_ single perovskite (SP), each of which self-assembled from perovskite precursors with strongly-interacting interfaces have been synthesised through a cation-deficiency strategy by Xu *et al.*^596^ (Fig. 35). The composite with optimised phase composition and structure exhibited competitive overall OER performance (*η* = 270 mV; *j* = 10 mA cm^−2^; 0.1 M KOH).

### 7.3 Spinel-based electrode materials for oxygen and hydrogen evolution

#### 7.3.1 Spinel-based electrode materials for oxygen evolution

Spinel (more precisely magnesia spinel) is a frequently-occurring mineral from the class of “oxides and hydroxides” with the idealised chemical composition MgAl_2_O_4_ (a magnesium aluminate from a chemical point-of-view). Spinel group minerals mostly share the composition AB_2_X_4_ (where A and B are metal ions), can be colourless, but depending on their composition, can also present very different colours: red, lavender, blue, green, brown, black or yellow. Originally, they were therefore coveted gemstones,^685,686^ like the Black Prince's Ruby and the “Timur ruby” in the British Crown Jewels.^687^

Their diverse compositions, electron configurations and valence states, yield a wide range of magnetic-,^688–690^ optical-^691,692–694^ electrical-^690,695–699^ and catalytic-^700,701–711^ properties.

Many review papers have already been published and deal exclusively^712,713,1318^ or in part^451,575,576,579,581,584,585,588,714–716^ with spinel materials for oxygen electrocatalysis.

After briefly working out some general characteristics, the present section will focus on illuminating the publications that can be considered pioneering work both for the development of design principles and for the successful application of these design principles to water oxidation, *i.e.*, work that deals with fascinatingly-active and stable OER electrocatalysts. In addition, very current, promising results will be highlighted.

**Fig. 36:**
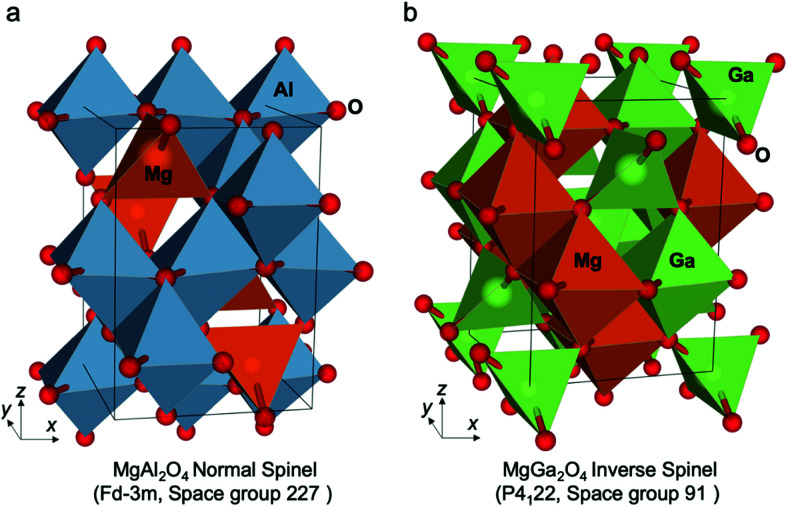
Normal and inverse spinel structures. (a) MgAl_2_O_4_ normal and (b) MgGa_2_O_4_ inverse ground state atomic configurations. In each case the unit cell is shown by solid black lines. Octahedral and tetrahedral atomic coordination environments are also identified by the coordination polyhedra in each case. Reproduced with permission from ref. 717. Copyright Nature Publishing 2020.

In an AB_2_X_4_ spinel metal A ions (in +2 or +4 oxidation state) occupy the centers of tetrahedral-coordinated positions, metal B ions (in +3 or +2 oxidation state) occupy the centers of octahedral coordinated positions, and the anion (*e.g.*, O^2−^) is located at the polyhedral vertexes (for normal spinels see Fig. 36a). The tetrahedral spaces are usually smaller than the octahedral ones. Cations with smaller radii preferentially occupy the A sites, while larger cations preferentially occupy the B sites.

One type of cation may occupy different positions, *i.e.*, tetrahedral and octahedral interstices. Depending on their distribution, spinels are therefore distinguished into three classes: normal, inverse (Fig. 36a and b)^717^ and complex spinels In normal spinels AB_2_X_4_, cations A solely occupy octahedral centers and B tetrahedral centers. This is valid for the “original type of spinel” MgAl_2_O_4_. For inverse spinels (B(AB)X_4_), half of the B cations occupy tetrahedral positions and the remaining half, the octahedral-coordinated centers: MgGa_2_O_4_ or NiFe_2_O_4_ are typical representatives’ inverse spinels (Fig. 36b). In complex spinels, both sort of metal ions partially occupies both the tetrahedral and octahedral interstices: CuAl_2_O_4_ is an example of complex spinel.

Defects are crucial to the spinel's properties, and in particular can significantly increase their catalytic activity.^709,718–721^

Like perovskites, spinels are an important class of widely available,^722^ thermodynamically stable,^723^ relatively cost- and environmental-friendly^724^ OER electrocatalysts with a well-known good efficiency.^709^ Spinels are accessible *via* a number of methods: high-temperature solid-phase synthesis starting from metals, metal oxides, -halides, hydroxides or other salts;^725^ spray pyrolysis;^706^ vapor phase methods at lower temperature;^726,727^ low-temperature methods are also feasible, like solution phase (sol–gel, hydrothermal- or solvothermal-) approaches^721,728–732^ or *wet-deposition*-based techniques like *e.g.* electrodeposition,^733^ electrospinning,^702,734,735^ or dip-coating.^736^

**Fig. 37:**
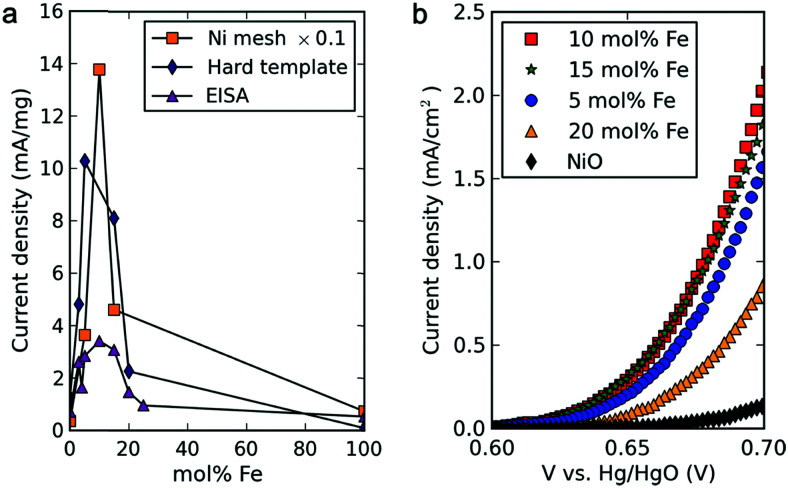
(a) Electrochemical oxygen evolution activity at a fixed overpotential of 360 mV for the varying synthesis methods and compositions of mixed metal oxide electrocatalysts. (b) Geometric area-normalised polarisation (scan rate = 1 mV s^−1^) data of mixed Ni–Fe oxide catalysts (synthesised by the EISA method) showing the highest activity for 10 mol % Fe oxide. Reproduced with permission from ref. 736 Copyright American Chemical Society 2012.

Landon *et al.*^736^ reported on the synthesis of spinel-phase based Fe–Ni Oxides with different Ni to Fe ratio by using three different synthesis strategies: evaporation-induced self-assembly, hard templating and dip-coating (sample names = EISA, hard template and Ni mesh, respectively). Regardless of the selected synthesis method, the Ni–Fe oxide catalysts comprising a mixed NiO/NiFe_2_O_4_ phase exhibited substantially higher activity than pure oxides, the activity peaking near 10 mol% Fe (Fig. 37a). Reasonable OER activities, although not competitive to recently-developed OER electrocatalysts were shown (*η* = 440 mV; *j* = 2 mA cm^−2^; 1 M KOH; Fig. 37b).

**Fig. 38:**
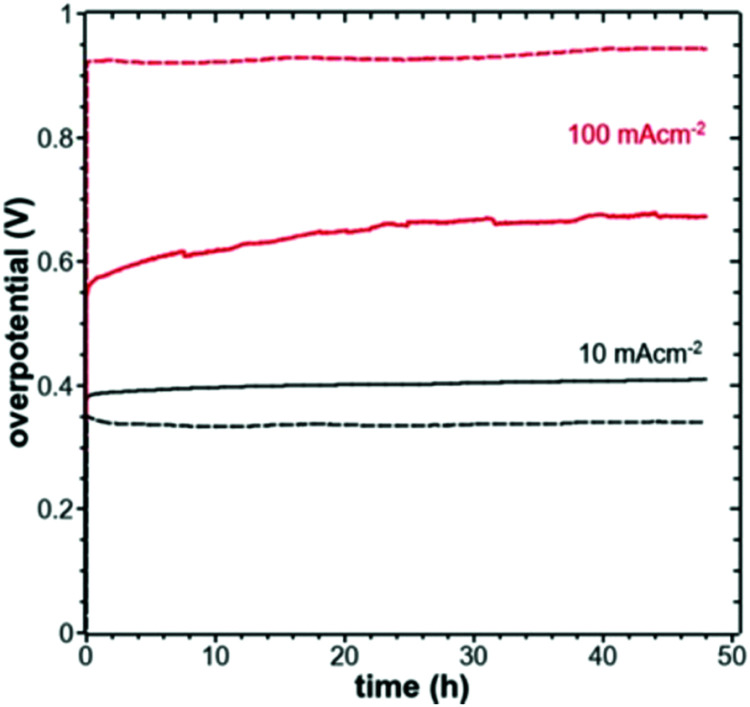
Plot of the overpotential as a function of time at current densities of 10 mA cm^−2^ (black) and 100 mA cm^−2^ (red) measured in 1 M KOH for films deposited at 50 °C (dashed lines) and 103 °C (full lines). Reproduced with permission from ref. 733. Copyright American Chemical Society 2020.

Crystalline and amorphous films of Co_3_O_4_ are accessible *via* a low-temperature route comprising electrodeposition, *e.g.*, on stainless steel. Koza *et al.*^733^ demonstrated convincing overall OER properties of such steel-supported spinel films (*η* = 400 mV at *j* = 10 mA cm^−2^; pH = 13; Fig. 38).

**Fig. 39:**
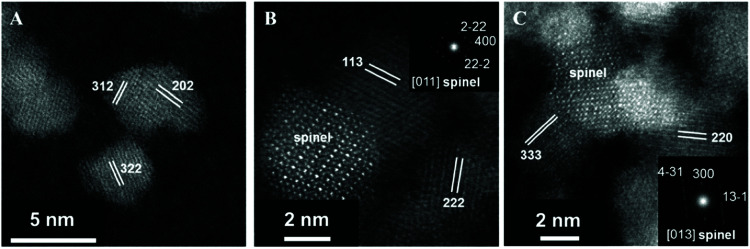
High-resolution images of spinel-type CoV_2–*x*_Fe_*x*_O_4_ (*x* = 0–2) nanoparticles: (A) CoFe_2_O_4_, (B) CoFeVO_4_, and (C) CoV_2_O_4_. Reproduced with permission from ref. 703. Copyright American Chemical Society 2018.

The high-temperature solid-state method is useful for large-scale applications, but requires long reaction times.^737^ In general, all synthesis strategies allow creating defects in spinel structures using specific settings for the respective synthesis method.^738–740^ All processes discussed so far are also suitable to produce spinel-based nanoparticles,^721,730,731,741^ whereas vapor-phase processes are particularly suited to synthesise 2d-structured materials.^726^ In view of their practical application in real water electrolysers, the vast majority of recently published papers in the field of spinel-based water electrocatalysis rely on nanocrystalline systems,^727^*e.g.* generated *via* sol–gel-based methods^742–746^ as demonstrated by Chakrapani *et al.* who synthesised uniform and highly dispersed CoV_2−*x*_Fe_*x*_O_4_ (*x* = 0–2) spinel nanoparticles^703^ (Fig. 39).

However, it was found that well-dispersed spinel-structured nanoparticles are also accessible by hydrothermal synthesis using additional surfactants such as ethylenediamine,^731^ polyvinylpyrrolidone and polyethylene glycol,^747^ cetyltrimethylammonium bromide or ethanol^748^ or upon solvothermal routes^749^ based on *e.g.* dimethylforamide (DMF),^749,750^ alcohols^102,751^ or polyethylenglycole.^752^ Although even highly faceted nanoparticles can be elaborated *via* template-free hydrothermal approaches^753^ the exploitation of hard-^672^ or soft templates^754^ still represents the method of choice, when regularly-shaped nanoparticles are desired.

**Fig. 40:**
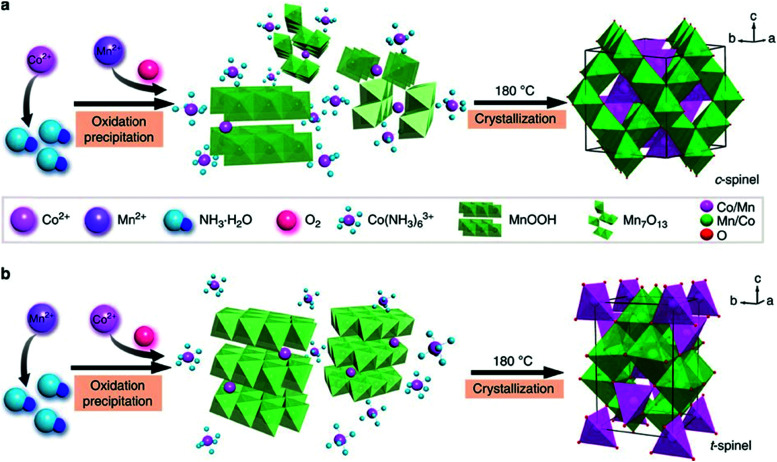
Schematic synthesis of cubic (a) and tetragonal (b) spinel phases, involving two steps of oxidation precipitation and crystallisation. Reproduced with permission from ref. 707. Copyright Nature Publishing 2015.

In addition, spinel-based nano-scaled materials are accessible by precipitation- based strategies^755–757^ or upon an oxidation-precipitation routes.^707^ The precipitation route might be expanded by templates: transition metal (*e.g.* Fe) hydroxides can be precipitated in alkaline solution; if Al^3+^ is simultaneously present in solution Al(OH)_3_ is precipitated as well, which in principle allows the generation of mesoporous spinel oxides *via* this hard template-based strategy.^758^ In general, porous structures are available *via* the usage of templates^754,759^ or are accessible by carbonate and oxalate-based precipitants, which will form CO_2_ upon thermal decomposition.^760,761^ Small (20 < particle size < 30 nm) but agglomerated cobalt manganese (CoMnO) spinels have been synthesised *via* a solution-oxidation-precipitation route.^707^ The tailored generation of cubic and tetragonal-phase material was achieved by simply reordering the addition of Co^2+^ and Mn^2+^-containing metal salts in the oxidation/precipitation step (Fig. 40). A hybrid material comprising carbon-supported CoMnO spinel particles synthesised this way exhibied reasonable alkaline OER activity (*η* = 500 mV; *j* = 10 mA cm^−2^, pH 13), though the use of carbon as a conductive additive presents issue in terms of long-term durability (it will irremediably corrode upon OER) and may bias the OER activity measurement as shown by Poux *et al.* for perovskite oxides during ORR-OER.^762^

**Fig. 41:**
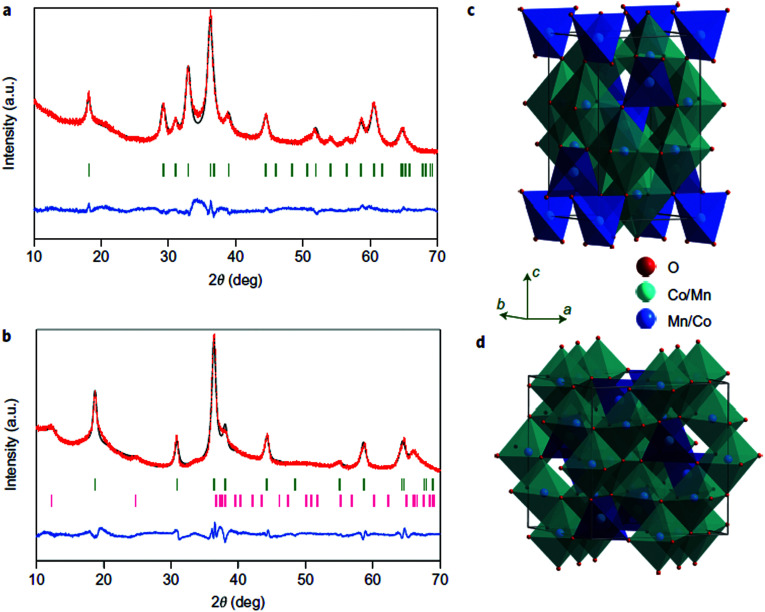
Structural analysis of the synthesised nanocrystalline spinels. (a and b) Rietveld- refined XRD patterns of CoMnO-B (a) and CoMnO-P (b) with experimental data (red dots), calculated profiles (black line), allowed Bragg diffraction positions (vertical bars) and difference curve (blue line). (c and d) Schematic representation of tetragonal (c) and cubic (d) spinels. Reproduced with permission from ref. 763. Copyright Nature Publishing 2015.

Cheng *et al.* introduced a particular synthesis route which takes advantage of oxidation and precipitation to generate a Mn-based spinel precursor, followed by reduction and renewed precipitation (reduction-recrystallisation route).^763^ Depending on the type of reducer (NaH_2_PO_4_ or NaBH_4_) cubic-phase- (CoMnO-P) or tetragonal-phase Co_*x*_Mn_3−*x*_O_4_ (CoMnO-B) was obtained (Fig. 41). The OER activity of electrodes prepared with the CoMnO-B-based spinels exhibited reasonable catalytic activity (*η* = 635 mV at *j* = 2.5 mA cm^−2^; pH = 13). DFT calculations provided insight into the capability of the material towards oxygen adsorption, a usual descriptor of ORR activity. OER being the reverse process of the ORR, one predicts that the tetragonal material should provide higher OER activity, in agreement with experimental finding.

**Fig. 42:**
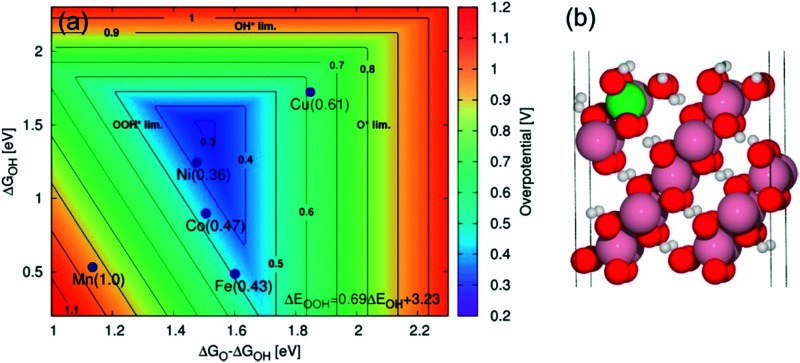
(a) 2D map of theoretical overpotentials *η* for the doped 101̄4 surface of β-CoOOH as function of Δ*G*_O_ − Δ*G*_OH_ and Δ*G*_OH_. The individual values of *η* are indicated in brackets. Improvement in activity relative to undoped surface is obtained in the case of Ni with *η* = 0.36 V and Fe with 0.43 V. Reproduced with permission from ref. 711. Copyright American Chemical Society 2013.

Bajdich *et al.* performed an in-depth evaluation of the activity of spinel-phase cobalt oxides which covers (i) the determination of the stability (under anodic electrode conditions) – and (ii) of the OER activity of selected surfaces in bulk material.^711^ The investigations resulted in a calculated Pourbaix diagram clearly unmasking β-CoOOH as the OER catalytic active phase for alkaline water electrolysis; its OER activity can be enhanced by surface substitution of Co by Ni (Fig. 42), as experimentally confirmed by the well-known highly-active Ni_*y*_Co_1−*y*_O_*x*_.

**Fig. 43:**
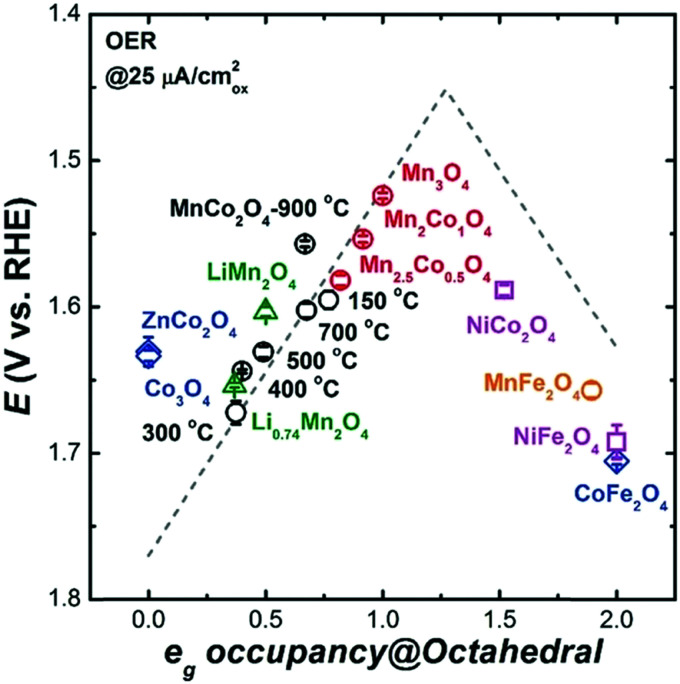
OER activity on various spinels as a function of e_g_ occupancy of the active element at octahedral site. Reproduced with permission from ref. 704. Copyright Wiley 2017.

As with perovskites,^611^ descriptors for oxygen electrocatalysis (ORR + OER) have also been developed for spinels.^704^ For MnCo_2_O_4_ species with different electronic structures, the Mn in octahedral centers is identified as the active site. Plotting the ORR/OER activity against the Mn valence state in octahedral site, results in a volcano curve, whose summit locates at the Mn valency of ∼+3. This finding was transferred to other transition-metal-spinels and the active cation e_g_ occupancy in octahedral sites was found the dominating descriptor for spinels ORR/OER activity as well (Fig. 43).

Several strategies to improve the electrocatalytic properties of spinel electrocatalysts have been considered, including fine-tuning the phase and composition (doping of well-known spinels with metal ions or the combination of spinels with other compounds in a hybrid material strategy)^764–771,1324^), the introduction of core–shell architectures or general crystal engineering on the nano- or micron-scale.^772–778^ In one of these exciting works, Bell *et al.*^771^ showed how metals like Au, Pt, Pd, Cu, Co can be used to enhance the OER activity of metal oxides (Co_3_O_4_ in this study). The electrochemical activity is influenced by the increase in the Co(iv) proportion following the increased oxidation of cobalt oxide by gold (Au has greater electronegativity than Pt or Pd).

**Fig. 44:**
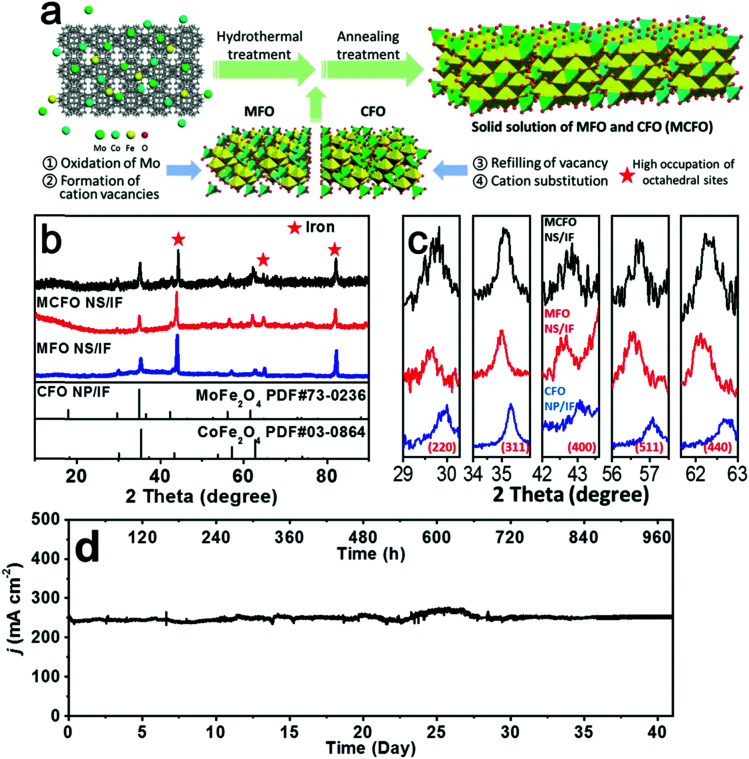
(a) Scheme of synthesis route for MCFO NS/IF. (b and c) XRD patterns of MCFO NS/IF, MFO NS/IF and CFO NP/IF. (d) The chronoamperometric plot of OER on MCFO NS/IF at 1.49 V *versus* RHE in 1.0 M KOH for 1000 h (25 °C). Reproduced with permission from ref. 784. Copyright Wiley 2021.

Latest efforts aimed in further incrementing the activity of spinel-based OER electrocatalysts.^779–785^ A nano-scaled oxide hybrid material comprising CoFe_2_O_4_ spinel modified by CeO_2_ (CeO_2_@CoFe_2_O_4_) displayed outstanding OER activity in 1 M KOH: a very low overpotential (*η* = 213 mV) was enough to reach *j* = 100 mA cm^−2^.^780^ A strategy to increase the number of octahedral OER active sites on the surface of spinel oxides was recently shown by Yue *et al.*:^784^ a solid solution comprising MoFe_2_O_4_ and CoFe_2_O_4_ nanosheets supported on iron foam was synthesised through a hydrothermal route + annealing step (Fig. 44a–c). Additional cation vacancies induced by oxidation of Mo led to cations filling into unoccupied octahedral interstices (cationic misalignment) and to high occupation of octahedral sites, hence to an increased number of OER active sites. The OER activity of the material is convincing, with *j* = 250 mA cm^−2^ at *E* = 1.49 V *vs.* RHE in 1 M KOH (Fig. 44d).

#### 7.3.2 Spinel-based electrode materials for hydrogen evolution

As Section 7.2 mentions, metal oxides are traditionally more resistant in OER than in HER condition. Pure binary spinel-based oxides (not specifically treated) either lack of sufficient activity or durability for HER electrocatalysis.^786,787^ It is therefore understandable that in early studies spinel-based oxides were clearly assigned the role of the oxygen-evolving electrode in water electrolysis experiments.^788^

10–20 years ago, spinel-based materials were investigated for their photocatalytic properties to promote hydrogen evolution.^789,790^ Thus, for years, the trend was to use complex spinels for HER electrodes.

Ternary Copper-cobalt-oxide spinels (Cu_*x*_Co_3−*x*_O_4_) were tested for OER and HER under close-to-real industrial water electrolysis conditions (*j* = 1 A cm^−2^; 1.0 M NaOH):^787^ (i) doping of Co_3_O_4_ with copper significantly increased the coating conductivity (maximum at *x* = 0.3), (ii) accelerated life test showed larger durability of electrodes with *x* = 0.3 (cathode life = *ca.* 518 h, *versus* 190 h for Co_3_O_4_).

Zhu *et al.* reported astonishing HER activity (*η* = 400 mV; *j* = 400 mA cm^−2^) for Co_3_O_4_ microtube arrays (Co_3_O_4_-MTA) that even outperformed the HER activity of Pt/C.^791^ However, never before and never again afterwards could Co_3_O_4_ be attested to such a high level of activity. One notices that the electrocatalytic HER testing was carried out with a Pt counter-electrode. Obviously, during the HER experiments (continuous cyclic voltammetry scanning for 2000 cycles in aggressive medium: 1 M KOH) Pt from the counter-electrode could have been transferred to the working electrode.^1269^ So, these experiments should be reproduced/verified, with the nature of the counter-electrode and cell geometry more compatible to best practices.^792,1744^

All spinel-based materials (M_3_O_4_) that proved efficient and stable HER electrodes are altered by doping,^789,793–799^ or are otherwise modified spinels *e.g.*, hybrid materials that contain pure spinel M_3_O_4_ (binary metal oxides)^800^ besides another *e.g.*, inorganic compound or contain or represent more complex spinels *e.g.* AB_2_X_4_.^801–806^

**Fig. 45:**
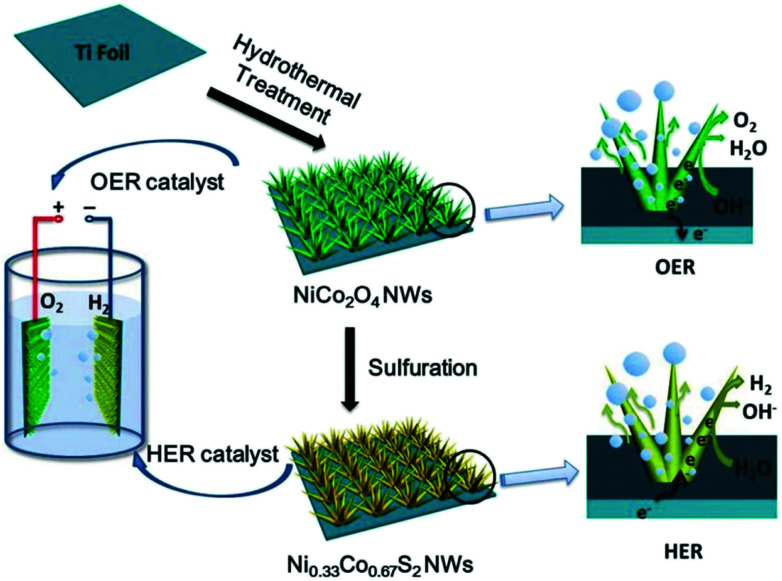
Schematic illustration of the synthesis of NiCo_2_O_4_ and Ni_0.33_Co_0.67_S_2_ nanowires, and the utilisation of these homologous Ni–Co based nanowires as OER and HER catalysts for water splitting. Reproduced with permission from ref. 796. Copyright Wiley 2015.

Peng *et al.* studied a spinel-based nanowire electrode system for full water electrolysis.^796^ NiCo_2_O_4_ nanowires, subjected to sulphuration to yield Ni_0.33_Co_0.67_S_2_ nanowires (Fig. 45), showed good alkaline HER performance (*η* = 100 mV, *j* = 10 mA cm^−2^, pH 14). However, upon sulphuration, NiCo_2_O_4_ loses its spinel structure and the pyrite structure can be assigned to Ni_0.33_Co_0.67_S_2_.

**Fig. 46:**
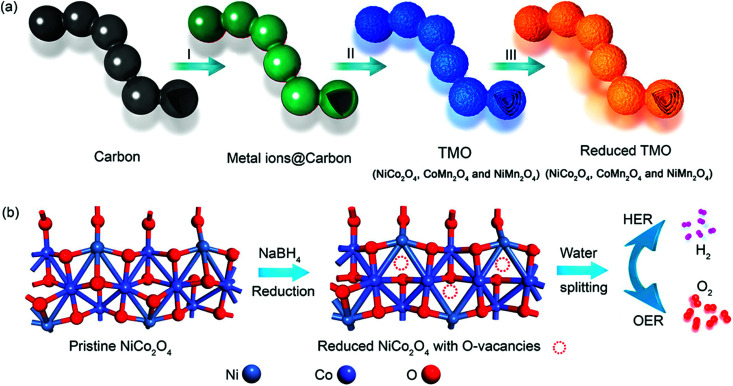
(a) Schematic illustration of the formation process of R-TMO with a necklace-like multishelled hollow structure for water splitting. (I) The absorption of metal ions on the carbon, (II) calcination of the absorbed carbon, and (III) reduction of the TMO to obtain R-TMO with a necklacelike multishelled hollow structure. (b) Schematic illustration of creating oxygen vacancy defects on the surface of NCO after reduction. Reproduced with permission from ref. 797. Copyright American Chemical Society 2018.

In 2018, complex spinel transition metal oxides (TMO's such as NiCo_2_O_4_, CoMn_2_O_4_ or NiMn_2_O_4_) with a multi–shell hollow structure (necklace-like) were introduced, which were reduced with NaBH_4_ in aqueous solution (Fig. 46).^797^ The reduction treatment contributed to their bifunctionality and resulted in reasonable OER alkaline activity (*η* = 250 mV; *j* = 10 mA cm^−2^) and good HER activity (*η* = 300 mV; *j* = 200 mA cm^−2^) combined with good durability in 1 M KOH.

Insertion of non-metals like S, P into transition metal-based spinels has become an established strategy to improve their HER electrocatalytic properties.^799,800,807–809^ Wang *et al.*^799^ also demonstrated that P-doping of Co_3_O_4_ spinel leads to improved OER activity (*η* = 260 mV; *j* = 20 mA cm^−2^) paired with good HER activity (*η* = 140 mV; *j* = 100 mA cm^−2^) in 1 M KOH.

Muthurasu *et al.* recently presented a hybrid material comprising Co_3_O_4_/MoS_2_ heterostructure capable to act as anode and cathode in alkaline water electrolysis.^800^ An OER current density of *j* = 20 mA cm^−2^ was obtained at *η* = 230 mV (HER: *η* = 205 mV, *j* = 10 mA cm^−2^) in 1 M KOH. Williamson *et al.* recently reported the synthesis of small thiospinel CoNi_2_S_4_ nanocrystals with an average size of 4.8–10.7 nm.^810^

Among spinel-structured NiCo_2_O_4_, NiCo_2_S_4_ and NiCo_2_Se_4_, NiCo_2_Se_4_ was found to demonstrate higher oxygen and hydrogen evolution reaction activities (245 mV and 122 mV for *j* = 10 mA cm^2^) respectively) compared to those of NiCo_2_O_4_ and NiCo_2_S_4_.^809^

**Fig. 47:**
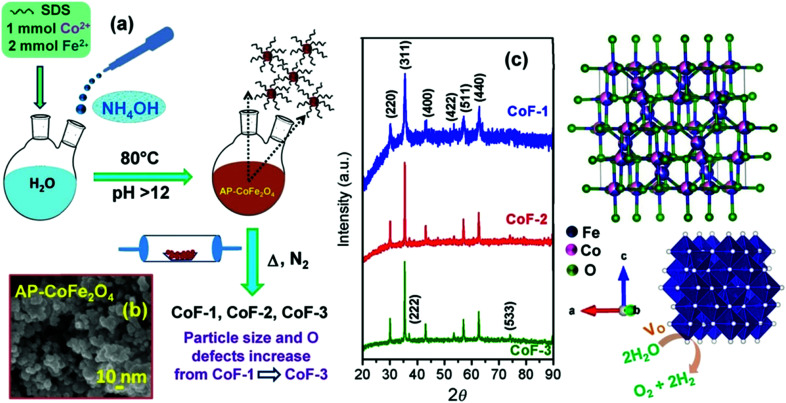
(a) Schematic illustration of the formation of AP-CoFe_2_O_4_ through the coprecipitation method followed by thermal treatment under N_2_ to obtain CoF-1, CoF-2, and CoF-3. (b) Field-emission (FE) SEM image of AP-CoFe_2_O_4_. (c) XRD patterns of CoF-1, CoF-2, and CoF-3 NPs. Reproduced with permission from ref. 811. Copyright Wiley 2020.

**Fig. 48:**
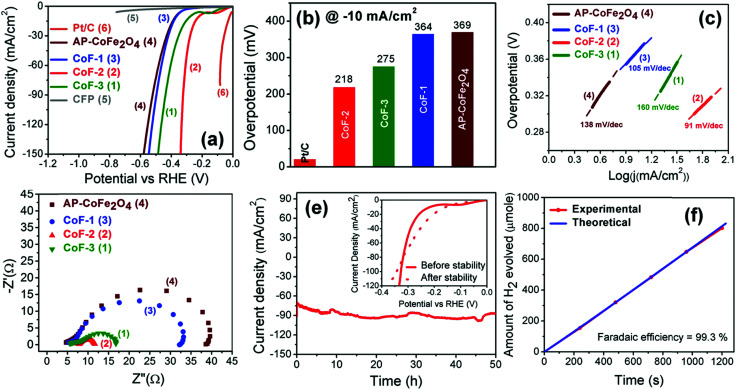
(a) LSV polarisation curves for the HER. (b) Overpotential values reach a current density of 10 mA cm^−2^. (c) Tafel and (d) Nyquist plots of CoFe_2_O_4_ NPs for the HER recorded at 0.4 V *versus* RHE. (e) Chronoamperometric stability test for CoF-2 performed at 0.35 V *versus* RHE. Inset shows the LSV polarisation curves before and after stability tests. (f) Experimental and theoretical gas evolution at 0.764 V *versus* RHE for the HER of CoF-2. Reproduced with permission from ref. 811. Copyright Wiley 2020.

Oxygen-defect density is a useful control tool to adjust the electrocatalytic properties that are relevant for water splitting.^811^ The work covers multi pH water-splitting (alkaline-, neutral and acidic pH) and in addition seawater electrolysis. CoFe_2_O_4_ NPs have been generated by precipitation and the as-prepared material (AP-CoFe_2_O_4_) was calcinated at 350 °C, 550 °C and 650 °C (samples CoF-1, CoF-2 and CoF-3), which resulted in an increase of the particle size from 8 nm (AP-CoFe_2_O_4_), 10 nm, 20 nm and 55 nm (samples CoF-1, CoF-2 and CoF-3; Fig. 47). Sample CoF-2 showed the best intrinsic HER activity (*η* = 218 mV, *j* = 10 mA cm^−2^) for water electrolysis carried out at pH 14 (Fig. 48).

In summary, spinel-based materials are more predestined to act as oxygen-evolving electrodes than as hydrogen-evolving electrodes (a large number of papers are dedicated to spinel-structured materials for the OER), which stems from the intrinsically-larger oxide materials stability in oxidising (OER) than in reducing (HER) conditions. However, recent efforts clearly show that, upon suitable design strategy based on *e.g.*, oxygen vacancy engineering to increase the density of catalytic active sites or doping that may end in better electrical conductivity, highly active and durable spinel structured HER electrocatalysts can also be achieved.

### 7.4 Transition metal layered double hydroxide OER catalysts for alkaline electrolytes

Late 3d transition metal-based (Ni, Fe, Co, Mn) hydroxides and oxyhydroxides (generally indicated in the following as (oxy)hydroxides) comprise highly active catalysts for the OER in alkaline and neutral pH electrolytes.^812^ Besides their direct synthesis, surface reconstruction of metal or metal oxide nanoparticles and electrodes in alkaline electrolyte might also result in formation of surface metal oxyhydroxides acting as the OER catalysts (see for example the section related to OER on steels, the surface of which can be close to (oxy)hydroxides). For this reason and the very high activity reported for some of these catalysts (Table 9), they represent an interesting area of research for both fundamental insights into the OER mechanism and practical application as anode catalysts in water electrolysers.

#### 7.4.1 Crystal structure of single-metal based (oxy)hydroxide OER catalysts

**Fig. 49:**
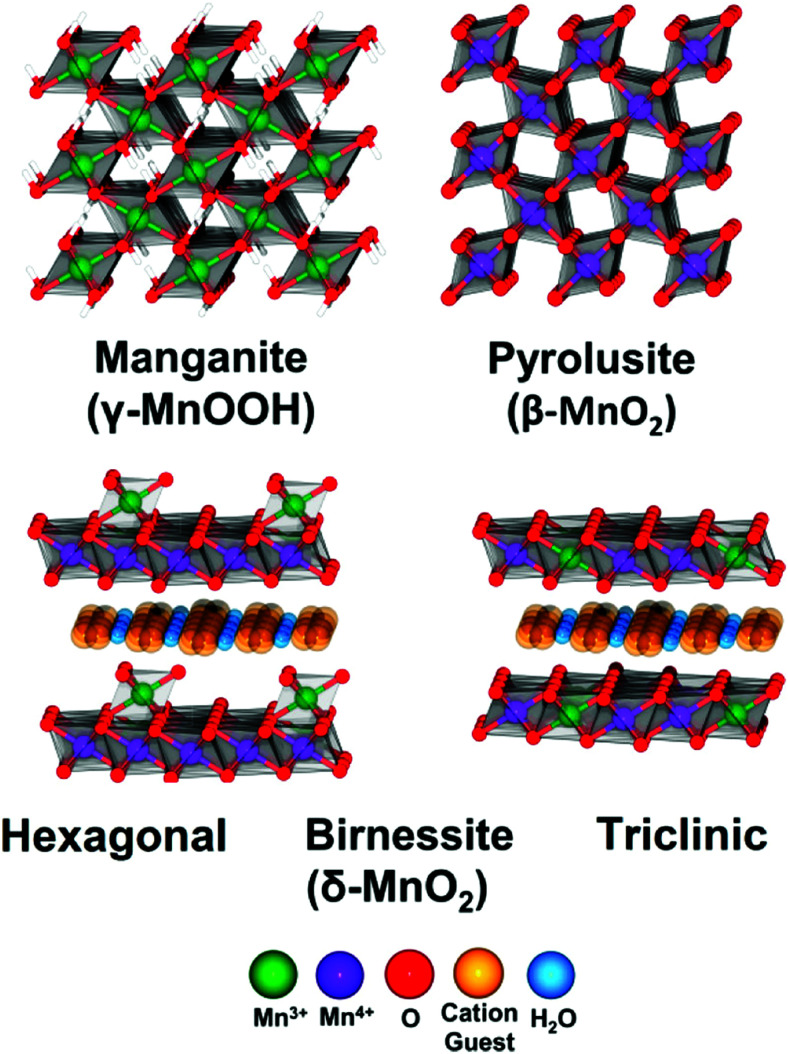
Typical structures of selected Mn-oxides and oxyhydroxides. Reproduced with permission from ref. 820. Copyright ACS 2016.

Single-metal based (oxy)hydroxides, while not being among the most active OER catalysts within this material family, provide the basis on which more complex and active multinary (oxy)hydroxides can be designed. Therefore, their study is important to provide fundamental insights and guide the rational design of improved catalysts. Among them, Ni hydroxides can be prepared in the crystalline brucite-like β-phase, β-Ni(OH)_2_, which is characterised by layers of edge-sharing octahedra, where the metal atoms occupy the center of the octahedra and OH groups the corners. Co hydroxides can also be synthesised in this structure (β-Co(OH)_2_). In addition, other phases have been reported, *i.e.*, water intercalated Ni(OH)_2_ (α-phase) and others characterised by various types of defects and turbostratic disorder. Ni oxyhydroxide phases, *i.e.*, anhydrous β-NiOOH and water and cation intercalated γ-NiOOH, form under applied anodic potentials. Co hydroxides also transform to the oxyhydroxide phase (β-CoOOH) under applied anodic potentials. Therefore, for these two families of catalysts the as-prepared hydroxide phases are not the catalytically-active phases for OER, which typically occurs at higher potentials than the oxidation of the metal centres from 2+ to higher oxidation states (*i.e.* ∼1.4 V and ∼1.1 V *vs.* RHE for Ni(OH)_2_ and Co(OH)_2_, respectively). Fe centers with 2+ oxidation state being unstable in oxygen environment, Fe oxyhydroxides are typically obtained instead of Fe hydroxides (Fe^2+^Fe^3+^ layered double hydroxide, also known as “green rust”, can be synthesised but is unstable in air). Atomic structures for Fe oxyhydroxides range widely from diaspore-type α-FeOOH,^813^ boehmite-type γ-FeOOH and other γ-polymorphs,^813^ to β-FeOOH.^814^ Crystalline and amorphous manganese oxides and oxyhydroxides (structural depictions of which are displayed in Fig. 49) have been investigated for acidic, neutral, and alkaline water oxidation.^815–819^ Among the oxyhydroxide phases, manganite γ-MnOOH showed better catalytic performance than other MnO_*x*_ materials and consists of corner-linked octahedra.^820^ Feitknechtite β-MnOOH has also been observed as one of the components of an active multiphase Mn-based electrocatalyst.^821^ γ-MnOOH is the most stable polymorph of MnOOH, however it was also observed during OER to convert into MnO_2_ and deactivate, revealing a general instability issue for Mn oxyhydroxides.^820^

#### 7.4.2 Crystal structure of binary and multiple transition metal (oxy)hydroxide OER catalysts

**Fig. 50:**
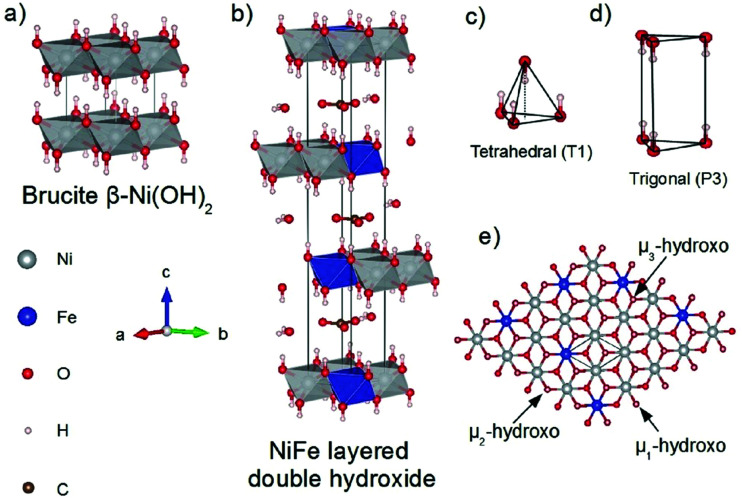
Typical structure of Brucite type Ni(OH)_2_ and hydrotalcite-like NiFe LDH. Reproduced with permission from ref. 812. Copyright Wiley 2016.

Introducing metal with oxidation states 3+ into a metal hydroxide host where the host metals are in oxidation states 2+, leads to the intercalation of charge-compensating anions and water in the region between the brucite-like metal hydroxide layers. This structure, typical of the mineral hydrotalcite, is known as layered double hydroxide (LDH) crystal structure.^812^Fig. 50 shows a comparison of the crystal structure of β-Ni(OH)_2_ (Brucite) and of NiFe LDH. Similarly, to Ni(OH)_2_, it was confirmed for NiFe and CoFe LDH that the prepared crystal structure (α-LDH) deprotonate under potential control, transforming into a γ-LDH phase, which is the catalytically active phase under OER^822^ and is characterised by contracted interlayer and intralayer atomic distances and switching of intercalated anions to cations.

Ternary and multiple metal-based (oxy)hydroxides have also been investigated, where the additional metals have been introduced as dopants into the synthesis of the binary metal LDHs,^823^ and by systematic compositional studies, for example, by high through-put methods.^824,825^ Selected examples are discussed in the following section.

#### 7.4.3 OER activity and stability trends among transition metal (oxy)hydroxides

The OER activity trend among the Ni, Fe, Co, Mn monometallic (oxy)hydroxides in alkaline electrolytes that have been purified from Fe impurities reveals that when Fe oxyhydroxides are deposited as ultrathin film or small clusters on a conductive electrode, they show the highest activity.^826,827^ Due to the poor electrical conduction of Fe oxyhydroxides, the performance of these materials is severely hindered with thicker electrodes.^826^ Co (oxy)hydroxides follow in the activity trend, while pure Ni (oxy)hydrixides and Mn (oxy)hydroxides show the lowest activity. However, due to different metal dissolution rates, the stability trend was found to be the opposite: NiO_*x*_H_*y*_ > CoO_*x*_H_*y*_ ≫ FeO_*x*_H_*y*_.^827^

**Fig. 51:**
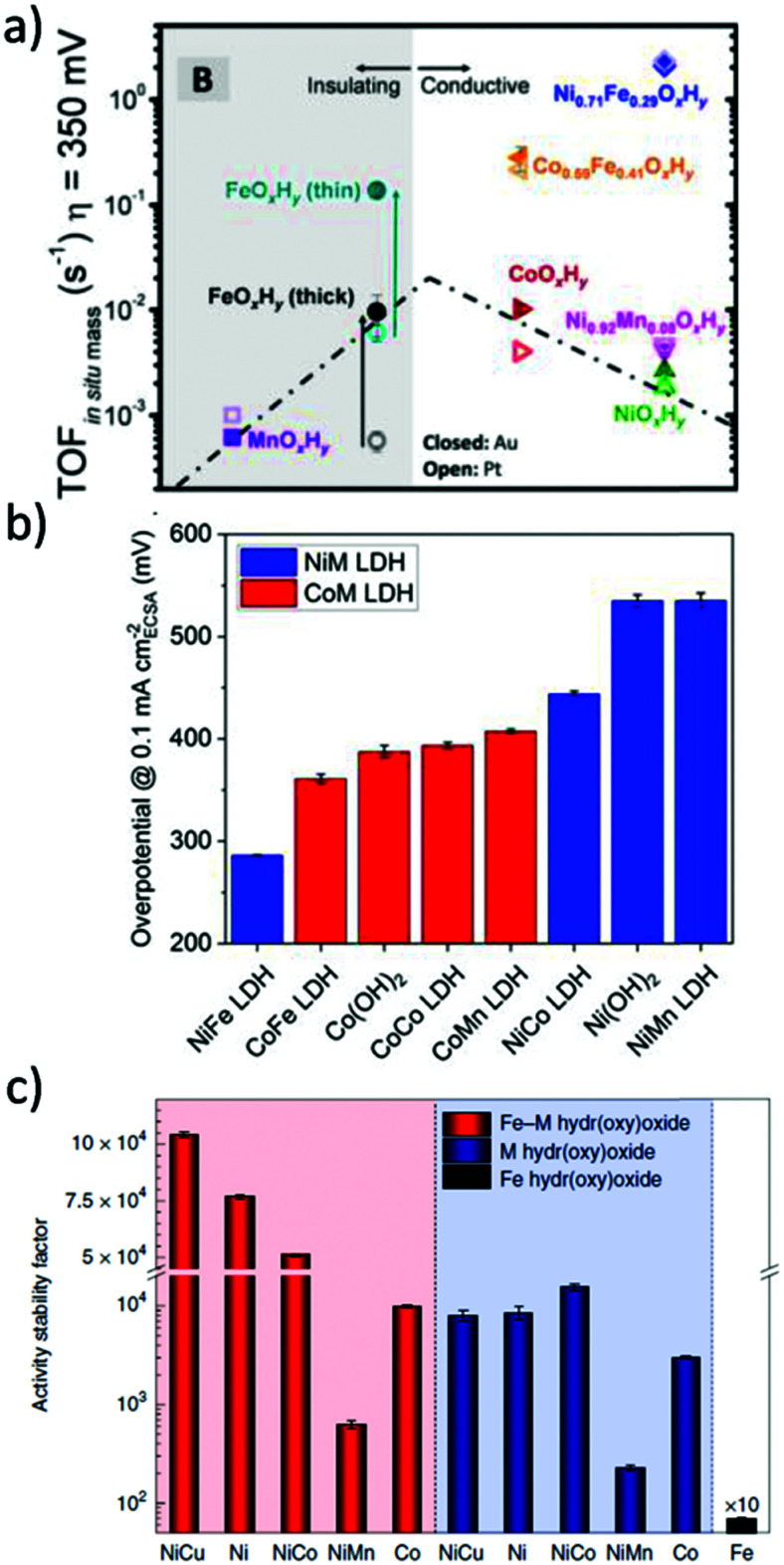
Transition metal (oxy)hydroxides and LDHs OER performance trends. (a) Activity trend as effective turnover frequencies (TOF) at overpotential *η* = 350 mV and based on the total mass of the electrodeposited catalyst films calculated from quartz crystal microbalance measurements and ordered based on the atomic number of the host/primary metal cation. Electrolyte: 1 M KOH. Reproduced from ref. 826. Copyright American Chemical Society 2015. (b) Intrinsic activity trend as OER overpotentials at ECSA-normalised current densities of 0.1 mA cm^−2^_ECSA_ for crystalline transition metal LDHs. Electrolyte: 0.1 M KOH. Reproduced from ref. 831. Copyright Wiley 2021. (c) Activity stability factor (ASF) trend for Fe containing (red bars) and Fe-free (blue bars) transition metal hydroxy oxide clusters. The Fe containing catalysts were obtained by adding Fe nitrate to the 0.1 M KOH electrolyte. Reproduced with permission from ref. 827. Nature Publishing 2020.

A thorough purification of the alkaline electrolyte is important when benchmarking these catalysts, since trace amounts of Fe impurities in the electrolyte significantly enhances the activity of Ni-based and Co-based (oxy)hydroxides significantly.^104,828^ Consequently, Fe-activated Ni hydroxide catalysts and, in general, NiFe (oxy)hydroxides are among the most active OER electrocatalysts at alkaline pH (Fig. 51a). CoFe oxyhydroxides are also more active than Co oxyhydroxides.^103^ To evaluate the catalytic activity of transition metal (oxy)hydroxides the OER overpotentials are typically compared at a fixed geometric current density, *i.e.*, *j* = 10 mA cm^−2^ (different types of intrinsic activity metrics were proposed in the literature (see Section 7.2). However, determining the intrinsic activity for these catalysts is challenging, since the nature of the active sites is often unknown, and their surface concentration is also difficult to estimate due to rough surface morphologies. This affects the calculation of turn over frequencies (TOF). Also, the evaluation of the electrochemical surface area (ECSA) to calculate surface specific activities must be performed with care, since the electrical conductivity of LDHs changes with the applied potentials^829^ resulting in a narrow or non-existing potential window that is free of faradaic current in the conductive regime. This limits the range of potentials where certain electrochemical techniques can be applied, such as the ones based on cyclic voltammetry or electrochemical impedance spectroscopy (EIS). Furthermore, metal oxidation peaks in cyclic voltammograms often overlap with the OER faradaic current and model catalysts with smooth planar surfaces for the conversion of the calculated values, *i.e.*, capacitances, in the unit of an area are not always available.^830^ Recently, a method to calculate the ECSA based on the capacitance of the adsorbed OER intermediates (*C*_a_), instead of the more commonly used double layer capacitance, was proposed for a series of transition metal based LDH catalysts.^831^*C*_a_ was calculated by EIS at 1.6 V_RHE_, and normalised by the specific unit area capacitance that was obtained from a smooth Ni(OH)_2_ surface from ref. 832. This method surpasses most of the mentioned limitations, providing surface-based intrinsic activities and calls for new experiments that provide specific unit area capacitances for the different LDHs. The general intrinsic activity trend was NiFe LDH > CoFe LDH > Fe-free Co-containing catalysts > Fe-Co-free Ni-based catalysts (Fig. 51b).

In contrast to the activity, these catalysts stability has been less systematically investigated. The most commonly performed stability tests range from short term stability tests (2 hours) at low current densities of (*j* = 10 mA cm^−2^)^833^ mostly used for preliminary screening, to galvanostatic stability tests over longer time, for example hundreds of hours,^834,835^ as well as at higher current densities (>100 mA cm^−2^).^835,836^ Chronoamperometry measurements, for example at the applied cell potential of 1.6 V,^835^ have been also used as well as protocols simulating the natural day-night light cycle,^835^ and stability tests at higher temperatures (>80 °C)^837^ and high KOH concentration (>1 M).^835,836^ Most of these studies were performed on NiFe (oxy)hydroxide catalysts. At room temperature, stability tests of NiFe (oxy)hydroxide catalysts generally show very promising results with the stability of dozens of hours, and in some cases even more. However, stability tests must be ran also at operation-relevant temperatures for alkaline electrolysers, *i.e.*, ∼80 °C.^838^ Recently, Chung *et al.* extended to late 3d transition metal (oxy)hydroxide catalysts a previously proposed metrics called activity-stability factor (ASF)^839^ that takes into account both activity and stability (Fig. 51c).^827^ This was obtained by the evaluation of both the OER activity, in terms of current densities at 1.7 V *vs.* RHE, and the stability, as the rates of metal dissolution. Finally, the ASF was calculated as activity/stability ratio and expressed the amount of O_2_ that is produced per dissolved active site. Ni-based and Co-based (oxy)hydroxide clusters that incorporated Fe, which were prepared by adding Fe nitrate to the electrolytes, showed higher ASF than their Fe-free analogues. In these Fe-containing catalysts the authors found higher Fe dissolution than the metal host dissolution, suggesting that the poor activity retention in Fe-free electrolytes was related to the dissolution of Fe active sites. Finally, they suggested that Fe dissolution and electrochemical re-deposition yields dynamically stable Fe active sites, providing a strategy for designing better catalysts.

In the following sections, we will focus mostly on Ni-based (NiFe) LDH and (oxy)hydroxide catalysts, which have been the most investigated in alkaline electrolytes, and on their activity (for their stability we refer to the discussion in this section). Later, selected results obtained with the other transition metal LDH and (oxy)hydroxide catalysts will be summarised.

#### 7.4.4 OER activity of NiFe (oxy)hydroxide catalysts

NiFe LDHs, and more generally NiFe oxyhydroxides, are among the most active OER catalysts in alkaline electrolyte.^812,840,841^ The most common methods to synthesise NiFe (oxy)hydroxides consist of electrodeposition,^104,829,1279^ co-precipitation at constant pH^842^ homogeneous precipitation methods involving solvothermal or hydrothermal treatments,^112,843–848,1864^ phase transformation by soft chemistry (chimie douce),^849^ or electrochemical conditioning in alkaline electrolyte (without or in the presence of Fe impurities in the case of uptake using a Ni oxide/hydroxide electrode)^128,850,851,1792^ pulsed-LASER ablation in liquid^852^ and photochemical metal-organic deposition.^853,854^

In particular, the electrochemical conditioning in alkaline electrolyte that leads to activation of an NiFe oxyhydroxide surface allowed the investigations of NiFe-based pre-catalysts with different electrical conductivity and structural properties, such as metal alloys,^850,1752^ phosphides,^855^ sulphides,^856–858^ (oxy)fluoride^859,860^ and selenides.^861,862^ A detailed review of these materials can be found in ref. 863. In addition, NiFe-based nitrides have also been investigated.^864–866^ Furthermore, composite and hybrid catalyst materials employing NiFe (oxy)hydroxide and nanocarbon materials were also prepared to achieve better active sites utilisation and improve the electrical conductivity.^844,867–871^ Besides carbon, different supports have also been investigated, and gold has been found to affect the intrinsic activity of NiFe (oxy)hydroxide thin films.^104,851,872–875^ However, the authors note that carbon cannot be considered a stable support for OER operation, according to its unavoidable corrosion in such alkaline oxidising conditions, in particular in presence of metal (-oxide) catalysts.^876,877,1361,1363–1365,1367^

**Fig. 52:**
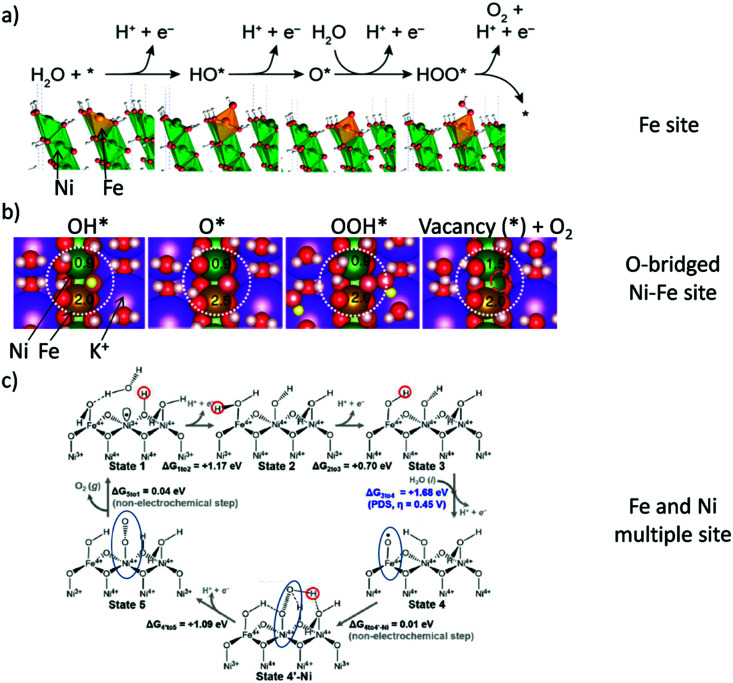
NiFe (oxy)hydroxide active site and OER mechanism by DFT calculations. (a) Proposed OER pathway involving the HO*, O* and HOO* intermediates and with a Fe atom site that was substituted in the (011̄2) surface of γ-NiOOH as the active site. Reproduced with permission from ref. 878. American Chemical Society. (b) A second proposed OER mechanism and intermediates on the H-saturated O-bridged Ni–Fe site as active site at the (01–10) surface of γ-NiFe LDH. The reaction centers are highlighted by large dotted white circles, the vacancy by a small pink dashed circle. The magnetic moments of Ni and Fe during OER are also given. Reproduced with permission from ref. 1775. Copyright Nature Publishing 2020. (c) A third example of proposed mechanism for OER on Ni_1−*x*_Fe_*x*_OOH catalyst. Blue ovals highlight the synergistic role of Ni and Fe sites in forming key reaction intermediates. Reproduced with permission from ref. 888. Copyright American Chemical Society 2018.

The OER activity of NiFe (oxy)hydroxides was found to depend on many parameters, including Fe content, electrolyte pH, cations, and structural disorder, among others. Fe incorporation in Ni-based (oxy)hydroxides catalysts^821,878,879,1244,1771^ decreases in the overpotentials by 200–300 mV with respect to Fe-free Ni(OH)_2_ and generally a maximum in activity is reached for 10–50% Fe metal content.^812^ Fe metal sites at the high index surfaces of Fe doped γ-NiOOH have been suggested as the OER active sites by a combination of *operando* X-ray absorption spectroscopy (XAS) and DFT+*U* calculations (Fig. 52a).^878^ The classical OER mechanism where the adsorbed OH*, O*, OOH*, intermediates form on top of the active site was considered.^127,880^ Several works and results supported this hypothesis,^842,851^ while others considered alternative mechanisms and sites.^881–883^ For example, the electronic effect of Fe atoms on Ni sites, which acted as superior Lewis acid and promoted the formation of tetravalent Ni, was discussed by Li *et al.*^884^ In their proposed mechanism, the increased population of Ni^4+^ leads to greater Ni–O covalency, and thus greater oxyl character by Ni(iii)–O˙ resonant contribution, with the oxyl radical finally promoting O–O formation. Drevon *et al.* performed *in situ* XAS at the oxygen K-edge and their results are consistent with the presence of an electron deficient oxygen site prior to O–O formation.^885^ This observation might be related to the superoxo species (NiOO^−^) or “negatively charged oxygen” ligands that were previously proposed to participate to the OER mechanism on NiFe and Ni (oxy)hydroxide catalysts.^78,110,886^ Recently, DFT calculations by Dionigi and Zeng *et al.* confirmed that Fe sites are more active than Ni sites, but revealed that O-bridged Fe–Ni reaction centers and the synergy between the two metals stabilise OER intermediates that are unfavourable on single Fe sites or on O-bridged metal-metal sites of the same metal (Fig. 52b).^1775^ Therefore, they proposed that the bridging oxygen between Ni and Fe atoms in the γ-phase of NiFe LDH is the active sites. The OER mechanism starts from the deprotonation of that bridging O site, which is saturated by H under OER conditions according to the calculated surface phase diagrams and follows a Mars-van-Krevelen mechanism involving the surface lattice oxygen. With the O_2_ release, a vacancy is formed that will be refilled in the next cycle by OH^−^ from the electrolyte.

The hypothesis of lattice oxygen involvement into the OER mechanism (LOER) was also investigated by isotope labelling experiments.^847,850,887^ Roy *et al.* investigated electrochemically activated NiFe alloy nanoparticles using isotope-labelling experiments with an electrochemical mass spectrometry setup and concluded that the OER is only limited to the near-surface region and does not proceed *via* lattice oxygen exchange.^850^ Following a different experimental approach, Lee *et al.* performed ^18^O-labeling experiments in combination with *in situ* Raman spectroscopy:^847^ lattice oxygen participation in the OER for Fe-free NiOOH was proposed, probably *via* formation of NiOO^−^ intermediates. However, while oxygen exchange was observed in ultrathin NiFe LDH if the catalyst was in the reduced state (Ni atoms in Ni^2+^ state), the experiments with oxidised ultrathin NiFe LDH catalyst agreed with Roy *et al.*^850^ Recently, LOER was directly confirmed for Fe-free Ni(OH)_2_/NiOOH by Ferreira *et al.* by using a novel differential electrochemical mass spectrometry (DEMS) cell interface and isotope labelling experiments.^887^ Furthermore, the authors observed evidences of LOER also for NiFe LDH, supporting previous hypothesis of a Mars-Van-Krevelen mechanism.^850,1775^ Differences in the literature regarding the detection of LOER on NiFe oxyhydroxide catalysts may be caused by rapid ligand exchange prior to LOER detection, different pre-treatment protocols or arising from structural differences among the investigated catalysts.

The synergy between Ni and Fe as the origin of the enhanced activity was also proposed by Goddard and co-workers.^111,888^ Their proposed mechanism involved multiple sites and their DFT calculations revealed that the formation of a key O˙ intermediate is stabilised on the high spin d^4^ Fe^4+^ site, while the subsequent O–O coupling is catalysed on low spin d^6^ Ni^4+^ site (Fig. 52c). The deprotonation of the OOH adsorbed on Ni^4+^ forms O_2_^−^ on the Ni^4+^ site, in agreement with previous experimental findings that suggested a NiOO^−^ species^78^ before the final release of O_2_. Despite no universally accepted mechanism is agreed on, these works highlight the importance of Fe and especially its interaction and synergy with Ni, as the origin of the enhanced (and stabilised) OER activity of NiFe (oxy)hydroxides.

Another important factor affecting the activity of NiFe oxyhydroxides is the pH of the electrolyte, as revealed by the super-Nernstian behaviour on the NHE scale.^844,889,1864^

Electrolyte alkaline cations have also been observed to have an effect on the activity of NiFe oxyhydroxide catalysts, following generally the trends of increasing activity from smaller to larger cations, *i.e.* Cs^+^ > Na^+^ ≈ K^+^ > Li^+^,^79,889^ and K^+^ ≈ Mg^2+^ ≥ Na^+^ ≫ Ca^2+^.^890^ Such a trend was suggested to result from intrinsic effects due to modification of the adsorption energies of OER intermediates (*OH, *O, *OOH) on Ni(Fe)OOH,^890^ and to better stabilisation by larger cations of the superoxo OER intermediate (NiOO^−^) in Fe-free NiOOH.^79^ Recently, it has been highlighted that intrinsic cation effects should be carefully decoupled from indirect pH effects:^889^ at parity of cation concentration, the electrolyte pH increases from small to large alkali metal cations in the order LiOH < NaOH < KOH< RbOH < CsOH. After taking into account pH differences, the intrinsic promoting effect of alkaline cations on the activity was found to be minor, when compared to Fe substitution and pH differences,^889^ but still revealed that a lower activity is obtained with Li+ in respect, for example, to K+, as also previously observed with 316L activated steel, which presents a near similar surface structure as NiFe LDHs and (oxy)hydroxides.^1277^

Besides cations, anions have also been the subject of experimental and theoretical studies. Many intercalated anions have been shown to quickly exchange to carbonate when the electrolyte is in equilibrium with ambient conditions.^891^ However, by carefully employing carbonate-free electrolyte, Hunter *et al.* found that the activity correlates with the pKa of the conjugate acid of the interlayer anions.^891^ Zhou *et al.* showed by DFT calculations that the intercalated anions affect the electronic structure of surface metal atoms, which may lead to higher activity.^892^

The impact of structural disorder on the activity was also investigated. Fe incorporation in NiOOH was found to lead to higher structural disorder.^893,1279^ From XAS observation, Smith *et al.* reported structural distortions on the oxidised form of a series of NiFe oxyhydroxide catalysts that were induced by Fe incorporation^854^ and proposed the introduction of localised structural distortions as strategy to improve the Ni-based (oxy)hydroxide activity. Lattice distortion and introduction of tensile strain into NiFe-LDH was also obtained by Zhou *et al.* by ball milling and correlated with improved performance.^894^ Recently, Lee *et al.* found a volcano-type correlation with the structural disorder and the TOF of Fe sites as a function of Fe content.^893^ Therefore, they suggested that structural disorder should be optimised to improve NiFe LDH activity.

#### 7.4.5 Trimetallic and multimetallic LDH and oxyhydroxide catalysts

The investigation of ternary and multinary transition metal (oxy)hydroxides aims to overcome the catalytic performance of the most active binary NiFe and CoFe (oxy)hydroxides.

For CoFe (oxy)hydroxides, Zhang *et al.* reported a CoFeW oxyhydroxide catalyst in which tungsten modulated the electronic structure of the catalyst, resulting in enhanced activity.^823^ Cr was also reported to enhance the activity of CoFe (oxy)hydroxides, and Chen *et al.* found an optimal composition of Co_5_Fe_3_Cr_2_ (oxy)hydroxide, with Cr affecting the Co electronic structure, resulting in higher TOF.^895^ Among Co-based quaternary metal oxyhydroxides, Zhang *et al.* reported ultrathin CoCuFeMo (oxy)hydroxides nanosheets with enhanced OER performance.^896^

Cr addition was also investigated for NiFe (oxy)hydroxides:^897,898^ electrodeposited NiFeCr (oxy)hydroxide thin film catalyst where Cr was found to dissolve and re-deposit on the surface^898^ showed higher activity than the bimetallic NiFe oxyhydroxide catalyst and the authors suggested Cr^6+^ sites as the active sites, which was supported by DFT calculations. Iron and vanadium co-doped nickel (oxy)hydroxide was reported by Jiang *et al.*:^899^ the metal composition of Ni_3_Fe_0.5_V_0.5_ resulting in highest activity and the authors suggested combining DFT and XAS that the V site with neighbouring Fe atoms is the active site. Other investigated metal additions to NiFe oxyhydroxides include Mo,^900^ Ce,^901^ and Mn.^902^ Chung *et al.* proposed Fe–NiCu oxyhydroxide as a new promising catalyst, showing higher activity than the Cu-free Fe–Ni catalyst and a remarkable stability, leading to the highest ASF among the investigated catalysts.^827^

Many researchers also studied the possible synergies arising from Ni–Co–Fe compositions, and the results generally pointed to a small enhancement *versus* binary NiFe (oxy)hydroxide catalysts, which suggested a minor but positive synergistic effect.^903–905,1752,1794^ High-throughput mapping methods screening generally metal oxides, highlighted metal combinations such as Ni–Fe–Co–Ce,^824^ Ni–Fe–Al, Ni–Fe–Ga, and Ni–Fe–Cr,^825^ which, on the basis of possible surface reconstruction, are also promising for metal (oxy)hydroxides.

Zhang *et al.* performed DFT calculations on both NiFeX and NiCoX series, where X (X = W, Mo, Nb, Ta, Re and MoW) consisted in a high-valence transition-metal dopant and acted as modulator of the electronic structure:^906^ for both NiFeX and CoFeX oxyhydroxides, the presence of Mo and W significantly enhances the activity. Furthermore, the quaternary CoFeMoW oxyhydroxide is expected to be slightly more active than the ternary CoFeW and CoFeMo based catalysts, while the same is not expected for the NiFe-based series. Experiments in 1.0 M KOH agreed with the calculation results. The stability was high: the NiFeMo oxyhydroxide catalyst was stable in an electrolyser anode that delivered 300 mA cm^−2^ at ∼1.7 V consistently over 120 h in 30% KOH electrolyte at 85 °C.

**Fig. 53:**
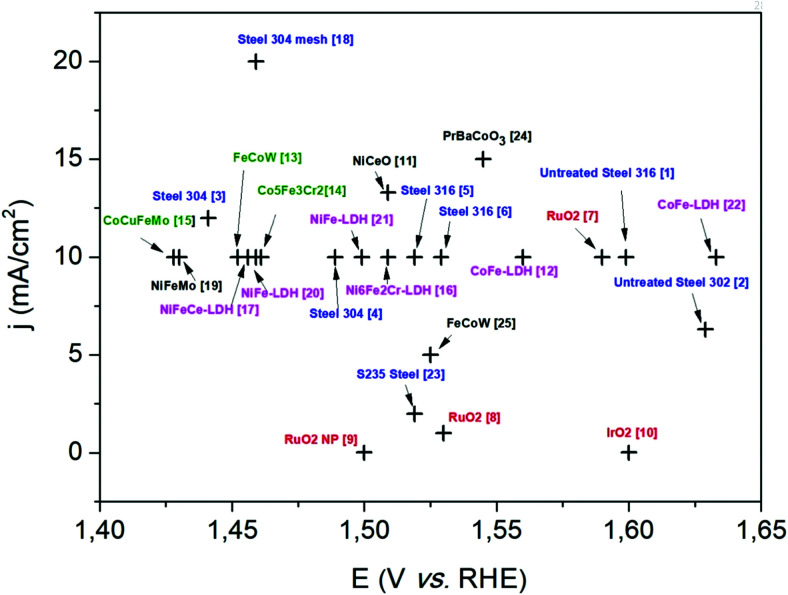
A comparison of the OER activity of commercially available OER electrode materials like RuO_2_, IrO_2_ (highlighted in red), steel based OER materials (highlighted in blue), transition metal layered double hydroxides (pink), oxyhydroxides (black) and other state of the art OER electrocatalysts (green). The displayed OER materials are unmodified AISI 316 steel (1),^1238^ unmodified AISI 302 steel (2),^1275^*ex situ* modified steel 304 (3),^1286^*ex situ* modified steel 304 (4),^370^*ex situ* modified steel 316 (5),^1295^*ex situ* modified steel 302 (6),^1222^ RuO_2_ (7),^908^ RuO_2_ (8),^908^ RuO_2_ nanoparticles (9),^26^ IrO_2_^908^ (10), NiCeO on gold (11),^909^ CoFe LDH (12),^823^ gelled FeCoW oxyhydroxide (13),^823^ Co_5_Fe_3_Cr_2_ (oxy)hydroxide (14),^895^ CoCuFeMo(oxy)hydroxide (15),^896^ Ni_6_Fe_2_Cr LDH (16),^897^ NiFeCe LDH (17),^901^*ex situ* modified steel 304 mesh (18),^1228^ NiFeMo (19),^906^ Porous monolayer NiFe LDH (20),^846^ NiFe LDH (21),^894^ CoFe LDH (22),^1775^*ex situ* modified steel S235 (23),^1288^ PrBaCoO_3_ (24),^591^ FeCoW oxyhydroxide (25).^823^

Finally, it is worth to mention that NiFe LDH when employed as HER/OER bifunctional catalyst is able to reach very low overpotentials.^113,907^ For this catalyst a dynamical self-optimization mechanism was reported, which involved an increased crystallinity at the cathode during HER, resulting in a significantly lower HER overpotential than the pristine catalyst.^113^ Further bifunctional electrocatalysts will be discussed in Section 8.3. Fig. 53 displays a comparison of the OER activity determined in 1 M KOH of commercially available OER materials, *i.e.*, currently used in electrolyzers (RuO_2_, IrO_2_, stainless steels) together with the corresponding data of the materials discussed in Section 7.4.

To summarise this section, typical metals introduced to boost the catalytic performance of binary NiFe and CoFe (oxy)hydroxides, consist mainly in non-3d high-valence transition-metal cations, such as Mo^6+^, and W^6+^, the Co–Ni–Fe combination and other 3d transition-metals such as Cr, V and Cu, while Ce, Ga, and Al are also considered promising dopants.

Other examples incorporating non-metallic elements such as P, N, S, Se and F, as well as composite catalysts involving nanocarbon materials, have been briefly discussed in the case of NiFe (oxy)hydroxide in the corresponding section. For these catalysts, often a surface reconstruction to oxyhydroxides is expected. Nonetheless, improvements in electrical conductivity and catalyst site accessibility have been observed.^827,1752^

### 7.5 Compounds of metals and group 3, 4, 5, 6 non-metals as HER electrocatalysts

To avoid a broad content-related overlap with other sections, Section 7.5 is exclusively devoted to compounds that consist of metal elements and non-metal elements, whereby the non-metallic elements should be limited to elements of main groups 3, 4, 5 and 6 with the exception of oxygen. The reader will find a solid number of review articles dealing in whole^915–919^ or in part^920–927^ with the subject of Section 7.5, and is directed to these articles and additional information on these subjects. The compounds discussed in this subsection are divided into five classes (i) metal borides (Section 7.5.1), (ii) metal carbides (Section 7.5.2), (iii) metal pnictides (Section 7.5.3), (iv) metal chalcogenides (7.5.4) and (v) metal-nonmetal compounds bearing different nonmetal elements (Section 7.5.5). Section 7.5 is in principle restricted to compounds that are noble elements-free, except for Rh_2_C and RuB_2_ which fit better here than in Section 6.

**Fig. 54:**
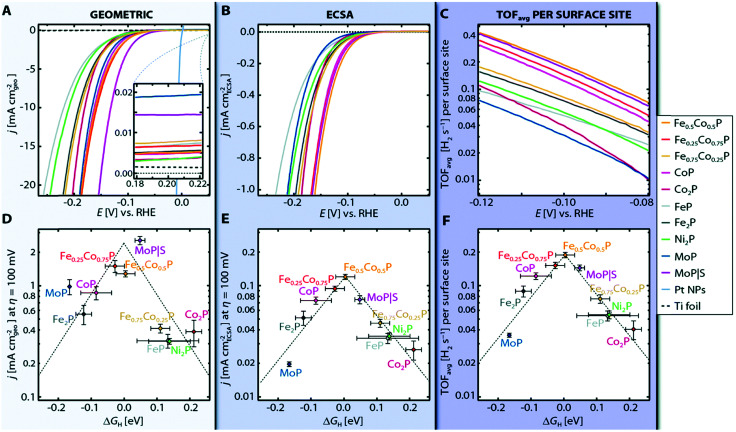
(a–c) HER activity of TMPs. (A) Linear sweep voltammograms (LVSs) per geometric area of representative TMP electrodes. The HER activity of Pt nanoparticles (NPs) is displayed for comparison. (d) Activity volcano for the HER showing the geometric current density from (A) at an overpotential of *Z* = 100 mV as a function of hydrogen adsorption free energy (DGH). (e) Activity volcano for the HER showing the ECSA normalised current density from (B) at *Z* = 100 mV as a function of DGH. (f) Activity volcano for the HER showing the average TOF from (C) at *Z* = 100 mV as a function of DGH. Reproduced with permission from ref. 928. Copyright RSC 2015.

The HER intermediate being the H-adsorbed active site after electrochemical discharge of a proton, an efficient HER electrocatalyst is characterised by neither too high nor too low M–H_ads_ bond strengths. The HER efficiencies of a series of catalysts can be estimated by calculating the standard free energies of H adsorption, *e.g.*, by DFT calculations. These enable to construct volcano-shape relations *e.g.*, between Δ*G*_H*_ and the exchange current density, the compounds present at or near the top of the diagram being considered HER active: typical representatives of compounds assigned to Section 7.5 are arranged relatively high in such plots (Fig. 54).^928^ This theoretical-analytical approach clarifies why *e.g.*, binary metal-non-metal species are promising HER electrocatalysts. When comparing binary metal oxides with binary metal-sulphur or metal-phosphorus compounds, one expects oxides to be more sensitive to reductive potentials than metal-sulphur or metal-phosphorus compounds, since the negative charge density (localised at the central metal ion in the metal-S or metal-P species) is higher than for the metal oxides (oxygen is more electronegative than S or P). As mentioned in Sections 7.2 and 7.3, metal oxides are (due to reductive conditions that occur on the HER side) less durable upon HER operation. This qualitative reasoning explains why metal chalcogenides with the heavier elements from main group 6 are better-suited than metal oxides for HER electrocatalysis. This agrees very well with the observation that phosphide-, sulfide- and selenide- based surfaces in many cases are transformed into their corresponding oxides during catalysis, with only their core intact with the pre-catalyst (at least under oxidative conditions).

#### 7.5.1 Metal-borides used as electrocatalysts for supporting the hydrogen evolution reaction

In their pioneering work Paul *et al.*^929^ discovered that nickel boride (Ni_2_B) doped with small amounts of Mo, W, Cr is more HER active than RANEY® Nickel. Nickel boride was further investigated for its cathodic water splitting ability in the 1970s.^930^

Amorphous nickel boride (Ni_2_B) as well as heterogeneous mixtures of nickel boride and nickel were checked for their electrocatalytic HER properties in 1 M NaOH solution at 70 °C by Los *et al.*^931^ At only 113 mV overpotential a current density of 250 mA cm^−2^ was reached, which is a fantastic performance, assuming the simplicity of the electrode preparation procedure.

Nickel boride, with which the development of HER electrodes based on metal boride was started, is still being discussed and further developed.^932–937^ Thus, electroless plated NiB_0.54_ film exhibited high activity for electrocatalytic H_2_ evolution (*j* = 10 mA cm^−2^ at overpotentials (*η*) of 45 mV in 0.5 M H_2_SO_4_, 54 mV in 1.0 M pH 7 phosphate buffer solution (PBS), and 135 mV in 1.0 M KOH).^933^

MoB, purchased from commercial sources, was used as a working electrode supporting hydrogen evolution upon using a Pt counter electrode in alkaline (pH 14) and acidic regime (pH 0) by Vrubel *et al.*^938^ MoB was found reasonably-active (*η* = 195 mV, *j* = 10 mA cm^−2^, pH 0; *η* = 200 mV, *j* = 10 mA cm^−2^, pH 14) and very stable for the HER. In addition, the HER activity of MoB derived from repeated CV measurements, improves as the number of repeated scans increases. Moreover, the activity determined at pH 0 and pH 14 is almost equal, which seldom happens (Pt is one such catalyst^939^). The authors explain the increasing HER efficiency of MoB electrodes by progressive reductive removal of surface oxides from the surface upon HER. The increase in efficiency could, however, also be caused by the Pt transfer from the counter to the working electrode, as already discussed above. The experiments conducted by Vrubel *et al.*^938^ should therefore be reproduced/verified and all electrochemical experiments should be carried out in accordance with established protocols of best electrochemical practice.^1744^

Commercially available powders of TiB_2_, WB and ZrB_2_ as prospective hydrogen evolution electrocatalysts in 0.1 M sulphuric acid have been evaluated by Wirth *et al.*^940^ The HER activity (derived from Tafel measurements) was rather mediocre with overpotentials of 800 mV required for *j* = 20 mA cm^−2^.

**Fig. 55:**
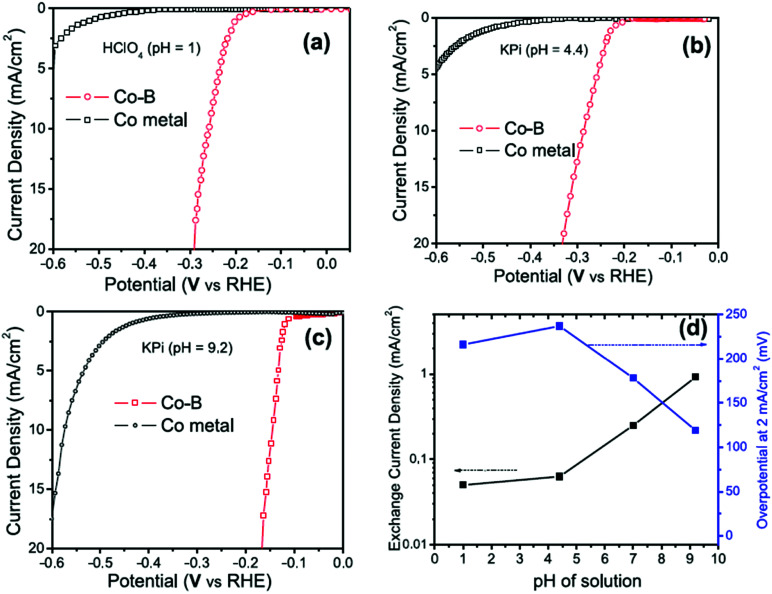
Linear polarisation curves with iR correction for CoB catalyst compared with Co metal in (a) pH 1 (0.1 M HClO_4_), (b) pH 4.4 (0.5 M KH_2_PO_4_) and (c) pH 9.2 (0.4 M K_2_HPO_4_) obtained with scan rate of 10 mV s^−1^. (d) Plot of overpotential (at 2 mA cm^−2^) and exchange current density values as a function of pH values of the solution used to test the CoB catalyst. Reproduced with permission from ref. 941. Copyright Wiley 2019.

Powder consisting of amorphous CoB nanoparticles generated through a precipitation route was pressed to obtain pellets that have directly been used as HER electrodes.^941^ Highly active Co sites are, created by electronic transfer from B to Co obviously responsible for the very good HER activity and the robustness of the electrode at pH 1, 4.4 and 9.2 (Fig. 55). The catalyst performed best at pH 9.2 resulting in *j* = 20 mA cm^−2^ current density at an overpotential of *η* = 170 mV.

Amorphous cobalt boride with a stoichiometry close to 1.9 : 1 (Co_1.9_B) was synthesised *via* reduction of CoCl_2_ with NaBH_4_ in aqueous solution by Masa *et al.*^942^ The heat-treated material exhibited reasonable activity for HER activity: *j* = 30 mA cm^−2^ at *η* = 300 mV in 1 M KOH.

Three different categories of boride-based materials have been investigated in the last 4 years as highly active compounds for electrocatalysis of hydrogen evolution: (i) hybrid materials with binary metal borides;^943,944^ (ii) ternary^945^-, quaternary^946,947^ metal borides; (iii) noble metal borides.^948,949^

**Fig. 56:**
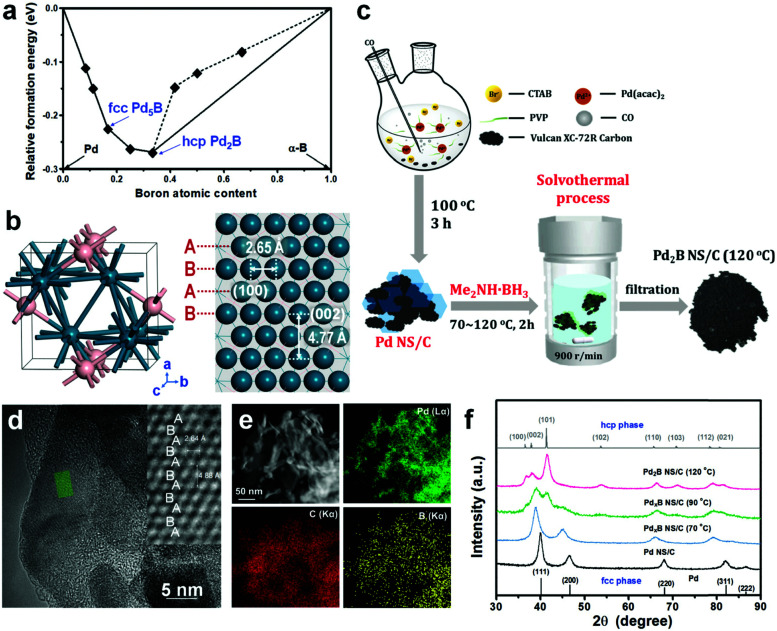
(a and b) DFT results for Pd–B alloy formation energy convex hull and hcp Pd2B crystal.22 (c) Schematic representation of the synthetic route for Pd_2_B NS/C. (d) HRTEM image of Pd_2_B NS. The inset is the magnified image of the rectangular region in (d). (e) STEM image and STEM-EDS element mapping Pd_2_B. (f) XRD patterns illustrating the phase transformation from Pd (fcc) to Pd_2_B (hcp) at different reaction temperatures. Reproduced with permission from ref. 948. Copyright RSC 2019.

From the point-of-view of HER efficiency (determined in acidic regime) the most convincing results were achieved with Pd_2_B.^948^ Pd_2_B nanosheets supported on carbon were synthesised *via* a two-step sol–gel/solvothermal approach using Pd(ii) acetylacetonate as Pd precursor (Fig. 56). Pd–B forms a stable alloy; hcp phase is the thermodynamically most favored structure with B in the octahedral sites of the Pd lattice (Fig. 56a and b) and is reached at 120 °C (Fig. 56f); with only 15.3 mV overpotential at *j* = 10 mA cm^−2^ Pd_2_B even surpassed Pt/C (*η* = 30.1 mV; *j* = 10 mA cm^−2^; 0.5 M H_2_SO_4_).

**Fig. 57:**
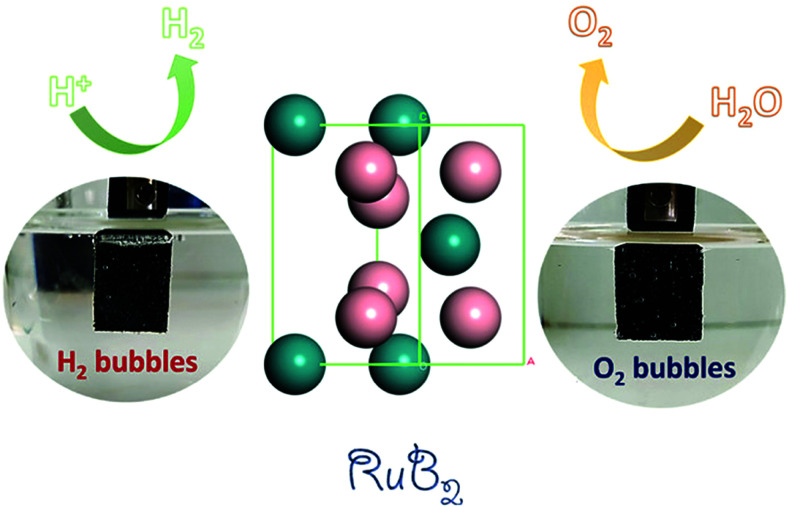
Schematic representation of the use of RuB_2_ as an anode and cathode in a water electrolysis approach. Reproduced with permission from ref. 949. Copyright American Chemical Society 2020.

Rutheniumboride (RuB_2_) was proven a good HER electrocatalyst at pH 0, too (*η* = 100 mV; *j* = 50 mA cm^−2^) and its bifunctionality (Fig. 57) allows full water splitting at a low cell voltage of 1.525 V (*j* = 10 mA cm^−2^).^949^

**Fig. 58:**
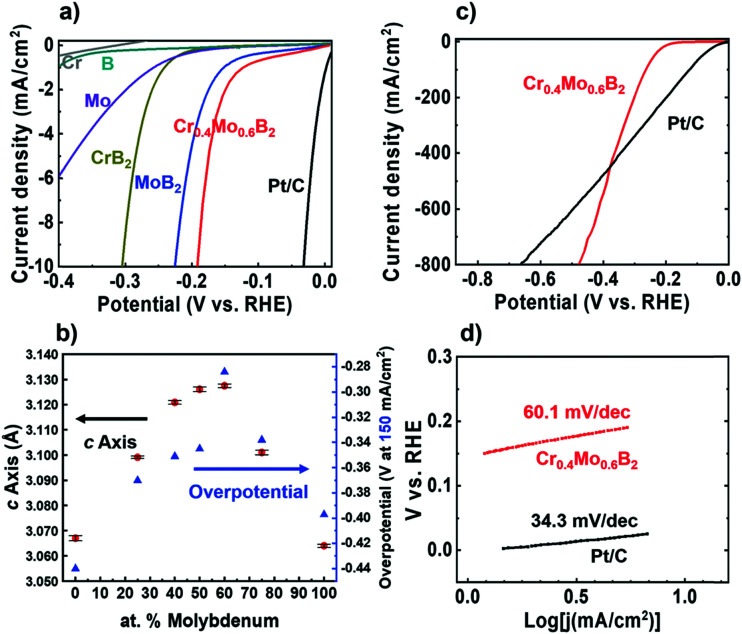
Linear sweep polarisation curves of different materials recorded in 0.5 M H_2_SO_4_ (current density normalised with the electrode's geometric surface area). (b) Plots of the lattice parameter c and the overpotential (at 150 mA cm^−2^ current density) as a function of molybdenum content. (c) Linear sweep polarisation curves showing the high current density behaviours of Cr_0.4_Mo_0.6_B_2_ and 20% Pt/C. (d) Tafel plots of Cr_0.4_Mo_0.6_B_2_ and 20% Pt/C. Reproduced with permission from ref. 945. Copyright Wiley 2020.

If high HER current densities (>100 mA cm^−2^) are sought, a ternary metal boride was shown advantageous over competitors (Fig. 58a):^945^ the HER activity of Cr_1−*x*_Mo_*x*_B_2_ (*x* = 0, 0.25, 0.4, 0.5, 0.6, 0.75) follows the same canonic-like behaviour as the *c* lattice parameter (Fig. 58b), *i.e.* the ternary representatives of the sample series showed higher HER activity (Fig. 58a), the maximum being achieved with Cr_0.4_ Mo_0.6_B_2_ (Fig. 58a, c and d). Remarkably, Cr_0.4_ Mo_0.6_B_2_ even outperformed Pt/C at high HER current densities (>500 mA cm^−2^, Fig. 58c).

Very recently quaternary borides (*e.g.*, nickel-cobalt-molybdenum-boride: Ni-CMB) were considered;^946^ nickel incorporation on Co sites being claimed to significantly increase the conductivity. Convincing alkaline HER catalytic activity was shown from long-term chronopotentiometry (*η* = 130 mV; *j* = 100 mA cm^−2^; 1.0 M KOH).

The results shown above clearly demonstrate that borides can be amongst the best HER electrocatalysts in terms of both activity and durability.

#### 7.5.2 Metal-carbides used as electrocatalysts for supporting the hydrogen evolution reaction

The anodic oxidation of hydrogen (HOR) on tungsten carbide in acidic solution was observed by Böhm *et al.* already more than 50 years ago.^950^ The electronic density-of-states of tungsten carbide near the Fermi level is closer to that of platinum than of tungsten:^951^ carbides were said to have a platinum-like behaviour.^952–954^ Numerous publications have appeared confirming the ability of various transition metal carbides to act as catalysts for the heterogeneous catalysis of a wide variety of reactions.^955–960^ To the authors' knowledge, HER electrocatalysis on carbides was first investigated in 1975 by Sokolsky *et al.*^955^ who carried out polarisation measurements in 1.0 N acids (H_3_PO_4_, HCl, H_2_SO_4_): a HER overpotential of 250 mV was measured for *j* = 1.8 mA cm^−2^. Over works followed for tungsten carbide electrodes^961–965^ but either they lack a detailed examination of the electrode composition or an identification of the catalytically-active phase^961^ or the basic results were later not reproduced by others.^966^ In addition, some investigated materials (SiC, TiC, B_4_C, Mo_2_C, NbC, TaC, VC, Ni_3_C, Co_3_C) show a HER activity that is not competitive with precious metals^940,963,967–971^ or carbides are used in composites and do not present the catalytic active phase.^964,965,972–975^ The present literature shows that the research now concentrates somewhat on tungsten—^966,976,977^ and molybdenum carbide.^978–980,1348^

**Fig. 59:**
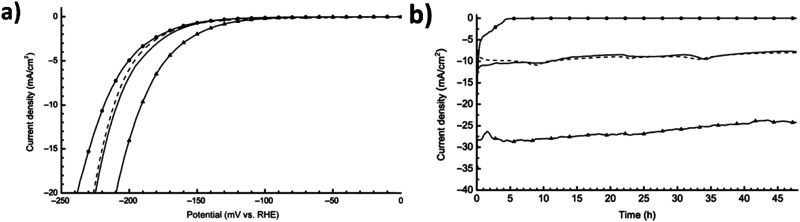
(a) Polarisation curves (10th) of MoB and Mo_2_C at pH 0 and 14. Scan rate = 1 mV s^−1^. MoB, pH 0, 2.5 mg cm^−2^ (- - - -); MoB, pH 14, 2.3 mg cm^−2^ (—●—); Mo_2_C, pH 0, 1.4 mg cm^−2^ (—); Mo_2_C, pH 14, 0.8 mg cm^−2^ (—▲—). The iR drop was corrected. (b) Time dependence of catalytic currents during electrolysis over 48 h for MoB and Mo_2_C at pH 0 and 14. The iR drop was corrected. MoB, pH 0, −195 mV (- - - -); MoB, pH 14, −200 mV (—●—); Mo_2_C, pH 0, −195 mV (—); Mo_2_C, pH 14, 0.8 mg cm^−2^ (—▲—). Reproduced with permission from ref. 938. Copyright Wiley 2012.

The HER electrocatalysis *via* carbides was later on taken up by Harnisch *et al.*^966^ A mixture of different tungsten carbide species WC and W_2_C together with W and WO_2_ has been synthesised *via* reductive carburisation.^981^ It turned out that WC was the part of the mixture with the highest HER activity (*η* = 400 mV; *j* = 30 mA cm^−2^; pH 7). In addition to MoB,

In addition to MoB, Vrubel's report also includes the corresponding carbide (Mo_2_C).^938^ Whereas in acidic (pH 0) MoB and Mo_2_C exhibited the same HER activity (Fig. 59a) with an overpotential of 195 mV for *j* = 10 mA cm^−2^ (Fig. 59b), in base (pH 14), Mo_2_C was significantly more efficient than MoB (*η* = 160 mV; *j* = 27 mA cm^−2^; pH 14).

**Fig. 60:**
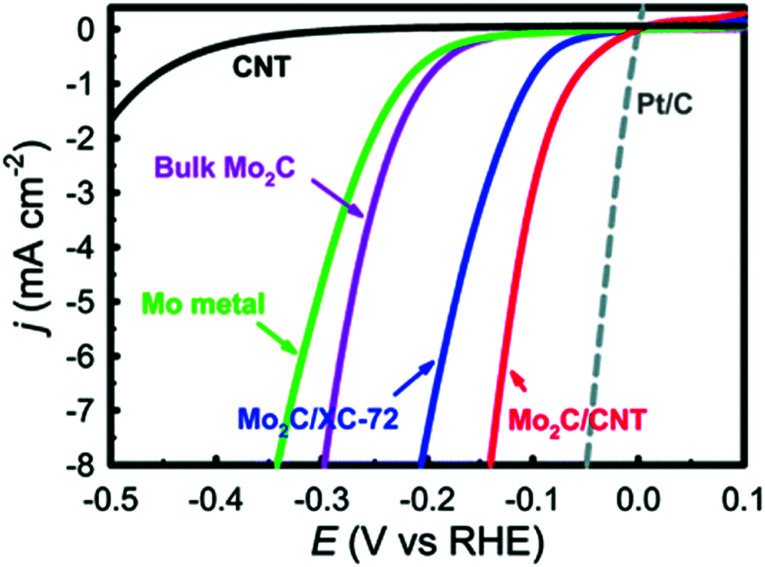
The polarisation curves of nanostructured Mo_2_C/CNT, Mo_2_C/XC, bulk Mo_2_C, Mo metal, Pt/C and CNT in 0.1 M HClO_4_. Reproduced with permission from ref. 1348. Copyright RSC 2013.

Adzic *et al.* investigated molybdenum carbide (β-Mo_2_C) nanoparticles supported either by carbon nanotubes (CNT) or carbon black (XC-72).^1348^ Mo_2_C/CNT performs best in the HER in 0.1 M HClO_4_ within the sample series (*η* = 63 mV; *j* = 1 mA cm^−2^), followed by Mo_2_C/XC-72 and Mo_2_C and Mo metal (Fig. 60).

Mo_2_C nanoparticles stabilised by a carbon layer on reduced graphene oxide (RGO) sheets turned out to be a durable HER electrocatalyst (*η* = 140 mV; 13.8 mA cm^−2^; 0.5 M H_2_SO_4_).^982^ A comparable HER activity determined in 0.5 M sulphuric acid was obtained by Girault *et al.* for Mo_2_C nanowires.^983^ Nanosised Mo_2_C is also accessible *via* a reactive template route based on C_3_N_4_ however pure carbides (nitrogen free) require high decomposition temperatures (>1500 K).^984^

**Fig. 61:**
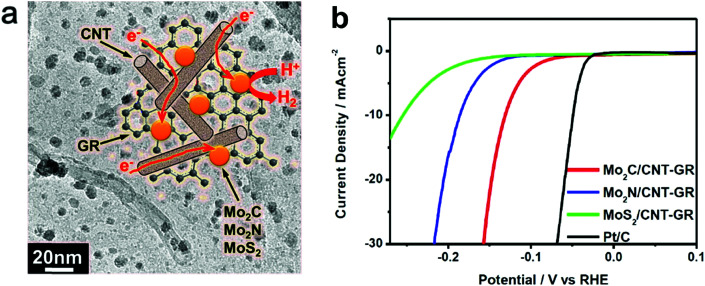
(a) Schematic illustration of Mo_2_C-, Mo_2_N-, and MoS_2_-nanoparticles anchored on carbon nanotubes (in turn) attached to graphene and the corresponding discharging of H^+^ ions leading to HER. (b) Polarisation curves derived from the nanoparticle-CNT-graphene hybrid electrocatalyst. Measurements were performed in 0.5 M H_2_SO_4_. Reproduced with permission from ref. 985. Copyright American Chemical Society 2014.

Youn *et al.* investigated Mo_2_C, MoS_2_ and Mo_2_N nanoparticles anchored on carbon nanotube (CNT)-graphene hybrid support and found that the carbide-type hybrid is the best HER catalyst (Fig. 61a and b).^985^

Besides beta phase Mo_2_C (Fe_2_N structure) the synthesis and catalytic testing of three other phases (α-MoC_1−*x*_, η-MoC and γ-MoC) was shown by Leonard *et al.*^986^ γ-MoC was the most stable for acidic HER. However, as confirmed by other researchers, β-Mo_2_C has the highest HER activity Fig. 61b.

In order to further improve the overall HER properties, metal ion doping of metal carbides was attempted, producing, for example, ternary metal carbides. Hybrid materials with a more complex architecture, which include (binary) metal carbides, were also generated as potential HER electrocatalysts.^987–1001^

Due to the large number of publications in this area, we have selected three recently-published articles which, regardless of the number of citations they received, guarantee a certain variety in terms of novelty, catalytic activity, synthesis strategy, design criteria and the type of material examined.

**Fig. 62:**
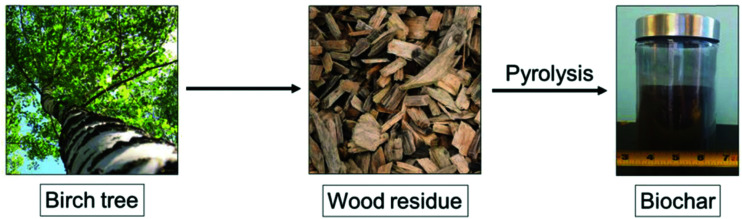
Schematic representation of biochar formation. Reproduced with permission from ref. 1002. Copyright Wiley 2018.

A catalyst made from renewable raw materials fulfill sustainability criteria in a perfect way. Humagain *et al.* recently reported on porous Mo_2_C HER electrocatalyst, synthesised using forestry residue biochar as a carbon source^1002^ (Fig. 62).

**Fig. 63:**
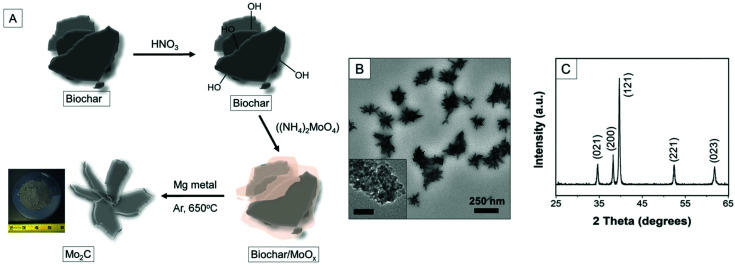
(A) Schematic representation of synthesis of Mo_2_C nanostructures. (B) TEM image (inset: scale bar = 25 nm) and (C) the powder XRD pattern of Mo_2_C derived from biochar. Reproduced with permission from ref. 1002. Copyright Wiley 2018.

Reduction of a Mo/biochar composite by Mg at 650 °C followed by purification steps resulted in β-Mo_2_C (Fig. 63). This catalyst material designed from regrowable resources turned out to highly actively and stably supporting HER in 0.5 M H_2_SO_4_ (*η* = 35 and 60 mV, *j* = 10 mA cm^−2^, 100 mA cm^−2^ respectively).

**Fig. 64:**
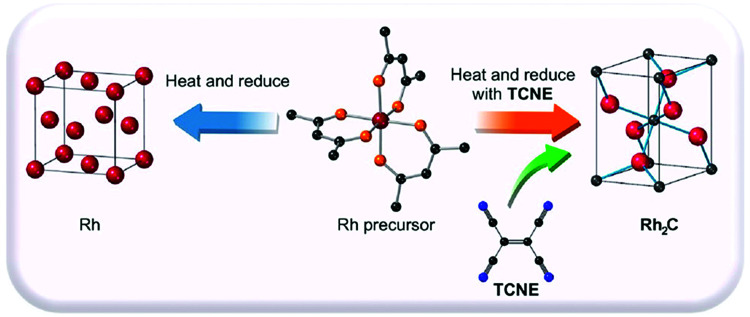
Schematic presentation of the synthesis of Rh_2_C. Reproduced with permission from ref. 1003. Copyright American Chemical Society 2020.

**Fig. 65:**
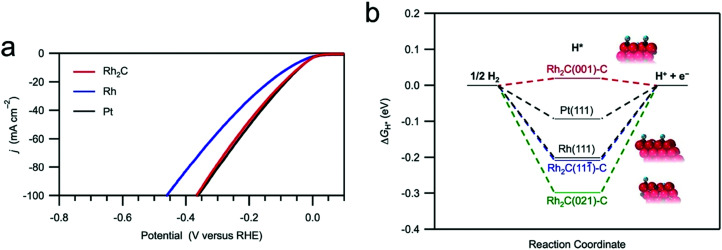
(a) The HER polarisation curves of 20 wt% Rh_2_C/C (red), Pt/C (black), and Rh/C (blue). The data were recorded in a 1.0 M KOH electrolyte with a scan rate of 50 mV s^−1^. (b) The 3-state free energy diagram for HER. The structural models show the OH* pre-covered surfaces used for calculations. The blue spheres are hydrogen atoms, and the gray spheres are oxygen atoms. Reproduced with permission from ref. 1003. Copyright American Chemical Society 2020.

Precious metal carbides do not appear in the precious metal-carbon phase diagrams and a sensible synthetic method to generate precious metal carbides has not been established until recently. Two years ago, rhodium carbide (Rh_2_C) was synthesised through a sol–gel synthesis route at high temperatures using Rh(iii) acetylacetonate as metal precursor and tetracyanoethylene (TCNE)^1003^ (Fig. 64 and 65). Fig. 65b shows the free energy diagram of H* on OH*pre-covered surfaces. The catalyst-H adduct shows a free energy close to zero and such species are regarded as highly active towards promoting the HER. Indeed, Rh_2_C exhibits a comparable HER activity to Pt (Fig. 65a).

**Fig. 66:**
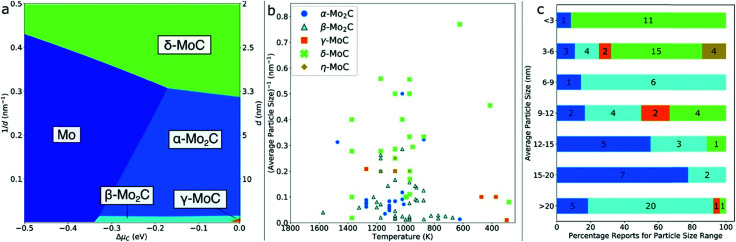
(a) Lowest energy 2-D phase diagram by projecting 3-D diagram onto Δ*μ*_C_ − 1/*d* axis. (b) Inverse average particle size *vs.* temperature for experimental reports. For clarity, plasma-based syntheses were omitted from Fig. 66b. (c) Stacked bar graph for percentage of experimental reports at given average particle sizes. Reproduced with permission from ref. 1005. Copyright American Chemical Society 2020.

Although nano-scaled transition metal carbides have become an emerging class of HER active materials,^1004^ conventional phase diagrams fail to precisely describe the phase stability of nanocrystalline materials. DFT calculations were used to determine the volume and surface energies for known Mo and W carbide phases, and the results of all efforts were combined by creating particle size-dependent phase diagrams^1005^ (Fig. 66a): bigger particles are more likely to end up in β-Mo_2_C and γ-MoC. Fig. 66b presents the inverse average particle size *vs.* (synthesis) temperature diagram derived from experimental reports. Generally high synthesis temperature will lead to bigger particles; bulk material is very often achieved through high-temperature solid-state reactions and if (*T* > 600 K) more frequently led to beta phased Mo_2_C or reports dedicated to bigger particles are more frequently based on beta-phased material. The theoretical predictions (Fig. 66a) are confirmed by the experimental findings (Fig. 66b and c).

A huge number of scientific papers are dedicated to studies that deal with HER on metal-carbide-based compounds: more than 2000 articles can be assigned to this content (ISI-Thomson-Reuters). The content of this work can be aptly summarised with the statement that the catalytic activity of metals can be increased by alloying with C and is on par with that of the reference material Pt or platinum on carbon (Pt/C).

#### 7.5.3 Metal pnictides used as electrocatalysts to support the hydrogen evolution reaction

To the best of the authors' knowledge, there is only one report dealing with the potential use of metal arsenides as cathodes for water electrolysis.^1006^ However, neither the activity nor the durability towards HER electrocatalysis in 0.5 M sulphuric acid was convincing with overpotentials exceeding *η* = 300 mV for *j* = 20 mA cm^−2^. Among the metal pnictides only nitrides and phosphides have received much attention as cathode materials in water electrolysis experiments and we will therefore focus on discussing metal nitrides (Section 7.5.3.1) and metal phosphides (Section 7.5.3.2).

##### 7.5.3.1 Metal nitrides as electro catalysts to support the hydrogen evolution reaction

All readers of this article have certainly seen at least one representative of the transition metal nitride, less in a laboratory than in a hardware store: the gold-coloured TiN coated twist drills. Transition metal nitrides are accessible by high-temperature metals nitriding with N_2_,^940^ by nitriding metal precursors with ammonia^1007,1008^ (less often) by high-frequency plasma treatment in N_2_,^1009^*via* high vacuum-supported approaches like chemical vapor deposition,^1009^*via* high vacuum supported approaches like chemical vapor deposition^1010,1011^ or *via* physical vapor deposition directly from the metal in nitrogen atmosphere upon reactive sputtering.^1012,1013^ The high-temperature approaches to some extent suffer from O and C contaminations, which may affect the catalytic properties.

Similar to carbides, formatting early transition-metal nitrides modifies the nature of the d-band of the base metals and leads to different catalytic properties than for the parent metals (that more closely resemble those of Group VIII noble metals).^918,1014^ In addition, the electric conductivity of transition metal nitrides is in the metallic range. In general, early transition metal nitrides exhibit excellent activities for catalysing diverse reactions:^1015,1016^ for example, molybdenum nitride acts near-similarly to platinum for hydrocarbons hydrogenolysis. Although already used 15 years ago for the photocatalytically-initiated hydrogen evolution,^11,1017,1018^ metal nitrides were only used for the electrocatalytical HER since 2011.^1347^ We already tried to reasonably explain why metal oxides are more sensitive to negative electrode potentials than sulphides, with the consequence that oxides are used more as OER electrode materials than as HER electrode materials. This could also explain why some metal nitrides (CoN,^1026^ Co_4_N,^1019^ Fe_3_N/Fe_4_N^1020^) have amazing activity for the OER rather than for the HER (N is electronegative). Recently, several reviews have been published that deal with transition metal nitrides as potential electrode material for water electrolysis purposes.^1021–1025^

Among the binary metal nitrides molybdenumnitride stands out in some ways as this type of material has been more intensively investigated as a potential HER electrocatalyst.^1026–1029^

The HER activity of carbon-supported Nickel-Molybdenum nitride (NiMo_4.7_N_*x*_/C) was determined in 0.1 M HClO_4_ and found adequate (*η* = 200 mV; *j* = 3.5 mA cm^−2^) but not competitive with current HER electrocatalysts, *e.g.*, of the carbide family.^1347^ Wirth *et al.* investigated besides borides, carbides, sulphides and carbonitrides a series of transition metal nitrides (AlN, Ta_3_N_5_, TiN) derived from industrial manufacturing routes as potential HER electrodes in 0.1 M sulphuric acid;^940^ the nitrides did not prove show active (*η* = 763 mV (Ta_3_N_5_)–973 mV (AlN); *j* = 20 mA cm^−2^.

**Fig. 67:**
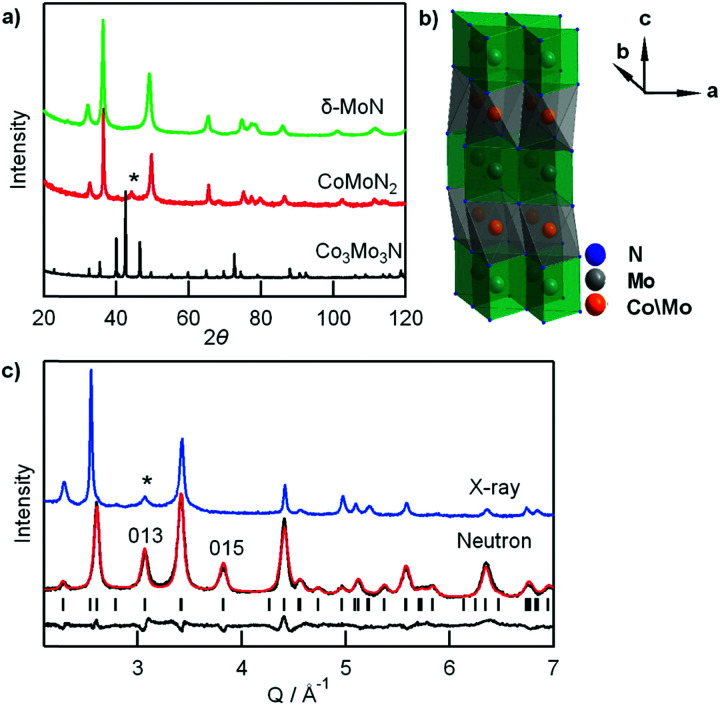
(a) Lab X-ray powder diffraction patterns of Co_3_Mo_3_N, CoMoN_2_, and δ-MoN. Asterisk marks the impurity peak of cobalt metal. (b) Four-layered crystal structure of CoMoN_2_. (c) Rietveld refinements of neutron diffraction for CoMoN_2_ showing observed data (black line), calculated pattern (red line) and difference curve (bottom line). Lab X-ray diffraction data (blue line) in same *Q* (= 2π/d) range between 2 and 7 Å^−1^ do not clearly show superstructure peaks such as the 013 and 015 reflections which are intense in neutron diffraction data. Reproduced with permission from ref. 1030. Copyright American Chemical Society 2013.

Khlaifah and co-workers achieved a breakthrough with respect to the development of transition metal-based nitrides with at least reasonable HER catalytic activity.^1030^ Cobalt molybdenum nitride (Co_0.6_Mo_1.4_N_2_) exhibited, as revealed from neutron powder diffraction data, a layered structure with alternating layers of trigonal prismatic and octahedral coordination for the Mo and Co (Fig. 67) and was found to reasonably catalyse the HER (*η* = 250 mV; *j* = 10 mA cm^−2^; 0.1 M HClO_4_).

**Fig. 68:**
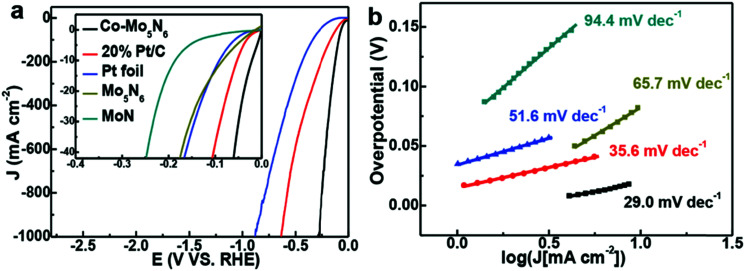
Electrochemical measurements. (a) HER polarisation curves of the samples in 1.0 M KOH. (b) Corresponding Tafel plots. Reproduced with permission from ref. 1031. Copyright Wiley 2020.

Co-Mo_5_N_6_, at first sight a similar-composed material, turned out to be a composite with coexisting metallic cobalt and a nitrogen-rich molybdenum nitride phase.^1031^ It's HER performance is among the best ever published (*η* = 19 mV; *j* = 10 mA cm^−2^; Tafel slope = 29.0 mV dec^−1^; 1.0 M KOH, Fig. 68).

In general, there is no deep evidence that nitrogen atoms act as the HER active sites; one rather assumes that N centers in nitrides are simple spectators.^1014^ To more easily study HER mechanism, some scientists rather did a U-turn and investigated (again) binary metal nitrides.^1032^

The first work that reports on molybdenum nitride that has proven good characteristics for HER catalysis appeared in 2014.^1032^ Atomically thin molybdenum nitride (MoN) nanosheets prepared by liquid exfoliation of the bulk material in *N*-methyl-pyrrolidone (NMP) *via* ultrasonication. Apical Mo atoms on the surface of the nanosheets present the catalytic active sites which feeds the assumption that through nitriding Mo behaves like precious metals. However, from a performance standpoint, MoN (*η* = 300 mV; *j* = 37 mA cm^−2^; 0.5 M H_2_SO_4_) is still a long way from platinum. Youn *et al.*^985^ and Ma *et al.*^1033^ confirmed that in direct comparison with carbides, nitrides of the same family (Mo_2_C, Mo_2_N) cannot keep up in terms of efficiency.

Substantially better HER performance was shown by Shalom *et al.* for Ni_3_N grown on Ni foam^1034^ (*η* = 500 mV; *j* = 100 mA cm^−2^; 1 M KOH). The capability of Ni_3_N to efficiently promote HER was later confirmed by different groups.^1035–1037^

**Fig. 69:**
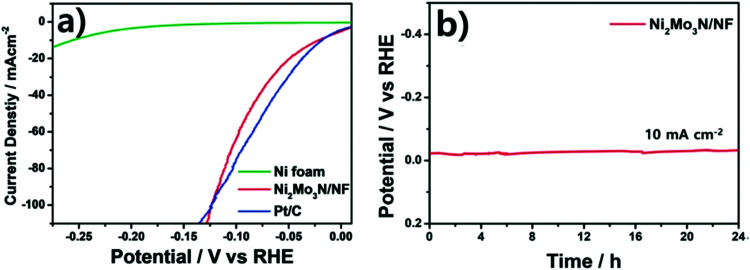
Electrochemical characterisation for the prepared catalysts. (a) Polarisation curves. (b) Stability measurement. Reproduced with permission from ref. 1042. Copyright RSC 2021.

Bimetallic nickel-based nitrides, *i.e.*, ternary metal nitrides comprising nickel were found slightly more efficient compared to binary nickel nitride species:^866,1038–1043^ Ni_3_FeN nanoparticles^866^ are on par with Pt/C, in particular at high current densities (*η* = 300 mV, *j* = 100 mA cm^−2^), and similar for Ni_2_Mo_3_N nanoparticles grown on nickel foam (Fig. 69).^1042^

**Fig. 70:**
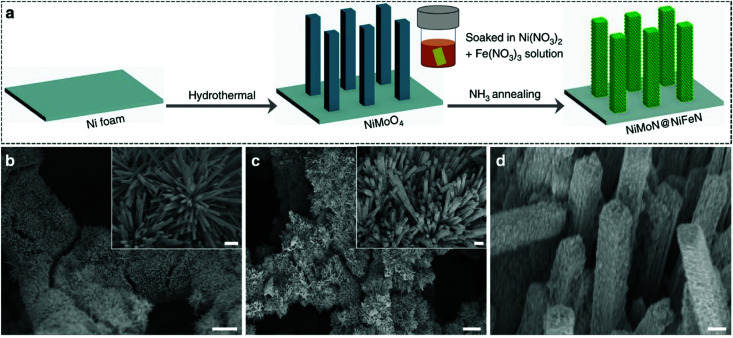
Synthesis and microscopic characterisation of the as-prepared NiMoN@NiFeN catalyst. (a) Schematic illustration of the synthesis procedures for the self-supported 3D core–shell NiMoN@NiFeN catalyst. (b–d) SEM images of (b) NiMoN and (c and d) NiMoN@NiFeN at different magnifications. Reproduced with permission from ref. 1044 Copyright Nature Publishing 2019.

A very consistent implementation of the strategy to use bimetallic nitrides was recently shown by Yu *et al.*^1044^ NiFeN core NiMoN shell-architectured nanoparticles (NiMoN@NiFeN) as well as NiMoN core only particles (Fig. 70) were described as being even more efficient than Pt/C in HER experiments in 1 M KOH: *η* = 127 mV for *j* = 500 mA cm^−2^.

Another way that appears promising for the production of highly active transition metal nitrides for HER electrocatalysis is to choose those that contain noble elements to a certain extent.^1045^

We do not want to close this subsection until we have introduced another compound. Hexagonal boron nitride efficiently supports ORR^1046^ and has considerable hydrogen adsorption capability.^1047^ In fact, it was demonstrated by Uosaki *et al.*^1048^ that HER proceeds very efficiently on a nanosheet of hexagonal boron nitride (BNNS) on gold substrate.

As a summary the HER performance of most of the nitride-based electrocatalysts discussed so far is slightly below that of typical representatives of the boride or carbide type. However, some of the bimetallic nitrides and intelligently structured composites, including transition metal nitrides, in particular, have HER activities that are definitely among the best performances ever identified. Some publications claim that the metal centers act as catalytically active centers; however, solid confirmation of this hypothesis has not yet been published, making further optimisation difficult.

##### 7.5.3.2 Metal phosphides as electro catalysts to support the hydrogen evolution reaction

Due to the enormous number of articles published on this topic, concision is awkward and can only be achieved by focusing on the pioneering work, and on groundbreaking results (heavily cited) results. Additional literature is also accessible, which summarises the collected results very well in the form of review articles,.^58,1049–1053^

Several decades ago, metal phosphides, at that time basically synthesised starting from highly reactive elemental phosphorus, were used in the field of metallurgy, hydrodesulphurisation, pesticides and for photocatalytic degradation.^1054,1055^ Photocatalytically-initiated hydrogen evolution on the phosphide-solution interface has been the topic of several papers published in the 1970 s.^1056,1057^

Pioneering work by Paseka and Burchardt showed that amorphous phosphides are able to promote HER in alkaline medium at low overpotentials.^1058,1059^

**Fig. 71:**
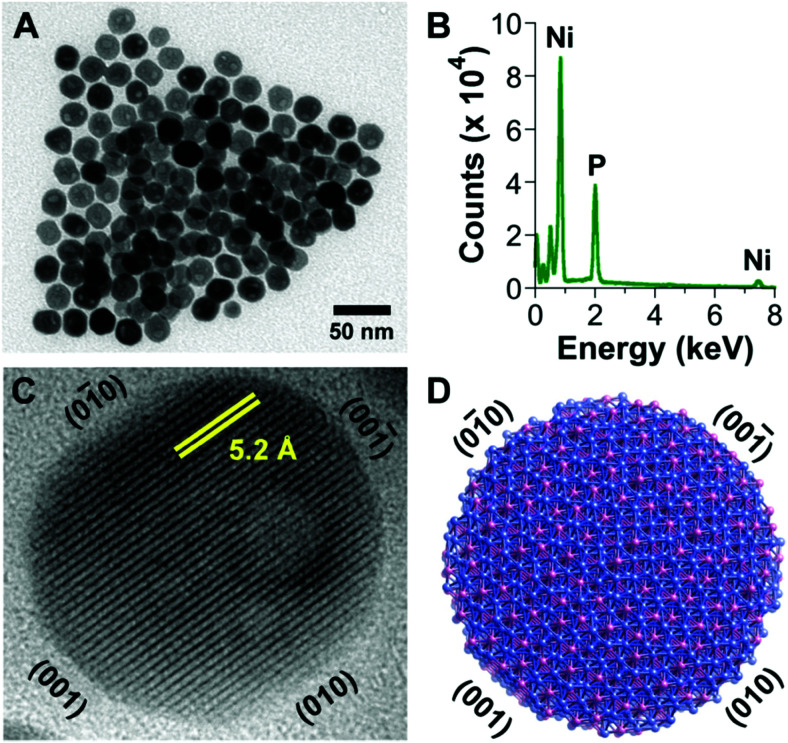
(A) TEM image and (B) EDX spectrum of Ni_2_P nanoparticles. (C) HRTEM image of a representative Ni_2_P nanoparticle, highlighting the exposed Ni_2_P(001) facet and the 5.2 Å lattice fringes that correspond to the (010) planes. (D) Proposed structural model of the Ni_2_P nanoparticles. Reproduced with permission from ref. 1259 Copyright 2013 American Chemical Society.

**Fig. 72:**
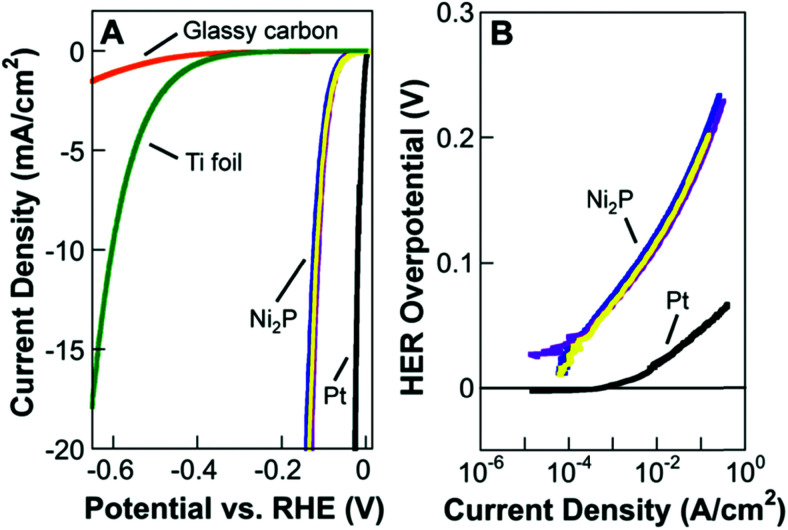
(A) Polarisation data for three individual Ni_2_P electrodes in 0.5 M H_2_SO_4_, along with glassy carbon, Ti foil, and Pt in 0.5 M H_2_SO_4_, for comparison. (B) Corresponding Tafel plots for the Ni_2_P and Pt electrodes. Reproduced with permission from ref. 1259 Copyright 2013 American Chemical Society.

Rodriguez and co-workers proposed that the (001) surface of Ni_2_P combines the favourable H-bonding of the hydrogenase systems with the thermal stability of a heterogeneous catalyst.^1060^ Other researchers noticed the potential mechanistic analogy between hydrodesulphurisation (HDS), the catalytic process by which sulphur impurities are removed from hydrocarbon fuels, and HER. Ni_2_P, one of the most active HDS catalysts,^1061^ should therefore be a promising HER electrocatalyst which was experimentally confirmed by the groups of Lewis and Schaak.^1259^ Ni_2_P nanoparticles (Fig. 71) highly actively and durably supports HER in 0.5 M H_2_SO_4_ (Fig. 72): *η* = 130 mV for *j* = 20 mA cm^−2^. Shortly after, Hu's group confirmed the ability of Ni2P for HER catalysis.^1062^

The field of metal phosphide HER catalysts has expanded since then rapidly. A variety of binary, ternary and higher order metal phosphides with *e.g.*, high-symmetry ionic crystal structures like NaCl type or more complex structures have been synthesised and checked for their electrocatalytic properties. The electrical conductivity differs depending on the composition and M-P bond (ionic, metallic or covalent) and ranges from semiconducting to metallic to superconducting.

Depending on the composition, the chemical properties also vary, which, for example, leads to an inertia in relation to the dissolution in acids/bases or which makes them easily soluble (Cd- or Zn phosphide) thereby affecting its suitability to act as an HER electrocatalyst. Basically Fe, Ni, Co, Cu, W and Mo phosphides were found promising cathode materials for water electrolysis. Based on DFT approaches, the P atom plays a major role for the catalytic activity of metal phosphide in metal phosphides. The ability of metal phosphides to act as proton-reducing species (to initiate HER) stands and falls with the trend of the negatively-charged P atom to donate electrons. Thus, for the same metal phosphide, increasing the percentage of P in the total number of atoms is known to enhance the HER activity (Ni_5_P_4_ > Ni_2_P)^1063^ and an increased P content leads to better corrosion resistance.^1064^ On the other hand, if P is continuously doped in metals, the conductivity decreases.^926^

Metal phosphides have been prepared in various forms (bulk, single crystals, films, nanoscaled solids) and, depending which form is intended, are accessible *via* various synthesis routes comprising solid state reaction of the elements and red phosphorous at high temperature,^1065^ solid state reaction of reactive metal phosphides with transition metals,^1066^ phosphidation of metal oxides,-hydroxides or reduction of metal phosphates,^1067^ solvothermal approaches,^1068^ organometallic precursor-based routes performed in high boiling solvents^1069^ and high vacuum CVD or PVD-based techniques.^1070–1072^

Originally the HER properties of Ni2P were checked in alkaline-^1062^ and in acidic regime.^1259^ Meanwhile transition metal phosphides have also been shown to be active HER supporting catalysts under neutral pH conditions.^1073^ Several strategies have been exploited to further enhance the electrocatalytic activity of phosphide-based HER electrocatalysts. Among them (i) developing HER active compounds with hydrophilic and aerophobic surfaces; (ii) increasing the conductivity of the electrocatalyst by firmly attaching the HER active (phosphide-based compound) phase to *e.g.*, CNTs, graphite, graphene;^1074–1076^ (iii) doping metal phosphides with other metals to bimetallic phosphides;^1077^ (iiii) doping of metal phosphides with other nonmetals.^1078^ The most widely studied materials in relation to phosphide-based-electrode materials for water electrolysis include nickel phosphides (Ni_2_P;^1062,1079,1253^ Ni_5_P_4_;^1080,1081^ Ni_12_P_5_,^1082,1083^ cobalt phosphides (CoP,^1084–1088^ Co_2_P,^1064,1079,1089^ CoP_2_^1090,1091^), molybdenum phosphides (MoP,^1092,1093^ Mo_3_P,^1093^ MoP_2_^1094^), tungsten phosphides (WP,^1095,1096^ WP_2_^1097,1098^), iron phosphides (FeP,^1099–1104^ Fe_2_P,^1079,1105^ FeP2^1100,1106^). The best results collected up to 2016 for binary nickelphosphides for HER electrocatalysis in 0.5 M H_2_SO_4_ were achieved with Ni_5_P_4_ (*η* = 23 mV; *j* = 10 mA cm^−2^),^1080^ (*η* = 62 mV; *j* = 20 mA cm^−2^).^1081^ CoP showed the best HER efficiencies that were achieved with the help of binary cobalt phosphides (*η* = 48 mV; 10 mA cm^−2^),^1087^ (*η* = 59 mV; 20 mA cm^−2^) in the same electrolyte. Among the binary iron phosphides FeP turned out to be superior to Fe_2_P or FeP_2_ based HER electrocatalysts (*η* = 34 mV; *j* = 10 mA cm^−2^ and *η* = 43 mV; *j* = 20 mA cm^−2^).^1104^ A much lower HER efficiency was obtained when acidic HER was catalysed by binary molybdenum, tungsten- or copper^1107^ phosphides. Among this type of electrocatalysts, MoP exhibited the best results in 0.5 M H_2_SO_4_ (*η* = 90 mV; *j* = 10 mA cm^−2^ and *η* = 105 mV; *j* = 20 mA cm^−2^).^1092^

It can generally be said that binary transition metal phosphides perform worse for alkaline HER than in acids: at pH 14, FeP^1108^ and CoP^1073^ required overpotentials in the 200 mV range for *j* = 10 mA cm^−2^. Metal doping, *i.e.*, conversion of binary metal phosphides to ternary- or quaternary metal phosphides, was found efficient to increase the metal phosphide efficiency for alkaline HER electrocatalysis: (Ni_0.33_Fe _0.67_)_2_P leads to *η* = 214 mV at *j* = 50 mA cm^−2^ in 1 M KOH.^1109^ HER electrocatalysis at pH 14 upon the ternary phosphide MoCoP was actively and durably promoted as well (*η* = 40 mV; *j* = 10 mA cm^−2^).^1110^ One of the best HER performances determined for HER electrocatalysis in alkaline medium upon phosphides was achieved with WniCoP: *η* = 30 mV for *j* = 10 mA cm^−2^.^1111^

Composites with coexisting phases that differ in terms of their chemical nature and that are in close contact present unique interfacial interactions.^1112^

**Fig. 73:**
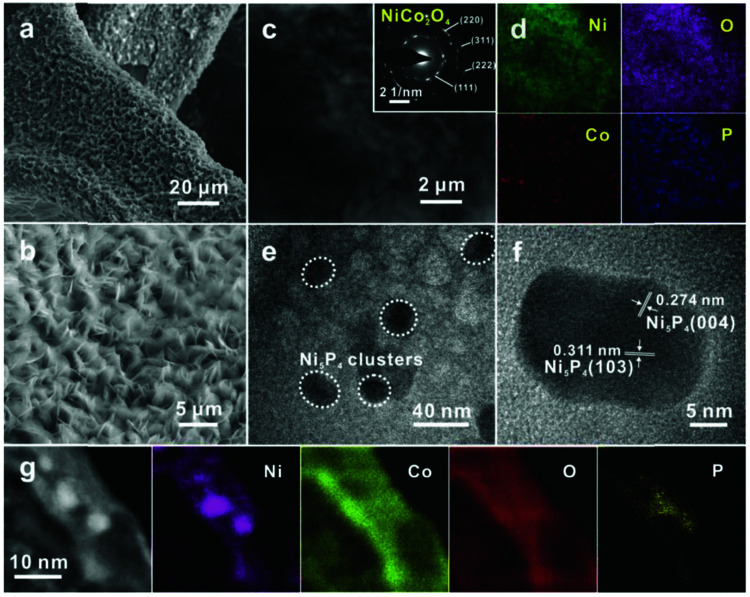
(a) SEM image showing the uniformly distributed Ni_5_P_4_@NiCo_2_O_4_ nanoflakes on graphene/Ni foam. (b) High-magnification SEM image of Ni_5_P_4_@NiCo_2_O_4_ nanoflakes. (c) Low-magnification TEM image and (d) corresponding energy dispersive spectroscopy (EDS) elemental mapping images of Ni_5_P_4_@NiCo_2_O_4_ nanoflakes. (e) High-resolution TEM image showing that nanometric Ni_5_P_4_ clusters are nested on the nanoflakes. (f) HRTEM image of one single Ni_5_P_4_ nanocluster. (g) HRTEM image and corresponding elemental mapping images of Ni_5_P_4_@NiCo_2_O_4_. Reproduced with permission from ref. 1112 Copyright Wiley 2018.

In 2018 Zhang *et al.* reported on Ni_5_P_4_ nested on NiCo_2_O_4_ (Ni_5_P_4_@NiCo_2_O_4_, Fig. 73) as a heterogeneous structured HER electrocatalyst generated by phosphating of NiO firmly attached to NiCo_2_O_4_: Ni_5_P_4_@NiCo_2_O_4_ exhibited very good HER activity (*η* = 27 mV for *j* = 10 mA cm^−2^; 1.0 M KOH).

In 2020 and 2021, more than 1000 articles were found with the search terms “phosphide hydrogen evolution” (ISI web of knowledge). Extremely active phosphide-based-HER electrocatalysts were very recently developed for acidic^1113–1116^ and alkaline HER electrocatalysis.^1117–1119^

**Fig. 74:**
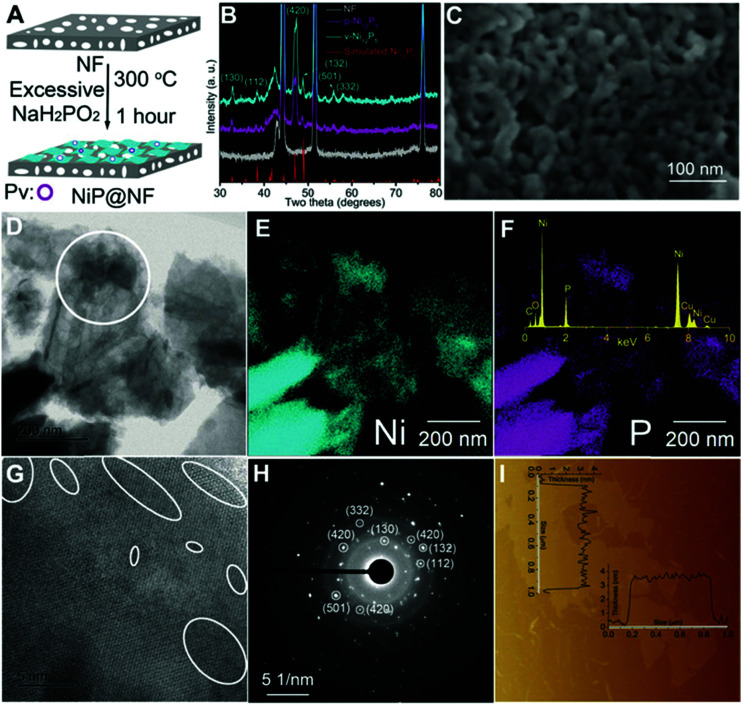
Synthesis and characterisation of catalysts. (A) Synthesis procedure. (B) XRD patterns. (C) SEM image. (D) TEM image. (E and F) TEM EDS elemental mapping images of Ni and P; inset: EDS spectrum. (G) High-resolution TEM image; white circles mark the possible Pv areas. (H) SAED of the white circle area in panel (D). (I) AFM image; insets: height distribution curves; note that (C–I) all show images/data for v-Ni_12_P_5_. Reproduced with permission from ref. 1119 Copyright Wiley 2020.

**Fig. 75:**
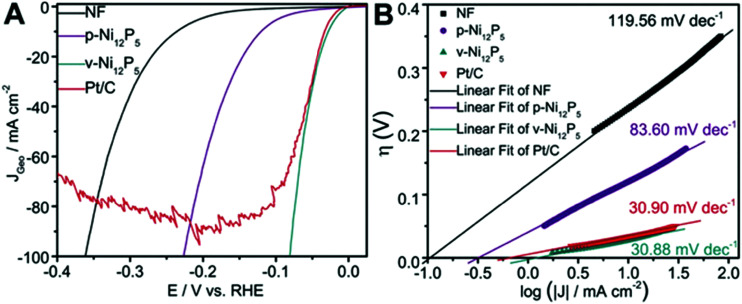
Electrochemical measurements. (A) Polarisation curves. (B) Tafel plots. Reproduced with permission from ref. 1119 Copyright Wiley 2020.

Duan *et al.* investigated the special role of phosphorous vacancies in nickel phosphide to boost the HER efficiency in alkaline solution by two orders of magnitude^1119^ (Fig. 74). Possible P-defective sites are obtained using high resolution TEM (Fig. 74g). Ni_12_P_5_ with vacancies (v-Ni_12_P_5_) outperformed non-defective Ni_12_P_5_ (p-Ni_12_P_5_) as well as Pt/C with respect to HER efficiency in polarisation measurements carried out in 1 M KOH (Fig. 75).

As a summary of Section 7.5.3.2, it can be said that the latest findings impressively confirm the outstanding efficiency of phosphide based HER electrocatalysts.

#### 7.5.4 Metal chalcogenides as electrocatalysts to support the hydrogen evolution reaction

Many review articles summarise results on chalcogenides as promising water electrolysis electrode materials,^926,1120–1126,1330^ Many transition metal chalcogenides naturally occur in Earth's crust, MoS_2_ exists as the mineral molybdenite.^1127^ Many properties are certainly worth mentioning, but with a potential use as a catalyst in particular, a specialty of the metal chalcogenides seems superficially interesting: The properties of the transition metal chalcogenides in their bulk states can significantly differ from their nanoscale counterparts, which spurred the efforts of scientists to synthesise *e.g.* two-dimensionally layered transition metal dichalcogenides (2D TMC), which up to some extent freezes the unique properties of the nanoscale material on a macroscopic scale.^1128^ The present section will focus on the pioneering works plus some groundbreaking (and heavily cited) results on the theme.

Transition metal chalcogenides are accessible through solvothermal aproaches,^1155^ electrodeposition,^1783^ high-vacuum-supported deposition methods (PVD and CVD^1129^), exfoliations (top-down approaches), *e.g.* realised through sonication^1130^ and intercalation^1131,1132^ as well as upon bottom-up strategies (*e.g.* injection of a precursor solution to a (hot) metal precursor solution (hot injection method)).^1133^

From the late 1970s onwards, transition metal chalcogenides began to be considered as HER electrode material in water electrolysis,^1134^ while use for photocatalytic water splitting began about 4 years later.^1135^

Vandenborre *et al.* published the first journal report dealing with electrocatalytic HER on pure metal chalcogenides appeared in 1984: NiS_2_ led to *j* = 30 mA cm^−2^ at around *η* = 100 mV in 1 M NaOH, which is a respectable efficiency value.^1136^

**Fig. 76:**
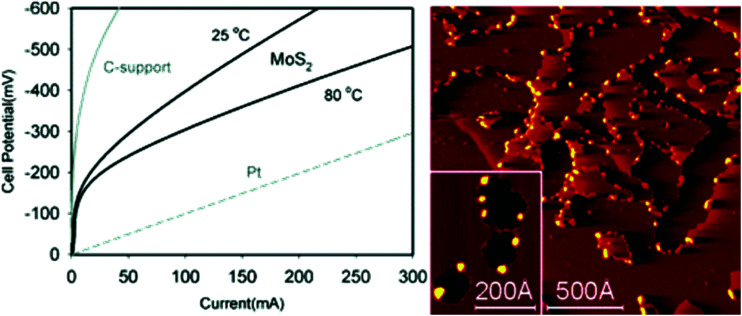
Polarisation curve for hydrogen evolution on Pt, daihope C-support, and MoS_2_ cathodes. The potentials are measured with respect to a carbon-supported Pt anode in a proton exchange membrane electrode assembly. (Right) STM images of MoS_2_ nanoparticles on modified graphite. Reproduced with permission from ref. 1137 Copyright 2005 American Chemical Society.

Little research has been undertaken in the following years on this field. This research received a kind of initial spark with the in-depth investigation of MoS_2_ material, widely used in industry for the hydrodesulphurisation of petroleum, which was identified as an efficient HER catalyst in acidic media based on the theoretical and experimental results of Nørskov and co-workers^1137^ and Chorkendorff and co-workers.^1703^ The computational results predicted that graphite-supported MoS_2_ should be a good HER electrocatalyst.^1137^ This was experimentally confirmed upon using MoS_2_ nanoparticles supported on carbon (*η*∼200 mV; *j* = 50 mA cm^−2^; Fig. 76).^1137^ MoS_2_ remained in the focus of interest of numerous researchers.^1140^

Li *et al.* showed that S-vacancies in MoS_2_ create gap states that allow favourable hydrogen adsorption.^1139^ Strained MoS_2_ with S vacancies proved to be a competitive HER electrode material *η* = 200 mV; *j* = 20 mA cm^−2^; pH 0.2). Long before appearance of this work lattice strain engineering proved to be a powerful tool to tune the electronic structure hence the electrocatalytic properties.^1152^

To date many transition sulphides, selenides and some tellurides exhibited competitive HER: MoS_2_,^1138–1140^ MoS_3_,^1141^ MoSe_2_,^1142^ CoS,^1143^ Co_9_S_8_^1144^ CoSe_2_,^1145^ Co_7_Se_8_,^1146^ NiMo_3_S_4_,^1147^ CoSe_2_-SnSe_2_,^1148^ NiS_2_,^1149^ Ni_3_S_2_,^1149^ Ni_3_Se_2_,^1150^ NiS, NiSe,^1151^ NiS_0.5_Se_0.5_,^1152^ Ni_3_ S_2_-CdS,^1153^ MoS_2_ in Cu_2_S matrix,^1154^ FeS,^1155^ FeS_2_,^1156,1157^ FeSe,^1158^ Co doped FeSe_2_^1159^ Ni_3_Bi_2_S_2_,^1160^ Bi_2_Te_3_,^1161^ MX_2_ (M = V, Nb, and Ta; *X* = S, Se, and Te),^1162^ TaS_2_,^1163^ CoTe_2_,^1164^ NiTe_2_,^1164^ MoTe_2_,^1165^ WS_2_,^1166^ WSe_2_^1167–1170^ NiCo_2_S_4_,^1455^ MoS_2_-CuS,^1171^ CuS,^1172^ Cu_2_Se,^1173^ CoTe2-CdTe.^1174^

This list shows that sulphides and selenides are the most frequently investigated metal chalcogenides for water electrolysis (only some tellurides (MoTe_2_^1165^) have been included in this investigation so far).

**Fig. 77:**
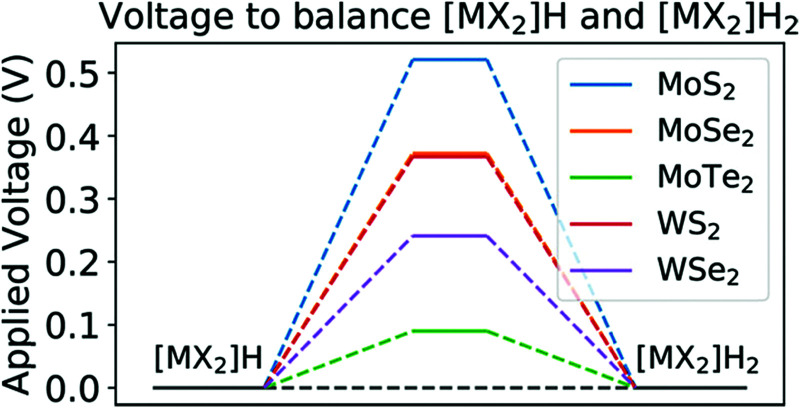
Required applied potential to obtain a zero-reaction energy for the rate determining Volmer step from [MX_2_]H to [MX_2_]H_2_. Reproduced with permission from ref. 1138 Copyright 2018 American Chemical Society.

That is rather surprising because, from a theoretical point-of-view tellurides (in general) should not be less active than the lighter homologues of the sixth main group. Huang *et al.*^1138^ compared the reaction energy (Δ*G*_H_2__) for the rate-determining Volmer step [MX_2_]H to [MoX_2_]H_2_ (for X = S–Te and M = Mo, W) and calculated the voltage required to obtain Δ*G*_H_2__ = 0 (Voltage to balance [MX_2_]H and [MoX_2_]H_2_; Fig. 77): the voltage minimum of approximately 90 mV is reached for X = Te (MoTe_2_).

Different approaches are currently employed to increase the number of active sites and to improve the electrocatalytic properties of metal chalcogenides. For instance, the optimisation of the chemical composition leads to an increase in the intrinsic electrocatalytic activity, which in turn is based on a reduction in the free hydrogen adsorption energy Δ*G*_H_ (intrinsic).^1139^ The optimisation of the structure leads to an increase in the number of catalytic active sites, hence an increase in the extrinsic catalytic activity.^1152^

A special realisation of the structure optimisation results from the creation of heterostructured systems, in which *e.g.*, identically composed compounds are in close contact with each other such as nanorods and sheets (of the same material); or different crystalline phases of materials with identical stoichiometry form a nanostructured composite. In addition, lattice strain engineering (see above) is a powerful tool for structure optimisation.

**Fig. 78:**
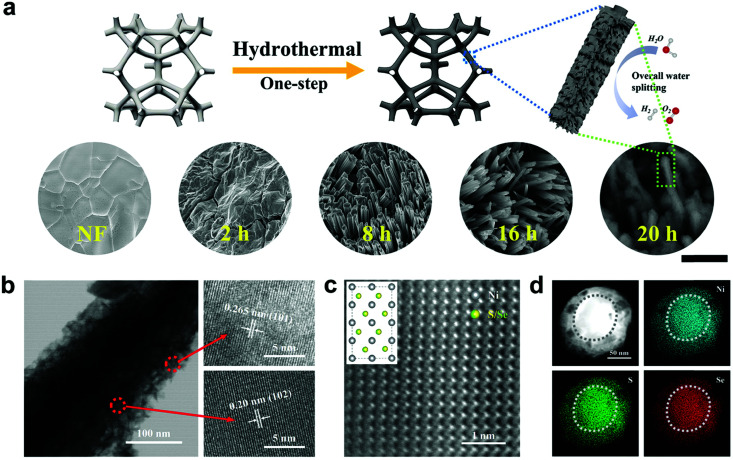
Synthesis and structural characterisations. (a) Schematic illustration of the synthetic procedures of NiS_*x*_Se_1−*x*_ nanocomposites. The scale bars for SEM images are 2 μm. (b) TEM and HRTEM images of NiS_0.5_Se_0.5_. (c) Atomic-resolution HAADF-STEM image (inset shows the corresponding schematic atom arrangement). (d) HAADF-STEM and EDS mapping images of NiS_0.5_Se_0.5_ from the cross-section view. Reproduced with permission from ref. 1152. Copyright Wiley 2020.

A series of lattice-strained homogeneous NiS_*x*_Se_1−*x*_ nanorod@nanosheet hybrid (homogeneous composed but heterostructured NiS_*x*_Se_1−*x*_) firmly attached to Ni foam have been synthesised upon a hydrothermal route (Fig. 78):^1152^ the NiS_0.5_Se_0.5_ representative with 2.7% lattice strain is ideally able to support HER + OER at overvoltages of 70 mV (HER) or 257 mV (OER) (*j* = 10 mA) cm^−2^; 1 M KOH).

A common feature of the papers published on this topic over the past two years is the significant increase in HER activity.^1175–1180^

**Fig. 79:**
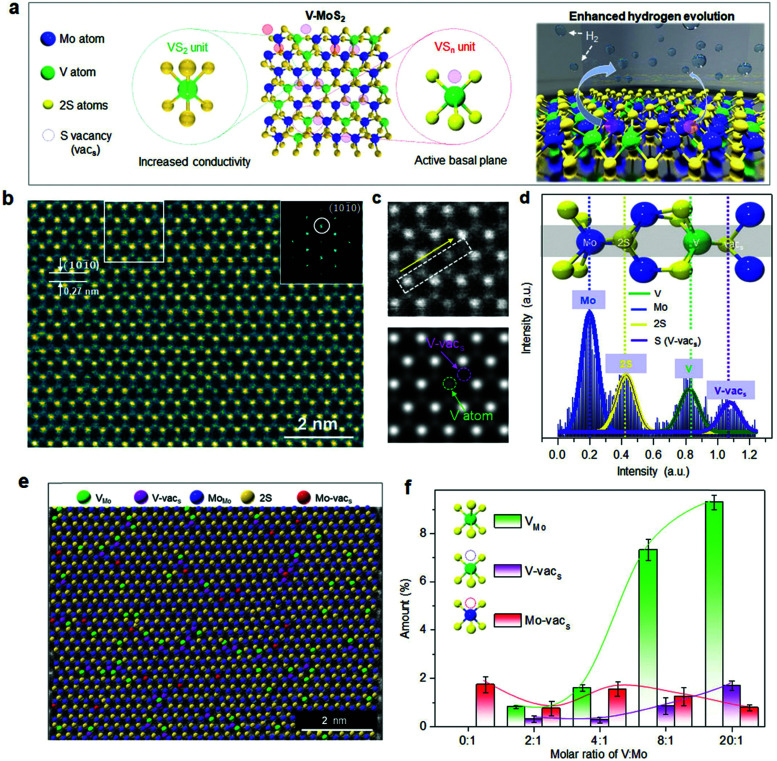
Atomic structure of monolayer V-MoS_2_. (a) Schematic of V–MoS_2_ with VS_2_ and VS_n_ units and hydrogen evolution on V–MoS_2_*via* basal-plane activation. (b) ADF-STEM image at 9.3% V concentration, indicating a d-spacing of 0.27 nm for 2H–MoS_2_ and the corresponding electron-diffraction-pattern of (101–0) plane in the inset. (c) STEM image of white square region in (b) and simulated image and (d) the corresponding intensity profile. (e) False-coloured ADF-STEM image of monolayer V-MoS_2_ with Mo-substituted V atom (V_Mo_), sulphur-vacancy next to V atom (V-vac_s_), Mo atom (Mo_Mo_), two S atoms (2S), and sulphur-vacancy next to Mo atom (Mo-vac_s_). (f) Atomic % distribution of V_Mo_, V-vac_s_, and Mo-vac_s_ as a function of molar ratio of V to Mo precursor. Statistical analysis data were obtained from false-coloured ADF-STEM images. Reproduced with permission from ref. 1178 Copyright Wiley 2021.

Metallic vanadium sulphide (VS_n_) embedded in a MoS_2_ film (to result in a V-MoS_2_ film) are highly active HER catalysts, as shown by Kim's group:^1178^ VS_n_ units are formed in the basal plane of MoS_2_ (Fig. 79), leading to an impressive HER current density of 1000 mA cm^−2^ at 600 mV overpotential during water electrolysis experiments.

**Fig. 80:**
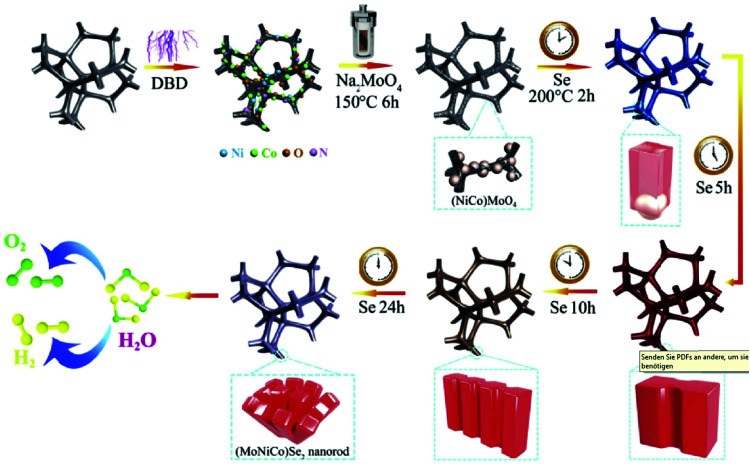
The schematic fabricating processes of MoSe_2_–NiSe_2_–CoSe_2_ nanorods on the PNCF surface. Reproduced with permission from ref. 1175 Copyright Elsevier 2020.

A Mo–Ni–Co tri-metallic selenide nanorod arrays-based HER electrode turned out to be able to achieve high current densities (0.3 A cm^−2^) at acceptable overpotentials (*η* = 350 mV) as well^1175^ (Fig. 80).

To summarise, metal chalcogenides are without a doubt some of the most promising electrodes to promote the HER. However, most of them are not yet at the absolute benchmark level and need to be (further) modified. For example, materials that contain metal chalcogenide as part of a composite, *e.g.*, in combination with metal phosphides or nitrides, achieve the efficiency of Pt/C or the best species discussed so far in Section 7.

#### 7.5.5 Metal-nonmetal compounds bearing different nonmetal elements as potential HER electrocatalysts

This subsection discusses metal-non-metal based multicomponent materials, such as composite materials that include *e.g.*, mixed metal boride, carbide, nitride, phosphide, oxide sulphide, selenide and telluride phases. It is not limited to heterostructured materials, but also includes homogeneously structured multi-element compounds, for example homogeneously structured sulphonitride phases. These multielement compounds have also been included in several review articles^577,916,924,1120^ and we focus on what has been reported recently and describe only extremely efficient HER electrocatalysts.

WCN-based electrodes were found to highly actively and stably catalyse HER as shown by Zhao *et al.*^1181^ or by Chen *et al.*^1182^ A series of binary NiP_2_, NiSe_2_ and ternary NiP_*x*_Se_*y*_ compounds have been synthesised and checked for their HER catalytic capabilities:^1183^ NiP_1.93_Se_0.07_ exhibited the best HER performance (*η* = 84 mV; *j* = 10 mA cm^−2^; 0.5 M H_2_SO_4_). HER efficiency experienced a real boost from work that was published very recently.^1179,1184–1189^

**Fig. 81:**
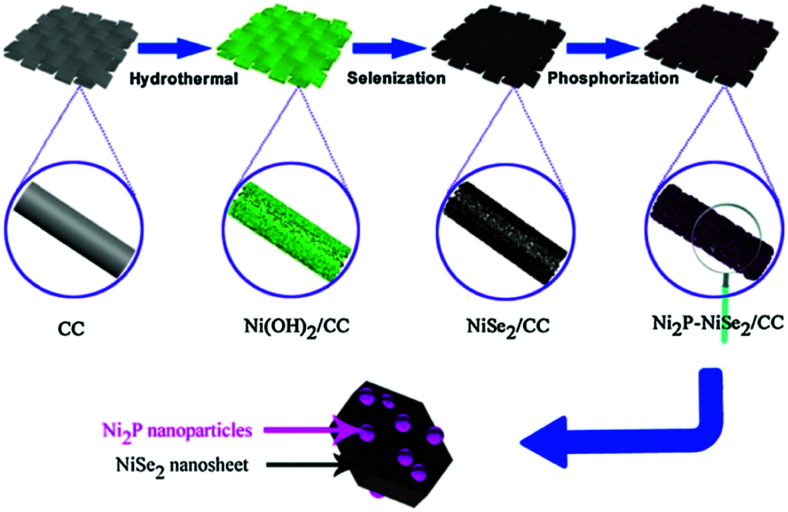
Schematic illustration of synthesis of Ni_2_P–NiSe_2_/CC heterostructure catalyst. Reproduced with permission from ref. 1188 Copyright Elsevier 2020.

Phosphorisation of NiSe_2_ nanoplate arrays delivered a self-supported electrocatalyst comprising a nickel chalcogenide (NiSe_2_) and a nickel pnictide (Ni_2_P) phase^1188^ (Fig. 81) and turned out to highly efficiently and stably support HER electrocatalysis in 1 M KOH (*η* = 66 mV; *j* = 10 mA cm^−2^).

**Fig. 82:**
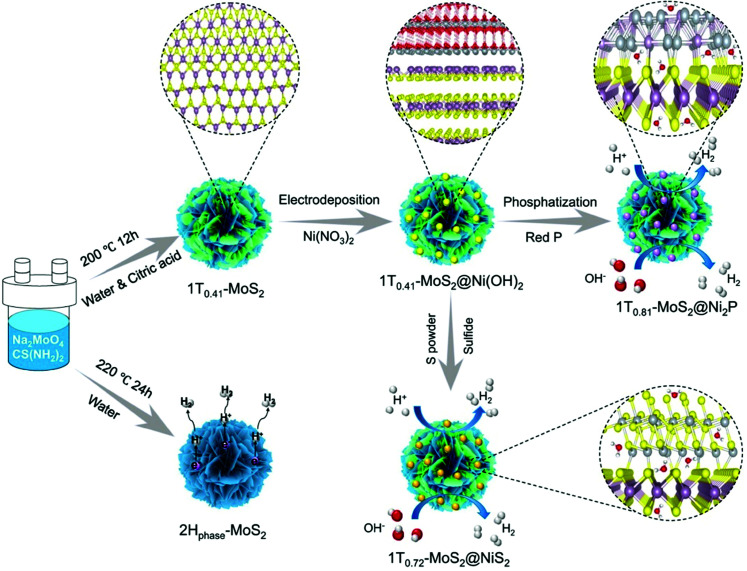
Schematics of the 1T_0.72_-MoS_2_@NiS_2_ and 1T_0.81_-MoS_2_@Ni_2_P synthesis steps. Reproduced with permission from ref. 1185 Copyright Nature Publishing 2021.

**Fig. 83:**
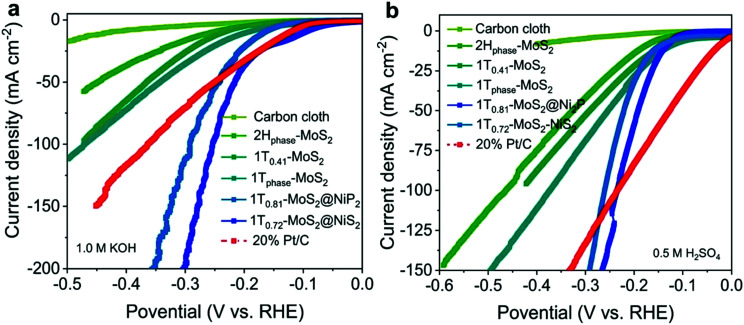
The electrocatalytic HER performance of 1T_0.72_-MoS_2_@NiS_2_ and 1T_0.81_-MoS_2_@Ni_2_P hybrid materials in comparison with MoS_2_, carbon cloth and Pt/C. (a) LSV curves in 1 M KOH. (b) LSV curves in 0.5 M H_2_SO_4_. Reproduced with permission from ref. 1185 Copyright Nature Publishing 2021.

A very recently-published work aimed at improving the interface between a transition metal chalcogenide-based- and a transition metal phosphide-based phase.^1185^ Materials comprising MoS_2_/NiS_2_ phases (Fig. 82) were found to support HER electrocatalysis efficiently and stably with the MoS_2_/NiS_2_ material performing slightly better. In particular in alkaline media, these two-phase multi-element species significantly outperform Pt/C (Fig. 83).

**Fig. 84:**
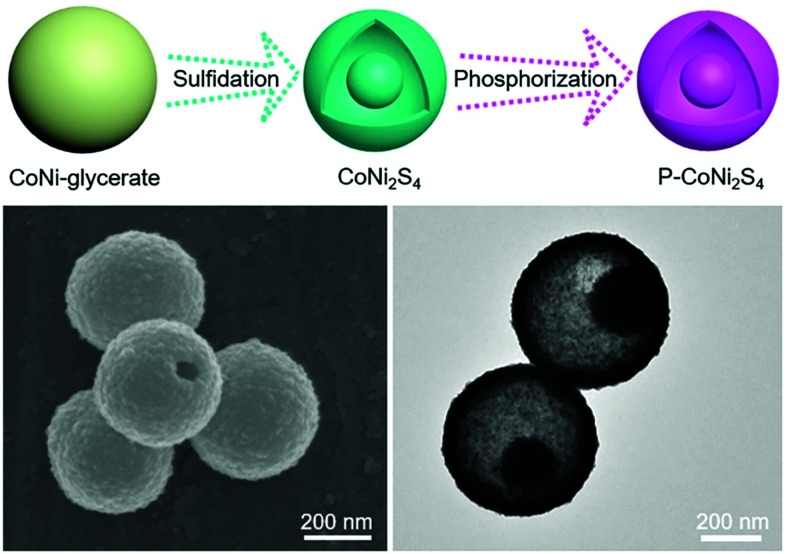
Schematic presentation of the synthesis steps leading to P-CoNi_2_S_4_ YSS particles and TEM images of these particles. Reproduced with permission from ref. 1179 Copyright Wiley 2021.

Even slightly better HER activity (*h* = 280 mV; *j* = 400 mV, 1 M KOH) was recently measured for phosphorous doped CoNi_2_S_4_^1179^ particles with yolk–shell architecture (P-CoNi_2_S_4_ YSS, 570 nm in diameter); a spherical interior solid CoNi_2_S_4_ core is surrounded by a porous shell made of the same material and separated from the core by a void space, Fig. 84).

This selected literature search confirms that metal-nonmetal compounds bearing different nonmetal elements belong to the most promising hydrogen evolution catalysts.

### 7.6 Steel-based HER and OER electrocatalysts

According to the EN 10020 standard established by the European Committee for Standardisation, steel is a material in which the mass fraction of iron is greater than that of any other element present in the material and the carbon content is generally less than 2%. Mild steel is the most common form of steel due to its low price. Corrosion-resistant steel can be achieved by coating procedures, *e.g.*, applied to mild steel,^1190,1191^ or by the chosen ingredients leading to so-called stainless steels.

Maurer and Strauss from the Krupp company registered two patents on stainless steel in autumn 1912, which were issued in 1918.^1192,1193^ In the Strauss-Maurer phase diagram, three main families of nickel-chromium based steels are isolated: martensitic (low nickel and low chromium content), austenitic (higher nickel content), and ferritic (high chromium content) steels.

The first reports of electrocatalytically initiated water splitting on steel surfaces were rather fundamental research studies, concerning the kinetic study (particularly) of HER^1194–1202^ and OER revealing that, 40–50 years ago, water splitting was not seriously taken into consideration as a technique suitable for the production of alternative fuels. Later steel has been intensively investigated as a conductive support for OER or HER active species^1203–1235,1238^ as well as for OER or HER catalytic active alloys.^1236^ In one of the latest published articles dedicated to the exploitation of steel as a conductive substrate for HER active electrocatalysts, Jothi *et al.*^1213^ describes a very interesting approach that uses scrap stainless steel wires to construct very active hydrogen-evolving electrodes under industrial conditions.^1213^

However, this subsection focuses on the use of steel as a real electrocatalyst, thus presenting the catalytic active species itself. It is very difficult to distinguish between approaches that take advantage of steel just as a conductive substrate (classical substrate-layer architecture) and approaches that are based on doping the outer sphere of the steel without completely or partly destroying the role of steel to act as the active catalyst itself, *i.e.*, ingredients of steel as well as embedded atoms or ions are both active for catalytic promotion. This topic is the subject of a recent review article.^22^

#### 7.6.1 HER electrocatalysis on steel

Various groups are still researching the mechanism of hydrogen evolution on steel surfaces and it is suggested that the active site for water reduction is the protonated Fe-OH_2_^+^ group and, therefore, hydrogen evolution on steel surfaces should be discussed following the Volmer–Tafel–Heyrovsky scheme, generally valid for metal surfaces.^1221,1237^

It is a common knowledge that untreated steels basically show reasonable OER activity^1238^ but quite poor HER activity^1239–1241^ for electrocatalytically initiated water splitting in aqueous solutions. Advanced tools were developed to unmask activity composition relationships that allow a knowledge-based tailoring of the composition and structure of the catalytically active outer sphere.^1240^

Studies that use steel-based materials to promote light-driven or photoelectrocatalytic hydrogen evolution are still rare.^1242–1247^ The electrocatalytically driven HER on (mild) steel surfaces in aqueous solution was first examined by Leach and Saunders in 1965^1194^ and the first time that stainless steel was reported as a hydrogen-evolving electrode in an alkaline medium dates back to 1970,^1195^ or to 1976^1196^ (acid) and 1977^1197^ (neutral). These early studies lacked any kind of investigation of the HER efficiency as, for instance, determination of the long-term current–voltage behaviour or the Faraday efficiency.

Decades later Olivares-Ramirez *et al.*^1248^ compared the HER behaviour of three different steel types: 304, 316, and 430 : 316 steel was the best, owing to its highest Ni content. A more detailed investigation of efficiency aspects of the HER on 316 steel surfaces was presented in 2010 by De Silva Munoz *et al.*:^1249^ equal performance was reached in phosphate solution (1 M KH_2_PO_4_) and in 25 wt% KOH solution, with the advantage of working at milder pH 4. However, the overall efficiency of untreated stainless steels for HER is rather low (*η* = 340 mV; *j* = 1.3 mA cm^−2^; pH 4).^1249^ Steel 316 samples, mechanically or chemically surface-modified, were checked for their full water-splitting capabilities in 30 wt% KOH in 2016.^1250^ The sum of the overpotentials for full water splitting at *j* = 175 mA cm^−2^ occurring on both sides amounted to 1270 mV for mechanically-treated steel electrodes.^1250^

Some of the authors evaluated Ni42 steel as a potential HER electrode material for water electrolysis at pH between 0 and 14.6.^1239^ Electro-oxidised samples obtained after hard anodisation in 7.2 M NaOH showed superior HER properties (*η* = 333 mV at *j* = 10 mA cm^−2^; pH 13). However, the performance did not even come close to that of state-of-the-art, noble HER electrocatalysts.^1239^ In addition, a Pt counter-electrode was used, which can falsify the results, since positive potentials applied to the Pt counter-electrode (oxygen development takes place) can lead to the dissolution of Pt and consequently to its deposition on the working electrode.^1251–1255^ It has been shown that this is a serious problem at least for long-term polarisation experiments in strong acids.^1744^ As shown later in the manuscript, the drawing of OER active components from the inside of the material to the surface of the material by electrochemical measures that goes along with corrosion-engineering applied to the steel, represents one essential strategy to increase the OER activity of steels. However, the transition-metal hydroxides obtained through corrosion engineering exhibit weak surface hydrogen adsorption at alkaline conditions leading to sluggish HER kinetics^1256^*versus* noble metals electrocatalysts.^1257^ It is therefore reasonable to assume that the apparently naturally low activity of steel to promote HER at negative electrode potentials has its origin in the absence of adequate noble ingredients.

Therefore, without doping with known HER active ingredients, steel-based HER electrocatalysts, whose HER performance is comparable to that of the current state-of-the-art HER electrocatalysts, such as carbon-supported platinum (Pt/C),^906,1258^ or transition metal phosphides,^1259,1260^ can hardly be synthesised. Doping might be realised through addition of noble metals or, in case non-metal doping is intended, through reaction of the metal-surface with nonmetals or nonmetal-containing compounds *e.g.*, at higher temperature.

Non-metal doping of steel, for example, nitriding, is highly established to improve mechanical properties.^1261^ Enhanced HER activity of 316 steel was obtained by surface modification of 316 steel upon a sulphurisation, phosphorisation, and nitridation procedures in early 2017.^1262^ Sulphurisation turned out to be the most effective modification procedure (*η* = 136 mV; *j* = 10 mA cm^−1^; 1.0 M KOH).^1262^ Shortly thereafter, simultaneous nitridation and phosphorisation were applied to 304-type steel mesh, leading to self-supported HER catalysts stably and efficiently supporting alkaline HER (*η* = 230 mV; *j* = 12 mA cm^−1^; 1 M KOH).^1263^ Later, mono (nitrogen)-doped anodised stainless-steel mesh exhibited slightly better activity and high stability towards HER in 1 M KOH (*η* = 146 mV; *j* = 10 mA cm^−1^; 1 M KOH).^1264^ Besides wet electrochemical approaches, a nitrogen glow discharge plasma has been found to be capable for nitrogen-doping into the surface of steel 316 and resulted in a substantial enhancement of the HER activity of 316 steel (*η* = 220 mV at *j* = 10 mA cm^−2^, 20 wt% KOH).^1265^

Also in 2017, Anantharaj^1266^ reported on stainless steel scrubber (AISI 434 steel) used as working electrodes directly for OER and HER electrocatalysis.

**Fig. 85:**
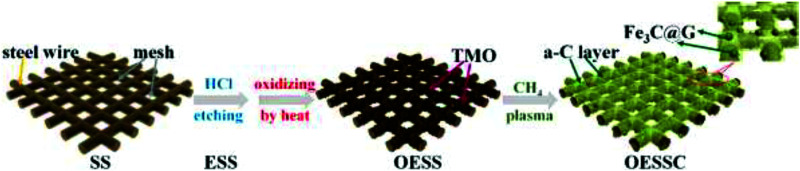
Schematic illustration of the fabrication procedure of SS-based electrodes. Reproduced with permission from ref. 1267. Copyright Elsevier 2019.

The creation of metal carbides on the surface of steel essentially serves the purpose of increasing the surface hardness. The first example of carbide-modified steel as a potential HER electrocatalyst was published in early 2019:^1267^ Graphene-encapsulated Fe_3_C nanoparticles obtained on the surface of stainless steel 316L samples (Fig. 85) exhibited substantially improved HER activity in 1.0 M KOH.

Austenitic stainless steel 304, wet-chemically treated in boiling NaNO_3_/NiCl_2_ solution followed by phosphorisation (Ni-P doped) exhibited an increased HER activity (*j* = 10 mA cm^−2^ at *η* = 149 mV; 1 M KOH) and were found to be stable towards HER for 25 h.^1268^

We mentioned the borderline cases that do not just take advantage of steel as a conductive support based on classical coating strategies (like electrodeposition, physical vapor deposition, …) and steel ingredients still take actively part in the catalysed chemical reaction but in interaction with substances applied to the steel from the outside. This latter procedure can be realised through fine doping at a low level. Ring *et al.*^1269^ found that the HER activity of a Ni_42_ steel electrode drastically increases when using a Pt counter electrode. Simultaneously to hydrogen evolution occurring on the Ni_42_ working electrode, a platinum transfer from the counter electrode to the Ni_42_ electrode takes place, thereby substantially improving the HER activity of the Ni_42_ alloy (*η* = 140 mV at *j* = 10 mA cm^−2^; pH 1). Upon repetitive cycling of the potential of a steel 316 electrode between −0.2 V *vs.* RHE and +1.4 V *vs.* RHE in 6.0 M NaOH, Fe/Ni-oxide species are formed on the steel electrode.^1270^ Decoration of the conditioned surface with low level of gold completed the surface modification procedure and resulted in an enhancement of the HER activity.

**Fig. 86:**
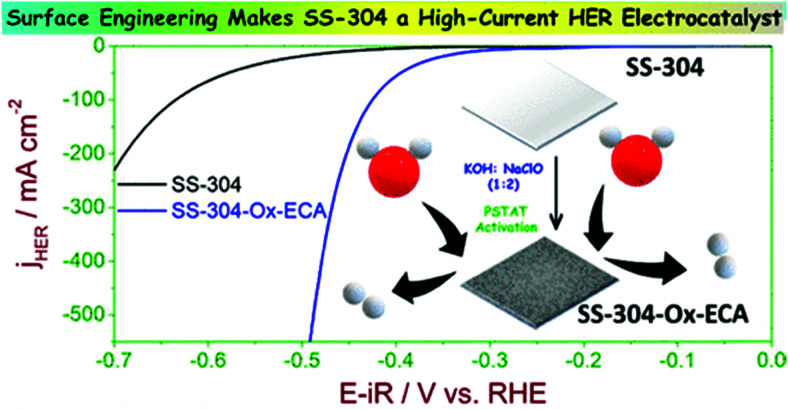
The HER activity of stainless steel 304 was enhanced in a two-step activation process comprising chemical oxidation (KOH + NaClO). Reproduced with permission from ref. 1271. Copyright American Chemical Society 2020.

Surface engineering consisting of chemical oxidation (KOH + NaClO) and electrochemical potentiostatic resurfacing applied to AISI 304 steel resulted in enhanced HER activity (*η* = 550 mV; *j* = 200 mA cm^−2^; 1.0 M KOH); (Fig. 86)^1271^ due to Ni(OH)_2_ formation.

In a three-step procedure comprising chemical etching in HCl, electrochemical anodisation followed by thermal treatment the HER properties of steel 304 were substantially improved.^1272^ The etching step creates a rough surface and remove of chromium oxide. The anodisation leads to tubular iron oxide-based nanostructures and thermal annealing reduces the oxide layer.

Based on the results described above, the steel based HER electrocatalysts produced by various surface modification processes have a bright future, particularly when taking into consideration the low investment costs due to the low price and cheap mass-production.

#### 7.6.2 OER electrocatalysis upon steel

The electrocatalytically-initiated water oxidation reaction contributes to most of the cell overvoltage, owing to the sluggish OER kinetics.^1273^ Here, are not addressed approaches that are based on using steel as a conductive substrate only.

##### 7.6.2.1 Oxygen evolution on untreated or *in situ* treated Ni-Cr-based stainless steels

Around 40 years ago mild steel was pre-treated with Inco Type 123 nickel powder containing paint and afterwards sintered for 10 min in NH_3_ atmosphere at 870 °C:^1274^ substantial interdiffusion of nickel and iron occurs upon heat-treatment, leading to an OER electrocatalyst with convincing activity and durability (>1000 h) for water electrolysis in 30 wt% KOH at 80 °C (*η* = 200 mV at *j* = 100 mA cm^−2^). No data from electrolysis tests under normal laboratory conditions were shown, making it difficult to compare these early results with more recent ones. AISI 302 steel turned out to be an efficient and durable oxygen-evolving electrode in strong alkaline environment (*η* ≈ 400 mV at *j* = 6.3 mA cm^−2^; pH 14).^1275^ However, changes of the morphology whilst long-term usage and characterisation of the catalytic active species/determination of the faradaic efficiency were not shown. A study that is rather dedicated to unmask the mechanism of the layer formation on steel 430, 304 and 316 than with determining the ability to split water was presented by Abreu *et al.* in 2006.^1276^

The use of AISI 316L stainless steel as a simple, stable and competitive oxygen-evolution electrode in alkaline media for aqueous lithium–air batteries has been reported by Moureaux *et al.*^1277^ Long term (>3000 h) polarisation was performed in 5 M LiOH (*η* ≈ 500 mV) at an averaged current density of *j* ≈ 20 mA cm^−2^. The changes of the catalyst regarding, for example, crack formation and composition of the surface in operation were investigated in detail. After 500 h of activation *via* polarisation the steel electrode outperforms many non-PGM (and even noble) OER electrocatalysts in alkaline environments. Remarkably, the electrode shows self-healing capabilities as the “active layer” is formed *in situ* from the components of the bulk stainless steel. Would this layer detaches or degrades, it would reform *in situ* using the bulk components of the stainless steel according to the same mechanisms as for the first layer.

Three years later Sun *et al.* investigated the same material exploited for OER electrocatalysis in more diluted alkaline medium.^1238^ Without any pre-treatment, steel 316 showed satisfying OER electrocatalytic capability (at eye level with pure Nickel):^1278,1279^*η* = 370 mV at *j* = 10 mA cm^−2^ and sufficient durability (20 h of chronopotentiometry-CP. This presents the first study in which the charge-to-oxygen-conversion rate whilst OER electrocatalysis was quantified for 316 steel-based catalysts. Heterolayered Ni–Fe hydroxide/oxide nanostructures created on 316 steel upon constant current density electrolysis through dealloying plus surface oxidation.^1280^ Thickness, morphologies and compositions of the nanostructures did strongly depend on the electrolysis time. Under optimised preparation conditions, the anode proved active and stable under near-industrial electrolysis conditions (*η* = 380 mV; *j* = 400 mA cm^−2^; *T* = 348 K; 1.0 M KOH).

##### 7.6.2.2 Oxygen evolution on *ex situ* treated Cr–Ni-based stainless steels

This subsection covers materials treated (activated) in a different medium from their medium of usage.

The first example of a series of studies in which steel was for the first time intentionally surface modified (without bringing heteroelements) prior to electrocatalysis in order to improve the electrocatalytic water-splitting properties was shown in 2015.^1281^

AISI 304 stainless steel was, upon a very straightforward surface oxidation in an air/chlorine mixture at room temperature, converted into a durable OER electrocatalyst with acceptable OER activity at pH 13 (*η* ≈ 260 mV at *j* = 1.5 mA cm^−2^) and pH 7 (*η* ≈ 500 mV at *j* = 0.65 mA cm^−2^).

**Fig. 87:**
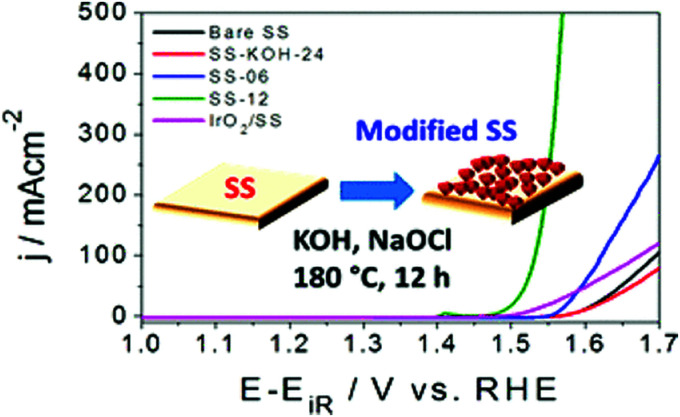
Fe_2_O_3_//NiO nanocrystals were formed on the surface of corroded AISI 304 steel and significantly improved the capability of the material to act as an OER electrode. Reproduced with permission from ref. 1285 Copyright American Chemical Society 2017.

X-ray photoelectron spectroscopy (XPS) analyses showed that a thin film of FeCr oxide was formed on the stainless steel treated with chlorine/air. The use of iron chromium oxide-based catalysts is not limited to water electrolysis but has received general attention for catalysis (reforming of ethylene glycol in aqueous phase,^1282^ pyrolysis of diesel fuel,^1283^ H_2_ formation from biomass^1284^). Anantharaj *et al.* used a combination of KOH and hypochlorite as the corroding agent and promoted the OER, enhancing NiO incorporated Fe_2_O_3_ nanocrystals whilst removing Cr on the surface.^1285^ This strategy substantially enhanced the OER activity of stainless steel AISI 304 (Fig. 87).

The best OER performance (*η* = 212 mV, *j* = 12 mA cm^−2^, 1.0 M KOH) determined for flat AISI 304 electrodes were achieved when the steel was pre-electrooxidised under harsh electrochemical conditions (*j* = 1.8 A cm^−2^ in 7.2 M NaOH).^1286^ The aim of this study was to mimic the composition (67 at % Ni, 33-at % Fe) of recently developed advanced- and highly active Fe–Ni-based OER electrocatalysts (Ni_(2/3)_Fe_(1/3)_) made by the Bell and Boettcher groups.^104,1279^

Anodic water-splitting in neutral medial is considered to be more challenging than in alkaline regime and the overpotentials obtained at pH 7 required for comparable OER current densities are substantially higher.^1281^ Lee *et al.* reported in 2017 about a 304-steel-based electrode that sufficiently supports oxygen evolution at pH 6.7–7.3;^1287^ after electrochemical oxidation in strong alkaline medium, the samples exhibited good performances (*η* = 504 mV at *j* = 10 mA cm^−2^) in a CO_2_-saturated bicarbonate electrolyte. Spectroscopic analyses unmasked NiOOH as the active species.

As mentioned, mild steel was investigated as potential HER electrode in the late 1960s.^1194^ Schäfer *et al.* found that pre-oxidation with Cl_2_/air treatment of S235 steel before performing OER electrocatalysis at pH 13 and pH 7 can significantly improve its electrocatalytic activity.^1288^ The OER kinetics at pH 13 were moderate (*η* = 347 mV at *j* = 2 mA cm^−2^); however, the one determined at pH 7 (*η* = 462 mV at *j* = 1 mA cm^−2^) is comparable to that of the CoPi catalyst introduced by Nocera and Kanan in 2008.^1309^

In an update of their initial work^1277^ the group around Chatenet intended to extend the developed concepts to more widely used electrolytes and reported in 2019 on *ex situ* (in 5.0 M LiOH or in 5 M KOH) activated steel of the same austenitic steel type 316L for use in KOH electrolyte.^1454^ The steel-based anodes generated this way were compared to *in situ* (in 5 M KOH or in 5 M LiOH) activated steel 316 L with respect to OER: (i) *ex situ*-activated electrodes perform comparable to *in situ*-activated ones (the latter being a much more time-consuming procedure), the resulting OER activities in KOH electrolytes being high compared to other non-precious metal electrocatalysts; (ii) KOH(aq) is a better electrolyte for activation than LiOH(aq), whatever the final alkaline electrolyte used. (iii) 316 L electrodes did not show significant degradation in performance and surface over a few 100 h of OER operation, which should be highlighted given the very large current densities experienced (a few 100s of mA cm^−2^).

Very often electro-activation of austenitic stainless steel was carried out in strong alkaline media upon applying relatively high current densities whereas the OER properties have been checked thereafter in more diluted alkalines.^1286^ Very recently a group from Japan has taken a different path;^1289^ using 1.0 M KOH for the anodisation-based electroactivation of 316 stainless steel carried out at *j* = 30 mA cm^−2^ followed by the evaluation of the OER properties in 7 M KOH, basically done at *j* = 100 mA cm^−2^. This *soft electroactivation* resulted in the formation of a 50 nm thick nanofiber layer comprising Ni–Fe hydroxide (catalyst layer). However, through applying 20 000 potential scans the outer sphere (catalyst layer) was found to be unchanged whereas an NiFe-hydroxide interlayer was formed in between substrate and catalyst layer. The overall OER efficiency was comparable to the ones usually achieved with activated austenitic stainless steels.

Austenitic stainless steels like AISI 316 or 304 show after long tern usage as OER electrode in alkaline media exhibit cracks on their surface^1277,1287,1867^ which, were more a sign of self-healing power than of limited stability.

As a catalytic active material, binary Fe–Ni systems are of great general importance (they are known Fischer-Tropsch catalysts^1290^); they catalyse the selective conversion of furfural to methylfuran,^1291^ of m-cresol to toluene^1292^ and have been used for the catalysis of the steam reforming reaction (tar → syngas)^1293^ or the partial oxidation of methane to syngas.^1294^

Both chemical- and electrochemical activation have been applied to a stainless steel plate.^1295^ The stainless steel was corroded in ammonium solution at 200 °C under pressure, resulting in reasonable activity (*η* = 290 mV at *j* = 10 mA cm^−2^; 1.0 M KOH) and durability. A different austenitic stainless steel, namely AISI 302 was chemically activated using peroxydisulphates leading to a uniform brown film comprising Fe(Ni)OOH with rippled sheet structure:^1222^ this material outperforms pure nickel (*η* = 300 mV at *j* = 10 mA cm^−2^; Tafel slope = 34 mV dec^−1^).

**Fig. 88:**
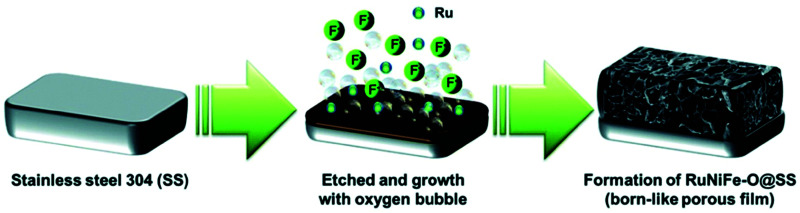
Schematic of the formation mechanism of RuNiFe-O@SS electrocatalyst. Reproduced with permission from ref. 1298 Copyright RSC 2021.

Selenisation was found to be a very good method to increase the OER activity of stainless steels.^1229,1296,1297^ When high-temperature is applied to austenitic steels, a ternary phase NiFeSe forms, *i.e.*, Se in the nickel iron selenide directly bonds to iron through a covalent bonding, hence steel does not simply act as a conductive substrate. Recently, Xiao *et al.*^1229^ reported on 304 stainless steel with a modified surface by thermo-selenisation and subsequent acid etching (enlargement of the surface): Se_*x*_Ni_0.75_Fe_0.25_OOH is claimed to be the catalytic active phase showing sufficient activity (*η* = 293 mV; *j* = 500 mA cm^−2^; 1.0 M KOH). The number of papers dealing with noble-metal-doped steel surfaces,^1269^ or noble metal doped surface modified steels^1298^ is still rather limited. Very recently Kim *et al.*^1298^ reported on a straightforward surface modification strategy applied to stainless steel AISI 304 (Fig. 88) comprising an etching procedure followed by anodisation. A Ni–Fe oxide containing periphery with trace amounts of Ru was created that converts the steel into an effective full water splitting electrocatalyst (RuNiFe-O@SS; cell voltage of 1.83 V in 1.0 M KOH, *j* = 100 mA cm^−2^).

##### 7.6.2.3 3D-Steel-based OER electrocatalysts

**Fig. 89:**
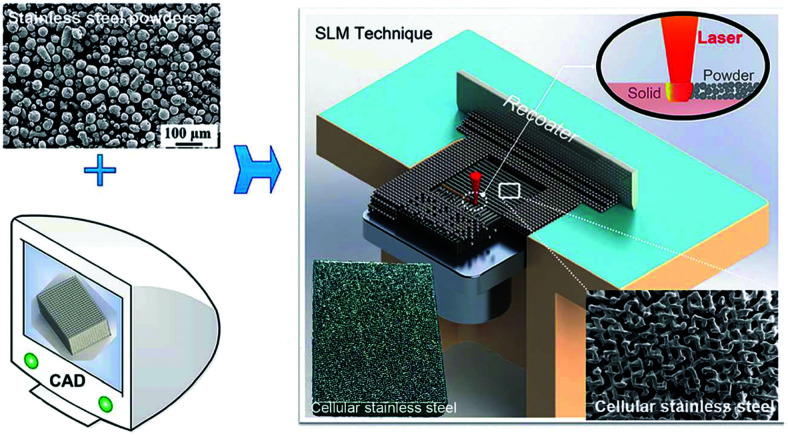
Schematic representation of the fabrication process of the CESS. Reproduced with permission from ref. 1299. Copyright RSC 2017.

To increase the catalytic active surface per projected area, 3D steel-based electrode materials have been developed. Huang *et al.*^1299^ have chosen a quite time consuming, unusual approach; a 3D stainless steel electrode designed *via* CAD technique was generated *via* selective LASER melting of stainless steel powder (Fig. 89). The authors think that the high current densities (*η* = 332 mV at *j* = 40 mA cm^−2^ at pH 14) are basically due to the electrode geometry and are not solely based on the electrode material itself.

Assuming that 3D structures based on steel like stainless steel sponges,^1300^ felts,^1301^ mats,^1296^ tantangles, scrubbers^1266^ and different sorts of meshes^1223,1228,1302^ are omnipresent and are already exploited as electrodes for water electrolysis, the usefulness of a time-consuming generation is at least worthy of discussion.

Transforming rusty stainless-steel mesh into stable cathodes for batteries applications was shown.^1303^ Generally, surface modified steel meshes have become popular as 3D electrodes for water splitting purposes exhibiting a high current density and relatively low electrode potential (*η* = 230 mV at *j* = 20 mA cm^−2^)^1228^ outperforming Ni metal-based catalysts like Ni foam.

Fast removal of gas bubbles is a prerequisite for an efficient splitting of water into its gaseous cleavage products. It was found that Nonwoven stainless-steel fabrics are suitable for increased gas bubble escape rate during the water electrolysis process.^1210^

In an update of their initial work Schäfer *et al.* applied a phosporisation procedure to S235 steel^1304^ capable to convert the starting material into a quite active and stable OER electrocatalyst (*η* = 326 mV at *j* = 10 mA cm^−2^; 1 M KOH). Recently-published studies focused either on increasing the OER efficiency or on further increasing the long-term stability of the steel-based anode with respect to oxygen evolution:^1296,1305^ modified stainless steel (316L) fiber felt^1306^ through an electrooxidation-based approach ended up in Fe/Ni/Cr hydroxides/oxides exhibiting good long-term durability (550 h of chronopotentiometry at *j* = 100 mA cm^−2^; *E* = 1.54 V *vs.* RHE).

**Fig. 90:**
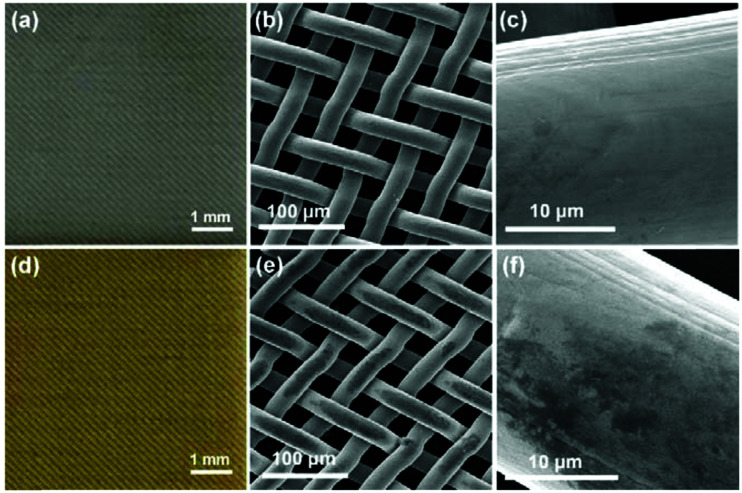
Digital photos (a and d) and SEM images (b, c, e and f) of SSM-Pristine (a–c) and SSM-Cathodisation (d–f). Reproduced with permission from ref. 1307. Copyright Elsevier 2020.

The most commonly-used strategy to activate austenitic stainless steel for better OER properties involves polarisation at positive potentials, which yields Ni-based species enrichment on the surface.^1277,1286,1295^ Etzold's group reported on a cathodisation-activation process carried out at potentials down to −0.6 V *vs.* RHE applied to stainless steel (316L) mesh in 0.1 M KOH (Fig. 90).^1307^ Obviously, Ni diffusion occurs through HER mediated adsorption induced surface segregation. The reduced Ni-species are then oxidised to NiOOH/Ni(OH)_2_ during OER, converting stainless steel mesh into an active OER electrocatalyst (*η* = 319 mV at *j* = 100 mA cm^−2^; 1.0 M KOH).

**Fig. 91:**
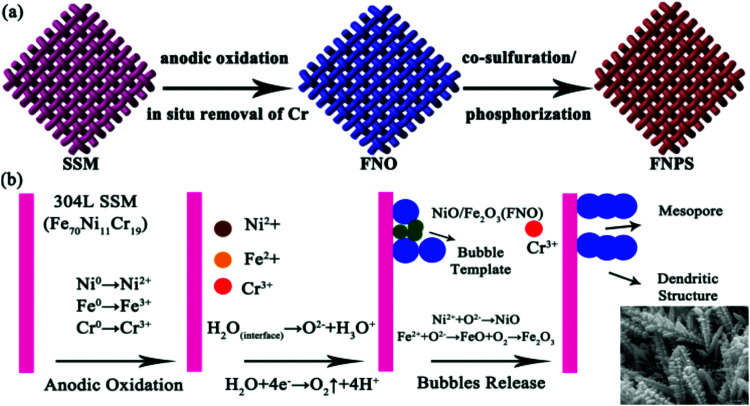
An OER-based current density of *j* = 100 mA cm^−2^ was achieved at an overpotential of *η* = 173 mV in 1 M KOH. Reproduced with permission from ref. 1308. Copyright Elsevier 2020.

Commercial 304 stainless steel mesh has recently been converted into a highly active and stable OER electrocatalyst (for more than 2 months of operation in 1.0 M KOH electrolyte).^1308^ The strategy comprises oxygen gas bubble formation that acts together with the release of Cr as a co-template. Conductivity and active site density are then increased by a co-sulphuration/phosphorisation step (Fig. 91).

##### 7.6.2.4 Oxygen evolution on *ex situ*-treated co-based steels

Cobalt-based electrode materials that actively support anodic water splitting, particularly under neutral conditions, have been known for more than ten years when Nocera and Kanan reported on the Co-Pi OER electrocatalyst.^1309^ Among them are Co-based cobalt borate/graphene,^1310^ nano-scaled cobalt oxide-based catalysts like Co_3_O_4_ nanowire arrays,^1311^ and graphene Co_3_O_4_ nanocomposites.^1519^ Some steels contain a considerable amount of cobalt.^1312^ Schäfer *et al.* reported in 2016 on the possible use of a cobalt-containing hot-work steel as an electrode for water electrolysis.^40^ The cobalt content on the surface of X_20_CoCrWMo_10-9_ was substantially enhanced following chromium and iron depletion whilst electro-oxidation in alkaline media. An intrinsically-grown, Co_3_O_4_-based ceramic–alloy composite with absolute benchmark OER activity at pH 7 was generated this way (*η* = 298 mV at *j* = 10 mA cm^−2^), significantly outperforming IrO_2_–RuO_2_,^40^ Co–Pi,^1309^ or graphene Co_3_O_4_ nanocomposites^1518^ in neutral electrolyte. Co_3_O_4_ is one of the most favoured compounds in inorganic materials science with advanced functionality (sensor applications,^1313–1315^ lithium storage,^1316^ supercapacitor^1317^). It has been investigated in depth for various applications in the broader context of heterogeneous catalysis (OER-^1512^ and ORR^1318–1320^ photocatalysis^1321^). It sufficiently catalyses the oxidation of CO,^1322^ which plays a major role in cleaning air and car emissions^1323^ and represents one of the most extensively investigated material in heterogeneous catalysis.

**Fig. 92:**
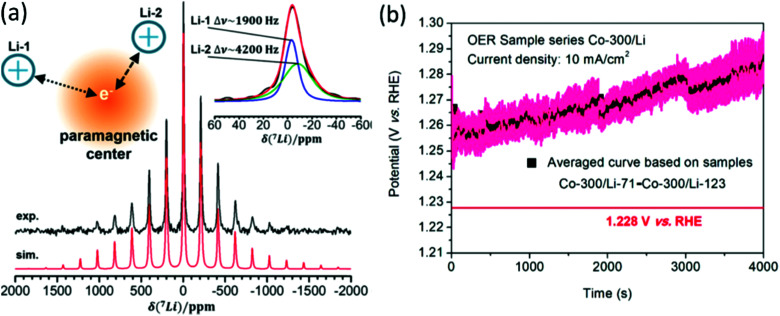
(a).7Li MAS NMR spectrum of (as-prepared) Co-300/Li recorded at 11.7 T and a MAS frequency of 25.0 kHz, showing a broad, asymmetric spinning sideband pattern characteristic of a strong electron-Li dipolar interaction. (b). Averaged chronopotentiometry curve based on 53 samples of the sample series Co-300/Li (blacksquares) with standard error bars (magenta). Reproduced with permission from ref. 1324. Copyright American Chemical Society 2018.

In an update, Schäfer *et al.* applied lithium ion doping to the Co_3_O_4_ comprising the outer sphere of the electro-oxidised tool steel X_20_CoCrWMo_10-9_.^1324^ XPS investigation carried out for the nonlithiated (Co-300) and lithiated samples (Co-300/Li) revealed an energy gap between the oxidation-state of the weakest and strongest oxidised cobalt ion that becomes significantly more pronounced upon lithiation. This suggests Li intercalation into the cobalt-containing layers, resulting in a valence mixture of Co(iv) and Co(ii). Two distinct Li^+^ sites located at fixed positions within the Co-containing steel–ceramic framework can be unmasked *via* solid state NMR spectroscopy due to their different interaction with the paramagnetic Co(ii) or Co(iv) centers (Fig. 92a). The lithiated steel exhibited substantial oxygen evolution at pH-neutral conditions close to the thermodynamic limit (Fig. 92b) and therefore outperforms all other materials compared to what is published in earlier contributions with respect to the voltage–current behaviour.

However, the unique OER properties only last about 2 h (*j* = 10 mA cm^−2^) or 5 h (*j* = 5 mA cm^−2^) in 0.1 M KH_2_PO_4_/K_2_HPO_4_ mixtures.

##### 7.6.2.5 Oxygen evolution on *ex situ*-treated steels at low pH Values

A few papers report on steel-based oxygen evolving electrodes used for water electrolysis at low pH value. As iron is the main compound of steel, it is fully understandable that creation of corrosion-resistant (protecting) layers on steel substrate is a prerequisite to successfully design reasonably-stable steel-based anodes working in acidic regimes. The first report on anodic water splitting realised by steel-based electrodes appeared in 2017:^41^ cobalt-based tool steel X_20_CoCrWMo_10-9_ was converted though electrooxidation in LiOH electrolyte into a reasonably active and stable OER electrode (39 μg mm^−2^ weight loss after 50 000 s of chronopotentiometry at *j* = 10 mA cm^−2^ in 0.05 M H_2_SO_4_; *η* = 574 mV at *j* = 10 mA cm^−2^). The OER mechanism is believed significantly impact the material removal associated with the release of oxygen from or near the surface.

The so-called “oxide route” is used for materials that release oxygen out of the metal oxide-containing surface^1325,1326^ whereas for a different group of materials, adsorbed water molecules represent the oxygen source responsible for the OER (solution route).^422^

Typically, the oxide route leads to dominating dissolution process upon disruption of the surface, *i.e.*, yields instability. Electrochemical oxidation of Ni_42_ steel in LiOH (sample Ni_42_Li_205_) is believed to result in the formation of a metal oxide-containing outer zone that supports solution route-based OER in acidic regime accompanied by good stability:^1327^ stable overpotentials down to 445 mV are required for *j* = 10 mA cm^−2^ in 0.5 M sulphuric acid.

**Fig. 93:**
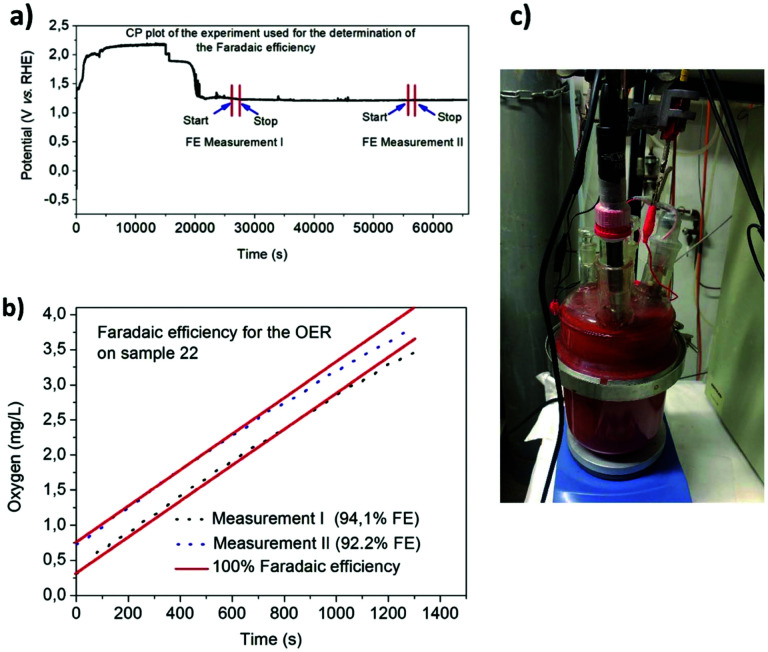
Faradaic efficiency measurements of the OER on Ni_42_ (sample 22) in a sulphuric acid/Fe_2_O_3_ suspension during chronopotentiometric measurements at 30 mA cm^−2^. Electrode area: 2 cm^2^. The areas where the FE measurements begin and end are highlighted. (b) Correlation of oxygen evolution (black dotted curve: measurement 1; blue dotted curve: measurement 2) with the charge passed through the electrode system (the red line corresponds to 100% faradaic efficiency). Reproduced with permission from ref. 1328. Copyright Royal Society of Chemistry 2020.

The first example of water electrolysis of a suspension was reported in 2020;^1328^ the basic idea of this approach was being to completely relocate the oxygen-evolving centers from the electrode to the bulk electrolyte, which should ideally be accompanied by a substantial reduction in the weight loss of the electrode during operation. An electrolysis set up, that consisted of a Ni_42_ stainless steel anode and of Fe_2_O_3_ (hematite) which is suspended in high concentration in sulphuric acid and acted as the electrolyte, exhibited oxygen evolution electrocatalysis at extremely low potential (1.26 V *vs.* RHE; 0.5 M H_2_SO_4_, *j* = 30 mA cm;^2^Fig. 92b and 93a).

**Scheme 4:**
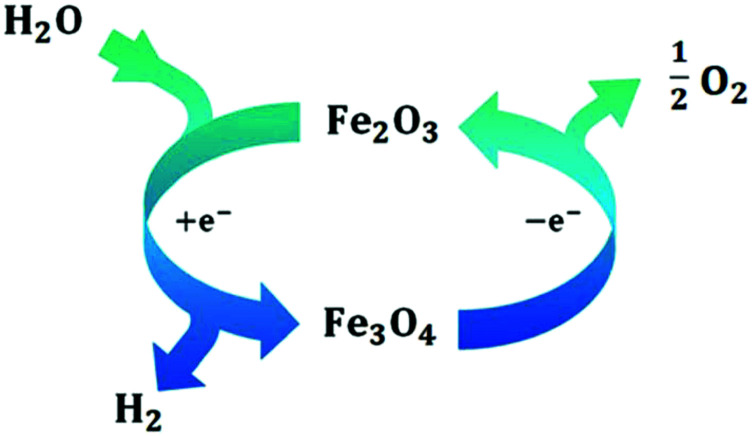
A cyclic process ensures electrocatalytically initiated splitting of water mediated through two different oxide species. Reproduced with permission from ref. 1328. Copyright Royal Society of Chemistry 2020.

The anode mass loss was negligible, and consisted exclusively of metals from the non-PGM during 100 h of operation. Experiments to clarify the mechanism suggest that Fe_2_O_3_ is converted to an Fe(ii)/Fe(iii) oxide species at the cathode, which is then converted back to Fe_2_O_3_, releasing molecular oxygen upon contact with the anode (Scheme 4).

An almost quantitative charge to oxygen conversion (>92%) was confirmed by faradaic efficiency measurements (Fig. 93b and c).

A potential around 1.26 V *vs.* RHE (corresponding to *η* = 30 mV) for *j* = 30 mA cm^2^ determined in 0.5 M sulphuric acid is currently unparalleled is still unparalleled in water electrolysis. The already mentioned steel-based approaches for acidic water splitting require overpotentials that are least 25 times higher^41,1327^ than the ones derived from suspension-based approaches. Thus, for instance ternary iridium-based systems are known to be a potential candidate as an anode material that ensures reasonable activity and stability for the splitting of acids.^1329^ However, the overpotentials derived from these mixed oxides are 15 times higher than the one derived from the sulphuric acid/hematite electrolyte system.

To clarify why the water splitting reaction mediated by means of an electrocatalytically driven cycle with suspended iron oxide species is advantageous in comparison to classic electrolysis (clear electrolyte), the energy balances for the assumed electrochemical half-cell reactions must be drawn up. Under the assumption that HER and reduction of Fe(ii) to Fe(iii) simultaneously occurs the cathodic half-cell reaction can be defined as:

15 Fe^3+^ + 3e^−^ + 2H^+^ → Fe^2+^ + H_2_

with a standard reaction Gibbs energy Δ*G*^0^_R_ of −74.2 kJ mol^−1^ which corresponds to a standard half-cell potential of +0.256 V *vs.* RHE. Given the overall reaction (gross):

16 Fe_3_O_4_ + 2H^+^ → Fe_2_O_3_ + Fe^2+^ + 0.5O_2_ + H_2_

with a standard reaction Gibbs energy of 194.3 kJ mol^−1^ and. Based on this difference of standard half-cell potentials (Δ*E* = 0.67 V), the thermodynamic half-cell potential of the OER amounts to +0.926 V *vs.* RHE, significantly below the thermodynamic half-cell potential of the water oxidation reaction (1.229 V *vs.* RHE).

## 8 Research into carbon-based HER and OER electrocatalysts

This section is dedicated to carbon-based OER and HER electrocatalysts, preferably working in aqueous media, that do not contain any “bulk” metal at all, and thus go beyond the classifications of *noble-metal-free* or *precious-metal-free*.^1330^ Metal-free catalysts that promote water- splitting upon radiation (photocatalytic water splitting)^1331^ will not be discussed here. Some reviews are entirely devoted to metal-free OER and HER catalysts.^1332^ Generally, metal-free electrocatalysts can be seen as cost-effective and environmental-friendly.^1333^ Some approaches even convert natural substances like cellulose^1334^ or clay^1381^ into water-splitting catalysts. Carbon, the main component of almost all metal-free catalysts, is the most abundant element in the world and can be produced with low manufacturing costs on a large scale. Few papers describe metal-free water-splitting catalysts which are not based on carbon or in which carbon is not the main component: semiconductor-based materials can be seen as a metal-free and carbon-free catalysts, for example antimonene nanosheets, which were identified as a potential catalyst for water electrocatalysis.^1335^ When used as water-splitting electrodes, non-metallic electrocatalysts are often chemically modified, at least on the surface. In particular, when used as oxygen-evolving electrodes, they are converted *e.g.*, to hydroxides or oxyhydroxides.

### 8.1 Catalysts with a carbon skeletal structure

**Fig. 94:**
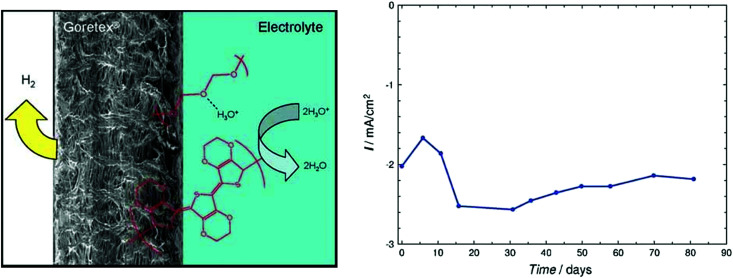
Schematic of PEDOT-based HER electrode (left side). Long-term performance of PEDOT–PEG on Goretex/Au in 1 M H_2_SO_4_ under N_2_ at −0.35 V *vs.* SCE. Reproduced with permission from ref. 1339. Copyright 2010 Wiley.

The development of (non-metal-containing) conducting polymers goes back to 1977, when Heeger and MacDiarmid discovered that oxidation with chlorine, bromine, or iodine increases the conductivity of vapour-made polyacetylene by a factor of 10^9^ in the groups of.^1336^ The low stability and conductivity remained fundamental disadvantages of early forms of so-called inherently conducting polymers.^1337,1338^ The development of organic materials that are reasonably resistant, particularly towards oxidative potentials poses a special hurdle. For understandable reasons, organic materials are more suitable to act as reductive electrodes. Winther-Jensen *et al.*^1339^ reported a polymer composite composed of poly 3,4-ethylenedioxy-thio-phene (PEDOT) and a nonconductive polymer of the polyethylene glycol (PEG) family that was found to electrocatalyse proton reduction. The PEDOT-PEG based electrocatalyst coated on porous Goretex® membrane (Fig. 94 left side) was stable towards long-term HER in 1 M H_2_SO_4_, with decent HER properties: *j* = 2.5 mA cm^−2^ at *η* = 60 mV (Fig. 94 right side). However, rather weak HER efficiency was found in neutral media.^1340^

Recent studies showed that nitrogen-doped or nitrogen and B or S or P-codoped carbon nanomaterials (nanotubes, graphene) can be alternative to PGM materials for ORR^1341–1345^ and HER^1346^ exhibiting an activity at least comparable to that of some traditional metal-based catalysts like Mo- or Ni-based systems.^1347,1348^

These results are in stark contrast to the ones reported so far: ORR and OER electrocatalysts were based on metal oxides, and the conductive substrate consisted of carbon-based materials at best.^1349^

The work based on (*N*(5)-ethlyflavinium ion Et-Fl^+^) published by Mirzakulova *et al.*^1350^ presents the first example of water oxidation electrocatalysis on a metal-free catalyst. Although the OER activity shown is weak, the work has opened a new category of water oxidation electrocatalysts.

Carbon cloth,^1351^ a cheap textile characterised by high mechanical strength, low weight, flexibility and high electric conductivity, has been intensively investigated as a conductive substrate to support various electrocatalysts for HER,^1352^ OER-^1353,1354^ or alcohol oxidation.^1355^ The relatively low surface area of carbon cloth made its direct exploitation as water oxidation electrodes a bit more demanding. Acidic oxidation represents one possible approach to substantially increase the overall OER activity of carbon cloth. Cheng *et al.* performed a chemical oxidation (pretreatment) in alkaline electrolyte yielding groups like COO^−^ upon applying positive potentials:^1356^*η* = 477 mV was required for an OER current density of 10 mA cm^−2^. The OER activity of undoped carbon-based materials developed thereafter (in most cases) remained rather low despite new efforts^1357^ and it has been found that substantial improvement in the electrocatalytic properties of carbon-based materials is very difficult to achieve without substantial replacement of carbon atoms with heteroatoms. However, at least two recently published papers clearly demonstrate that intelligently structured materials that additionally contain oxygen-based functional groups can exhibit respectable electrocatalytic OER properties (*η* = 300 mV at *j* = 10 mA cm^−2^ in 1 M KOH)^1358^ (*η* = 334 mV at *j* = 10 mA cm^−2^; 0.5 M H_2_SO_4_).^1359^

However, irreversible carbon oxidation upon OER is thermodynamically favourable, hence unavoidable, and kinetically-accelerated for functionalised carbon surfaces^1360^ (*e.g.* graphite which is more resistant, but not corrosion-proof),^1361,1362^ even moreso in presence of metal-based catalysts, whatever the pH of operation.^1363–1368^ This issue is already extremely serious in fuel cells (both acidic and alkaline),^1369^ and is worse in water electrolysers as the OER electrode operates at least *ca.* 0.5 V higher in potential than the ORR electrode in a fuel cell. Having high-surface area (disorganised and/or functionalised) carbons will have dual consequences: larger area and possibly activity for the desired reaction (OER), but also for the parasitic one (carbon corrosion), leaving little hope to obtain a stable carbon-based OER catalyst, which explains why carbon is essentially ignored by several groups for OER electrodes, both as a catalyst support and active material.

### 8.2 Heteroatom doping of carbon-based OER electrocatalysts

Heteroatom doping improves the electrical conductivity and catalytic properties, in particular the OER activity of carbons. Paraknowitsch and Sakaushi review how doping with nitrogen, boron, sulphur and phosphorus influences carbons with respect to the suitability for energy applications.^1370,1371^

While boron changes the electronic structures of carbon materials in the opposite way, but just as beneficially as nitrogen does, synergistic effects result when both dopants are used concomitantly at the same time.^1370^ Especially nitrogen doped carbons turned out to be astonishingly stable towards oxygen, *i.e.*, are able to chemically activate oxygen while not reacting themselves.^1372,1373^

In professional circles even the designation *noble carbons* made the rounds for nitrogen doped carbon-based materials.^1374^

**Fig. 95:**
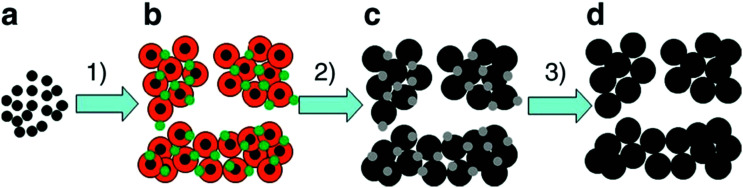
Steps: (1) synthesis of melamine formaldehyde (MF) polymer with nickel nitrate and carbon particles; (2) pyrolysing metal-salt/MF-polymer precursor; and (3) acid leaching of the pyrolysed samples. Materials: (a) carbon particles (black dot); (b) carbon particles covered with MF polymer (yellow sphere) and nickel nitrate (green dot); a sample of pyrolysed N/C material that was not subjected to acid leaching was also prepared for a reference and was termed N/C–NiO_*x*_ (c) N/C–NiO_*x*_ catalyst (grey dot, NiO_*x*_); and (d) N/C catalyst. Reproduced with permission from ref. 1375. Copyright Nature Publishing.

Nakanishi *et al.*^1375^ synthesised and investigated nitrogen-doped graphite nanomaterials (N/C) by pyrolysis of a melamine/formaldehyde polymer and nickel nitrate (Fig. 95).^1375^ An OER-based current density of 10 mA cm^−2^ was achieved at 1.61 V *vs.* RHE in 0.1 M KOH.

The location of the dopant (*e.g.*, N) within the crystal (edge, corner) influences the catalytic properties for singly-doped carbon-based nanomaterials.^1375^ Edge-selectively phosphorus-doped graphene (G-P) showed reasonable OER activity (*η* = 230 mV at *j* = 10 mA cm^−2^; 1.0 M KOH); however, the study lacks long term OER stability measurements.^1376^

**Fig. 96:**
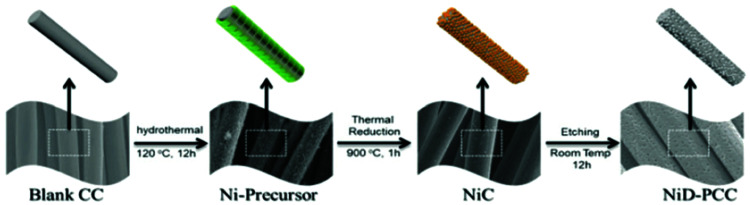
Schematic illustration of the synthesis of the monolith 3D NiDPCC. Reproduced with permission from ref. 1377. Copyright 2010 Royal Society of Chemistry (RSC).

Instead of exclusively using non-metal-containing starting materials, metal-free catalysts can also be produced from a metal-containing precursor material or on a metal-containing template if the metal component is completely removed by an etching process,.^1377–1383^ Balogun *et al.* infiltrated carbon cloth with a Ni precursor, then removed the metal content, leading to the porous carbon cloth doped with N-heteroatom (NiD-PCC) without traces of Ni (Fig. 96).^1377^ The NiD-PCC turned out to be a reasonably active anode (*η* = 360 mV; *j* = 10 mA cm^−2^; 1.0 M KOH).

In the past three years various groups studied heteroatom-doped organic frameworks as potential electrocatalysts for OER.^1384–1388^ Among the investigated materials are nitrogen doped ones^1384–1387^ as well as S,N-doped ones.^1388^ OER catalysis in acid is still demanding for non-noble metal (Fe, Mn, Co, Ni) containing anodes due to the combination of oxidative potentials and aggressive media which causes dissolving of the electrode material. Thus, particularly when OER at low pH value is intended, metal-free electrocatalysts could be a welcome alternative to transition metal based OER catalysts. Two recently reported metal-free OER electrocatalysts were investigated in acidic regime.^1385,1386^ Amino-rich carbon framework (amino-HNC), synthesised from polyaniline nanofibers, electrochemically grown on carbon paper, showed both good OER activity (*η* = 281 mV; *j* = 10 mA cm^−2^; 0.5 M H_2_SO_4_) but also high stability.^1386^ Core–shell architecture is winning strategy to improve properties of materials of different functionality and dimensionality. Carbon black//nitrogen-doped graphite core–shell structured material exhibited substantially-improved OER properties (*η* = 472 mV; *j* = 10 mA cm^−2^; 0.5 M H_2_SO_4_) relative to a nanocarbon-based electrode.^1385^

### 8.3 Bifunctional catalysts

**Fig. 97:**
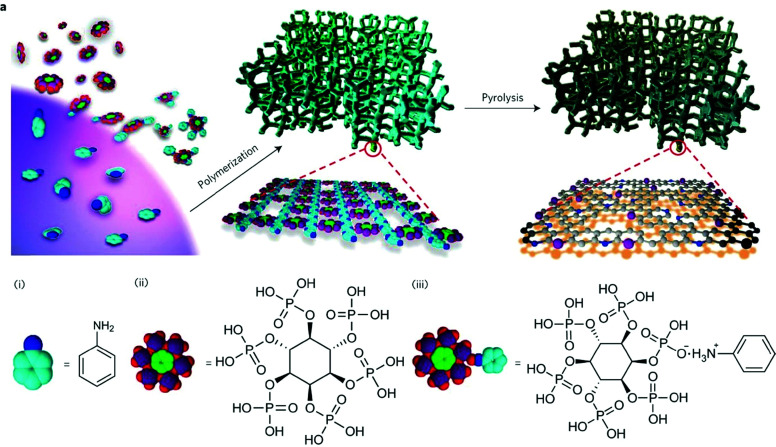
(a) Schematic illustration of the preparation process for the NPMC foams. An aniline (i)–phytic acid (ii) complex (iii) is formed (for clarity, only one of the complexed anilines is shown for an individual phytic acid), followed by oxidative polymerisation into a three-dimensional PANi hydrogel crosslinked with phytic acids. For clarity, only a piece of the two-dimensional network building block is shown in the enlarged view under the three-dimensional PANi hydrogel and only a piece of the two-dimensional NPMC network building block is shown in the enlarged view under the three-dimensional NPMC). Reproduced with permission from ref. 1389 Copyright Nature Publishing 2015.

To the best of the authors knowledge, the first metal-free bifunctional ORR and OER electrocatalysts were published in 2015.^1389^ Mesoporous carbon foam co-doped with nitrogen and phosphorous (Fig. 97) exhibits a surface area of 1.66 m^2^ g^−1^ with good ORR and OER (*η* = 270 mV at 5 mA cm^−2^ current density in 6 M KOH) performance. The most active site was identified to be N-dopant.^1390,1391^ Calculations revealed that besides N,P co-doping, graphene edges are crucial for their bifunctionality. Sakaushi *et al.* showed that a mesoporous nitrogen-doped noble carbon based on an ionic liquid can efficiently support OER and ORR in tetraethylene glycol dimethyl ether (TEGDE).^1374^

Template -based methods for the generation of bifunctional (OER/ORR) catalysts have been developed as well^1380,1383^*e.g.* by Wang *et al.* to generate nitrogen-doped mesoporous graphene framework (NMGF).^1380^ The template synthesis strategy was exploited to prepare a defective nanocarbon with B and N doped nanocarbon):^1383^ reasonable OER activity (*η* ≈ 250 mV at *j* = 10 mA cm^−2^; 1 M KOH) was achieved and its use in an air cathode resulted in low charge/discharge roundtrip efficiency and reasonable lifetime in a homemade rechargeable Zn–air battery.

**Fig. 98:**
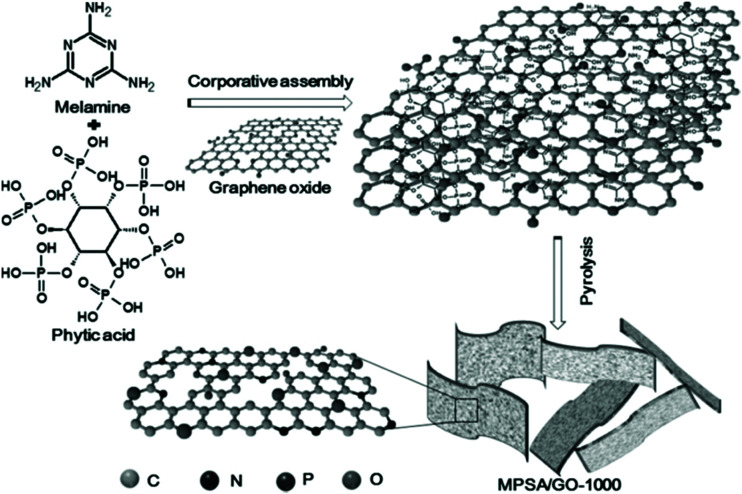
Preparation process of N,P-doped 3D porous graphitic carbon. Reproduced with permission from ref. 1392. Copyright Wiley 2016.

Metal-free catalysts which are particularly suitable for catalysing reduction reactions, *i.e.* HER or ORR have been in the focus lastly.^1334,1378,1379,1381,1392^ A bifunctional ORR/HER electrocatalyst based on porous graphitic carbons co-doped with nitrogen and phosphorus^1392^ was developed, presenting the first example of a metal-free electrocatalyst suitable to promote ORR plus HER (Fig. 98), the latter with good activity (*η* = 210 mV at *j* = 30 mA cm^−2^).^1392^

Inexpensive and naturally-abundant cellulose nanofibrils have been converted in a catalyst with reasonable (ORR/HER) bifunctionality comprising an N,S-doped carbon nanofiber network coated with N, P-doped carbon nanoparticles.^1334^ With optimised composition the catalyst exhibited onset of HER at overpotentials in the 200 mV region and demonstrated good activity: *j* = 10 mA cm^−2^ at *η* = 331 mV (0.5 M H_2_SO_4_).

Ws,N-Doped carbon nano tubes were checked for their ORR/HER properties in the same year (2016).^1378^ MnO_*x*_ nanorods have been used as a reactive template for generation of the carbon tubes *via* a wet-chemical route.^1378^ The annealed material delivered a bifunctional ORR/HER electrocatalyst which exhibited onset of HER (here defined as the overpotential for *j =* 0.2 mA cm^−2^) of 95 mV in 0.5 M H_2_SO_4_. The development of metal-free ORR/HER electrocatalysts continues to enjoy great popularity.^1379,1381,1393^ However, there is still a pronounced performance gap between the Pt-C benchmark and some of the recently developed materials.^1381^ Notably, the very recently developed ORR/HER material showed slightly better activity and stability towards HER.^1379,1393^ An interesting approach that takes (partly) usage of generally available starting material was shown by Cai *et al.*^1393^ Cigarette butts, which are mainly composed of cellulose acetate, were found to easily absorb dicyandiamide dissolved in methanol. After infiltration followed by calcination in nitrogen, porous N-doped carbon with high pyridinic N content was achieved (pyridinic N favoring hydrogen desorption). An optimised material exhibited a cathodic current density of 10 mA cm^−2^ at around 143 mV overpotential in 0.5 M sulphuric acid.^1393^ Bifunctional ORR/HER electrocatalysts that exhibit quite good HER performance in alkaline regime are rarely found. Very recently, Huang *et al.* evaluated N, O and P-doped hollow carbons synthesised using Co_2_P nanoparticles as both P source and sacrificial template; the HER activity was reasonable: *j* = 10 mA cm^−2^ at *η* = 290 mV in 1 M KOH.

Metal-free Bifunctional catalysts bifunctional HER/OER properties have also been targetted.^1335,1394–1399^ The relevance of using the same catalyst in such different conditions (strongly reductive at the negative HER electrode and strongly oxidant at the positive OER electrode) remains an open question: why would an optimised HER catalyst in terms of activity/durability would also be optimised for the OER? However, such materials will be briefly discussed below.

O,N,P-doped porous graphite carbon/oxidised carbon cloth (ONPPGC/OCC) has been recently synthesised starting from aniline, phytic acid and oxidised carbon cloth.^1399^ Full water splitting upon applying ONPPGC/OCC at both anode and cathode resulted in a cell voltage of *U*_cell_ = 1.66 V for *j* = 10 mA cm^−2^ in 1 M KOH. In addition, ONPPGC/OCC exhibited acceptable activity towards full water splitting in 0.5 M H_2_SO_4_: *U*_cell_ = 1.75 V at *j* = 10 mA cm^−2^.^1399^

Yue *et al.*^1394^ synthesised N,F-doped graphene nanosheets (NFPGNS) starting from D301 anion exchange resin, upon absorption of Na_3_Co(NO_2_)_6_ and KF. Acceptable HER activity (*η* = 330 mV; *j* = 10 mA cm^−2^; 1 M KOH) combined with acceptable OER activity was measured from steady state polarisation measurements. N-enriched polydopamine analogue was used as a carbon precursor for the generation of another OER/HER bifunctional electrocatalyst by using a spherical SiO_2_ template;^1395^ the catalyst had high pyridinic N content, and was reasonably active: *j* = 10 mA cm^−2^ at *U*_cell_ = 1.74 V; pH 14.

Pyrolysing metal-organic framework (zeolitic imidazole framework-8 ZIF-8) enables to prepare metal-free bifunctional catalysts as well. A highly N-doped (8.4 at%) carbon material with a high specific surface area was prepared that way. After cathodic polarisation treatment (CPT), N and O-containing functional groups were formed at the surface, likely explaining the satisfying electrochemical water splitting capabilities (*j* = 10 mA cm^−2^; *U*_cell_ = 1.82 V; 0.1 M KOH).^1396^

Commercial graphite powder exfoliated into graphene nanosheets and solvothermally treated in a steel autoclave followed by low temperature annealing exhibited astonishing electrochemical capabilities in 1.0 M KOH^1398^ (HER: *η* = 194 mV at *j =* 10 mA cm^−2^; OER: *η* = 304 mV at *j =* 10 mA cm^−2^).^1398^ However, the long-term performance and material's durability were not explored.

Recently, in a nucleophilic substitution reaction, 1,4 phenylenediamine and phlogoglucinol were reacted with cyanuricchloride in the presence of a base the resulting hybrid porous organic polymer (POP)^1397^ carbonised at 700 °C showed reasonable OER activity in 1.0 M KOH (*j* = 10 mA cm^−2^ at *η* = 430 mV) and samples calcinated at 900 °C exhibited reasonable HER activity (*η* = 190 mV at *j* = 10 mA cm^−2^) in 1 M sulphuric acid, obviously a result of pyridinic and pyrrolic N existing in the polymer.

As already mentioned above most of the metal-free catalysts are carbon-based ones. Exceptions are very seldom. However, profound electrochemical properties have been demonstrated for exfoliated Sb for applications in terms of energy conversion and CO_2_ fixation.^1400–1404^

Recently Ren *et al.* investigated antimonene nanosheets as potential bifunctional water (full water) splitting catalyst.^1335^ Their HER and OER performances are not satisfying: in 0.5 M KOH, *η* = 280 mV for *j*_HER_ = 1 mA cm^−2^:

It would be advantageous if one and the same electrode material could support different (desired) electrode reaction like OER, ORR and HER, possibly after conversion into different optimised catalytic active species. Recently, triple functional metal-free electrocatalysts, which enable both reduction reactions in aqueous solution (HER and ORR) and oxidation reaction (OER), have been evaluated.^1382,1405,1406^ N,P,F-Doped graphene capable to support OER, HER and ORR were accessible by pyrolysis of polyaniline-coated graphene oxide in the presence of ammonium hexafluorophosphate.^1406^ Whereas single wall carbon nanotubes (SWCNT) and C_60_ fullerene, viewed in isolation do not show any significant catalytic activity, the combination of both compounds, *i.e.* a connection implemented in a suitable manner, does.^1405^

**Fig. 99:**
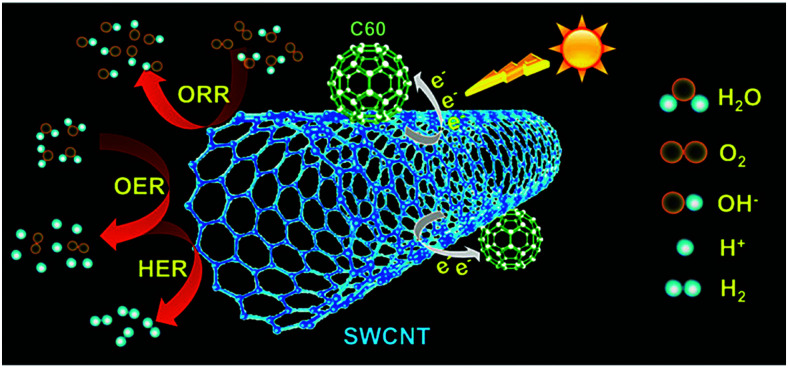
Illustration of charge-transfer process and ORR/OER/HER on C_60_-SWCNTs. Reproduced with permission from ref. 1405. Copyright American Chemical Society 2019.

Buckminsterfullerene adsorbed onto SWCNT acts as an electron acceptor, ensuring an intermolecular charge transfer; this results in the formation of a triple functional (HER, OER and ORR), solely carbon-based material (Fig. 99) with reasonable activity for OER (*η* = 460 mV; *j* = 10 mA cm^−2^; pH 0) and HER (*η* = 380 mV; *j* = 10 mA cm^−2^; pH 13).

### 8.4 Carbon-nitrogen based catalysts with high N content

**Fig. 100:**
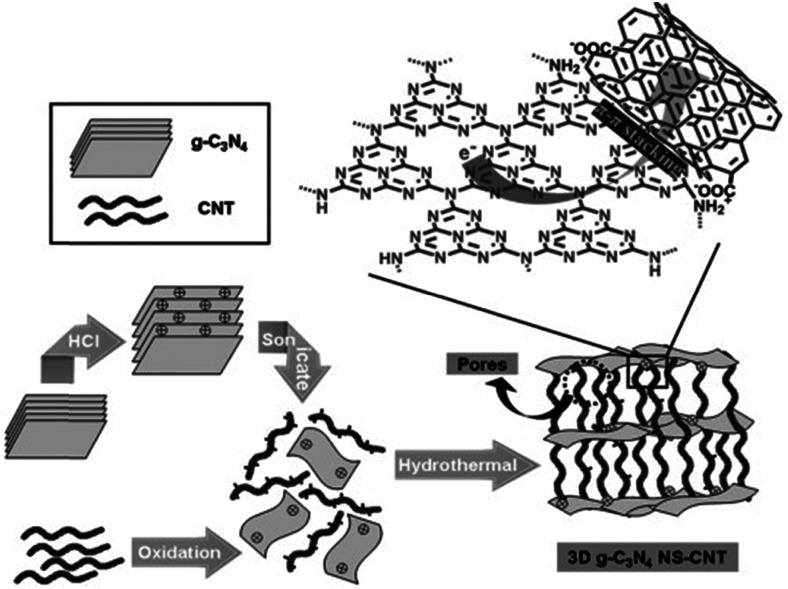
Fabrication of the 3D g-C3N4 NS-CNT porous composite. Reproduced with permission from ref. 1410. Copyright Wiley 2014.

Graphitic carbon nitride (g-C_3_N_4_) is one of the oldest reported artificial polymers in the scientific literature. The use of g-C_3_N_4_ in heterogeneous catalysis began about 15 years ago in 2006.^1407^ It combines high nitrogen content with high chemical and thermal stability. However, g-C_3_N_4_ is known to have extremely low conductivity^1408^ and bulk samples show a rather low density of catalytic active sites. It was, however demonstrated that g-C_3_N_4_ nanosheets/graphene composites or g-C_3_N_4_ nanosheets/carbon nanotube composites (Fig. 100) can be OER active^1409,1410^ (*η* = 400 mV at *j* = 20 mA cm^−2^ in 0.1 M KOH),^1410^ (*η* ≈ 800 mV at *j* = 35 mA cm^−2^; 0.1 M KOH),^1409^ respectively. Unfortunately, long-term stability towards OER over a significant period (>10 h duration) has not been proven, and the intrinsic susceptibility for carbon to corrode in OER regime makes the authors suspicious that the catalyst is durable in operation (see above). A more complex hybrid material (S-doped carbon nitride/carbon nanotube/carbon fibre^1411^) was recently shown^1411^ with reasonable OER/HER activity in 1.0 M KOH (*U*_cell_ = 1.8 V at *j* = 10 mA cm^−2^).

Poor contact between graphitic carbon nitride fragments and carbon and the resulting inhomogeneity may be overcome by choosing alternative routes to C_3_N_4_ based polymers, for instance based on a single carbon-nitrogen sources such as guanidine hydrochloride.^1412^

Doping of graphitic carbon nitride matrix with P or S or P and S was indeed found to be an effective way to manipulate electronic structure and electrochemical properties.^1413,1414^ In their report Lee *et al.* describe a theoretical structure-activity relationship in g-C_3_N_4_ for OER and ORR electrocatalysis based on an understanding of the effects of dopants considering the possible reaction pathways based on the Eley–Rideal mechanism.^1415^ For X_Y_-C_3_N_4_ (where X and Y indicate the dopant and doping site on C_3_N_4_, respectively), P_C_S_C_–C_3_N_4_ (C_3_N_4_ with P and S codoped at the carbon site) shows better bifunctional performance of OER/ORR with competitive overpotentials at 0.42 and 0.27 V, respectively, compared to conventional Pt and RuO_2_ catalysts.

## 9 Concepts for electrode preparation

In water electrolysis, as in any electrochemical processes, electrocatalysts are used to increase the charge-transfer kinetics and to maximise the energy efficiency of the redox processes taking place at the interfaces. The electrochemical performance of the catalytic layers essentially depends on three factors: the catalysts’ intrinsic electrochemical activity, deployed electrochemical surface area (ESCA) and accessibility to reactants and products, the latter depending more on active layer engineering than on electrocatalyst engineering. Research into materials with optimised electrocatalytic properties therefore requires, on the one hand, to measure the intrinsic electrochemical activity of each half-cell reactions of interest and, on the other hand, nano-structuring so to maximise the surface area of the electrocatalytic particles|electrolytes interface (and the material texture to make it compatible with fast mass-transport in the active layer). Nano-structuring can be obtained using different manufacturing processes. This section describes methods and techniques to manufacture electrocatalysts and electrodes used in water electrolysis applications: electrodeposition, chemical precipitation, self-assembly, atomic layer deposition, physical vapour deposition, spray pyrolysis, ultrasonic spraying *etc.*, all enable to tailor the materials electrocatalytic and mass-transfer properties.

### 9.1 PEM water electrolysis

#### 9.1.1 Preparation of OER catalysts

##### 9.1.1.1 Conventional OER oxides

**Fig. 101:**
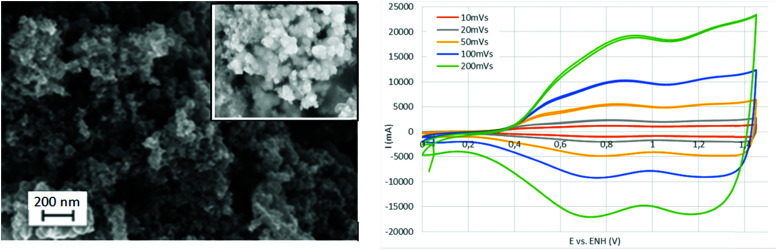
(left) SEM micrographs of unsupported IrO_2_ nanoparticles used at the anode of PEM water electrolysis cells. (right) *In situ* cyclic voltammograms recorded on IrO_2_ at the anode of a PEM water electrolysis cell, at different scan rates. Reproduced with permission from ref. 116. Copyright Elsevier 2016.

Noble metal oxides have been used in electrochemistry since the 1960's. Iridium oxide (IrO_2_) and ruthenium oxide (RuO_2_) have been widely employed in the chlor-alkali and chlorine industry, in the so-called dimensionally stable anodes (DSA®). They are also used as electrocatalysts at the anode of PEMWE in the form of unsupported oxide particles (IrO_2_, RuO_2_ or their solid solutions^1416^). They have metal-like electronic conductivity (6 × 10^−5^–5 × 10^−5^ Ω cm), a feature resulting from their electronic structure.^1417^ However, the risks associated with the possible formation of higher ruthenium oxides (volatility and toxicity) as well as the poorer stability of Ru and RuO_2_*versus* Ir and IrO_2_^1796^ have so far led to a preference for the use of IrO_2_ alone in PEMWE. These oxides are synthesised by calcination of precursor salts. The synthesis of PtOx by fusion of chloroplatinic acid and sodium nitrate (300–600 °C) has been first described by R. Adams.^1418^ The same method can be used to synthesise IrO_2_ and RuO_2_ or their solid solutions.^1419,1420^ In a preferred manner, IrO_2_ can be synthesised from H_2_IrCl_6_·H_2_O mixed with NaNO_3_ in aqueous solution, dried, grinded, and preheated at 350 °C for 1 h. The optimum condition for IrO_2_ synthesis is a calcination temperature of ∼550 °C, using a mass ratio of H_2_IrCl_6_·H_2_O to NaNO_3_ of 1 : 20. The resulting IrO_2_ electrocatalyst has a high OER activity (90 mA cm^−2^ at +1.5 V *vs.* RHE), a high crystallinity (90%) and a large specific surface area (126 m^2^ g^−1^).^1421^ Unsupported IrO_2_ shows high electroactivity and stability (practical applications require operation in the upper range of the 50–80 kh interval). Scanning electron microscopy (SEM) images of nano structured IrO_2_ are shown in Fig. 101 (left). Fig. 101 (right) shows typical cyclic voltammograms (CVs) measured *in situ*, using the cathode of the cell as reference and counter electrode simultaneously (see Section 11). The underpotential deposition and desorption of hydrogen ad-atoms takes place at potentials lower than +0.4 V *vs.* RHE. At potentials above, the peaks are attributed to the Ir(iii)/Ir(iv) and the Ir(iv)/Ir(v) redox couples.^1422^

Other synthesis methods have also been reported in the literature. IrO_2_ nanoparticles (NPs) can be synthesised using wet-chemical processes. For example, the nanoparticles can be prepared by reducing metal chlorides in ethylene glycol using PVP as a capping agent, then annealed in air at 400 °C. In that case,^1423^ a specific activity of up to 3.5 μA cm^−2^ of oxide was reported at +1.53 V *vs.* RHE. Rutile–IrO_2_ NPs were also prepared by first synthesising metallic Ir NPs in an organic solution followed by air oxidation. An intrinsic OER mass activity (the current per gram of catalyst) of 10 A g_oxide_^−1^ was reported at +1.48 V *vs.* RHE.^25^

##### 9.1.1.2 Supporting oxides

The price of iridium and the significant fluctuations in its price on the raw materials market challenges the development of the PEMWE. Efforts have been made among the scientific community to find OER alternatives to IrO_2_, but the task is very challenging and, to date, no real solutions to this problem have been proposed. Most research efforts focus on the reduction or Ir loadings (a factor of ten is targeted compared to 2.0 mg cm^−2^ loadings commonly used; ∼ 1.0 mg cm^−2^ is already common good practice at the industrial scale). Different approaches have been investigated and reported in the literature. Conventional OER electrocatalysts are made of micrometre sized IrO_2_ particles. Since 90% of the atoms of a 1 nanometre-sized cuboctahedral Ir particle are exposed at the surface, the cost issue could be alleviated by decreasing the size of the anodic electrocatalyst, assuming that the increased adsorption strength of oxygenated species on the smallest nanocrystallites does not significantly lower their intrinsic OER activity and stability. Decreasing the IrO_2_ crystallite size to *ca.* 5–15 nm is required to improve both OER mass activity and stability, while leading to a drastic reduction of the Ir content at the anode of a PEM water electrolyser.

The synthesis and use of self-standing nanometric IrO_2_ particles cause several problems. Their implementation at the anode of PEM water electrolysis cell can be achieved by using appropriate electron-conducting supports having (i) a large specific surface area to maximise the distribution of the nanoparticles (NPs) while preventing their agglomeration/aggregation, (ii) an optimal pore size distribution to allow easy access of reactants to the electrode and products removal from the electrode. The catalyst support should also withstand high electrochemical potentials (+1.8 to +2.1 V *vs.* SHE), highly acidic environment and moderate operating temperature (<80–90 °C). Carbon blacks, the usual catalyst support in PEMFCs,^1424^ and any types of carbonaceous structures are strongly unstable in PEMWE anodes (carbon is oxidised at potentials above +0.207 V *vs.* SHE) and cannot be used for that purpose. On the contrary, antimony-doped tin dioxide (ATO) substrates (aerogels or nanotubes) have more chances to meet these requirements.^444,1420,1425,1426^ They offer a large specific surface area and allow fast mass transport at high current density, a field in which PEM electrolysers outperform their alkaline counterparts. Moreover, their morphology is amenable to the specifications of PEM water electrolysis and their electronic conductivity can be tuned depending on the nature and concentration of dopant. This field of research is still very active, and works are in progress to improve the electrochemical stability of such materials, *e.g.*, by tailoring their doping: while Sb-doped SnO_2_ type supports were shown to non-negligibly dissolve, Ta-doped or Nb-doped SnO_2_ supports with appropriate dopant concentrations were found more stable under acidic OER conditions.^34^ Scott *et al.* showed that the Nb_2_O_5_ addition to RuO_2_ was found to increase the stability of RuO_2_ and in some cases performance was improved. In this work, a bimetallic RuO_*x*_-Nb_1−*x*_O_2_ catalyst was prepared as an anode catalyst for the OER using Adams and hydrolysis methods.^1427^

Recently, a one-step organometallic chemical deposition (OMCD) method was reported to prepare a crystalline iridium oxide nanoparticle of 2.3 nm on antimony-doped tin oxide. In comparison to a commercial IrO_2_–TiO_2_ benchmark, the crystalline IrO_2_ showed a 7-fold increase in Ir mass-specific activity, as well as excellent stability.^1428^

**Fig. 102:**
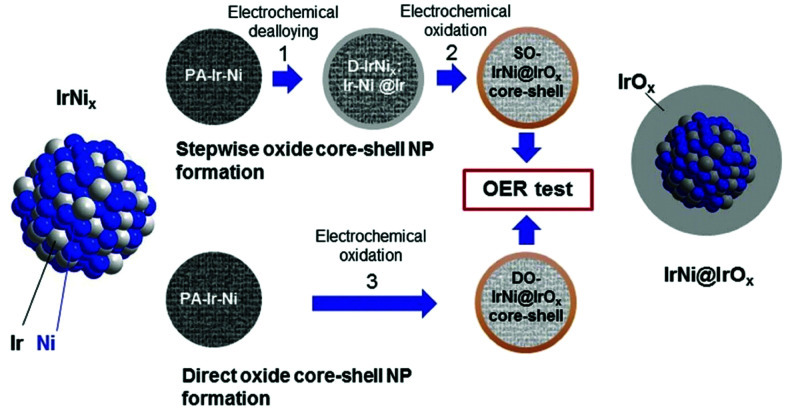
Overview of the protocol used for the synthesis of SO-IrNi@IrO_*x*_ and DO-IrNi@IrO_*x*_ hybrid core–shell nanoparticle catalysts. Reproduced with permission from ref. 1429. Copyright. RSC 2014.

The development of core@shell structures composed of a highly active and durable metal oxide (IrO_2_) shell, covering a cheaper and more abundant transition metal (such as cobalt, nickel, copper), is another option for reducing Ir loadings but stability problems and risks of corrosion and dissolution in PEMWE conditions have led to limited progress so far. An example is described in Fig. 102.^1429^

**Fig. 103:**
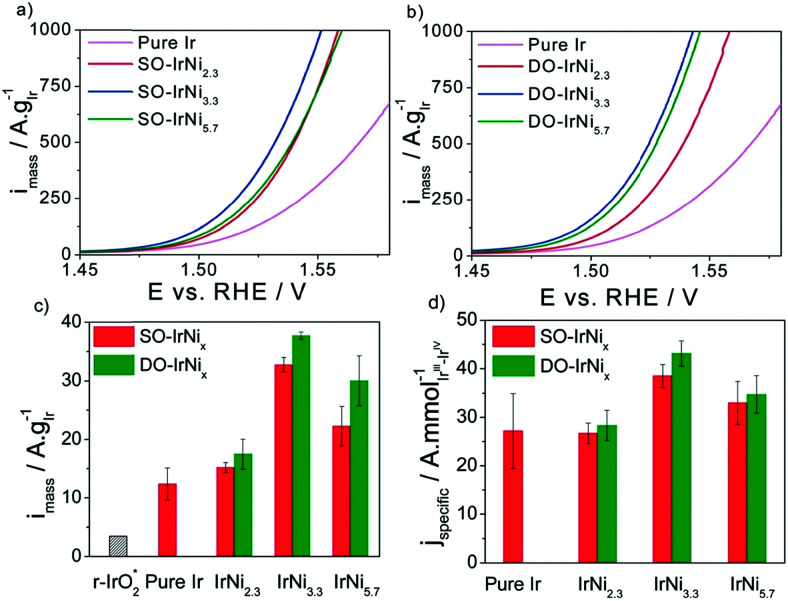
(a and b) sweep voltammetry and catalytic oxygen evolution reaction (OER) activities of stepwise oxidised (SO) IrNi_*x*_ and directly oxidised (DO) IrNi_*x*_ core–shell nanoparticles, compared to pure Ir nanoparticles. (c) Ir mass-based activities and (d) specific activities at 0.25 V overpotential. Reproduced with permission from ref. 1429. Copyright RSC 2014.

In this example, IrO_*x*_ core–shell nanocatalysts were prepared using a two-step procedure:(i) synthesis of supported IrNi_*x*_ bimetallic nanoparticles (a previously-documented polyol process, involving 1,2-tetradecadiol as a reducing agent and oleylamine and oleic acid as capping ligands, was used to make Ni rich Ir–Ni bimetallic NPs); (ii) preparation of IrNi@IrOx hybrid core–shell catalysts. To make dealloyed metallic core–shell NPs (“D-IrNi_*x*_”), the IrNix NP precursor alloys (PA-IrNix) were first electrochemically dealloyed. Selective surface-oxidation led to stepwise-oxidised (SO) metal oxide core–shell NPs “SO-IrNi@IrO_*x*_” of high mass activity (Fig. 103a). Alternatively (Fig. 103b), DO-IrNi@IrO_*x*_ NPs were directly obtained by coupling dealloying/oxidation steps (“DO-IrNi_*x*_”). The SO-IrNi_*x*_ or DO-IrNi_*x*_ nomenclatures emphasise the parent precursor alloy's stoichiometry, while the SO-IrNi@IrO_*x*_ and DO-IrNi@IrO_*x*_ nomenclatures emphasise the chemical core–shell structure. The particles thus obtained have an almost pure and nanometre-thick surface layer of IrO_*x*_. The inner central zones are more metallic and enriched in Ni. Interestingly (Fig. 103c and d), the OER activity of these core–shell particles is 3 times greater than that measured on the reference catalysts (IrO_2_ and RuO_2_). The turnover frequency (TOF) of the most active IrNi@IrOx catalysts is greatly increased. This concept of core–shell nanoparticles is quite general and can potentially be applied to the synthesis of other noble metal nanoparticles, paving the way for nanostructured PEMWE electrodes with significantly-reduced noble metal contents.

#### 9.1.2 Preparation of HER catalysts

**Fig. 104:**
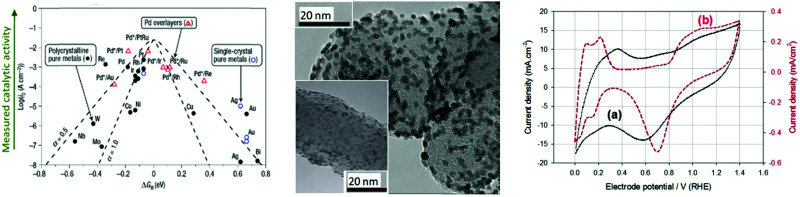
(a) Volcano plots of *j*_0_*vs.* M–H bond energy. (b) SEM micrographs of nano-Pt/C electrocatalysts for the HER. (c) Cyclic voltammograms measured (a) on metallic Pt in 1 M H_2_SO_4_; (b) *in situ* at the cathode of a PEM water electrolysis cell. Reproduced with permission from ref. 1431 Copyright Elsevier 2014.

Due to its very high charge-transfer kinetics and reversibility, the HER reaction in aqueous acid media is probably the most studied and documented electrochemical reaction. The selection of HER electrocatalysts is facilitated by considering volcano plots of the exchange current density *j*_0_ as a function of the energy of the metal-H (M–H) bond (Fig. 104a). The binding energy of intermediate hydrogen ad-atoms plays a critical role in the HER kinetics and platinum is the most efficient catalyst, at least in acids. In the early days of PEMWE (the 1980s), unsupported Pt nanoparticles were used at the cathode of PEMWE cells. These Pt nanoparticles were either synthesised separately and then coated onto the polymer membrane, or synthesised directly onto the polymer, usually by chemical reduction of precursor platinum salts such as hexachloroplatinic acid, using soft chemical reducers (NaBH_4_, H_2_).^1430^ For cost and environmental reasons, efforts have been made to reduce Pt loadings.^1269^ Over the past decades, progress made in PEMFC technologies led to the development of quite efficient Pt/C catalysts which can also be used for the HER in PEMWE cells (Fig. 104b). Cyclic voltammograms (CVs) recorded *in situ* (such measurements require the implementation of a reference electrode, see Section 11) are a bit distorted but similar in shape to those recorded in liquid acid electrolytes^1431^ (Fig. 104c).

##### 9.1.2.1 Preparation of Pt/C cathode catalyst

**Fig. 105:**
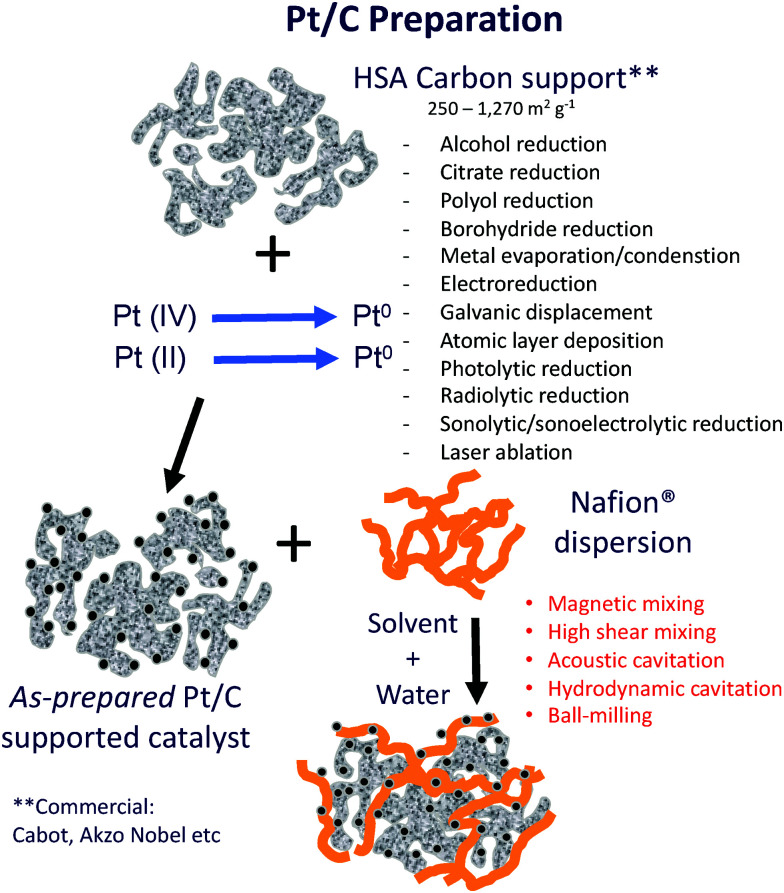
Various routes for preparing PEMFC, and PEMWE Pt/C catalyst and catalyst ‘inks’.

There are several methods to prepare Pt/C catalysts and can be categorised as chemical and physical routes (Fig. 105). The chemical routes usually involve the reduction of Pt(ii) or Pt(iv) salts on high surface area carbon substrates (250–1270 m^2^ g^−1^, Cabot, Akzo Nobel *etc.*) by the polyol, borohydride, alcohol, and citrate reduction and metal evaporation, metal condensation, LASER ablation methods as well as electrodeposition and galvanic displacement, whilst the physical methods entails atomic layer deposition, photolytic, radiolytic, sonolytic and sonoelectrolytic reduction.^1432^

Different carbon substrates, different platinum precursors and different techniques can be used for the synthesis of appropriate Pt/C HER electrocatalysts, which are now commercially available (Johnson Matthey Fuel Cells, Tanaka, Umicore, HySA, *etc.*). Impregnation/reduction techniques are commonly used. For example, carbon particles can be soaked in Pt(NH_3_)_2_(NO_3_)_2_ solutions, evaporated to dryness and then decomposed in air (typically at 260 °C for a few hours). Colloidal suspensions of platinum can also be adsorbed on carbon,^1433^ and there are many techniques to perform the so: Bonnemann,^1434^ polyol,^1435^ water-in-oil^1436^ are typical colloidal methods that are widely employed to elaborate of low-temperature fuel cells and electrolysers catalysts (they are not specific to PEMWE catalysts). Impregnation-reduction, an also widely employed technique to prepare Pt/C for PEMFC applications^1437^ (in this case with an electrochemical reduction), was used to coat Pt nanoparticles onto multi-wall carbon nanotubes (MWCNT). This was achieved by using hexachloroplatinic acid (H_2_PtCl_6_) as precursor Pt salt and formaldehyde as reducing agent. After 20 minutes of impregnation, the mixture was heated to 80 °C and stirred for 3 h. Because of the lower corrosion rate of highly graphitised MWCNT and the improved contact between metal nanoparticles and carbon support, the resulting catalyst exhibited higher electrochemical stability.^1438^

Atomic layer deposition (ALD) of platinum has also been reported to improve the HER performance of Pt-based catalysts immobilised on functionalised Vulcan carbon. Compared to the industrial 20 wt% Pt/C catalyst, the 7.1 wt% Pt-based composite catalyst exhibited significantly-increased HER activity and stability.^1439^ Improvements are usually driven by research on PEMFC. Interesting results were obtained by inserting platinum nanoparticles (PtNPs) into shortened hollow graphitised carbon nanofibers (PtNP@SGNF): they achieve unprecedented electrochemical stabilisation for oxygen reduction reactions in fuel cells. Unlike commercial Pt/C electrocatalysts, the basic activity and electrochemical surface area of PtNP@SGNF remains unchanged after 50 000 potential cycles during durability tests.^1440^ The stability of highly-graphitised carbon nanotubes supports (heat treatment at 2800 °C) improves the durability of platinum catalysts.

**Fig. 106:**
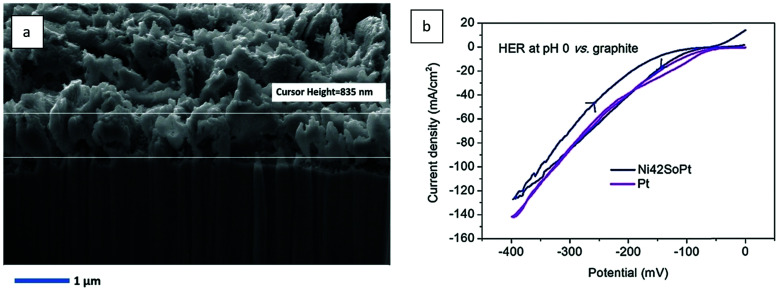
(a) SEM photograph showing the cross-section of the Ni_42_/Pt interface (Pt thickness ∼800–900 nm); the Pt loading is 1.8 mg cm^−2^. (b) comparison of the HER performances of Pt and Ni_42_SoPt at pH = 0. Reproduced with permission from ref. 1269. Copyright Wiley 2019.

Another approach reported in the literature is to deposit reduced amounts of platinum on cheap (*e.g.*, stainless steel) substrates. The easy contamination of working electrodes with trace amounts of platinum, when Pt counter electrodes are used in three electrode cells, is well-established. This can happen quite easily, during the evaluation of the HER electrochemical activity of non-PGM based materials. However, such experimental setup's flaw can be turned into a beneficial effect to develop highly active and stable HER electrocatalysts. This can be achieved by electrochemical etching of platinum using a platinum anode:^1269^ electrodeposition of platinum occurred at the surface of Ni_42_ steel (106a) during repeated HER CV scans in sulphuric acid (Fig. 106), Pt coming from the (desired) progressive dissolution of the Pt counter-electrode (on which OER is the dominant reaction). The HER which forms hydrogen bubbles interferes with the electro-crystallisation of platinum on Ni_42_ steel and this leads to porous Pt layers of large specific area. The process can be assisted by ultrasonication, which is beneficial in terms of activity and stability.

Apart from the chemical approach, it has been shown recently that Pt/C can be produced by using ultrasound in the presence and absence of electrochemistry.^1432^ Ultrasound produces H˙ (and OH˙) radicals *in situ* acting as reducing agents for the production of Pt NPs^1441^ and Pt/C in Nafion®.^1442^

##### 9.1.2.2 Non-conventional HER catalysts

**Fig. 107:**
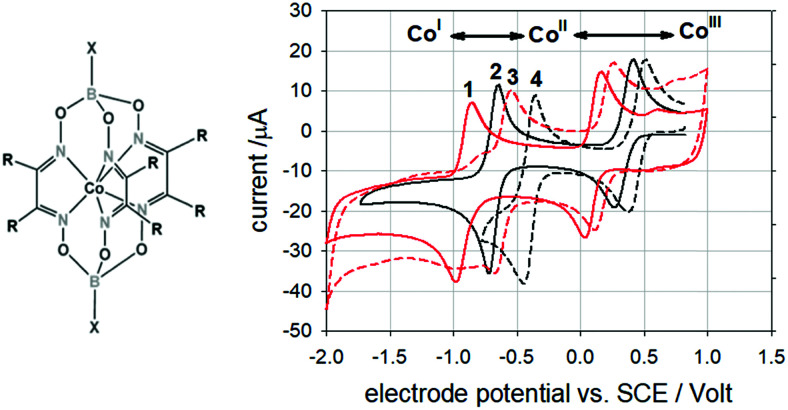
(a) General chemical formulae of cobalt clathrochelates. (b) Cyclic voltammograms recorded on three different cobalt clathrochelates in acetonitrile (10 mV s^−1^). Complex 1: X = *n*-butane and R = cyclohexane; complex 2: X = F and R = methyl group; complex 3: X = *n*-butane and R = phenyl group; complex 4: X = F and R = phenyl group. Reproduced with permission from ref. 1443 Copyright Wiley 2008.

The Pt content used at PEMWE cathodes is low (<0.1–0.2 mg_Pt_ cm^−2^) but nevertheless contributes to the higher cost of PEMWEs compared to A(EM)WEs. In addition, platinum is extremely sensitive to the presence of impurities (organic or inorganic), which imposes severe constraints on the management of the water purity used in PEMWEs. So, the search for alternatives to platinum remains a subject of interest for the scientific community and the industry. The idea is to replace platinum with transition metals (Ni, Co, Fe), the same as those used in alkaline water electrolysis. In alkaline media, passivation of these metals prevents their dissolution. Hence, they cannot be used directly in acid media, where these metal oxides are not sufficient durable. Molecular chemistry offers some interesting solutions to such problem. Cobalt, nickel, and iron ions can be introduced in inorganic cage-like organic structures such as the clathrochelates shown on Fig. 107a.^1443^ In homogeneous solution, these complexes are chemically stable, and the redox properties of the central metallic center can be tuned by selecting appropriate peripherical radicals of various electro-attractive effects. With cobalt as active center, two redox waves (corresponding to Co^III^/Co^II^ and Co^II^/Co^I^ redox couples) are observed (Fig. 107b). The position of the current peaks along the potential axis can be significantly shifted towards more positive potentials (to favour the HER) when peripheral radicals of increasing electro-attractive strengths are used as substituents.

Such complexes and other molecular-type catalysts have been widely studied on the fundamental side (see Sections 10 and 11). They can be implemented at the cathode of PEM water electrolysis cells after adsorption at the surface of appropriate substrates such as carbonaceous compounds, commonly used to support Pt nanoparticles. The main difficulty remains despite everything the functionalisation of these catalysts on these substrates to form practical electrodes. At the laboratory scale, in order to evaluate the electroactivity of these molecular materials, simple functionalisation techniques (*e.g.* physisorption by impregnation,^1444,1445^ or by ultrasonics^1446^) can be used. They generally yield thin layers, which give satisfactory results but do not guarantee lifespans compatible with the targeted applications. More sophisticated functionalisation techniques are needed to produce highly active and stable monolayers. Electro-grafting is an interesting technique to use for that purpose. A two-step procedure consisting of (i) the electro-grafting of a monolayer of a diazonium derivative onto a carbonaceous substrate of interest and (ii) the chemical grafting of the compounds of interest onto the surface by simple chemical reaction, is commonly used. The technique has been used for electro-grafting of a cobalt clathrochelate containing carboxylic end-groups:^1447^ a monolayer-thick deposit is obtained, corresponding to very low metal loadings (in the pg cm^−2^ range).

##### 9.1.2.3 Thiomolybdate compounds

In the quest for HER electrocatalysts sufficiently active and stable in acid electrolytes in PEMWE, encouraging results have been obtained with thiomolybdate compounds (molybdenum is a hundred times more abundant in the Earth crust than Pt).^1448^ MoS_2_ nanocrystallites were found active towards the hydrogen dissociation reaction in the 1980s (in the field of Hydrodesulphurisation in the oil & gas industry). Molybdenum sulphur-based catalysts were also found active for the reverse hydrogen oxidation reaction (HOR) and over the last years, for hydrogen formation in electro- or photo-catalysis HER processes. Sulphur-enriched clusters (e.g, [Mo_3_S_13_]^2−^), are highly stable in acidic media and have been reported to exhibit a HER activity comparable to those of PGMs such as Pt.^1449^ Tests and results obtained under real PEMWE conditions are scarce, but the preliminary ones reported in the literature show that replacing platinum induces a cell voltage increase by approximately 250 mV in the activation area: *U*_cell_ = 2.0 V is reached at only *j* = 500 mA cm^−2^ compared to 1.5 A.cm^−2^ with Pt. The compounds are chemically stable and reasonably HER electroactive (this is encouraging) but the level of performance obtained with such catalytic systems based on {MoS} is too low to consider them as good candidates to replace Pt for the HER in industrial PEMWEs.

#### 9.1.3 Manufacturing of membrane electrode assemblies

Generally, the catalyst ink (Pt/C or other catalyst-supported catalyst + IPA + water + ionomer) may be either deposited to the gas diffusion layer (GDL) to yield a gas diffusion electrode (GDE), also known as catalyst-coated substrate (CCS) or the polymeric proton exchange membrane (*e.g.*, PFSA, *n*-PBI *etc.*) to form a catalyst-coated membrane (CCM). CCSs are usually prepared by screen-printing, hand-painting, ink-jetting, spreading, spraying (air and ultrasonic), (electro)deposition, ionomer impregnation, and sputtering. CCMs are produced by decaling, screen-printing, hand-painting, spraying (air and ultrasonic), impregnation/reduction, evaporation/deposition, sputtering, and dry spraying.

The electrodes used in PEMWEs have the particularity of being very thin (a few microns) and porous (they must allow the gases produced to pass and allow water to access the catalytic sites). In the literature, they are rather designated by the terms “catalytic layers” or sometimes “thin-film electrodes”. This is a porous mixture essentially containing the catalyst particles and the ionomer ensuring their ionic contact with the membrane. The composition and microstructure of these CLs are critical since they dictate the overall cell efficiency, and, to a large extent, its durability. The catalytic ink can be deposited either on both sides of the membrane to form a self-standing CCM, or onto an external substrate (CCS) which is then pressed against the membrane. The term membrane electrode assembly (MEA) is also commonly used.

**Fig. 108:**
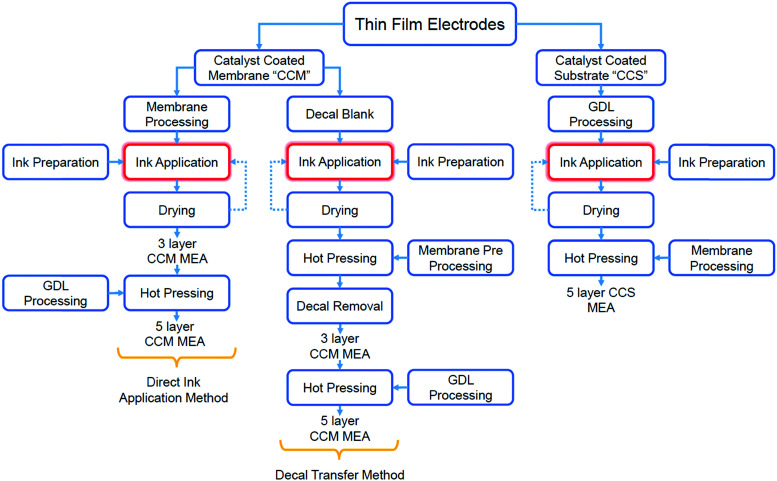
Overview of multi-steps processes used for CLs, CCMs, and MEAs manufacturing for PEM fuel cells.

A large number of processes have been reported in the literature to form or coat catalyst particles directly onto (PFSA) membranes, mainly to perform laboratory tests. Electroless plating has been very popular for a long period of time. For example, hexachloroplatinic acid can be chemically reduced onto the membrane by cross-permeation of a chemical reducer such as sodium borohydride.^1450^ Alternatively, the membrane can be first soaked into a solution of the chemical reducer for impregnation and then into a solution of the platinum precursor salt or be soaked in an aqueous solution containing a cationic species of platinum precursor before chemical reduction. By adjusting operating conditions, thin Pt layers deeply anchored onto the membrane are obtained.^1430^ Such processes are less interesting for the coating of iridium-containing anodes, though electrochemical coating has also been reported in that case.^1451^ CCS manufacturing is widely used in fuel cell technology (Fig. 108).

**Fig. 109:**
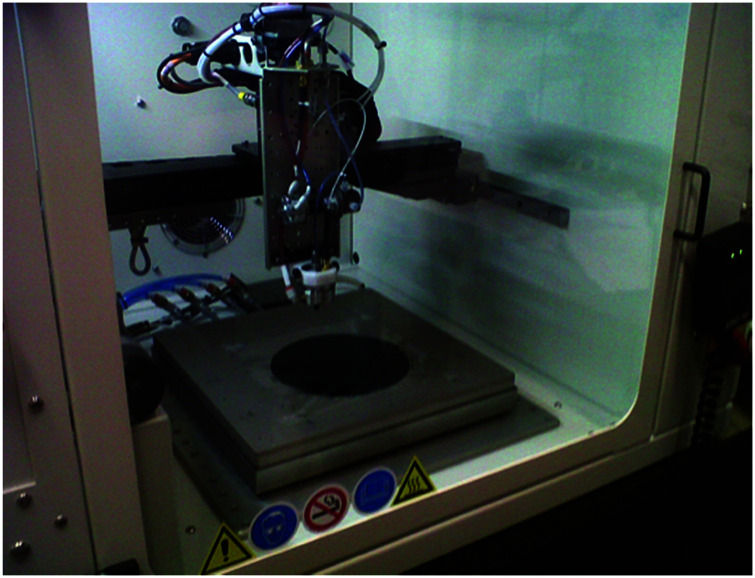
Photograph of a catalytic ink printer used to spray catalytic inks onto PFSA membranes (batch process, lab-scale). Reproduced with permission from Paris-Saclay University.

**Fig. 110:**
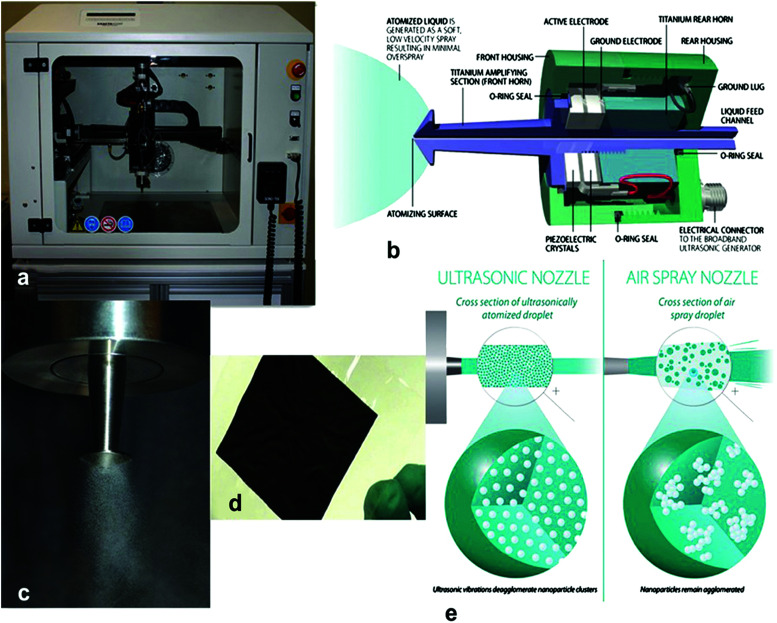
(a) Sono-Tek Ultrasonic Spray system—‘ExactaCoat’; (b) representation of the vibrating nozzle cross-section; (c) mist formation of the liquid schematic; (d) CCMs for PEMFC, DMFC, and PEMWE manufactured by the Sono-Tek system; (e) representation of nanoparticle de-agglomeration *via* the ultrasonic-spray method *vs.* the air spray method. Reproduced with permission from ref. 1432. Copyright MDPI 2019.

In early PEMFC applications, the platinum nanoparticles (used at the hydrogen anode and the oxygen cathode) were deposited on carbon GDLs to form GDEs, which are then pressed against the membrane. The need for large volume manufacturing and quality-monitoring has led to the narrowing down of available technologies for the development of automated coating processes, and particularly in the elaboration of CCMs;^1452^ this even more applies to PEMWE materials. In the latter case, catalyst particles (Pt/C for the cathode and IrO_2_ for the anode) are usually synthesised *ex situ* and then coated onto the membrane to form a CCM. There are basically two main options. The catalytic inks (a mixture of catalyst particles and ionomer in a solvent) are sprayed either directly onto the membrane, or onto a PTFE substrate and then transferred onto the membrane by hot pressing (so-called decal or electrode transfer method). This is performed using a catalytic ink printer, usually equipped with an ultrasonication nozzle to maintain the particle of catalyst in suspension, such as the one shown in Fig. 108. Direct spray onto the membrane is simpler (Fig. 109) but care must be taken to avoid solvent impregnation into the membrane and its detrimental swelling, favouring expansion/contraction upon the CCM elaboration process, hence possible destabilisation of the active layer (cracks, delamination). In that context, the decal method is interesting because solvent can be evaporated before transferring the electrodes to the membranes. Alternatively, magnetron-sputtering can also be used to form the particles onto a substrate.^1453^ Automated and continuous roll-to-roll manufacturing processes now commonly-used in PEMFC technologies are also becoming available for PEMWEs (Fig. 110).

### 9.2 Alkaline water electrolysis

Most of the previous techniques and materials can also be used in alkaline water electrolysis,^40,1454,1455^ (see Sections 3.1.1 and 5.1). However, the alkaline medium renders possible the use of non-PGM catalysts in AWE, for which the preparation methods can sharply differ. The present section highlights some of these differences, being admitted that the huge diversity of A(EM)WE catalyst/electrode materials does not enable isolating standardised strategies for their preparation/assembly.

#### 9.2.1 Preparation of OER catalysts

**Fig. 111:**
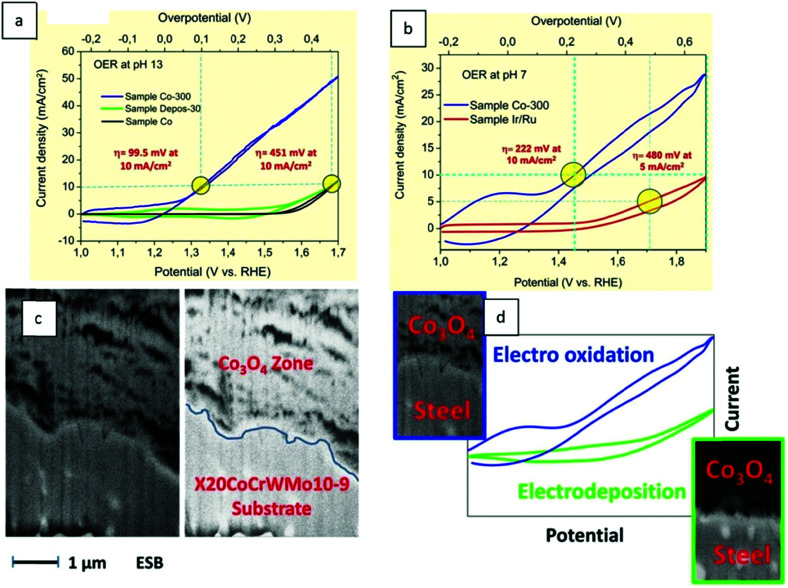
(a) Comparison of the electrochemical OER properties of sample Co-300 with sample Ir/Ru and sample Depos-30 in pH 13 (b) and pH 7. (c) SEM micrograph of a FIB machined cross section of sample Co-300. (d) A diagram represents difference between sample Co-300 (electro oxidation) and sample Depos-30 (electrodeposition) as a function of OER properties. Reproduced with permission from ref. 40. Copyright RSC 2016.

Bulk (standalone) electrodes are possible in A(EM)WE, thanks to the use of non-PGM catalysts. Steels are example, provided they are properly activated. The electro-activation of a Co tool steel, X_20_CoCrWMo_10-9_, resulted in a new composite material (X_20_CoCrWMo_10-9_/Co_3_O_4_ half-cell reaction of water electrolysis) with previously unmatched effectiveness^40^ (Fig. 111). Electrocatalytic properties, observed not only at pH 7 corrected with 0.1 M phosphate buffer, but also at pH 13, were far superior to those of single-phase IrO_2_–RuO_2_, Co_3_O_4_, or Fe/Ni-based catalysts (Fig. 111a and b). Co_3_O_4_ was identified as the dominant compound on the surface of the X_20_CoCrWMo_10-9_/Co_3_O_4_ by XPS and FTIR experiments. The composite does not correspond to the traditional substrate intrinsic formation of the Co-enriched outer layer. For comparison purposes, the author prepared electrodeposited Co_3_O_4_ on stainless steel (sample Depos-30) by a two-step electrochemical approach has been used to coat the substrate, consisting of (I) electrodeposition of Co(OH)_2_ and (II) electrochemical oxidation of Co(OH)_2_ to Co_3_O_4_ (Fig. 111c). Thus, although the surface composition of Depos-30 and Co-300 is comparable, the OER efficiency is not (Fig. 111a and b), and the high catalytic activity of sample Co-300 cannot be explained solely by the fact that its “outer sphere” is primarily made up of Co_3_O_4_. Since the base material in both samples is the same (X_20_CoCrWMo_10-9_), it is likely that the conditions and the distance between the substrate and the surface significantly impact the material's ability to act as a good OER electrocatalyst.^1324^

**Fig. 112:**
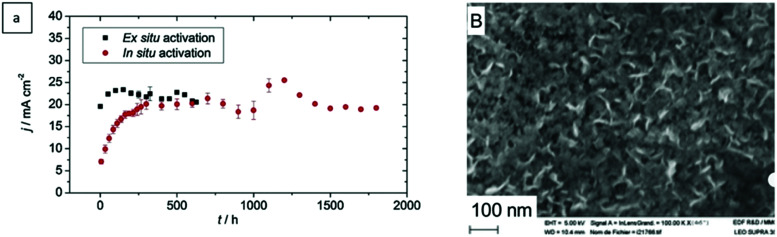
Comparison of the 316L SS electrodes OER (test conducted at *E* = +1.75 V *vs.* RHE) after accelerated activation and the *in situ* activated 316L SS electrodes OER in 5.0 M LiOH at *T* = 25 °C (A). SEM of the surface of the 316L SS electrode activated (B). Reproduced with permission from ref. 1454. Copyright Elsevier 2019.

Without the addition of hetero-elements or the inclusion of deposits at their surface, 316L stainless steel (SS) electrodes can be activated for the oxygen evolution reaction (OER).^1277^ This activation can be either *in situ* (surface modification during OER operation, a process that is slow and takes time) or accelerated (*ex situ*: alternating low/high potential steps). Both techniques allow the creation of a catalytic surface from SS bulk components under experimental conditions that are similar to those encountered in real-world applications, ensuring long-term stability and high activity of the surfaces. *ex situ*-Activated electrodes work similarly to *in situ*-activated electrodes, with higher OER activities in KOH electrolytes than other noble-metal-free electrodes. In long-term OER activity (>300 h), activated 316L electrodes are remarkably stable^1454^ (Fig. 112). As a result, activated SS, which is inexpensive and readily available, may be a very competitive OER material for A(EM)WEs, the materials being also compatible with operation as recharge (oxygen) electrode in metal-air batteries.^1454^

**Fig. 113:**
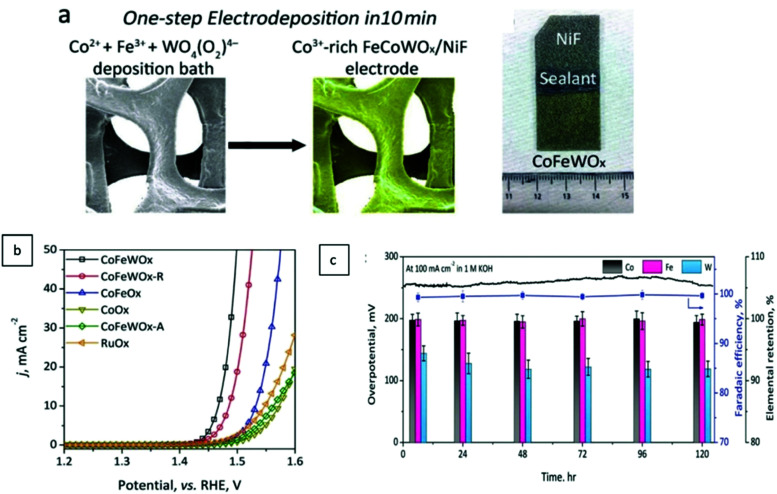
A schematic illustration of the electrodeposition of CoFeWOx on NFs (a), In 1.0 M KOH aqueous electrolyte, catalytic output of catalysts deposited on glassy carbon electrodes (GCEs) for OER in three-electron configuration (b). The electrolyser was tested for stability at 100 mA cm^−2^ in 1.0 M KOH electrolyte. During electrolysis, the elemental preservation of Co, Fe, and W in FeCoWOx/NiF (c). Reproduced with permission from ref. 1456. Copyright Wiley 2020.

Octahedral coordinated trivalent cobalt cations (CoOh^3+^) in metal oxyhydroxides are highly active catalytic sites for the OER; however, previous synthetic methods have limited control over these sites. Octahedral-coordinated trivalent cobalt cations (CoOH^3+^) in metal oxyhydroxides are highly-active catalytic sites for the OER; however, previous synthetic methods have limited control over these sites. A scalable electrodeposition method was developed in conjunction with *in situ* oxidation to generate amorphous Co–Fe–W trimetallic oxyhydroxides enriched in Co^3+^ (Fig. 113a and b). Co^3+^ sites comprise 72% of the Co atoms, according to X-ray absorption and computational studies. The electronic structure of Co is influenced by Fe and W in a synergistic manner, resulting in a favourable coordination environment. With an impressive TOF of 1.96 s^−1^ at *η* = 300 mV, a low Tafel slope of 32 mV dec^−1^, and a small activation energy of 53 kJ mol^−1^ in alkaline electrolyte, the Co–Fe–W oxyhydroxide exhibits high OER activity. In two-electrode water electrolysers, the catalyst directly deposited on Ni foams acts as a robust alkaline OER electrode: *j* = 100 mA cm^−2^ at *η* = 234 mV, 120 h durability at *j* = 100 mA cm^−2^ (Fig. 113b and c), which is ideal for practical water splitting applications.^1456^

**Fig. 114:**
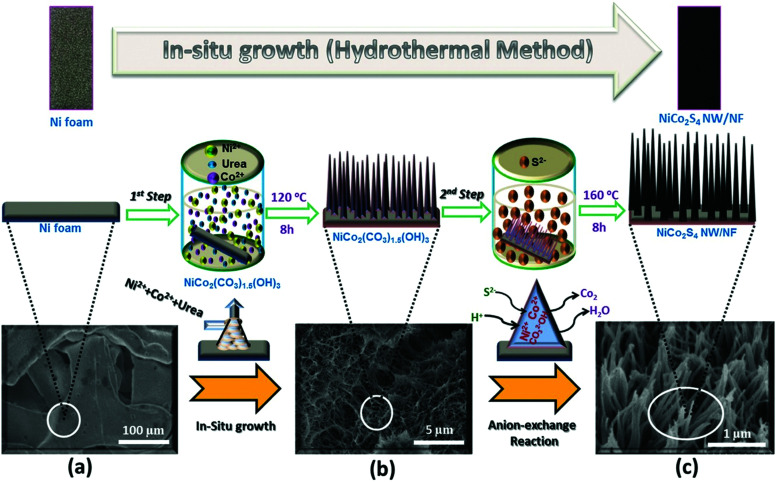
The formation of NiCo_2_S_4_ nanowire arrays on Ni foam and their morphology are depicted schematically. (a) Ni foam substrate, (b) *in situ* growth of NiCo_2_(Co_3_)_1.5_(OH)_3_ nanowire arrays on Ni foam (1st step), (c) hydrothermal anion exchange reaction with full growth of hierarchical NiCo_2_S_4_ nanowire arrays on Ni foam (2nd step) (a). OER polarisation curves (iR-corrected) of NiCo_2_S_4_ NW/NF, Ni_3_S_2_/NF, NiCo_2_O_4_/NF, NiCo_2_S_4_, bare Ni foam, and IrO_2_ with a scan rate of 10 mV s^−1^ (b). HER polarisation curves (iR-corrected) of NiCo_2_S_4_ NW/NF, Ni_3_S_2_/NF, NiCo_2_O_4_/NF, NiCo_2_S_4_, bare Ni foam, and Pt/C (40%) with a scan rate of 10 mV s^−1^. (c). Reproduced with permission from ref. 1455. Copyright Wiley 2016.

*In situ*-Grown 1D NiCo_2_S_4_ nanowire arrays on 3D Ni foams are effective bifunctional electrocatalysts in strongly alkaline electrolytes: binder-free self-made NiCo_2_S_4_ NW/NF electrode delivered *j* = 10 mA cm^−2^ at *η*_OER_ = 260 mV and *η*_HER_ = 210 mV in 1.0 M KOH. These good performances are explained by the material's high surface area, well-separated nanowire structure and uniform length, that was supposed to enhance mass-transport. When used in AWE, the NiCo_2_S_4_ NW/NF catalyst maintained continuous evolution of H_2_ and O_2_ at *j* = 10 mA cm^−2^ and *U*_cell_ = 1.63 V (Fig. 114), showing that overall water splitting is possible with a bifunctional electrocatalyst.^1455^

#### 9.2.2 Preparation of HER catalysts

**Fig. 115:**
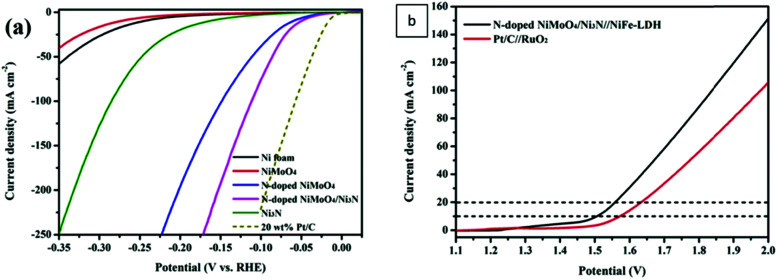
Polarisation curves of Ni foam, NiMoO_4_, N-doped NiMoO_4_, Ni_3_N and N-doped NiMoO_4_/Ni_3_N heterostructure (a). LSV curves of N-doped NiMoO_4_/Ni_3_N//NiFe-LDH and commercial Pt/C//RuO_2_ systems in 1.0 M KOH solution without iR correction (b). Reproduced with permission from ref. 1457. Copyright American Chemical Society 2020.

N-Doped NiMoO_4_/Ni_3_N heterostructure was investigated as a HER electrocatalyst. Its low band gap and high conductivity allows for good carrier transport and transition. The N doping increases the number of active sites on the surface of NiMoO_4_, an advantage for the HER reactivity. Construction of a heterostructure with extended heterogeneous interface enabled to speed up water decomposition, which the authors related to improved hydrogen intermediate adsorption/desorption and increased sites reactivity. Compared to NiMoO_4_, the N-doped NiMoO_4_/Ni_3_N heterostructure achieved efficient HER: *η* = 51 mV at *j* = 10 mA cm^−2^ (Fig. 115a) and a lower Tafel slope value of 45 mV dec^−1^. Coupled with an excellent OER catalyst (NiFe-LDH) in a two-electrode electrolyser, the N-doped NiMoO_4_/Ni_3_N heterostructure needed 1.506 and 1.559 V at *j* = 10 and 20 mA cm^−2^, respectively, with excellent reliability;^1457^ these cell voltage values being lower than for Pt/C//RuO_2_ (Fig. 115b) (1.573 and 1.634 V, respectively), though this not only depends on the intrinsic materials, but also their implementation in efficient GDEs.^1457^

**Fig. 116:**
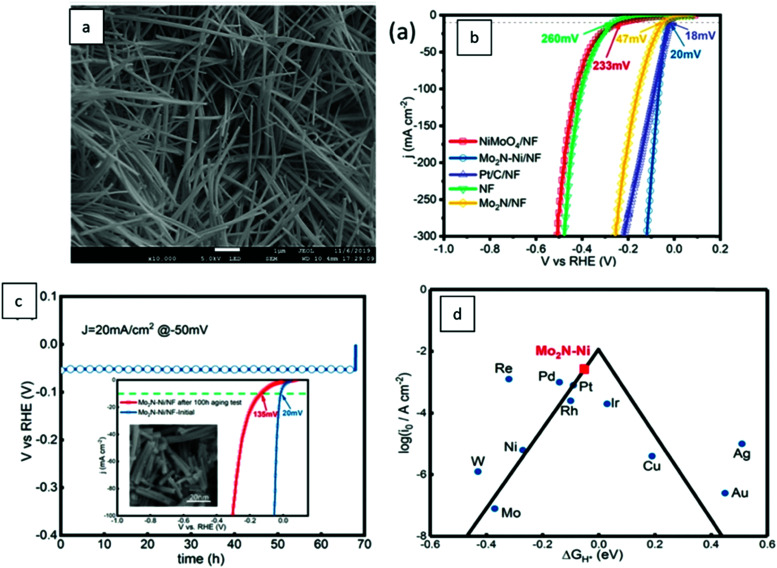
SEM image of Mo_2_N–Ni/NF(a). LSV curves with iR correction in 1 M KOH (b). LSV curves of Mo_2_N–Ni/NF before and after a 100 h aging test, and the SEM image of Mo_2_N–Ni/NF after a 100 h aging test (c). Volcano plot of i_0_ as a function of Δ*G*_H_* for Mo_2_N–Ni and some typical reported electrocatalysts (d). Reproduced with permission from ref. 1458. Copyright American Chemical Society 2020.

Mo_2_N–Ni heterostructure on Ni foam was created by reducing NiMoO_4_ as a precursor during nitridation at different temperatures and for different durations. Such heterostructure between the Mo_2_N phase and metal Ni was shown to improve H-OH dissociation for hydrogen production and thus greatly accelerated the HER (Fig. 116). In alkaline electrolytes, the catalyst showed activity close to that of Pt surfaces, but further research is required to enhance its efficiency in acidic electrodes for large-scale applications. To account for these impressive catalytic performances, the authors put forth the vicinity between Mo_2_N and Ni moieties, that improves the adsorption free energy of H* at active sites, according to DFT calculations (Fig. 116d). This article showcases that simple methods can be used to develop composite electrocatalysts with transition metal and transition metal nitrides-based heterostructures, with large HER activity.^1458^

## 10 Molecular compounds for water electrocatalysis

### 10.1 Molecular compounds for homogeneous- and heterogeneous water oxidation electrocatalysis

**Fig. 117:**
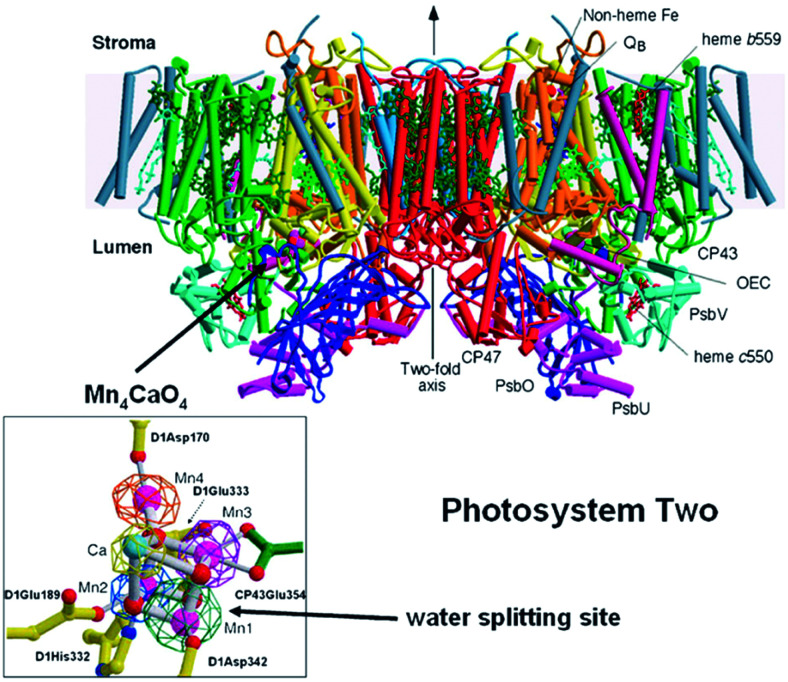
Side view of the structure of Photosystem II, the water splitting enzyme of photosynthesis. This structure was determined by X-ray crystallography. With permission from ref. 1465. Copyright AAAS 2004.

The development of molecular OER catalysts is fundamentally justified, as these are molecular species that enable water to be split *via photosynthesis* in nature and therefore serve as ideal models to develop artificial OER catalysts.^1459–1461^ The most efficient molecular water oxidation catalyst is the naturally-occurring *Water Oxidising Complex* (also known as the Oxygen Evolving Centre) of *Photosystem II* (*PSII-WOC*), which is completed by a collector of light energy. The CaMn_4_O_*x*_ core^1462^ is the active site of Photosystem II^1463,1464^ (Fig. 117). Besides this genius water oxidation core, the efficient removal of electrons (transferred to the complex through the oxidation step) *via* a conductive tyrosine residue coupled to the light absorbing oxidative P680 ^0/+^ complex represents the secret of the smart oxidation process. Being inspired from Nature, the water oxidation complex of PSII has led to a couple of model catalysts for photocatalytic water splitting, referred to as *bio-inspired molecular catalysts* leading to so called artificial photosynthesis.

Molecular systems have the advantage of being easier to study and in addition are considered to be more active per metal center.^1466^ This advantage is however practically attenuated by the imperfect accessibility and low density per unit volume of the active sites, thereby usually resulting into poor surface/volumetric activities *versus* inorganic metal-based catalysts, not to speak from the (often poor) durability of such catalytic moieties. The detailed knowledge of the composition, structure and mechanism of action of the oxygen evolving centre of photosystem II substantially helped to understand the sequential steps of water oxidation occurring through catalytically-active (inorganic) species on macroscopic electrodes and stimulated researchers in developing more efficient potential heterogeneous (solid state) water oxidation electrocatalysts. In addition, it helped improving water splitting photocatalysts, as treated extensively in review articles.^1460–1470^ Photocatalyst materials made up *e.g.*, of molecular assemblies can contain additional catalytic components, often called cocatalysts, that catalyse electrochemical redox reactions (also called electrocatalyst).^1471^

This section deals with molecular OER and HER electrocatalysts (preferably working without sacrificial oxidant) that catalyse water splitting electrochemically, are in the dissolved state or are fixed to macroscopic electrodes (heterogenised) and do not represent a co-catalyst in photocatalyst materials. Water splitting mediated through metal organic framework (MOFs), nanoparticles (not being dissolved in the electrolyte) are at the boundary between molecular and solid-state catalysts and will not be discussed here. Water splitting supported by molecular electrocatalysts can, in principle, be assigned to both heterogeneous catalysis and homogeneous catalysis. When the catalytic active molecular species have been immobilised (heterogenised) by embedding them into a porous macroscopic electrode or by loading them onto a flat metal-oxide or semiconductor oxide-based macroscopic electrode (ITO, FTO), heterogeneous water electrocatalysis is carried out. When the molecular species capable to work as water oxidation electrocatalyst are in the dissolved state in the electrolyte and a macroscopic electrode is used for charge-transfer (namely the regeneration of the reduced form of the molecular species, which was reduced upon oxidising water molecules), homogeneous water electrolysis is performed because catalyst and substrate are in the same phase. The classification of this procedure does not change in case an additional sacrificial oxidant (*e.g.*, Ce(iv) salts) are added. If regeneration of the molecular catalyst is ensured solely chemically by a sacrificial oxidant (see explanation below) homogeneously-catalysed water oxidation, *i.e.*, chemical water oxidation, is carried out. When skimming a paper, it is indeed sometimes not an easy task to decide whether groups have carried out heterogeneous water catalysis or homogeneous water catalysis.^1472,1473,1631^

It is evident from all investigations that whenever molecular catalysts are immobilised (transition from homogeneous catalysis to heterogeneous catalysis), the electrochemical results, *e.g.*, the OER current density to potential relationship, become significantly better.

Design rules have been postulated to develop effective, molecular-based catalysts.^1474^ Ligands of a successful water oxidation complex should be able to withstand strong oxidative potentials; finally, high oxidation states of the central metal need to be accessible at moderate potentials.^1475^

Meyer's blue dimer^1476^ presents the first reported homogeneous (artificial) water oxidising complex (WOC) which functions upon the exploitation of a sacrificial oxidant (Ce(iv)).

**Fig. 118:**
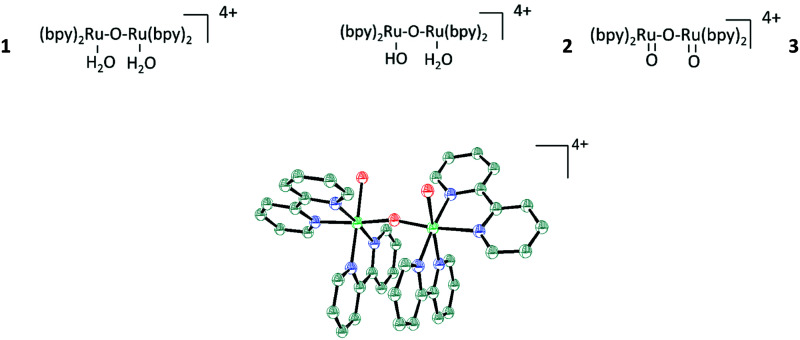
Molecular structure of *cis*,*cis*-[(bpy)_2_Ru(H2O)Ru^III^ORu^III^(OH2)-(bpy)_2_]^4+^ (1).^1477^ Reprinted with permission from ref. 1477 Copyright 1985. American Chemical Society.

Hydrolysis of (bpy)_2_RuCl_2_ (bpy is 2,2′ bipyridine) delivers deep blue solution of (bpy)_2_Ru(H_2_O)Cl^+^ which upon reaction with AgNO_3_ is converted to the oxo-bridged Ru(iii)–Ru(iii) dimeric anion 1 (Fig. 118).

Electrochemically initiated oxidation of 1*via* a glassy carbon electrode may lead to the Ru(iv)–Ru(iii) dimeric anion species 2 through proton-coupled electron transfer (PCET) and upon additional single electron transfer steps may end in the oxo bridged Ru(v)–Ru(v) dimeric anion 3 (*i.e.* a total of a four-electron oxidation process) which is considered to be able to oxidise water into oxygen (OER) as shown in eqn (17). In the absence of dimer, electrolysis upon usage of a glassy carbon electrode did not lead to oxygen evolution.17
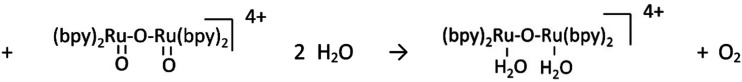
Interestingly intensive oxygen evolution was obtained only in case Ce(iv) which has a standard reduction potential of 1.72 V,^1478^ has been added.

Cerium(iv) turned out to be a powerful one electron oxidant which in the role of the sacrificial oxidant regenerates the catalyst (reconversion of the Ru(iii)–Ru(iii) system to the Ru(v)–Ru(v) system) *via* stepwise transfer of 4 electrons each of which taken by one Ce(iv) ion.^1479^ Thus, it was suggested that Ru(v)–Ru(v) dimeric anion 3 act as the active part of the water oxidation catalyst (WOC), *i.e.*, is capable to oxidise water into oxygen. The blue dimer and species derived from blue dimer are still the subject of current investigations and the elucidation of the catalysis mechanism by the blue dimer is still incomplete yet.^1480–1486^

**Fig. 119:**
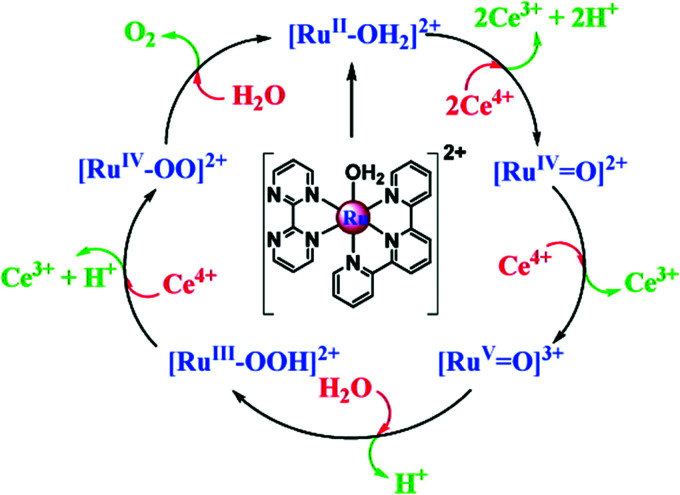
Catalytic cycle for water oxidation by single-site ruthenium-based complexes *via* water nucleophilic attack (WNA), in 0.1 mol L^−1^ de HNO_3_. At pH 0 beyond the steps shown an extra pathway occurs, the [Ru^IV^–OO]^2+^ is further oxidised to [RuV–OO]^3+^, the O_2_ release yields [Ru^III^–OH]^3+^ starting another cycle. Reprinted with permission from ref. 1487. Copyright 2008 American Chemical Society.

The mechanism of water oxidation upon a single site ruthenium polypyridine complex carried out with sacrificial oxidising Ce(iv) was elucidated in 2008^1487,1488^ (Fig. 119).

The catalytic cycle is based on Ce(iv) as sacrificial oxidant (Ox^+^), which are used in many of the subsequently developed systems in most of which they replace an electrode (as mentioned 3 in eqn (17) can be generated through conversion of 1 chemically by adding Ce(iv) instead of using an electrode) or photoelectrode. Thus, they function as a kind of helping agent for the molecular WOC (homogeneous photocatalyst)^1489^ (eqn (18). To ensure reasonable practicability sacrificial oxidants are not wanted and the exploitation of an electrocatalyst (indirect) or photocatalyst (direct) is preferred for solar to fuel conversion.18
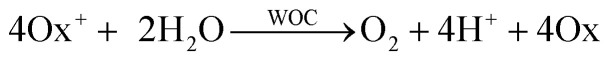


#### 10.1.1 Ruthenium/osmium polypyridine-based molecular OER catalysts

The blue dimer catalyst has limitations and more active single-site polypyridyl Ru aqua complexes have been developed.^1487,1490–1493^

**Fig. 120:**
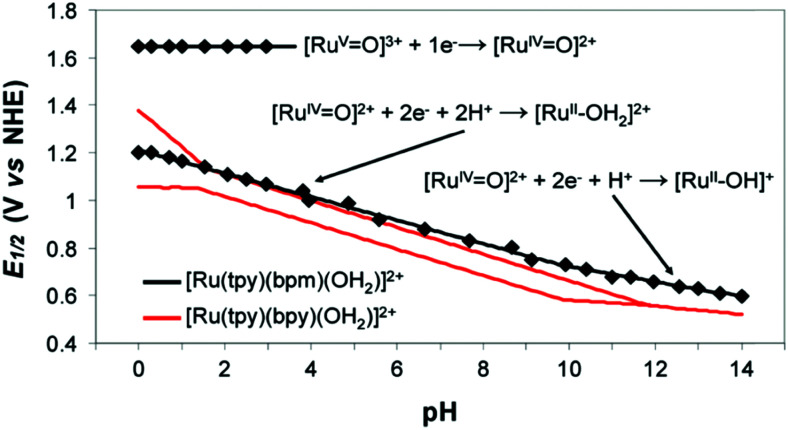
Plots of *E*1/2 (V *vs.* NHE) *vs.* pH for the Ru(v/iv) and Ru(iv/ii) redox couples of [Ru(tpy)(bpm)(OH_2_)]^2+^ and for the Ru(iv/iii) and Ru(iii/ii) redox couples of [Ru(tpy)(bpy)(OH_2_)]^2+^ in aqueous solution (I) 0.1 M; T) 298 K; glassy carbon working electrode). Reprinted with permission from ref. 1479 Copyright 2008. American Chemical Society.

Ru(tpy)(bpm) (OH_2_)^2+^ and Ru(tpy)(bpz) (OH_2_)^2+^ (tpy is 2,2′:6′2′′-terydine, bpm is 2,2′-bipyrimidine and bpz is 2,2′-bipyrazine) have proven to undergo hundreds of turnovers without decomposition.^1487^ The potential -pH diagram of both species is shown in Fig. 120.

A strategy to enhance (blue dimer based) water oxidation catalysed by Ce(iv) system is to add redox mediators ([Ru (bpy)_2_LL]^2+^ (LL = bpy, bpm, bpz), exhibiting substantially faster electron-transfer kinetics when compared to Ce(iv) system.^1494^

**Fig. 121:**
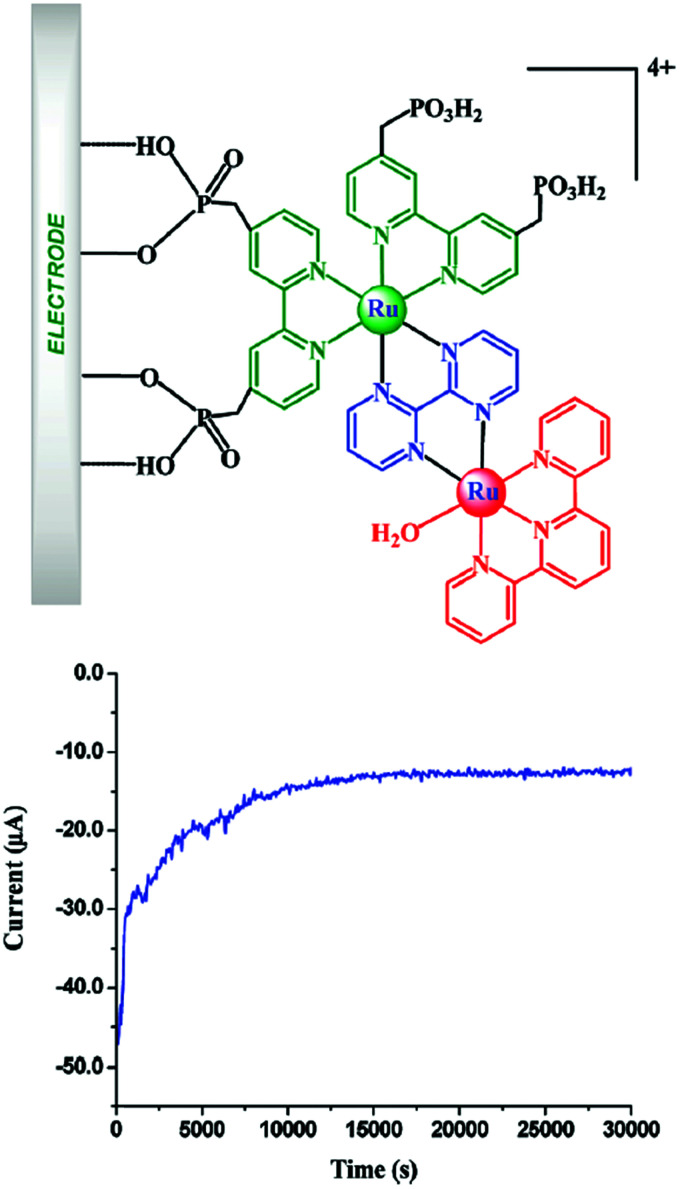
Electrolysis of [(4,4′-((HO)_2_P(O)CH_2_)_2_bpy)2RuII(bpm)–RuII(tpy)(OH_2_)]^4+^ on FTO at 1.8 V in 1.0 M HClO_4_: turnovers > 8900; rate) 0.3 s^−1^; current density ≈ 6.7 μA cm^−2^; *Γ* ≈ 7 × 10^−11^ mol cm^−2^; (A) 1.95 cm^2^. For [(4,4′-((HO)_2_P(O)CH_2_)_2_bpy)_2_Ru^II^(bpm)Ru^II^-(Mebimpy)(OH_2_)]^4+^ on FTO at 1.8 V in 1.0 M HClO_4_: turnovers >28 000; rate) 0.6 s^−1^; current density ≈ 14 μA cm^−2^; *Γ* ≈ 7 × 10^−11^ mol cm^−2^; (A) 1.95 cm^2^. Reprinted with permission from ref. 1495 Copyright Wiley 2009. American Chemical Society.

Combining phosphonate surface-binding and mediator-catalyst assembly (electron-transfer mediator and catalyst function in the same molecule firmly attached to FTO or ITO electrode) ensured sustained electrolysis of 1.0 M HClO_4_ for more than 20 hours (Fig. 121^1495^).

However, the current density was rather low and the overall OER efficiency based on voltage–current behaviour and upon similar materials anchored to TiO_2_, is not close to be competitive *versus* conventional alloy-based OER electrodes (not to speak from their durability).^40,1454,1496^ Some very recently-developed ruthenium polypyridine-based OER electrocatalysts for modification of electrodes were found to be somewhat more active and stable towards OER:^1497^ OER faradaic efficiency values in acid with ruthenium-polypyridine-based materials loaded on FTO or glassy carbon are in between 13^1498^ and 50%.^1499^ Very rarely values around 90% have been obtained^1500,1515^ (Table 12), when faradaic efficiencies of *e.g.*, alloy-based OER electrocatalysts are (determined in acids) often >80%.^833,1501–1503^ The catalyst performance can, in addition, be evaluated in terms of turnover numbers (TONs, defined as moles of produced product per mole of catalyst) and turnover frequencies (TOFs, defined as moles of produced product per mole of catalyst per unit of time). Ruthenium complexes are known to reach TOFs of up to 50 000 s^−1^.^1500^

**Table 12:** Faradaic efficiency of the electrochemically promoted water oxidation reaction using Ru complexes. Abbreviations: tpy = 2,2′:6′,2′′-terpyridine, H2bda = 2,2′-bipyridine-6,6′-dicarboxylic acid, PO(OH)_2_)_2_-bpy = 4,4′-bismethlylenephosphonato-2,2′-bipyridine, bpy = 2,2′-bipyridine, 4-Mebpy-4′-bimpy = 4-(methylbipyridin-4′-yl)-*N*-(benzimidazole)-N′-pyridine), (PO**3**OH**2**)2-bpy) = 2,2′-bipyridine-4,4′-diyldiphosphonic acid. Tda = [2,2′:6′,2′′-terpyridine]-6,6′′-dicarboxylate

Compound	Faradaic efficiency (%)	Ref.
Poly[{Ru(H_2_O)(phen)}_2_(tpy2ph)]	39	1496
[Ru(H_2_O)(tpy)(PO(OH)_2_)2-bpy]]^2+^	27	1499
poly-[Ru(bda)(4-vinylpyridine)_2_]	13	1498
[(bpy)_2_Ru(4-Mebpy-4′-bimpy)Ru(H_2_O) (tpy)]^4+^	28	1504
[Ru(H_2_O)(Mebimpy)(PO_3_OH_2_)_2_-bpy]]^2+^	50	1499
[RuIV(OH)(tda-κ-N_3_O)(pyridine)_2_]	92	1500
[Ru^III^(tPaO-κ-N^2^OPOC)(py)_2_]^2−^	93	1515

Improvement of the activity of polypyridine-based molecular OER catalysts has occurred mainly by chosing redox-active metal matching with compatible ligands (first coordination sphere). Coordination of functional groups (weak interaction) of the ligands to the central ruthenium referred to as second coordination spheres was exploited later on to improve the catalytic activity.^1505–1507^ The oxidation potentials of metal complexes can be reduced by negatively charged ligands.^1500,1508,1509^

**Fig. 122:**
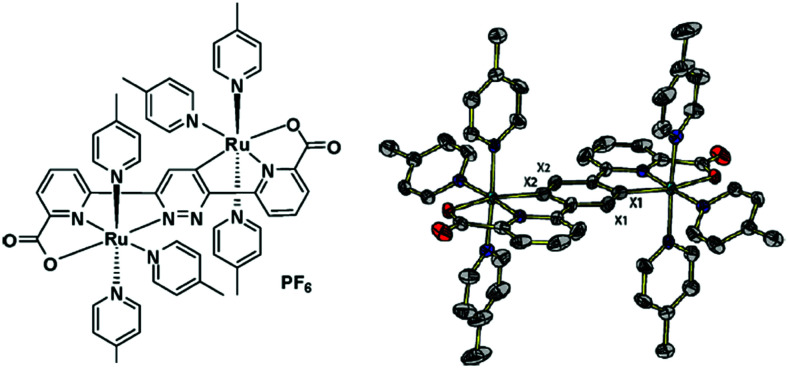
Left side. Structure of a dinuclear ruthenium complex with a negatively charged dicarboxylate ligand. Right side ORTEP view of the cation of the complex with thermal ellipsoids at the 50% probability level. H atoms are omitted for clarity. Reprinted with permission from ref. 1510. Copyright ACS 2009.

A binuclear ruthenium complex bearing a negatively-charged carboxylate ligand function as the WOC was generated and investigated in Sun's group^1510^ (Fig. 122). The same group checked ligands with different σ-donor proprieties, *i.e.*, phosphate- and sulphonate-based bipyridine ligands for Ruthenium coordination as well:^1511^*j* = 0.7 mA cm^−2^ at *η* ≈ 500 mV was reached in bulk electrolysis experiments at in pH 1. Although this consists of poor OER activity when compared to state-of-the-art catalysts, this is an example of true homogeneous water electrocatalysis as the catalyst is dissolved in the electrolyte. Viewed in this light, activity can be considered high. Generally, it seems to be characteristic of many of these studies that great emphasis has been placed on possible reaction mechanisms.^1512^ However, a throughout electrochemical characterisation underpinning the OER activity in detail (inclusive long-term behaviour at reasonable current density) confirming high activity, stability and practicability is very often missing.

**Fig. 123:**
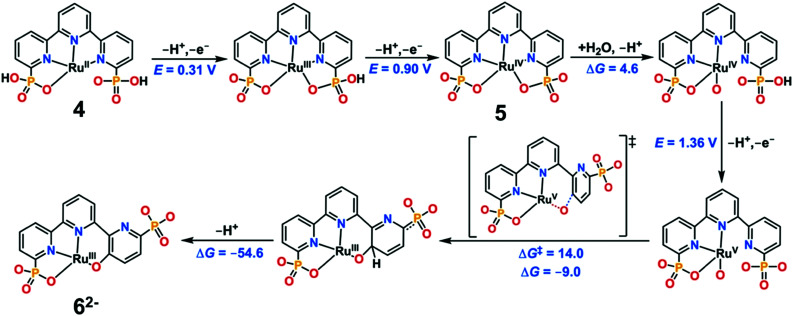
Computed Reaction Pathway at pH 7.0 for the Generation of the Catalytically Active Species [Ru^III^(tPaO-κ-N^2^O_P_O_C_)(py)2]^2−^, 6^2−^, from the Precursor Complex [Ru^II^(H_2_tPa-κ-N^3^O)(py)_2_], 2. Redox potentials (E) in units of volts (V) *vs.* NHE, and Δ*G*s and Δ*G*‡ in units of kcal mol^−1^. Axial pyridyl ligands are omitted for clarity. Reprinted with permission from ref. 1516. Copyright 2020 American Chemical Society.

The development and in-depth evaluation of ruthenium polypyridine complexes^1513,1514^ above all with tda based σ-donors has continued^1515–1517^(Fig. 123 and Table 13). However, the activity *e.g.*, of complex 6 at pH 7 (*j* = 0.8 mA cm^−2^ at *η* = 600 mV) is still very weak when compared to metal (alloy)-based systems.^40^

**Table 13:** The electrochemical performance of molecular water oxidation catalysts mentioned in Section 10.1. Abbreviations: tda^2−^: [2,2′:6′,2′′-terpyridine]-6,6′′-dicarboxylate

Compound	*η* [μV]/*j* [mA cm^−2^]	pH	Type	Ref.
[Ru^IV^(OH)(tda-K-N^3^O)(py2)]^+^	680/0.3	7	Homogeneous	1500
[Ru^III^(tPaO-κ-N^2^O_P_O_C_)(py)2]^2−^	600/0.8	7	Homogeneous	1515
[Ru(tda)(4,4′-bipy]_*n*_ (4,4′-bpy)	630/30	7	Heterogeneous	1518
[Ru(NH_3_)_5_Cl]^2+^ in Nafion	700/0.12	5.4	Heterogeneous	1530
[Ru(NH_3_)_5_Cl]^2+^ in Pt black	700/3.8	6.8	Heterogeneous	1531
[(NH_3_)_5_Ru(μ-O)Ru(NH_3_)_4_(μ-O)Ru(NH_3_)_5_]^6+^	670/8	6.8	Heterogeneous	1535
Ir-N-Heterocyclic carbene (Ir-NHC) on graphene	250/2.5	7	Heterogeneous	1551
Ir-N-Heterocyclic carbene (Ir-NHC) on carbon nanotubes	800/60	7	Heterogeneous	1551
Fe(tpfc)Cl) on FTO	630/0.75	10	Heterogeneous	1577
Cabalt-*b*-octafluoro-hangman corrole	700/0.10	7	Homogeneous	1586
[NiL](ClO_4_)_2_; *L* = 5,5,7,12,12,14 hexamethyl-1,4,8,11 tetraazacyclotetradecane	730/0.9	7	Homogeneous	1601
NiL; L = 2,2′-((1*E*,1′*E*)-((4-chloro-5-methyl-1,2-phenylene) bis(azanylylidene)) bis(methanylylidene))diphenolate	305/5.5	11	Heterogeneous	1616
(bpy)Cu(OH)_2_; bpy = bipyridine	860/6	13	Homogeneous	1620
CuSO_4_; boron-doped diamond as WE	1000/30	11	Homogeneous	1623
[(TGG^4−^)Cu^II^–OH_2_]^2−^; TGG = triglycylglycine	700/0.5	11	Homogeneous	1524

Upon anchoring to multiwalled CNTs, the electrocatalytic properties of tda complexes were substantially improved (*η* = 630 mV; *j* = 30 mA cm^−2^, pH 7).^1518^ However, graphene,^1519^ graphene oxide,^1520^ carbon nanotubes^1521,1522^ or graphene/carbon nanotubes^1523^ loaded with non- noble transition metal-oxides or with ruthenium directly are, to the best of authors knowledge, not as costly and known to be very active water splitting catalysts as well.

#### 10.1.2 Other ruthenium- or osmium-containing non-solid-state catalysts

The solution chemistry and electrochemical behaviour of ruthenium ammine complexes have been intensively studied.^1525–1528^ Mononuclear ruthenium ammine complexes are known to catalyse water oxidation in the presence of Ce(iv).^1529^ In addition, water oxidation electrocatalysis was performed with [Ru(NH_3_)_5_Cl]^2+^ complex incorporated in Nafion without Ce(iv) support (*η* = 700 mV; *j* = 0.12 mA cm^−2^; pH 5.4).^1530^ Substantially better voltage–current behaviour (*η* = 700 mV; *j* = 3.8 mA cm^−2^; pH 6.8) was found when [Ru(NH_3_)_5_Cl]^2+^ was incorporated in Pt black.^1531^ More detailed catalytic performance measurements have been carried out with trinuclear [(NH_3_)_5_Ru(μ-O)Ru(NH_3_)_4_(μ-O)Ru(NH_3_)_5_]^6+^ incorporated in Nafion:^1532–1534^ reasonable OER efficiencies were only achieved if the OER electrocatalyst was incorporated in Pt black (*j* = 8 mA cm^−2^ and *η* = 670 mv; pH 6.8).^1535^ The intrinsic catalytic activity of ruthenium ammine complexes turned out to be as follows [(NH_3_)_5_Ru(μ-O)Ru(NH_3_)_4_(μ-O)Ru(NH_3_)_5_]^6+^ > [(NH_3_)_5_Ru–O–Ru(NH_3_)_5_]^4+^ > [Ru(NH_3_)_5_Cl]^2+^:^1536^ the multinuclear complexes are more active because they are capable for a one-step-4-electron transfer, while the mononuclear complex needs two molecule for O_2_ evolution.^1536^

The vast majority of the papers dealing with water splitting mediated through Ru-containing molecular systems are based on Ru-pyridine/polypyridine or Ru-ammine complexes. However, some other non-solid-state-based Ru-containing catalysts have been designed and used for water electrocatalysis.^1537–1543^

However, no breakthrough in terms of an acceptably high catalytic activity has yet been achieved with ruthenium-containing non solid state electrocatalysts for truly homogeneous electrocatalysis.

Os polypyridyl complexes such as Os(tpy)(bpy)(OH_2_)^2+^ are promising as the redox potentials for Os(iii/ii) couples and couples with higher oxidation states were found to be lower by 0.3–0.4 V relative to their Ru analogs enabling access to *e.g.* M^V^=O^3+^ at relatively low potentials.^1544,1545^

#### 10.1.3 Iridium-based molecular water oxidation catalysts

**Fig. 124:**
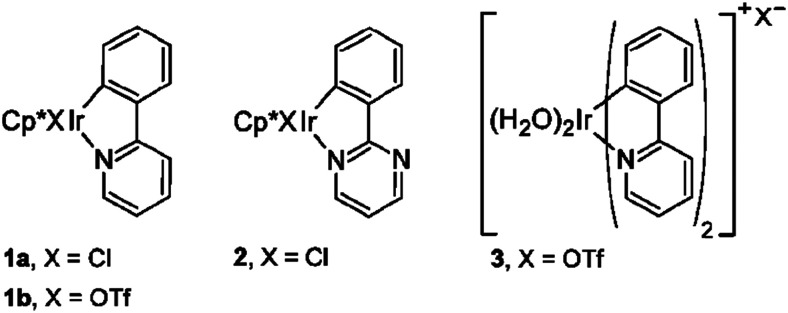
Iridium catalysts for water oxidation. Reprinted with permission from ref. 1547 Copyright 2009. American Chemical Society.

Pyridine-iridium complexes like cyclometalated bis-phenylpyridine diaquo iridium(iii) complexes have been introduced by Bernard *et al*.^1546^ The materials have been throughout electrochemically- investigated for homogeneous water electrocatalysis using CAN when evaluating the OER properties: the OER activity, *i.e.*, the (OER-based) current density to potential ratio is rather weak. Three different cyclopentadienyl iridium complexes (Fig. 124) were synthesised and characterised by Brudvig and Crabtree.^1547^

In view of the considerable material costs (Ir metal, expenses for the organic ligands) the overall electrochemical activity with current densities *j* < 1 mA cm^−2^ over a wide potential range is very poor. Iridium complexes with differently stabilised triazole-derived carbene ligands for water oxidation catalysis have been evaluated upon using different sacrificial oxidants by Mazloomi *et al.*^1548^ Several other works based on homogeneous water electrocatalysis mediated through molecular iridium containing species have appeared.^1549,1550^ Generally, rather time-consuming approaches and considerable material costs lead to a rather low, achieved catalytic activity.^1549,1550^

As expected, much higher OER efficiency can be achieved if heterogeneous electrocatalysis is sought. As for instance Iridium, N-heterocyclic carbenes (NHC) immobilised *via* graphene, exhibited substantially better current voltage behaviour: *j* = 2.5 mA cm^−2^ at *η* = 250 mV^1551^ in neutral medium.

**Fig. 125:**
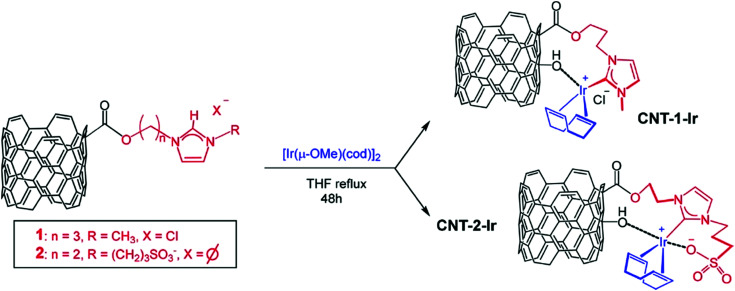
General Procedure for the Synthesis of Hybrid Carbon Nanotubes-Based Ir^I^-NHC Catalysts. Reprinted with permission from ref. 1552. Copyright 2019. American Chemical Society.

Carbon nanotube supported Ir-NHC complexes as water oxidation catalysts have been shown recently by Nieto *et al.*^1552^ (Fig. 125) up to *j* = 60 mA cm^−2^ at *η* ≈ 800 mV was reached with the best catalyst (CNT-2-Ir, investigated pH 7 (steady-state measurements).

The authors believe that the publishing activity on iridium- or ruthenium-containing molecular species for electrocatalysis purposes has recently slowed, most likely due to the scarcity of the element and the recently significantly improved catalytic activity obtained with Fe, Co, or Ni-based molecules.

#### 10.1.4 Earth-abundant molecular catalysts for OER

First raw transition metal containing complexes have been specifically investigated as potential water splitting catalysts.^1553^

##### 10.1.4.1 Manganese containing complexes

Inspired in large part by the structure of the oxygen evolving complex in Photosystem II, inorganic clusters, *e.g.*, tetramanganese ones have been considered for water oxidation mediated by molecular systems. Due to the absence of organic ligands, they are intrinsically more stable than the ones discussed so far.

Brudvig and Crabtree reported on a dinuclear manganese complex [(OH_2_)Mn(tpy)(O)_2_Mn(tpy)(OH_2_)] with considerable activity for OER upon using OCl^−^ or HSO_5_^−^.^1554^ However, OER can only partly be assigned to water oxidation and, in addition thermodynamically- favoured formation of MnO_4_^−^ which is known to be inactive to support OER, is a serious obstacle.^1468,1555^

A manganese complex capable of oxidising water to oxygen in homogeneous solution when using a single-electron oxidant ([Ru(bpy)_3_]^3+^) in neutral phosphate buffer was introduced by Karlsson *et al.*^1556^

Tetramanganese (Mn_4_ fragment containing) clusters have preferably been exploited to drive OER under light illumination,^1557^ under both light illumination plus an applied potential (photoelectrocatalytic water splitting)^1558^ or were even found to be unable to catalyse water oxidation.^1559,1560^

##### 10.1.4.2 Iron containing complexes

Iron-containing complexes have been designed and investigated as potential oxygen evolution centers as well.^1561–1570^ However, very often, these complexes decompose under the strongly oxidising test conditions and the formed iron ions create iron-oxide, which is the real active species promoting water oxidation with release of oxygen.^1571,1572^

In 2010, a series of Fe^III^ complexes containing tetraamido macrocyclic ligands (Fe^III^-TAMLs) were reported as the first example of molecular iron WOCs by Bernhard and Collins.^1573^

**Fig. 126:**
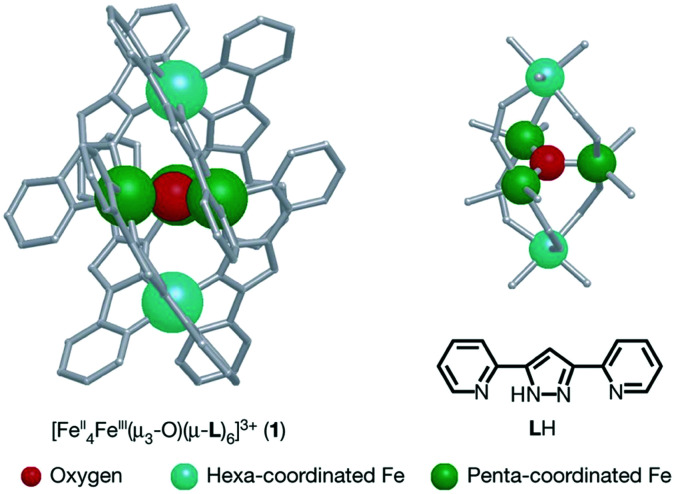
Ball-and-stick representations of the molecular structure (left) and the Fe_5_O core structure (right) of [Fe^II^_4_Fe^III^(μ_3_-O)(μ-**L**)_6_]^3+^. Three penta-coordinated iron centres are bridged by an oxygen atom in μ_3_-fashion to form a triangle structure, and two hexa-coordinated iron centres are connected to the triangle structure by six **L**s. Reprinted with permission from ref. 1574. Copyright 2016. Nature Publishing.

A pentanuclear iron complex ([Fe^II^_4_Fe^III^(μ_3_-O)(μ-L)_6_]^3+^; LH = 3,5-bis(2-pyridyl)pyrazole; Fig. 126) was designed by Okamura *et al.*^1574^ and, dissolved in an acetonitrile/water mixture, checked for its water oxidation capabilities (homogeneous water electroctatalysis).

However, only μA cm^−2^ were reached at a certain potential, far from solid-state-based electrodes. This group later developed a pentanuclear iron electrocatalyst with electron donating and withdrawing new ligands.^1575^ However, in practice reasonable current densities were not achieved.

Karim *et al.* investigated the electrocatalytic activity of a newly synthesised dinuclear oxo-bridged iron complex [(FeLCl)_2_O](FeCl_4_)_2_] (L = (2-(pyrridin-2-yl)oxazolidi-ne-4,4-diyl.^1576^

During bulk electrolysis in organic solvent/aqueous NaOH mixtures the catalyst showed a TON of 408 in 1 h and TOF of 0.11s^−1^.

**Fig. 127:**
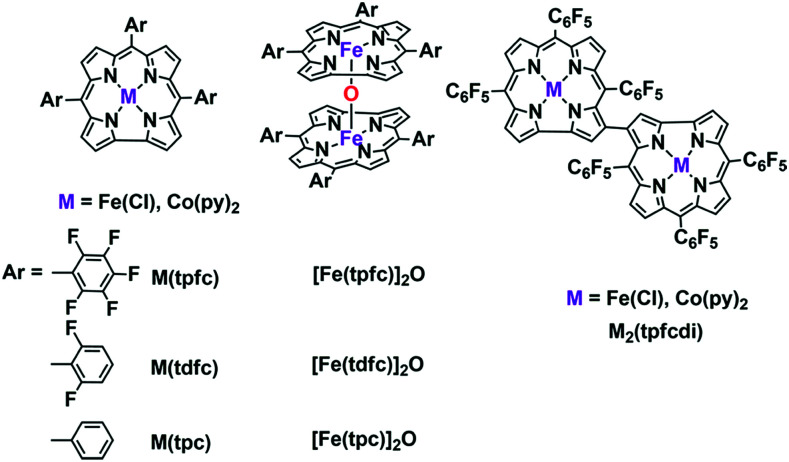
Iron and Cobalt Metallocorroles tested and compared as WOCs in the study presented by Sinha *et al.*^1577^ Reprinted with permission from ref. 1577 Copyright 2020. American Chemical Society.

A current study addressed the water-oxidising ability of mononuclear and two types of binuclear iron corroles: μ-oxo bridged and linked through β-pyrrole C atoms (Fig. 127).^1577^

**Fig. 128:**
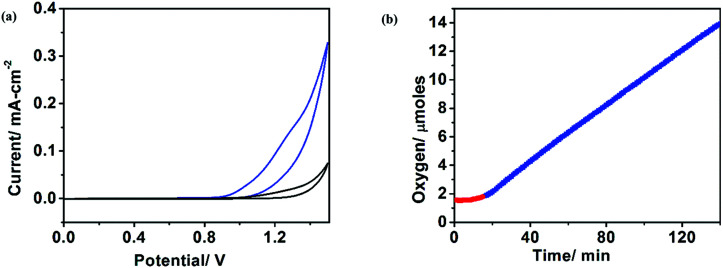
Cyclic voltammograms (V *vs.* Ag/AgCl) of Nafion films, loaded (blue trace) and not loaded (black trace) with Fe(tpfc)Cl, on FTO electrodes in pH 10 phosphate–KOH buffer (scan rate of 100 mV s^−1^; catalyst loading 1.6 nmol cm^−2^). (b) Evolution of oxygen before (red) and after (blue) application of a potential of 1.5 V. Reprinted with permission from ref. 1577 Copyright 2020.American Chemical Society.

The electrocatalysts were heterogenised (loaded on Nafion films on FTO) and electrochemically fully characterised in pH 10 buffer solution (Fig. 128). Generally, the bimetallic species were not as efficient as their monometallic counterparts. The electrode-adsorbed iron corrole Fe(tpfc)Cl exhibited a faradaic efficiency of >95%; *j* ≈ 0.75 mA cm^−2^ at *η* ≈ 630 mV.

##### 10.1.4.3 Cobalt containing complexes

**Fig. 129:**
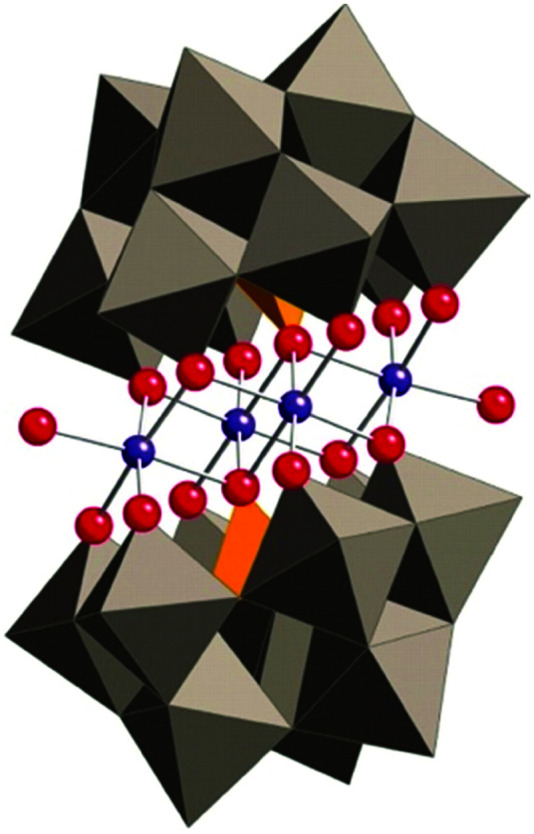
X-Ray structure of Na_10_[Co_4_(H_2_O)_2_(PW_9_O_34_)_2_] in combined polyhedral ([PW_9_O_34_] ligands) and ball-and-stick (Co_4_O_16_ core) notation. Co atoms are purple; O/OH_2_(terminal), red; PO_4_, orange tetrahedra; and WO_6_, gray octahedra. Hydrogen atoms, water molecules, and sodium cations are omitted for clarity. Reprinted with permission from ref. 1583 Copyright 2010. AAAS.

Cobalt salts or simple cobalt complexes have been investigated as potential water oxidation catalysts.^1578–1582^ Water oxidation was observed on [Co_4_(H_2_O)_2_(PW_9_O_34_)_2_]^10−^ upon adding a stable stoichiometric (sacrificial) oxidant (Fig. 129).^1583^

**Fig. 130:**
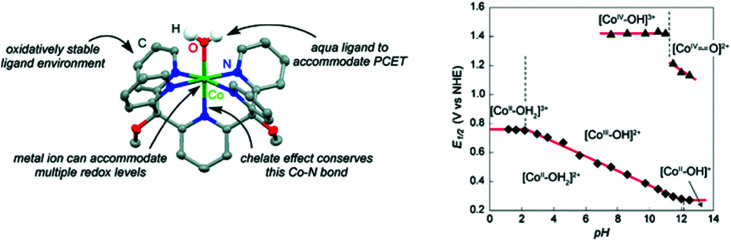
A structural representation of [Co^II^(Py5)(OH_2_)](ClO_4_)_2_] (left image). Pourbaix diagram for [Co^II^(Py5)(OH_2_)](ClO_4_)_2_]. Reprinted with permission from ref. 1585 Copyright 2011 RSC.

A complex with a tetravalent Co centre stabilised by PCET was unknown until 2011.^1584^ Wasylenko *et al.* exploited the oxidatively stable pentadentate ligand environment of 2,6-(bis(bis-2-pyridyl)methoxy-methane)-pyridine (Py5) to form the stable coordination compound, [Co(Py5)(OH_2_)](ClO_4_)_2_ (Fig. 130).^1585^ This compound when converted to a Co(iv) species is capable to function as a (homogeneous) water oxidation catalyst in the presence of a base (Fig. 130).^1585^

Kinetic and electrochemical studies suggests that the complex acts as a molecular catalyst. The OER current density reached at certain overpotential was very low (<1 mA cm^−2^), limiting the practical interest of such compound.

**Fig. 131:**
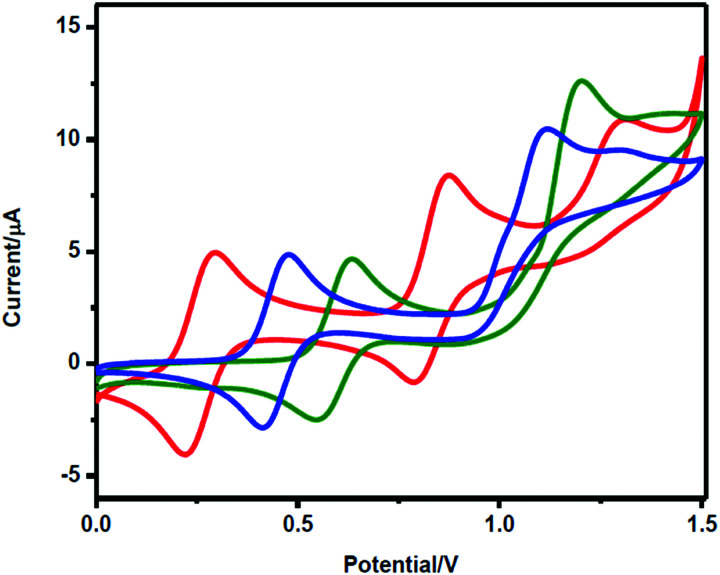
Water oxidation in the presence of Co(tpc)Py_2_ (red), Co(tdfc)Py_2_ (blue) and Co(tpfc)Py_2_ (green) in acetonitrile on adding 4.8% water. Reprinted with permission from ref. 1577 Copyright 2020. American Chemical Society.

Cobalt corroles are known to be capable to support water oxidation catalysis^1577,1586–1588^ (Fig. 131). Faradaic efficiency determinations confirmed in many cases quantitative charge to oxygen conversion. However, the electrochemical OER current density achieved upon applying a certain overpotential was rather weak.

**Fig. 132:**
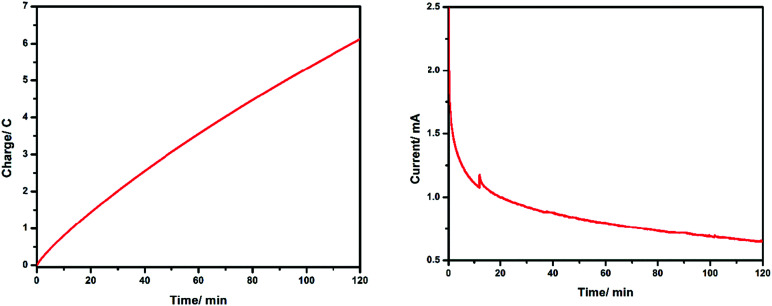
The charge *vs.* time and current *vs.* time plots obtained from chronoamperometric measurements at an applied potential of 1.5 V for two hours to the Co(tpfc)Py_2_ -loaded Nafion film. Reprinted with permission from ref. 1577 Copyright 2020 American Chemical Society.

Among the cobalt complexes, Co(tpfc)Py_2_ exhibited the best OER efficiency (FE ≈ 95%) and current-potential ratio (Fig. 132). However, parallel investigations on the corresponding iron analogues uncovered that the iron corroles are better OER catalysts not only in terms of efficiency but also in stability.

Molecular species containing cobalt, which can support water oxidation, have not lost any of their attractiveness as a research topic.^1589–1599^ Just the contrary- when inserting *e.g.*, “cobalt-water-oxidation” into Thomson Reuters ISI Web of knowledge (Advanced search; field tag: TI = cobalt water oxidation) around 20% of the results can be assigned to cobalt containing molecules as either heterogeneous or homogeneous water oxidation catalysts.

##### 10.1.4.4 Nickel containing complexes

Whereas solid-state Ni-based catalysts have been studied intensively in science and technology and are state of the art in many industrial systems, molecular Ni complexes did not receive that much attention for water oxidation, at least until recently.^1553^

**Fig. 133:**
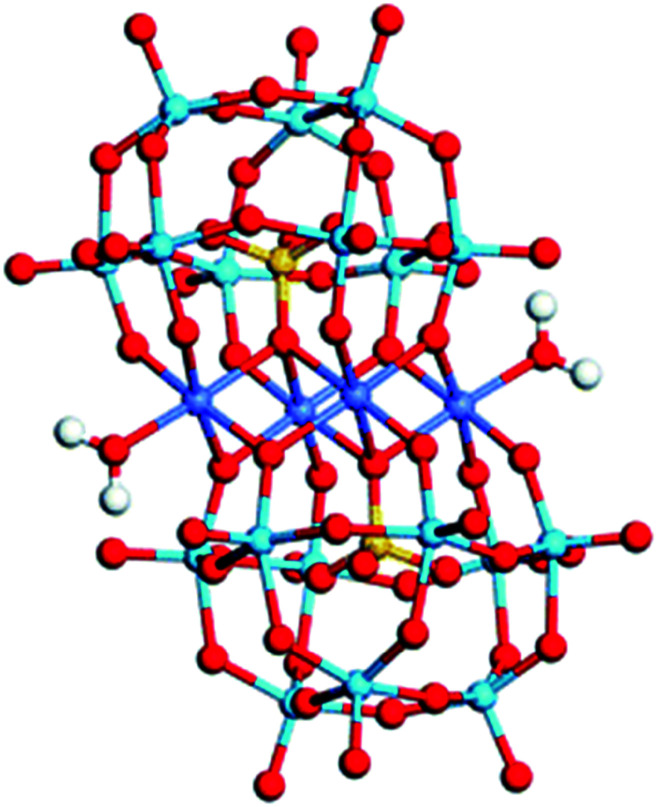
Structural model of [Ni_4_(H_2_O)_2_(SiW_9_O_34_)_2_]^12−^ (M = Ni; W: blue, O: red; S: yellow; H: white; M: dark blue). Reprinted with permission from ref. 1600. Copyright 2012 RSC.

The first Ni-based non-solid-state water oxidation catalyst, sandwich-type tetra nickel polyoxometalate K_11_Na_1_[Ni_4_(H_2_O)_2_(SiW_9_O_34_)_2_]·*n*H_2_O which is based on a Keggin-type building block (Fig. 133) shows the anion) was reported by Car *et al.*^1600^

**Fig. 134:**
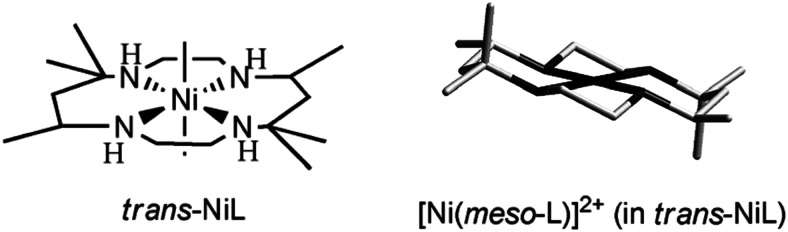
The structure of [Ni(meso-L)]^2+^. Reprinted with permission from ref. 1601. Copyright 2014 Wiley.

Zhang *et al.* reported in 2014 about a Ni-containing complex with a cyclam-like meso ligand [Ni(*meso*-L](ClO_4_)_2_ with L = 5,5,7,12,12,14 hexamethyl-1,4,8,11 tetraazacyclotetradecane (Fig. 134)^1601^ suitable for water oxidation electrocatalysis (*η* = 730 mV; *j* = 0.9 mA cm^−2^).

The faradaic efficiency amounted to 97.5%. When the bulkiness of the ligands (number of methyl groups of the macrocyclic ligand) is varied, it majorly influences catalyst activity.^1602–1604^

Many other groups reported about nickel complexes capable to work as water oxidation catalysts upon using porphyrin-, cyclam, oxamidate-, and pyridine-based ligand frameworks.^1605–1612^ However, one needs to distinguish between the OER activity that originates from nickel oxide particles or nickel salts that have been formed from the nickel complex (follow-up reaction) under water electrolysis condition and the true OER activity of the corresponding nickel complex.^1613^

Up to now, the investigations of nickel complexes as potential electrocatalysts for water oxidation are extremely popular and many other examples were introduced throughout the last years.^1614–1619^ It is not a surprise that better catalytic activity (up to *j =* 5.5 mA cm^−2^; *η* = 305 mV; pH 11)^1616^ was revealed when the molecular species is immobilised on a macroscopic electrode, thus heterogenic catalysis has been performed,^1614–1616^ although in some case respectable efficient homogeneous electrocatalysis was shown.^1619^

##### 10.1.4.5 Copper containing complexes

Elizarova *et al.* were the first to evaluate the OER properties of copper salts and copper containing complexes^1579^ (CuCl_2_, [Cu(bpy)_2_Cl_2_], [Cu(bpy)_3_Cl_2_]) in homogenous water catalysis at pH 10. Faradaic efficiencies in between 32% and 43% were determined, but detailed electrochemical data were not provided.

**Fig. 135:**
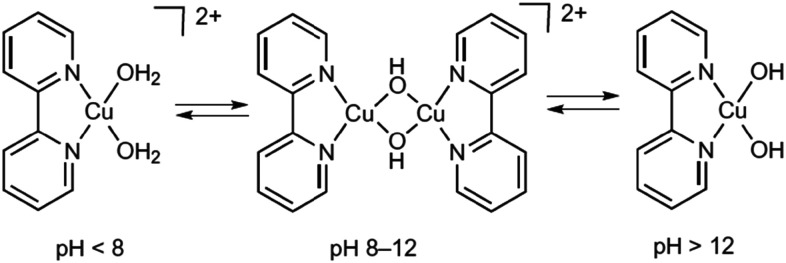
The aqueous speciation of a 1 : 1 copper(ii):bpy solution, observed by EPR. Reprinted with permission from ref. 1620. Copyright 2012 Nature Publishing.

More than 30 years later three Copper Bipyridinium complexes [(bpy)Cu(μ-OH)]_2_X_2_ (X = CH_3_COO^−^, CF_3_SO_3_^−^, and SO_4_^2−^) were checked for their water splitting capabilities by Barnett *et al.*^1620^ (Fig. 135).

A more detailed investigation unmasked (bpy)Cu(OH)_2_ as the major species present under electrocatalytic conditions at pH 13, which is consistent with earlier findings.^1621,1622^ The catalyst exhibited a rather moderate activity (*η* = 860 mV at *j* = 6 mA cm^−2^) derived from amperometry measurements.

Meyer *et al.* reported on simple Cu(ii) salts as potential water oxidation catalysts.^1623^ CuSO_4_ dissolved in 1 M Na_2_CO_3_ is capable to reasonably promote the OER: *j* = 30 mA cm^−2^ at *η* = 1000 mV when boron-doped diamond is the working electrode, a decent activity for real homogenous electrocatalysis, even if the catalyst is not even close to solid-state based electrocatalysts at pH 11. A complete review of Cu-containing molecular complexes is not wanted here, but it is wise to say that substantial less research activity can be assigned to copper-based molecular OER catalysts in direct comparison^1624^ with their cobalt-, iron- or nickel analogues.^1625–1629^ Recently there have been reports about activities that are getting somewhat better.^1629^ However, even in the more recent publications, the activity indicated for heterogeneous catalysis remains significantly higher than that inferred (with identical species) from homogeneous catalysis.^1629^

### 10.2 Molecular compounds for homogeneous- and heterogeneous water reduction electrocatalysis

In this section, we will focus on molecular compounds based on metals abundant in the Earth's crust that supports hydrogen evolution *via* heterogeneous or homogeneous electrocatalysis preferably in aqueous systems. The molecular species catalysing HER may either be attached to an electrode surface to realise heterogeneous catalysis or freely diffusing in the electrolyte (homogeneous catalysis). In the latter case, the electrode solely provides electrons to the molecular catalyst. Many reviews dedicated to HER electrocatalysts have been published^16,62,1630–1637^ and we will concentrate on the most recent results, limiting ourselves to examples reporting efficient electrocatalysis upon molecular catalysts (Table 14).

**Table 14:** The electrochemical performance of molecular water reduction catalysts mentioned in Section 10.2

Compound	*η* [mV]/*j* [mA cm^−2^]	pH	Type	Ref.
Nickel diphosphine complex on multiwalled carbon nanotubes (MWCNTs)	300/4.5	0	Heterogeneous	1677
[(PY_5_Me_2_)MoO(PF_6_)_2_)]; PY5Me_2_ = 2,6-bis(1,1-bis(2-pyridyl)ethyl)pyridine	600/2	7	Homogeneous	1679
Cu polyoxometalate complex embedded into carbon cloth	95.4/10	13	Heterogeneous	1687
[Co_3_(C_24_S_12_)]_*n*_ (Co-PTC complex; PTC = perthiolated coronene) on carbon film	227/10	0	Heterogeneous	1688
[Co(Py3Me-Bpy)OH_2_] (PF_6_)_2_	1000/10	7	Homogeneous	1689
Bpy = *N*,*N*-bis(2-pyridinylmethyl)-2,2′-bipyridine-6-methanamine				
Long chain Zr-porhyrine complex	60/10	0	Heterogeneous	1700
Ru-tannic acid complex (Ru-TA) on activated carbon cloth	29/10	14	Heterogeneous	1701
*N*,*N*,*N*′,*N*′-Tetramethyl-*p*-phenylenediamine intercalated between 1T′ phase MoS_2_ nanosheets	150/10	0	Heterogeneous	1708
Ni-Quinazoline-2(1*H*)-thione on glassy carbon	250/1.4	0	Heterogeneous	1647
Co(ii)bis(diselenoimidodiphosphinato)	630/10	14	Heterogeneous	1648
[Co{(SePiPr2)2N}2] on Au				
Co(bpbH_2_)Cl_2_] (bpbH2: *N*,*N*′-bis(20-pyridinecarboxamide)-1,2-benzene)	1350/1.5	7	Homogeneous	1649
	1260/1.4	8.6		

A detailed discussion of possible mechanistic ways to reduce protons, in the area of which either experimentally^1638–1640,1662,1665,1666^ or theoretically^1641–1644^ significant research activities have been carried out, is dispensed at this point.

Nature provides exquisite examples of catalysts in the form of hydrogenase enzymes which are based on cheaper, abundant metals like iron and nickel for proton to hydrogen catalysis and achieve considerable efficiency.^1645,1646^

**Fig. 136:**
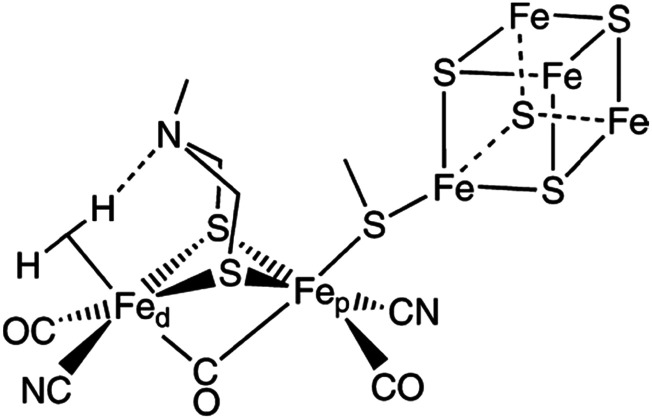
A proposed structure of the active site of the [FeFe] hydrogenase enzyme. Reprinted with permission from ref. 1646 Copyright 2007. American Chemical Society.

Dinuclear iron^1646^ or nickel-iron complexes^1646,1650–1652^ represent the actives sites of the enzymes (Fig. 136). Both classes of hydrogenases can catalyse both proton reduction or hydrogen oxidation, but it is common claims that [Fe] only hydrogenases have a greater activity for the HER, while [NiFe] hydrogenases are more efficient for the conversion of hydrogen to protons (HOR). Thus, inspired by nature functional Fe-Fe hydrogenase were deliberately imitated.^1653–1657^ Although cobalt has no biological relevance and is significantly less abundant in the Earth crust (∼30 ppm) than Fe (6.3%) or Ni (90 ppm) it is a promising metal centre for molecular and solid state electrocatalysts. Starting more than 40 years ago proton reduction was reported for a series of Ni^II^ and Co ^II^ tetraazamacrocycles.^1658–1660^ Fisher and Eisenberg reported on such a cobalt-based species that catalyses hydrogen production from pure water with up to 80% faradaic yield at potentials as low as −1,36 V *vs.* RHE on a mercury pool electrode.^1658^ Cyclopentadienyl cobalt complexes were also among the earliest proton reduction catalysts examined in aqueous solutions: Grätzel *et al.* reported on [Co(Cp-COOH)_2_]^+^ to serve as water reduction electrocatalysts at −0.66 V *vs.* RHE in pH 6.5 phosphate buffer solution.^1661^ Cobalt complexes with glyoxime-based macrocycles have proven their ability to chemically-^1662^ or (decades later) electrocatalytically^1663–1670^ reduce protons in terms of homogeneous catalysis above all in non- aqueous solvents. Cobalt cage complexes were checked for suitability to act as HER electrocatalysts as well by groups of Grätzel and Sargeson.^1661,1671^ As expected, catalysis experiments resulted either in quite modest current to potential ratios in case homogeneous catalysis was performed^1672^ or, in case the catalytic active species have been immobilised substantial higher current density was reached at certain overpotential values.^1669,1673^

**Fig. 137:**
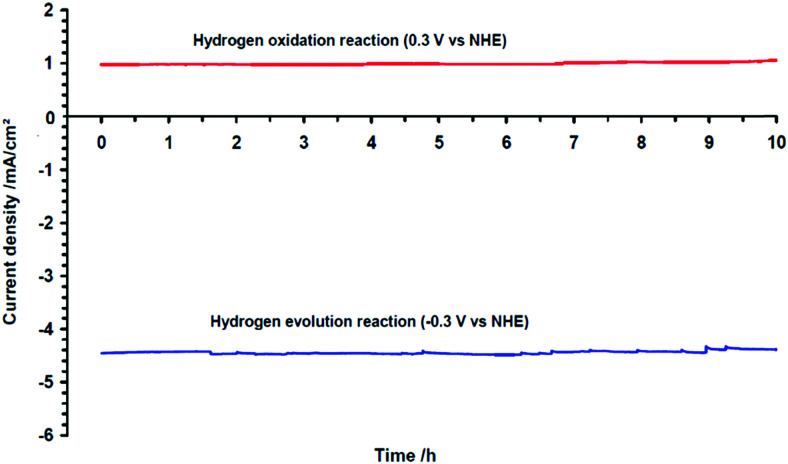
Long-run electrolysis experiments for both hydrogen evolution and oxidation carried out respectively at −0.3 and +0.3 V *vs.* NHE in H_2_SO_4_ (0.5 mol L^−1^) on a membrane electrode on which MWCNTs have been deposited and further Ni-functionalised. Reprinted with permission from ref. 1677 Copyright 2009 AAAS.

Ni bis(phosphine) complexes known to facilitate H_2_ oxidation^1636^ have also been deeply investigated for water reduction purposes, above all in the group of Dubois.^1636,1674–1676^ Nickel diphosphine complexes were later on covalently attached onto multiwalled carbon nanotubes (MWCNTs) and used for heterogeneous HER electrocatalysis in 0.5 M H_2_SO_4_: they exhibited onset of hydrogen evolution at *η* < 50 mV and *j* = 4.5 mA cm^−2^ at *η* = 300 mV, derived from long-term bulk electrolysis (Fig. 137).^1677^ This represents an outstanding HER efficiency compared to other molecular based HER electrocatalysts. However, a commercial electrode comprising platinum loaded on a membrane still exhibited roughly two orders of magnitude higher HER-based current density at a given potential^1677^ (not speaking for the consequent durability of Pt electrodes for the HER).

**Fig. 138:**
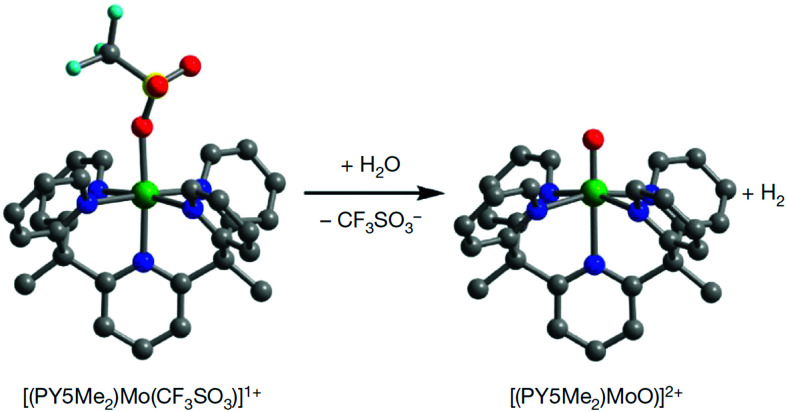
Reaction of [(PY5Me_2_)Mo(CF_3_SO_3_)]^1+^ with water to form [(PY5Me_2_)MoO]^2+^ and release H_2._ Reprinted with permission from ref. 1679. Copyright 2010. Nature publishing.

Organometallic oxo derivates that show activity as a catalyst for the water reduction reaction were introduced by Parkin and Bercaw.^1678^ High valency metal oxo species, namely [(PY_5_Me_2_)MoO(PF_6_)_2_)] (Fig. 138), right side shows the structure of the cation) with the pentadentate ligand 2,6-bis(1,1-bis(2-pyridyl)ethyl)pyridine (PY5Me_2_) have been exploited as a robust HER catalyst for real homogeneous water electrocatalysis by Karunadasa *et al.*^1679^

Since the metal cores in hydrogenases are in a sulphur-rich environment, the development of complexes using macrocyclic sulphur containing ligands was considered a promising bio-inspired design principle mainly pioneered by Sellmann *et al.*^1680–1683^

Moreover, the usefulness of using S-containing ligands, which can be guessed from the catalysts found in nature, was supported by theoretical considerations: synergy between metal- and ligand-based redox activities influences catalysts performance.^1684^ Especially the redox activity of a dithiolato ligand and a metal centre enables a complex redox behaviour consisting of multi-step electron transfer processes between delocalised π electrons and metal d-electrons.^1685^ The π back donating (electron rich-) sulphur is ideal to stabilise of low-oxidation-states in the central metal, allowing the existence of different metal hydride intermediates.

However, despite considerable success in the structure modelling of hydrogenases, the new biomimics show only a low level of activity in connection with high overvoltages, so immediate optimisation prospects appear to be quite limited. In addition, the low stability of some molecular species under electrolysis condition naturally questions whether it is purposeful to develop complexes with smartly designed organic ligands if at the end degradation and metal deposition occurs in a variety of aqueous media.^1686^ Unless it is known exactly whether the newly designed molecular catalyst is the catalytically active species or just the precursor for the active species, it is practically impossible to assign a specific catalytic activity to the metal complex. At the end of this section, the authors would like to go into the research results that have been developed over the last 5–6 years.

Polyoxometallate of the Keggin type has proven ability to work as OER electrocatalysts.^1600^ Very recently a series of Keggin type polyoxometalate (POM) based Cu containing metal-organic complexes have been synthesised, immobilised upon embedding into carbon cloth and checked as molecular HER electrocatalysts for heterogeneous water catalysis.^1687^ The organic was varied to evaluate a possible structure-activity relationship and the highest HER performance can be observed in 0.1 M KOH: *η* = 95 mV at *j* = 10 mA cm^−2^. Perthiolated coronene (PTC) ligand for complexation of Co was used leading to a catalyst with the formula [Co_3_(C_24_S_12_)]_*n*_ exhibiting unusual high conductivity (45 S cm^−1^). The Co-PTC catalyst deposited on carbon films showed a Tafel slope of 189 mV dec^−1^: *η* = 227 mV at *j* = 10 mA cm^−2^; pH 0.^1688^

**Fig. 139:**
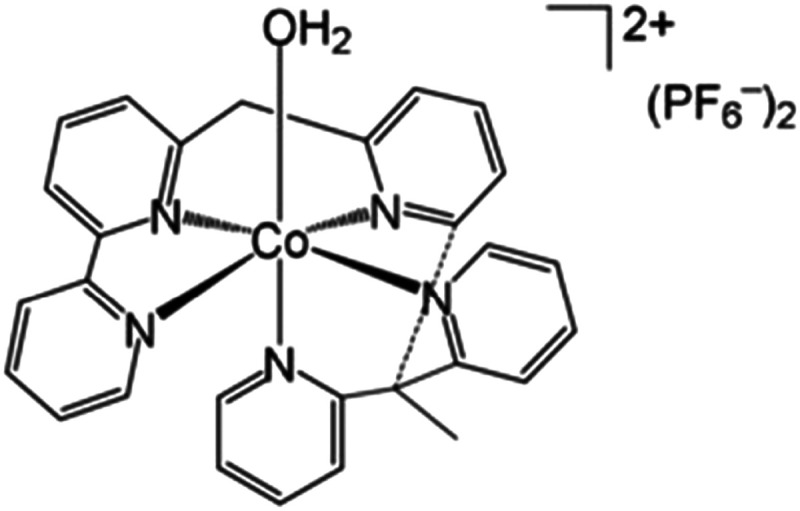
[Co(Py3Me-Bpy)OH_2_] (PF_6_)_2_. Reprinted with permission from ref. 1689. Copyright Wiley VCH.

As mentioned above, many metal organic complexes are prone to substantial degradation under electrocatalysis conditions. Webster *et al.* suggested to use soft pyridine groups to improve the stability of a low-valent Co^I^ complex during catalysis, thereby leading to higher HER activity.^1689^ Real homogenously catalysed HER was shown upon [Co(Py3Me-Bpy)OH_2_] (PF_6_)_2_ with Bpy = *N*,*N*-bis(2-pyridinylmethyl)-2,2′-bipyridine-6-methanamine (Fig. 139) in neutral phosphate buffered medium (pH 7): *j* = 10 mA cm^−2^ at *η* = 1000 mV was obtained with near-quantitative charge-to hydrogen-conversion rate.

In several recently published articles^1690–1699^ brilliant theoretical or experimental investigation of catalytic pathways and characterisation of intermediates, as well as highly-advanced structure-property relationships have been shown, which might guide future catalyst design of metal organic complexes. However, convincing activity and stability at least compatible with practical application, constitutes the vast exception.

Long-chain-like zirconium porphyrin-based coordination complexes were recently successfully fabricated *via* a two-step strategy.:^1700^ promising HER properties were measured (*η* = 60 mV at *j* = 10 mA cm^−2^; Tafel slope of 87 mV dec^−1^; 0.5 M sulphuric acid).

**Fig. 140:**
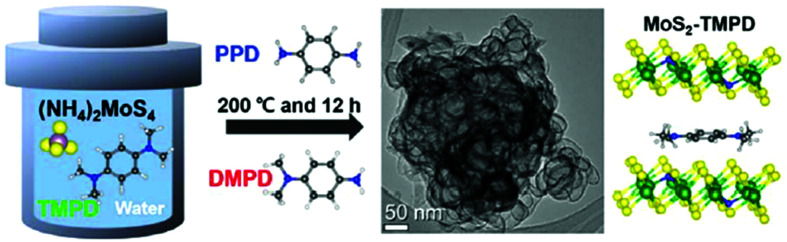
One-step procedure of hydrothermal reaction for the synthesis of MoS_2_ nanosheets that were intercalated with PPD, DMPD, and TMPD. Reprinted with permission from ref. 1708. Copyright Royal Society of Chemistry.

Immobilising a Ru-tannic acid (Ru-TA) coordination complex on activated carbon cloth (ACC) was recently reported;^1701^ due to the tight coordination between Ru^III^ and tannic acid in alkaline medium, the immobilised molecular Ru-TA/ACC electrocatalyst exhibits quite good HER efficiency (*η* = 29 mV; *j* = 10 mA cm^2^; 1.0 M KOH) which can be seen as a highly-competitive performance. Solid-state transition metal-chalcogenides like *e.g.* MoS_2_ are well known to actively support HER.^1702,1703^ Recent investigations show that metastable, semi-metallic 1T′ (distorted 1T) molybdenum disulphide present a particularly HER active phase.^1704–1707^ Kwak *et al.*^1708^ report on the hydrothermal synthesis of 1T′ phase MoS_2_ nanosheets that was intercalated with a series of alkylated *p*-phenylenediamine molecules (*p*-phenylenediamine (PPD), *N*,*N*-dimethyl-*p*-phenylenediamine (DMPD), and *N*,*N*,*N*′,*N*′-tetramethyl-*p*-phenylenediamine (TMPD) see Fig. 140.: intercalation goes hand-in-hand with substantial charge-transfer (0.40*e*, 0.73*e*, and 0.84*e* per molecule for PPD, DMPD, and TMPD), suggesting that the TMPD complex has the best HER activity. Indeed, for tetramethyl PD, one obtains *η* = 0.15 V at *j* = 10 mA cm^−2^ with a Tafel slope of 35 mV dec^−1^, underpinning the very good HER performance.

The coordinated metal centre does not present the catalytic active spot. The most active site is the nitrogen atom next to S vacancies as was shown by first principal calculations. Metal complexes in a way build a scientific bridge between the areas of homogeneous biological and heterogeneous solid-state catalysts. Challenges that scientist had to deal with include the low density of metal active sites compared to the overall size of the macromolecules and limited stability under electrolysis conditions. On the plus side we can mention the easiness regarding fine tuning and studying catalytic mechanism at a molecular level. Particularly when it comes to practical applicability, they fail in most of the relevant aspects. Classical heterogeneous catalysts lead to better apparent activity and durability, hence they are to date the only materials that can cope with practical water electrolysis.

## 11 Characterisation methods

Water electrolysis reactions are electrochemical reactions, and as such, any electrochemical technique has its own interest to evaluate catalyst materials, electrodes (usually in 3-electrode cell) or full electrolysis cells (2-electrode cell). However, water electrolysis reactions also convey a double specificity. Firstly, the reactions at stake, the HER at the cathode – negative electrode in a water electrolysis cell, and OER at the anode – positive electrode in a water electrolysis cell, are multiple step reactions; hence, reaction intermediates are produced/consumed, which one will need to measure/quantify, to unveil the reaction mechanisms (see Section 2), a prerequisite to the discovery of more active (and durable) electrocatalysts. Hence, a variety of physicochemical methods coupled to electrochemistry are used by the research community to assist mechanism and kinetics understanding. The most relevant, in the authors’ opinion, will be addressed hereafter. Secondly, both the HER and OER do generate gases (molecular hydrogen and molecular oxygen, respectively), which, if the rate of the reaction is sufficiently fast (a target for industrial systems), will not be purely dissolved (oversaturated) in the liquid electrolyte (water) but instead will generated bubbles.^822^ These bubbles will likely induce considerable difficulties in the electrochemical (and physicochemical) experiments and must therefore be considered, so that the techniques at stake are not biases by them. The present section aims at covering these aspects. Methods that are relevant to evaluate the performance of full electrolysis cells (2-electrode cells) will be addressed in Section 11.1; methods to evaluate individual electrodes (in 3-electrode cells) will be covered in Section 11.2 and finally, more advanced techniques to characterise the constitutive materials of the electrolyser (with special emphasis of the electrocatalysts, but also minorly on the membranes) will be addressed in Section 11.3. This Section 11 will introduce short-term performance characterisations and accelerated degradation tests (ADT) or accelerated stress tests (AST), whereas Section 12 will revisit most of the techniques for long-term durability assessment.

### 11.1 Two-electrode cell characterisations of the full electrolysis cell

**Fig. 141:**
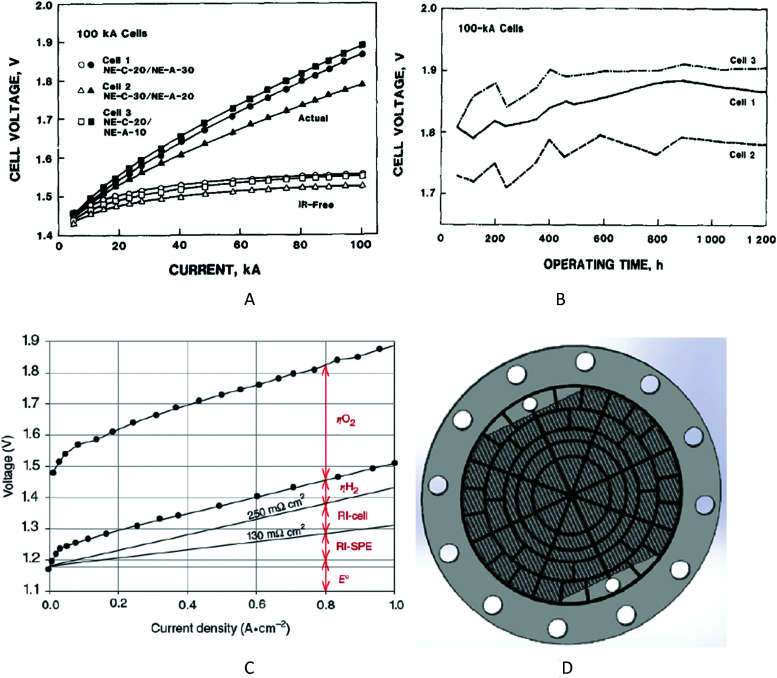
(A) Polarisation analyses of the three 100 kA Generation alkaline water electrolysers (AWE) of the Varennes experimental plant. A current of 100 kA corresponds to a current density of 0.25 A cm^−2^. (B) Long-term performance of the 100 kA cells of the Varennes experimental plant, monitored at a constant current of 100 kA (0.25 A cm^−2^). The electrolyte consists of 25% KOH at 70 °C. Reproduced from ref. with permission from Elsevier. (C) Typical PEMWE polarisation plots and corresponding individual voltage terms in the low current density range (0–1 A cm^−2^), for a temperature *T* = 90 °C and an overall pressure *P* = 1 bar. Reproduced from^1711^ with permission from Wiley-Verlag. (D) Device enabling local current density and temperature measurement during PEMWE operation. Reproduced from ref. 1712 with permission from Elsevier.

The most common and easiest mean to characterise a water electrolysis cell (in a non-destructive manner) is to perform measurements in the actual cell without intruding any external probe (without inserting any device in the cell for the measurement, *i.e.*, no reference electrode for 3-electrode cell measurements). In that case, acquiring polarisation plots, *i.e.*, the [current–cell voltage] characteristics in quasi-stationary conditions, is a widely-used methodology^1709,1710^ that is readily practicable for industrial cells (Fig. 141a, full symbols). The quasi-stationary conditions correspond to very slow solicitation of the system and enable to avoid any disturbance of the measured currents from capacitive effects that could be overwhelming for large-surface area electrodes (which is often the case in practical systems). The best manner to record a polarisation plot is to impose the current to the cell and measure its stable voltage (which may take a while), or to impose the cell voltage and measure the current drawn by the cell after its stabilisation. This is likely done by successive chronopotentiometric (resp. chronoamperometric) steps, whose duration should be long enough to enable the measured signal stabilisation prior any new jump to another quasi-stationary operating point. Then, longer-term chronopotentiometry (Fig. 141b) or potentiometry, enable to evaluate whether the cell performance can maintain *versus* time (Fig. 141b),^1710^ and these durability aspects will be more thoroughly addressed in Section 11.3. Polarisation plots are at the basis of any performance characterisation for studies dealing with water electrolysis, but are not always performed in a correct manner, *i.e.*, at sufficiently slow rate to avoid capacitive effects. There are indeed numbers of studies, where authors apply the measurement by using (cyclic) linear sweep voltammetry (CV/LSV) experiments at too high potential sweep rate, thereby resulting in pronounced capacitive currents. In such conditions, it is not unusual that some authors conclude that water electrolysis is possible below the thermoneutral voltage, which makes no sense, because some of the energy is provided to the water splitting in the form of heat, that is not quantified by the simple electrochemical signals (but that would definitely be consumed in real operation, at a cost). When the polarisation plot is properly acquired, it provides information regarding the various faradaic contributions to the cell characteristics. However, this information is not directly available without extra-analyses. For example, the raw polarisation plot depends on the whole cell (the contributions from the two electrodes cannot be separated) and is non-negligibly affected by the high-frequency resistance of the cell.

**Fig. 142:**
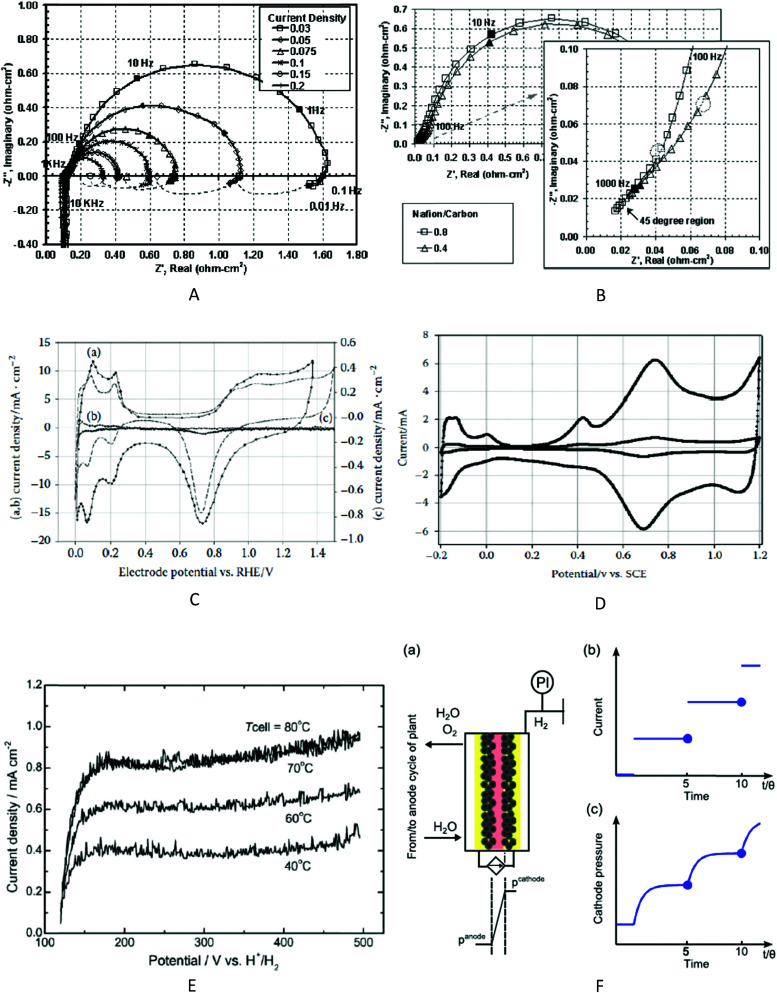
(A) Example of Nyquist plots of electrochemical impedance spectroscopy (EIS) measured on an operating unit proton exchange membrane fuel cell (PEMFC) in H_2_/O_2_ operation at several constant current densities. The membrane electrode assembly (MEA) is based on Nafion 112 Catalyst Coated Membrane with anode/cathode loading of 0.4/0.4 mg_Pt_ cm^−2^ and Nafion/carbon weight ratio of approximately 0.8; frequency range of 100 kHz–0.01 Hz; peak-to-peak perturbation of ±0.02 A cm^−2^. (B) Corresponding, complex-plane impedance for MEAs with Nafion/Carbon weight ratio of 0.8 and 0.4, respectively. Data corrected for pure resistance and inductance calculated from model. The 45° region enables to evaluate the proton resistance using a transmission line model. Reproduced from ref. 1715 with permission from the Electrochemical Society. Cyclic voltammetry of a (C) Pt/C-based cathode of PEMWE and (D) an IrO_2_-based anode before and after operation. Reproduced from ref. 1720 with permission from CRC press. (E) Example of H_2_ crossover measurement through the membrane in a PEMFC, as a function of the temperature. Reproduced from ref. 1726 with permission from Elsevier. (F) (a) Schematic view of the high-pressure water electrolyser test cell, (b) applied current profile and (c) resulting pressure profile during the experiment, with θ the characteristic time constant of the system, defined by the fraction of its permeance and its capacity. Reproduced from ref. 1727 with permission from Elsevier.

High-frequency resistance (HFR) measurement is a usual complement to polarisation plots^1713,1714^ the best manner to measure the cell HFR is electrochemical impedance spectroscopy (EIS), though other techniques like the current interrupt are also performed on occasion, this latter technique leading to large error if the time-constant of the measuring device is not sufficiently small. The methodology of HFR measurement in a water electrolyser is no different to that regularly applied for fuel cells,^242^ which has been practically democratised by the team of General Motors:^1715^ the cell high-frequency resistance can be measured as a function of operating parameters and of core (electrode and electrolyte) materials parameters (Fig. 142a). The intercept of the high-frequency loop with the real axis is the high-frequency resistance (on Fig. 142a), its value is *ca.* 0.09 Ω cm^2^, whatever the current density applied in the range surveyed). It is thanks to the measurement of the HFR that the (so called IR-free) polarisation plot can be corrected from the IR-drop, as performed in Fig. 142a (open symbols). The HFR originates from the conductivity and thickness of the electrolyte, the potential presence of bubbles (that not only lower the conductivity of the electrolyte, but also can mask the electrodes,^17,1716,1717^ not speaking from the fact they can mechanically destabilise the electrodes^822^), the internal resistance of the electrodes and current collectors and the interfacial contact resistance between these various materials.^822^ Many authors have attempted to unveil these different contributions,^17^ like for example the interfacial resistance between the membrane and active layers, and the membrane and electrodes’ contribution to the HFR.^1718^ Going deeper in the analysis of EIS data, one can evaluate the proton resistance in PEMWE (or PEMFC) electrodes, associated to the hindrance of proton transport within the composite electrodes as a function of electrode parameters and/or of the cell operating parameters. This however requires that the impedance analysis is made in conditions where the electrode that is targeted is the limiting one in the assembly. One manner to do this is to have one electrode maintained under H_2_ (it will thus play the role of counter electrode and reference electrode, as the HER/HOR are fast reactions) and the other in N_2_-purged water^1714,1719^ (it will play the role of the working electrode). In that case, the EIS of the working electrode will give insight into its own limitations, *e.g.*, by the proton-resistance (Fig. 142b). These methodologies, although exemplified for PEMFCs in Fig. 142 (and widely used in these systems^1715,1720^) can be applied to water electrolysis cells and start to be.^1714,1719,1721^

In Fig. 142c, the various contributions of the polarisation plot are separated for a classical PEMWE unit cell. One naturally sees the effect of the IR-drop, both brought by the solid polymer electrolyte (RI-SPE), the other components of the cell, *i.e.*, the electrodes, porous transport layers and interfaces between these components and the membrane (RI-cell). As the HER and OER are complex reactions, important contributions to the cell voltage depend on the overpotential associated to these reactions, noted *η*_H_2__ and *η*_O_2__ in Fig. 142c. These are connected to the activation (charge-transfer) overpotential values, *i.e.*, the intrinsic kinetics of the reactions on the considered electrocatalysts weighted by the developed electrochemical surface area (ECSA) of the active layers, and also to the mass-transfer overpotential values, that reflect the mass-transfer hindrance to/from the catalytic sites. These individual overpotential values can hardly be directly measured in 2-electrode cell (except by electrochemical impedance spectroscopy,^1722^ where the authors particularly evaluate how varying the porous transport layer at the OER electrode affects the mass-transport limitation in their cell) and will be addressed in Section 11.2 related to 3-electrode cell measurements.

Chronometric measurements like those of Fig. 141b enable to evaluate the coulometry of the reactions *versus* time, and, by combining measurement of the gas flows, one can evaluate the faradaic efficiency (FE) of the gas production.^1723^ Direct measurement of the H_2_ content in the O_2_ flow exiting the anode using a proper sensor (or on-line mass spectrometry^20^) also enables asserting the FE.^242,1724^ Usually, this efficiency is close to 100% when pure water is split, a dense separator (membrane) is used, and high current densities applied (like in industrial water electrolysis). Deviation from 100% FE is likely when impure water is electrolysed (see the example of sea water electrolysis in Section 13^1725^), when significant gas cross-over is experienced (likely in membraneless cells^17,822,1723^ – and this also has consequences in terms of safety of operation) and when the catalysts materials experience major degradation issues (see Section 5), but this is, again, usually not the case in practical state-of-the-art water electrolysis cells.

In conditions where one electrode of the cell plays the role of counter-electrode and reference electrode (which means it is operated under hydrated H_2_, see above), one can typically evaluate the response of the other electrode (which will play the role of the working electrode), *e.g.* by cyclic voltammetry^1714,1721^ This methodology, widely employed in fuel cells,^83,1728^ also finds applications in water electrolysers. Fig. 142c and d show typical characterisations of PEMWE electrodes,^1721^ where the active area can be followed in a non-destructive manner before/after PEMWE operation. The active area can simply be derived from the voltametric features of the working electrode in supporting electrolyte, *i.e.*, in absence of faradaic reaction (which is asserted when the working electrode is maintained in inert atmosphere, like N_2_ or Ar-saturated water). The technique is particularly suited for PGM-based electrodes (Pt/C or IrO_2_-based), as PGMs have well-defined signatures in supporting electrolytes. Besides, the cyclic voltammetry in such conditions can be employed (in H_2_/N_2_ or Ar) to evaluate the hydrogen crossover through the electrolyte (usually the membrane, and specifically performed in PEMFCs)^1726^ (Fig. 142e); in that case, though, the potential sweep rate must be very slow, so that the capacitive current of the working electrode is decreased (it scales with the potential sweep rate) sufficiently to let the faradaic contribution (*e.g.* H_2_ oxidation at the working electrode, following H_2_ crossover from the counter-reference electrode compartment) overwhelm the capacitive contribution; the working electrode then gives a plateau-like current corresponding to the mass-transfer-limited HOR current, the current at the plateau depending on the amount of H_2_ crossing over from the counter-reference electrode compartment. It must be noted, though, that IrO_2_ OER electrocatalysts are not the best suited for such H_2_-pump measurements.^1724,1729,1730^ Bensman *et al.* reviewed techniques that can be used to measure the H_2_ crossover in operating water electrolysers,^1727^ and they proposed a refined method (the so-called current compensation technique) to measure the H_2_ crossover in pressurised electrolysers (Fig. 142f). The permeate flux is compensated by an electrochemical gas evolution reaction at the electrode of the high-pressure side to maintain steady-state conditions, and the required current is measured, leading to a direct quantification of the H_2_ crossover. Their procedure allows *in situ* quantification of hydrogen crossover in assembled PEMWE cells under electrolysis conditions, without the need for inert gases or external sensors. One must note that such crossover of H_2_ plays a non-negligible role on the durability of the water electrolyser catalysts, and in particular of its OER anode.^20^

When relevantly performed, 2-electrode cell operation enables measuring in a very precise manner the kinetics of water electrolysis (and fuel cell) reactions. In that case, the one electrode (counter and reference, fed with hydrated H_2_) shall be reasonably loaded in catalyst, not to be limiting *versus* the other electrode (working), which shall on the contrary be made limiting on purpose, *i.e.*, by having “minimal” loading of catalyst. This mode of operation is valid to evaluate OER/ORR and HER/HOR catalysts, as relevantly performed by Gasteiger *et al.*^42,84^ For HER/HOR evaluation, the hydrogen pump mode was used, which enabled to measure the HER/HOR kinetics with minimal limitations from mass-transport hindrance, a usual issue in the characterisations of very fast reactions, as is the HER/HOR.

Whereas these methodologies have mostly been employed to characterise PEMFCs, there is no real limitation for their application to water electrolysis cells. Besides, although they require that the electrolyser is not in normal operation for the measurement, they are fully applicable without dismantling the cell, a great advantage in terms of non-destructive (hence fast, possibly on-site) diagnostics of the electrolysis cell.

In complement, authors recently proposed home-made designed and built segmented unit cells that enable such measurements at the local scale within a MEA.^1731,1732^ Segmented sensor plates equipped for local current density and temperature measurements (Fig. 141d) are also available for such measurements and have been applied to unit PEMWE.^1712^ These techniques were firstly proposed for fuel cell characterisations^1733–1739^ as recently reviewed.^1740^

Additional measurements are also possible at the scale of a unit water electrolysis cell (or even the stack). For example (and without being exhaustive), compression of the water electrolyser MEA can be evaluated using a pressure-sensitive film, which gives indications on whether the compression is homogeneous (or not) on the whole MEA surface,^1712,1741^ which has an impact on the cell operation. Using a precision Ohmmeter also enables to quantify the contact resistance between some of the cell components (*e.g.*, the bipolar plate and the porous transport layer) as a function of the stack assembly (compaction) pressure.^1741^

Whatever their interest and ease of application, two-electrode cell measurement in real electrolyser cells are insufficient if one wants to access the intrinsic activity of the electrocatalysts, so as to properly evaluate the reaction overvoltage values. In that case, one needs to perform 3-electrode cell measurements.

### 11.2 Three-electrode cell characterisations of individual electrodes

In complement to 2-electrode cell measurements (which are possible at industrial facilities), laboratory researchers usually perform 3-electrode cell measurements, in which they independently control the potential of the working electrode (WE) and counter-electrode (CE) *versus* a properly chosen reference-electrode (RE). The nature and position of the RE should be optimal to enable noiseless measurements, and not bias the operation of the WE (it shall not mask the surface of the WE to avoid disturbance of the current lines, be sufficiently close of the WE to limit the Ohmic drop, and not lead to the pollution of the electrolyte, which is possible *e.g.*, with Cl^−^ containing references, Cl^−^ being a poison to many electrocatalysts encountered in water electrolysis, *e.g.*, Pt^1742^). By using a RE, the electrochemical signal of the WE can be isolated from that of the CE, and Ohmic-drop correction can be performed in a dynamic manner (although this can yield difficulties under bubbles evolution regime, where the conductivity of the electrolyte may non-negligibly change in operation, hence rendering awkward precise and direct Ohmic-drop correction). Three-electrode cell measurements are also compatible with experiments in which the CE compartment is separated from the working electrode (by a membrane or a glass frit), thereby limiting the influence of the CE (because it produces by-products in operation) on the behaviour of the working electrode. Nevertheless, it is always required to adopt the “proper” CE, both in terms of nature of its constitutive material, and in terms of surface area (so that the potential difference between the WE and CE is not limited by the compliance of the potentiate, which can become critical if large currents are experienced and/or if the Ohmic drop is non-negligible). The nature of the CE should be chosen to sustain the WE current (not to be counter-electrode limited) and does not pollute the electrolyte by products of its major or side reactions.^1743^ In that prospect, metallic CE may dissolve, hence favouring deposits at the WE surface, this phenomenon being likely when the working electrode is in reduction (*e.g.*, under HER) and the counter electrode in oxidation (*e.g.*, OER in competition with metal dissolution). This effect is well-known by the community and recalled in several “good practice papers”.^1744,1745^ It can nevertheless be used as an advantage to “activate” an electrode at minimal materials’ cost^1269^ (in that case using the dissolution of a Pt CE to provoke subtle deposition of Pt at the WE surface), even though plenty examples of the literature suffer such effect in an uncontrolled manner (not even evoked by their authors).

**Fig. 143:**
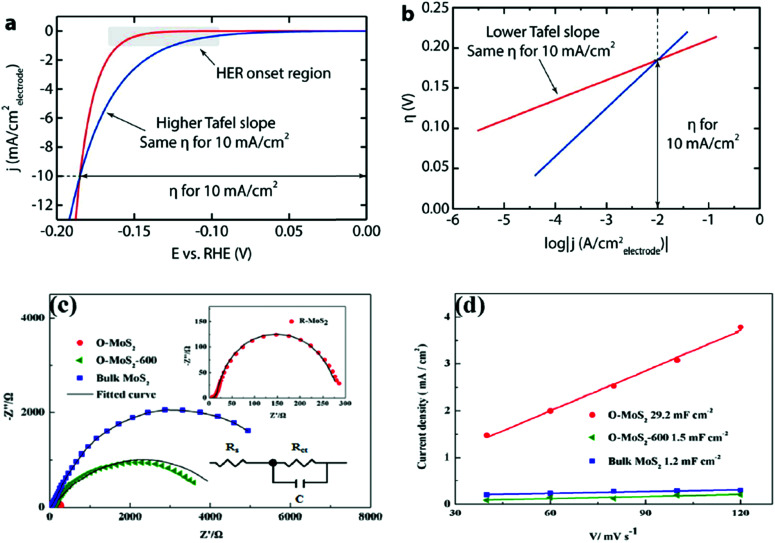
Basics of water electrolysis kinetic markers’ determination, for the example of the HER. (a) HER onset potential and overpotential at a current density of 10 mA cm^−2^ and (b) corresponding Tafel slopes. The blue electrocatalyst would be better for operation at low current density, while the red one would be better at high current density (i.e., in an industrial water electrolyser). Reproduced from with permission from ref. 1746 with permission from the American Chemical Society. (c) Example of electrochemical impedance spectroscopy measurements (EIS) enabling double layer capacitance measurements and (d) similar determination of the double layer capacitance from cyclic voltammetry measurements. Reproduced from ref. 1755, with permission from Elsevier.

Polarisation plots can be easily measured in 3-electrode cells, usually in the rotating disk electrode (RDE) configuration, that enables to control the mass-transport rate, hence operate in quasi-stationary conditions. However, for water electrolysis reactions, bubbles of H_2_ or O_2_ cannot be avoided, leading to issues in the measurements, especially at high current density; for that reason, modified RDE setup have been proposed in the literature, that enable more reliable measurements at high current density.^244^ Polarisation plots obtained in RDE or modified RDE configuration may be used to isolate “Tafel slopes”, a widely-used marker of the catalytic activity of a given material towards the reaction at stake (Fig. 143a and b). From these, additional activity markers’ can be determined, like onset potential and overvoltage at a given current density,^1746^ possibly after Ohmic-drop and mass-transport corrections (the latter being usually non-necessary for water electrolysis reactions, owing to the fact that the reactant is water, *i.e.* the solvent, at least if the generated bubbles are “properly” expelled from the electrode surface, this is normally the case in RDE, except when porous active layers are used-in these, bubbles might be trapped inside the pores of the active layer-see below).^20^

3-Electrode cell measurement are ideal for ECSA characterisations because they enable to really isolate the behaviour of the working electrode. These measurements are possible for PGM-based catalysts, either by hydrogen underpotential deposition (Pt, Pd), CO-stripping (Pt, Ru), metal oxide reduction (all PGM and alloys, as exemplified in^1747–1750^). When the electrocatalyst is non-PGM, only the latter technique makes sense,^1286^ but is not necessarily very practical. For Ni-based catalysts, integrating the peaks relative to the Ni^II^/Ni transition enables to assess the developed area of metallic nickel, while that of the Ni^III^/Ni^II^ transition enables to evaluate the active area of oxidised nickel,^96,1751,1752^ similar measurements being also possible with Co-oxide based catalysts.^1753^ For materials like MoS_2_, transition metal oxides, (including noble ones^1754^) *etc.*, one can simply measure the double layer capacitance of the catalyst material in a potential region where the electrode does not lead to quantitative change of oxidation state (by cyclic voltammetry^833^ or by EIS,^1755^Fig. 143c and d), or in a region where a characteristic redox is witnessed by cyclic voltammetry,^244,1755^ these values possibly being calibrated *via* sorption isotherm measurements.^1756^

A very important aspect of electrochemical characterisations of water electrolysis catalysts is to find experimental markers to quantify the initial catalytic activity for the desired reaction (HER or OER), or (better) at the same time the activity and short-term stability of this activity. Indeed, as stated in opening of this section, the operating conditions of water electrolysis are very harsh (highly reducing conditions at the cathode, and highly oxidising conditions at the anode, not speaking from the hindrances connected to the evolution of gas bubbles and the rather high temperature and operating current density of industrial cells). To that goal, authors regularly propose metrics, and some relevant ones are listed hereafter.

**Fig. 144:**
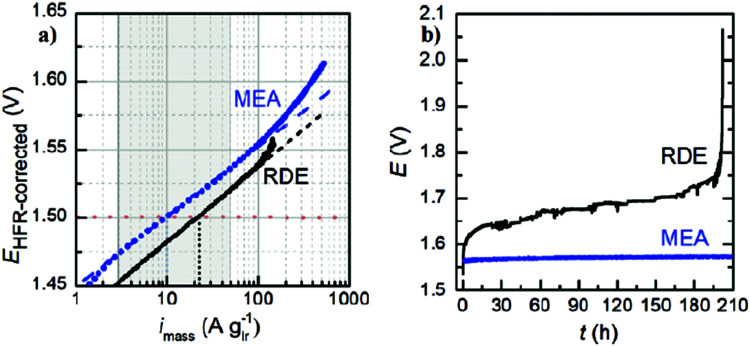
Evaluation of the OER (a) activity and (b) stability *versus* time at a given representative OER current in MEA (blue) and RDE (black) configuration for a state-of-the-art commercial IrO_2_ catalyst. Reproduced from ref. 20 with permission from Wiley.

An example of figure-of-merit is the electrocatalyst ability to exhibit the lowest overvoltage when delivering a small (not to be limited by mass-transport) but non-negligible (not to be biased by capacitive currents) current (*e.g.*, 10 mA cm^−2^ in absolute value) of HER or OER (Fig. 144a). Other authors propose to compare the mass or specific activities of the catalyst materials,^244,1752,1757^ usually evaluated at a relevant electrode potential value. Used in combination with the proper ECSA characterisation of the catalyst, these markers enable to assess the turnover frequency (TOF) or turnover number (TON) of the catalytic sites at stake.^1757^ A refinement is to evaluate the overpotential value measured at a relevant current density (*e.g.*, +/− 10 mA cm^−2^) *versus* the same after 2 h of operation.^833,1758^ Another metric is the so-called “stability number” which was recently proposed to benchmark electrocatalyst stability from 3-electrode cell measurements; it has been set for Ir-containing catalysts and is defined as the ratio between the amounts of evolved oxygen and dissolved iridium, thereby linking the activity to the stability of the OER materials.^1759^ Thanks to this methodology, Cherevko *et al.* proposed that for many OER catalysts, the activity scales inversely to the durability (evaluated in the short-term), which would mean that active catalysts would not be durable in operation.^1759^ This vision is however not unanimous, others claiming that accelerated degradation tests performed in 3-electrode cell measurements at the lab scale do not necessarily match real water electrolysis data, and that real electrolyser cell experiments only should be used to evaluate the catalysts’ durability.^20^ One illustration of this drawback of 3-electrode cell measurements was recently provided by the group of Gasteiger: chronopotentimetric measurements performed in the RDE setup, fail to provide information on the long-term stability of nanostructured OER catalysts, as a result of the bubbles build-up in the volume of the thin layer of catalyst immobilised at the RDE tip (Fig. 144), the mass-transport in RDE being incapable to effectively evacuates these trapped bubbles in long-term RDE operation.^20,1760,1761^ They however remark that (short-term) catalytic activity of HER and OER can be relevantly assessed in RDE configuration.^20^

This short literature review shows that bridging the short time scale fundamental experiments to the long-time scale in real operation is therefore still very needed, and indeed, the research community actively addresses the issue nowadays. More insights into this topic will be provided in Section 12.

### 11.3 Physicochemical techniques coupled to electrochemistry to unveil how water electrolysers and their core materials operate

Several *ex situ* materials characterisation techniques (electron microscopies, X-ray diffraction, Raman and infra-red spectroscopies, chemical or elemental analyses, *etc.*) find their interest to determine the (initial or post-test) composition and microstructure of water electrolysis catalysts and evaluate whether these properties are positive or negative with respect their catalytic activity for the HER and OER and/or their durability in operation. When used *ex situ*, these techniques are common for scientists of the field of fuel cells/electrolysers and are by no means specific to water electrolysis. In that context, they will not be addressed here in more details. Other advanced *ex situ* techniques enable to probe the surface composition and/or electronic states of catalysts; atomic probe tomography,^1762^ X-ray emission spectroscopy (XES) and X-ray photoelectron spectroscopy (XPS)^1763^ are for example encountered in the recent OER literature to explore the fine composition and electronic properties of OER catalysts. *Ex situ* (and non-necessarily associated with electrochemistry) methods are also used to characterise non-catalytic material of the water electrolysis cell. A few examples are provided hereafter in a non-exhaustive manner. Porosimetry (*i.e.*, mercury intrusion porosimetry^1732^) enables to assess the gas transport properties of electrodes and porous transport layers (PTL).^1711^ The wetting properties of the PTL are also of importance since these drive the nucleation and evacuation of the bubbles from the PTL surface in liquid water.^1764^ Basic corrosion and interfacial contact resistance measurements enable to test potential bipolar (or separator) plate materials.^241^

Now, the real endeavour in characterising water electrolysis materials processes is to perform such characterisations under current load, *i.e.*, *in situ* or *operando*. Such methodologies have really been democratised for two decades, from the fast and remarkable development of numerous physicochemical characterisation tools, that are available at synchrotron beamline, or even at the laboratory scale. The most striking of them are listed hereafter, in a non-exhaustive manner, recent reviews providing more depth on this matter.^1765,1766^

One technique of choice when it comes to water electrolysis is to detect the gas bubbles, using tailored cells with windows and fast video-cameras, when then help to model the hindrance of the bubbles on the cell performances.^1717,1767^ This is particularly important for membraneless systems (*e.g.*, alkaline water electrolysers) in which bubbles compromise the ionic conduction in the electrolyte, can favour products intermixing, hence decrease of the FE safety issues.^240^*Operando* dynamic specific resistance measurement was also proposed to evaluate how gas bubbles do detach during the OER on vanadate-modified surfaces.^1768^ Such observations are often at the basis of modelling of the electrolyser operation,^1769^

On-line gas chromatography^238^ or mass-spectrometry^1770^ are useful when it comes to analyse the purity of the H_2_ or O_2_ gases that exit the cell (two-electrode operation, in real water electrolyser cell); they can also be used in more model conditions (3-electrode cell), to evaluate the capabilities of one material towards the desired reaction and to probe possible (gas-evolving) parasitic reactions (*e.g.* Cl_2_ evolution in sea-water electrolysis, CO_2_ formation from carbon oxidation). Differential electrochemical mass spectrometry (DEMS) enables such measurements and can quantify gaseous or volatile species,^110,1771^ particularly in transient (non-stationary) conditions, *e.g.*, during accelerated degradation tests. DEMS or on-line EMS can be used with isotopic materials and water, to further shed light on the activity or degradation mechanisms, for example to illustrate whether lattice oxygen from metal oxides is evolved or not during OER.^1772^

**Fig. 145:**
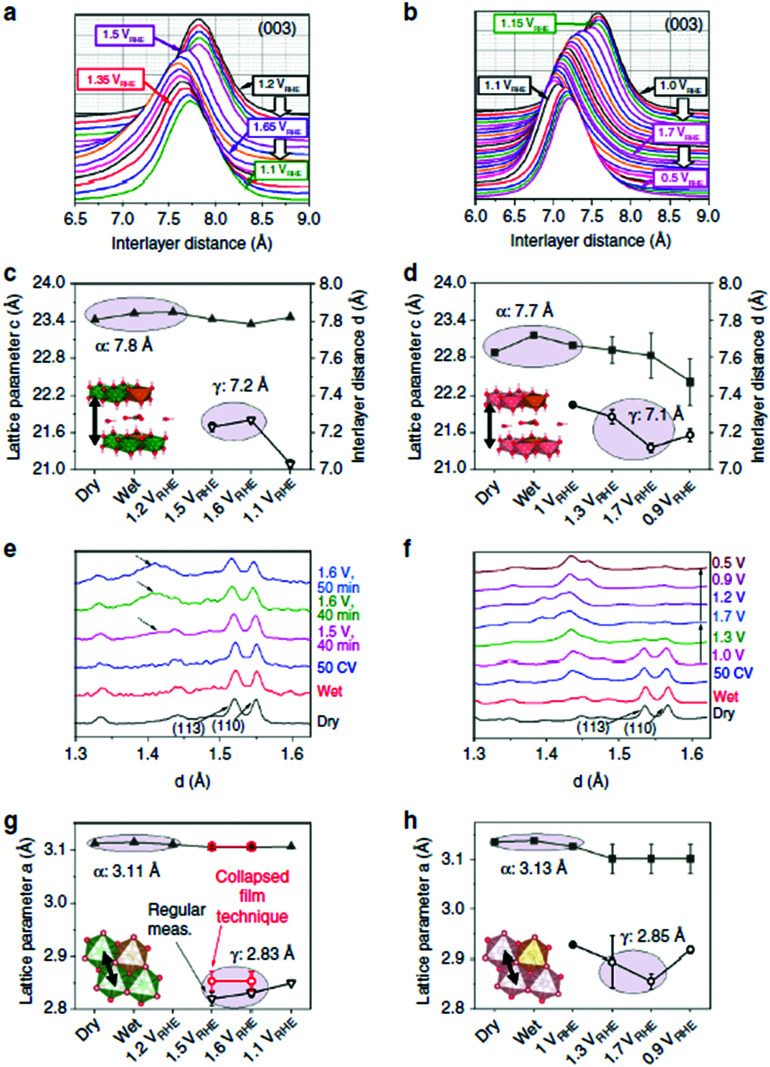
Evolution of the interlayer spacing and intralayer metal–metal distances of NiFe and CoFe LDHs from WAXS measurement. (a and b) Normalized and background-subtracted (003) peak obtained during *in situ* WAXS in 0.1 M KOH and potential steps for NiFe LDH (a) and CoFe LDH (b). (c and d) Interlayer distances for NiFe LDH (c) and CoFe LDH (d) obtained by Rietveld refinement. (e and f) *In situ* WAXS patterns for d-values close to the (110) peak of NiFe LDH (e) and CoFe LDH (f). For NiFe LDH, the WAXS patterns at the reported potentials were obtained by the collapsed film technique. In e, the dashed arrows highlight the feature associated to the γ-phase. (g and h) Lattice parameter a, corresponding to the intralayer metal–metal distance in NiFe LDH (g) and CoFe LDH (h) obtained by Rietveld refinement. Full and open symbols are used for different phases. Error bars represent SD provided by Topas for the refined parameters. Reproduced with permission from ref. 1775 Copyright Springer-Nature 2020.

X-ray are unique probes when it comes to *in situ* or *operando* characterisation of catalytic materials. X-ray Absorption Spectroscopy (XAS) enables the analysis of the chemical state, oxidation state of water electrolysis catalysts in operation (under potential control), and can enable to reconstruct the surface structure of operating active sites, an endeavour into the elucidation of the complex OER or HER mechanisms.^110,1752,1753,1771–1774^ XAS has for example been coupled with *operando* X-ray scattering and density functional theory (DFT) calculations, to unveil the catalytically active phase, reaction center and the OER mechanism of NiFe and CoFe (MFe) layered double hydroxides (LDHs) catalysts for the alkaline OER.^1775^ High-energy X-ray diffraction can also be performed *operando*, leading to the fine structure of the nanostructured catalysts upon water electrolysis; performed on IrNi@IrOx core–shell nanoparticles and combined with XAS and DFT calculations, it enabled to assert that lattice vacancies are generated following nickel leaching during the catalyst's activation, thereby producing shortened Ir–O metal ligand bonds and larger number of d-band holes in the iridium oxide shell, which overall increases the materials OER activity.^1776^*Operando* wide angle X-ray scattering (WAXS) complements the picture, enabling to access very fine geometric parameters of the catalyst materials’ lattice upon operation^1775^ (Fig. 145).

Near-ambient pressure X-ray photoelectron spectroscopy (nap-XPS) cannot be considered a real *operando* technique for water electrolysis; however, it enables to evaluate the state of surface of catalysts materials when in contact with *ca.* 20 mbar of gaseous species (*e.g.*, H_2_, O_2_, H_2_O), which can provide insights into the behaviour of the materials in real operation.^1765,1777–1781^ Like for other *operando* spectroscopies, these measurements are only possible provided the *in situ* cell and operating conditions are optimised both for the electrochemical and spectrometric insights, a difficult task at electrified solid|liquid interfaces on which gas bubbles are permanently released.^1780,1781^

Raman spectroscopy is a powerful tool to characterise oxides and was historically used prior/after electrochemistry to unveil how catalysts changed upon OER operation.^1782^ Until recently, *operando* Raman was not conducted to characterise water electrolysis reactions, because of obvious experimental issues induced by the unavoidable bubbles’ evolution. The picture changed starting in 2011 when Yeo and Bell performed *in situ* Raman spectroscopy to evaluate cobalt oxide OER catalysts.^771^ Then in 2015, Kornienko *et al.* combined *operando* Raman spectroscopy and XAS to characterise CoS_2_ catalysts under HER regime;^1783^ their results enabled to build a molecular model in which the cobalt atom is in an octahedral CoS_2_-like state and is surrounded by a first shell of sulphur atoms, the latter being preferentially exposed to electrolyte relative to bulk CoS_2_. They proposed that such CoS_2_-like clusters are generated in cathodic polarisation, thereby exposing a high density of catalytically active sulphur sites for enhanced HER. Other studies using Raman spectroscopy soon followed,^1768,1784–1790^ demonstrating its clear interest to unveil catalysts’ structural changes upon operation, elucidate their possible active sites and the intermediates formed during (water) electrolysis.

Electrochemical quartz crystal micro/-nanobalance is also a reported technique to survey water electrolysis catalysts.^1785^ Firstly, demonstrated for very model Pd surfaces,^1791^ it has since them been used for more practical nanostructured catalysts.^1792–1794^

Inductively coupled plasma mass spectrometry (ICP-MS), a classical technique for trace analyses, was recently coupled on-line to electrochemistry by the group of Mayrhofer. Initially demonstrated for corrosion applications and then fuel cell catalysis, the technique has been employed with great success to probe the short-time stability of water electrolysis catalysts, upon fast-potential variation experiments.^1795–1799^ However, this tool is employed, so far, with liquid electrolytes and in operating conditions that may non-negligibly differ from the real application, and therefore it has yet to be demonstrated that the conclusions deriving from such measurements fully apply to the same catalyst materials when operated in real water electrolysers.^20^

**Fig. 146:**
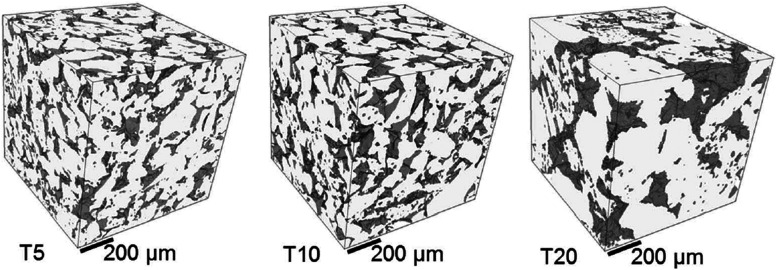
Example of tomographic elucidation of a PTL porous structure. Reproduced with permission from ref. 1801. Copyright Elsevier 2017.

Because the management of bubbles and liquid water is critical in low-temperature water electrolysers, and because this largely depends on the porosity and porous structure of the catalyst layers and porous transport layers, X-ray tomographic microscopy imaging is popular to study PEMWE electrodes and unveil their porous structure/morphology^1800,1801^(Fig. 146). By measuring the influence of the PTL structure on the mass transport overpotential *versus* the current density, operating pressure and temperature, the authors^1192^ demonstrated that the interface properties between the catalyst layer and the PTL had a major influence on the cell performance.

Water management in a water electrolyser is a critical issue, as is in PEMFECs or in AEMFCs. It can be surveyed by Small Angle Neutron Scattering (SANS) in operating cells, as initially demonstrated by Morin *et al.* in operating PEMFC^1802^ and lately applied to evaluate water electrolysis membranes.^1803^ Neutron imaging methodologies now start to be used for two-phase flow investigations in the porous structure of PEMWE electrodes and PTL,^1804^ which enables unveiling the mass-transport mechanisms.^1805^

This selected literature review demonstrates that the research community is very active and inventive to finds manner to elucidate complex problems. The techniques listed here have all great interest to improve water electrolyser materials and cells. However, long-term operation and durability in these conditions can only be relevantly assessed by tests performed in real electrolysers, which is the topic of Section 5.

## 12 Enhanced water splitting with externally applied fields

In general, in electrochemistry, the overpotential (*η*) of a galvanic and electrolytic cell is made of three important components: the activation overpotential (*η*_activation_), the Ohmic overpotential (*η*_Ohmic_) and the concentration overpotential (*η*_concentration_), each term having an impact on the cell efficiency. Low-temperature water electrolysers have many assets, although they suffer from molecular hydrogen and oxygen bubble accumulation at the electrode surfaces and in the electrolyte, leading to a high Ohmic voltage drop (IR) and a large reaction overpotential in turns yielding high operational energy consumption and costs.^1806,1807^

H_2_ and O_2_ gas bubble evolutions during electrochemical water splitting lead to electrochemical losses, owing to the fact that the electrochemical reaction rates for both reactions are purely controlled by the interfacial phenomenon in the three-phase zone (TPZ) where H_2_ and O_2_ gas bubbles, electrolyte and electrode surface are in contact with each other.^1808^ In first approximation, the practical cell voltage (*V*_cell_) for electrochemical water splitting technologies obeys eqn (19).^1806–1808^

19 *V*_cell_ = |*E*_c_ − *E*_a_| + *I* × ∑*R* = *E*^rev^ + |*ηa*| + |*ηc*| + *I* × (*R*_c_ + *R*_m_ + *R*_b_ + *R*_e_)

where *E*_c_ (or *E*_HER_) is the HER cathode potential, *E*_a_ (or *E*_OER_) is the OER anode potential, *I* is the applied current, ∑*R* is total Ohmic resistance, *E*^rev^ is the reversible potential (Nernst), *η*_a_ is the anode overpotential, *η*_c_ is the cathode overpotential, *R*_c_ is the circuit resistance, *R*_m_ is the membrane/separator resistance, *R*_b_ is the bubble resistance, and *R*_e_ is the electrolyte resistance.^1809^

Eqn (19) shows that *V*_cell_ depends greatly upon the overpotential and Ohmic voltage drop and therefore, reducing the anodic and cathodic overpotentials (*η*_a_, *η*_c_) and the total Ohmic resistance (∑*R*) is paramount to reducing energy consumption. During water electrolysis, *R*_c_ and *R*_m_ are usually constant and can be reduced by better wiring and membrane/separator optimisation. However, it is not the situation for *R*_b_ as many evolved gas bubbles generated on the electrode surfaces act as an insulating layer (similar to “passivation”), which significantly reduces the effective electrode surface area (*A*_eff_). In this case, the bubble coverage (*θ*) on the electrode surface yields increased bubble resistance, *R*_b_. This fraction of the electrode surface covered with “sticking” *i.e.*, adhering gas bubbles is well-known to affect substantially: (i) the mass (*m*) and heat (*h*) transfer, (ii) the limiting current density (*j*_lim_), (iii) the overpotential and (iv) the Ohmic resistance (∑*R*). In other words, when the evolved gas bubbles cover the electrode surface, they cause electrolyte access blockage and lead to reactant starvation resulting to an exponential increase of the cell voltage with the current density (*j*). Since the Ohmic resistance and the overall cell overpotential depend on bubble surface coverage, *θ*, effective gas-bubbles removal at the electrode surface should in theory reduce the cell voltage.^1808^ Additionally, the dispersion of the bubbles in the electrolyte decreases its conductivity and in turns increases *R*_e_ and thus, the current distribution on the electrode surface increases yielding high cell voltages.^1810–1812^

In general, hydrogen and oxygen gas bubbles evolving on the electrolyser electrode surfaces and in an electrolyte affect: (i) *η*_activation_ as the adhering bubbles decrease *A*_eff_, (ii) *η*_Ohmic_ due to a blockage of ionic pathways available for electronic transport, and (iii) *η*_concentration_ due to the dissolved gas products and the decrease in supersaturation levels within the electrolyte. There are several methods for reducing the total overpotential and total Ohmic resistance in water electrolysis, for example, by either increasing the electrolyte movement *i.e.*, mass-transfer, by using gravity,^1810,1811^ by centrifugal acceleration field,^1812^ by mechanical stirring,^1813,1814^ by using a magnetic field,^1809,1812–1820^ or by employing ultrasound,^1821–1836^ at the gas-evolving electrodes and electrolyte.

### 12.1 Mechanical stirring

Many studies have shown that stirring the electrolyte away from/at the electrode surface affects gas-bubble evolution and hence bubble coverage.^1813,1814^ Eigeldinger and Vogt^1813^ demonstrated that electrolyte flow past the electrode surface strongly affects the fractional bubble coverage and increasing the flow rate lowers the bubble coverage, increases efficient gas bubble removal at the electrode surface, and in turns reduces the Ohmic resistance, electrode overpotential and the limiting current density. However, as stated in Section 9, mechanical stirring only affects the “surface” of the electrode and not its inner porosity, in which bubbles might remain trapped. This is even the case in small-scaled porous rotating disk electrode layers, as put forth by the group of Gasteiger.^1760,1761^

### 12.2 Magnetic field

Magneto-electrochemistry is a niche area of electrochemistry that has been around for over 40 years, in which magnetic fields are applied to electrochemical systems. It was found that magnetic fields affect mass-transfer, limiting current density and charge-transfer due to Lorentz and Kelvin forces, magneto-hydrodynamics (MHD), chiral-induced spin selectivity, and hyperthermia (local heating of the electrode materials).^1815^ A recent contribution of some of the authors reviews magnetic effects in electrochemistry.^1815^

In the literature, there are several studies that focus on applying magnetic fields to water electrolysis. Overall, magnetic forces (Lorentz and Kelvin) improve bubbles’ removal at the electrode surface, enhance mass-transfer, reduce cell voltage and electrolyte/electrode Ohmic resistance. Employing ferromagnetic catalysts can yield improved efficiencies than those using paramagnetic and diamagnetic catalytic materials.^1809,1816^

For example, Iida *et al.*^1817^ reported improved water electrolysis efficiencies by reducing the electrode overpotential in a magnetic field under alkaline (4.46 and 0.36 M KOH) and acidic (0.05 M H_2_SO_4_) conditions. The OER overpotential was further reduced than the HER overpotential under the presence of a magnetic field, due to the different gas bubble sizes from both processes. They associated the findings to MHD convection, that affects bubbles’ detachment at the electrode surface, leading to a significant reduction of the void fraction and surface coverage by the gas bubbles: MHD convection plays an important role for bubbles’ nucleation, growth and detachment.^1818^

Using a specially-designed electrode (transparent glass) for AWE, Matsushima *et al.*^1819^ showed that the magnetic field (1.0 T) affects gas bubble removal remarkably due to MHD convection. Lin *et al.*^1820^ applied simultaneously pulse potentials (up to 4 V) and magnetic fields (up to 4.5 T) to Ni electrodes immersed in KOH. By applying this strategy, they managed to reduce power consumption by 88% with a 38% increase in current compared to conventional DC electrolysis. Kaya *et al.*^1837^ showed that by using cost-effective graphite (anode) and high carbon steel (cathode) electrodes immersed in low KOH concentrations (5–15 wt%) and in the presence of a magnetic field, higher hydrogen production rates (up to 17%) when compared to conventional conditions were achieved. They attributed the findings to efficient hydrogen and oxygen gas bubbles removal at the electrode surfaces caused by MHD convection. The same group^1838^ demonstrated that by applying a magnetic field (0.5 T) on a single PEMWE cell could improve performances up to 56% (@ 2.5 V), particularly at lower flow rates, where Lorenz and buoyancy forces are predominant towards gas bubbles’ removal.

The use of magnetic field to improve water electrolysis is currently seen as a promising method to reduce the so-called “bubble overpotential”, to minimise power consumption and thus to increase electrolyser efficiencies. As an example, in 2021, the European Commission granted a 4 year project (June 2021–May 2025) under the EU Horizon 2020 programme entitled “Spin-polarised Catalysts for Energy-Efficient AEM Water Electrolysis – SpinCat”.^1839^ SpinCat develops a series of novel magnetic earth-abundant catalysts that can enhance OER catalytic activity by a factor of three *via* the use of magnetic fields (spin polarisation) as compared to state-of-the-art OER catalysts.

In an alternative approach, some of the authors of the contribution used alternative magnetic field and magnetic@catalytic (FeC@Ni core–shell) nanoparticles to heat the latter to their Currie temperature and promote enhanced HER and OER.^29^ It is possible that other magnetic effects as those listed above and recalled in ref. 1815 are also at stake in their experiments.

### 12.3 Ultrasound in water splitting

Another method is to apply power ultrasound,^1840^ sonochemistry^1841^ (ultrasound in chemistry) and sonoelectrochemistry^1841,1842^ (ultrasound in electrochemistry) in the solution and at the gas-evolving electrode. The use and application of ultrasound in chemical, physical and biological sciences can be divided into two distinct groups: (a) low frequency ultrasound or *power ultrasound* (20 kHz–2 MHz) and (b) high frequency ultrasound or *diagnostic ultrasound* (2–10 MHz). Power ultrasound (PUS), a process intensification technology, is regarded as the propagation and the effect of an ultrasonic wave when transmitted through a liquid, leading to (i) the creation of cavities (or voids) and cavitation bubbles (acoustic cavitation bubbles) as well as (ii) acoustic streaming.

#### 12.3.1 Sonochemistry in water splitting

Sonochemistry is a relatively new concept that received attention in the late 1970's and has been defined as the application of ultrasound in chemistry. In the late 1980's and early 1990's, the area was revived by Mason^1843^ and Suslick.^1844^ The significant effects caused by acoustic cavitation in a liquid is the “Sonochemistry and Sonoluminesence”.^1843–1845^

**Fig. 147:**
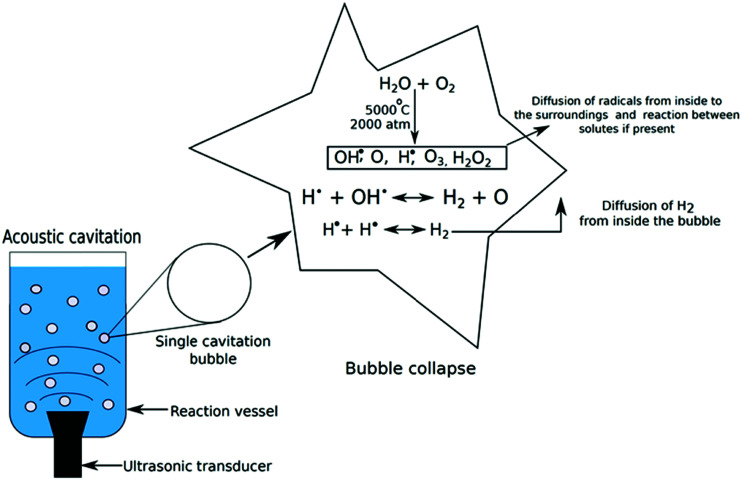
Production of sonolysis species by acoustic cavitation.^1846^

Acoustic cavitation of an ultrasonicated liquid can be defined as the activation of pre-existing nuclei to form stable or transient bubbles in the liquid. These cavitation bubbles usually contain gas molecules such as N_2_, O_2_ and other gases as well as vapour from the liquid. When these bubbles grow in size, they become unstable and then violently collapse creating localised transient high temperatures and pressures at STP. The collapsing of these acoustic bubbles on a solid surface also leads to the formation of microjets being directed towards the surface of the solid material at speeds of up to 200 m s^−1^. It is well-accepted in the field that the cavitation bubble collapse leads to near adiabatic heating of the vapour that is inside the bubble, creating the so-called “hotspot” in the liquid, where: (1) high temperatures (*ca.* 5000 K) and high pressures (*ca.* 2000 atms) are generated with a collision density of 1.5 kg cm^−2^ and pressure gradients of 2 TPa cm^−1^, with lifetimes shorter than 0.1 μs and cooling rates above 10^9–10^ K s^−1^ during the collapsing of cavitation bubbles. At the high temperature and pressure generated by bubble collapse, the liquid vapour and gas molecules generate various highly reactive radicals and other species^1845^ (Fig. 147).^1846^

In the case of ultrasonicated water, water vapour is ‘pyrolysed’ into these ‘microreactors’ and dissociates to lead to the formation of extremely reactive species such as hydroxyl radicals (˙OH), hydrogen radicals (H˙), and hydroperoxyl radicals (˙OOH) as well as hydrogen peroxide (H_2_O_2_) – a process known as *water sonolysis*.H˙ + H˙ → H_2_ (*x*)H˙ + ˙OOH → O_2_ + H_2_ (*x*)H˙ + H_2_O → ˙OH + H_2_ (*x*)H˙ + H_2_O_2_ → H_2_ + HO_2_˙ (*x*)During water sonolysis, molecular hydrogen is produced and the sonolyic species diffuse out from the interior of the bubble into the surroundings and react with solutes present in the aqueous solution.^1843–1845^

To this day, there are a few reports focusing solely on the application of ultrasound for the production of hydrogen. For example, Sasikala *et al.*^1846^ showed that hydrogen produced by water sonolysis can be improved by adding suspended metal oxide microparticles (γ-Al_2_O_3_, TiO_2_ and SiO_2_) during ultrasonication, due to the increased number of cavitation bubbles caused by the presence of these particles. They also demonstrated that hydrogen production rates significantly increased by adding methanol to water during ultrasonication, as it was found that the alcohol was efficiently scavenging ˙OH radicals and thus thwarting ˙OH and H˙ recombination.

However, since 2015, it has been an upsurge of interest in the area, for example, Merouani and Hamdaoui^1847^ by using modelling tools, reported in great detail the mechanisms of the sonochemical production of hydrogen. In 2019, Islam *et al.*^1821^ reviewed the area followed by Dincer *et al.*^1822,1823^ who investigated the challenges and opportunities of the use of ultrasound in hydrogen production.

#### 12.3.2 Sonoelectrochemistry in water splitting

There are only a few reports in the literature dealing with the effects of PUS on the HER and OER. For example, in 1992, Cataldo^1824^ studied the effects of ultrasound (30 kHz) on the HER and ClER (chlorine evolution reaction) on Pt and carbon electrodes immersed in NaCl (6.0 M), HCl (6.0 M) and acidified NaCl (5.0 M NaCl/1.1 M HCl). He found that effective removal of hydrogen and chlorine gas bubbles at the electrode surface leads to better gas yields. Walton *et al.*^1825^ showed that PUS (38 kHz) slightly affected the HER, OER and ClER at a platinised Pt electrode immersed in 1.0 M H_2_SO_4_ and 2.5 M NaCl/0.1 M HCl due to efficient removal of adhering product species on the electrode surface. McMurray *et al.*^1826^ showed PUS (20 kHz) affected the HER and OER on a titanium sonotrode when the vibrating ultrasonic horn was acting as the working electrode immersed in a neutral aqueous 0.7 M Na_2_SO_4_/0.1 M NaOH electrolyte; he concluded that these observations were mainly due to enhanced mass transport and increased metallic corrosion rates induced by intense agitation and cavitation at the electrode surface.

Moriguchi^1841^ and Pollet *et al.*^1827^ showed that ultrasound decreases the electrode overpotential for the OER and HER on Ag, Pt and SS (stainless steel) electrodes immersed in aqueous solutions. Pollet *et al.*^1827^ also showed that the onset potentials for hydrogen and oxygen were both reduced with increasing ultrasonic power; no appreciable change in the Tafel slopes were observed, although the exchange current density (*j*_o_) values were different in the absence and presence of ultrasound. They postulated that this decrease in overpotentials could be due to either changes in electrode surface, changes in electrode surface temperature, degassing at the electrode surface or a combination of all.

Budischak *et al.*^1828^ also studied the effects of ultrasound on HER in 2.0 M KOH using Pt as a working electrode and found that ultrasound can greatly improve water electrolysis efficiency, especially at intermediate current densities. Li *et al.*^1829^ demonstrated that the HER was affected by ultrasound in a *pseudo*-water electrolyser comprising of two dimensionally stable anodes (DSA, RuO_2_ and IrO_2_ plated Ti electrodes) used as working and counter electrodes immersed in weak alkaline solutions (0.1 M, 0.5 M and 1.0 M NaOH). PUS aided in removing the thin layer of bubbles at the electrode surface, especially at lower concentrations, thus yielding energy saving for hydrogen production of up to 25%. In their conditions, no evident effects of ultrasound on the OER were observed. Li *et al.*^1830^ investigated the effects of ultrasound (25.3 kHz and 33.3 kHz) on a pure graphite electrode immersed in 0.40 M NaOH electrolytes; the cell voltage was much lower under ultrasonic conditions at the two frequencies employed than under *silent* conditions (cell voltage reductions at a current density of 200 mA cm^−2^ for 0.1 M, 0.5 M and 1.0 M NaOH was +320 mV, +100 mV and +75 mV respectively.

Pollet and co-researchers^1831–1834^ found that ultrasound could practically remove H_2_ and O_2_ gas bubbles efficiently from the electrode surfaces and electrolyte in turns improving electrochemical hydrogen and oxygen production rates. They investigated the effects of ultrasound (20 kHz) on hydrogen production from acidic and base electrolytes on several electrode materials used both as anodes and cathodes (316 stainless steel, carbon graphite, POCO carbon, Morganite carbon, nickel, and titanium);^1831–1834^ PUS increased the hydrogen and oxygen production rates due to the efficient electrode cleaning, electrode surface/solution degassing and enhanced mass-transfer of electroactive species to the electrode surface. Zadeh^1833,1834^ used ultrasound (20 and 40 kHz) to generate hydrogen from carbon and nickel alloy electrodes immersed in NaOH and KOH electrolytes (up to 15 M): the sonoelectrochemical hydrogen production is enhanced by 14% and 25% for NaOH and KOH respectively, the electrolyte conductivity playing an important role in the hydrogen yield.

Lin and Hourng^1835^ demonstrated by EIS that PUS (133 kHz @ transmitted powers of 225, 450, 675, and 900 W) enhanced the activity and concentration impedances and greatly improved the removal of hydrogen bubbles at Ni electrode surfaces immersed in a series of concentrations of 10, 20, 30 and 40 wt% KOH electrolytes: (i) at 30 wt% KOH and at low potentials, PUS improved the activation polarisation, and (ii) concentration polarisations were improved under ultrasonic conditions due to efficient degasification at the electrode surface. Under optimum conditions (+4 V, 40 wt% KOH, 2 mm electrode gap, 225 W), the difference in current density was found to be 240 mA cm^−2^ yielding a power saving of 3.25 kW and a gain in power efficiency of up to 15%.

**Fig. 148:**
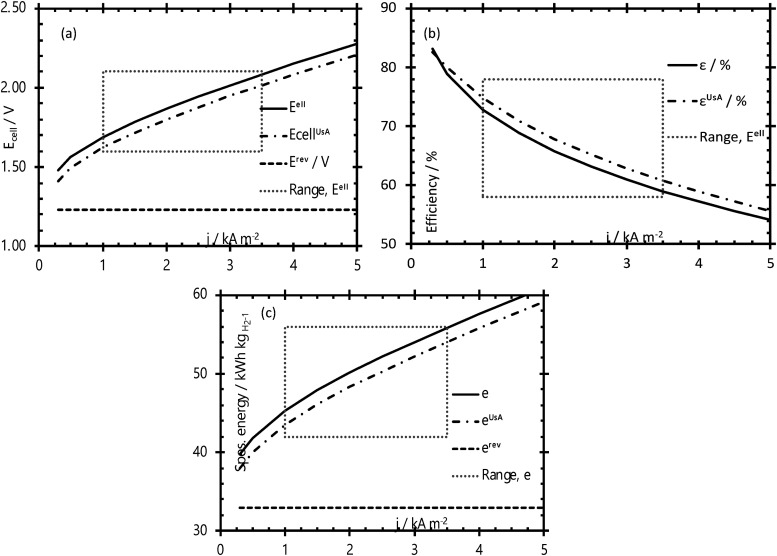
Effect of ultrasound on (a) cell voltage (*E*_cell_), (b) efficiency (*ε*) and (c) specific energy (e) for hydrogen production (*UsA = ultrasound-assisted).^1841^

In 2019, Islam *et al.*^1841^ reviewed the area showed that PUS can be a used as a powerful tool to overcome the limitations of electrochemical water splitting technologies for hydrogen production *via*: (i) electrode surface cleaning and activation, (ii) increased mass-transfer in the bulk electrolyte and near the electrode surface, and (iii) efficient degassing at the electrode surface and electrolyte. They also showed that ultrasound can improve the electrolytic efficiency (up to 15–20%) caused by increased ion concentration and bubble removal at the electrode surface (Fig. 148).

**Fig. 149:**
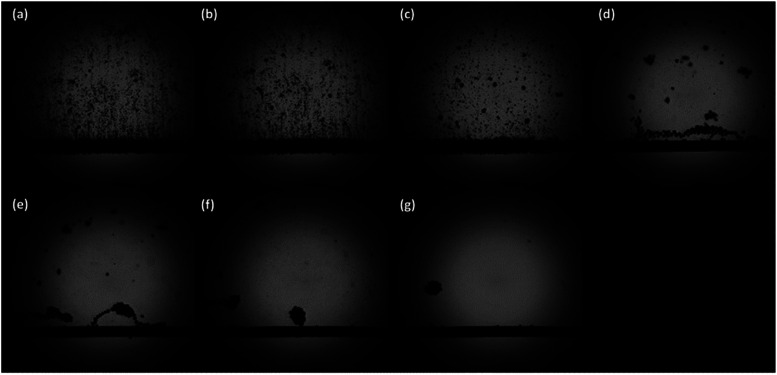
Hydrogen evolution on a Pt wire in the absence (top left corner) and presence of ultrasound (26 kHz, 100% ultrasonic amplitude). The applied potential was set at −1.30 V *vs.* RHE – (a) 0 μs, (b) 100 μs, (c) 200 μs, (d) 300 μs, (e) 400 μs, (f) 500 μs, (g) 600 μs. The time between each image is 10^−4^ s (100 μs) filmed at 10 000 frames per second. Reproduced with permission from ref. 1836. Copyright Elsevier 2020.

Very recently, it was observed by Pollet *et al.*^1836^ that ultrasound (26 kHz, up to ∼75 W cm^−2^, up to 100% acoustic amplitude, ultrasonic horn) significantly affects the HER currents with an ∼250% increase in current density achieved at maximum ultrasonic power on a Pt polycrystalline electrode immersed in a weak acidic electrolyte (0.5 M H_2_SO_4_; Fig. 149). At *j* = −10 mA cm^−2^, a Δ*E*_HER_ shift of ∼+20 mV was observed, at 26 kHz and at 100% acoustic amplitude. At the same ultrasonic frequency and acoustic power, a nearly 100% increase in the exchange current density and a 30% decrease in the Tafel slope was observed in the low overpotential region, although in the high overpotential region, the Tafel slopes were not significantly affected when compared to *silent* conditions. Overall, ultrasound did not dramatically change the HER mechanism but instead, increased currents at the Pt surface area through effective hydrogen bubble removal).^1836^

Overall, the effects of ultrasound on the HER and OER processes are due possibly due to the following combination of effects: (i) depolarisation mainly due to highly efficient electrolyte stirring, in turns reducing and even eliminating the contribution of concentration gradients to the overpotential, (ii) effective electrode surface activation caused by acoustic cavitation, and (iii) gas bubble removal from the bulk electrolyte and the electrode surface due to efficient degasification induced by intense agitation, acoustic cavitation and acoustic streaming. However, literature indicates that no studies have been undertaken to shed some light on whether power ultrasound affects the HER and OER mechanisms. Table 15 shows a summary of the experimental conditions employed for the sonoelectrochemical production of hydrogen.

**Table 15:** Summary of sonoelectrochemical hydrogen production. Modified from ref. 1841

Ultrasonic frequency (kHz)	Ultrasonic power or intensity	Reactions	Electrode material	Electrolyte and concentration	Cell voltage (V)	Current density	Ref.
30	1–2 W cm^−2^	HER	Carbon rod	6.0 M NaCl, 6.0 M HCl, 5.0 M NaCl + 1.1 M HCl	8, 10, 12, 20	2.7, 6.5, 7.6 A dm^−2^	1824
		ClER					
38	—	HER	Platinised platinum	1.0 M H_2_SO_4_, 2.5 M NaCl/0.1 M HCl	—	50 mA cm^−2^	1825
		OER					
		ClER					
20	26 W cm^−2^	HER	Titanium alloy sonotrode	0.7 M Na_2_SO_4_ (maintained pH at 7 by using 0.1 M NaOH)	—	—	1826
		OER					
20	43 W cm^−2^	HER	Ag, stainless steel, carbon, platinum	Na_2_S_2_O_3_/NaHSO_3_	—	—	1827
500		OER					
42	300 W	HER	Platinum	2.0 M KOH	—	—	1828
60	50 W cm^−2^	OER	DSA–RuO_2_ and IrO_2_ plated on titanium	0.1, 0.5 M, 1.0 M NaOH	—	20–400 mA cm^−2^	1829
25.3	—	OER	Pure graphite	0.40 M NaOH	—	20–200 mA cm^−2^	1830
33.3							
20	139.72, 1186.6, 2349.8 W	HER	Carbon graphite, POCO carbon, platinum	NaOH (0.1, 0.2, 0.3, 0.4, 0.5, 1.0 M)	—	<200 mA cm^−2^	1831
33		OER		NaCl (0.1, 0.2, 0.3, 0.4, 0.5, 1.0 M)			
		ClER		H_2_SO_4_ (0.1, 0.2, 0.3, 0.4, 0.5, 1.0 M)			
20	20.7 W cm^−2^	HER	Carbon (Morganite)	NaOH (0.1, 0.2, 0.4, 0.5, 1.0 M)	<3	<200 mA cm^−2^	1832
40		OER		NaCl (0.1, 0.2, 0.4, 0.5, 1.0 M)			
		ClER		H_2_SO_4_ (0.1, 0.2, 0.4, 0.5, 1.0 M)			
20	—	HER	Carbon, nickel alloy (Rolls-Royce)	0.1 M NaOH	<3	<200 mA cm^−2^	1833
40		OER		0.1 M, 1.0 M, 10 M, 15 M KOH			
20	—	HER	Nickel	0.1 M NaOH	<3	<200 mA cm^−2^	1834
		OER		0.1 M KOH			
133	225, 450, 675, 900 W	HER	Pure nickel	10, 20, 30, 40 wt% KOH	<4	<2 A cm^−2^	1835
		OER					
26	75 W cm^−2^	HER	Pt	0.5 M H_2_SO_4_	—	—	1836

As a conclusion to this section, it must be mentioned that, although many different physic-assisted water electrolysis concepts have been successfully demonstrated, the net gain in efficiency has not be precisely quantified, *i.e.*, the cost of generation of the physical signal has not been optimised (and in some case evaluated) *versus* the gain in electrochemical output. In essence, doing so is not easy, especially at the laboratory scale, and only well-dimensioned setups (production plants) will enable really assessing where the game is worth to be played. So, there is a wealth of technological and industrial studies that need to be achieved prior these physics-assisted water electrolysis processes become an industrial reality.

## 13 Water splitting from seawater, wastewater and other non-pure sources

The electrochemical splitting of pure water requires substantial electrical input energy, since the resistance of pure water is 18 MΩ cm. In contrast, the resistance of tap water and seawater are up to six orders of magnitude lower (*R*_Seawater_ = 20 Ω cm) which, viewed from the perspective of conductivity, in principle allows energy efficient splitting of water. Sea water covers nearly 70% of the earth's surface and presents the most abundant aqueous feedstock on earth (∼97% of the total water^1848^). In areas where fresh water is scarce, the direct use of seawater is advantageous to avoid the costs of water treatment. However, seawater is highly corrosive and contains Cl^−^ ^1849^ (3.5% average global salinity) and microorganisms^1850^ that can impact metal corrosion. Especially the chloride anions (∼0.5 M in seawater) poses serious challenges for the OER electrode that is set to oxidative hence positive potentials. Parasitic electrochemical reactions can occur on the anode and may lead to side products like chlorine or binary Cl–O compounds. Perhaps these reasons are basically responsible that the number of reports dedicated to electrocatalysis of seawater remains till to date within a manageable range.^503,1851–1861,1864,1868^ Oxygen evolution and chlorine evolution will in general always compete with each other, and since chlorine is a valuable intermediate in industry, it depends on perspective to decide whether OER or CER is the undesirable parasitic reaction.^1862,1863^ It is therefore understandable that electrocatalysts that are able to selectively support or suppress one or the other reaction are of great interest.^1862^

The authors dare to say that, whenever hydrogen is intended to be produced electrochemically, the OER will be (compared to CER) the preferred other water-splitting half-cell reaction because transportation of chlorine is difficult and the projected hydrogen demand is enormous and hard to bring in line with the local chlorine demand.

**Fig. 150:**
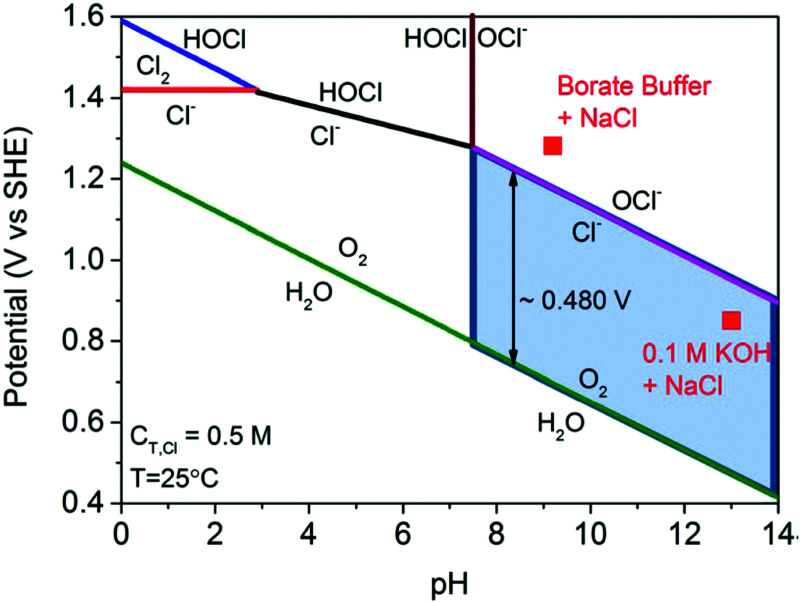
Pourbaix diagram for electrolysis of 0.5 M NaCl. The electrode potential for OER is included as well, assuming oxygen partial pressure of 0.021 MPa. The red square points show the operating potentials (*vs.* SHE) after 1 h constant current density of 10 mA cm^−2^ with NiFe LDH catalyst in 0.1 M KOH + 0.5 M NaCl (pH 13) and 0.3 M borate buffer + 0.5 M NaCl (pH 9.2) electrolyte. Reproduced with permission from ref. 1864 Copyright Wiley 2016.

The water oxidation reaction obeys the Nernst equation and consequently shows strong pH dependence. Unlike OER, the equilibrium potential of the chorine evolution reaction (CER):

20 2Cl^−^ → Cl_2_ + 2e^−^

does not depend on pH with the consequence that, under acidic conditions, the OER equilibrium potential *vs.* the normal hydrogen electrode (NHE) is only 130 mV^1864^ lower than that of chlorine evolution at pH 0 and 298 K. Therefore, in acidic solutions, the CER can in principle occur and can compete with the OER which is nevertheless thermodynamically favoured over CER as can be taken from the Pourbaix diagram^1865^ (Fig. 150).

In contrast to the four-electron oxidation reaction OER, CER is a two-electron reaction with only a single intermediate. Due to the faster kinetics, the parasitic CER can become the dominant anodic reaction in acidic electrolytes on several metal oxide-based electrocatalysts.^1862,1866,1867^ A more substantial broader gap between onset of OER and CER obtained at pH 0 can be expected at somewhat higher pH (up to pH 3). At even higher pH values, a second parasitic, electron-consuming reaction must be considered, namely the hypochlorite formation reaction (HFR):

21 Cl^−^ + 2OH^−^ → ClO^−^ + H_2_O + 2e^−^ *E*^0^ = +0.89 V_NHE,pH 14_

In contrast to CER, the equilibrium potential of the HFR slows down with increasing pH and the potential difference to OER is fixed 480 mV. Even when taking into consideration the faster kinetics of the HFR relative to the OER (HFR represents a two-electron transfer reaction), the “safety distance” of almost 500 mV will be sufficient to suppress HFR in the case of not so large overvoltage values for the OER.

All of these considerations inevitably show that water splitting of chloride ion containing media is more advantageous in alkaline media than in the neutral or acidic regime. To the best of the authors knowledge, Bennett *et al.*^503^ was the first to report on the direct electrolysis of seawater. Current densities of 155 mA cm^−2^ in conventional seawater electrolysers equipped with standard electrodes, *i.e.*, TiO_2_/RuO_2_-based DSA, PbO_2_, and graphite electrodes, exhibited a faradaic efficiency for chlorine evolution up to 92% upon exploitation of neutral, unbuffered seawater. OER and CER taking place on the anode led to a substantial drop of the pH of the electrolyte in the immediate vicinity of the electrode based on the equations,

22 2H_2_O → O_2_ + 4H^+^ + 4e^−^

23 Cl_2_ + H_2_O → HClO + Cl^−^ + H^+^

which minimises the difference between equilibrium potentials for OER and CER (the thermodynamic voltage of OER becomes more anodic) and therefore increases the compatibility of CER. Furthermore, high practical current densities lead to high OER overpotentials, which disadvantages the OER (at practical current density) compared with the CER even more than under equilibrium conditions. Upon adding Mn^2+^ solution and acidification with HCl, chlorine gas formation stopped after a while and a MnO_2_ coating was formed on the TiO_2_/RuO_2_ DSA anode. An electrode prepared this way was found to efficiently produce oxygen from seawater (*η* = 720 mV; *j* = 1000 mA cm^−2^) with faradaic efficiency (FE) exceeding 99%. Obviously, this unusual performance was either caused by an increment of the exchange current density for the OER or by a decrease of the exchange current density for the CER.

A Japanese group took advantage of this material and modified MnO_2_ (deposited on IrO_2_-coated titanium substrate) for water electrolysis of seawater, showing high selectivity towards oxygen evolution by doping with molybdenum or tungsten,^1851–1853^ or by simultaneous addition of both transition metals.^1854^ This group reported later on more temperature-stable (up to 90 °C) triple oxide-based anodes.^1855^

Thin films of Nocera's Co–Pi system were also found to be suitable electrocatalysts for selective water oxidation in Pi electrolyte in the presence of 0.5 M NaCl at neutral pH.^1868^ The buffer solution used by the authors suppresses an acidification of the electrolyte. However, the current density (around 1 mA cm^−2^) was too low to be of practical importance and most likely chlorine formation was simply not obtained due to the weak oxidative potential applied to the anode (1.30 V *vs.* NHE).

**Fig. 151:**
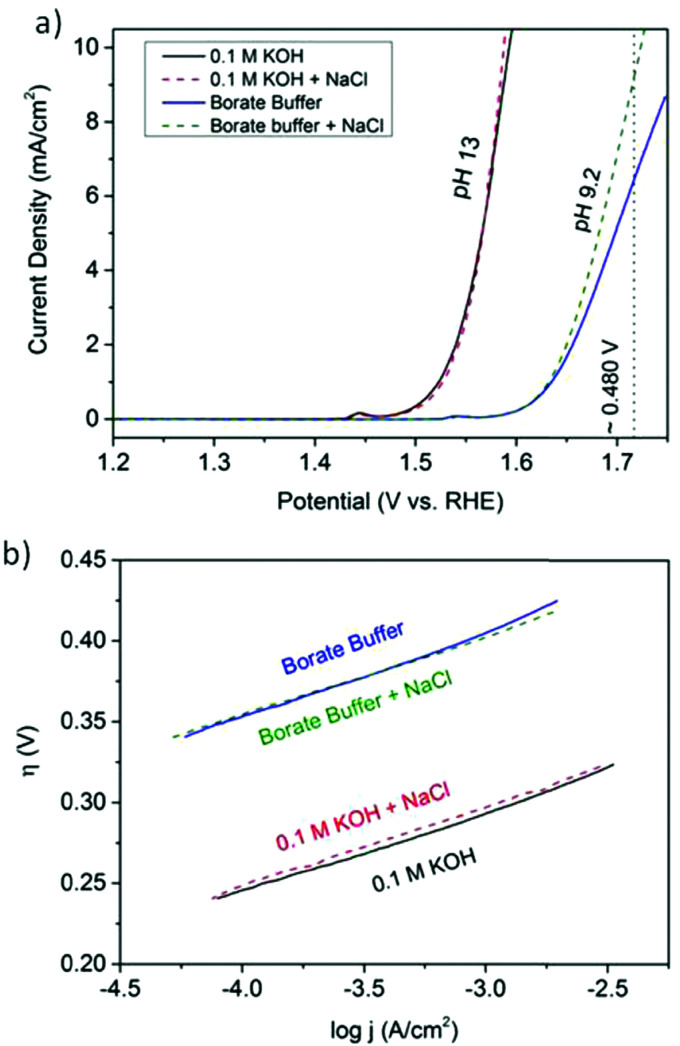
(a) Electrocatalytic OER activities of NiFe LDH nanoplates supported on carbon, measured using LSV in four different electrolytes after CV “break-in” (50 cycles). A potential of approximately 480 mV, corresponding to the design criteria limit, is marked by a dashed vertical line. (b) Corresponding Tafel plot for low current density *j*. Measurement conditions: room temperature, 1600 rpm, and scan rate of 1 mV s^−1^. Reproduced with permission from ref. 1864. Copyright Wiley 2016.

Taking into consideration both thermodynamics and kinetics, Dionigi *et al.* defined design criteria for reasonable seawater splitting and chose 480 mV as the upper limit for the OER overpotential (at *j* = 10 mA cm^−2^) and 7.5 as the lower pH value of the electrolyte based on the fact that, below pH 7.5, the gap between *E*_0_ (HFR) and *E*_0_ (OER) becomes smaller than 480 mV (Fig. 150).^1864^ The authors synthesised NiFe-layered double hydroxide (NiFe LDH) by a solvothermal method.^1864^ Glassy carbon (GC) with 0.1 mg cm^−2^ NiFe-LDH loading used as an OER electrode in borate buffer (pH 9.2) and 0.1 M KOH (pH 13), with or without additional NaCl (0.5 M) exhibited 100% oxygen/hydrogen selectivity. Chloride ions did not adversely affect the OER activity of the NiFe LDH catalysts at current densities up to 10 mA cm^−2^ and, in case of pH 9.2, chloride ions even boost the OER activity (Fig. 151).

**Fig. 152:**
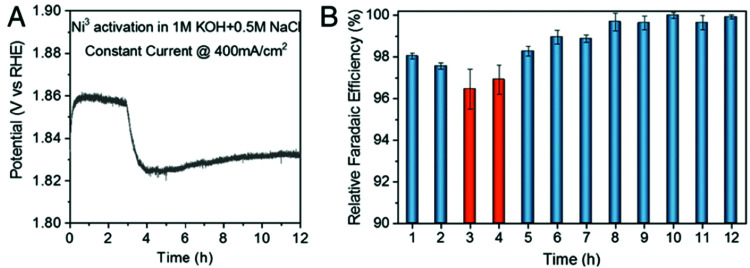
Cation-selective layer generation during anodic activation (A) chronopotentiometry plot whilst second activation step in salty electrolyte. (B) The associated OER relative faradaic efficiency plots for O_2_ production. Reproduced with permission from ref. 1857 Copyright PNAS 2019.

Industrially required current densities (0.4 < *j* < 1 A cm^−2^) that can be realised in long-term experiments without substantial degradation of the catalytically active compounds are for most of the common electrode materials still very challenging. Kuang *et al.*^1857^ recently reported a multilayer (hierarchical) anode consisting of NiFe hydroxide coated on a nickel sulphide (NiS_*x*_) layer formed on porous Ni foam (NiFe/NiS_*x*_–Ni). They stated that, during anodic activation of NiFe/NiS_*x*_–Ni successively in 1 M KOH and in 1 M KOH/0.5 M NaCl, sulphate ions and carbonate ions are formed and intercalated in the NiFe-layered double hydroxide which increased the OER activity (Fig. 152).

Obviously polyanion-rich passivating layers are *in situ*-generated in the anode and lead to a repelling of chloride anions and thus suppress parasitic reactions with chlorine containing reactants.

**Fig. 153:**
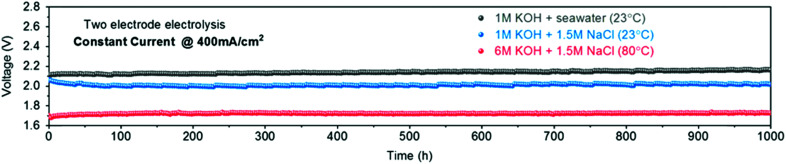
Durability tests (1000 h) recorded at a constant current of 400 mA cm^−2^ of the seawater-splitting electrolyser under 1 M KOH + real seawater at room temperature and 6 M KOH electrolyte at 80 °C, respectively. (h) Reproduced with permission from ref. 1857. Copyright PNAS 2019.

Full water splitting upon exploitation of an anode designed in this way and a Ni–NiO–Cr_2_O_3_ hydrogen evolution reaction cathode was shown at a cell voltage of 1.7 V as delivering *j* = 400 mA cm^−2^ current density in 6 M KOH/1.5 M NaCl at 80 °C^1857^ (Fig. 153).

In a more recent work, commercial Ni foam was converted *via* a one-step surface modification route into a porous, S-doped Ni/Fe (oxy)hydroxide electrocatalyst capable for water oxidation performed in 1 : 1 mixtures of 1 M NaOH and 1 M NaCl at pH 14 reaching a current density of 100 mA cm^−2^ at around 300 mV overpotential.^1859^

**Fig. 154:**
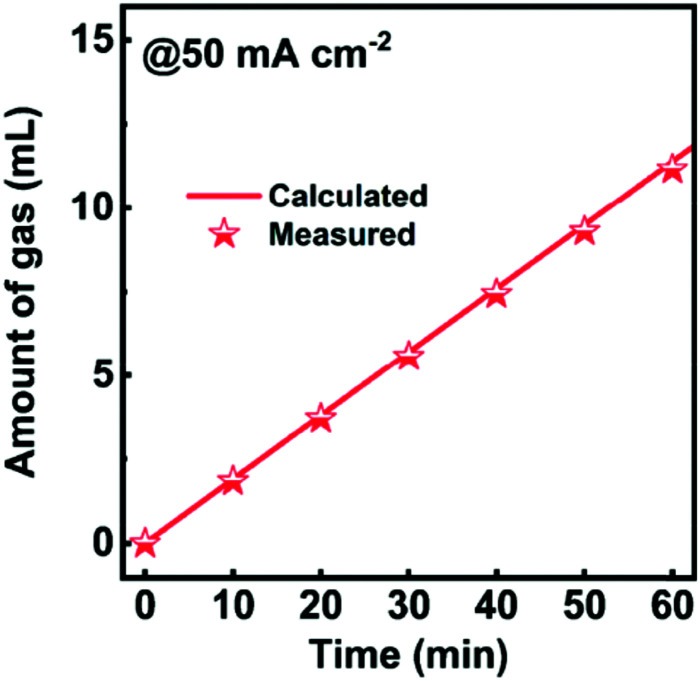
Theoretically calculated and experimentally measured O_2_ amounts for NCFPO/C@CC as a function of time in the NaCl + KOH electrolyte. Reproduced with permission from ref. 1858. Copyright ACS 2020.

The approaches that scientists developed for splitting salty electrolytes are not solely restricted to metal-based substrates. Song *et al.*^1858^ recently developed carbon-coated sodium cobalt-iron pyrophosphate (Na_2_Co_1−*x*_Fe_*x*_P_2_O_7_/C; 0 ≤ *x* ≤ 1) nanoparticles loaded on carbon cloth (NCFPO/C@CC) as a promising OER electrocatalyst for alkaline seawater electrolysis. The catalyst exhibited competitive current density to overpotential relationship (*η* = 270 mV at *j* = 10 mA cm^−2^) in 0.1 M KOH/0.5 M NaCl solution mixtures as well as long term durability. Even at *j* = 50 mA cm^−2^, this material showed an OER FE of close to 100% (Fig. 154).

**Fig. 155:**
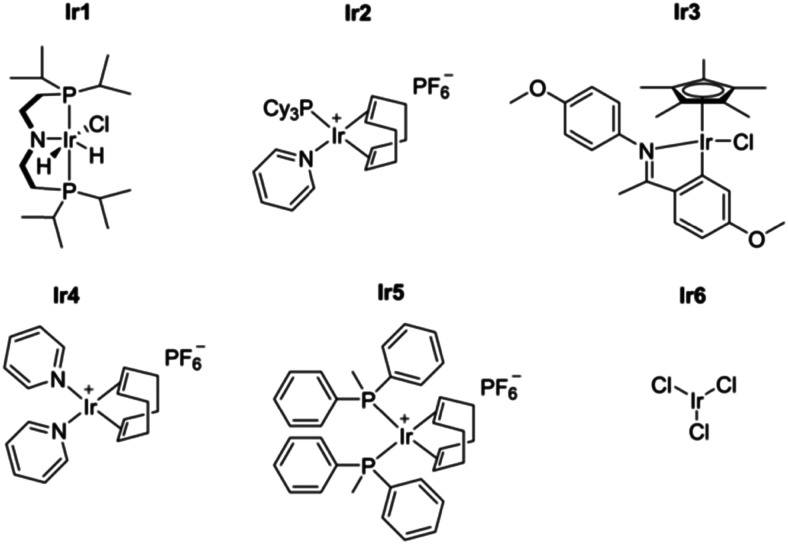
Survey of Ir-based organometallics subject to pyrolysis with activated carbon. Ir1, Ir2, Ir3, Ir4, Ir5, and Ir6 correspond to the following organometallics, respectively: chlorodihydrido[bis(2-diisopropylphosphino)ethylamine]iridium(iii), (1,5-yclooctadiene)(pyridine)(tricyclohexylphosphine)-iridium(i) hexafluorophosphate,chloro(5-methoxy-2-{1-[(4-methoxyphenyl)imino-*N*]ethyl}phenyl-C)(1,2,3,4,5 pentamethylcyclopentadienyl)iridium(iii), bis(pyridine)(1,5-cyclooctadiene) iridium(i)hexafluorophosphate, (1,5-cyclooctadiene)bis(methyldiphenylphosphine)iridium(i) hexafluorophosphate, and iridium chloride. Reproduced with permission from ref. 1860 Copyright Wiley 2020.

As already mentioned, it is particularly difficult to selectively form O_2_ gas in the acidic range at the anode in the presence of chloride ions. This is certainly the reason why studies reporting saltwater electrolysis at low pH levels can rarely be found. Ko *et al.*^1860^ chose a not very widely used method for the generation of OER electrodes. A series of catalysts have been produced by pyrolysing Ir organometallics in the presence of a Norit® activated carbon as conductive substrate. Tailored heteroatom doping is possible through specific choice of the Ir organometallic compound (Fig. 155). Due to low Ir doping (2–6 wt%), the overall costs can be kept within limits. A respectably low overpotential (*η* = 283 mV) was required for *j* = 10 mA cm^−2^ OER-based current density in 0.1 M HClO_4_ + 5 wt% NaCl.

Also molecular-based approaches have been taken into consideration: Karunadasa *et al.* checked their molybdenum-oxo catalyst also for suitability to support hydrogen evolution when dissolved in natural salt water and obtained onset of hydrogen evolution at about −0.81 V *vs.* RHE.^1679^ On the one hand, this underlines the feasibility in principle, but it also shows the long way to go in order to achieve the practical applicability of homogeneous water catalysis.

Several groups are investigating microbial electrolysis of wastewater for purification^1869^ or hydrogen gas production purposes.^1870,1871^ Bio-catalysed electrolysis (microbial electrolysis) for hydrogen production was independently discovered by two research groups.^1872,1873^ Bacteria can be exploited to generate hydrogen gas upon an electrolysis with electrode reactions similar to the ones occurring in a microbial fuel cell (MFC). The working principle of an MFC is based on oxidation of organic compounds by bacteria under formation of CO_2_, protons plus electrons.^1874^ Molecular oxygen present at the cathode will undergo an ORR, resulting in a potential difference between anode and cathode which in turn can lead to the flow of electricity. If the flow of current is forced by applying voltage between anode and cathode, hydrogen gas is produced at the cathode though reduction of protons.

Usually, when using electrochemical approaches for the treatment of salt-containing wastewater, chlorine is generated as the active waste-degrading compound^1875,1876^ (while also being a pollutant).^1877–1880^ A non-microbial electrolysis-based approach for purification of organic-polluted wastewaters with high salt loads (mostly NaCl) without chlorine formation has been recently demonstrated,^1881^ in which real diaminodiphenylmethane-production wastewater (10 wt% NaCl) was electrochemically purified upon using a boron-doped diamond anode and an oxygen-depolarised cathode (ODC). The anodically produced oxidants, which are either hydroxyl radical or ozone, are obviously responsible for the effective degradation of waste materials.

A very recently published report^1882^ deals with an analysis of seawater electrolysis technologies for the production of green hydrogen based on economic, ecological, and social criteria upon using a multicriteria decision-making (MCDM) approach. Five different MCDM techniques have been used in this study to ensure a consistent ranking (Analytic Hierarchy Process (AHP), Choosing By Advantages (CBA), Simple Additive Weighting (SAW), Complex Proportional Assessment (COPRAS), and Technique for Order of Preference by Similarity to Ideal Solution (TOPSIS). These different MCDM approaches have been applied to a set of different electrolyser technologies.

Direct electrolysis of seawater (DES) was compared with alkaline water electrolysis (AWE), proton exchange membrane (PEM) water electrolysis, and solid oxide electrolysis (SOE) which are used after the demineralisation of seawater. The best economic approach will produce hydrogen at lowest levelised costs, which requires an estimation of investment costs, operation and maintenance costs (O&M), nominal lifetime, costs based on impurities in feed water, and costs caused by power changes. The criteria related to the environmental factor must focus on aspects that could affect the environment in some way and criteria belonging to the social factor assess the risk of harm that could arise for workers and are specific to each technology. With regard to almost all criteria, direct electrolysis of salty water is outperformed by a combination of up-to-date de-ionisation technology plus alkaline water electrolysis (AWE) and proton exchange membrane (PEM) water electrolysis, respectively.

Only in terms of resilience can DES be considered on par with PEM. All the MCDM methods agree on the ranking, with the best option being PEM followed by AWE. As such, there is good reason that, even if salt water is ubiquitous, it is not used as an electrolyte for water electrolysis purposes. Thus, solely demineralised water is used as an electrolyte on board nuclear submarines where water electrolysis technologies are frequently found as life support systems for oxygen production.^1883^ The authors therefore think that further investing resources in exploration of the direct electrolysis of seawater is at least worthy of discussion.

## 14 Markets and costs for hydrogen electrolysis

Hydrogen is undergoing a renaissance. Major financial institutes are positioning themselves to advise on hydrogen^1884–1886^ in anticipation of a growing commercial market. The European Union's 2020 hydrogen strategy signalled a step-change in commitment to the technology, establishing a target for 40 GW of electrolysers installed over the coming decade.^259^ The industry has responded with manufacturing scale-up and the advent of “gigafactories”^1887,1888^ – mirroring the GW-scale production plants for lithium-ion batteries.

For these plans to materialise and embed hydrogen as a mainstream part of the global energy system, it is critical that hydrogen achieves cost competitiveness against incumbent technologies. The two most important drivers of hydrogen cost are the capital cost (capex) of the electrolyser and the input fuel cost of electricity (opex). Both costs vary widely across regions, between technologies and over time.

This section reviews the markets for hydrogen and anticipated scale-up of the industry. The focus is on current capital costs of electrolysis devices and the influence of components and manufacturing stages. Projected developments in capital costs over time and surveys the drivers for potential cost reduction are reviewed. Finally, the levelised cost of hydrogen production is presented, which factors in all capital and operating costs.

### 14.1 Commercial status of hydrogen electrolysis

**Fig. 156:**
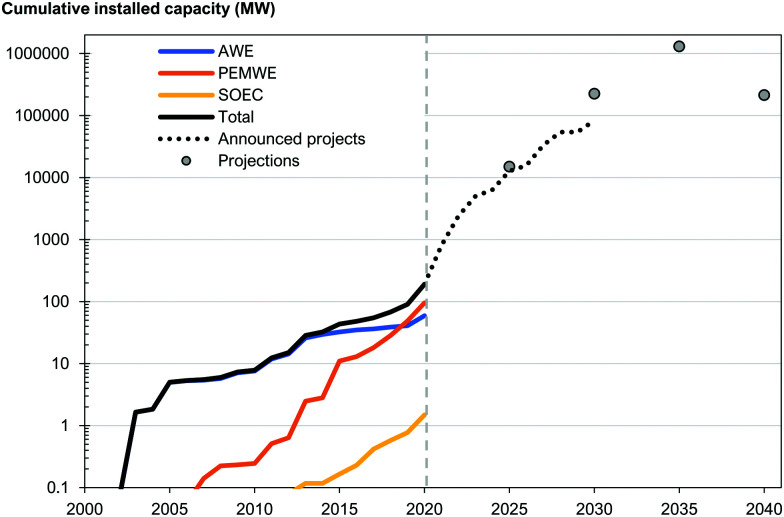
The cumulative installed capacity of modern hydrogen electrolysers, split by technology; with analysts’ projections for future market size. Historical data from Buttler and IEA,^18,1891^ and future trajectories from Aurora and the ETC.^1890,1892^

Electrolysis only provides around 1 to 2% of global hydrogen production, or around 7 Mt per year.^1889^ This share is set to increase though; Fig. 156 shows the global installed hydrogen electrolyser capacity over time, and near-term projections from various sources. Global capacity has grown rapidly over the last decade, by an average of 32% per year since 2010. AWE was the most mature technology, forming over 90% of global capacity as recently as 2010. However, growth since then has only been 19% per year, whereas PEMWE capacity has grown at 80% per year, overtaking the installed capacity of AWE in 2019. Aurora identifies over 200 GW of new electrolysis projects planned for delivery by 2040,^1890^ of which 85% is located within Europe. This suggests that the market will accelerate over the coming decade with 75% annual growth.

#### 14.1.1 Markets for hydrogen

Widespread optimism about the prospects for hydrogen is not a new phenomenon.^229,1893,1894^ Hydrogen technologies have been a faithful adherent to the Gartner-Hype Cycle model,^1895^ experiencing cycles of excessive expectations followed by disillusion and bankruptcies.^229,1896^

The potential markets for hydrogen are changing, as competition from other low-carbon technologies intensifies. In previous decades, passenger vehicles^36^ and home-heating systems^1897^ were thought of as the leading sectors to be served by hydrogen. Their prospects are now seen as waning, as battery electric vehicles^1898^ and electric heat pumps^1899^ have gained early ground in the transition away from fossil fuels.

**Fig. 157:**
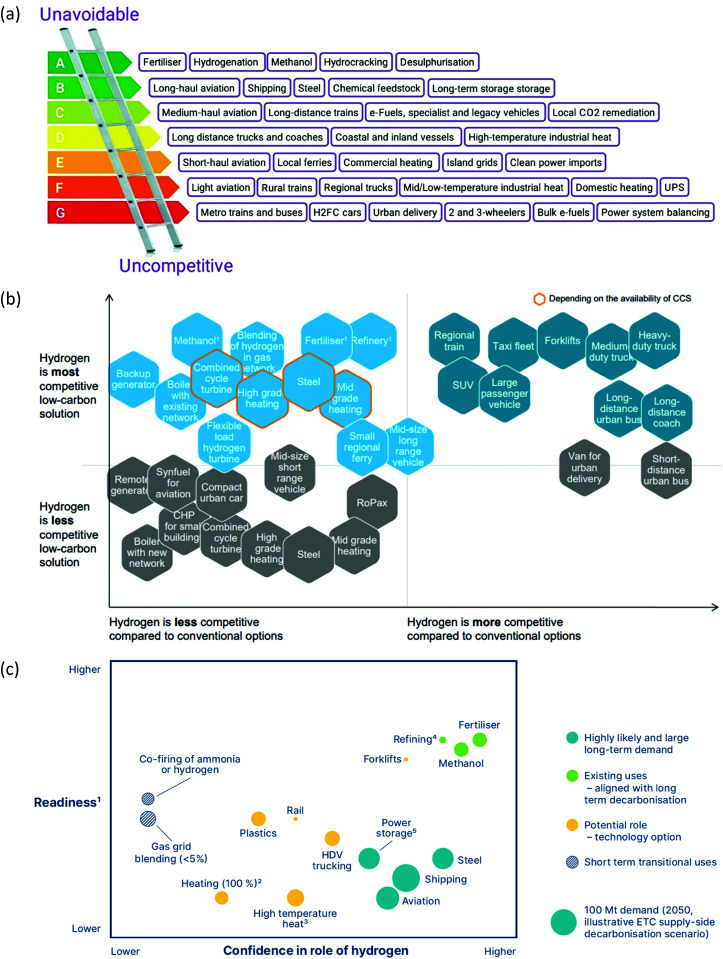
The perceived competitiveness of hydrogen across different market sectors. (a) The ‘hydrogen ladder’ popularised by Liebreich Associates,^1900^ which ranks applications from uncompetitive to unavoidable. (b) The competitiveness of hydrogen applications *versus* low-carbon and conventional alternatives, from the Hydrogen Council.^1901^ (c) The assessment of multiple potential uses of hydrogen performed by SYSTEMIQ for the ETC.^1892^

Fig. 157 shows two examples of analysts’ expectations for where hydrogen will be competitive. The role of hydrogen is less contested for decarbonising specific industrial sectors (*e.g.*, fertiliser and refining), heavy duty transport (shipping, aviation, trucks and buses) and especially for long-duration electricity storage.

#### 14.1.2 Major manufacturers of electrolysers

The global electrolyser market is relatively concentrated. Buttler & Spliethoff surveyed the market in 2018, finding only 33 medium to large suppliers in total: 20 AWE, 12 PEMWE, and 1 SOEC suppliers.^18^ This situation may change as the market is dynamic with acquisitions being common (for example Hydrogenics being purchased by Cummins and Air Liquide).^1902^

IRENA^1903^ and the ETC^1892^ and various market research firms discuss the main technology manufacturers. Some prominent examples are listed by technology in Table 16.

**Table 16:** A non-exhaustive selection of major manufacturers of electrolysers

AWE	PEMWE	SOEC	AEMWE
Asahi Kesei (Japan)	Cummins (US)*	Ceres (UK)	Enapter (Italy)
John Cockerill (France/Belgium)	Elogen (Germany)	Haldor Tøpsoe (Denmark)	
McPhy (France)	ITM Power (UK)	Sunfire (Germany)	
Teledyne (US)	NEL (Norway)*	Toshiba (Japan)	
Thyssenkrupp (Germany)	Siemens (Germany)		
Tianjin Mainland (China)			
Yangzhou Chungdean (China)			

### 14.2 Current capital cost of electrolysers

As with many areas in the energy sector, capex plays a defining role in the overall economic viability of hydrogen electrolysis. The cost of electrolytes will be critically important to their success, and competitiveness against other routes to producing hydrogen and other low-carbon fuels. The cost of electrolysers is relatively difficult to quantify for four reasons:

(1) The technology is still at an early stage of commercial development (so data are not readily available);

(2) Costs differ substantially by technology due to design and materials requirements, as well as the maturity and scale of production;

(3) Prices vary strongly based on country of manufacture, with a prominent disparity between China and the rest of the world;

(4) Prices are changing rapidly as manufacturers increase their scale of production.

#### 14.2.1 Survey of current electrolyser costs

**Fig. 158:**
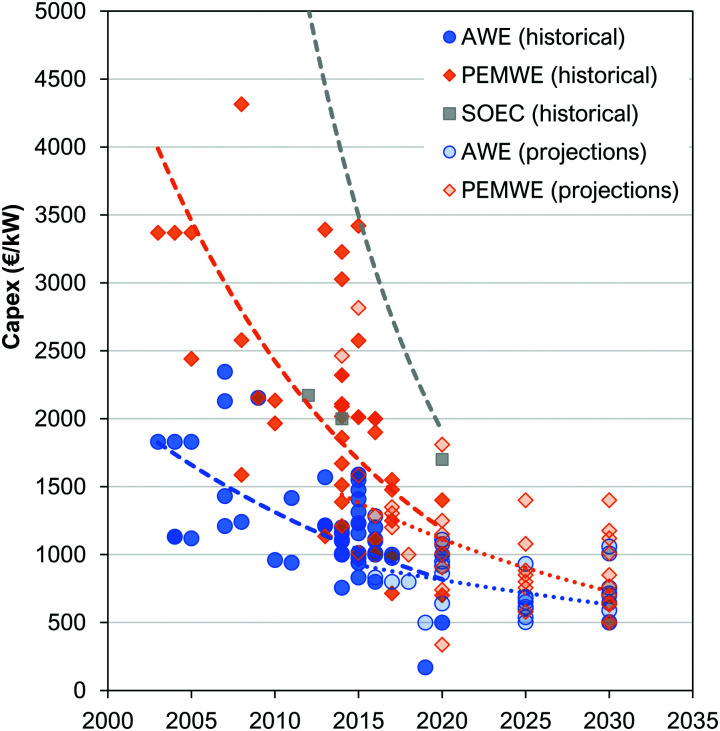
Capex costs of electrolysers, both historical and projections for alkaline, PEM and SOEC technologies. Data compiled from ref. 1903–1906.

Current estimates of electrolyser costs vary by an order of magnitude from €170 to 2.300 per kW of capacity (Fig. 158). Values are differentiated by technology type, with estimates for AWE at €170–1000 kW^−1^, PEMWE at €700–2000 kW^−1^, and SOEC at ∼€2000 kW^−1^. The minimum cost for alkaline electrolysers of €170 kW^−1^ ($200 kW^−1^) is noteworthy, a value cited in several organisations relating to claims of cost from recent Chinese manufacturing plants (see Section 14.3 and 0).

It is evident from Fig. 158 that costs have been rapidly falling in recent years. BNEF estimate that the capex of large-scale electrolysers fell by 40–50% in the five years to 2019.^1907^ Specifically, AWEs fell from $2000 to $1200 kW^−1^ over the period, while PEMWEs fell from $2800 to $1400 kW^−1^.

#### 14.2.2 Influence of materials and components

AWE and PEMWE electrolysers are relatively mature technologies, with several products commercially available at known prices. SOEC only surpassed 1 MW of capacity installed in 2019, so greater variation and uncertainty surrounds their costs. For the more novel technologies considered in this paper (AEMWEs, PCCELs), costs can only be speculated upon as large 100 + kW systems have not yet been built.

**Fig. 159:**
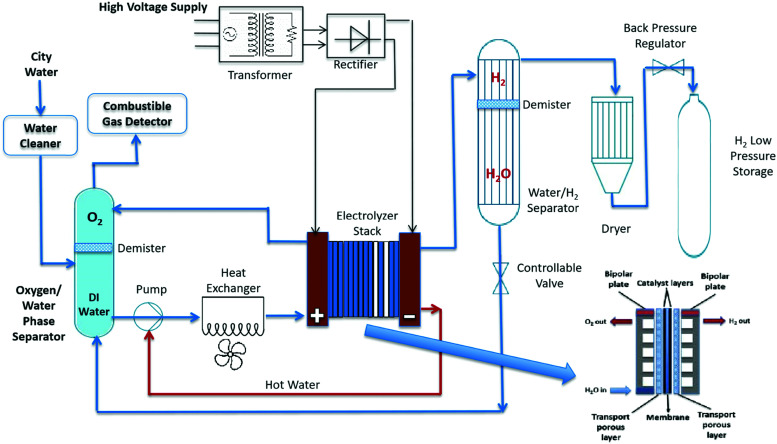
Typcial schematic of a PEMWE system. Source ref. 1908:

Electrolysis systems consist of more than just the electrolyser stack (Fig. 159). Ancillary equipment, known as the balance-of-plant (BoP) include the power conditioning (transformer and rectifier to condition the DC supply), water treatment (purification and heating), and hydrogen conditioning (separation, drying and pressurisation). All these components are mature technologies and used in a wide array of other industries and settings.

**Fig. 160:**
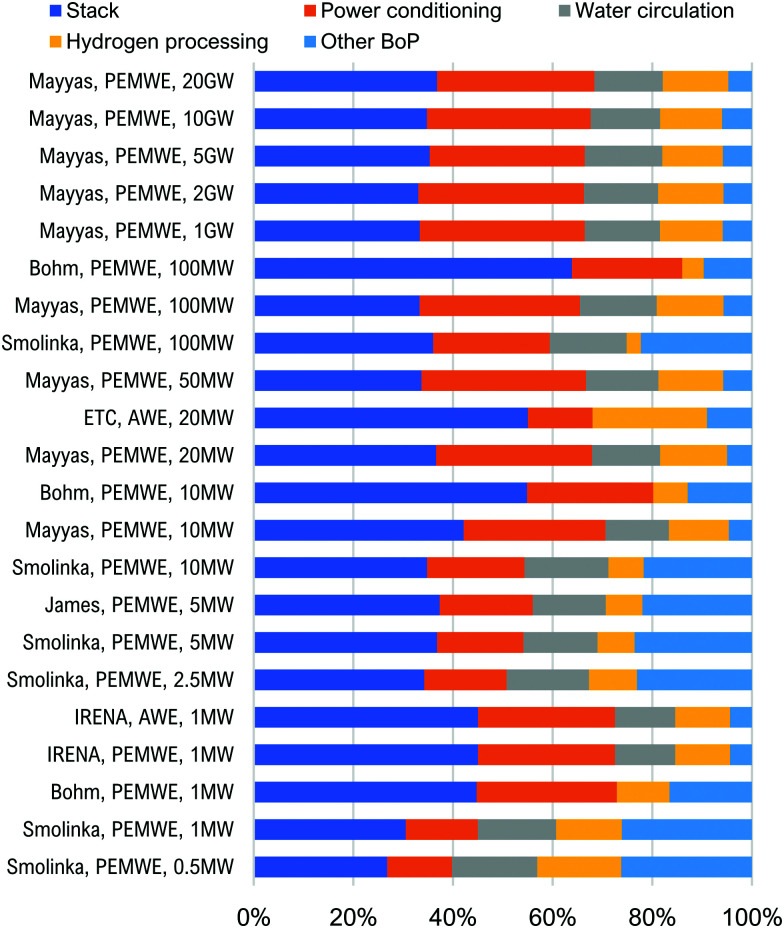
Comparison of the cost contribution of different electrolyser components. Data from ref. 1892, 1903, 1904 and 1909–1911.

The cost contribution of the electrolyser stack itself varies widely across literature, from 27% to 64%. Fig. 160 shows a range of study estimates of the contribution to capex from different electrolyser components.

For example, IRENA calculates the stack contributes 45% of total system cost.^1903^ The remainder comes from the balance-of-plant components: power supply (28%), water circulation (12%), hydrogen processing (11%) and cooling (4%).^1903^ Mayyas and Mann similarly model the stack as contributing 40% of the total system cost,^1909^ with the BOP share mostly coming from the power supply. The share from balance-of-plant grows with scale of production, from 60% at 10 MW per year to 70% at 1 GW per year due to declining stack production costs.^1909^ IRENA^1903^ and ETC^1892^ also present breakdowns of AWE cost, giving 45% and 55% share respectively to the electrolyser stack. The majority of this cost is from manufacturing the diaphragm/electrode package, and the breakdown of BOP costs is similar to that for PEMWE.

**Fig. 161:**
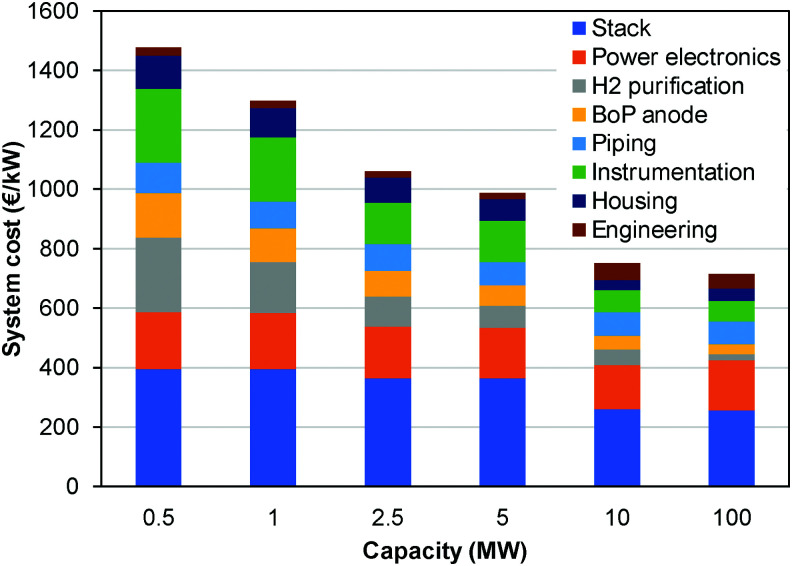
Component contribution to PEMWE electrolysis system cost at different capacities. Data from ref. 1904.

Broadly as the capacity or production levels increase, the contribution from the stack increases. Lower cost estimates are associated with larger capacity installations: Fig. 161 shows a breakdown of system costs for different capacities. Whilst there are some cost reductions associated with the stack cost, their largely modular design lends less favourably to economies of scale. However, substantial cost reductions are achieved with the balance-of-plant, including hydrogen and water conditioning.

There are very few publicly-available inventories for electrolysis stacks to understand the contributing components of the costs and it is likely that there is a large variation across manufacturers and scales of production. NREL suggest that AWE stacks cost 100 USD kW^−1^ (1 MW capacity, producing 10 to 20 units per year).

**Fig. 162:**
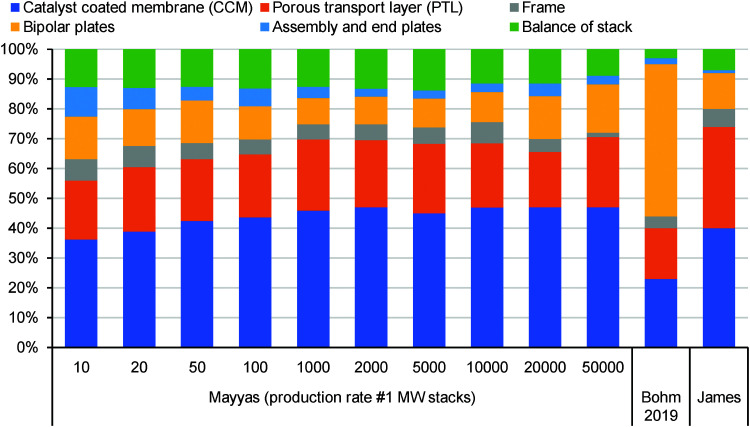
Estimates of cost contribution of different PEM electrolyser stack elements from three studies. Data from ref. 1909–1911.

There are differences in the literature on the cost contribution from different stack elements see Fig. 162 for PEMWE. The catalyst-coated membrane is typically the largest cost (23 to 47% of total) due to use of iridium and platinum, whereas bipolar plates represent a high cost (9 to 51%) depending on the material used: higher costs associated with titanium plates, whereas lower costs may be from gold-coated steel manufacture.^1908^

### 14.3 Future capital cost of electrolysers

Another complication in assessing the economics of hydrogen electrolysis is that costs are rapidly changing over time. New hydrogen production technologies are being developed and established technologies are undergoing continual refinement. Combined with the rapid scale-up of manufacturing, there is widespread expectation that current prices will continue to fall. This has been observed widely across the energy sector, with prominent examples being solar PV panels,^1912^ offshore wind farms,^1913^ electricity storage systems^1914^ and hydrogen fuels cells.^1915^

#### 14.3.1 Experience curve analysis

Experience curves are an empirical approach used to track the development of a product's price as a function of its cumulative installed capacity. For each doubling of installed capacity, historical prices are often observed to fall by a fixed percentage – known as the experience rate (ER). Product price has been observed to relate to the experience by:7.1
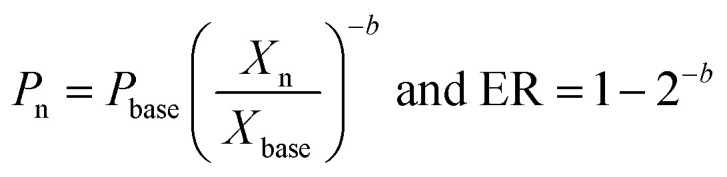
where *P*_n_ is the price of a specific unit, *P*_base_ is the price of a reference unit, *X*_n_ is the experience, *X*_base_ is the cumulative experience gained before the construction of the product, and *b* is an exponent. Experience can be represented by number of units, or more commonly by the production capacity (*e.g.*, MW of electrolyser).

Experience curves are well established within the energy sector for modelling future product prices,^1916,1917^ and can be traced back to Wright's Law^1918^ from the 1930s. Solar photovoltaic panels are a prime example, with module prices falling by 23% for each doubling of capacity between 1976 and 2019.^1919^ Experience rates for energy technologies typically lie in the region of 5 to 30%.^1914,1915,1920^

Neij argues that modular technologies such as electrolysers should experience higher learning rates than monolithic products such as turbines.^1921^ Malhotra and Schmidt^1922^ show empirically that simple and standardised products such as solar panels or LED lights have higher learning rates (18–22%) than complex or customised/bespoke technologies such as conventional power plants or building insulation (3–5%). With electrolyser stacks being modular assemblies of standard repeated units, electrolysis would appear to fit the ‘simple and standardised’ group of technologies, which ought to experience the highest of these learning rates.

IRENA^1903^ and Saba *et al.*^1923^ survey previous studies of learning rates for electrolysers (Table 17). As there are relatively few studies to date, these learning rates are compared to estimates for hydrogen fuel cell systems, which “can be adapted also to electrolysers”.^1923^ Various studies have suggested that fuel cells have comparable learning rate in the region of 15 to 21%.^1915,1924–1927^

**Table 17:** Estimates for the learning rate for hydrogen electrolysers

Technology	Notes	Learning rate (%)	Ref.
AWE	Hypothetical, 1977–1994	10	Thomas^1928^
AWE	Observed, 1972–2004	18 ± 13	Schoots^1929^
AWE	Observed, 1956–2014	18 ± 6	Schmidt^1914^
AWE	Projection for 2020–30	9	Hydrogen Council^1901^
PEMWE	Projection for 2020–30	13	Hydrogen Council^1901^

**Fig. 163:**
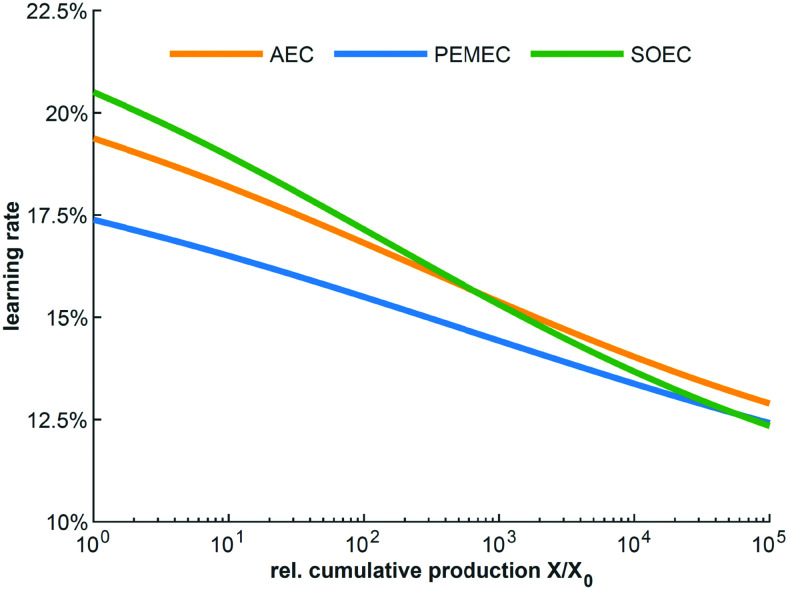
The development of experience rates for electrolysis stack modules as a function of cumulative production. Reproduced from Böhm *et al.*^1910^

Böhm *et al.*^1910^ anticipate that the experience rate for electrolysers will decline over time as cumulative production increases (Fig. 163). This would occur because the core components of the electrolyser (catalyst layers, bipolar plates) are expected to have the higher learning rates than the generic components (flanges and pumps) and, as these core components become cheaper, their impact on the overall system's rate of cost decline will weaken.

These estimated learning rates can be combined with a forecast for the future market size (in terms of GW of capacity installed) to create future cost projections. Schmidt *et al.*^1914^ provides an example of this, projecting the price of alkaline electrolysers up to a cumulative capacity of 100 GW. When combined with a market projection, which is conservative in today's terms, this gives prices of $1300 kW^−1^ in 2030 and $970 kW^−1^ in 2040.

**Fig. 164:**
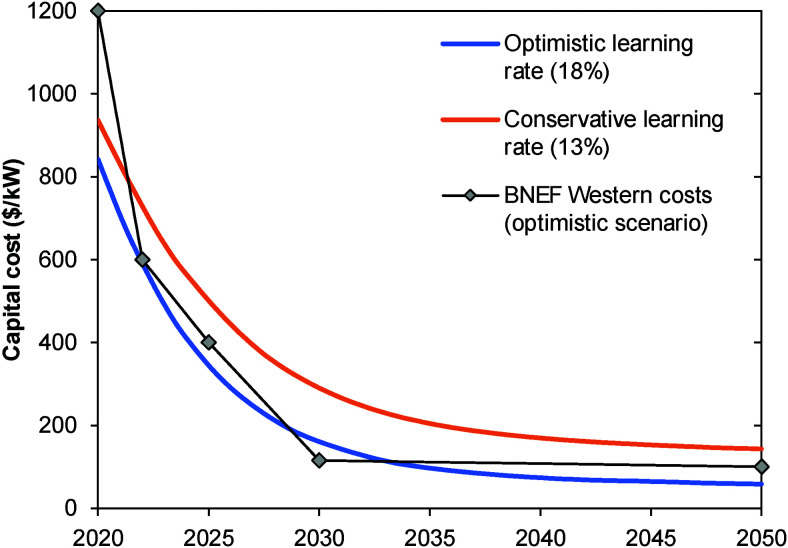
Cost projections from ETC based on optimistic and conservative learning rates for electrolysers (technology-neutral); compared to the BNEF scenario for costs outside of China. Data from ref. 1892.

ETC^1892^ provides another example yielding much lower costs: attaining $160 kW^−1^ in 2030 and $80 kW^−1^ in 2040 in their ‘optimistic scenario’ (Fig. 164). This prediction uses an 18% learning rate, the same as in Schmidt *et al.*, but yields much lower prices due to a lower reference price for electrolysis ($825 kW^−1^ in 2020 compared to $1340 kW^−1^ in ref. 1914) and more optimistic scenario for market growth (3300 GW installed by 2040 *versus* 270 GW in ref. 1914). This comparison highlights the sensitivity of experience curve analyses to their specific assumptions.

#### 14.3.2 Expert elicitation analysis

**Fig. 165:**
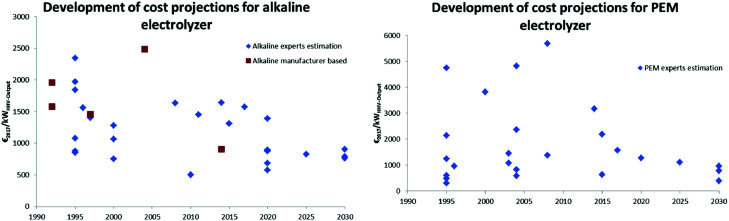
Cost projections for alkaline and PEM electrolysers surveyed from the literature. Reproduced from Saba *et al.*^1923^

Due to the scarcity of empirical data, studies have compiled expert estimates of future costs. Saba *et al.*^1923^ compile a list of estimates for AWE and PEMWE electrolyser costs spanning back to the 1990s (Fig. 165). For both technologies they see cost estimates falling and converging to below $1000 kW^−1^ after 2020.

Bertuccioli *et al.*^1906^ provided trajectories for AWE and PEMWE costs out to 2030, using expert elicitation with 22 people from industry and academia. The expert estimates for AWE systems cost fell from $1100 kW^−1^ ($900–1300 range) in 2015 to $700 ($450–950 range) in 2030. For PEMWE, the estimates were $1.900 kW^−1^ ($1.450–2.350 range) in 2015 falling to $900 ($300–1.500 range) in 2030.

**Fig. 166:**
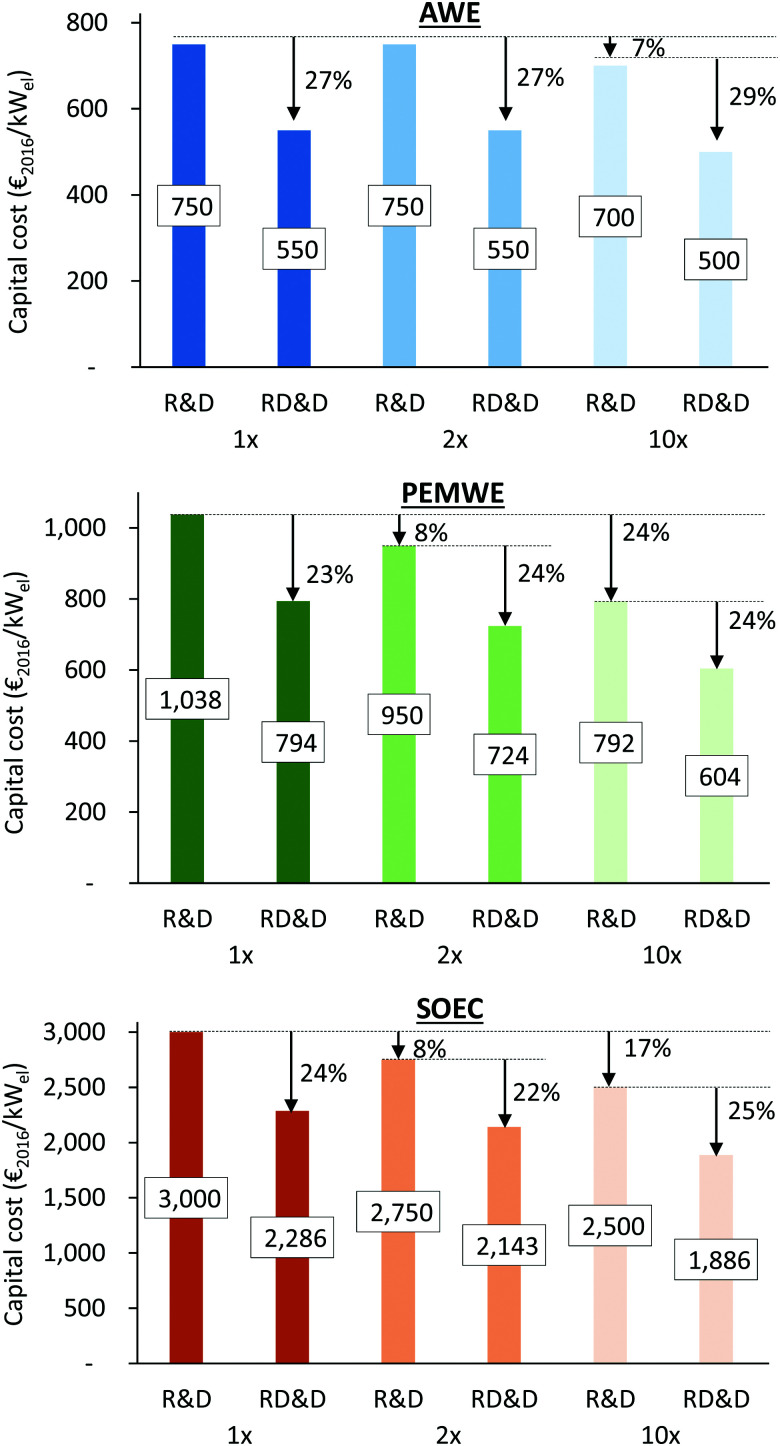
Estimated capital costs for water electrolysis in 2030 from expert elicitations conducted by Schmidt *et al.*^1930^ The median cost from all experts is given by technology (top to bottom). Each panel shows the relative impact of increased R&D funding (1x, 2x, 10x) by bars labelled R&D. This impact combined with production scale-up due to increased deployment is shown by bars labelled RD&D. Reproduced from ref. 1930.

Similarly, Schmidt *et al.*^1930^ conducted an expert elicitation with ten people from industry and academia to gauge opinion on future cost reductions with both increased R&D funding and production scale-up (Fig. 166). These elicitations yielded similar ranges to those from Bertuccioli *et al.* albeit with narrower ranges in 2030. The experts estimated that increased R&D funding for water electrolysis could lower capital costs by 7–24% by 2030, with the weakest effect seen for AWE due to its maturity. Production scale-up was consistently thought to reduce costs by a further 22–29% across all technologies (Fig. 166).

### 14.4 Drivers of cost reduction

Cost reductions are likely to be driven by a quickly maturing and growing market, namely: manufacturing scale-up, plant size increases, design improvements, and shifting production to cheaper world regions.

#### 14.4.1 Electrolyser plant size

**Fig. 167:**
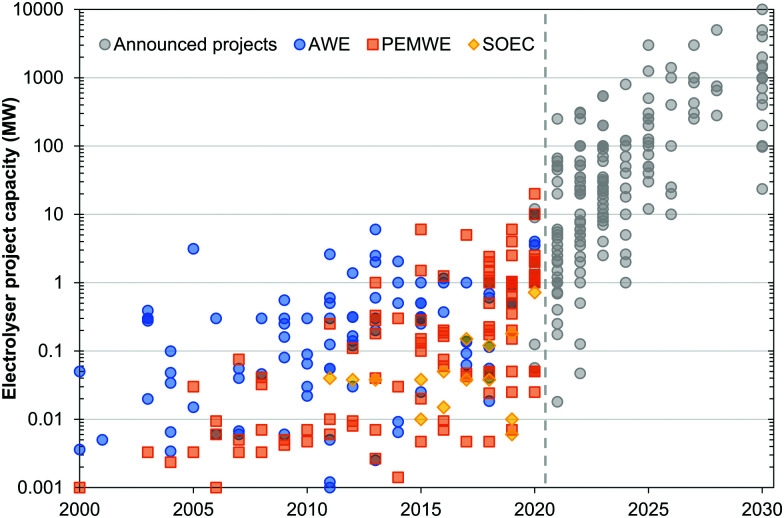
The size of individual electrolysis plants commissioned over the last two decades, and announced by companies for construction during the next decade. Compiled using data from IEA^1891^ and Aurora.^1890^

Whilst electrolysers spent several decades at the kW scale, the size of individual electrolyser projects has increased markedly over the last decade as manufacturing supply-chains mature. Between 2010 and 2017, AWE systems increased in size from 120 kW to 2 MW on average, and PEMWE increased from 10 kW to 2.9 MW.^1891^ Projects are expected to increase by three orders of magnitude over the coming decade, with rapid scale-up from 1–5 MW in 2020 to 30–300 MW by 2025^1890,1891^ (Fig. 167).

**Fig. 168:**
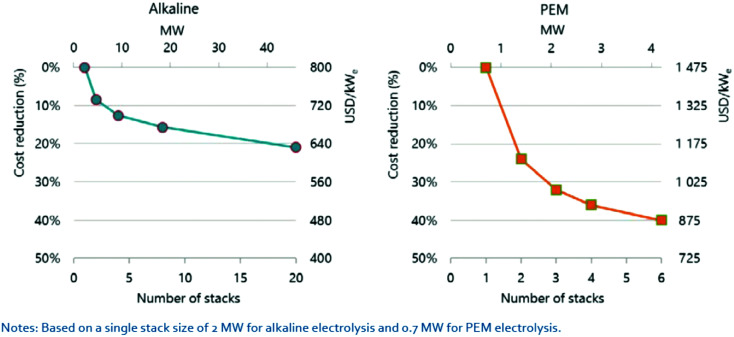
Estimate of cost reduction associated with plant size increases for AWEs and PEMWEs. Reproduced from the IEA.^1889^

The impact of increasing plant size reduces system cost *via* economies of scale. As the capacity of the system increases the material and energy requirement typically reduces per unit of production (Fig. 168).

#### 14.4.2 Manufacturing scale-up

Manufacturing scale-up also gives substantial potential for cost reduction. As with increasing plant size, increasing economies of scale in manufacturing can significantly reduce specific costs such as energy and material requirements and labour *via* increased automation and increased learning rates. For electrolysers, a move away from manual stacking and connecting, towards high volume manufacturing methods such as LASER-cutting, plastic injection moulding and 3D-printing could contribute to cost reductions.

Increased learning from manufacturing experience will help to de-risk system design and utilise finer margins (*e.g.*, lower material requirements) to optimise cost, efficiency and lifetimes. Costs of capital and building were identified by Mayyas as being large contributors to low-volume-production of stack elements such as the catalyst-coated membrane, bipolar plates and porous transport layer (for PEMWE) and could be all but eliminated at large manufacturing volumes (of over 2000 units per year).^1909^

#### 14.4.3 Design improvements

There are several technical improvements that may increase efficiency or reduce cost for electrolyser stacks and are specific to each electrolyser technology type. For AWEs, increasing current density from 0.2–0.4 up to 0.6 A cm^−2^*via* better mixed metal oxide catalysts may be achievable.^1931^ A higher temperature operation would enable increased efficiency with more stable electrodes and electrolytes, and zero-gap designs which remove the distance between electrodes would decrease the resistance associated with electrolyte and bubble formation.^1931,1932^

For PEMWEs, higher current densities can be achieved, from 0.6–2 up to >3 A cm^−2^*via* improved electrode design, catalyst coating and thinner membranes. Reducing the use of iridium and platinum with thinner coatings may reduce cost, as well as a replacement of titanium in bipolar plates and porous transport layers with a high-conductivity/stable coatings on low-cost materials such as steel. The rectifier, which converts AC current to DC, represents a large proportion of capex which could be reduced if a DC supply was used and required only a DC/DC converter.

For SOEC, capex reductions are achievable *via* reducing operating temperatures to ∼450 °C from reducing electrode polarisation resistance. This would help to avoid the requirement for high-temperature exotic materials and enable the use of lower cost materials such as stainless steel. So far, SOECs are still at an early stage of development and there is a need to prove lifetimes and improve cell and stack designs.

**Fig. 169:**
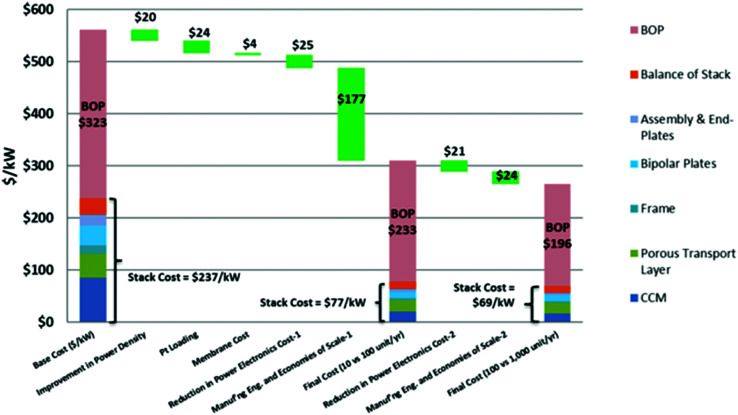
Future cost reductions for PEMWE systems across different production scales. Reproduced from Mayyas and Mann.^1909^

To illustrate the combined potential cost reductions associated with design improvements, increased plant size and manufacturing scale up, Fig. 169 shows an example cost reduction for a PEMWE system. The largest improvements are made from manufacturing economies of scale increasing production from 10 to 100 units per year, but total costs may be reduced from ∼$560 to 270 kW^−1^.

#### 14.4.4 Shifting production centres

Another source of anticipated cost reductions is the shift of production from the west (primarily Europe and America) to China. This mirrors the experience seen with other low-carbon technologies; for example, the price of solar PV panels fell rapidly when production shifted from Germany and the US to China.^1933^

BNEF cite three reasons for lower costs in China: lower costs for raw materials and labour, higher utilisation rates for factories, and lower spending on R&D and marketing.^1934^ Others suggest that production quality is a factor, in particular lower durability and reliability.^1935^ BNEF announced that Chinese-made AWEs sold for $200 kW^−1^ in 2019, 83% less than Western-made systems at the time.^1934^ In addition, the lessons from COVID 19 could also spark a re-industrialising of Europe.

This was more bullish than other sources, as according to the IEA AWEs cost $500 kW^−1^. BNEF assumes that costs from Western manufacturers could converge with those from Chinese manufacturers over the coming decade.^1934^ Failing to become more competitive on cost could result in a declining market share for these manufacturers, and ultimately bankruptcy. Agora propose that EU-wide innovation support is key to the success of electrolysis manufacturing in Europe.^1935^

### 14.5 Levelised cost of hydrogen production

While capital costs are important, they are only one component of the overall lifetime cost. The total cost of construction and operation – and thus the cost of hydrogen produced – also depends primarily on the cost of electricity purchased, and on technical parameters such as the cell efficiency and lifetime.

Just as renewable and conventional power stations can be summarised by their levelised cost of energy (LCOE), the total cost of electrolysis can be summarised by the levelised cost of hydrogen (LCOH), also known as the levelised cost of gas (LCOG). This quantifies the total cost of production discounted over the system's lifetime, per unit of hydrogen generated (*e.g.*, $ kg^−1^ or $ MW^−1^)

The LCOH provides a fair comparison by factoring in all technical and economic parameters: capital cost, operating costs, production efficiency, system lifetime, performance degradation and the cost of energy used. This concept can be used to explore important trade-offs, for example the use of better materials to increase the durability or efficiency of the system. This will likely increase the capital cost but reduce operating costs due to less maintenance required or less electricity needing to be purchased.

#### 14.5.1 Calculation of LCOH

The levelised cost of hydrogen can be described as the total lifetime cost of the investment in a hydrogen production technology divided by its cumulative delivered hydrogen. Its value reveals the average price that hydrogen must be sold for to make the system break-even financially.^19^ Both costs and hydrogen production are discounted according to the investment's cost of capital (also known as the discount rate), to reflect the time-value of money. Costs incurred many years into the future, or the value of hydrogen that is sold far into the future will have less importance to the viability of the investment decision made today.

As with the levelised cost of storage (LCOS), there are various definitions employed which may include or exclude relevant parameters such as end-of-life disposal of the system, electrolyser stack replacement or capacity degradation over the lifetime.^1936^

The levelised cost of hydrogen^1936^ is given by:7.2
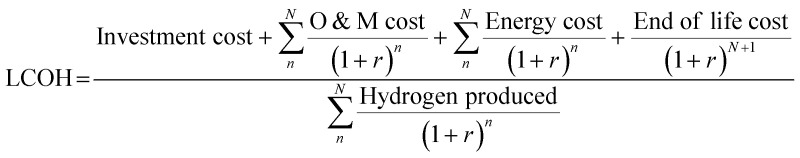
summing up all cost categories in each year (*n*) up to the system's lifetime (*N*), and discounting each by the project's discount rate (*r*).

#### 14.5.2 The importance of electricity costs

The total cost of hydrogen production from electricity chiefly comprises the electrolyser capex and the cost of electricity used as input to the electrolyser. The IEA notes that with increasing utilisation, capex has a decreasing impact on hydrogen costs, whereas electricity purchase becomes the main cost component for water electrolysis.^1889^

The latter is governed by the producing technology and the regional environment. A key distinction is whether electricity is purchased from a region's power grid or directly from a low-carbon or renewable generation source. Wholesale power market prices vary around the world due to differences in generation mix and the fuels used, emissions prices and taxation; but a primary driver in most markets is the global or regional price of fossil fuels.^1937,1938^ Electricity prices also see substantial short-term and long-term volatility, varying diurnally with demand and availability of renewable energy, and seasonally with fluctuating fossil-fuel prices.^1939,1940^

**Fig. 170:**
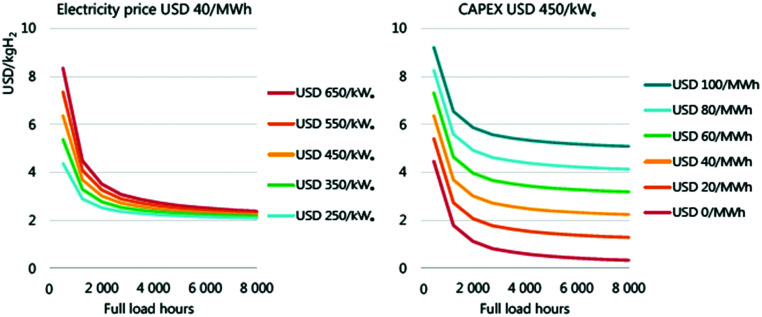
Hypothetical future levelised cost of hydrogen production from electrolysers as a function of capital cost (left) and electricity cost (right). Calculations assume a discount rate of 8% and efficiency of 69% (LHV). Reproduced from IEA.^1889^

Many studies^1889,1892,1903,1936^ consider power prices in the range of $40–60 MW^−1^ h^−1^, as this broadly reflects the long-term average seen across Europe and North America, or $20 MW^−1^ h^−1^ as a sensitivity to reflect the trend of power prices falling as the share of renewable energy increases.^1941^Fig. 170 shows the impact of power price on the cost of delivered hydrogen.

Given the role of water electrolysis in decarbonising energy systems, there is a key focus on ‘green hydrogen’ produced solely from renewable electricity. The cost of electricity generation from solar PV has fallen by a factor of 7 between 2010 and 2020, and for wind it has halved over the same period.^1912^ This is primarily due to falling capital costs which are experienced worldwide, but there are also strong regional variations due to the underlying productivity of wind and solar farms.^1942,1943^

**Fig. 171:**
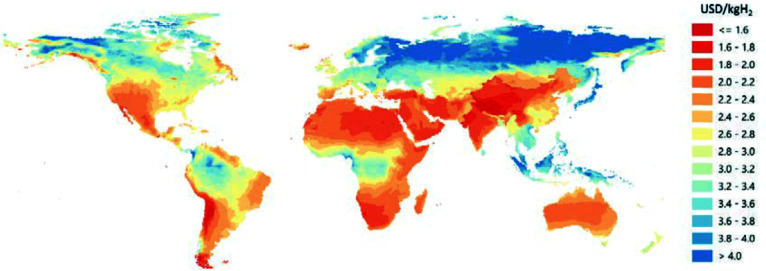
Modelled cost of hydrogen production using solar PV or wind as electricity source. Reproduced from IEA.^1889^

Every region has different solar and wind generation characteristics which would affect hydrogen production and costs if installed (Fig. 171). For regions with high-capacity factors, the cost of electricity generation is cheap, reducing the cost of hydrogen production.

If green hydrogen is produced from hard-linking wind or solar PV with an electrolyser, the lowest cost hydrogen production requires consideration of the trade-off between installed solar/wind capacity and installed electrolysis capacity: this governs the average utilisation rate of the electrolyser.

For a 1 MW electrolyser system, 1 MW of installed wind capacity would supply an average of 400 kW (with an average capacity factor of 40%). The utilisation rate of the electrolyser would be the same as the capacity factor of the wind. To achieve higher electrolyser utilisation and to decrease the levelised electrolyser capex, higher quantities of wind must be installed. The increase in utilisation will be governed by the wind output curve and installing extra capacity will yield an oversupply of electricity at some points during the year. This oversupply could be exported if there is an available connection or used on-site, otherwise it would have to be curtailed. Consequently, there may be a trade-off between lowering cost from increased electrolyser utilisation and increasing cost from curtailed wind capacity.

#### 14.5.3 Hydrogen production cost estimates

Studies have converged around a cost of around $5 per kg for electrolytic hydrogen produced today. This can be converted to $150 MW^−1^ h^−1^*via* the energy content of hydrogen (33.3 kW h per kg at lower heating value)^1944^ to give easier comparison with electricity prices.

IRENA projects that the levelised cost of gas could fall from around $5 kg^−1^ today ($2.70–6 kg^−1^ range depending on conditions) to $1 kg^−1^ in the future.^1903^ Most of this saving comes from two key interventions: an 80% reduction in electrolyser capex (from $750 to $150 kW^−1^) which saves $1.80 kg^−1^; and a halving of electricity input cost (from $53 to $20 MW^−1^ h^−1^) which saves $1.40 kg^−1^.^1903^ Similarly, ETC models hydrogen costs in Europe at being €5.10 kg^−1^ today (assuming $780 kW^−1^ capital costs).^1892^ This could fall to €3.60 kg^−1^ in future with 500 TW h (10 Mt) annual demand for hydrogen, and further to €1.70 kg^−1^ with 1,100 TW h (22 Mt) annual demand. Again, the main savings come from reducing capital costs ($1.30 kg^−1^) and abundant cheap renewable electricity ($1.10 kg^−1^).^1892^

**Fig. 172:**
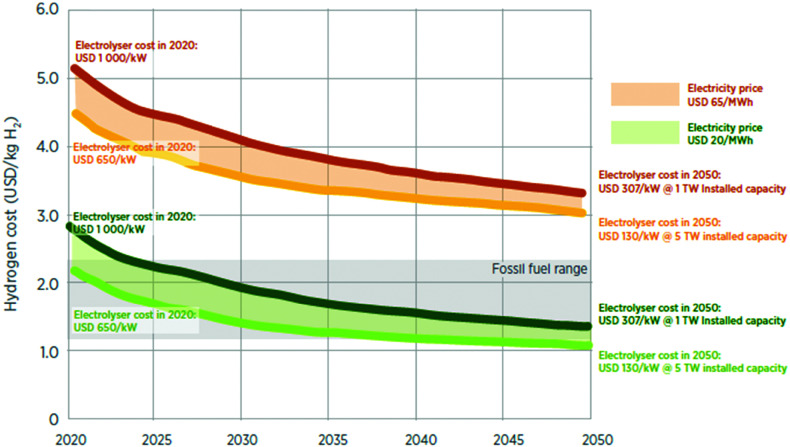
Cost projections for green hydrogen production over time, as a function electrolyser capital cost and electricity price. Reproduced from IRENA.^1903^

Agora is more optimistic, suggesting hydrogen could cost $2.60 kg^−1^ today when using PV in North Africa as the electricity source.^1935^ This cost could fall to $1.90–2.20 kg^−1^ in 2025, and further to $1.30 in 2030 if there is convergence towards Chinese manufacturing costs ($115 kW^−1^), or to $1.90 kW^−1^ with IEA's assumption for minimum capex.^1935^ The influence of the key drivers is summarised in Fig. 172.

Academic studies similarly estimate hydrogen production costs of $4.50 kg^−1^ ^1945^ when using offshore wind, and $5.00–6.10 kg^−1^ when using renewable energy;^1946^ in niche applications, although not yet for industrial-scale $3.48 kg^−1^.^1905^ Provided that recent market trends continue the hydrogen production costs are assumed to reduce to $2.7 kg^−1^. These scenarios also agree with current industry announcements. Areva H2Gen report a cost of $3.90 kg^−1^ from a fully-utilised 1 MW PEMWE system (8000 operating hours per year) at a power price of $55 MW^−1^ h^−1^.^1947^ Enapter whises to reduce the cost of hydrogen from their household-scale (2.4 kW) AEMWEs from $7.60 in 2020 to $1.60 kg^−1^ in 2030, plus around $3 kg^−1^ for electricity consumed.^1947^

#### 14.5.4 Comparison to other technologies

**Fig. 173:**
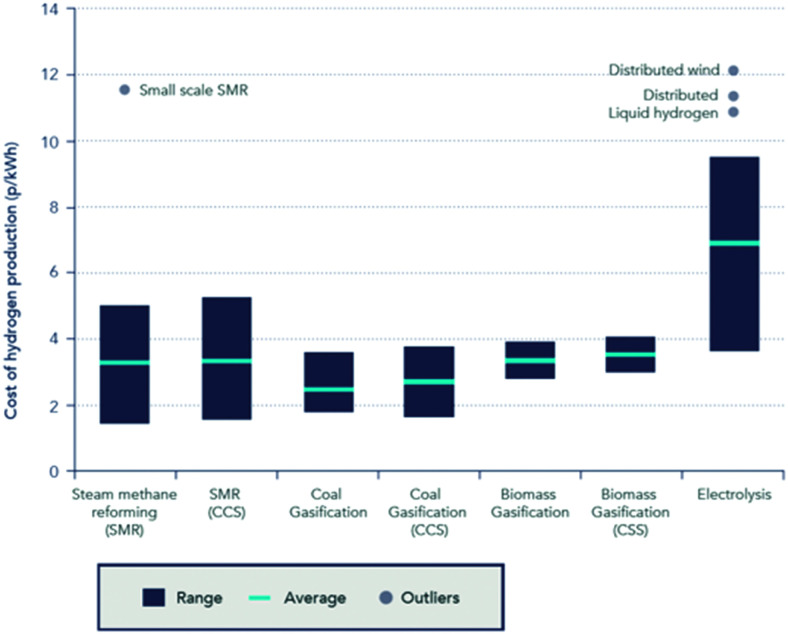
Levelised cost of hydrogen production from different production technologies. Reproduced from ref. 1948 and 1949.

Producing hydrogen from electrolysis has been the highest cost yet lowest emission form of hydrogen generation. As shown in Fig. 173, production of hydrogen from fossil fuels is the cheapest option, following by fossil fuel production with carbon capture and storage and biomass gasification. Electrolysis has been seen as approximately twice the cost of the alternative methods but this may change in the future as the cost of electrolysis and low carbon electricity generation becomes ever cheaper and manufacturing scale-up is realised.

**Fig. 174:**
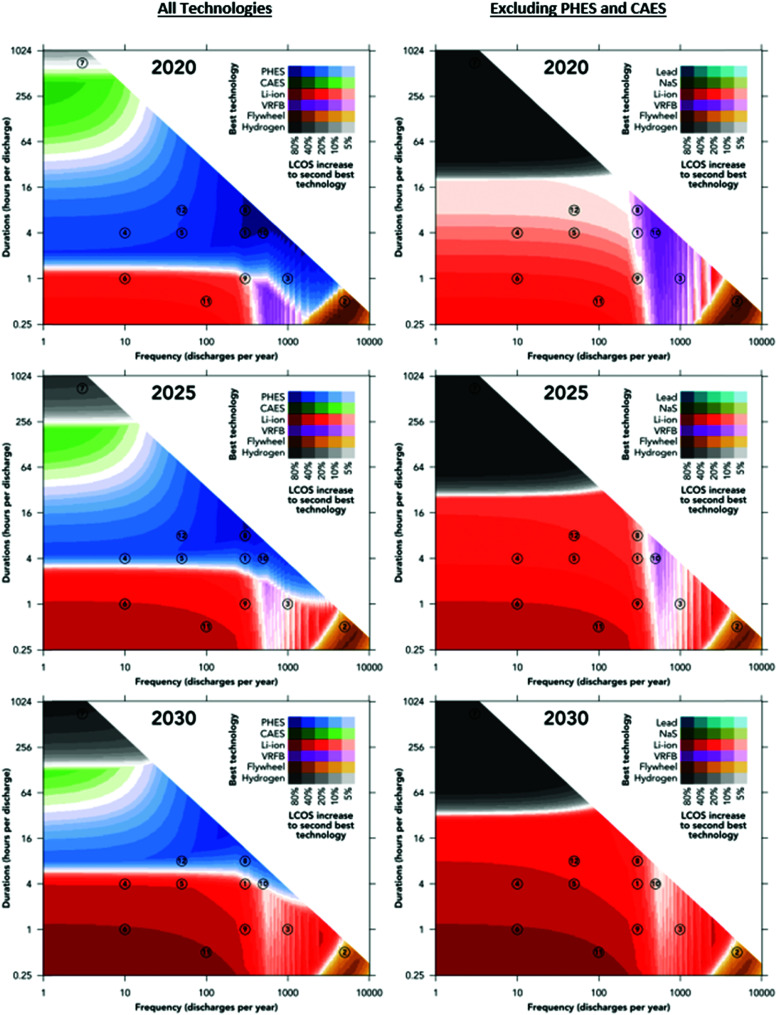
The most cost-effective storage technology, in terms of lowest levelised cost of storage, as a function of the application requirement. Each panel shows the technology with the lowest levelised cost for all possible combinations discharge duration and annual cycle requirements. Left panels consider all modelled technologies, and right panels exclude pumped hydro and underground compressed air (as these have geological pre-requirements). Circled numbers represent the requirements of 12 common power-systems applications which are monetised. Colours represent technologies with lowest LCOS. Shading indicates the difference in levelised cost between the best and second-best technologies, so darker areas indicate a strong cost advantage of the prevalent technology.

However, the cost of electrolysing hydrogen and then converting it back to electricity is favourable compared to other energy storage technologies. Schmidt *et al.*^1936^ calculated the levelised cost of storage for several technologies (including electrochemical, mechanical, pumped hydro) across all major power systems applications, and projected these into the future based on experience rates and market growth scenarios. The most cost-effective storage technology for the full spectrum of applications is shown in Fig. 174.

Hydrogen storage (comprising electrolysis and a fuel cell) was found to be especially effective for long-duration seasonal storage due to its technical characteristics. At present, hydrogen is the lowest-cost technology with more than one month (7000 h) of discharge time; and in regions of the world which cannot use pumped hydro or underground compressed air storage, hydrogen is the lowest-cost solution for discharge durations beyond one day. The operating window in which hydrogen is cost competitive is expected to broaden over time as its costs should fall more rapidly than those for mature pumped hydro.

## 15 Summary and outlook

In the present contribution, water electrolysis is addressed in a comprehensive manner, with insights spanning from textbook knowledge to the latest scientific strategies and industrial developments. The contribution bases its argumentation on thorough and relevant literature on the topic, and details key aspects of water electrolysis in 14 sections.

Firstly, Section 2 gave insight into the fundamentals of the two reactions that take place in water electrolysers: the HER at the negative electrode (where H_2_ is produced) and the OER at the positive electrode (where O_2_ is produced). The basic mechanisms of these reactions are given, with special insights into their limiting steps, which enables to pave the way to optimised electrocatalysts discovery and better electrode engineering.

Section 3 overviewed the various water electrolyser technologies; while high-temperature systems (solid oxide electrolyser cell (SOEC), proton conducting ceramic electrolyser cell (PCCEC)) are in-principle more efficient, they are also submitted to harsh materials constraints, which requires a wealth of engineering optimisation and until now prevented their commercialisation. Molten carbonate electrolyser cell (MCEC), although less studied from a scientific perspective (the materials issues seem handleable), received increasing attention on the industrial side recently, and could become commercial in a close future. Low-temperature water electrolysers are now commercial. Alkaline water electrolyser (AWE) are commercialised since decades and AWE systems are robust and do not depend on platinum group metals, but are also less intensive and efficient, incompatible with intermittent operation and H_2_ compression, unlike their proton exchange membrane counterparts (PEMWE). The latter enable better performances, but are limited by the costs of their constitutive materials. Last, anion exchange membrane water electrolysers (AEMWE), although still at their infancy, could combine the interests of AWE (no PGM catalysts) and of PEMWE (thin membrane for good gas separation, compatibility with intermittency and H_2_ compression); intense research efforts are presently devoted to PEMWEs and AEMWEs.

Section 4 listed key performance indicators (KPI) and technology targets for these systems, with special emphasis to low-temperature water electrolysers, which have more chance to meet wide-scale commercialisation in the next decade.

Section 5 emphasised the need of research in terms of materials science and electrochemistry for the various technologies evaluated in this review. Then, Sections 6–8 focused on practical research efforts for the various families of electrode materials that are (or could be) employed in low-temperature water electrolysers.

Section 6 starts by a short review of state-of-the-art PGM-based catalysts for the HER and OER. It emphasises the fact that, if their today's performances are acceptable, the target is to keep these performances at smaller PGM-loading, which can be achieved by downsizing the particles/crystallites size, and/or alloying the active material (Pt, Ir) with less costly elements, and/or supporting them on stable conductive substrates (two strategies which may influence the activity and stability of the obtained composite, in good or in bad). The poor abundance of PGM in the Earth's crust motivates the search for alternative (non-PGM-based) catalysts.

Section 7 reported about the very comprehensive literature dealing with PGM-free based HER and OER electrocatalysts; obviously, many of the references are related to materials for AWEs (and AEMWEs), but some of it also addresses PEMWEs. Among this rich literature, some concerns metal dioxides as OER and HER electrocatalysts (PbO_2_ and MnO_2_ as electrode material for oxygen evolution). Metal oxides in the perovskite or spinel structure as OER and HER electrocatalysts are also surveyed, as well as transition metal layered double hydroxide OER catalysts for alkaline electrolytes. Finally, a rather recently-investigated class of non-PGM materials will be evaluated as well: steel-based electrodes for both HER and OER electrocatalysis. All these materials (and in particular the transition metal layered double hydroxides and steels) will have a chance to be employed in future A(EM)WE systems. As far as PEMWEs are concerned, durability issues when using non-PGM are a bit harder to handle, and these materials should not be used in such systems in the next decade.

Because metals in general may experience scarcity if used at the large scale (even for the non-PGM) mentioned in Section 7, Section 8 addressed the intense research efforts of the scientific community into metal-free (or with ultra-small metal content) HER and OER electrocatalysts. Catalysts with a carbon skeletal structure are dealt with first, and then heteroatom-doped carbons for OER, bifunctional catalysts and catalysts with a carbon-nitrogen skeletal structure, such as carbon nitride-graphene composites-based catalysts with high N content. Although not deployed industrially, these materials may be part of the solution in the long-term.

Since the performance of a given catalyst in a real water electrolysis cell depends not only on its intrinsic activity but also (and very importantly) on the way it is used in gas generating electrodes, Section 9 provides detailed basic concepts of 2D and 3D-electrode preparation. The section highlighted manners to elaborate HER and OER catalysts, but also how to prepare electrodes and membrane electrode assemblies to be used in practical systems.

Molecular compounds for HER and OER is a topic where the research community is very productive. Inspired by nature, these materials have some assets (selectivity, turnover frequency), but the poor accessibility of their active site and low durability are two real challenges to their practical usage. They were surveyed in Section 10.

Section 11 reviewed methods to characterise both electrocatalysts materials and electrodes. These span from two-electrode cell characterisations of the full electrolysis cell (possible in real system), three-electrode cell characterisations of individual electrodes (usually performed at the laboratory scale in more model conditions); importantly, physicochemical techniques coupled to electrochemistry are also addressed, the literature being extremely rich on the subject, because these are mandatory characterisations to unveil how water electrolysers and their core materials operate, a prerequisite to mechanisms determination and materials/structures optimisation.

Section 12 then evaluated how externally-applied fields like mechanical stirring, magnetic field and ultrasound could enhance water splitting, these strategies usually being applied for low-temperature cells.

Finally, because purified-water is by far not the most abundant and easily-available on the planet, Section 13 focused on water splitting from non-pure water (saline water, seawater, neutral water pH, wastewater), while Section 14 provided a solid cost-analysis.

The wealth of information contained in this review shows how dynamic research is on the topic of water electrolysis. It makes clear that materials science and electrochemistry are at the basis of new discoveries of more efficient electrode/electrolyte materials, but one must not lose sight that engineering of these materials is also mandatory to turn them into long-lasting efficient electrodes for water splitting.

### Outlook

The recent abnormal climatic episodes experienced in summer 2021 in Germany/Belgium (extreme flooding) and USA/Canada (severe droughts and subsequent gigantic forest fires) made even clearer the sad reality of major climate disturbance on our planet. It makes no doubt it is caused by global warming, itself related to major release of greenhouse gases in the atmosphere since the industrial revolution. The strategy to mitigate this issue is clear: the greenhouse gases emissions must be (very) significantly cut. One manner to do so is to rely more on renewable energies, which implies that renewable electricity is efficiently stored at the large scale and the long-term. *Power-to-hydrogen* (and then conversion of hydrogen into electricity in fuel cells) is an obvious strategy to that goal.

Water electrolysis when driven by renewable electricity represents a green, *i.e.*, a CO_2_ footprint free *power-to-hydrogen* route. To effectively counteract global temperature rise, greenhouse gas reducing techniques must be enforced internationally or, in other words, water electrolysis will only make a substantial contribution to the reduction of greenhouse gases emissions if it is widely deployed. The cross-border application of water electrolysis technologies however presupposes that it can be adapted to the different circumstances of the countries (costs for electricity, global solar radiation, availability of wind-active centers, availability of water). A technology always has the best chance of asserting itself if it is cheaper than competing processes, *i.e.*, in this case water electrolysis needs to become more economical than processes that are based on the exploitation of oil, coal and natural gas, this being completely independent on the fact that the last-mentioned methods counteract the goal of reducing the emission of greenhouse gases. The economy of water electrolysis is not only determined by the physical-chemical efficiency based on the cell voltage necessary to end up in a certain current density in combination with the charge to gas conversion rate. In addition to the durability of the electrode material in particular and the overall maintenance costs, the acquisition costs due to the electrode materials and the device design also play a role. Nevertheless, optimization of water electrolysis electrodes, *i.e.*, the improvement of OER and HER electrocatalysts and the intensification of the electrocatalyst-conductive support interaction is and remains amongst the most important adjustment screw that needs to be turned in order to make a significant leap towards highly efficient electrocatalytic water splitting.

Some of the authors have worked extensively on perovskite-based OER electrode materials. Many recently published articles report on composite materials containing perovskite as the active component for the OER. Although much research effort has been devoted to the development of OER-active perovskites, we believe that the development of composites that support perovskites as the OER electrocatalyst and ensures an intense, synergistic interaction between the electrocatalyst and the conductive support or at the electrocatalyst/interlayer interface, is a sensible strategy worth pursuing. This certainly also applies to spinel-based water-splitting electrodes. More complex spinel-containing hybrid materials have recently emerged as highly efficient supported OER catalysts. Further strategies to increase the number of OER active sites as *e.g.*, cations filling of unoccupied interstices leading to cationic misalignment need to be implemented into a broader spectrum of spinel for OER electrocatalysis. In addition, spinel-type materials are promising HER supporting materials giving them bifunctionality.

A more thorough investigation of effects that occur when nonmetals such as S, P are incorporated into transition-metal based spinels could lead to a more informed knowledge-based development of useful material design strategies and should result in more HER-active spinel's. However, currently metal pnictides, metal carbides, metal borides, metal chalcogenides are more competitive HER-promoting electrocatalysts; binary borides and carbides are among the best binary HER electrocatalysts in terms of both activity and durability. Among the materials that consist of metal elements and non-metal elements (main groups 3, 4, 5 and 6), hybrid composed phases with coexisting metallic and a non-metal rich phase belong to the absolute bench mark species. This has been shown, for example, for molybdenum nitride-based electrode materials. This concept should be extended and successfully transferred to other metal/nonmetal compounds. Besides the further understanding and improvement of well-established metal/non-metal based HER active compounds we recommend the more intensive investigation of up to now less investigated electrocatalytic active metal/non-metal composed compounds as for instance transition metal tellurides. From a theoretical point-of-view tellurides (in general) should not be less active than the lighter homologues of the sixth main group.

Steels have proven to be outstandingly efficient and outstandingly durable as electrode materials for water electrocatalysis. Recently, suspension-based approaches have emerged; *e.g.*, it has been found that transition metal oxides are reasonable additions to sulfuric acid-based electrolytes. Currently, however, the amount of solid material that needs to be added to the clear electrolyte to have the desired effect, *i.e.*, to significantly reduce the overpotential required to achieve a given current density is high and is about 30 g per 100 mL of electrolyte. This means that adapting to current electrolyzer technologies is almost impossible. Therefore, scientists should aim to significantly reduce the required amount of added oxidic solid compound.

Generally, non-metal based electrocatalysts can still not compete with metal-based ones with respect to efficiency for OER or HER electrocatalysis. The improvement of the contact between conductive support and the periphery composed of the real catalytic active phase seems to be a promising route to increase the catalytic activity. Besides doping of the conductive host lattice, *e.g.*, graphitic carbon nitride matrix with P or S or P and S was already found to be an effective way to manipulate electronic structure and electrochemical properties which means not only the increased catalytic efficiency but also the reduction of the intrinsic susceptibility for carbon to corrode in OER regime.

The authors think that the embedding of molecular compounds into conductive support might indeed become a promising strategy to result in effective (heterogeneous) water electrocatalysis. Thus, whenever molecular species are used in solid-state electrocatalysis, at least reasonable catalytic efficiency can be achieved. However, to date, molecular compounds that enable homogeneous water catalysis have not been able to represent a viable strategy to achieve competitive current densities at moderate overpotentials.

Besides the optimisation of the electrode materials there are several methods for reducing the total overpotential and total Ohmic resistance in water electrolysis, for example, by increasing the electrolyte movement (by using gravity, centrifugal acceleration field, mechanical stirring, magnetic field; employing ultrasound at the gas-evolving electrodes and electrolyte). These are promising strategies to further increase the efficiency of water electrolysis and in particular the combination of externally applied fields with newly developed state-of-the-art electrode materials and cell designs should be further investigated.

Among electrolyser technologies, in particular AEMWE is very promising in terms of total cost of ownership. Although this technique has been intensively investigated lately, studies on cell performance stability remain rare. The existing studies that comprise performance stability tests for AEMWE at constant current density showed a substantial reduction in already about 100 hours after commissioning, probably owing to chemical degradation of the anion conducting polymers at high pH value. Thus, on the one hand, further tests examining the durability of the membrane in long-term use are urgently needed, and further improvement of the membrane against base-related degradation must be tackled. Also, the interfacial contact between the anion-exchange membrane (AEM) and the catalytic layers need to be further optimized, as it can simply not be prepared by coupling conventional AWE electrodes with an AEM. These materials and chemical-engineering aspects should without any doubt be seriously handled by the research community in the future, if one wants to have AEMWEs at large for the storage of renewable electricity. The same applies as well for PEMWEs, the goal of engineering being in that case to lower the amount of PGM used in the electrodes and to reach longer service-life in real (renewable electricity storage) operation.

Finally, and although this has not been amongst the primary points of focus of this review, working on system aspects, balance-of-plant and control and command of the water electrolyzer (regardless of the technology used) is not less important.

We encourage the scientists involved in this striking field of research to avoid taking wrong turns. To give an example, water purification techniques have been very well established and with regard to economic criteria, direct saltwater electrolysis is almost always surpassed by a combination of the latest deionization technology plus alkaline water electrolysis (AWE) or proton exchange membrane (PEM) water electrolysis. The authors therefore think that further investing resources in exploration of the direct electrolysis of seawater is at least worthy of discussion.

This shows that, in the present times where hydrogen is called to become a major energy vector in our societies, the research community as a whole as multiple challenges to handle, which should find their solutions be a clever coupling between complementary multidisciplinary approaches spanning from basic materials sciences (electrocatalysis, polymer chemistry and physics), chemical and materials engineering all the way to mechanical and electrical engineering. This is the only solution by which the complex systems that are water electrolyzers will become sufficiently technologically-advanced and economically-viable to be deployed at large (and coupled to fuel cells or other hydrogen-using devices), an endeavor to lower our greenhouse gases emissions.

## Author contributions

MC (particularly) wrote Section 2 + Section 6 (partly) as well as Sections 11 and 15. He edited the manuscript as a whole in detail. BGP wrote Section 12 as well as Section 6. MZB wrote Section 3.2.1 and particularly edited Section 2. DD wrote Section 5.2 and edited the manuscript as a whole in detail. FD wrote Section 7.4. JD evaluated Sections 2 and 6 as well as Sections 11 and 15. PM wrote Sections 3, 9 and 5.3 and edited the manuscript as a whole in detail. RDB partly wrote Section 1 and edited the manuscript as a whole in detail. ME wrote Section 2. IS wrote Section 14 and he edited the manuscript as a whole in detail. He was, in addition responsible for finalizing the main text and SI files prior submission. PB wrote/edited Section 14. YSH wrote Section 1 and basically edited sections dedicated to the materials parts. HS wrote Sections 7.1, 7.2, 7.3, 7.5, 7.6, 8, 10, 13 and partly contributed to Section 2.

## Conflicts of interest

There are no conflicts to declare.

## Supplementary Material

CS-051-D0CS01079K-s001

## References

[cit1] “Sir David Attenborough: We Must Act on Population”. Population Matters. *5 October 2018.* Retrieved 1 December 2019

[cit2] Gregus J., Guillebaud J. (2020). Eur. J. Contracept. Reprod. Health Care.

[cit3] Bongaarts J., O’Neill B. C. (2018). Science.

[cit4] Dincă M., Surendranath Y., Nocera D. G. (2010). Proc. Natl. Acad. Sci. U. S. A..

[cit5] Poizot P., Dolhelm F. (2011). Energy Environ. Sci..

[cit6] Deetz R. J., Reek J. N.-H., van der Zwaan B. C.-C. (2018). Energy Environ. Sci..

[cit7] Beaudin M., Zareipour H., Schellenberglabe A., Rosehart W. (2010). Energy Sustain. Dev..

[cit8] Staffell I., Pfenninger S. (2018). Energy.

[cit9] Hauer A. , Quinnell J. and Lavemann E., Energy Storage Technologies – Characteristics, Comparison, and Synergies, in Transfer to Renewable Energy Systems, ed. D. Stolten and V. Scherer, Wiley-VCH Verlag GmbH & Co., Weinheim, Germany, 2013, ch. 27, pp. 555–577

[cit10] *The Future of Hydrogen: Seizing Today's Opportunities*, International Energy Agency, Paris, France, June 2019. https://www.iea.org/reports/the-future-of-hydrogen

[cit11] Kudo A., Miseki Y. (2009). Chem. Soc. Rev..

[cit12] Walter M. G., Warren E. L., McKone J. R., Boettcher S. W., Mi Q., Santori E. A., Lewis N. S. (2010). Chem. Rev..

[cit13] Bard A. J., Fox M. A. (1995). Acc. Chem. Res..

[cit14] Cook T. R., Dogutan D. K., Reece S. Y., Surendranath Y., Teets T. S., Nocera D. G. (2010). Chem. Rev..

[cit15] Kanan M. W., Surendranath Y., Nocera D. G. (2009). Chem. Soc. Rev..

[cit16] Artero V., Chavarot-Kerlidou M., Fontecave M. (2011). Angew. Chem., Int. Ed..

[cit17] Zeng K., Zhang D. (2010). Prog. Energy Combust. Sci..

[cit18] Buttler A., Spliethoff H. (2018). Renewable Sustainable Energy Rev..

[cit19] U.S. Energy Information Administration, International Energy Outlook 2019

[cit20] Bernt M., Hartig-Weiß A., Tovini M. F., El-Sayed H. A., Schramm C., Schröter J., Gebauer C., Gasteiger H. A. (2020). Chem. Ing. Tech..

[cit21] Varcoe J. R., Atanassov P., Dekel D. R., Herring A. M., Hickner M. A., Kohl P. A., Kucernak A. R., Mustain W. E., Nijmeijer K., Scott K., Xu T., Zhuang L. (2014). Energy Environ. Sci..

[cit22] Schäfer H., Chatenet M. (2018). ACS Energy Lett..

[cit23] Lamy C., Millet P. (2020). J. Power Sources.

[cit24] Wei C., Rao R. R., Peng J., Huang B., Stephens I. E.-L., Risch M., Xu Z. J., Shao-Horn Y. (2019). Adv. Mater..

[cit25] Lee Y., Suntivich J., May K. J., Perry E. E., Shao-Horn Y. (2012). J. Phys. Chem. Lett..

[cit26] Tackett B. M., Sheng W., Chen J. G. (2017). Joule.

[cit27] Fabbri E., Nachtegaal M., Binninger T., Cheng X., Kim B. J., Durst J., Bozza F., Graule T., Schaublin R., Wiles L., Pertoso M., Danilovic N., Ayers K. E., Schmidt T. J. (2017). Nat. Mater..

[cit28] Han B., Stoerzinger K. A., Tileli V., Gamalski A. D., Stach E. A., Shao-Horn Y. (2017). Nat. Mater..

[cit29] Niether C., Faure S., Bordet A., Deseure J., Chatenet M., Carrey J., Chaudret B., Rouet A. (2018). Nat. Energy.

[cit30] Zhu Y., Chen G., Zhong Y., Chen Y., Ma N., Zhou W., Shao Z. (2018). Nat. Commun..

[cit31] Gatard V., De Masi D., Chattot R., Marin I. M., Revert J. M. A., Fazzini P. F., Encinas T., Martin V., Faure S., Deseure J., Carrey J., Chaudret B., Chatenet M. (2021). Electrocatalysis.

[cit32] Dinh Nguyen M.-T., Ranjbari A., Catala L., Brisset F., Millet P., Aukauloo A. (2012). Coord. Chem. Rev..

[cit33] Pizzutilo E., Geiger S., Grote J. P., Mingers A., Mayrhofer K. J.-J., Arenz M., Cherevko S. (2016). J. Electrochem. Soc..

[cit34] Abbou S., Chattot R., Martin V., Claudel F., Solà-Hernández L., Beauger C., Dubau L., Maillard F. (2020). ACS Catal..

[cit35] Kurtz J. , Sprik S., Saur G. and Onorato S., *Fuel Cell Electric Vehicle Durability and Fuel Cell Performance*, National Renewable Energy Laboratory (NREL), Denver, Colorado Technical Report NREL/TP-5400-73011, March 2019. https://www.nrel.gov/docs/fy19osti/73011.pdf

[cit36] Pollet B. G., Kocha S. S., Staffell I. (2019). Curr. Opin. Electrochem.

[cit37] Price E. (2017). Johnson Matthey Technol. Rev..

[cit38] EUR 29267 EN, JRC107053 , in EU Harmonised Test Procedure: Electrochemical Impedance Spectroscopy for Water Electrolysis Cells, ed. T. Malkow, A. Pilenga and G. Tsotridis, Publications Office of the European Union, Luxembourg, 2018 10.2760/67321

[cit39] EUR 29300 EN, JRC112082 , in EU Harmonised Terminology for Low Temperature Water Electrolysis for Energy Storage Applications, ed. G. Tsotridis and A. Pilenga, Publications Office of the European Union, Luxembourg, 2018 10.2760/014448

[cit40] Schäfer H., Chevrier D. M., Kuepper K., Zhang P., Wollschläger J., Daum D., Steinhart M., Hess C., Krupp U., Müller-Buschbaum K. (2016). Energy Environ. Sci..

[cit41] Schäfer H., Küpper K., Müller-Buschbaum K. M., Daum D., Steinhart M., Wollschläger J., Krupp U., Schmidt M., Han W., Stangl J. (2017). Nanoscale.

[cit42] Durst J., Siebel A., Simon C., Hasché F., Herranz J., Gasteiger H. A. (2014). Energy Environ. Sci..

[cit43] Hamann C. H. and Vielstich W., Elektrochemie, Wiley-VCH Verlag GmbH & Co., Weinheim, Germany, 4th edn, 2015

[cit44] Huang J., Chen S., Eikerling M. (2021). J. Chem. Theory Comput..

[cit45] Tesch R., Kowalski P. M., Eikerling M. H. (2021). J. Phys: Condens. Matter.

[cit46] Jun H., Malek A., Zhang J., Eikerling M. H. (2016). J. Phys. Chem. C.

[cit47] Huang J., Zhu X., Eikerling M. (2021). Electrochim. Acta.

[cit48] Jun H., Zhang J., Eikerling M. (2018). Phys. Chem. Chem. Phys..

[cit49] Santos D. M.-F., Cesar A. C., Sequeira J. L., Figueiredo J. L. (2013). Quim. Nova.

[cit50] Mittelsteadt C. , Norman T., Rich M. and Giner J. W., PEM Electrolyzers and PEM Regenerative Fuel Cells Industrial View, in Electrochemical Energy Storage for Renewable Sources and Grid Balancing, Elsevier, Waltham, MA, 2015, ch. 11, pp. 159–181

[cit51] Bockris J. O.'M. and Reddy A. K.-N., Modern Electrochemistry 1, Plenum Publishing Corporation, New York, 1977, ch. 1–4, vol. 1, pp. 1–460

[cit52] Bockris J. O.'M. , Reddy A. K.-N. and Gamboa-Aldeco M., Modern Electrochemistry 2A, Kluwer Academic/Plenum Publishers, New York, 2nd edn, 2018, ch. 6–7, pp. 771–1216

[cit53] Eikerling M. and Kulikovsky A. A., Polymer Electrolyte Fuel Cells – Physical Principles of Materials and Operation, CRC Press, 2014

[cit54] Bazant M. Z. (2013). Acc. Chem. Res..

[cit55] Bandal H. A., Jadhav A. R., Kim H. (2017). J. Alloy Comp..

[cit56] Phillips R. , Gannon W. J. F. and Dunnill C. W., Alkaline Electrolysers, Electrochemical Methods for Hydrogen Production, The Royal Society of Chemistry, 2020, ch. 2, pp. 28–58

[cit57] Schillings J., Doche O., Tano Retamales M., Bauer F., Deseure J., Tardu S. (2017). Int. J. Multiph. Flow.

[cit58] Lasia A. (2019). Int. J. Hydrogen Energy.

[cit59] Anantharaj S., Ede S. R., Karthick K., Sankar S. S., Sangeetha K., Karthik P. E., Kundu S. (2018). Energy Environ. Sci..

[cit60] Shi Y., Zhang B. (2016). Chem. Soc. Rev..

[cit61] Fabbri E., Habereder A., Waltar K., Kötz R., Schmidt T. J., Kotz R., Schmidt T. J., Kötz R., Schmidt T. J., Kotz R., Schmidt T. J. (2014). Catal. Sci. Technol.

[cit62] McKone J. R., Marinescu S. C., Brunschwig B. S., Winkler J. R., Gray H. B. (2014). Chem. Sci..

[cit63] Zhang Q., Guan J. (2020). J. Power Sources.

[cit64] Conway B. E., Sattar M. A., Gilroy D. (1969). Electrochim Acta.

[cit65] Conway B. E., Liu T. C. (1987). J. Chem. Soc. Faraday Transact.

[cit66] Hoare J. P. , in Advances in Electrochemistry and Electrochemical Engineering, ed. P. Delahay and C. W. Tobias, Interscience, New York, 1966, vol. 6, pp. 201–288

[cit67] Tseung C. C., Jasem S. (1977). Electrochim. Acta.

[cit68] Pourbaix M. , Atlas d‘Équilibres Électrochemiques a 25 °C, Gauthiers-Villars, Paris, 1963

[cit69] Doyle R. L., Godwin I. L., Brandon M. P., Lyons M. E.-C. (2013). Phys. Chem. Chem. Phys..

[cit70] Mavros M. G., Tsuchimochi T., Kowalczyk T., McIsaac A., Wang L.-P., Van Voorhis T. (2014). Inorg. Chem..

[cit71] Reier T., Nong H. N., Teschner D., Schlögl R., Strasser P. (2017). Adv. Energy Mater..

[cit72] Doyle R. L., Lyons M. E.-C. (2013). Phys. Chem. Chem. Phys..

[cit73] Li X., Cheng Z., Wang X. (2021). Electrochem. Energy Rev..

[cit74] Polyansky D. E., Muckerman J. T., Rochford J., Zong R., Thummel R. P., Fujita E. (2011). J. Am. Chem. Soc..

[cit75] Giuseppe M., Giannozzi P., Bonapasta A. A., Guidoni L. (2013). J. Am. Chem. Soc..

[cit76] Nakagawa T., Beasley C. A., Murray R. W. (2009). J. Phys. Chem. C.

[cit77] Pan Y., Xu X., Zhong Y., Ge L., Chen Y., Veder J.-P. M., Guan D. (2020). Nat. Commun..

[cit78] Diaz-Morales O., Ferrus-Suspedra D., Koper M. T.-M. (2016). Chem. Sci..

[cit79] Garcia A. C., Touzalin T., Nieuwland C., Perini N., Koper M. T.-M. (2019). Angew. Chem., Int. Ed..

[cit80] Stoerzinger K. A., Diaz-Morales O., Kolb M., Rao R. R., Frydendal R., Qiao L., Wang X. R. (2017). ACS Energy Lett..

[cit81] Fernandez E. M., Moses P. G., Toftelund A., Hansen H. A., Martinez J. I., Abild-Pedersen F., Kleis J., Hinnemann B., Rossmeisl J., Bligaard T., Norskov J. K. (2008). Angew. Chem..

[cit82] Norskov J. K., Rossmeisl J., Logadottir A., Lindqvist L., Kitchin J. R., Bligaard T., Jonsson H. (2004). J. Phys. Chem. B.

[cit83] Gasteiger H. A., Kocha S. S., Sompalli B., Wagner F. T. (2005). Appl. Catal., B.

[cit84] Neyerlin K. C., Gu W., Jorne J., Gasteiger H. A. (2007). J. Electrochem. Soc..

[cit85] Zalitis C. M., Kramer D., Kucernak A. R. (2013). Phys. Chem. Chem. Phys..

[cit86] Anantharaj S., Noda S. (2020). Small.

[cit87] Bockris J. O. ’M., Potter E. C. (1952). J. Chem. Phys..

[cit88] Conway B. E., Salomon M. (1964). Electrochim. Acta.

[cit89] Tilak B. V., Chen C. P. (1993). J. Appl. Electrochem..

[cit90] Chen L. (1996). J. Electrochem. Soc..

[cit91] Jaksic J., Vojnovic M., Krstajic N. (2000). Electrochim. Acta.

[cit92] Mahmood N., Yao Y., Zhang J. W., Pan L., Zhang X., Zou J. J. (2018). Adv. Sci..

[cit93] Yao Z., Yan J., Anthony V., Qiao S. Z. (2018). Angew. Chem., Int. Ed..

[cit94] Oshchepkov A. G., Bonnefont A., Parmon V. N., Savinova E. R. (2018). Electrochim. Acta.

[cit95] Oshchepkov A. G., Bonnefont A., Saveleva V. A., Papaefthimiou V., Zafeiratos S., Pronkin S. N., Parmon V. N., Savinova E. R. (2016). Top. Catal..

[cit96] Oshchepkov A. G., Braesch G., Bonnefont A., Savinova E. R., Chatenet M. (2020). ACS Catal..

[cit97] Oshchepkov A. G., Bonnefont A., Savinova E. R. (2020). Electrocatalysis..

[cit98] Trasatti S. (1972). J. Electroanal. Chem. Interfacial Electrochem..

[cit99] Sabatier P. (1911). Ber. Deut. Chem. Ges..

[cit100] Balandin A. A. (1946). Zh. Obshei Khimii.

[cit101] Hansen J. N., Prats H., Toudahl K. K., Secher N. M., Chan K., Kibsgaard J., Chorkendorff I. (2021). ACS Energy Lett..

[cit102] Jun H., Li M., Eslamibidgoli M. J., Eikerling M., Groß A. (2021). J. Am. Chem. Soc..

[cit103] Burke M. S., Kast M. G., Trotochaud L., Smith A. M., Boettcher S. W. (2015). J. Am. Chem. Soc..

[cit104] Trotochaud L., Young S. L., Ranney J. K., Boettcher S. W. (2014). J. Am. Chem. Soc..

[cit105] Friebel D., Louie M. W., Bajdich M., Sanwald K. E., Cai Y., Wise A. M., Cheng M.-J., Sokaras D., Weng T.-C., Alonso-Mori R., Davis R. C., Bargar J. R., Nørskov J. K., Nilsson A., Bell A. T. (2015). J. Am. Chem. Soc..

[cit106] Tkalych A. J., Houlong L., Carter E. A. (2017). ACS Catal..

[cit107] Eslamibidgoli M. J., Groß A., Eikerling M. (2017). Phys. Chem. Chem. Phys..

[cit108] Mohammad M. J., Huang J., Kowalski P. M., Eikerling M. H., Groß A. (2021). Electrochim. Acta.

[cit109] Ananth G. R., Martirez J. M.-P., Carter E. A. (2020). J. Am. Chem. Soc..

[cit110] Gorlin M., De Araujo J. F., Schmies H., Bernsmeier D., Dresp S., Gliech M., Jusys Z., Chernev P., Kraehnert R., Dau H., Strasser P. (2017). J. Am. Chem. Soc..

[cit111] Xiao H., Shin H., Goddard W. A. (2018). PNAS.

[cit112] Lu Z., Xu W., Zhu W., Yang Q., Lei X., Liu J., Li Y., Sun X., Duan X. (2014). Chem. Commun..

[cit113] Qiu Z., Tai C.-W., Niklasson G. A., Edvinsson T. (2019). Energy Environ. Sci..

[cit114] Liu Z., Wang G., Zhu X., Wang Y., Zou Y., Zang S., Wang S. (2020). Angew. Chem..

[cit115] Lee H.-W., Muralidharan P., Ruffo R., Mari C. M., Cui Y., Kim D. K. (2010). Nano Lett..

[cit116] Rozain C., Rozain C., Mayousse E., Guillet N., Millet P. (2016). Appl. Catal., B.

[cit117] Rozain C., Rozain C., Mayousse E., Guillet N., Millet P. (2016). Appl. Catal., B.

[cit118] Hegge F., Lombeck F., Cruz Ortiz E., Bohn L., von Holst M., Kroschel M., Hübner J., Breitwieser M., Strasser P., Vierrath S. (2020). ACS Appl. Energy Mater..

[cit119] Mo J., Kang Z., Yang G., Retterer S. T., Cullen D. A., Toops T. J., Green J. B., Zhang F.-Y. (2016). Appl. Energy.

[cit120] Schröder J., Mints V. A., Bornet A., Berner E., Fathi Tovini M., Quinson J., Wiberg G. K.-H., Bizzotto F., El-Sayed H. A., Arenz M. (2021). J. Am. Chem. Soc..

[cit121] Kadyk T., Bruce D., Eikerling M. (2016). Sci. Rep..

[cit122] Zeradjanin A. R., Narangoda P., Spanos I., Masa J., Schlögl R. (2021). Curr. Opin. Electrochem..

[cit123] Zeradjanin A. R., Topalov A. A., Van Overmeere Q., Cherevko S., Chen X. X., Ventosa E., Schuhmann W., Mayrhofer K. J.-J. (2014). RSC Adv..

[cit124] Zeradjanin A. R. (2018). Curr. Opin. Electrochem..

[cit125] Eslamibidgoli M. J., Eikerling M. H. (2015). ACS Catal..

[cit126] Halck N. B., Petrykin V., Krtil P., Rossmeisl J. (2014). Phys. Chem. Chem. Phys..

[cit127] Man I. C., Su H.-Y., Calle-Vallejo F., Hansen H. A., Martínez J. I., Inoglu N. G., Kitchin J., Jaramillo T.
F., Nørskov J. K., Rossmeisl J. (2011). Chem. Cat. Chem..

[cit128] Craig M. J., Coulter G., Dolan E., Soriano-López J., Mates-Torres E., Schmitt W., García-Melchor M. (2019). Nat. Commun..

[cit129] Divanis S., Kutlusoy T., Boye I. M.-I., Man I. C., Rossmeisl J. (2020). Chem. Sci..

[cit130] Groß Axel, Sakong Sung (2019). Curr. Opin. Electrochem..

[cit131] Ooka H., Huang J., Exner K. S. (2021). Front. Energy Res..

[cit132] Huang Z.-F., Song J., Dou S., Li X., Wang J., Wang X. (2019). Matter.

[cit133] Song J., Wei C., Huang Z.-F., Liu C., Zeng L., Wang X., Xu Z. J. (2020). Chem. Soc. Rev..

[cit134] Calle-Vallejo F., Koper M. T.-M., Bandarenka A. S. (2013). Chem. Soc. Rev..

[cit135] Buvat G., Eslamibidgoli M. J., Garbarino S., Eikerling M., Guay D. (2020). ACS Appl. Energy Mater..

[cit136] Kasun Kalhara Gunasooriya G. T., Nørskov J. K. (2020). ACS Energy Lett..

[cit137] Yeon Kim D., Ha M., Kim K. S. (2021). J. Mater. Chem. A.

[cit138] Back S., Tran K., Ulissi Z. W. (2020). ACS Appl. Mater. Interfaces.

[cit139] Tran K., Ulissi Z. W. (2018). Nat. Catal..

[cit140] Alberi K., Nardelli M. B., Zakutayev A., Mitas L., Curtarolo S., Jain A., Fornari M., Marzari N., Takeuchi I., Green M. L., Kanatzidis M. (2018). J. Phys. D: Appl. Phys..

[cit141] De Luna P., Wei J., Bengio Y., Aspuru-Guzik A., Sargent E. (2017). Nature.

[cit142] Zhou T., Song Z., Sundmacher K. (2019). Engineering.

[cit143] Freeze J. G., Kelly H. R., Batista V. S. (2019). Chem. Rev..

[cit144] Malek A., Wang Q., Baumann S., Guillon O., Eikerling M., Malek K. (2021). Front. Energ. Res..

[cit145] Tabor D. P., Roch L. M., Saikin S. K., Kreisbeck C., Sheberla D., Montoya J. H., Dwaraknath S., Aykol M., Ortiz C., Tribukait H., Amador-Bedolla C. (2018). Nat. Rev. Mater..

[cit146] Häse F., Roch L. M., Aspuru-Guzik A. (2019). Trends Chem..

[cit147] Debe M. K. (2012). Nature.

[cit148] Eslamibidgoli M. J., Huang J., Kadyk T., Malek A., Eikerling M. (2016). Nano Energy.

[cit149] Gloaguen F., Andolfatto F., Durand R., Ozil P. (1994). J. Appl. Electrochem..

[cit150] Gloaguen F., Durand R. (1997). J. Appl. Electrochem..

[cit151] Bultel Y., Genies L., Antoine O., Ozil P., Durand R. (2002). J. Electroanal. Chem..

[cit152] Chan K., Eikerling M. (2012). J. Electrochem. Soc..

[cit153] Sadeghi E., Putz A., Eikerling M. (2013). J. Electrochem. Soc..

[cit154] Bultel Y., Ozil P., Durand R. (1998). J. Appl. Electrochem..

[cit155] Nørskov J. K., Bligaard T., Logadottir A., Kitchin J. R., Chen J. G., Pandelov S., Stimming U. (2005). J. Electrochem. Soc..

[cit156] Exner K. S. (2021). Electrochim. Acta.

[cit157] Bandarenka A. S., Koper M. T.-M. (2013). J. Catal..

[cit158] Nørskov J. K., Bligaard T., Rossmeisl J., Christensen C. H. (2009). Nat. Chem..

[cit159] Strmcnik D., Kodama K., van der Vliet D., Greeley J., Stamenkovic V. R., Marković N. M. (2009). Nat. Chem..

[cit160] Xue S., Garlyyev B., Watzele S., Liang Y., Fichtner J., Pohl M. D., Bandarenka A. S. (2018). ChemElectroChem.

[cit161] Liu E., Li J., Jiao L., Doan H. T.-T., Liu Z., Zhao Z., Huang Y., Abraham K. M., Mukerjee S., Jia Q. (2019). J. Am. Chem. Soc..

[cit162] Waegele M. M., Gunathunge C. M., Li J., Li X. (2019). J. Chem. Phys..

[cit163] Suntivich J., Perry E. E., Gasteiger H. A., Shao-Horn Y. (2013). Electrocatal.

[cit164] Tymoczko J., Colic V., Ganassin A., Schuhmann W., Bandarenka A. S. (2015). Catal. Today.

[cit165] Zhu S., Hu X., Zhang L., Shao M. (2016). J. Phys. Chem. C.

[cit166] Mills J. N., McCrum I. T., Janik M. J. (2014). Phys. Chem. Chem. Phys..

[cit167] Magnussen O. M., Groß A. (2019). J. Am. Chem. Soc..

[cit168] Kornyshev A. A. (2007). J. Phys. Chem. B.

[cit169] Bazant M., Storey B. D., Kornyshev A. A. (2011). Phys. Rev. Lett..

[cit170] Damaskin B. B., Petrii O. A. (2011). J. Solid State Electrochem.

[cit171] Kuznetsov A. M. , Charge Transfer in Physics, Chemistry and Biology: Physical Mechanisms of Elementary Processes and an Introduction to the Theory, Gordon and Breach, Amsterdam, 1995

[cit172] Kuznetsov A. M., Ulstrup J. (2000). Electrochim. Acta.

[cit173] Henstridge M. C., Laborda E., Rees N. V., Compton R. G. (2012). Electrochim. Acta.

[cit174] Kim Y.-T., Lopes P. P., Park S.-A., Lee A. Y., Lim J., Back S., Jung Y., Danilovic N., Stamenkovic V., Erlebacher J., Snyder J., Markovic N. M. (2017). Nat. Commun..

[cit175] Saveleva V. A., Wang L., Luo W., Zafeiratos S., Ulhaq-Bouillet C., Gago A. S., Friedrich K. A., Savinova E. R. (2016). J. Phys. Chem. Lett..

[cit176] Hartig-Weiss A., Miller M., Beyer H., Schmitt A., Siebel A., Freiberg A. T.-S., Gasteiger H. A., El-Sayed H. A. (2020). ACS Appl. Nano Mater..

[cit177] Sol.-Hernandez L., Claudel F., Maillard F., Beauger C. (2019). Int. J. Hydrogen Energy.

[cit178] Saveleva V. A., Wang L., Kasian O., Batuk M., Hadermann J., Gallet J.-J., Bournel F., Alonso-Vante N., Ozouf G., Beauger C., Mayrhofer K. J.-J., Cherevko S., Gago A. S., Friedrich K. A., Zafeiratos S., Savinova E. R. (2020). ACS Catal..

[cit179] Böhm D., Beetz M., Schuster M., Peters K., Hufnagel A. G., Döblinger M., Böller B., Bein T., Fattakhova-Rohlfing D. (2020). Adv. Funct. Mater..

[cit180] Abbott D. F., Lebedev D., Waltar K., Povia M., Nachtegaal M., Fabbri E., Copéret C., Schmidt T. J. (2016). Chem. Mater..

[cit181] Oh H. S., Nong H. N., Reier T., Gliech M., Strasser P. (2015). Chem. Sci..

[cit182] Oh H.-S., Nong H. N., Reier H. N. T., Bergmann T. A., Gliech A. M., Ferreira de Araujo J., Willinger E., Schlögl R., Teschner D., Strasser P. (2016). J. Am. Chem. Soc..

[cit183] Binninger T., Schmidt T. J., Kramer D. (2017). Phys. Rev. B.

[cit184] Pacchioni G. (2013). Phys. Chem. Chem. Phys..

[cit185] Pacchioni G., Freund H. J. (2018). Chem. Soc. Rev..

[cit186] Bayrak Pehlivan İ., Arvizu M. A., Qiu Z., Niklasson G. A., Edvinsson T. (2019). J. Phys. Chem. C.

[cit187] Huang J., Zhang J., Eikerling M. (2017). J. Phys. Chem. C.

[cit188] Zhang Y., Yufan J. H., Huang J., Eikerling M. (2021). Electrochim. Acta.

[cit189] Greeley J., Stephens I. E.-L., Bondarenko A. S., Johansson T. P., Hansen H. A., Jaramillo T. F., Rossmeisl J., Chorkendorff I., Nørskov J. K. (2009). Nat. Chem..

[cit190] Greeley J., Jaramillo T. F., Bonde J., Chorkendorff I. B., Norskov J. K. (2006). Nat. Mater..

[cit191] Ma Y., He Z., Wu Z., Zhang B., Zhang Y., Ding S., Xiao C. (2017). J. Mater. Chem. A.

[cit192] He Z. D., Wei J., Chen Y.-X., Santos E., Schmickler W. (2017). Electrochim. Acta.

[cit193] Hansen H. A., Rossmeisl J., Nørskov J. K. (2008). Phys. Chem. Chem. Phys..

[cit194] Gossenberger F., Juarez F., Groß A. (2020). Front. Chem..

[cit195] Eslamibidgoli M. J., Eikerling M. H. (2018). Curr. Opin. Electrochem..

[cit196] Calle-Vallejo F., Koper M. T.-M. (2012). Electrochim. Acta.

[cit197] Groß A. (2021). Curr. Opin. Electrochem..

[cit198] Over H. (2021). ACS Catal..

[cit199] You B., Tang M. T., Tsai C., Abild-Pedersen F., Zheng X., Li H. (2019). Adv. Mater..

[cit200] Buvat G., Eslamibidgoli J. M., Youssef A. H., Garbarino S., Ruediger A., Eikerling M., Guay D. (2019). ACS Catal..

[cit201] Park J., Sa Y. J., Baik H., Kwon T., Joo S. H., Lee K. (2017). ACS Nano.

[cit202] Abidi N., Lim K. R.-G., Seh Z. W., Steinmann S. N. (2021). WIREs Comput Mol Sci..

[cit203] Li Q., Ouyang Y., Lu S., Bai X., Zhang Y., Shi L., Ling C., Wang J. (2020). Chem. Commun..

[cit204] Ringe S., Clark E. L., Resasco J., Walton A., Seger B., Bell A. T., Chan K. (2019). Energy Environ. Sci..

[cit205] Sung S., Forster-Tonigold K., Groß A. (2016). J. Chem. Phys..

[cit206] Rossmeisl J., Skúlason E., Björketun M. E., Tripkovic V., Nørskov J. K. (2008). Chem. Phys. Lett..

[cit207] Chan K., Nørskov J. K. (2015). J. Phys. Chem. Lett..

[cit208] Lozovoi A. Y., Alavi A., Kohanoff J., Lynden-Bell R. M. (2001). J. Chem. Phys..

[cit209] Otani M., Sugino O. (2006). Phys. Rev. B.

[cit210] Otani M., Hamada I., Sugino O., Morikawa Y., Okamoto Y., Ikeshoji T. (2008). Phys. Chem. Chem. Phys..

[cit211] Jinnouchi R., Anderson A. B. (2008). Phys. Rev. B.

[cit212] Sundararaman R., Letchworth-Weaver K., Schwarz K. A., Gunceler D., Ozhabes Y., Arias T. A. (2017). SoftwareX.

[cit213] Letchworth-Weaver K., Arias T. A. (2012). Phys. Rev. B.

[cit214] Schnur S., Groß A. (2009). New J. Phys..

[cit215] Malek A., Eikerling M. H. (2018). Electrocatalysis.

[cit216] Laidler J. (1996). Pure Appl. Chem..

[cit217] Kozuch K. S., Martin J. M.-L. (2011). ChemPhysChem.

[cit218] Nørskov J. K., Bligaard T., Hvolbæk B., Abild-Pedersen F., Chorkendorff I., Christensen C. H. (2008). Chem. Soc. Rev..

[cit219] Koper M. T. M. (2013). J. Solid State Electrochem.

[cit220] Deiman J. R., van Troostwijk A. P., de la Metherie Lettre a M. (1789). J. Phys. Chim. L'hist. Nat..

[cit221] Lilley S. (1948). Ann. Sci..

[cit222] Hall C. M. , Process of reducing aluminium from its fluoride salts by electrolysis, patent, US400664A, 1886

[cit223] Héroult P. L.-T. , Procédé électrolytique pour la préparation de l’aluminium, patent, FR175711, 1886

[cit224] Castner H. Y. , Process of and experimental apparatus for electrolytic decomposition of alkaline salts, patent, FR528322, 1894

[cit225] IRENA, *Green Hydrogen Cost Reduction: Scaling up Electrolysers to Meet the 1*.*5 °C Climate Goal*, International Renewable Energy Agency, Abu Dhabi, 2020, https://www.irena.org/publications/2020/Dec/Green-hydrogen-cost-reduction

[cit226] Bowen C. T., Davis H. J., Henshaw B. F., Lachance R., LeRoy R. L., Renaud R. (1984). Int. J. Hydrogen Energy.

[cit227] Kreuter W., Hofmann H. (1998). Int. J. Hydrog. Energy.

[cit228] Tarnay D. S. (1985). Int. J. Hydrogen Energy.

[cit229] Staffell I., Scamman D., Velazquez Abad A., Balcombe P., Dodds P. E., Ekins P., Shah N., Ward K. R. (2019). Energy Environ. Sci..

[cit230] Millet J.-C. , Cellules d'électrolyse chlore-soude, in Technique de l'ingénieur, Technique de l'ingénieur, Paris, 2008, vol. J 4 804, pp. 1–16

[cit231] Millet P. and Grigoriev S., in Renewable Hydrogen Technologies: Production, Purification, Storage, Applications and Safety, ed. L. M. Gandía, G. Arzamendi and P. M. Diéguez, Elsevier Science, Netherlands, 2013, ch. 2, pp. 19–41

[cit232] Marini S., Salvi P., Nelli P., Pesenti R., Villa M., Berrettoni M., Zangari G., Kiros Y. (2012). Electrochim. Acta.

[cit233] Hall D. E. (1981). J. Electrochem. Soc..

[cit234] Hall D. E. (1982). J. Electrochem. Soc..

[cit235] Hall D. E. (1983). J. Electrochem. Soc..

[cit236] Damien A. , Hydrogène par électrolyse de l’eau, in Technique de l'ingénieur, Technique de l'ingénieur, Paris, 1992, vol. J 6 366, pp. 1–9

[cit237] Brauns J., Schönebeck J., Kraglund M. R., Aili D., Hnát J., Žitka J., Mues W., Jensen J. O., Bouzek K., Turek T. (2021). J. Electrochem. Soc..

[cit238] Haug P., Koj M., Turek T. (2017). Int. J. Hydrogen
Energy.

[cit239] Schillings J., Doche O., Deseure J. (2015). Int. J. Heat Mass Transfer.

[cit240] Brauns J., Turek T. (2020). Processes.

[cit241] Carmo M., Fritz D. L., Mergel J., Stolten D. (2013). Int. J. Hydrogen Energy.

[cit242] Stähler M., Stähler A., Scheepers F., Carmo M., Lehnert W., Stolten D. (2020). Int. J. Hydrogen Energy.

[cit243] Xie X., Chen S., Zhou Y., Hu X. (2020). Int. J. Electrochem. Sci..

[cit244] Kroschel M., Bonakdarpour A., Kwan J. T.-H., Strasser P., Wilkinson D. P. (2019). Electrochim. Acta.

[cit245] Liu C., Shviro M., Gago A. S., Zaccarine S. F., Bender G., Gazdzicki P., Morawietz T., Biswas I., Rasinski M., Everwand A., Schierholz R., Pfeilsticker J., Müller M., Lopes P. P., Eichel R.-A., Pivovar B., Pylypenko S., Friedrich K. A., Lehnert W., Carmo M. (2021). Adv. Energy Mater..

[cit246] Schuler T., Ciccone J. M., Krentscher B., Marone F., Peter C., Schmidt T. J., Büchi F. N. (2020). Adv. Energy Mater..

[cit247] Bühler M., Holzapfel P., McLaughlin D., Thiele S. (2019). J. Electrochem. Soc..

[cit248] Yasutake M., Kawachino D., Noda Z., Matsuda J., Lyth S. M., Ito K., Hayashi A., Sasaki K. (2020). J. Electrochem. Soc..

[cit249] Laguna-Bercero M. A. (2012). J. Power Sources.

[cit250] Bo Y., Wenqiang Z., Jingming X., Jing C. (2010). Int. J. Hydrogen Energy.

[cit251] Cai Q., Haw A. W.-V., Adjiman C. S., Brandon N. P. (2012). Comput. Aided Chem. Eng..

[cit252] Arunkumar P., Uthayakumar A., Subrayan R., Cha S. W., Moorthy S. B.-K. (2019). Nanomater. Energy.

[cit253] Wang R., Dogdibegovic E., Lau G. Y., Tucker M. C. (2019). Energy Technol..

[cit254] Banner J., Akter A., Wang R., Pietras J., Sulekar S., Marina O. A., Gopalan S. (2021). J. Power Sources.

[cit255] Fu Y. S., Poizeau S., Bertei A., Qi C., Mohanram A., Pietras J. D., Bazant M. Z. (2015). Electrochim. Acta.

[cit256] Fu Y., Jiang Y., Poizeau S., Dutta A., Mohanram A., Pietras J. D., Bazant M. Z. (2015). J. Electrochem. Soc..

[cit257] Wang Y., Li W., Ma L., Li W., Liu X. (2020). J. Mater. Sci. & Technol..

[cit258] IEA, 2021, “Net Zero by 2050: A Roadmap for the Global Energy Sector”. International Energy Agency

[cit259] European Commission, 2020. “A hydrogen strategy for a climate-neutral Europe” https://ec.europa.eu/energy/sites/ener/files/hydrogen_strategy.pdf

[cit260] Snieckus D. , Hydrogen electrolyser market booms with '1,000-fold' growth in frame by 2040: Aurora, 2021, https://www.rechargenews.com/energy-transition/hydrogen-electrolyser-market-booms-with-1-000-fold-growth-in-frame-by-2040-aurora/2-1-1009199

[cit261] Woodward E. , Han O. and Forbes C., Enabling the European hydrogen economy, Aurora Energy Research, 2021

[cit262] Huang T., He G., Xue J., Otoo O., He X., Jiang H., Zhang J., Yin Y., Jiang Z., Douglin J. C., Dekel D. R., Guiver M. D. (2020). J. Membr. Sci..

[cit263] Zhou J., Unlu M., Vega J. A., Kohl P. A. (2009). J. Power Sources.

[cit264] Amel A., Gavish N., Zhu L., Dekel D. R., Hickner M. A., Ein-Eli Y. (2016). J. Membr. Sci..

[cit265] Liu Y., Dai J., Zhang K., Ma L., Qaisrani N. A., Zhang F., He G. (2017). Ionics.

[cit266] Li X., Yu Y., Meng Y. (2013). ACS Appl. Mater. Interfaces.

[cit267] Yan X., Gu S., He G., Wu X., Benziger J. (2014). J. Power Sources.

[cit268] Han J., Peng H., Pan J., Wei L., Li G., Chen C., Xiao L., Lu J., Zhuang L. (2013). ACS Appl. Mater. Interfaces.

[cit269] Kumari M., Douglin J. C., Dekel D. R. (2021). J. Membr. Sci..

[cit270] Lin X., Liu Y., Poynton S. D., Ong A. L., Varcoe J. R., Wu L., Li Y., Liang X., Li Q., Xu T. (2013). J. Power Sources.

[cit271] Oh B. H., Kim A. R., Yoo D. J. (2019). Int. J. Hydrogen Energy.

[cit272] Wang G., Weng Y., Chu D., Xie D., Chen R. (2009). J. Membr. Sci..

[cit273] Wang G., Weng Y., Zhao J., Chu D., Xie D., Chen R. (2010). Polym. Adv. Technol..

[cit274] Yadav V., Rajput A., Sharma P. P., Jha P. K., Kulshrestha V. (2020). Colloids Surf., A.

[cit275] Wang W., Wang S., Li W., Xie X., Lu Y. (2013). Int. J. Hydrogen Energy.

[cit276] Hu Q., Shang Y., Wang Y., Xu M., Wang S., Xie X., Liu Y., Zhang H., Wang J., Mao Z. (2012). Int. J. Hydrogen Energy.

[cit277] Liu G., Shang Y., Xie X., Wang S., Wang J., Wang Y., Mao Z. (2012). Int. J. Hydrogen Energy.

[cit278] Willdorf-Cohen S., Mondal A. N., Dekel D. R., Diesendruck C. E. (2018). J. Mater. Chem. A.

[cit279] Lin B., Xu F., Chu F., Ren Y., Ding J., Yan F. (2019). J. Mater. Chem. A.

[cit280] Xue J., Liu X., Zhang J., Yin Y., Guiver M. D. (2020). J. Membr. Sci..

[cit281] Pan J., Han J., Zhu L., Hickner M. A. (2017). Chem. Mater..

[cit282] Zhegur A., Gjineci N., Willdorf-Cohen S., Mondal A. N., Diesendruck C. E., Gavish N., Dekel D. R. (2020). ACS Appl. Polym. Mater..

[cit283] Das G., Park B. J., Kim J., Kang D., Yoon H. H. (2019). Sci. Rep..

[cit284] Janarthanan R., Horan J. L., Caire B. R., Ziegler Z. C., Yang Y., Zuo X., Liberatore M. W., Hibbs M. R., Herring A. M. (2013). J. Polym. Sci., Part B: Polym. Phys..

[cit285] Fujimoto C., Kim D. S., Hibbs M., Wrobleski D., Kim Y. S. (2012). J. Membr. Sci..

[cit286] Liu X., Wu D., Liu X., Luo X., Liu Y., Zhao Q., Li J., Dong D. (2020). Electrochim. Acta.

[cit287] McHugh P. J., Das A. K., Wallace A. G., Kulshrestha V., Shahi V. K., Symes M. D. (2021). Membranes.

[cit288] Chu J. Y., Lee K. H., Kim A. R., Yoo D. J. (2018). Polymers.

[cit289] Vandiver M. A., Horan J. L., Yang Y., Tansey E. T., Seifert S., Liberatore M. W., Herring A. M. (2013). J. Polym. Sci., Part B: Polym. Phys..

[cit290] Bosnjakovic A., Danilczuk M., Schlick S., Xiong P. N., Haugen G. M., Hamrock S. J. (2014). J. Membr. Sci..

[cit291] Biancolli A. L.-G., Bsoul-Haj S., Douglin J. C., Barbosa A. S., de Sousa R. R., Rodrigues O., Lanfredi A. J.-C., Dekel D. R., Santiago E. I. (2022). J. Membr. Sci..

[cit292] Henkensmeier D., Cho H., Brela M., Michalak A., Dyck A., Germer W., Duong N. M.-H., Jang J. H., Kim H. J., Woo N. S., Lim T. H. (2014). Int. J. Hydrogen Energy.

[cit293] Lee H. J., Choi J., Han J. Y., Kim H. J., Sung Y. E., Kim H., Henkensmeier D., Ae Cho E., Jang J. H., Yoo S. J. (2013). Polym. Bull..

[cit294] Thomas O. D., Soo K. J.-W. Y., Peckham T. J., Kulkarni M. P., Holdcroft S. (2012). J. Am. Chem. Soc..

[cit295] Thomas O. D., Soo K. J.-W. Y., Peckham T. J., Kulkarni M. P., Holdcroft S. (2011). Polym. Chem..

[cit296] Fan J., Willdorf-Cohen S., Schibli E. M., Paula Z., Li W., Skalski T. J.-G., Sergeenko A. T., Hohenadel A., Frisken B. J., Magliocca E., Mustain W. E., Diesendruck C. E., Dekel D. R., Holdcroft S. (2019). Nat. Commun..

[cit297] Zheng Y., Ash U., Pandey R. P., Ozioko A. G., Ponce-González J., Handl M., Weissbach T., Varcoe J. R., Holdcroft S., Liberatore M. W., Hiesgen R., Dekel D. R. (2018). Macromolecules.

[cit298] Lee B., Yun D., Lee J. S., Park C. H., Kim T. H. (2019). J. Phys. Chem. C.

[cit299] Zhang M., Kim H. K., Chalkova E., Mark F., Lvov S. N., Chung T. C.-M. (2011). Macromolecules.

[cit300] Kostalic H. A., Clark T. J., Robertson N. J., Mutolo P. F., Longo J. M., Abruña H. D., Coates G. W. (2010). Macromolecules.

[cit301] Robertson N. J., Kostalik H. A., Clark T. J., Mutolo P. F., Abruna H. D., Coates G. W. (2010). J. Am. Chem. Soc..

[cit302] Zhegur-Khais A., Kubannek F., Krewer U., Dekel D. R. (2020). J. Membr. Sci..

[cit303] Douglin J. C., Varcoe J. R., Dekel D. R. (2020). J. Power Sources Adv..

[cit304] Buggy N. C., Du Y. F., Kuo M. C., Ahrens K. A., Wilkinson J. S., Seifert S., Coughlin E. B., Herring A. M. (2020). ACS Appl. Polym. Mater..

[cit305] Gupta G., Scott K., Mamlouk M. (2018). J. Power Sources.

[cit306] Biancolli A. L.-G., Barbosa A. S., Kodama Y., de Sousa R. R., Lanfredi A. J.-C., Fonseca F. C., Rey J. F.-Q., Santiago E. I. (2021). J. Power Sources.

[cit307] Li D., Park E. J., Zhu W., Shi Q., Zhou Y., Tian H., Lin Y., Serov A., Zulevi B., Baca E. D., Fujimoto C., Chung H. T., Kim Y. S. (2020). Nat. Energy.

[cit308] Tsai T. H., Maes A. M., Vandiver M. A., Versek C., Seifert S., Tuominen M., Liberatore M. W., Herring A. M., Coughlin E. B. (2013). J. Polym. Sci., Part B: Polym. Phys..

[cit309] Zhou J., Guo J., Chu D., Chen R. (2012). J. Power Sources.

[cit310] Vinodh R., Ilakkiya A., Elamathi S., Sangeetha D. (2010). Mater. Sci. Eng., B.

[cit311] Dekel D. R. (2013). ECS Trans..

[cit312] Amel A., Smedley S. B., Dekel D. R., Hickner M. A., Ein-Eli Y. (2015). J. Electrochem. Soc..

[cit313] Liu Y., Zhou J., Hou J., Yang Z., Xu T. (2019). ACS Appl. Polym. Mater..

[cit314] Dekel D. R. , Alkaline Membrane Fuel Cells, Encyclopedia of Applied Electrochemistry, Springer New York, NY, 2014 10.1007/978-1-4419-6996-5

[cit315] Agel E., Bouet J., Fauvarque J. F. (2001). J. Power Sources.

[cit316] Mohanty A. D., Bae C. (2014). J. Mater. Chem. A.

[cit317] Marino M. G., Kreuer K. D. (2015). ChemSusChem.

[cit318] Katzfuß A., Gogel V., Jörissen L., Kerres J. (2013). J. Membr. Sci..

[cit319] Ko B. S., Sohn J. Y., Shin J. (2012). Polymer.

[cit320] Hugar K. M., Kostalik H. A., Coates G. W. (2015). J. Am. Chem. Soc..

[cit321] Gjineci N., Aharonovich S., Dekel D. R., Diesendruck C. E. (2020). ACS Appl. Mater. Interfaces.

[cit322] Gjineci N., Aharonovich S., Willdorf-Cohen S., Dekel D. R., Diesendruck C. E. (2020). Eur. J. Org. Chem..

[cit323] Wang J., Zhao Y., Setzler B. P., Rojas-Carbonell S., Ben Yehuda C., Amel A., Page M., Wang L., Hu K., Shi L., Gottesfeld S., Xu B., Yan Y. (2019). Nat. Energy.

[cit324] Liu Y., Wang J., Yang Y., Brenner T. M., Seifert S., Yan Y., Liberatore M. W., Herring A. M. (2014). J. Phys. Chem. C.

[cit325] Yan X., Gu S., He G., Wu X., Zheng W., Ruan X. (2014). J. Membr. Sci..

[cit326] Gu S., Skovgard J., Yan Y. S. (2012). ChemSusChem.

[cit327] Noonan K. J.-T., Hugar K. M., Kostalik H. A., Lobkovsky E. B., Abruña H. D., Coates G. W. (2012). J. Am. Chem. Soc..

[cit328] Kong X., Wadhwa K., Verkade J. G., Schmidt-Rohr K. (2009). Macromolecules..

[cit329] Jang H., Hossain M. A., Sutradhar S. C., Ahmed F., Choi K., Ryu T., Kim K., Kim W. (2017). Int. J. Hydrogen Energy.

[cit330] Zhang B., Gu S., Wang J., Liu Y., Herring A. M., Yan Y. (2012). RSC Adv..

[cit331] Hossain M. A., Jang H., Sutradhar S. C., Ha J., Yoo J., Lee C., Lee S., Kim W. (2016). Int. J. Hydrogen Energy.

[cit332] Disabb-Miller M. L., Zha Y., DeCarlo A. J., Pawar M., Tew G. N., Hickner M. A. (2013). Macromolecules.

[cit333] Zha Y., Disabb-Miller M. L., Johnson Z. D., Hickner M. A., Tew G. N. (2012). J. Am. Chem. Soc..

[cit334] Chen N., Zhu H., Chu Y., Li R., Liu Y., Wang F. (2017). Polym. Chem..

[cit335] Zhu T., Xu S., Rahman A., Dogdibegovic E., Yang P., Pageni P., Kabir M. P., Zhou X. D., Tang C. (2018). Angew. Chem., Int. Ed..

[cit336] Wang Y., Rapakousiou A., Astruc D. (2014). Macromolecules.

[cit337] Zhu T., Sha Y., Firouzjaie H. A., Peng X., Cha Y., Dissanayake D., Smith M. D., Vannucci A. K., Mustain W. E., Tang C. (2020). J. Am. Chem. Soc..

[cit338] Gu S., Wang J., Kaspar R. B., Fang Q., Zhang B., Coughlin E. B., Yan Y. (2015). Sci. Rep..

[cit339] Liu X., Xie N., Xue J., Li M., Zheng C., Zhang J., Qin Y., Yin Y., Dekel D. R., Guiver M. D. (2022). Nat. Energy.

[cit340] Xu Y., Zhao C. H., Liu Q. L. (2013). J. Appl. Polym. Sci..

[cit341] Kwasny M. T., Zhu L., Hickner M. A., Tew G. N. (2018). J. Polym. Sci., Part A: Polym. Chem..

[cit342] Aggarwal K., Bsoul S., Li S., Dekel D. R., Diesendruck C. E. (2021). Macromol. Rapid Commun..

[cit343] Aggarwal K., Bsoul S., Douglin J. C., Dekel D. R., Diesendruck C. E. (2022). Chem. – Eur. J..

[cit344] Lin X., Wu L., Liu Y., Ong A. L., Poynton S. D., Varcoe J. R., Xu T. (2012). J. Power Sources.

[cit345] Zhang Q., Li S., Zhang S. (2010). Chem. Commun..

[cit346] Xue B., Dong X., Li Y., Zheng J., Li S., Zhang S. (2017). J. Membr. Sci..

[cit347] Kim D. S., Fujimoto C. H., Hibbs M. R., Labouriau A., Choe Y. K., Kim Y. S. (2013). Macromolecules.

[cit348] Zhu Y., Ding L., Liang X., Shehzad M. A., Wang L., Ge X., He Y., Wu L., Varcoe J. R., Xu T. (2018). Energy Environ. Sci..

[cit349] Hu M., Ding L., Shehzad M. A., Ge Q., Liu Y., Yang Z., Wu L., Xu T. (2019). J. Membr. Sci..

[cit350] Zhu L., Yu X., Hickner M. A. (2018). J. Power Sources.

[cit351] Kwasny M. T., Tew G. N. (2017). J. Mater. Chem. A.

[cit352] Yang Q., Li L., Lin C. X., Gao X. L., Zhao C. H., Zhang Q. G., Zhu A. M., Liu Q. L. (2018). J. Membr. Sci..

[cit353] Ge Q., Liu Y., Yang Z., Wu B., Hu M., Liu X., Hou J., Xu T. (2016). Chem. Commun..

[cit354] Merle G., Wessling M., Nijmeijer K. (2011). J. Membr. Sci..

[cit355] You W., Noonan K. J.-T., Coates G. W. (2020). Prog. Polym. Sci..

[cit356] Duan Q., Ge S., Wang C. Y. (2013). J. Power Sources.

[cit357] Wang L., Peng X., Mustain W. E., Varcoe J. R. (2019). Energy Environ. Sci..

[cit358] Shang C., Wang Z., Wang L., Wang J. (2020). Int. J. Hydrogen Energy.

[cit359] Douglin J. C., Singh R. K., Haj S., Li S., Biemolt J., Yan N., Varcoe J. R., Rothenberg G., Dekel D. (2021). Chem. Eng. J. Adv..

[cit360] Yassin K., Rasin I. G., Willdorf-Cohen S., Diesendruck C. E., Brandon S., Dekel D. R. (2021). J. Power Sources Adv..

[cit361] Dekel D. R. (2018). J. Power Sources.

[cit362] Leng Y., Chen G., Mendoza A. J., Tighe T. B., Hickner M. A., Wang C. Y. (2012). J. Am. Chem. Soc..

[cit363] Aili D., Hansen M. K., Renzaho R. F., Li Q. F., Christensen E., Jensen J. O., Bjerrum N. J. (2013). J. Membr. Sci..

[cit364] Pavel C. C., Cecconi F., Emiliani C., Santiccioli S., Scaffidi A., Catanorchi S., Comotti M. (2014). Angew. Chem., Int. Ed..

[cit365] Park J. E., Kim M. J., Lim M. S., Kang S. Y., Kim J. K., Oh S. H., Her M., Cho Y. H., Sung Y. E. (2018). Appl. Catal., B.

[cit366] Kraglund M. R., Carmo M., Schiller G., Ansar S. A., Aili D., Christensen E., Jensen J. O. (2019). Energy Environ. Sci..

[cit367] Cha M. S., Park J. E., Kim S., Han S.-H., Shin S.-H., Yang S. H., Kim T.-H., Yu D. M., So S., Hong Y. T. (2020). et al.. Energy Environ. Sci..

[cit368] Liu Z., Sajjad S. D., Gao Y., Yang H., Kaczur J. J., Masel R. I. (2017). Int. J. Hydrogen Energy.

[cit369] Liu J., Kang Z., Li D., Pak M., Alia S. M., Fujimoto C., Bender G., Kim Y. S., Weber A. Z. (2021). J. Electrochem. Soc..

[cit370] Henkensmeier D., Najibah M., Harms C., Zitka J., Hnat J., Bouzek K. (2021). J. Electrochem. Energy Convers. Storage.

[cit371] Dioxide Materials, *Anion Exchange Membranes*, 2021. https://dioxidematerials.com/products/anion-exchange-membranes

[cit372] Motealleh B., Liu Z., Masel R. I., Sculley J. P., Richard Ni Z., Meroueh L. (2021). Int. J. Hydrogen Energy.

[cit373] Li D., Motz A. R., Bae C., Fujimoto C., Yang G., Zhang F. Y., Ayers K. E., Kim Y. S. (2021). Energy Environ. Sci..

[cit374] Ito H., Miyazaki N., Ishida M., Nakano A. (2016). Int. J. Microgravity Sci. Appl..

[cit375] Ito H., Kawaguchi N., Someya S., Munakata T. (2019). Electrochim. Acta.

[cit376] Park H. J., Chu X., Kim S. P., Choi D., Jung J. W., Woo J., Baek S. Y., Yoo S. J., Chung Y. C., Seong J. G., Lee S. Y., Li N., Lee Y. M. (2020). J. Membr. Sci..

[cit377] Smith K. C., Dmello R., Soc J. E., Soc M. J.-E., Smith K. C., Dmello R. (2016). Porous-Electrode.

[cit378] Gottesfeld S., Dekel D. R., Page M., Bae C., Yan Y., Zelenay P., Kim Y. S. (2018). J. Power Sources.

[cit379] Arges C. G., Zhang L. (2018). ACS Appl. Energy Mater..

[cit380] Weissbach T., Wright A. G., Peckham T. J., Sadeghi Alavijeh A., Pan V., Kjeang E., Holdcroft S. (2016). Chem. Mater..

[cit381] Zhang J., Zhu W., Huang T., Zheng C., Pei Y., Shen G., Nie Z., Xiao D., Yin Y., Guiver M. D. (2021). Sci. Adv..

[cit382] You W., Padgett E., MacMillan S. N., Muller D. A., Coates G. W. (2019). PNAS.

[cit383] Dekel D. R., Amar M., Willdorf S., Kosa M., Dhara S., Diesendruck C. E. (2017). Chem. Mater..

[cit384] Müller J., Zhegur A., Krewer U., Varcoe J. R., Dekel D. R. (2020). ACS Mater. Lett..

[cit385] Choe Y. K., Fujimoto C., Lee K. S., Dalton L. T., Ayers K., Henson N. J., Kim Y. S. (2014). Chem. Mater..

[cit386] Arges C. G., Wang L., Parrondo J., Ramani V. (2013). J. Electrochem. Soc..

[cit387] Pham T. H., Olsson J. S., Jannasch P. (2018). J. Mater. Chem. A.

[cit388] Arges C. G., Ramani V. (2012). PNAS.

[cit389] Miyanishi S., Yamaguchi T. (2016). Phys. Chem. Chem. Phys..

[cit390] Wierzbicki S., Douglin J. C., Kostuch A., Dekel D. R., Kruczała K. (2020). J. Phys. Chem. Lett..

[cit391] Parrondo J., Wang Z., Jung M. S.-J., Ramani V. (2016). Phys. Chem. Chem. Phys..

[cit392] Espiritu R., Mamlouk M., Scott K. (2016). Int. J. Hydrogen Energy.

[cit393] https://www.platinum.matthey.com/prices/price-charts

[cit394] Vesborg P. C.-K., Jaramillo T. F. (2020). RSC Adv..

[cit395] Minke C., Suermann M., Bensmann B., Hanke-Rauschebach R. (2021). Int. J. Hydrogen Energy.

[cit396] Pollet B. G., Staffell I., Adamson K. A. (2015). Int. J. Hydrogen Energy.

[cit397] Ji H.-I., Lee J.-H., Son J.-W., Yoon K. J., Yang S., Kim B.-K. (2020). J. Korean Ceram. Soc..

[cit398] Iwahara H., Esaka T., Uchida H., Maeda N. (1981). Solid State Ionics.

[cit399] Choi S., Davenport T. C., Haile S. M. (2019). Energy Environ. Sci..

[cit400] Duan C., Kee R., Zhu H., Sullivan N., Zhu L., Bian L., Jennings D., O’Hayre R. (2019). Nat. Energy.

[cit401] Medvedev D. (2019). Int. J. Hydrogen Energy.

[cit402] Ghezel-Ayagh H. , Sullivan N. and O’Hayre R., *Proton-Conducting Ceramic Electrolyzers for High-Temperature Water Splitting*, 2019. https://www.hydrogen.energy.gov/pdfs/review19/p177_ghezel-ayagh_2019_p.pdf

[cit403] Huynh M., Ozel T., Liu C., Lau E. C., Nocera D. G. (2017). Chem. Sci..

[cit404] Hu C., Zhang L., Gong J. (2019). Energy Environ. Sci..

[cit405] Wang S., Lu A., Zhong C.-J. (2021). Nano Convergence.

[cit406] Dubouis N., Grimaud A. (2019). Chemical Sci..

[cit407] Zeng M., Li Y. (2015). J. Mater. Chem. A.

[cit408] Medford A. J., Vojvodic A., Hummelshøj J. S., Voss J., Abild-Pedersen F., Studt F., Bligaard T., Nilsson A., Nørskov J. K. (2015). J. Catal..

[cit409] Gutić S. J., Dobrota A. S., Fako E., Skorodumova N. V., López N., Pašti I. A. (2020). Catalysts.

[cit410] Quaino P., Juarez F., Santos E., Schmickler W. (2014). Beilstein J. Nanotechnol..

[cit411] Marković N., Grgur B., Ross P. N. (1997). J. Phys. Chem. B.

[cit412] Marković N. M., Sarraf S. T., Gasteiger H. A., Ross P. N. (1996). Faraday Trans..

[cit413] Shinagawa T., Garcia-Esparza A. T., Takanabe K. (2015). Sci Rep..

[cit414] Conway B., Bai L. (1986). J. Electroanal. Chem. Interfacial Electrochem..

[cit415] Xie L., Liu Q., Shi X., Asiri A. M., Luo Y., Sun X. (2018). Inorg. Chem. Front..

[cit416] Mahmood J., Li F., Jung S. M., Okyay M. S., Ahmad I., Kim S. J., Park N., Jeong H. Y., Baek J. B. (2017). Nat. Nanotechnol..

[cit417] Strmcnik D., Uchimura M., Wang C., Subbaraman R., Danilovic N., van der Vliet D., Paulikas A. P., Stamenkovic V. R., Markovic N. M. (2013). Nat. Chem..

[cit418] Danilovic N., Subbaraman R., Strmcnik D., Chang K. C., Paulikas A. P., Stamenkovic V. R., Markovic N. M. (2012). Angew. Chem., Int. Ed..

[cit419] Chen Z., Duan X., Wei W., Wang S., Ni B.-J. (2020). Nano Energy.

[cit420] Xie Y., Cai J., Wu Y., Zang Y., Zheng X., Ye J., Cui P., Niu S., Liu Y., Zhu J., Liu X., Wang G., Qian Y. (2019). Adv. Mater..

[cit421] Huang Z.-F., Wang J., Peng Y., Jung C.-Y., Fisher A., Wang X. (2017). Adv. Energy Mater..

[cit422] Willsau J., Wolter O., Heitbaum J. (1985). J. Electroanal. Chem. Interfacial Electrochem..

[cit423] Damjanovic A., Birss V., Boudreaux D. (1991). J. Electrochem. Soc..

[cit424] Reier T., Oezaslan M., Strasser P. (2012). ACS Catal..

[cit425] Damjanovic A. (1992). Electrochim. Acta.

[cit426] Vetter K., Schultze J. (1972). J. Electroanal. Chem. Interfacial Electrochem..

[cit427] Bizzotto F., Ouhbi H., Fu Y., Wiberg G. K.-H., Aschauer U., Arenz M. (2019). ChemPhysChem.

[cit428] Lopes P. P., Strmcnik D., Tripkovic D., Connell J. G., Stamenkovic V., Markovic N. M. (2016). ACS Catal..

[cit429] Wu G., Zheng X., Cui P., Jiang H., Wang X., Qu Y., Chen W., Lin Y., Li H., Han X., Hu Y., Liu P., Zhang Q., Ge J., Yao Y., Sun R., Wu Y., Gu L., Hong X., Li Y. (2019). Nat. Commun..

[cit430] Zhu J., Xue Q., Xue Y. Y., Ding Y., Li F. M., Jin P. J., Chen P., Chen Y. (2020). ACS Appl. Mater. Interfaces.

[cit431] Fu L., Zeng X., Huang C., Cai P., Cheng G., Luo W. (2018). Inorg. Chem. Front..

[cit432] Arminio-Ravelo J. A., Quinson J., Pedersen M. A., Kirkensgaard J. J.-K., Arenz M., Escudero-Escribano M. (2020). ChemCatChem.

[cit433] Jiang B., Wang T., Cheng Y., Liao F., Wu K., Shao M. (2018). ACS Appl. Mater. Interfaces.

[cit434] Roy S. B., Akbar K., Jeon J. H., Jerng S.-K., Truong L., Kim K., Yi Y., Chun S.-H. (2019). J. Mater. Chem. A.

[cit435] Zhang J., Wang G., Liao Z., Zhang P., Wang F., Zhuang X., Zschech E., Feng X. (2017). Nano Energy.

[cit436] Xue Q., Gao W., Zhu J., Peng R., Xu Q., Chen P., Chen Y. (2018). J. Colloid Interface Sci..

[cit437] Boshnakova I., Lefterova E., Slavcheva E. (2018). Int. J. Hydrogen Energy.

[cit438] Faria L. A.-D., Boodts J. F.-C., Trasatti S. (1996). J. Appl. Electrochem..

[cit439] Silva L. A.-D., Alves V. A., Trasatti S., Boodts J. F.-C. (1997). J. Electroanal. Chem..

[cit440] Sun W., Wang Z., Zhou Z., Wu Y., Zaman W. Q., Tariq M., Cao L.-M., Gong X.-Q., Yang J. (2019). Chem. Commun..

[cit441] Ozouf G., Cognard G., Maillard F., Chatenet M., Guétaz L., Heitzmann M., Jacques P. A., Beauger C. (2018). J. Electrochem. Soc..

[cit442] Cognard G., Ozouf G., Beauger C., Berthomé G., Riassetto D., Dubau L., Chattot R., Chatenet M., Maillard F. (2017). Appl. Catal., B.

[cit443] Cognard G., Ozouf G., Beauger C., Dubau L., López-Haro M., Chatenet M., Maillard F. (2017). Electrochim. Acta.

[cit444] Cognard G., Ozouf G., Beauger C., Jiménez-Morales I., Cavaliere S., Jones D., Rozière J., Chatenet M., Maillard F. (2017). Electrocatalysis.

[cit445] Claudel F., Dubau L., Berthomé G., Sola-Hernandez L., Beauger C., Piccolo L., Maillard F. (2019). ACS Catal..

[cit446] Suen N. T., Hung S. F., Quan Q., Zhang N., Xu Y. J., Chen H. M. (2017). Chem. Soc. Rev..

[cit447] Chen D., Chen C., Baiyee Z. M., Shao Z., Ciucci F. (2015). Chem. Rev..

[cit448] Roger I., Shipman M., Symes M. (2017). Nat. Rev. Chem..

[cit449] Hunter B. M., Gray H. B., Muller A. M. (2016). Chem. Rev..

[cit450] Blakemore J. D., Crabtree R. H., Brudvig G. W. (2015). Chem. Rev..

[cit451] Gao J., Tao H., Liu B. (2021). Adv. Mater..

[cit452] Conway B. E. and Tilak B. V., in Advances in Catalysis, ed. D. D. Eley, H. Pines and P. B. Weisz, Academic Press, New York, 1992, vol. 38, p. 18

[cit453] Bockris J. O. 'M., Otagawa T. (1983). J. Phys. Chem..

[cit454] Bockris J. O. 'M., Otagawa T. (1984). J. Electrochem. Soc..

[cit455] Rong X., Parolin J., Kolpak A. M. (2016). ACS Catal..

[cit456] Bai L., Lee S., Hu X. (2021). Angew. Chem., Int. Ed..

[cit457] Beer H. B. (1965). Pat. Engl..

[cit458] Cattarin S., Musiani M. (2007). Electrochim. Acta.

[cit459] Mohammadi N., Yari M., Allahkaram S. R. (2013). Surf. Coat. Technol..

[cit460] Li X., Pletcher D., Walsh F. C. (2011). Chem. Soc. Rev..

[cit461] Jones P., Lind R., Wynne-Jones W. F.-K. (1954). Trans. Faraday Soc..

[cit462] Koch D. F.-A. (1959). Aust. J. Chem..

[cit463] Pavlov D., Rogachev T. (1978). Electrochim. Acta.

[cit464] Kötz E. R., Stucki S. (1987). J. Electroanal. Chem..

[cit465] Dimitrov M. K. (1990). J. Power Sources.

[cit466] Rogachev T. (1988). J. Power Sources.

[cit467] Astachov I. I., Weisberg E. S., Kabanov B. N. (1964). Dokl. Acad. Nauk..

[cit468] Foller P. C., Goodwin M. L. (1984). Ozone: Sci. Eng..

[cit469] Velichenko A. B., Girenko D. V., Kovalyov S. V., Gnatenko A. N., Amadelli R., Danilov F. I. (1998). J. Electroanal. Chem..

[cit470] Babak A. A., Amadelli R., De Battisti A., Fateev V. N. (1994). Electrochim. Acta.

[cit471] Amadelli R., Armelao L., Velichenko A. B., Nikolenko N. V., Girenko D. V., Kovalyov S. V., Danilov F. I. (1999). Electrochim. Acta.

[cit472] Ho J. C.-K., Tremiliosi Filho G., Simpraga R., Conway B. E. (1994). J. Electroanal. Chem..

[cit473] Pavlov D., Monahov B. (1998). J. Electrochem. Soc..

[cit474] Pavlov D., Monahov B. (1996). J. Electrochem. Soc..

[cit475] Velichenko A. B., Amadelli R., Baranova E. A., Girenko D. V., Danilov F. I. (2002). J. Electroanal. Chem..

[cit476] Polcaro A. M., Palmas S., Renoldi F., Mascia M. (1999). J. Appl. Electrochem..

[cit477] Zhou M. H., Dai Q. Z., Lei L. C., Ma C., Wang D. H. (2005). Environ. Sci. Technol..

[cit478] Wei G.-L., Wang J.-R. (1994). J. Power Sources.

[cit479] Monashov B., Pavlov D., Kirchev A., Vasilev S. (2003). J. Power Sources.

[cit480] Duan X. Y., Li J. R., Chang L. M., Yang C. W. (2016). J. Water Reuse Desalin..

[cit481] Zhao G., Zhang Y., Lei Y., Lv B., Gao J., Zhang Y., Li D. (2010). Environ. Sci. Technol..

[cit482] Shmychkova O., Luk’yanenko T., Velichenko A., Meda L., Amadelli R. (2013). Electrochim. Acta.

[cit483] Cattarin S., Frateur I., Guerriero P., Musiani M. (2000). Electrochim. Acta.

[cit484] Li Y., Jiang L., Liu F., Li J., Liu Y. (2014). RSC Adv..

[cit485] Abaci S., Pekmez K., Yildiz A. (2005). Electrochem. Commun..

[cit486] Cao J., Zhao H., Cao F., Zhang J. (2007). Electrochim. Acta.

[cit487] Shmychkova O., Luk’yanenko T., Amadelli R., Velichenko A. (2013). J. Electroanal. Chem..

[cit488] Chen C., Wang X., Xu R., Zhang Y., Fang S., Ju A., Jiang W. (2021). RSC Adv..

[cit489] Pourbaix M. , Atlas of electrochemical Equilibria in Aqueous Solutions, Pergamon Press, Bristol, 1966

[cit490] Roche I., Chainet E., Chatenet M., Vondrak J. (2007). J. Phys. Chem. C.

[cit491] Garcia A. C., Herrera A. D., Ticianelli E. A., Chatenet M., Poinsignon C. (2011). J. Electrochem. Soc..

[cit492] Garcia A. C., Lima F. H. B., Ticianelli E. A., Chatenet M. (2013). J. Power Sources.

[cit493] Moureaux F., Stevens P., Chatenet M. (2013). Electrocatalysis.

[cit494] Meng Y., Song W., Huang H., Ren Z., Chen S.-Y., Suib S. L. (2014). J. Am. Chem. Soc..

[cit495] Selvakumar K., Senthil Kumar S. M., Thangamuthu R., Kruthika G., Murugan P. (2014). Int. J. Hydrogen Energy.

[cit496] Cao Y. L., Yang H. X., Ai X. P., Xiao L. F. (2003). J. Electroanal. Chem..

[cit497] Benbow E. M., Kelly S. P., Zhao L., Reutenauer J. W., Suib S. L. (2011). J. Phys. Chem. C.

[cit498] Kokhanov G. N., Anganova R. A., Milova N. G. (1972). Elektrokhimiya.

[cit499] Shembel E. M., Kalinovskii E. A., Moskalevich V. L., Mazur O. M., Artamonov V. G. (1972). Elektrokhimiya.

[cit500] Morita M., Iwakura C., Tamura H. (1977). Electrochim. Acta.

[cit501] Morita M., Iwakura C., Tamura H. (1978). Electrochim. Acta.

[cit502] Morita M., Iwakura C., Tamura H. (1979). Electrochim. Acta.

[cit503] Bennett J. E. (1980). Int. J. Hydrog. Energy.

[cit504] Jiao F., Frei H. (2010). Chem. Commun..

[cit505] Gorlin Y., Jaramillo T. F. (2010). J. Am. Chem. Soc..

[cit506] Fekete M., Hocking R. K., Chang S. L.-Y., Italiano C., Patti A. F., Arena F., Spiccia L. (2013). Energy Environ. Sci..

[cit507] Frydendal R., Paoli E. A., Chorkendorff I., Rossmeisl J., Stephens I. E.-L. (2015). Adv. Energy. Mater..

[cit508] Kim J., Kim J. S., Baik H., Kang K., Lee K. (2016). RSC Adv..

[cit509] Zheng X., Yu L., Lan B., Cheng G., Lin T., He B., Ye W., Sun M., Ye F. (2017). J. Power Sources.

[cit510] Ye Z., Li T., Ma G., Dong Y., Zhou X. (2017). Adv. Funct. Mater..

[cit511] Nakayama M., Tanaka A., Sato Y., Tonosaki T., Ogura K. (2005). Langmuir.

[cit512] Vondrak J., Klapste B., Velicka J., Sedlarikova M., Reiter J., Roche I., Chainet E., Fauvarque J. F., Chatenet M. (2005). J. New Mater. Electrochem. Syst..

[cit513] Tripkovic V., Hansen H. A., Vegge T. (2018). ChemSusChem.

[cit514] Fang M., Han D., Xu W.-B., Shen Y., Lu Y., Cao P., Han S., Xu W., Zhu D., Liu W., Ho J. C. (2020). Adv. Energy Mater..

[cit515] Perdew J. P., Burke K., Ernzerhof M. (1996). Phys. Rev. Lett..

[cit516] Chan Z. M., Kitchaev D. A., Weker J. N., Schnedermann C., Lim K., Ceder G., Tumas W., Toney M. F., Nocera D. G. (2018). PNAS.

[cit517] Gupta P. K., Bhandari A., Saha S., Bhattacharya J., Pala R. G.-S. (2019). J. Phys. Chem. C.

[cit518] Takashima T., Hashimoto K., Nakamura R. (2012). J. Am. Chem. Soc..

[cit519] Li Y.-F., Liu Z.-P. (2018). J. Am. Chem. Soc..

[cit520] Tao H. B., Fang L., Chen J., Yang H. B., Gao J., Miao J., Chen S., Liu B. (2016). J. Am. Chem. Soc..

[cit521] Tompsett D. A., Parker S. C., Islam M. S. (2014). J. Mater. Chem. A.

[cit522] Boppana V. B.-R., Jiao F. (2011). Chem. Commun..

[cit523] Heese-Gärtlein J., Morales D. M., Rabe A., Bredow T., Schuhmann W., Behrens M. (2020). Chem. – Eur. J..

[cit524] Katrib A., Leflaive P., Hilaire L. (1996). Catal. Lett..

[cit525] Jin Y., Shen P. K. (2015). J. Mater. Chem. A.

[cit526] Li B. B., Liang Y. Q., Yang X. J., Cui Z. D., Qiao S. Z., Zhu S. L., Li Z. Y., Yin K. (2015). Nanoscale.

[cit527] Tang Y., Gao M., Liu C., Li S., Jiang H., Lan Y., Han M., Yu S. (2015). Angew. Chem., Int. Ed..

[cit528] Li X., Yu J., Jia J., Wang A., Zhao L., Xiong T., Liu H., Zhou W. (2019). Nano Energy.

[cit529] Jin Y., Wang H., Li J., Yue X., Han Y., Shen P. K., Cui Y. (2016). Adv. Mater..

[cit530] Wang B., Zhang Z., Zhang S., Cao Y., Su Y., Liu S., Tang W., Yu J., Ou Y., Xie S., Li J., Ma M. (2020). Electrochim. Acta.

[cit531] Guha P., Mohanty B., Thapa R., Kadam R. M., Satyam P. V., Jena B. K. (2020). ACS Appl. Energy Mater..

[cit532] Carp O., Huisman C. L., Reller A. (2004). Prog. Solid State Chem..

[cit533] Hoffmann M. R., Martin S. T., Choi W., Bahnemann D. W. (1995). Chem. Rev..

[cit534] Song Y., Roy P., Paramasivamm I., Schmucki P. (2010). Angew. Chem., Int. Ed..

[cit535] Kolbrecka K., Przyluski J. (1994). Electrochim. Acta.

[cit536] Walsh F. C., Wills R. G.-A. (2010). Electrochim.
Acta.

[cit537] Kim C., Kim S., Choi J., Lee J., Kang J. S., Sung Y.-E., Lee J., Choi W., Yoon J. (2014). Electrochim. Acta.

[cit538] Cai L., Cho I. S., Logar M., Mehta A., He J., Lee C. H., Rao P. M., Feng Y., Wilcox J., Prinz F. B., Zheng X. (2014). Phys. Chem. Chem. Phys..

[cit539] Ang Y., Kao L. C., Liu Y., Sun K., Yu H., Guo J., Liou S. Y.-H., Hoffmamm M. R. (2018). ACS Catal..

[cit540] Miles M. H., Huang Y. H., Srinivasan S. (1978). J. Electrochem. Soc..

[cit541] Alves V. A., Da Silva L. A., Boodts J. F.-C. (1998). Electrochim. Acta.

[cit542] Li N., Xia W. Y., Wang J., Liu Z. L., Li Q. Y., Chen S. Z., Xu C. W., Lu X. H. (2015). J. Mater. Chem. A.

[cit543] Elangovan E., Ramamurthi K. (2005). Appl. Surf. Sci..

[cit544] Zhang H.-X., Feng C., Zhai Y.-C., Jiang K.-L., Li Q.-Q., Fan S.-S. (2009). Adv. Mater..

[cit545] Spasov D. D., Ivanova N. A., Pushkarev A. S., Pushkareva I. V., Presnyakova N. N., Chumakov R. G. (2019). Catalysts.

[cit546] Jiang L., Sun G., Zhou Z., Sun S., Wang Q., Yan S. (2005). J. Phys. Chem. B.

[cit547] Salavati-Niasari M., Ghiyasiyan-Arani M. (2021). Inorg. Chem. Front..

[cit548] Bi H., Zuo X., Liu B., He D., Bai L., Wang W., Li X., Xiao Z., Sun K., Song Q., Zang Z., Chen J. (2021). J. Mater. Chem. A.

[cit549] Lee M.-Y., Han S., Lim H., Kwon Y., Kang S. (2020). ACS Sustainable Chem. Eng..

[cit550] Sinclair W. R., Peters F. G., Stilling D. W., Koonce S. E. (1965). J. Electrochem. Soc..

[cit551] Shanthi E., Dutta V., Banerjee A., Chopra K. L. (1980). J. Appl. Phys..

[cit552] Kane J., Schweizer H. P., Kern W. (1976). J. Electrochem. Soc..

[cit553] Pommier R., Gril C., Marucchi J. (1981). Thin Solid Films.

[cit554] Mor G. K., Shankar K., Paulose M., Varghese O. K., Grimes C. A. (2006). Nano Lett..

[cit555] Xie Y., Deng Y., Yang C., Zeng Z., Li Y., Chen G. (2017). J. Alloys Comp..

[cit556] Xu J. Y., Liu G. Y., Li J. L., Wang X. D. (2012). Electrochim. Acta.

[cit557] Wang X., Gao M. Z. (2018). Nanoscale.

[cit558] Song Y., Liu H., Dong W., Li M. (2020). Int. J. Hydrogen Energy.

[cit559] Sreekanth T. V.-M., Nam N. D., Kim J., Yoo K. (2020). J. Electroanal. Chem..

[cit560] Kanhere P., Chen Z. (2014). Molecules.

[cit561] Wang W., Tade M. O., Shao Z. (2015). Chem. Soc. Rev..

[cit562] Lee J.-W., Kim H.-S., Park N.-G. (2016). Acc. Chem. Res..

[cit563] Rodionov I. A., Zvereva I. A. (2016). Russ. Chem. Rev..

[cit564] Moniruddin M., Ilyassov B., Zhao X., Smith E., Serikov T., Ibrayev N., Asmatulu R., Nuraje N. (2018). Mater. Today Energy.

[cit565] Yun S., Vlachopoulos N., Qurashi A., Ahmad S., Hagfeldt A. (2019). Chem. Soc. Rev..

[cit566] Nasir M. S., Yang G., Ayub I., Wang S., Wang L., Wang X., Yan W., Peng S., Ramakarishna S. (2019). Appl. Catal., B.

[cit567] Chen J., Dong C., Idriss H., Mohammed O. F., Bakr O. M. (2020). Adv. Energy Mater..

[cit568] Huang H., Pradhan B., Hofkens J., Roeffaers M. B.-J., Steele J. A. (2020). ACS Energy Lett..

[cit569] Bian H., Li D., Yan J., Liu S. (2021). J. Energy Chem..

[cit570] Wang W., Xu M., Xu X., Zhou W., Shao Z. (2020). Angew. Chem., Int. Ed..

[cit571] Kumar A., Kumar Ajay, Krishnan V. (2020). ACS Catal..

[cit572] Gupta S., Kellogg W., Xu H., Liu X., Cho J., Wu G. (2016). Chem. – Asian J..

[cit573] Irvine J. T.-S., Neagu D., Verbraeken M. C., Chatzichristodoulou C., Graves C., Mogensen M. B. (2016). Nat. Energy.

[cit574] Zhu Y., Zhou W., Shao Z. (2017). Small.

[cit575] Jamesh M.-I., Sun X. (2018). J. Power Sources.

[cit576] Ghosh S., Basu R. N. (2018). Nanoscale.

[cit577] Li A., Sun Y., Yao T., Han H. (2018). Chem. – Eur. J.

[cit578] Xu X., Wang W., Zhou W., Shao Z. (2018). Small Methods.

[cit579] Rana M., Mondal S., Sahoo L., Chatterjee K., Karthik P. E., Gautam U. K. (2018). ACS Appl. Mater. Interfaces.

[cit580] Bo X., Dastafkan K., Zhao C. (2019). ChemPhysChem.

[cit581] Feng C., Faheem B., Fu J., Xiao Y., Li C., Li Y. (2020). ACS Catal..

[cit582] Khan R., Mehran M. T., Naqvi S. R., Khoja A. H., Mahmood K., Shahzad F., Hussain S. (2020). Int. J. Energy Res..

[cit583] Wang J., Choi S., Kim J., Cha S. W., Lim J. (2020). Catalysts.

[cit584] Wu Z.-P., Lu X. F., Zang S.-Q., Lou X. W. (2020). Adv. Funct. Mater..

[cit585] Zhu Y., Lin Q., Zhong Y., Tahini H. A., Shao Z., Wang H. (2020). Energy Environ. Sci..

[cit586] Zhao J.-W., Shi Z.-X., Li C.-F., Ren Q., Li G.-R. (2021). ACS Mater. Lett..

[cit587] Sun C., Alonso J. A., Bian J. (2021). Adv. Energy Mater..

[cit588] Badreldin A., Abusrafa A. E., Abdel-Wahab A. (2021). ChemSusChem.

[cit589] Wei Y., Weng Z., Guo L., An L., Yin J., Sun S., Da P., Wang R., Xi P., Yan C.-H. (2021). Small Methods.

[cit590] Suntivich J., Gasteiger H. A., Yabuuchi N., Nakanishi H., Goodenough J. B., Shao-Horn Y. (2011). Nat. Chem..

[cit591] Grimaud A., May K. J., Carlton C. E., Lee Y.-L., Risch M., Hong W. T., Zhou J., Shao-Horn Y. (2013). Nat. Commun..

[cit592] Jung J. I., Jeong H. Y., Lee J. S., Kim M. G., Cho J. (2014). Angew. Chem., Int. Ed..

[cit593] Edgington J., Schweitzer N., Alayoglu S., Seitz L. C. (2021). J. Am. Chem. Soc..

[cit594] Nguyen T. X., Liao Y.-C., Lin C.-C., Su Y.-H., Ting J.-M. (2021). Adv. Funct. Mater..

[cit595] Kim J., Yin X., Tsao K.-C., Fang S., Yang H. (2014). J. Am. Chem. Soc..

[cit596] Xu X., Pan Y., Ge L., Chen Y., Mao X., Guan D., Li M., Zhong Y., Hu Z., Peterson V. K., Saunders M., Chen C.-T., Zhang H., Ran R., Du A., Wang H., Jiang S. P., Zhou W., Shao Z. (2021). Small.

[cit597] Zhao Y., Xu L., Mai L., Han C., An Q., Xu X., Liu X., Zhang Q. (2012). PNAS.

[cit598] Xu X., Chen Y., Zhou W., Zhu Z., Su C., Liu M., Shao Z. (2016). Adv. Mater..

[cit599] Hua B., Li M., Zhang Y.-Q., Sun Y.-F., Luo J.-L. (2017). Adv. Energy Mater..

[cit600] Sun Q., Dai Z., Zhang Z., Chen Z., Lin H., Gao Y., Chen D. (2019). J. Power Sources.

[cit601] Matsumoto Y., Kurimoto J., Sato E. (1979). J. Electron. Chem. Interfacial Electrochem..

[cit602] Kim J. S., Park I., Jeong E. S., Jin K., Seong W. M., Yoon G., Kim H., Kim B., Nam K. T., Kang K. (2017). Adv. Mater..

[cit603] Marelli E., Gazquez J., Poghosyan E., Mgller E., Gawryluk D. J., Pomjakushina E., Sheptyakov D., Piamonteze C., Aegerter D., Schmidt T. J., Medarde M., Fabbri E. (2021). Angew. Chem., Int. Ed..

[cit604] Liu H., Lei J., Yang S., Qin F., Cui L., Kong Y., Zheng X., Duan T., Zhu W., He R. (2021). Appl. Catal., B.

[cit605] Lee Y.-L., Kleis J., Rossmeisl J., Shao-Horn Y., Morgan D. (2011). Energy Environ. Sci..

[cit606] Mefford J. T., Rong X., Abakumov A. M., Hardin W. G., Dai S., Kolpak A. M., Johnston K. P., Stevenson K. J. (2016). Nat. Commun..

[cit607] Porokhin S. V., Nikitina V. A., Aksyonov D. A., Filimonov D. S., Pazhetnov E. M., Mikheev I. V., Abakumov A. M. (2021). ACS Catal..

[cit608] Mueller D. N., Machala M. L., Bluhm H., Chueh W. C. (2015). Nat. Commun..

[cit609] Calle-Vallejo F., Inoglu N. G., Su H.-Y., Martínez J. I., Man I. C., Koper M. T., Kitchin J. R., Rossmeisl J. (2013). Chem. Sci..

[cit610] Yang F., Xie J., Rao D., Liu X., Jiang J., Lu X. (2021). Nano Energy.

[cit611] Suntivich J., May K. J., Gasteiger H. A., Goodenough J. B., Shao-Horn Y. (2011). Science.

[cit612] Hwang J., Rao R. R., Giordano L., Katayama Y., Yu Y., Shao-Horn Y. (2017). Science.

[cit613] Guo Y., Tong Y., Chen P., Xu K., Zhao J., Lin Y., Chu W., Peng Z., Wu C., Xie Y. (2015). Adv. Mater..

[cit614] Diaz-Morales O., Raaijman S., Kortlever R., Kooyman P. J., Wezendonk T., Gascon J., Fu W. T., Koper M. T. (2016). Nat. Commun..

[cit615] Lee J. G., Hwang J., Hwang H. J., Jeon O. S., Jang J., Kwon O., Lee Y., Han B., Shul Y.-G. (2016). J. Am. Chem. Soc..

[cit616] Petrie J. R., Cooper V. R., Freeland J. W., Meyer T. L., Zhang Z., Lutterman D. A., Lee H. N. (2016). J. Am. Chem. Soc..

[cit617] Li B.-Q., Tang C., Wang H.-F., Zhu X.-L., Zhang Q. (2016). Sci. Adv..

[cit618] Zhu Y., Zhou W., Chen Z. G., Chen Y., Su C., Tade M. O., Shao Z. (2015). Angew. Chem., Int. Ed..

[cit619] Zhu Y., Zhou W., Zhong Y., Bu Y., Chen X., Zhong Q., Liu M., Shao Z. (2017). Adv. Energy Mater..

[cit620] Zhang D., Song Y., Du Z., Wang L., Li Y., Goodenough J. B. (2015). J. Mater. Chem. A.

[cit621] Dong F., Chen Y., Chen D., Shao Z. (2014). J. Mater. Chem. A.

[cit622] Yagi S., Yamada I., Tsukasaki H., Seno A., Murakami M., Fujii H., Chen H., Umezawa N., Abe H., Nishiyama N., Mori S. (2015). Nat. Commun..

[cit623] Xu X., Su C., Zhou W., Zhu Y., Chen Y., Shao Z. (2016). Adv. Sci..

[cit624] Sengodan S., Choi S., Jun A., Shin T. H., Ju Y.-W., Jeong H. Y., Shin J., Irvine J. T.-S., Kim G. (2014). Nat. Mater..

[cit625] Sun H., Chen G., Zhu Y., Liu B., Zhou W., Shao Z. (2017). Chem. – Eur. J..

[cit626] Hong W. T., Welsch R. E., Shao-Horn Y. (2016). J. Phys. Chem. C.

[cit627] Han B., Risch M., Lee Y.-L., Ling C., Jia H., Shao-Horn Y. (2015). Phys. Chem. Chem. Phys..

[cit628] Jung J.-I., Risch M., Park S., Kim M. G., Nam G., Jeong H.-Y., Shao-Horn Y., Cho J. (2016). Energy Environ. Sci..

[cit629] Niederberger M., Garnweitner G., Pinna N., Antonietti M. (2004). J. Am. Chem. Soc..

[cit630] Chen C. F., King G., Dickerson R. M., Papin P. A., Gupta S., Kellogg W. R., Wu G. (2015). Nano Energy.

[cit631] Han X., Hu Y., Yang J., Cheng F., Chen J. (2014). Chem. Commun..

[cit632] Zhuang S., Huang C., Huang K., Hu X., Tu F., Huang H. (2011). Electrochem. Commun..

[cit633] Lee D. U., Park H. W., Park M. G., Ismayilov V., Chen Z. (2015). ACS Appl. Mater. Interfaces.

[cit634] Zhang J., Zhao Y. B., Zhao X., Liu Z. L., Chen W. (2014). Sci. Rep..

[cit635] Ge X., Goh F. W.-T., Li B., Hor T. S.-A., Zhang J., Xiao P., Wang X., Zong Y., Liu Z. (2015). Nanoscale.

[cit636] Kim J., Chen X., Shih P.-C., Yang H. (2017). ACS Sustainable Chem. Eng..

[cit637] Bie S., Zhu Y., Su J., Jin C., Liu S., Yang R., Wu J. (2015). J. Mater. Chem. A.

[cit638] Thanh T. D., Chuong N. D., Balamurugan J., Hien H. V., Kim N. H., Lee J. H. (2017). Small.

[cit639] Pham T. V., Guo H. P., Luo W. B., Chou S. L., Wang J. Z., Liu H. K. (2017). J. Mater. Chem. A.

[cit640] Ji D., Liu C., Yao Y., Luo L., Wang W., Chen Z. (2021). Nanoscale.

[cit641] Wang T., Chen H., Yang Z., Liang J., Dai S. (2020). J. Am. Chem. Soc..

[cit642] Jin C., Cao X., Zhang L., Zhang C., Yang R. (2013). J. Power Sources.

[cit643] Xu Q., Han X., Ding F., Zhang L., Sang L., Liu X., Xu Q. (2016). J. Alloys Compd..

[cit644] Hardin W. G., Slanac D. A., Wang X., Dai S., Johnston K. P., Stevenson K. J. (2013). J. Phys. Chem. Lett..

[cit645] Hardin W. G., Mefford J. T., Slanac D. A., Patel B. B., Wang X., Dai S., Zhao X., Ruoff R. S., Johnston K. P., Stevenson K. J. (2014). Chem. Mater..

[cit646] Testino A., Buscaglia M. T., Buscaglia V., Viviani M., Bottino C., Nanni P. (2004). Chem. Mater..

[cit647] Ng J., Xu S., Zhang X., Yang H. Y., Sun D. D. (2010). Adv. Funct. Mater..

[cit648] Zhu T., Troiani H. E., Mogni L. V., Han M., Barnett S. A. (2018). Joule.

[cit649] Donn Matienzo D. J., Kutlusoy T., Divanis S., Di Bari C., Instuli E. (2020). Catalysts.

[cit650] Tham N. N., Ge X., Yu A., Li B., Zong Y., Liu Z. (2021). Inorg. Chem. Front..

[cit651] Gozzo C. B., Soares M. R.-S., Sczancoski J. C., Nogueira I. C., Leite E. R. (2019). Int. J. Hydrogen.

[cit652] Hwang C., Gwon O., Jo H., Ok K. M., Kim G. (2017). ChemElectroChem.

[cit653] Chen G., Zhou W., Guan D., Sunarso J., Zhu Y., Hu X., Zhang W., Shao Z. (2017). Sci. Adv..

[cit654] Pergolesi D., Fabbri E., D’Epifanio A., Di Bartolomeo E., Tebano A., Sanna S., Licoccia S., Balestrino G., Traversa E. (2010). Nat. Mater..

[cit655] Wang S., Yoon J., Kim G., Huang D., Wang H., Jacobson A. J. (2010). Chem. Mater..

[cit656] Stoerzinger K. A., Choi W. S., Jeen H., Lee H. N., Shao-Horn Y. (2015). Phys. Chem. Lett..

[cit657] Risch M., Stoerzinger K. A., Maruyama S., Hong W. T., Takeuchi I., Shao-Horn Y. (2014). J. Am. Chem. Soc..

[cit658] Hoat P. D., Ha H.-H., Hung P. T., Hien V. X., Lee S., Heo Y.-W. (2021). Thin Solid Films.

[cit659] Fang T., Huang H., Feng J., Hu Y., Qian Q., Yan S., Yu Z., Li Z., Zou Z. (2019). Research.

[cit660] Park H. W., Lee D. U., Park M. G., Ahmed R., Seo M. H., Nazar F., Chen Z. W. (2015). ChemSusChem.

[cit661] Therese G. H.-A., Dinamani M., Vishnu Kamath P. (2005). J. Appl. Electrochem..

[cit662] Park B.-K., Song R.-H., Lee S.-B., Lim T.-H., Park S.-J., Jung W. C., Lee J.-W. (2017). J. Power Sources.

[cit663] Liu G., Karuturi S. K., Chen H., Wang D., Ager J. W., Simonov A. N., Tricoli A. (2020). Solar Energy.

[cit664] Vignesh A., Prabu M., Shanmugam S. (2016). ACS Appl. Mater. Interfaces.

[cit665] Zhao B., Zhang L., Zhen D., Yoo S., Ding Y., Chen D., Chen Y., Zhang Q., Doyle B., Xiong X., Liu M. (2017). Nat. Commun..

[cit666] Zhen D., Zhao B., Shin H., Bu Y., Ding Y., He G., Liu M. (2017). Adv. Mater. Interfaces.

[cit667] Park H. W., Lee D. U., Zamani P., Seo M. H., Zazar L. F., Chen Z. W. (2014). Nano Energy.

[cit668] Wang Y., Ren J., Wang Y., Zhang F., Liu X., Guo Y., Lu G. (2008). J. Phys. Chem. C.

[cit669] Lu F., Wang Y., Jin C., Li F., Yang R., Chen F. (2015). J. Power Sources.

[cit670] Lee Y. C., Peng P. Y., Chang W. S., Huang C. M. (2014). J. Taiwan Inst. Chem. Eng..

[cit671] Wang Y., Cui X., Li Y., Chen L., Shu Z., Chen H., Shi J. (2013). Dalton Trans..

[cit672] Wang J., Qiu T., Chen X., Lu Y., Yang W. (2014). J. Power Sources.

[cit673] Wang J., Fu Y., Xu Y., Wu J., Tian J.-H., Yang R. (2016). Int. J. Hydrogen Energy.

[cit674] Shchukin D. G., Yaremchenko A. A., Ferreira M. G.-S., Kharton V. V. (2005). Chem. Mater..

[cit675] Sun Y.-F., Zhang Y.-Q., Yang Y.-L., Chen J., Hua B., Shi Y.-X., Wang C.-A., Luo J.-L. (2017). Appl. Catal., B.

[cit676] Xu J.-J., Wang Z.-L., Xu D., Meng F.-Z., Zhang X.-B. (2014). Energy Environ. Sci..

[cit677] Sennu P., Aravindan V., Nahm K. S., Lee Y. S. (2017). J. Mater. Chem. A.

[cit678] Deganello F., Oko D. N., Testa M. L., La Parola V., Tummino M. L., Soares C. O., Rivera J. G., Orozco G., Guay D., Tavares A. C. (2018). ACS Appl. Energy Mater..

[cit679] McBean C. L., Liu H., Scofield M. E., Li L., Wang L., Bernstein A., Wong S. S. (2017). ACS Appl. Mater. Interfaces.

[cit680] Schäfer H., Hess C., Tobergte H., Volf A., Ichlmann S., Eickmeier H., Voss B., Kashaev N., Nordmann J., Akram W., Hartmann-Azanza B., Steinhart M. (2015). Small.

[cit681] Zhou S., Miao X., Zhao X., Ma C., Qiu Y., Hu Z., Zhao J., Shi L., Zeng J. (2016). Nat. Commun..

[cit682] Wang P. F., Cheng Q., Mao C., Su W., Yang L., Wang G., Zou L., Shi Y., Yan C., Zou Z., Yang H. (2021). J. Power Sources.

[cit683] Selvadurai P. B., Xiong T., Huang P., Tan Q., Huang Y., Yang H., Balogun M.-S. (2021). J. Mater. Chem.
A.

[cit684] Oh N. K., Seo J., Lee S., Kim H.-J., Kim U., Lee J., Han Y.-K., Park H. (2021). Nat. Commun..

[cit685] Sutherland F. L., Zaw K., Meffre S., Giuliani G., Fallick A. E., Graham I. T., Webb G. B. (2009). Aust. J. Earth Sci..

[cit686] Federman D. , Consumer Guide To Colored Gemstones, Modern Jeweler Magazine, New York, 1990, p. 214

[cit687] *Sir Thomas Butler (1989). The Crown Jewels and Coronation Ceremony. Pitkin. p. 6. ISBN 978-0-85372-467-4*

[cit688] Marco J. F., Gancedo J. R., Gracia M., Gautier J. L., Rios E. I., Palmer H. M., Greaves C., Berry F. J. (2001). J. Mater. Chem..

[cit689] Hun Kim D., Aimon N. M., Sun X., Ross C. A. (2014). Adv. Funct. Mater..

[cit690] Yadav R. S., Kuřitka I., Vilcakova J., Havlica J., Masilko J., Kalina L., Tkacz J., Enev V., Hajdúchová M. (2017). J. Phys. Chem. Solid.

[cit691] Allix M., Chenu S., Veron E., Poumeyrol T., Kouadri-Boudjelthia E. A., Alahrache S., Porcher F., Massiot D., Fayon F. (2013). Chem. Mater..

[cit692] Sampath S. K., Cordaro J. F. (1998). J. Am. Ceram. Soc..

[cit693] Ewais E. M.-M., Besisa D. H.-A., El-Amir A. A.-M., El-Sheikh S. M., Rayan D. E. (2015). J. Alloys Comp..

[cit694] Sonoyama N., Kawamura K., Yamada A., Kanno R. (2006). J. Electrochem. Soc..

[cit695] Hemberger J., Lunkenheimer P., Fichtl R., Krug von Nidda H.-A., Tsurkan V., Loidl A. (2005). Nature.

[cit696] Padmaraj O., Venkateswarlu M., Satyanarayana N. (2015). Ceram. Int..

[cit697] Scharner S., Weppner W., Schmid-Beurmann P. (1999). J. Electrochem. Soc..

[cit698] Un Lee D., Kim B. J., Chen Z. (2013). J. Mater. Chem. A.

[cit699] Wu Z., Zhu Y., Ji X. (2014). J. Mater. Chem. A.

[cit700] Kessler T., Visintin A., de Chialvo M. R., Triaca W. E., Arvia A. J. (1989). J. Electroanal. Chem..

[cit701] Restovic A., Poillerat G., Koenig J. F., Chartier P. (1991). Thin Solid Films.

[cit702] Li M., Xiong Y., Liu X., Bo X., Zhang Y., Han C., Guo L. (2015). Nanoscale.

[cit703] Chakrapani K., Bendt G., Hajiyani H., Lunkenbein T., Greiner M. T., Masliuk L., Salamon S., Landers J., Schlögl R., Wende H., Pentcheva R., Schulz S., Behrens M. (2018). ACS Catal..

[cit704] Wei C., Feng Z., Scherer G. G., Barber J., Shao-Horn Y., Xu Z. J. (2017). Adv. Mater..

[cit705] Kang M. J., Park H., Jegal J., Hwang S. Y., Kang Y. S., Cha H. G. (2019). Appl. Catal., B.

[cit706] Wang W., Kuai L., Cao W., Huttula M., Ollikkala S., Ahopelto T., Honkanen A.-P., Huotari S., Yu M., Geng B. (2017). Angew. Chem., Int. Ed..

[cit707] Li C., Han X., Cheng F., Hu Y., Chen C., Chen J. (2015). Nat. Commun..

[cit708] Zhou Y., Sun S., Song J., Xi S., Chen B., Du Y., Fisher A. C., Cheng F., Wang X., Zhang H., Xu Z. J. (2018). Adv. Mater..

[cit709] Bao J., Zhang X., Fan B., Zhang J., Zhou M., Yang W., Hu X., Wang H., Pan B., Xie Y. (2015). Angew. Chem., Int. Ed..

[cit710] Zhu H., Zhang S., Huang Y.-X., Wu L., Sun S. (2013). Nano Lett..

[cit711] Bajdich M., García-Mota M., Vojvodic A., Nørskov J. K., Bell A. T. (2013). J. Am. Chem. Soc..

[cit712] Zhao Q., Yan Z., Chen C., Chen J. (2017). Chem. Rev..

[cit713] Liu X.-M., Cui X., Dastafkan K., Wang H.-F., Tang C., Zhao C., Chen A., He C., Han M., Zhang Q. (2021). J. Energy Chem..

[cit714] She Z. W., Kibsgaard J., Dickens C. F., Chorkendorff I., Norskov J. K., Jaramillo T. F. (2017). Science.

[cit715] Gonçalves J. M., Silva M. N.-T., Naik K. K., Martins P. R., Rocha D. P., Nossol E., Munoz R. A.-A., Angnes L., Rout C. S. (2021). J. Mater. Chem. A.

[cit716] Rajan A. G., Martirez J. M.-P., Carter E. A. (2020). ACS Catal..

[cit717] Pilania G., Kocevski V., Valdez J. A., Kreller C. R., Uberuaga B. P. (2020). Commun. Mater..

[cit718] Uchaker E., Cao G. Z. (2015). Chem. – Asian J..

[cit719] Armstrong A. R., Tee D. W., La Mantia F., Nova P., Bruce P. G. (2008). J. Am. Chem. Soc..

[cit720] Lim D., Kong H., Lim C., Kim N., Shim S. E., Baeck S.-H. (2019). Int. J. Hydrogen Energy.

[cit721] Sun J., Guo N., Shao Z., Huang K., Li Y., He F., Wang Q. (2018). Adv. Energy. Mater..

[cit722] Esposito L., Piancastelli A., Miceli P., Martelli S. (2015). J. Eur. Ceram. Soc..

[cit723] Lai C., Chen J., Knight J. C., Manthiram A., Navrotsky A. (2016). ChemPhysChem.

[cit724] Huang Y., Dong Y., Li S., Lee J., Wang C., Zhu Z., Xue W., Li Y., Li J. (2021). Adv. Energy Mater..

[cit725] Radaelli P. G., Horibe Y., Gutmann M. J., Ishibashi H., Chen C., Ibberson R. M., Koyama Y., Hor Y. S., Kiryukhin V., Cheong S. (2002). Nature.

[cit726] Araujo C., Almeida B. G., Aguiar M., Mendes J. A. (2008). Vacuum.

[cit727] Silwal P., Miao L., Stern I., Zhou X., Hu J., Kim D. H. (2012). Appl. Phys. Lett..

[cit728] Schroder H. (1967). Z. Phys. Chem..

[cit729] Singh V. K., Sinha R. K. (1997). Mater. Lett..

[cit730] Chen Z., Kronawitter C. X., Koel B. E. (2015). Phys. Chem. Chem. Phys..

[cit731] Guo C. X., Chen S., Lu X. (2014). Nanoscale.

[cit732] Reith L., Lienau K., Cook D. S., More R., Walton R. I., Patzke G. R. (2018). Chem. – Eur. J..

[cit733] Koza J. A., He Z., Miller A. S., Switzer J. A. (2012). Chem. Mater..

[cit734] Prabu M., Ketpang K., Shanmugam S. (2014). Nanoscale.

[cit735] Peng S., Li L., Hu Y., Srinivasan M., Cheng F., Chen J., Ramakrishna S. (2015). ACS Nano.

[cit736] Landon J., Demeter E., İnoglu N., Keturakis C., Wachs I. E., Vasić R., Frenkel A. I., Kitchin J. R. (2012). ACS Catal..

[cit737] Bid S., Sahu P., Pradhan S. K. (2007). Analysis Phys. E.

[cit738] Aymes D., Millot N., Nivoix V., Perriat P., Gillot B. (1997). Solid State Ion.

[cit739] Nie Y., Li L., Wei Z. D. (2015). Chem. Soc. Rev..

[cit740] Dziembaj R., Molenda M. (2003). J. Power Sources.

[cit741] Menezes P. W., Indra A., Bergmann A., Chernev P., Walter C., Dau H., Strasser P., Driess M. (2016). J. Mater. Chem. A.

[cit742] Cui H. T., Zayat M., Levy D. (2005). J. Sol-Gel Sci. Technol..

[cit743] Thirunakaran R., Sivashanmugam A., Gopukumar S., Dunnill C. W., Gregory D. H. (2008). Mater. Res. Bull..

[cit744] Park J., Joo J., Kwon S. G., Jang Y., Hyeon T. (2007). Angew. Chem., Int. Ed..

[cit745] Zhang S., Ying M., Yu J., Zhan W., Wang L., Guo Y., Guo Y. (2021). Appl. Catal., B.

[cit746] Massoudi J., Smari M., Nouri K., Dhahri E., Khirouni K., Bertaina S., Bessais L., Hlil E. K. (2020). RSC Adv..

[cit747] Rani N., Dehiya B. S. (2020). Ceram. Int..

[cit748] Acharyya S. S., Ghosh S., Siddiqui N., Konathala L. N.-S., Bal R. (2015). RSC Adv..

[cit749] Han J., Liu X., Wan H., Wu D., Chen G., Li J., Cao Y., Ma R. (2020). ACS Sustainable Chem. Eng..

[cit750] Yang J., Zhu G., Liu Y., Xia J., Ji Z., Shen X., Wu S. (2016). Adv. Funct. Mater..

[cit751] Indra A., Menezes P. W., Sahraie N. R., Bergmann A., Das C., Tallarida M., Schmeisser D., Strasser P., Driess M. (2014). J. Am. Chem. Soc..

[cit752] Gao X., Zhang H., Li Q., Yu X., Hong Z., Zhang X., Liang C., Lin Z. (2016). Angew. Chem., Int. Ed..

[cit753] Han X., He G., He Y., Zhang J., Zheng X., Li L., Zhong C., Hu W., Deng Y., Ma T.-Y. (2018). Adv. Energy Mater..

[cit754] Devaguptapu S. V., Hwang S., Karakalos S., Zhao S., Gupta S., Su D., Xu H., Wu G. (2017). ACS Appl. Mater. Interfaces.

[cit755] Jin C., Lu F., Cao X., Yang Z., Yang R. (2013). J. Mater. Chem. A.

[cit756] Zhao J., He Y., Chen Z., Zheng X., Han X., Rao D., Zhong C., Hu W., Deng Y. (2019). ACS Appl. Mater. Interfaces.

[cit757] Kong F. Q. (2012). Electrochim. Acta.

[cit758] Ding R., Lv L. L., Qi L., Jia M. J., Wang H. Y. (2014). RSC Adv..

[cit759] Ma T. Y., Dai S., Jaroniec M., Qiao S. Z. (2014). Chem. – Eur. J..

[cit760] Ding R., Qi L., Jia J. M., Wang H. Y. (2014). Nanoscale.

[cit761] Han X. P., Zhang T. R., Du J., Cheng F. Y., Chen J. (2013). Chem. Sci..

[cit762] Poux T., Napolskiy F. S., Dintzer T., Kéranguéven G., Istomin S. Y., Tsirlina G. A., Antipov E. V., Savinova E. R. (2012). Catal. Today.

[cit763] Cheng F., Shen J., Peng B., Pan Y., Tao Z., Chen J. (2011). Nat. Chem..

[cit764] Rosen J., Hutchings G. S., Jiao F. (2014). J. Catal..

[cit765] Da Silva L. M., Boodts J. F.-C., De Faria L. A. (2001). Electrochim. Acta.

[cit766] Amin H. M.-A., Baltruschat H., Wittmaier D., Friedrich K. A.-A. (2015). Electrochim. Acta.

[cit767] Pletcher D., Li X. H., Price S. W.-T., Russell A. E., Sonmez T., Thompson S. J. (2016). Electrochim. Acta.

[cit768] Svegl F., Orel B., Grabec-Svegl I., Kaucic V. (2000). Electrochim Acta.

[cit769] Li Y., Hasin P., Wu Y. (2010). Adv. Mater..

[cit770] Bikkarolla S. K., Papakonstantinou P. (2015). J. Power Sources.

[cit771] Yeo B. S., Bell A. T. (2011). J. Am. Chem. Soc..

[cit772] Cho Y., Lee S., Lee Y., Hong T., Cho J. (2011). Adv. Energy Mater..

[cit773] Zhuang Z., Sheng W., Yan Y. (2014). Adv. Mater..

[cit774] Sirisomboonchai S., Li X., Kitiphatpiboon N., Channoo R., Li S., Ma Y., Kongparakul S., Samart C., Abudula A., Guan G. (2020). J. Mater. Chem. A.

[cit775] Appiah-Ntiamoah R., Baye A. F., Kim H. (2020). ChemElectroChem.

[cit776] Jiang R., Baker D. R., Tran D. T., Li J., Leff A. C., Zhang S. S. (2020). ACS Appl. Nano Mater.

[cit777] Rivas-Murias B., Testa-Anta M., Torruella P., Estradé S., Peiró F., Rodríguez-Gonzalez B., Comesana-Hermo M., Salgueirino V. (2020). Chem. Mater..

[cit778] Kwon S., Lee J. H. (2020). Dalton Trans..

[cit779] Yang J. X., Dai B.-H., Chiang C.-Y., Chiu I.-C., Pao C.-W., Lu S.-Y., Tsao I.-Y., Lin S.-T., Chiu C.-T., Yeh J.-W., Chang P.-C., Hung W.-H. (2021). ACS Nano.

[cit780] Dai Z., Du X., Wang Y., Han X., Zhang X. (2021). Daltons Trans..

[cit781] Konno Y., Yamamoto T., Nagayama T. (2021). Nanoscale.

[cit782] Wani A. A., Bhat M. M., Sofi F. A., Bhat S. A., Ingole P. P., Rashid N., Bhat M. A. (2021). New J. Chem..

[cit783] Shah A. K., Bhowmick S., Gogoi D., Peela N. R., Qureshi M. (2021). Chem. Commun..

[cit784] Yue X., Qin X., Chen Y., Peng Y., Liang C., Feng M., Qiu X., Shao M., Huang S. (2021). Adv. Sci..

[cit785] Doppelbauer D., Aljabour A., Coskun H., Sun H., Gusenbauer M., Lumetzberger J., Primetzhofer D., Faina B., Duchoslav J., Kehrer M., Stifter D., Groiss H., Ney V., Ney A., Stadler P. (2021). Mater. Adv..

[cit786] Guo K., Wang Y., Huang J., Lu M., Li H., Peng Y., Xi P., Zhang H., Huang J., Lu S., Xu C. (2021). ACS Catal..

[cit787] Jia J., Li X., Chen G. (2010). Electrochim. Acta.

[cit788] Sufredini H. B., Cerne J. L., Crnkovic F. C., Machado S. A.-S., Avaca L. A. (2000). Int. J. Hydrogen Energy.

[cit789] Kale B. B., Baeg J.-O., Lee S. M., Chang H., Moon S.-J., Lee C. W. (2006). Adv. Funct. Mater..

[cit790] Saadi S., Bouguelia A., Trari M. (2006). Renew. Energy.

[cit791] Zhu Y. P., Ma T. Y., Jaroniec M., Qiao S. Z. (2017). Angew. Chem., Int. Ed..

[cit792] Chatenet M., Benziger J., Inaba M., Kjelstrup S., Zawodzinski T., Raccichini R. (2020). J. Power Sources.

[cit793] Kristajic N., Trasatti S. (1995). J. Electrochem. Soc..

[cit794] Kristajic N., Trasatti S. (1998). J. Appl. Electrochem..

[cit795] Huang L., Chen D., Luo G., Lu Y.-R., Chen C., Zou Y., Dong C.-L., Li Y., Wang S. (2019). Adv. Mater..

[cit796] Peng Z., Jia D., Al-Enizi A. M., Elzatahry A. A., Zheng G. (2015). Adv. Energy Mater..

[cit797] Peng S., Gong F., Li L., Yu D., Ji D., Zhang T., Hu Z., Zhang Z., Chou S., Du Y., Ramakrishna S. (2018). J. Am. Chem. Soc..

[cit798] Zhang J., Shang X., Ren H., Chi J., Fu H., Dong B., Liu C., Chai Y. (2019). Adv. Mater..

[cit799] Wang Z., Liu H., Ge R., Ren X., Ren J., Yang D., Zhang L., Sun X. (2018). ACS Catal..

[cit800] Muthurasu A., Maruthapandian V., Kim H. Y. (2019). Appl. Catal., B.

[cit801] Senthilkumar B., Selvan R. K., Vonothbabu P., Perelshtein I., Gedanken A. (2011). Mat. Chem. Phys..

[cit802] Flores-Lasluisa J. X., Quilez-Bermejo J., Ramirez-Perez A. C., Huerta F., Cazorla-Amoros D., Morallon E. (2019). Materials.

[cit803] Tan J., Xu S., Zhang H., Cao H., Zheng G. (2021). Electrochim. Acta.

[cit804] Lee J., Son N., Park N.-K., Ryu H.-J., Baek J.-I., Sohn Y., Do J. Y., Kang M. (2021). Electrochim Acta.

[cit805] Zareie-Darmian A., Farsi H., Farrokhi A., Sarhaddi R., Li Z. (2021). Phys. Chem. Chem. Phys..

[cit806] Ge J., Zhang W., Tu J., Xia T., Chen S., Xie G. (2020). Small.

[cit807] Ren C., Chen Y., Du L., Wang Q., Li L., Tian G. (2021). ChemElectroChem.

[cit808] Song W., Xu M., Teng X., Niu Y., Gong S., Liu X., He X., Chen Z. (2021). Nanoscale.

[cit809] Janani G., Yuvaraj S., Surendran S., Chae Y., sim Y., Song S.-J., Park W., Kim M.-J., Sim U. (2020). J. Alloys Compd..

[cit810] Wiliamson E. M., Tappan B. A., Mora-Tamez L., Barim G., Brutchey R. L. (2021). ACS Nano.

[cit811] Debnath B., Parvin S., Dixit H., Bhattacharyya S. (2020). ChemSusChem.

[cit812] Dionigi F., Strasser P. (2016). Adv. Energy Mater..

[cit813] Kurzman J. A., Dettelbach K. E., Martinolich A. J., Berlinguette C. P., Neilson J. R. (2015). Chem. Mater..

[cit814] Zou S. H., Burke M. S., Kast M. G., Fan J., Danilovic N., Boettcher S. W. (2015). Chem. Mater..

[cit815] Bergmann A., Zaharieva I., Dau H., Strasser P. (2013). Energy Environ. Sci..

[cit816] Ramirez A., Hillebrand P., Stellmach D., May M. M., Bogdanoff P., Fiechter S. (2014). J. Phys. Chem. C.

[cit817] Melder J., Bogdanoff P., Zaharieva I., Fiechter S., Dau H., Kurz P. (2020). J. Phys. Chem..

[cit818] Tesch M. F., Bonke S. A., Jones T. E., Shaker M. N., Xiao J., Skorupska K., Mom R., Melder J., Kurz P., Knop-Gericke A., Schlogl R., Hocking R. K., Simonov A. N. (2019). Angew. Chem., Int. Ed..

[cit819] Zaharieva I., Chernev P., Risch M., Klingan K., Kohlhoff M., Fischer A., Dau H. (2012). Energy Environ. Sci..

[cit820] Smith P. F., Deibert B. J., Kaushik S., Gardner G., Hwang S. J., Wang H., Al-Sharab J. F., Garfunkel E., Fabris L., Li J., Dismukes G. C. (2016). ACS Catal..

[cit821] Menezes P. W., Walter C., Hausmann J. N., Beltran-Suito R., Schlesiger C., Praetz S., Verchenko V. Y., Shevelkov A. V., Driess M. (2019). Angew. Chem., Int. Ed..

[cit822] Xu Y., Wang C., Huang Y., Fu J. (2021). Nano Energy.

[cit823] Zhang B., Zheng X., Voznyy O., Comin R., Bajdich M., García-Melchor M., Han L., Xu J., Liu M., Zheng L., García de Arquer F. P., Dinh C. T., Fan F., Yuan M., Yassitepe E., Chen N., Regier T., Liu P., Li Y., De Luna P., Janmohamed A., Xin H. L., Yang H., Vojvodic A., Sarge E. H. (2016). Science.

[cit824] Haber J. A., Xiang C. X., Guevarra D., Jung S. H., Jin J., Gregoire J. M. (2014). Chemelectrochem.

[cit825] Gerken J. B., Shaner S. E., Masse R. C., Porubsky N. J., Stahl S. S. (2014). Energy Environ. Sci..

[cit826] Burke M. S., Zou S. H., Enman L. J., Kellon J. E., Gabor C. A., Pledger E., Boettcher S. W. (2015). J. Phys. Chem. Lett..

[cit827] Chung D. Y., Lopes P. P., Martins P. F.-B. D., He H. Y., Kawaguchi T., Zapol P., You H., Tripkovic D., Strmcnik D., Zhu Y. S., Seifert S., Lee S., Stamenkovic V. R., Markovic N. M. (2020). Nat. Energy.

[cit828] Corrigan D. A. (1987). J. Electrochem. Soc..

[cit829] Batchellor A. S., Boettcher S. W. (2015). ACS Catal..

[cit830] Watzele S., Hauenstein P., Liang Y. C., Xue S., Fichtner J., Garlyyev B., Scieszka D., Claude F., Maillard F., Bandarenka A. S. (2019). ACS Catal..

[cit831] Dionigi F., Zhu J., Zeng Z., Merzdorf T., Sarodnik H., Gliech M., Pan L., Li W.-X., Greeley J., Strasser P. (2021). Angew. Chem., Int. Ed..

[cit832] Watzele S., Bandarenka A. S. (2016). Electroanalysis.

[cit833] McCrory C. C.-L., Jung S., Peters J. C., Jaramillo T. F. (2013). J. Am. Chem. Soc..

[cit834] Dresp S., Dionigi F., Loos S., Ferreira de Araujo J., Spöri C., Gliech M., Dau H., Strasser P. (2018). Adv. Energy Mater..

[cit835] Andronescu C., Barwe S., Ventosa E., Masa J., Vasile E., Konkena B., Moller S., Schuhmann W. (2017). Angew. Chem., Int. Ed..

[cit836] Lu X. Y., Zhao C. A. (2015). Nat. Commun..

[cit837] Andronescu C., Seisel S., Wilde P., Barwe S., Masa J., Chen Y. T., Ventosa E., Schuhmann W. (2018). Chem. – Eur. J..

[cit838] Chen R., Hung S.-F., Zhou D., Gao J., Yang C., Tao H., Yang H. B., Zhang L., Zhang L., Xiong Q., Chen H. M., Liu B. (2019). Adv. Mater..

[cit839] Kim Y. T., Lopes P. P., Park S. A., Lee A. Y., Lim J., Lee H., Back S., Jung Y., Danilovic N., Stamenkovic V., Erlebacher J., Snyder J., Markovic N. M. (2017). Nat. Commun..

[cit840] Barwe S., Andronescu C., Masa J., Schuhmann W. (2017). Curr. Opin. Electrochem..

[cit841] Gong M., Dai H. J. (2015). Nano Res..

[cit842] Diaz-Morales O., Ledezma-Yanez I., Koper M. T.-M., Calle-Vallejo F. (2015). ACS Catal..

[cit843] Chen J. Y.-C., Dang L. N., Liang H. F., Bi W. L., Gerken J. B., Jin S., Alp E. E., Stahl S. S. (2015). J. Am. Chem. Soc..

[cit844] Gong M., Li Y., Wang H., Liang Y., Wu J. Z., Zhou J., Wang J., Regier T., Wei F., Dai H. (2013). J. Am. Chem. Soc..

[cit845] Liu X., Wang X., Yuan X., Dong W., Huang F. (2016). J. Mater. Chem. A.

[cit846] Zhang X., Zhao Y., Zhao Y., Shi R., Waterhouse G. I.-N., Zhang T. (2019). Adv. Energy Mater..

[cit847] Lee S., Banjac K., Lingenfelder M., Hu X. (2019). Angew. Chem., Int. Ed..

[cit848] Yu L., Yang J. F., Guan B. Y., Lu Y., Lou X. W. (2018). Angew. Chem., Int. Ed..

[cit849] Demourguesguerlou L., Braconnier J. J., Delmas C. (1993). J. Solid State Chem..

[cit850] Roy C., Sebok B., Scott S. B., Fiordaliso E. M., Sorensen J. E., Bodin A., Trimarco D. B., Damsgaard C. D., Vesborg P. C.-K., Hansen O., Stephens I. E.-L., Kibsgaard J., Chorkendorff I. (2018). Nat. Catal..

[cit851] Klaus S., Cai Y., Louie M. W., Trotochaud L., Bell A. T. (2015). J. Phys. Chem. C.

[cit852] Hunter B. M., Blakemore J. D., Deimund M., Gray H. B., Winkler J. R., Müller A. M. (2014). J. Am. Chem. Soc..

[cit853] Smith R. D.-L., Prévot M. S., Fagan R. D., Zhang Z., Sedach P. A., Siu M. K.-J., Trudel S., Berlinguette C. P. (2013). Science.

[cit854] Smith R. D.-L., Pasquini C., Loos S., Chernev P., Klingan K., Kubella P., Mohammadi M. R., Gonzalez-Flores D., Dau H. (2018). Energy Environ. Sci..

[cit855] Zhang B., Lui Y. H., Zhou L., Tang X., Hu S. (2017). J. Mater. Chem. A.

[cit856] Zhang G., Feng Y.-S., Lu W.-T., He D., Wang C.-Y., Li Y.-K., Wang X.-Y., Cao F.-F. (2018). ACS Catal..

[cit857] Xu P., Li J., Luo J., Wei L., Zhang D., Zhou D., Xu W., Yuan D. (2018). Sci. Rep..

[cit858] Li B.-Q., Zhang S.-Y., Tang C., Cui X., Zhang Q. (2017). Small.

[cit859] Pei C., Gu Y., Liu Z., Yu X., Feng L. (2019). ChemSusChem.

[cit860] Guo L., Marcus K., Zhang S., Yang Z., Perea D. E., Zhou L., Du Y., Yang Y. (2017). ACS Catal..

[cit861] Xu X., Song F., Hu X. (2016). Nat. Commun..

[cit862] Nai J., Lu Y., Yu L., Wang X., Lou X. W. (2017). Adv. Mater..

[cit863] Li D., Liu H., Feng L. (2020). Energy Fuels.

[cit864] Zhao J., Zhang J.-J., Li Z.-Y., Bu X.-H. (2020). Small.

[cit865] Fu G., Cui Z., Chen Y., Xu L., Tang Y., Goodenough J. B. (2017). Nano Energy.

[cit866] Jia X., Zhao Y., Chen G., Shang L., Shi R., Kang X., Waterhouse G. I.-N., Wu L.-Z., Tung C.-H., Zhang T. (2016). Adv. Energy Mater..

[cit867] Xiong P., Zhang X., Wan H., Wang S., Zhao Y., Zhang J., Zhou D., Gao W., Ma R., Sasaki T., Wang G. (2019). Nano Lett..

[cit868] Tang C., Wang H.-S., Wang H.-F., Zhang Q., Tian G.-L., Nie J.-Q., Wei F. (2015). Adv. Mater..

[cit869] Ma W., Ma R., Wang C., Liang J., Liu X., Zhou K., Sasaki T. (2015). ACS Nano.

[cit870] Wang W., Liu Y., Li J., Luo J., Fu L., Chen S. (2018). J. Mater. Chem. A.

[cit871] Yu X., Zhang M., Yuan W., Shi G. (2015). J. Mater. Chem. A.

[cit872] Ali-Löytty H., Louie M. W., Singh M. R., Li L., Sanchez Casalongue H. G., Ogasawara H., Crumlin E. J., Liu Z., Bell A. T., Nilsson A., Friebel D. (2016). J. Phys. Chem. C.

[cit873] Chakthranont P., Kibsgaard J., Gallo A., Park J., Mitani M., Sokaras D., Kroll T., Sinclair R., Mogensen M. B., Jaramillo T. F. (2017). ACS Catal..

[cit874] Deng X., Sorescu D. C., Waluyo I., Hunt A., Kauffman D. R. (2020). ACS Catal..

[cit875] Gu H., Shi G., Chen H.-C., Xie S., Li Y., Tong H., Yang C., Zhu C., Mefford J. T., Xia H., Chueh W. C., Chen H. M., Zhang L. (2020). ACS Energy Lett..

[cit876] Zadick A., Dubau L., Sergent N., Berthomé G., Chatenet M. (2015). ACS Catal..

[cit877] Lafforgue C., Chatenet M., Dubau L., Dekel D. R. (2018). ACS Catal..

[cit878] Friebel D., Louie M. W., Bajdich M., Sanwald K. E., Cai Y., Wise A. M., Cheng M. J., Sokaras D., Weng T. C., Alonso-Mori R., Davis R. C., Bargar J. R., Norskov J. K., Nilsson A., Bell A. T. (2015). J. Am. Chem. Soc..

[cit879] Li X., Walsh F. C., Pletcher D. (2011). Phys. Chem. Chem. Phys..

[cit880] Rossmeisl J., Logadottir A., Nørskov J. K. (2005). Chem. Phys..

[cit881] Li Y. F., Selloni A. (2014). ACS Catal..

[cit882] Zaffran J., Toroker M. C. (2016). ChemistrySelect.

[cit883] Cai Z., Zhou D., Wang M., Bak S.-M., Wu Y., Wu Z., Tian Y., Xiong X., Li Y., Liu W., Siahrostami S., Kuang Y., Yang X.-Q., Duan H., Feng Z., Wang H., Sun X. (2018). Angew. Chem., Int. Ed..

[cit884] Li N., Bediako D. K., Hadt R. G., Hayes D., Kempa T. J., von Cube F., Bell D. C., Chen L. X., Nocera D. G. (2017). Proc. Natl. Acad. Sci. U. S. A..

[cit885] Drevon D., Gorlin M., Chernev P., Xi L. F., Dau H., Lange K. M. (2019). Sci. Rep..

[cit886] Trześniewski B. J., Diaz-Morales O., Vermaas D. A., Longo A., Bras W., Koper M. T.-M., Smith W. A. (2015). J. Am. Chem. Soc..

[cit887] de Araújo J. F., Dionigi F., Merzdorf T., Oh H. S., Strasser P. (2021). Angew. Chem., Int. Ed..

[cit888] Shin H., Xiao H., Goddard W. A. (2018). J. Am. Chem. Soc..

[cit889] Görlin M., Halldin Stenlid J., Koroidov S., Wang H.-Y., Börner M., Shipilin M., Kalinko A., Murzin V., Safonova O. V., Nachtegaal M., Uheida A., Dutta J., Bauer M., Nilsson A., Diaz-Morales O. (2020). Nat. Commun..

[cit890] Zaffran J., Stevens M. B., Trang C. D.-M., Nagli M., Shehadeh M., Boettcher S. W., CaspaCry Toroker M. (2017). Chem. Mater..

[cit891] Hunter B. M., Hieringer W., Winkler J. R., Gray H. B., Muller A. M. (2016). Energy Environ. Sci..

[cit892] Zhou D., Cai Z., Bi Y., Tian W., Luo M., Zhang Q., Zhang Q., Xie Q., Wang J., Li Y., Kuang Y., Duan X., Bajdich M., Siahrostami S., Sun X. (2018). Nano Res..

[cit893] Lee S., Bai L. C., Hu X. L. (2020). Angew. Chem., Int. Ed..

[cit894] Zhou D., Wang S., Jia Y., Xiong X., Yang H., Liu S., Tang J., Zhang J., Liu D., Zheng L., Kuang Y., Sun X., Liu B. (2019). Angew. Chem., Int. Ed..

[cit895] Chen J., Li H., Chen S., Fei J., Liu C., Yu Z., Shin K., Liu Z., Song L., Henkelman G., Wei L., Chen Y. (2021). Adv. Energy Mater..

[cit896] Zhang L., Cai W., Bao N. (2021). Adv. Mater..

[cit897] Yang Y., Dang L., Shearer M. J., Sheng H., Li W., Chen J., Xiao P., Zhang Y., Hamers R. J., Jin S. (2018). Adv. Energy Mater..

[cit898] Bo X., Hocking R. K., Zhou S., Li Y., Chen X., Zhuang J., Du Y., Zhao C. (2020). Energy Environ. Sci..

[cit899] Jiang J., Sun F., Zhou S., Hu W., Zhang H., Dong J., Jiang Z., Zhao J., Li J., Yan W., Wang M. (2018). Nat. Commun..

[cit900] Qin F., Zhao Z., Alam M. K., Ni Y., Robles-Hernandez F., Yu L., Chen S., Ren Z., Wang Z., Bao J. (2018). ACS Energy Lett..

[cit901] Xu H., Wang B., Shan C., Xi P., Liu W., Tang Y. (2018). ACS Appl. Mater. Interfaces.

[cit902] Lu Z., Qian L., Tian Y., Li Y., Sun X., Duan X. (2016). Chem. Commun..

[cit903] Stevens M. B., Enman L. J., Korkus E. H., Zaffran J., Trang C. D.-M., Asbury J., Kast M. G., Toroker M. C., Boettcher S. W. (2019). Nano Res..

[cit904] Smith R. D., Prévot M. S., Fagan R. D., Trudel S., Berlinguette C. P. (2013). J. Am. Chem. Soc..

[cit905] Zhu X., Tang C., Wang H.-F., Li B.-Q., Zhang Q., Li C., Yang C., Wei F. (2016). J. Mater. Chem. A.

[cit906] Zhang B., Wang L., Cao Z., Kozlov S. M., García de Arquer F. P., Dinh C. T., Li J., Wang Z., Zheng X., Zhang L. (2020). et al.. Nat. Catal..

[cit907] Luo J. S., Im J. H., Mayer M. T., Schreier M., Nazeeruddin M. K., Park N. G., Tilley S. D., Fan H. J., Grätzel M. (2014). Science.

[cit908] Stoerzinger K. A., Qiao L., Biegalski M. D., Shao-Horn Y. (2014). J. Phys. Chem. Lett..

[cit909] Ng J. W.-D., Garca-Melchor M., Bajdich M., Chakthranont P., Kirk C., Vojvodic A., Jaramillo T. F. (2016). Nat. Energy.

[cit910] Gong M., Zhou W., Tsai M.-C., Zhou J., Guan M., Lin M.-C., Zhang B., Hu Y., Wang D.-Y., Yang J. (2014). Nat. Commun..

[cit911] Martindale B. C.-M., Reisner E. (2016). Adv. Energy Mater..

[cit912] Tang C., Cheng N., Pu Z., Xing W., Sun X. (2015). Angew. Chem., Int. Ed..

[cit913] Hou D., Zhou W., Liu X., Zhou K., Xie J., Li G. (2015). Electrochim. Acta.

[cit914] Liu D., Lu Q., Sun X., Asiri A. M. (2015). Nanoscale.

[cit915] You B., Sun Y. (2016). ChemPlusChem.

[cit916] Anantharaj S., Ede S. R., Sakthikumar K., Karthick K., Mishra S., Kundu S. (2016). ACS Catal..

[cit917] Tiwari A. P., G. novak T., Bu X., Ho J. C., Jeon S. (2018). Catalysts.

[cit918] Chen W.-F., Muckerman J. T., Fujita E. (2013). Chem. Commun..

[cit919] Michalsky R., Zhang Y.-J., Peterson A. A. (2014). ACS Catal..

[cit920] Li X., Hao X., Abdula A., Guan G. (2016). J. Mater. Chem. A.

[cit921] Kaneti Y. V., Tang J., Salunkhe R. R., Jiang X., Yu A., Wu K. C.-W., Yamauchi Y. (2017). Adv. Mater..

[cit922] Zhang Y., Zhou Q., Zhu J., Yan Q., Dou S. X., Sun W. (2017). Adv. Funct. Mater..

[cit923] Indra A., Song T., Paik U. (2018). Adv. Mater..

[cit924] Huang J., Jiang Y., An T., Cao M. (2020). J. Mater. Chem. A.

[cit925] Yu Z.-Y., Duan Y., Feng X.-Y., Yu X., Gao M.-R., Yu S.-H. (2021). Adv. Mater..

[cit926] Morales-Guio C. G., Stern L.-A., Hu X. (2014). Chem. Soc. Rev..

[cit927] Faber M. S., Jin S. (2014). Energy Environ. Sci..

[cit928] Kibsgaard J., Tsai C., Chan K., Benck J. D., Nørskov J. K., Abild-Pedersen F., Jaramillo T. F. (2015). Energy Environ. Sci..

[cit929] Paul R., Buisson P., Joseph N. (1952). Ind. Eng. Chem..

[cit930] Srinivasan S. , Lu P. W. T., Kissel G., Kulesa F. and Orehotsky J., Report, Brookhaven Natl. Lab., Upton, NY, USA, BNL-25211 (1978) 4

[cit931] Los P., Lasia A. (1992). J. Electroanal. Chem..

[cit932] Zeng M., Wang H., Zhao C., Wei J., Qi K., Wang W., Bai X. (2016). ChemCatChem.

[cit933] Zhang P., Wang M., Yang Y., Yao T., Han H., Sun L. (2016). Nano Energy.

[cit934] Chen X., Yu Z., Wei L., Zhou Z., Zhai S., Chen J., Wang Y., Huang Q., Karahan H. E., Liao X., Chen Y. (2019). J. Mater. Chem A.

[cit935] Xu X., Deng Y., Gu M., Sun B., Liang Z., Xue Y., Guo Y., Tian J., Cui H. (2019). Appl. Surf. Sci..

[cit936] Lao J., Li D., Jiang C., Luo R., Peng H., Qi R., Lin H., Huang R., Waterhouse G. I.-N., Luo C. (2020). Int. J. Hydrogen Energy.

[cit937] Han C., Li W., Wang J., Huang Z. (2022). Nano Res..

[cit938] Vrubel H., Hu X. (2012). Angew. Chem., Int. Ed..

[cit939] Subbaraman R., Tripkovic D., Strmcnik D., Chang K. C., Uchimura M., Paulikas A. P., Stamenkovic V., Markovic N. M. (2011). Science.

[cit940] Wirth S., Harnisch F., Weinmann M., Schröder U. (2012). Appl. Catal., B.

[cit941] Gupta S., Patel N., Miotello A., Kothari D. C. (2015). J. Power Sources.

[cit942] Masa J., Weide P., Peeters D., Sinev I., Sun Z., Somsen C., Muhler M., Schuhmann W. (2016). Adv. Energy. Mater..

[cit943] Lu W., Liu T., Xie L., Tang C., Liu D., Hao S., Qu F., Du G., Ma Y., Asiri A. M., Sun X. (2017). Small.

[cit944] Zhuang Z., Li Y., Li Z., Lv F., Lang Z., Zhao K., Zhou L., Moskaleva L., Guo S., Mai L. (2018). Angew. Chem., Int. Ed..

[cit945] Park H., Lee E., Lei M., Joo H., Coh S., Fokwa B. P.-T. (2020). Adv. Mater..

[cit946] Dutta S., Han H., Je M., Choi H., Kwon J., Park K., Indra A., Kim K. M., Paik U., Song T. (2020). Nano Energy.

[cit947] Gupta S., Patel N., Fernandes R., Kadrekar R., Dashora A., Yadav A. K., Bhattacharyya D., Jha S. N., Miotello A., Kothari D. C. (2016). Appl. Catal., B.

[cit948] Chen L., Zhang L.-R., Yao L.-Y., Fang Y.-H., He L., Wei G.-F., Liu Z.-P. (2019). Energy Environ. Sci..

[cit949] Chen D., Liu T., Wang P., Zhao J., Zhang C., Cheng R., Li W., Ji P., Pu Z., Mu S. (2020). ACS Energy Lett..

[cit950] Böhm H., Pohl F. A. (1968). Wiss. Ber. AEG-Telefunken.

[cit951] Bennet L. H., Cuthill J. R., McAlister A. J., Erickson N. E., Watson R. E. (1974). Science.

[cit952] Böhm H. (1970). Electrochim. Acta.

[cit953] Levy R. B., Boudart M. (1973). Science.

[cit954] Scanlon M. D., Bian X., Vrubel H., Amstutz V., Schenk K., Hu X., Liu B. H., Girault H. H. (2013). Phys. Chem. Chem. Phys..

[cit955] Sokolsky D. V., Palanker V. S., Baybatyrov E. N. (1975). Electrochim. Acta.

[cit956] Ross P. N., Stoneheart P. (1975). J. Catal..

[cit957] Palanker V. S., Sokolsky D. V., Mazulevsky E. A., Baybatyrov E. N. (1976). J. Power Sources.

[cit958] Ross P. N., Stoneheart P. (1977). J. Catal..

[cit959] Palanker V. S., Gajyev R. A., Sokolsky D. V. (1977). Electrochim. Acta.

[cit960] Oyama S. T. (1992). Catal. Today.

[cit961] Nikolov I., Vitanov T., Nikolova V. (1980). J. Power Sources.

[cit962] Ma C. A., Zhang W. K., Chen D. H., Zhou B. X. (2002). Trans. Nonferrous Met. Soc. China.

[cit963] Mathews N. R., Miller E. L., Sebastian P. J., Hernandez M. M., Mathew X., Gambo S. A. (2004). Int. J. Hydrogen Energy.

[cit964] Wu M., Shen P. K., Wie Z., Song S., Nie M. (2007). J. Power Sources.

[cit965] Ma C., Sheng J., Brandon N., Zhang C., Li G. (2007). Int. J. Hydrogen Energy.

[cit966] Harnisch F., Sievers G., Schröder U. (2009). Appl. Catal., B.

[cit967] Ferri T., Gozzi D., Latini A. (2007). Int. J. Hydrogen Energy.

[cit968] Meyer S., Nikiforov A. V., Petrushina I. M., Köhler K., Christensen E., Jensen J. O., Bjerrum N. J. (2015). Int. J. Hydrogen Energy.

[cit969] Fan X., Peng Z., Ye R., Zhou H., Guo X. (2015). ACS Nano.

[cit970] Syugaev A. V., Lyalina N. V., Lomayeva S. F., Maratkanova A. N. (2016). J. Solid State Electrochem..

[cit971] Brar L. K., Gupta A., Pandey O. P. (2019). Catal. Today.

[cit972] Esposito D. V., Hunt S. T., Stottlemyer A. L., Dobson K. D., McCandless B. E., Birkmire R. W., Chen J. G. (2010). Angew. Chem., Int. Ed..

[cit973] Esposito D. V., Chen J. G. (2011). Energy Environ. Sci..

[cit974] Esposito D. V., Hunt S. T., Kimmel Y. C., Chen J. G. (2012). J. Am. Chem. Soc..

[cit975] Yan Y., Xia B., Qi X., Wang H., Xu R., Wang J.-Y., Zhang H., Wang X. (2013). Chem. Commun..

[cit976] Hunt S. T., Nimmanwudipong T., Roman-Leshkov Y. (2014). Angew. Chem., Int. Ed..

[cit977] Fan X., Zhou H., Guo X. (2015). ACS Nano.

[cit978] Sljukic B., Vujkovic M., Amaral L., Santos D. M.-F., Rocha R. P., Sequeira C. A.-C., Figueiredo J. L. (2015). J. Mater. Chem. A.

[cit979] Wu H. B., Xia B. Y., Yu L., Yu X.-Y., Lou X. W. (2015). Nat. Commun..

[cit980] Xiong J., Li J., Shi J., Zhang X., Suen N.-T., Liu Z., Huang Y., Xu G., Cai W., Lei X., Feng L., Yang Z., Huang L., Cheng H. (2018). ACS Energy Lett..

[cit981] Harnisch F., Schröder U., Quaas M., Scholz F. (2009). Appl. Catal., B.

[cit982] Pan L. F., Li Y. H., Yang S., Liu P. F., Yu M. Q., Yang H. G. (2014). Chem. Commun..

[cit983] Liao L., Wang S., Xiao J., Bian X., Zhang Y., Scanlon M. D., Hu X., Tang Y., Liu B., Girault H. H. (2014). Energy Environ. Sci..

[cit984] Alhajri N. S., Anjum D. H., Takanabe K. (2014). J. Mater. Chem. A.

[cit985] Youn D. H., Han S., Kim J. Y., Kim J. Y., Park H., Choi S. H., Lee J. S. (2014). ACS Nano.

[cit986] Wan C., Regmi Y. N., Leonard B. M. (2014). Angew. Chem., Int. Ed..

[cit987] Xu X., Nosheen F., Wang X. (2016). Chem. Mater..

[cit988] Liu Y., Yu G., Li G.-D., Sun Y., Asefa T., Chen W., Zou X. (2015). Angew. Chem., Int. Ed..

[cit989] Lin H., Liu N., Shi Z., Guo Y., Tang Y., Gao Q. (2016). Adv. Funct. Mater..

[cit990] Wu Z.-Y., Hu B.-C., Wu P., Liang H.-W., Yu Z.-L., Lin Y., Zheng Y.-R., Li Z., Yu S.-H. (2016). NPG Asia Mater..

[cit991] Yu Z.-Y., Duan Y., Gao M.-R., Lang C.-C., Zheng Y.-R., Yu S.-H. (2017). Chem. Sci..

[cit992] Lu C., Tranca D., Zhang J., Hernandez F. R., Su Y., Zhuang X., Zhang F., Seifert G., Feng X. (2017). ACS Nano.

[cit993] Zu M. Y., Liu P. F., Wang C., Wang Y., Zheng L. R., Zhang B., Zhao H., Yang H. G. (2018). ACS Energy Lett..

[cit994] Yan H., Xie Y., Jiao Y., Wu A., Tian C., Zhang X., Wang L., Fu H. (2018). Adv. Mater..

[cit995] Yu F., Gao Y., Lang Z., Ma Y., Yin L., Du J., Tan H., Wang Y., Li Y. (2018). Nanoscale.

[cit996] Jin H., Chen J., Mao S., Wang Y. (2018). ACS Appl. Mater. Interfaces.

[cit997] Cui T., Dong J., Pan X., Yu T., Fu Q., Bao X. (2019). J. Energy Chem..

[cit998] Najafi L., Bellani S., Oropesa-Nunez R., Prato M., Martín-García B., Brescia R., Bonaccorso F. (2019). ACS Nano.

[cit999] Lu X. F., Yu L., Zhang J., Lou X. W. (2019). Adv. Mater..

[cit1000] Ma Y., Chen M., Geng H., Dong H., Wu P., Li X., Guan G., Wang T. (2020). Adv. Funct. Mater..

[cit1001] Vikraman D., Hussain S., Karuppasamy K., Feroze A., Kathalingam A., Sanmugam A., Chun S.-H., Jung J., Kim H.-S. (2020). Appl. Catal., B.

[cit1002] Humagain G., MacDougal K., MacInnis J., Lowe J. M., Coridan R. H., MacQuarrie S., Dasog M. (2018). Adv. Energy Mater..

[cit1003] Wakisaka T., Kusada K., Wu D., Yamamoto T., Toriyama T., Matsumura S., Akiba H., Yamamuro O., Ikeda K., Otomo T. (2020). et al.. J. Am. Chem. Soc..

[cit1004] Kang J. S., Kim J., Lee M. J., Son Y. J., Chung D. Y., Park S., Jeong J., Yoo J. M., Shin H., Choe H., Park H. S., Sung Y.-E. (2018). Adv. Sci..

[cit1005] Shrestha A., Gao X., Hicks J. C., Paoluci C. (2021). Chem. Mater..

[cit1006] Gauthier J. A., King L. A., Stults F. T., Flores R. A., Kibsgaard J., Regmi Y. N., Chan K., Jaramillo T. F. (2019). J. Phys. Chem C.

[cit1007] Chakrapani V., Thangala J., Sunkara M. K. (2009). Int. J. Hydrogen Energy.

[cit1008] Shi J., Pu Z., Liu Q., Asiri A. M., Hu J., Sun X. (2015). Electrochim. Acta.

[cit1009] Zhang Y., Ouyang B., Xu J., Jia G., Chen S., Rawat R. S., Fan H. J. (2016). Angew. Chem., Int. Ed..

[cit1010] Crane E. L., Chiu H.-T., Nuzzo R. G. (2001). Phys. Chem. B.

[cit1011] Han Y., Yue X., Jin Y., Huang X., Shen P. K. (2016). J. Mater. Chem. A.

[cit1012] Kang Y.-S., Yong Y.-J., Lee P. S., Lee J.-Y. (2001). J. Electrochem. Soc..

[cit1013] Rosetolato D., Battaglin G., Ferro S. (2014). Electrochem. Commun..

[cit1014] Chen J. G. (1996). Chem. Rev..

[cit1015] Schlatter J. C., Oyama S. T., Metcalfe J. E., Lambert J. M. (1988). Ind. Eng. Chem. Res..

[cit1016] Choi J.-G., Brenner J. R., Colling C. W., Demczyk B. G., Dunning J. L., Thompson L. T. (1992). Catal. Today.

[cit1017] Kida T., Minami Y., Guan G., Nagano N., Akiyama M., Yoshida A. (2006). J. Mater. Sci..

[cit1018] Ma S. S.-K., Hisatomi T., Maeda K., Moriya Y., Domen K. (2012). J. Am. Chem. Soc..

[cit1019] Chen P., Xu K., Fang Z., Tong Y., Wu J., Lu X., Peng X., Ding H., Wu C., Xie Y. (2015). Angew. Chem., Int. Ed..

[cit1020] Yu F., Zhou H., Zhu Z., Sun J., He R., Bao J., Chen S., Ren Z. (2017). ACS Catal..

[cit1021] Xie J., Xie Y. (2016). Chem. – Eur. J..

[cit1022] Zhong Y., Xia X., Shi F., Zhan J., Tu J., Fan H. J. (2016). Adv. Sci..

[cit1023] Balogun M.-S., Huang Y., Qiu W., Yang H., Ji H., Tong Y. (2017). Mater. Today.

[cit1024] Han N., Liu P., Jiang J., Ai L., Shao Z., Liu S. (2018). J. Mater. Chem. A.

[cit1025] Theerthagiri J., Lee S. J., Murthy A. P., Madhavan J., Choi M. Y. (2020). Curr. Opin. Solid State Mater. Sci..

[cit1026] Jin H., Liu X., Vasileff A., Jiao Y., Zhao Y., Zheng Y., Qiao S.-Z. (2018). ACS Nano.

[cit1027] Ramesh R., Nandi D. K., Kim T. H., Cheon T., Oh J., Kim S.-H. (2019). ACS Appl. Mater. Interfaces.

[cit1028] Kozejova M., Latyshev V., Kavecansky V., You H., Vorobiov S., Kovalcikova A., Komanicky V. (2019). Electrochim. Acta.

[cit1029] Ramesh R., Sawant S. Y., Nandi D. K., Hyun Kim T., Kim D. H., Han S.-M., Jang Y., Ha M. G., Cho M. H., Yoon T., Kim S.-H. (2020). ChemSusChem.

[cit1030] Cao B., Veith G. M., Neuefeind J. C., Adzic R. R., Khalifah P. G. (2013). J. Am. Chem. Soc..

[cit1031] Lin F., Dong Z., Yao Y., Yang L., Fang F., Jiao L. (2020). Adv. Energy Mater..

[cit1032] Xie J., Li S., Zhang X., Zhang J., Wang R., Zhang H., Pan B., Xie Y. (2014). Chem. Sci..

[cit1033] Ma L., Ting L. R.-L., Molinari V., Giordano C., Siang Yeo B. (2015). J. Mater. Chem. A.

[cit1034] Shalom M., Ressnig D., Yang X., Clavel G., Fellinger T. P., Antonietti M. (2015). J. Mater. Chem. A.

[cit1035] Gao D., Zhang J., Wang T., Xiao W., Tao K., Xue D., Ding J. (2016). J. Mater. Chem. A.

[cit1036] Zhang Q., Wang Y., Wang Y., Al-Enizi A. M., Elzatahry A. A., Zheng G. (2016). J. Mater. Chem. A.

[cit1037] Xing Z., Li Q., Wang D., Yang X., Sun X. (2016). Electrochim. Acta.

[cit1038] Zhang Y., Ouyang B., Xu J., Chen S., Rawat R. S., Fan H. J. (2016). Adv. Energy Mater..

[cit1039] Wang Y. Y., Xie C., Liu D. D., Huang X. B., Huo J., Wang S. Y. (2016). ACS Appl. Mater. Interfaces.

[cit1040] Lai J., Huang B., Chao Y., Chen X., Guo S. (2019). Adv. Mater..

[cit1041] Zhou P., Xing D., Liu Y., Wang Z., Wang P., Zheng Z., Qin X., Zhang X., Dai Y., Huang B. (2019). J. Mater. Chem. A.

[cit1042] Park S. H., Jo T. H., Lee M. H., Kawashima K., Buddie Mullins C., Lim H.-K., Youn D. H. (2021). J. Mater. Chem. A.

[cit1043] Xiang J., Zou W., Tang H. (2021). Catal. Sci. Technol..

[cit1044] Yu L., Zhu Q., Song S., McElhenny B., Wang D., Wu C., Qin Z., Bao J., Yu Y., Chen S., Ren Z. (2019). Nat. Commun..

[cit1045] Kuttiyiel K. A., Sasaki K., chen W.-F., Su D., Adzic R. R. (2014). J. Mater. Chem. A.

[cit1046] Uosaki K., Elumalai G., Noguchi H., Masuda T., Lyalin A., Nakayama A., Taketsugu T. (2014). J. Am. Chem. Soc..

[cit1047] Ma R., Bando Y., Zhu H., Sato T., Xu C., Wu D. (2002). J. Am. Chem. Soc..

[cit1048] Uosaki K., Elumalai G., Dinh H. C., Lyalin A., Taketsugu T., Noguchi H. (2016). Sci. Rep..

[cit1049] Callejas J. F., Read C. G., Roske C. W., Lewis N. S., Schaak R. E. (2016). Chem. Mater..

[cit1050] Du H., Kong R.-M., Guo X., Qu F., Li J. (2018). Nanoscale.

[cit1051] Owens-Baird B., Kolenko Y. V., Kovnir K. (2018). Chem. – Eur. J..

[cit1052] Weng C.-C., Ren J.-T., Yuan Z.-Y. (2020). ChemSusChem.

[cit1053] El-Refaei S. M., Russo P. A., Pinna N. (2021). ACS Appl. Mater. Interfaces.

[cit1054] Wearden T. (1974). Electron. Power.

[cit1055] Krishnakumari M. K., Muktha Bai K., Majumder S. K. (1980). Bull. Environ. Contam. Toxicol..

[cit1056] Nakato Y., Ohnishi T., Tsubomura H. (1975). Chem. Lett..

[cit1057] Nakato Y., Tonomura S., Tsubomura H. (1976). Ber. Bunsenges. Phys. Chem..

[cit1058] Paseka I. (1995). Electrochim. Acta.

[cit1059] Burchardt T. (2000). Int. J. Hydrogen Energy.

[cit1060] Liu P., Rodriguez J. A. (2005). J. Am. Chem. Soc..

[cit1061] Senevirathne K., Burns A. W., Bussell M. E., Brock S. L. (2007). Adv. Funct. Mater..

[cit1062] Feng L., Vrubel H., Bensimon M., Hu X. (2014). Phys. Chem. Chem. Phys..

[cit1063] Pan Y., Liu Y., Zhao J., Yang K., Liang J., Liu D., Hu W., Liu D., Liu Y., Liu C. (2015). J. Mater. Chem. A.

[cit1064] Callejas J. F., Read C. G., Popczun E. J., McEnaney J. M., Schaak R. E. (2015). Chem. Mater..

[cit1065] Stein B. F., Walmsley R. H. (1966). Phys. Rev..

[cit1066] Ripley R. L. (1962). J. Less-Common Met..

[cit1067] Oyama S. T. (2003). J. Catal..

[cit1068] Kovnira K. A., Kolen’ko Y. V., Ray S., Lib J., Watanabe T., Itoh M., Yoshimura M., Shevelkov A. V. (2006). Solid State Chem..

[cit1069] Perera S. C., Tsoi G., Wenger L. E., Brock S. L. (2003). J. Am. Chem. Soc..

[cit1070] Motojima S., Haguri K., Takahashi Y., Sugiyama K. (1979). J. Less-Common Met..

[cit1071] Biefeld R. M. (1982). J. Cryst. Growth.

[cit1072] Schrey F., Boone T., Nakahara S., Robbins M., Appelbaum A. (1987). Thin Solid Films.

[cit1073] Tian J., Liu Q., Asiri A. M., Sun X. (2014). J. Am. Chem. Soc..

[cit1074] Liu Q., Tian J., Cui W., Jiang P., Cheng N., Asiri A. M., Sun X. (2014). Angew. Chem., Int. Ed..

[cit1075] Deng J., Ren P., Deng D., Bao X. (2015). Angew. Chem., Int. Ed..

[cit1076] Das D., Nanda K. K. (2016). Nano Energy.

[cit1077] Liang H., Gandi A. N., Anjum D. H., Wang X., Schwingenschlögl U., Alshareef H. N. (2016). Nano Lett..

[cit1078] Kibsgaard J., Jaramillo T. F. (2014). Angew. Chem., Int. Ed..

[cit1079] Read C. G., Callejas J. F., Holder C. F., Schaak R. E. (2016). ACS Appl. Mater. Interfaces.

[cit1080] Laursen A. B., Patraju K. R., Whitaker M. J., Retuerto M., Sarkar T., Yao N., Ramanujachary K. V., Greenblatt M., Dismukes G. C. (2015). Energy Environ. Sci..

[cit1081] Li J., Li J., Zhou X., Xia Z., Gao W., Ma Y., Qu Y. (2016). ACS Appl. Mater. Interfaces.

[cit1082] Wang C., Ding T., Sun Y., Zhou X., Liu Y., Yang Q. (2015). Nanoscale.

[cit1083] Kucernak A. R.-J., Naranammalpuram Sundaram V. N. (2014). J. Mater. Chem. A.

[cit1084] Popczun E. J., Read C. G., Roske C. W., Lewis N. S., Schaak R. E. (2014). Angew. Chem., Int. Ed..

[cit1085] Popczun E. J., Roske C. W., Read C. G., Crompton J. C., McEnaney J. M., Callejas J. F., Lewis N. S., Schaak R. E. (2015). J. Mater. Chem. A.

[cit1086] Ryu J., Jung N., Jang J. H., Kim H. J., Yoo S. J. (2015). ACS Catal..

[cit1087] Li Q., Xing Z., Asiri A. M., Jiang P., Sun X. (2014). Int. J. Hydrogen Energy.

[cit1088] Yang X., Lu A. Y., Zhu Y., Hedhili M. N., Min S., Huang K., Han Y., Li L. (2015). Nano Energy.

[cit1089] Pan Y., Lin Y., Chen Y., Liu Y., Liu C. (2016). J. Mater. Chem. A.

[cit1090] Wang J., Yang W., Liu J. (2016). J. Mater. Chem. A.

[cit1091] Huang Z., Chen Z., Chen Z., Lv C., Humphrey M. G., Zhang C. (2014). Nano Energy.

[cit1092] McEnaney J. M., Crompton J. C., Callejas J. F., Popczun E. J., Biacchi A. J., Lewis N. S., Schaak R. E. (2014). Chem. Mater..

[cit1093] Xiao P., Sk M. A., Thia L., Ge X., Lim R. J., Wang J. Y. (2014). Energy Environ. Sci..

[cit1094] Pu Z., Saana Amiinu I., Wang M., Yang Y., Mu S. (2016). Nanoscale.

[cit1095] McEnaney J. M., Chance Crompton J., Callejas J. F., Popczun E. J., Read C. G., Lewis N. S., Schaak R. E. (2014). Chem. Commun..

[cit1096] Pu Z., Liu Q., Asiri A. M., Sun X. (2014). ACS Appl. Mater. Interfaces.

[cit1097] Xing Z., Liu Q., Asiri A. M., Sun X. (2015). ACS Catal..

[cit1098] Du H., Gu S., Liu R., Li C. M. (2015). J. Power Sources.

[cit1099] Callejas J. F., McEnaney J. M., Read C. G., Crompton J. C., Biacchi A. J., Popczun E. J., Gordon T. R., Lewis N. S., Schaak R. E. (2014). ACS Nano.

[cit1100] Son C. Y., Kwak I. H., Lim Y. R., Park J. (2016). Chem. Commun..

[cit1101] Liu R., Gu S., Du H., Li C. M. (2014). J. Mater. Chem. A.

[cit1102] Tian J., Liu Q., Liang Y., Xing Z., Asiri A. M., Sun X. (2014). ACS Appl. Mater. Interfaces.

[cit1103] Zhang Z., Hao J., Yang W., Lu B., Tang J. (2015). Nanoscale.

[cit1104] Yang X., Lu A. Y., Zhu Y., Min S., Hedhili M. N., Han Y., Huang K. W., Li L. (2015). Nanoscale.

[cit1105] Zhang Y., Zhang H., Feng Y., Liu L., Wang Y. (2015). ACS Appl. Mater. Interfaces.

[cit1106] Jiang J., Wang C., Zhang J., Wang W., Zhou X., Pan B., Tang K., Zuo J., Yang Q. (2015). J. Mater. Chem. A.

[cit1107] Tian J., Liu Q., Cheng N., Asiri A. M., Sun X. (2014). Angew. Chem., Int. Ed..

[cit1108] Liang Y., Liu Q., Asiri A. M., Sun X., Luo Y. (2014). ACS Catal..

[cit1109] Li Y., Zhang H., Jiang M., Zhang Q., He P., Sun X. M. (2017). Adv. Funct. Mater..

[cit1110] Guan C., Xiao W., Wu H. J., Liu X. M., Zhang W. J., Zhang H., Ding J., Feng Y. P., Pennycook S. J., Wang J. (2018). Nano Energy.

[cit1111] Lu S. S., Zhang L. M., Dong Y. M., Zhang J. Q., Yan X. T., Sun D. F., Shang X., Chi J. Q., Chai Y. M., Dong B. (2019). J. Mater. Chem. A.

[cit1112] Zhang T., Yang K., Wang C., Li S. Y., Zhang Q. Q., Chang X. J., Li J. T., Li S. M., JiaJ. S. F., Wang B., Fu L. (2018). Adv. Energy Mater..

[cit1113] King L. A., McKenzie A. H., Capuano C., Manco J., Danilovic N., Valle E., Hellstern T. R., Ayers K., Jaramillo T. F. (2019). Nat. Nanotechnol..

[cit1114] Xin H., Dai Z., Zhao Y., Guo S., Sun J., Luo Q., Zhang P., Sun L., Ogiwara N., Kitagawa H., Huang B., Ma F. (2021). Appl. Catal., B.

[cit1115] Riyajuddin S., Azmi K., Pahuja M., Kumar S., Maruyama T., Bera C., Ghosh K. (2021). ACS Nano.

[cit1116] Cao E., Chen Z., Wu H., Yu P., Wang Y., Xiao F., Chen S., Du S., Xie Y., Wu Y., Ren Z. (2020). Angew. Chem., Int. Ed..

[cit1117] Zhang Y., Li N., Zhang Z., Li S., Cui M., Ma L., Zhou H., Su D., Zhang S. (2020). J. Am. Chem. Soc..

[cit1118] Wu L., Yu L., Zhang F., McElhenny B., Luo D., Karim A., Chen S., Ren Z. (2021). Adv. Funct. Mater..

[cit1119] Duan J., Chen S., Ortiz-Ledon C. A., Jaroniec M., Qiao S.-Z. (2020). Angew. Chem., Int. Ed..

[cit1120] Han J. H., Kwak M., Kim Y., Cheon J. (2018). Chem. Rev..

[cit1121] Pumera M., Sofer Z., Ambrosi A. (2014). J. Mater. Chem. A.

[cit1122] Zhang G., Liu H., Qu J., Li J. (2016). Energy Environ. Sci..

[cit1123] Anatharaj S., Kundu S., Noda S. (2020). J. Mater. Chem. A.

[cit1124] Giri A., Park G., Yang H., Pal M., Kwak J., Jeong U. (2018). Adv. Mater..

[cit1125] Maurya O., Khaladkar S., Horn M. R., Sinha B., Deshmukh R., Wang H., Kim T. Y., Dubal D. P., Kalekar A. (2021). Small.

[cit1126] Guo Y., Park T., Yi J. W., Henzie J., Kim J., Wang Z., Jiang B., Bando Y., Sugahara Y., Tang J., Yamauchi Y. (2019). Adv. Mater..

[cit1127] Lembke D., Bertolazzi S., Kis A. (2015). Acc. Chem. Res..

[cit1128] Coleman J. N., Lotya M., O’Neill A., Bergin S. D., King P. J., Khan U., Young K., Gaucher A., Ronan S. D., Smith J. (2011). et al.. Science.

[cit1129] Shifa T. A., Wang F., Liu K., Xu K., Wang Z., Zhan X., Jiang C., He J. (2016). Small.

[cit1130] Zhou K. G., Mao N. N., Wang H. X., Peng Y., Zhang H. L.-A. (2011). Angew. Chem., Int. Ed..

[cit1131] Feng J., Sun X., Wu C. Z., Peng L. L., Lin C. W., Hu S. L., Yang J. L., Xie Y. (2011). J. Am. Chem. Soc..

[cit1132] Wang C., He Q., Halim U., Liu Y., Zhu E., Lin Z., Xiao H., Duan X., Feng Z., Cheng R. (2018). Nature.

[cit1133] Jeong S., Yoo D., Jang J. T., Kim M., Cheon J. (2012). J. Am. Chem. Soc..

[cit1134] Harang H. et al. , Electrolyte Cell Active Cathode with Lov Overvoltage, Belgian Patent, No. 864275; Norsk Hydro, Oslo, Nederlands Patent, No 7801955, 1978

[cit1135] Yanagida S., Azuma T., Sakurai H. (1982). Chem. Lett..

[cit1136] Vandenborre H., Vermehren P., Leysen R. (1984). Electrochim. Acta.

[cit1137] Hinnemann B., Moses P. G., Bonde J., Jørgensen K. P., Nielsen J. H., Horch S., Chorkendorff I., Nørskov J. K. (2005). J. Am. Chem. Soc..

[cit1138] Huang Y., Nielsen R. J., Goddard W. A. (2018). J. Am. Chem. Soc..

[cit1139] Li H., Tsai C., Koh A. L., Cai L., Contryman A. W., Fragapane A. H., Zhao J., Han H. S., Manoharan H. C., Abild-Pedersen F., Norskov J. K., Zheng X. (2016). Nat. Mater..

[cit1140] Merki D., Fierro S., Vrubel H., Hu X. (2011). Chem. Sci..

[cit1141] Vrubel H., Merki D., Hu X. (2012). Energy Environ. Sci..

[cit1142] Gholamvand Z., McAteer D., Backes C., McEvoy N., Harvey A., Berner N. C., Hanlon D., Bradley C., Godwin I., Rovetta A., Lyons M. E.-G., Duesberg G. S., Coleman J. N. (2016). Nanoscale.

[cit1143] You B., Jiang N., Sun Y. (2016). Inorg. Chem. Front..

[cit1144] Feng L.-L., Li G.-D., Liu Y., Wu Y., Chen H., Wang Y., Zou Y.-C., Wang D., Zou X. (2015). ACS Appl. Mater. Interfaces.

[cit1145] Liu Q., Shi J., Hu J., Asiri A. M., Luo Y., Sun X. (2015). ACS Appl. Mater. Interfaces.

[cit1146] Masud J., Swesi A. T., Liyanage W. P.-R., Nath M. (2016). ACS Appl. Mater. Interfaces.

[cit1147] Jiang J., Gao M., Sheng W., Yan Y. (2016). Angew. Chem., Int. Ed..

[cit1148] Yu B., Qi F., Zheng B., Hou W., Zhang W., Li Y., Chen Y. (2018). J. Mater. Chem. A.

[cit1149] Zheng X., Han X., Zhang Y., Wang J., Zhong C., Deng Y., Hu W. (2019). Nanoscale.

[cit1150] Swesi A. T., Masud J., Nath M. (2016). Energy Environ. Sci..

[cit1151] Liu P. F., Zhang L., Zheng L. R., Yang H. G. (2018). Mater. Chem. Front..

[cit1152] Wang Y., Li X., Zhang M., Zhou Y., Rao D., Zhong C., Zhang J., Han X., Hu W., Zhang Y., Zaghib K., Wang Y., Deng Y. (2020). Adv. Mater..

[cit1153] Qu S., Huang J., Yu J., Chen G., Hu W., Yin M., Zhang R., Chu S., Li C. (2017). ACS Appl. Mater. Interfaces.

[cit1154] Bae C., Ho T. A., Kim H., Lee S., Lim S., Kim M., Yoo H., Montero-Moreno J. M., Park J. H., Shin H. (2017). Sci. Adv..

[cit1155] Di Giovanni C., Wang W.-A., Nowak S., Greneche J.-M., Lecoq H., Mouton L., Giraud M., Tard C. (2014). ACS Catal..

[cit1156] Giovanni C. D., Reyes-Carmona A., Coursier A., Nowak S., Greneche J. M., Lecoq H., Mouton L., Roziere J., Jones D., Peron J., Giraud M., Tard C. (2016). ACS Catal..

[cit1157] Faber M. S., Lukowski M. A., Ding Q., Kaiser N. S., Jin S. (2014). J. Phys. Chem. C.

[cit1158] Chanda D., Tufa R. A., Birdja Y. Y., Basu S., Liu S. (2020). Int. J. Hydrogen Energy.

[cit1159] Xu X., Ge Y., Wang M., Zhang Z., Dong P., Baines R., Ye M., Shen J. (2016). ACS Appl. Mater. Interfaces.

[cit1160] Sarkar S., Rawat A., Das T., Gaboardi M., Chakraborty S., Vinod C. P., Peter S. C. (2021). ChemSusChem.

[cit1161] Yin K., Cui Z. D., Zheng X. R., Yang X. J., Zhu S. L., Li Z. Y., Liang Y. Q. (2015). J. Mater. Chem. A.

[cit1162] Chia X., Ambrosi A., Lazar P., Sofer Z., Pumera M. (2016). J. Mater. Chem. A.

[cit1163] Yu Q., Zhang Z., Qiu S., Luo Y., Liu Z., Yang F., Liu H., Ge S., Zou X., Ding B., Ren W., Cheng H.-M., Sun C., Liu B. (2021). Nat. Commun..

[cit1164] Chia X., Sofer Z., Luxa J., Pumera M. (2017). Chem. – Eur. J..

[cit1165] McGlynn J. C., Dankwort T., Kienle L., Bandeira N. A. G., Fraser J. P., Gibson E. K., Cascallana-Matías I., Kamarás K., Symes M. D., Miras H. N., Ganin A. Y. (2019). Nat. Commun..

[cit1166] Merki D., Hu X. (2011). Energy Environ. Sci..

[cit1167] Wang X., Chen Y., Qi F., Zheng B., He J., Li Q., Li P., Zhang W., Li Y. (2016). Electrochem. Commun..

[cit1168] Sun Y., Zhang X., Mao B., Cao M. (2016). Chem. Commun..

[cit1169] Zou M., Chen J., Xiao L., Zhu H., Yang T., Zhang M., Du M. (2015). J. Mater. Chem. A.

[cit1170] Zou M., Zhang J., Zhu H., Du M., Wang Q., Zhang M., Zhang X. (2015). J. Mater. Chem. A.

[cit1171] Zhang L., Guo Y., Iqbal A., Li B., Gong D., Liu W., Iqbal K., Liu W., Qin W. (2018). Int. J. Hydrogen Energy.

[cit1172] Ji L., Zhu L., Wang J., Chen Z. (2017). Electrochim. Acta.

[cit1173] Zhu Y., Peng L., Zhu W., Akinwande D., Yu G. (2016). Chem. Mater..

[cit1174] Majhi K. C., Karfa P., Madhuri R. (2019). Electrochim. Acta.

[cit1175] Wang G., Chen W., Chen G., Huang J., Song C., Chen D., Du Y., Li C., Ostrikov K. K. (2020). Nano Energy.

[cit1176] Kwon H., Bae D., Won D., Kim H., Kim G., Cho J., Park H. J., Baik H., Jeong A.
R., Lin C.-H., Chiang C.-Y., Ku C.-S., Yang H., Cho S. (2021). ACS Nano.

[cit1177] Zhang S., Zhou Q., Shen Z., Jin X., Zhang Y., Shi M., Zhou J., Liu J., Lu Z., Zhou Y.-N., Zhang H. (2021). Adv. Funct. Mater..

[cit1178] Osei-Tutu Agyapong-Fordjour F., Yun S. J., Kim H.-J., Choi W., Kirubasankar B., Choim S. H., Adofo L. A., Boandoh S., Kim Y. I., Kim S. M., Kim Y.-M., Lee Y. H., Han Y.-K., Kim K. K. (2021). Adv. Sci..

[cit1179] Tong Y., Feng D., Chen P. (2021). ACS Sustainable Chem. Eng..

[cit1180] Zhai L., Lo T. W.-B., Xu Z.-L., Potter J., Mo J., Guo X., Tang C. C., Tsang S. C.-E., Lau S. P. (2020). ACS Energy Lett..

[cit1181] Zaho Y., Kamiya K., Hashimoto K., Nakanishi S. (2013). Angew. Chem., Int. Ed..

[cit1182] Chen W.-F., Schneider J. M., Sasaki K., Wang C.-H., Scheider J., Lyer S., Iyer S., Zhu Y., Muckerman J. T., Fujita E. (2014). ChemSusChem.

[cit1183] Zhuo J., Caban-Acevedo M., Liang H., Samad L., Ding Q., Fu Y., Li M., Jin S. (2015). ACS Catal..

[cit1184] Lu X. F., Zhang S. L., Sim W. L., Gao S., Wen X. (2021). Angew. Chem., Int. Ed..

[cit1185] Liu M., Wang J.-A., Klysubun W., Wang G.-G., Sattayaporn S., Li F., Cai Y.-W., Zhang F., Yu J., Yang Y. (2021). Nat. Commun..

[cit1186] Nguyen D. C., Tran D. T., Doan T. L.-L., Kim D. H., Kim N. H., Lee J. H. (2020). Adv. Energy Mater..

[cit1187] Liu C., Hu Y., Liu F., Liu H., Xu X., Xue Y., Zhang J., Li Y., Tang C. (2021). Int. J. Hydrogen Energy.

[cit1188] Liu C., Gong T., Zhang J., Zheng X., Mao J., Liu H., Li Y., Hao Q. (2020). Appl. Catal., B.

[cit1189] Asen P., Esfandiar A. (2021). Electrochim Acta.

[cit1190] Shen G. X., Chen Y. C., Lin C. J. (2005). Thin Solid Films.

[cit1191] Lu W. K., Elsenbaumer R. L., Wessling B. (1995). Synth. Met..

[cit1192] German Patent and Trademark Office: Patent of the German Empire Nr. 304126, issued on October 18, 1912

[cit1193] German Patent and Trademark Office: Patent of the German Empire Nr. 304159, issued on December 21, 1912

[cit1194] Leach J. S.-L., Saunders S. R.-J. (1966). J. Electrochem. Soc..

[cit1195] O’Brien R. N., Seto P. (1970). J. Electrochem. Soc..

[cit1196] Bicelli L. P., Romagnani C., Rosania M. T. (1976). J. Chim. Phys. Phys.-Chim. Biol..

[cit1197] Radhakrishnamurthy P., Sathyanarayana S., Reddy K. N. (1977). J. Appl. Electrochem..

[cit1198] Ateya B. G., Elnizamy F. M.-A. (1980). Corros. Sci..

[cit1199] Brown A. P., Krumpelt M., Loufty R. O., Yao N. P. (1982). Electrochim. Acta.

[cit1200] Gonzalez E. R., Avaca L. A., Carubelli A., Tanaka A. A., Tremiliosi-Filho G. (1984). Int. J. Hydrogen Energy.

[cit1201] Frankenthal R. P., Milner P. C. (1986). Corrosion.

[cit1202] Badea G. E., Maior I., Cojocaru A., Pantea I., Badea T. (2009). Rev. Roum. Chim..

[cit1203] Singh R. N., Pandey J. P., Anitha K. L. (1993). Int. J. Hydrogen Energy.

[cit1204] Dinamini M., Kamath P. V. (2000). J. Appl. Electrochem..

[cit1205] Varela H., Câmara G. A., Júnior H. S., Gonzalez E. R. (2000). Quím. Nova.

[cit1206] Elezovic N. R., Jovic V. D., Krstajic N. V. (2005). Electrochim. Acta.

[cit1207] Panek J., Antoni B. (2007). Surf. Coatings Technol..

[cit1208] Herraiz-Cardona I., Ortega E., Perez-Herranz V. (2011). Electrochim. Acta.

[cit1209] Leonard K. C., Tejedor-Anderson M. I., Anderson M. A. (2012). Int. J. Hydrogen.

[cit1210] Wang L., Huang X., Jiang S., Li M., Zhang K., Yan Y., Zhang H., Xue J. M. (2017). Appl. Mater. Interfaces.

[cit1211] Zhang H., de Souza e Silva J. M., Lu X., Santos de Oliveira C., Cui B., Li X., Lin C., Schweizer S. L., Maijenburg A. W., Bron M., Wehrspohn R. B. (2019). Adv. Mater. Interfaces.

[cit1212] Zhang H., de Souza e Silva J. M., de Oliveira C. S., Lu X., Schweizer S. L., Maijenburg A. W., Bron M., Wehrspohn R. B. (2020). MRS Adv..

[cit1213] Jothi V. R., Karuppasamy K., Maiyalagan T., Rajan H., Jung C.-Y., Yi S. C. (2020). Adv. Energy. Mater..

[cit1214] Gómez M. J., Diaz L. A., Franceschini E. A., Lacconi G. I., Abuin G. C. (2019). J. Appl. Electrochem..

[cit1215] Amrouche A., Messaoud F., Boutarek-Zaourar N., David P., Mossang E., Mansour S., Slimane M., Trari M. (2019). J. Solid State Electrochem..

[cit1216] Gomez M. J., Franceschini E. A., Lacconi G. I. (2018). Electrocatalysis.

[cit1217] Li H., He Y., He T., Shi H., Ma X., Zhang C., Yu H., Bai Y., Chen J., Luo P. (2021). Appl. Surf. Sci..

[cit1218] Zeng L., Zhao T. S., Zhang R. H., Xu J. B. (2018). Electrochem. Commun..

[cit1219] Zhu S., Duan G., Chang C., Chen Y., sun Y., Tang Y., Wan P., Pan J. (2020). ACS Sustainable Chem. Eng..

[cit1220] Youn J.-S., Jeong S., Oh I., Park S., Mai H. D., Jeon K.-J. (2020). Catalysts.

[cit1221] Zhang K., Liu Y., Wang B., Yu F., Yang Y., Xing L., Hao J., Zeng J., Mao B., Shi W., Yuan S. (2019). Int. J. Hydrogen Energy.

[cit1222] Tang D., Mabayoje O., Lai Y., Liu Y., Mullins C. B. (2017). ChemistrySelect.

[cit1223] Jadhav A. R., Puguan J. M.-C., Kim H. (2017). ACS Sustainable Chem. Eng..

[cit1224] Balram A., Zhang H., Santhanagopalan S. (2017). Mater. Chem. Front..

[cit1225] Hu C.-C., Wu Y.-R. (2003). Mater. Chem. Phys..

[cit1226] Qian L., Chen W., Huang R., Xiao D. (2015). RSC Adv..

[cit1227] Gu Y., Kim I., Nam Y. S. (2017). ChemCatChem.

[cit1228] Zhang Q., Zhong H., Meng F., Bao D., Zhang X., Wei X. (2018). Nano Res..

[cit1229] Xiao Y., Hu T., Zhao X., Hu F. X., Yang H. B., Li C. M. (2020). Nano Energy.

[cit1230] Barauskienė I., Valatka E. (2019). Electrocatalysis.

[cit1231] Maruthapandian V., Muthurasu A., Dekshinamoorthi A., Aswathy R., Vijayaraghavan S., Muralidharan S., Saraswathy V. (2019). ChemElectroChem.

[cit1232] Peng C., Huang R., Pan G., Liu W., Wang L. (2020). Ionics.

[cit1233] Gaward S. A., Nasr A., Fekry A. M., Filippov L. O. (2021). Int. J. Hydrogen Energy.

[cit1234] Edison T. N.-J. I., Atchudan R., Karthik N., Chandrasekaran S., Perumal S., Raja P. B., Perumal V., Lee Y. R. (2021). Fuels.

[cit1235] Wang H.-B., Zhu H., Sun Y. S., Ma F., Chen Y.-Z., Zeng D. J., Zhou L., Ma D.-Y. (2021). J. Alloys Comp..

[cit1236] Tan Q., Xiong T., Yang F., Huang P., Adekoya D., Huang Y., Balogun M.-S. (2021). J. Alloys Comp..

[cit1237] Hedenstedt K., Simic N., Wildlock M., Ahlberg E. (2016). J. Electroanal. Chem..

[cit1238] Yu F., Li F., Sun L. (2016). Int. J. Hydrogen Energy.

[cit1239] Schäfer H., Chevrier D. M., Zhang P., Stangl J., Müller-Buschbaum K., Hardege J. D., Kuepper K., Wollschläger J., Krupp U., Dühnen S., Steinhart M., Walder L., Sadaf S., Schmidt M. (2016). Adv. Funct. Mater..

[cit1240] Hu X., Tian X., Lin Y.-W., Wang Z. (2019). RSC Adv..

[cit1241] Yule L. C., Shkirskiy V., Aarons J., West G., Bentley C. L., Shollock B. A., Unwin P. R. (2019). J. Phys. Chem. C.

[cit1242] Li H., Xiao S., Zhou J., Zhao J., Liu F., Li G., Zhang D. (2019). Chem. Commun..

[cit1243] Chang C.-J., Lee Z., Wang C.-F. (2014). Int. J. Hydrogen.

[cit1244] Hsu M. H., Chang C. J. (2014). Int. J. Hydrogen.

[cit1245] Farrag H. H., Youssef Sayed S., Allam N. K., Mohammad A. M. (2020). J. Cleaner Prod..

[cit1246] Farrag H. H., Abbas A. A., Sayed S. Y., Alalawy H. H., El-Anadouli B. E., Mohammad A. M., Allam N. K. (2018). ACS Sustainable Chem. Eng..

[cit1247] Dlugosch T., Chnani A., Muralidhar P., Schirmer A., Biskupek J., Strehle S. (2017). Semicond. Sci. Technol..

[cit1248] Olivares-Ramirez J. M., Campos-Cornelio M. L., Uribe Godinez J., Borja-Arco E., Castellanos R. H. (2007). Int. J. Hydrogen Energy.

[cit1249] De Silva Munoz L., Bergel A., Feron D., Basseguy R. (2010). Int. J. Hydrogen.

[cit1250] Lavorante M. J., Franco J. I. (2016). Int. J. Hydrogen Energy.

[cit1251] Cherevko S., Topalov A. A., Zeradjanin A., Keeley G., Mayrhofer K. J.-J. (2014). Electrocatalysis.

[cit1252] Cherevko S., Zeradjanin A. R., Keeley G. P., Mayrhofer K. J.-J. (2014). J. Electrochem. Soc..

[cit1253] Topalov A. A., Cherevko S., Zeradjanin A. R., Meier J. C., Katsounaros I., Mayrhofer K. J.-J. (2014). Chem. Sci..

[cit1254] Topalov A. A., Zeradjanin A. R., Cherevko S., Mayrhofer K. J.-J. (2014). Electrochem. Commun..

[cit1255] Cherevko S., Keeley G. P., Geiger S., Zeradjanin A. R., Hodnik N., Kulyk N., Mayrhofer K. J.-J. (2015). ChemElectroChem.

[cit1256] Liu X., Gong M., Deng S., Zhao T., Shen T., Zhang J., Wang D. (2021). Adv. Funct. Mater..

[cit1257] Zhang C., Hong Y., Dai R., Lin X., Long L.-S., Wang C., Lin W. (2015). ACS Appl. Mater. Interfaces.

[cit1258] Van Dam H. E., Van Bekkum H. (1991). J. Catal..

[cit1259] Popczun E. J., McKone J. R., Read C. G., Biacchi A. J., Wiltrout A. M., Lewis N. S., Schaak R. E. (2013). J. Am. Chem. Soc..

[cit1260] Jin Z., Li P., Xiao D. (2016). Green Chem..

[cit1261] Menthe E., Bulak A., Olfe J., Zimmermann A., Rie K.-T. (2000). Surf. Coat. Technol..

[cit1262] Liu X., You B., Sun Y. (2017). ACS Sustainable Chem. Eng..

[cit1263] Balogun M.-S., Qiu W., Huang Y., Yang H., Xu R., Zhao W., Li G.-R., Ji H., Tong Y. (2017). Adv. Mater..

[cit1264] Yao M., Sun B., Wang N., Hu W., Komarneni S. (2019). Appl. Surf. Sci..

[cit1265] Allami S., Jalal N. M. (2019). Surf. Eng. Appl. Electrochem..

[cit1266] Anantharaj S., Chatterjee S., Swaathini K. C., Amarnath S., Subhashini E., Pattanayak D. K., Kundu S. (2018). ACS Sustainable Chem. Eng..

[cit1267] Lyu Y., Wang R., Tao L., Zou Y., Zhou H., Liu T., Zhou Y., Huo J., Jiang S. P., Zheng J., Wang S. (2019). Appl. Catal., B.

[cit1268] Gao Y., Xiong T., Li Y., Huang Y., Li Y., Balogun M.-S. (2019). ACS Omega.

[cit1269] Ring L., Pollet B. G., Chatenet M., Abbou S., Küpper K., Schmidt M., Huck M., Gries A., Steinhart M., Schäfer H. (2019). Angew. Chem., Int. Ed..

[cit1270] Sultana U. K., Fernando J. F.-S., O’Mullane A. P. (2020). Sust. Mater. Technol..

[cit1271] Anantharaj S., Sugime H., Noda S. (2020). ACS Appl. Energy Mater..

[cit1272] Kim M., Ha J., Shin N., Kim Y.-T., Choi J. (2020). Electrochim. Acta.

[cit1273] Koper M. (2011). J. Electroanal. Chem..

[cit1274] Hall D. E. (1982). J. Electrochem. Soc.: Electrochem. Sci. Technol..

[cit1275] Tiwari S. K., Singh A. K.-L., Singh R. N. (1991). J. Electroanal. Chem. Interfacial Electrochem..

[cit1276] Abreu C. M., Cristobal M. J., Losada R., Novoa X. R., Pena G., Perez M. C. (2006). Electrochim. Acta.

[cit1277] Moureaux F., Stevens P., Toussaint G., Chatenet M. (2013). J. Power Sources.

[cit1278] Lyons M. E.-G., Brandon M. P. (2010). J. Electroanal. Chem..

[cit1279] Louie M. W., Bell A. T. (2013). J. Am. Chem. Soc..

[cit1280] Todoroki N., Wadayama T. (2019). ACS Appl. Mater. Interfaces.

[cit1281] Schäfer H., Beladi-Mousavi S. M., Walder L., Wollschläger J., Kuschel O., Ichilmann S., Sadaf S., Steinhart M., Küpper K. (2015). ACS Catal..

[cit1282] Liu X., Shen K., Wang Y., Guo Y., Yong Z., Lu G. (2008). Catal. Commun..

[cit1283] Xanthopoulou G. (1999). Appl. Catal., A.

[cit1284] Marono M., Sanchez J. M., Ruiz E. (2010). Int. J. Hydrogen Energy.

[cit1285] Anantharaj S., Venkatesh M., Salunke A. S., Simha T. V.-S. H., Prabu V., Kundu S. (2017). ACS Sustainable Chem. Eng..

[cit1286] Schäfer H., Sadaf S., Walder L., Kuepper K., Dinklage S., Wollschläger J., Schneider L., Steinhart M., Hardege J., Daum D. (2015). Energy Environ. Sci..

[cit1287] Lee M., Jeon H. S., Lee S. Y., Kim H., Sim S. J., Hwang Y. J., Min J. (2017). J. Mater. Chem. A.

[cit1288] Schäfer H., Küpper K., Wollschlager J., Kashaev N., Hardege J., Walder L., Beladi-Mousavi S. M., Hartmann-Azanza B., Steinhart M., Sadaf S., Dorn F. (2015). ChemSusChem.

[cit1289] Todoroki N., Wadayama T. (2021). Electrochem. Commun..

[cit1290] Unmuth E. E., Schwartz L. H., Butt J. B. (1980). J. Catal..

[cit1291] Sitthisa S., An W., Resasco D. E. (2011). J. Catal..

[cit1292] Nie L., De Souza P. M., Noronha F. B., An W., Sooknoi T., Resasco D. E. (2014). J. Mol. Catal. A: Chem..

[cit1293] Wang L., Li D. L., Koike M., Koso S., Nakagawa Y., Xu Y., Tomishige K. (2011). Appl. Catal., A.

[cit1294] Wang J. G., Liu C. J., Zhang Y. P., Yu K. L., Zhu X. L., He F. (2004). Catal. Today.

[cit1295] Zhong H., Wang J., Meng F., Zhang X. (2016). Angew. Chem., Int. Ed..

[cit1296] Song S., Yu L., Xiao X., Qin Z., Zhang W., Wang D., Bao J., Zhou H., Zhang Q., Chen S., Ren Z. (2020). Mater. Today Phys..

[cit1297] Tian Z., Yang L., Wang Z., Xu C., Li D. (2021). Res. Chem. Intermed..

[cit1298] Kim M., Ha J., Kim Y.-T., Choi J. (2021). J. Mater. Chem. A.

[cit1299] Huang X., Chang S., Lee W. S.-V., Ding J., Xue J. M. (2017). J. Mater. Chem. A.

[cit1300] Rodrıguez Couto S., Sanroman M. A., Hofer D., Gubitz G. M. (2004). Bioresour. Technol..

[cit1301] Liu D., Zheng T., Buisman C., Ter Heijne A. (2017). ACS Sustainable Chem. Eng..

[cit1302] Chen J. S., Ren J., Shalom M., Fellinger T., Antonietti M. (2016). ACS Appl. Mater. Interfaces.

[cit1303] Zhu Y.-H., Yin Y.-B., Yang X., Sun T., Wang S., Jiang Y.-S., Yan J.-M., Zhang X. (2017). Angew. Chem., Int. Ed..

[cit1304] Han W., Kuepper K., Hou P., Akram W., Eickmeier H., Hardege J., Steinhart M., Schäfer H. (2018). ChemSusChem.

[cit1305] Zhang D., Kong X., Jiang M., Lei D., Lei X. (2019). ACS Sustainable Chem. Eng..

[cit1306] Zhu S., Chang C., Sun Y., Duan G., Chen Y., Pan J., Tang Y., Wan P. (2020). Int. J. Hydrogen Energy.

[cit1307] Zhang G.-R., Shen L.-L., Schmatz P., Krois K., Etzold B. J.-M. (2020). J. Energy Chem..

[cit1308] Yao M., Hu H., Wang N., Hu W., Komarneni S. (2020). J. Colloids Interface Sci..

[cit1309] Kanan M. W., Nocera D. G. (2008). Science.

[cit1310] Chen P., Xu K., Zhou T., Tong Y., Wu J., Cheng H., Lu X., Ding H., Wu C., Xie Y. (2016). Angew. Chem., Int. Ed..

[cit1311] He J., Peng Y., Sun Z., Cheng W., Liu Q., Feng Y., Jiang Y., Hu F., Pan Z., Bian Q. (2014). Electrochim. Acta.

[cit1312] Li S., Zhao K., Wang K., Yang M. (2017). Mater. Charact..

[cit1313] Cao A.-M., Hu J.-S., Liang H.-P., Song W.-G., Wan L.-J., He X.-L., Gao X.-G., Xia S.-H. (2006). J. Phys. Chem. B.

[cit1314] Li W., Xu L. N., Chen J. (2005). Adv. Funct. Mater..

[cit1315] Na C. W., Woo H. S., Kim I. D., Lee J. H. (2011). Chem. Commun..

[cit1316] Lou X. W., Deng D., Lee J. Y., Feng J., Archer L. A. (2008). Adv. Mater..

[cit1317] Xiong S., Yuan C., Zhang X., Xi B., Qian Y. (2009). Chem. – Eur. J..

[cit1318] Hamdani M., Singh R. N., Chartier P. (2010). Int. J. Electrochem. Sci..

[cit1319] Xu J., Gao P., Zhao T. S. (2012). Energy Environ. Sci..

[cit1320] Liang Y., Li Y., Wang H., Zhou J., Wang J., Regier T., Dai H. (2011). Nat. Mater..

[cit1321] Long M., Cai W. M., Cai J., Zhou B. X., Chai X. Y., Wu Y. H. (2006). J. Phys. Chem. B.

[cit1322] Xie X., Li Y., Liu Z.-Q., Haruta M., Shen W. (2009). Nature.

[cit1323] Arico A. S., Bruce P., Scrosati B., Tarascon J.-M., van Schalkwijk W. (2005). Nat. Mater..

[cit1324] Schäfer H., Kuepper K., Koppe J., Selter P., Steinhart M., Hansen M. R., Daum D. (2018). ACS Catal..

[cit1325] Wohlfahrt-Mehrens M., Heitbaum J. (1987). J. Electroanal. Chem..

[cit1326] Diaz-Morales O., Calle-Vallejo F., de Munck C., Koper M. T.-M. (2013). Chem. Sci..

[cit1327] Schäfer H., Küpper K., Schmidt M., Müller-Buschbaum K., Stangl J., Daum D., Steinhart M., Schulz-Kölbel C., Han W., Wollschläger J., Krupp U., Hou P., Liu X. (2018). Catal. Sci. Technol..

[cit1328] Huck M., Ring L., Küpper K., Klare J., Daum D., Schäfer H. (2020). J. Mater. Chem. A.

[cit1329] Seitz L. C., Dickens C. F., Nishio K., Hikita Y., Montoya J., Doyle A., Kirk C., Vojvodic A., Hwang H. Y., Norskov J. K., Jaramillo T. F. (2016). Science.

[cit1330] Zou X., Zhang Y. (2015). Chem. Soc. Rev..

[cit1331] Kundu S., Bramhaiah K., Bhattacharyya S. (2020). Nanoscale Adv..

[cit1332] Xu Y., Kraft M., Xu R. (2016). Chem. Soc. Rev..

[cit1333] Zhang P., Sun F., Xiang Z., Shen Z., Yun J., Cao D. (2014). Energy Environ. Sci..

[cit1334] Mulyadi A., Zhang Z., Dutzner M., Liu W., Deng Y. (2017). Nano Energy.

[cit1335] Ren X., Li Z., Qiao H., Liang W., Liu H., Zhang F., Qi X., Liu Y., Huang Z., Zhang D., Li J., Zhong J., Zhang H. (2019). ACS Appl. Energy Mater..

[cit1336] Chiang C. K., Fincher C. R., Park Y. W., Heeger A. J., Shirakawa H., Louis E. J., Gau S. C., MacDiarmid A. G. (1977). Phys. Rev. Lett..

[cit1337] Somasundrum M., Bannister J. V. (1993). J. Chem. Soc., Chem. Commun..

[cit1338] Jacobs R. C.-M., Janssen L. J.-J., Barendrecht E. (1985). Electrochim. Acta.

[cit1339] Winther-Jensen B., Fraser K., Ong C., Forsyth M., MacFarlane D. R. (2010). Adv. Mater..

[cit1340] Winther-Jensen B., MacFarlane D. R. (2011). Energy Environ. Sci..

[cit1341] Gong K., Du F., Xia Z., Durstock M., Dai L. (2009). Science.

[cit1342] Liu R., Wu D., Feng X., Müllen K. (2010). Angew. Chem., Int. Ed..

[cit1343] Zheng Y., Jiao Y., Ge L., Jaroniec M., Qiao S. Z. (2013). Angew. Chem., Int. Ed..

[cit1344] Liang J., Jiao Y., Jaroniec M., Qiao S. Z. (2012). Angew. Chem., Int. Ed..

[cit1345] Jiao Y., Zheng Y., Jaroniec M., Qiao S. Z. (2014). J. Am. Chem. Soc..

[cit1346] Zheng Y., Jiao Y., Li L. H., Xing T., Chen Y., Jaroniec M., Qiao S. Z. (2014). ACS Nano.

[cit1347] Chen W.-F., Sasaki K., Ma C., Frenkel A. I., Marinkovic N., Muckerman J. T., Zhu Y., Adzic R. R. (2012). Angew. Chem., Int. Ed..

[cit1348] Chen W.-F., Wang C.-H., Sasaki K., Marinkovic N., Xu W., Muckerman J. T., Zhu Y., Adzic R. R. (2013). Energy Environ. Sci..

[cit1349] Ng J. W.-D., Tang M., Jaramillo T. F. (2014). Energy Environ. Sci..

[cit1350] Mirzakulova E., Khatmullin R., Walpita J., Corrigan T., Vargas-Barbosa N. M., Vyas S., Oottikkal S., Manzer S. F., Hadad C. M., Glusac K. D. (2012). Nat. Chem..

[cit1351] Hand R., Nelson R. F., Obrien C. J., Carpenter A. K. (1972). J. Electrochem. Soc..

[cit1352] Wang X.-D., Xu Y.-F., Rao H.-S., Xu W.-J., Chen H.-Y., Zhang W.-X., Kuang D.-B., Su C.-Y. (2016). Energy Environ. Sci..

[cit1353] Nakayama M., Fujimoto K., Kobayakawa T., Okada T. (2017). Electrochem. Commun..

[cit1354] Liu Q., Asiri A. M., Sun X. P. (2014). Electrochem. Commun..

[cit1355] Wang A.-L., He X.-J., Lu X.-F., Xu H., Tong Y.-X., Li G.-R. (2015). Angew. Chem., Int. Ed..

[cit1356] Cheng N., Liu Q., Tian J., Xue Y., Asiri A. M., Jiang H., He Y., Sun X. (2015). Chem. Commun..

[cit1357] Ali A., Akyüz D., Asghar M. A., Koca A., Keskin B. (2018). Int. J. Hydrogen Energy.

[cit1358] Zhang Y., Fan X., Jian J., Yu D., Zhang Z., Dai L. (2017). Energy Environ. Sci..

[cit1359] Lei C., Zheng Q., Cheng F., Hou Y., Yang B., Li Z., Wen Z., Lei L., Chai G., Feng X. (2020). Adv. Funct. Mater..

[cit1360] Kinoshita K. , Carbon: electrochemical and physicochemical properties, John Wiley & Sons, New York, 1988

[cit1361] Ross P. N., Sattler M. (1988). J. Electrochem. Soc..

[cit1362] Castanheira L., Silva W. O., Lima F. H. B., Crisci A., Dubau L., Maillard F. (2015). ACS Catal..

[cit1363] Ross P. N., Sokol H. (1984). J. Electrochem. Soc..

[cit1364] Staud N., Ross P. N. (1986). J. Electrochem. Soc..

[cit1365] Staud N., Sokol H., Ross P. N. (1989). J. Electrochem. Soc..

[cit1366] Castanheira L., Dubau L., Mermoux M., Berthomé G., Caqué N., Rossinot E., Chatenet M., Maillard F. (2014). ACS Catal..

[cit1367] Lafforgue C., Maillard F., Martin V., Dubau L., Chatenet M. (2019). ACS Catal..

[cit1368] Möller S., Barwe S., Masa J., Wintrich D., Seisel S., Baltruschat H., Schuhmann W. (2020). Angew. Chem., Int. Ed..

[cit1369] Staffell I. , PhD thesis, University of Birmingham, 2010

[cit1370] Paraknowitsch J. P., Thomas A. (2013). Energy Environ. Sci..

[cit1371] Sakaushi K., Antonietti M. (2015). Bull. Chem. Soc. Jpn..

[cit1372] Fellinger T.-P., Hasche F., Strasser P., Antonietti M. (2012). J. Am. Chem. Soc..

[cit1373] Men Y., Siebenburger M., Qiu X., Antonietti M. (2013). J. Mater. Chem. A.

[cit1374] Sakaushi K., Fellinger T.-P., Antonietti M. (2015). ChemSusChem.

[cit1375] Zhao Y., Nakamura R., Kamiya K., Nakanishi S., Hashimoto K. (2013). Nat. Commun..

[cit1376] Zhao Z., Huang X., Xu L., Yan D., Huo J., Wang S. (2016). Chem. Commun..

[cit1377] Balogun M.-S., Qiu W., Yang H., Fan W., Huang Y., Fang P., Li G., Ji H., Tong Y. (2016). Energy Environ. Sci..

[cit1378] Sun T., Wu Q., Jiang Y., Zhang Z., Du L., Yang L., Wang X., Hu Z. (2016). Chem. – Eur. J..

[cit1379] Huang S., Meng Y., Cao Y., He S., Li X., Tong S., Wu M. (2019). Appl. Catal., B.

[cit1380] Wang H.-F., Tang C., Zhang Q. (2018). Catal. Today.

[cit1381] Singh D. K., Jenjeti R. N., Sampath S., Eswaramoorthy M. (2017). J. Mater. Chem. A.

[cit1382] Hu C., Dai L. (2017). Adv. Mater..

[cit1383] Sun T., Wang J., Qiu C., Ling X., Tian B., Chen W., Su C. (2018). Adv. Sci..

[cit1384] Lin Y.-X., Feng W.-J., Zhang J.-J., Xue Z.-H., Zhao T.-J., Su H., Hirano S. I., Li X.-H., Chen J.-S. (2018). Angew. Chem., Int. Ed..

[cit1385] Zhu Y., Zhang T., Lee J. Y. (2018). ChemElectroChem.

[cit1386] Zhao X., Su H., Cheng W., Zhang H., Che W., Tang F., Liu Q. (2019). ACS Appl. Mater. Interfaces.

[cit1387] Zhao M., Li T., Jia L., Li H., Yuan W., Li C. M. (2019). ChemSusChem.

[cit1388] Mondal S., Mohanty B., Nurhuda M., Dalapati S., Jana R., Addicoat M., Datta A., Jena B. K., Bhaumik A. (2020). ACS Catal..

[cit1389] Zhang J., Zhao Z., Xia Z., Dai L. (2015). Nat. Nanotechnol..

[cit1390] Li M., Zhang L., Xu Q., Niu J., Xia Z. (2014). J. Catal..

[cit1391] Zhang L., Xia Z. (2011). J. Phys. Chem. C.

[cit1392] Zhang J., Qu L., Shi G., Liu J., Chen J., Dai L. (2016). Angew. Chem., Int. Ed..

[cit1393] Zhang Q., Luo F., Ling Y., Guo L., Qu K., Hu H., Yang Z., Cai W., Cheng H. (2018). ChemCatChem.

[cit1394] Yue X., Huang S., Cai J., Jin Y., Shen P. K. (2017). J. Mater. Chem. A.

[cit1395] Zhang Z., Yi Z., Wang J., Tian X., Xu P., Shi G., Wang S. (2017). J. Mater. Chem. A.

[cit1396] Lei Y., Wang L., Zhai S., Wang Y., Karahan H. E., Xhen X., Zhou Z., Wang C., Sui X., Chen Y. (2018). Mater. Chem. Front..

[cit1397] Rajagopal V., Kathiresan M., Manivel P., Suryanarayanan V., Velayutham D., Ho K.-C. (2020). J. Taiwan Inst. Chem. Eng..

[cit1398] Zhao M., Zhang J., Xiao H., Hu T., Jia J., Wu H. (2019). Chem. Commun..

[cit1399] Lai J., Li S., Wu F., Saqib M., Luque R., Xu G. (2016). Energy Environ. Sci..

[cit1400] Gusmao R., Sofer Z., Bousa D., Pumera M. (2017). Angew. Chem., Int. Ed..

[cit1401] Li F., Xue M., Li J., Ma X., Chen L., Zhang X., MacFarlane D. R., Zhang J. (2017). Angew. Chem., Int. Ed..

[cit1402] Gao Y., Tian W., Huo C., Zhang K., Guo S., Zhang S., Song X., Jiang L., Huo K., Zeng H. (2019). J. Mater. Chem. A.

[cit1403] Guo S., Hu X., Zhou W., Liu X., Gao Y., Zhang S., Zhang K., Zhu Z., Zeng H. (2018). J. Phys. Chem. C.

[cit1404] Martínez-Periñán E., Down M. P., Gibaja C., Lorenzo E., Zamora F., Banks C. E. (2018). Adv. Energy Mater..

[cit1405] Gao R., Dai Q., Du F., Yan D., Dai L. (2019). J. Am. Chem. Soc..

[cit1406] Zhang J., Dai L. (2016). Angew. Chem., Int. Ed..

[cit1407] Goettmann F., Fischer A., Antonietti M., Thomas A. (2006). Chem. Commun..

[cit1408] Lyth S. M., Nabae Y., Morya S., Kuroki S., Kakimoto M., Ozaki J., Miyata S. (2009). J. Phys. Chem. C.

[cit1409] Tian J., Liu Q., Asiri A. M., Alamry K. A., Sun X. (2014). ChemSusChem.

[cit1410] Ma T., Ran J., Dai S., Jaroniec M., Qiao S. (2014). Angew. Chem., Int. Ed..

[cit1411] Peng Z., Yang S., Jia D., Da P., He P., Al-Enizi A. M., Ding G., Xie X., Zheng G. (2016). J. Mater. Chem. A.

[cit1412] Wahab M. A., Joseph J., Atanda L., Sultana U. K., Beltramini J. N., Ostrikov K., Will G., O’Mullane A. P., Abdala A. (2020). ACS Appl. Energy Mater..

[cit1413] Song L., Liu Z., Reddy A. L.-M., Narayanan N. T., Taha Tijerina J., Peng J., Gao G. H., Lou J., Vajtai R., Ajayan P. M. (2012). Adv. Mater..

[cit1414] Wang X. W., Sun G. Z., Routh P., Kim D. H., Huang W., Chen P. (2014). Chem. Soc. Rev..

[cit1415] Lee C. H., Jun B., Lee S. U. (2018). ACS Sustainable Chem. Eng..

[cit1416] Kötz R., Stucki S. (1986). Electrochim. Acta.

[cit1417] Honing J. M. , in Electrodes of metallic conductive oxides, ed. S. Trasatti, Elsevier Scientific, Amserdam, 1980

[cit1418] Adams R., Shriner L. L. (1923). J. Am. Chem. Soc..

[cit1419] Puthiyapura V. K., Pasupathi S., Su H., Liu X., Pollet B., Scott K. (2014). Int. J. Hydrogen Energy.

[cit1420] Mamlouk M., Pasupathi S., Pollet B. G., Scott K. (2014). J. Power Sources.

[cit1421] Liua Y., Wanga C., Leia Y., Liub F., Tian B., Wang J. (2018). Int. J. Hydrogen Energy.

[cit1422] Mitchel D., Rand D. A.-J., Woods R. (1978). J. Electroanal. Chem..

[cit1423] Nguyen T. D., Scherer G. G., Xu Z. J. (2016). Electrocatalysis.

[cit1424] Sharma S., Pollet B. G. (2012). J. Power Sources.

[cit1425] Nong H. N., Oh H. S., Reier T., Willinger E., Willinger M. G., Petkov V., Teschner D. (2015). Angew. Chem., Int. Ed..

[cit1426] Ozouf G., Beauger C. (2016). J. Mater. Sci..

[cit1427] Puthiyapura V. K., Pasupathi S., Basu S., Wu X., Su H., Varagunapandiyan N., Pollet B., Scott K. (2013). Int. J. Hydrogen Energy.

[cit1428] Rajan Z. S.-H. S., Binninger T., Kooyman P. J., Susac D., Mohamed R. (2020). Catal. Sci. Technol..

[cit1429] Nong H. N., Gan L., Willinger E., Teschner D., Strasser P. (2014). Chem. Sci..

[cit1430] Millet P., Durand R., Pinéri M. (1989). J. Appl. Electrochem..

[cit1431] Rozain C., Millet P. (2014). Electrochim. Acta.

[cit1432] Pollet B. G. (2019). Catalysts.

[cit1433] Bett J. A., Kinoshita K., Stoneheart P. (1974). J. Catal..

[cit1434] Grolleau C., Coutanceau C., Pierre F., Léger J. M. (2008). Electrochim. Acta.

[cit1435] Lebègue E., Baranton S., Coutanceau C. (2011). J. Power Sources.

[cit1436] Solla-Gullón J., Montiel V., Aldaz A., Clavilier J. (2000). J. Electroanal. Chem..

[cit1437] Adora S., Soldo-Olivier Y., Faure R., Durand R., Dartyge E., Baudelet F. (2001). J. Phys. Chem. B.

[cit1438] Wang J., Yin G., Shao Y., Wang Z., Gao Y. (2008). J. Phys. Chem. C.

[cit1439] Luo W., Gan J., Huang Z., Chen W., Qian G., Zhou X., Duan X. (2019). Front. Mater..

[cit1440] del Carmen Gimenez-Lopez M., Kurtoglu A., Walsh D. A., Khlobystov A. N. (2016). Adv. Mater..

[cit1441] Hansen H. E., Seland F., Sunde S., Burheim O. S., Pollet B. G. (2021). Mater. Adv..

[cit1442] Karousos D. S., Desdenakis K. I., Sakkas P. M., Sourkouni G., Pollet B. G., Argirusis C. (2017). Ultrason. Sonochem..

[cit1443] Pantani O., Naskar S., Guillot R., Millet P., Anxolabéhère E., Aukauloo A. (2008). Angew. Chem..

[cit1444] Varzatsky O. A., Chornenka N. V., Belov A. S., Grigoriev S. A., Pushkarev A. S., Millet P., Kalinichenko V. N., Voloshin Y. Z., Belaya I. G., Bugaenko M. G., Dedov A. G. (2018). Electrochim. Acta.

[cit1445] Millet P. (2016). ECS Trans..

[cit1446] Pollet B. G., Kocha S. S. (2021). Johnson Matthey Technol. Rev..

[cit1447] Al Cheikh J., Villagra A., Ranjbari A., Pradon A., Antuch M., Dragoe D., Millet P., Assaud L. (2019). Appl. Catal., B.

[cit1448] Al Cheikh J., Zakari R., Bhosale A., Villagra A., Leclerc N., Floquet S., Ghosh P. C., Ranjbari A., Cadot E., Millet P., Assaud L. (2020). Mater. Adv..

[cit1449] Yue D., Zhang T., Kan M., Qian X., Zhao Y. (2016). Appl. Catal., B.

[cit1450] Takenaka H. and Torikai E., Production of Ion-Exchange Membrane-Catalytic Electrode Bonded Material for Electrolytic Cells, Jap. Pat., 55-38934, 1980

[cit1451] Nagel H. and Stucki S., Method for electrolytic deposition of metals, US patent no 4326930, 1982

[cit1452] Kocha S. S. , Principle of MEA preparation, in Handbook of Fuel Cells, ed. W. Vielstich, A. Lamm and H. A. Gasteiger, Wiley, Chichester, 2003, vol. 3, pp. 538–565

[cit1453] Fedotov A. A., Grigoriev S. A., Lyutikova E. K., Millet P., Fateev V. N. (2013). Int. J. Hydrogen Energy.

[cit1454] Moureaux F., Stevens P., Toussaint G., Chatenet M. (2019). Appl. Catal., B.

[cit1455] Sivanantham A., Ganesan P., Shanmugam S. (2016). Adv. Funct. Mater..

[cit1456] Chen J., Li H., Yu Z., Liu C., Yuan Z. (2020). Adv. Energy Mater.

[cit1457] Liu X., Guo Y., Wang P., Wu Q., Zhang Q., Rozhkova E. A., Wang Z., Liu Y., Zheng Z., Dai Y., Huang B. (2020). ACS Appl. Energy Mater..

[cit1458] Liu X., Zhang L., Li L., Ye X., Chen H., Wie Z. (2020). Inorg. Chem..

[cit1459] Barber J. (2009). Chem. Soc. Rev..

[cit1460] McEvoy J. P., Brudvig G. W. (2006). Chem. Rev..

[cit1461] Siegbahn P. E.-M. (2009). J. Am. Chem. Soc..

[cit1462] Dismukes G. C. (2001). Science.

[cit1463] Loll B., Kern J., Saenger W., Zouni A., Biesiadka J. (2005). Nature.

[cit1464] Murray J., Barber J. (2007). J. Struct. Biol..

[cit1465] Ferreira K. N., Iverson T. M., Maghlaoui K., Barber J., Iwata S. (2004). Science.

[cit1466] van Leeuwen P. W.-N. M. , Homogenous Catalysis, Kluver Acedemic Publishers, 2004

[cit1467] Hisatomi T., Kubota J., Domen K. (2014). Chem. Soc. Rev..

[cit1468] Yagi M., Kaneko M. (2001). Chem. Rev..

[cit1469] Osterloh F. E. (2013). Chem. Soc. Rev..

[cit1470] Ran J. R., Zhang J., Yu J. G., Jaroniec M., Qiao S. Z. (2014). Chem. Soc. Rev..

[cit1471] Takanabe K. (2017). ACS Catal..

[cit1472] Folkman S. J., Soriano-Lopez J., Ramón Galán-Mascarós J., Finke R. G. (2018). J. Am. Chem. Soc..

[cit1473] Sconyers D. J., Blakemore J. D. (2017). Chem. Commun..

[cit1474] Garrido-Barros P., Gimbert-Surinach C., Matheu R., Sala X., LIobet A. (2017). Chem. Soc. Rev..

[cit1475] Helm M. L., Stewart M. P., Bullock R. M., DuBois M. R., DuBois D. L. (2011). Science.

[cit1476] Gersten S. W., Samuels G. J., Meyer T. J. (1982). J. Am. Chem. Soc..

[cit1477] Gilbert J. A., Eggleston D. S., Murphy W. R., Geselowitz G. A., Gersten S. W., Hodgson D. J., Meyer T. J. (1985). J. Am. Chem. Soc..

[cit1478] In CRC Handbook of Chemistry and Physics, ed. D. R. Lide, CRC Publishing, Boca Raton, FL, 86th edn, 2005, p. 8

[cit1479] Concepcion J. J., Jurss J. W., Brennaman M. K., Hoertz P. G., Patrocinio A. O.-T., Iha N. Y.-M., Templeton J. L., Meyer T. J. (2009). Acc. Chem. Res..

[cit1480] Hurst J. K., Zhou J., Lei Y. (1992). Inorg. Chem..

[cit1481] Chronister C. W., Binstead R. A., Ni J., Meyer T. J. (1997). Inorg. Chem..

[cit1482] Yamada H., Hurst J. K. (2000). J. Am. Chem. Soc..

[cit1483] Cape J. L., Hurst J. K. (2008). J. Am. Chem. Soc..

[cit1484] Liu F., Concepcion J. J., Jurss J. W., Cardolaccia T., Templeton J. L., Meyer T. J. (2008). Inorg. Chem..

[cit1485] Hurst J. K., Roemeling M. D., Lymar S. V. (2015). J. Phys. Chem. B.

[cit1486] Volpe A., Tubaro C., Natali M., Sartorel A., Brundvig G. W., Bonchio M. (2019). Inorg. Chem..

[cit1487] Concepcion J. J., Jurss J. W., Templeton J. L., Meyer T. J. (2008). J. Am. Chem. Soc..

[cit1488] Concepcion J. J., Tsai M.-K., Muckerman J. T., Meyer T. J. (2010). J. Am. Chem. Soc..

[cit1489] Parent A. R., Crabtree R. H., Brudvig G. W. (2013). Chem. Soc. Rev..

[cit1490] Tseng H.-W., Zong R., Muckerman J. T., Thummel R. (2008). Inorg. Chem..

[cit1491] Masaoka S., Sakai K. (2009). Chem. Lett..

[cit1492] Dovletoglou A., Adeyemi S. A., Meyer T. J. (1996). Inorg. Chem..

[cit1493] Masllorens E., Rodríguez M., Romero I., Roglans A., Parella T., Benet-Buchholz J., Poyatos M., Llobet A. (2006). J. Am. Chem. Soc..

[cit1494] Conception J. J., Jurss J. W., Templeton J. L., Meyer T. J. (2008). Proc. Natl. Acad. Sci. U. S. A..

[cit1495] Conception J. J., Jurss J. W., Hoertz P. G., Meyer T. J. (2009). Angew. Chem., Int. Ed..

[cit1496] Tsubonouchi Y., Eo T., Honta J., Sato T., Mohamed E. A., Zahran Z. N., Saito K., Yui T., Yagi M. (2020). J. Photochem. Photobiol., A.

[cit1497] Matias T. A., Parassulo A. L.-A., Benavides P. S., Guimaraes R. R., Dourado A. H.-B., Nakamura M., Cordoba de Torres S. I., Bertotti M., Araki K. (2018). Electrochim. Acta.

[cit1498] Ashford D. L., Sherman B. D., Binstead R. A., Templeton J. L., Meyer T. J. (2015). Angew. Chem., Int. Ed..

[cit1499] Concepcion J. J., Binstead R. A., Alibabaei L., Meyer T. J. (2013). Inorg. Chem..

[cit1500] Matheu R., Ertem M. Z., Benet-Buchholz J., Coronado E., Batista V. S., Sala X., Llobet A. (2015). J. Am. Chem. Soc..

[cit1501] Huynh M., Bediako D. K., Nocera D. G. (2014). J. Am. Chem. Soc..

[cit1502] Schäfer H., Küpper K., Müller-Buschbaum K., Daum D., Steinhart M., Wollschläger J., Krupp U., Schmidt M., Han W., Stangl J. (2017). Nanoscale.

[cit1503] Patel P. P., Datta M. K., Velikokhatnyi O. I., Kuruba R., Damodaran K., Jampani P., Gattu B., Shanti P. M., Damle S. S., Kumta P. N. (2016). Sci. Rep..

[cit1504] Norris M. R., Concepcion J. J., Fang Z., Templeton J. L., Meyer T. J. (2013). Angew. Chem., Int. Ed..

[cit1505] Yoshida M., Kondo M., Torii S., Sakai K., Masaoka S. (2015). Angew. Chem., Int. Ed..

[cit1506] Matias T. A., Kepper A., Heering Bartoloni F. (2020). Dalton Trans..

[cit1507] Das B., Ezzedinloo L., Bhadbhade M., Bucknallband M. P., Colbran S. B. (2017). Chem. Commun..

[cit1508] Lomoth R., Huang P., Zheng J., Sun L., Hammarström L., Akermark B., Styring S. (2002). Eur. J. Inorg. Chem..

[cit1509] Matheu R., Ghaderian A., Francàs L., Chernev P., Ertem M. Z., Benet-Buchholz J., Batista V. S., Haumann M., Gimbert-Suriñach C., Sala X., Llobet A. (2018). Chem. – Eur. J..

[cit1510] Xu Y., Akermark T., Gyollai V., Zou D., Eriksson L., Duan L., Zhang R., Akermark B., Sun L. (2009). Inorg. Chem..

[cit1511] Yang J., Wang L., Zhang S., Zou H., Chen H., Ahlquist M. S.-G., Duan L., Sun L. (2021). Nat Commun.

[cit1512] Duan L., Fischer A., Xu Y., Sun L. (2009). J. Am. Chem. Soc..

[cit1513] Watabe S., Tanahashi Y., Hirahara M., Yamazaki H., Takahashi K., Mohamed E. A., Tsubonouchi Y., Zahran Z. N., Saito K., Yui T., Yagi M. (2019). Inorg. Chem..

[cit1514] Dhiman R., Nagaraja C. M. (2018). Eur. J. Inorg. Chem..

[cit1515] Govindarajan N., Tiwari A., Ensing B., Jan Meijer E. (2018). Inorg. Chem..

[cit1516] Vereshchuk N., Matheu R., Benet-Buchholz J., Pipelier M., Lebreton J., Dubreuil D., Tessier A., Gimbert-Suriñach C., Ertem M. Z., Llobet A. (2020). J. Am. Chem. Soc..

[cit1517] Matheu R., Ertem M. Z., Gimbert-Surinach C., Sala X., LIobet A. (2019). Chem. Rev..

[cit1518] Hoque M. A., Gil-Sepulcre M., de Aguirre A., Elemans J. A.-A. W., Moonshiram D., Matheu R., Shi Y., Benet-Buchholz J., Sala X., Malfois M. (2020). et al.. Nat. Chem..

[cit1519] Zhao Y., Chen S., Sun B., Su D., Huang X., Liu H., Yan Y., Sun K., Wang G. (2014). Sci. Rep..

[cit1520] Fuehwald H. M., Moghaddam R. B., Zenkina O. V., Bradley Easton E. (2019). Catal. Sci. Technol..

[cit1521] Lu X. Y., Zhao C. (2013). J. Mater. Chem. A.

[cit1522] Wu J., Xue Y., Yan X., Yan W., Cheng Q., Xie Y. (2012). Nano. Res..

[cit1523] Peng M., Shi D., Sun Y., Cheng J., Zhao B., Xie Y., Zhang J., Guo W., Jia Z., Liang Z., Jiang L. (2020). Adv. Mater..

[cit1524] Zhang M.-T., Chen Z., Kang P., Meyer T. J. (2013). J. Am. Chem. Soc..

[cit1525] Ford P. C. (1970). Coord. Chem. Rev..

[cit1526] Lim H., Barclay D. J., Anson F. C. (1972). Inorg. Chem..

[cit1527] Ando I., Katae H., Okamura M. (2014). Inorganica Chimica Acta.

[cit1528] Metzker G., de Aguiar I., Martins S. C., Schultz M. S., Vasconcellos L. C. G., Franco D. W. (2014). Inorganica Chimica Acta.

[cit1529] Kaneko M., Ramaraj R., Kira A. (1988). Bull. Chem. Soc. Jpn..

[cit1530] Kinoshita K., Yagi M., Kaneko M. (1999). J. Mol Catal. A: Chem..

[cit1531] Yagi M., Yamaguchi T., Kaneko M. (1999). J. Mol. Catal. A: Chem..

[cit1532] Yagi M., Kinoshita K., Kaneko M. (1996). J. Phys. Chem..

[cit1533] Kinoshita K., Yagi M., Kaneko M. (1998). Macromolecules.

[cit1534] Yagi M., Kinoshita K., Kaneko M. (1997). J. Phys. Chem. B.

[cit1535] Ogino O., Nagoshi K., Yagi M., Kaneko M. (1996). J. Chem. Soc., Faraday Trans..

[cit1536] Nagoshi K., Yagi M., Kaneko M. (2000). Bull. Chem. Soc. Jpn..

[cit1537] Howells A. R., Sankarraj A., Shannon C. (2004). J. Am. Chem. Soc..

[cit1538] Sartorel A., Miro P., Salvaodori E., Romain S., Carraro M., Scorrano G., Di Valentin M., Llobet A., Bo C., Bonchio M. (2009). J. Am. Chem. Soc..

[cit1539] Geletii Y. V., Botar B., Kögerler P., Hillesheim D. A., Musaev D. G., Hill C. L. (2008). Angew. Chem., Int. Ed..

[cit1540] Geletii Y. V., Besson C., Hou Y., Yin Q., Musaev D. G., Quinonero D., Cao R., Hardcastle K. I., Proust A., Kögerler P., Hill C. L. (2009). J. Am. Chem. Soc..

[cit1541] Dzhabieva Z. M., Avdienko O. P., Antonova Y. I., Yu. Tkachenko V., Dzhabiev T. S. (2015). Rus. J. Phys. Chem. A.

[cit1542] Dzhabievaa Z. M., Shilov G. V., Yu. Tkachenko V., Avdeeva L. V., Dzhabiev T. S. (2016). Russ. J. Inorg. Chem..

[cit1543] Tkachenko Y. Y., Dzhabieva Z. M., Tkachenko L. I., Avdeeva L. V., Dzhabiev T. S. (2020). Russ. J. Electrochem..

[cit1544] Pipes D. W., Meyer T. J. (1986). Inorg. Chem..

[cit1545] Meyer T. J., Huynh M. V.-H. (2003). Inorg. Chem..

[cit1546] McDaniel N. D., Coughhlin F. J., Tinker L. L., Bernhard S. (2008). J. Am. Chem. Soc..

[cit1547] Hull J. F., Balcells D., Blakemore J. D., Incarvito C. D., Eisenstein O., Brudvig G. W., Crabtree R. H. (2009). J. Am. Chem. Soc..

[cit1548] Mazloomi Z., Margalef J., Gil-Sepulcre M., Romero N., Albrecht M., Llobet A., Sala X., Pamies O., Dieguez M. (2020). Inorg. Chem..

[cit1549] van Dijk B., Menendez Rodriguez G., Wu L., Hofmann J. P., Macchioni A., Hetterscheid D. G.-H. (2020). ACS Catal..

[cit1550] Volpe A., Sartorel A., Graiff C., Bonchio M., Biffis A., Baron M., Tubaro C. (2020). J. Organomet. Chem..

[cit1551] Sánchez-Page B., Pérez-Mas A. M., González-Ingelmo M., González L., González Z., Victoria Jiménez M., Pérez-Torrente J. J., Blasco J., Subías G., Álvarez P., Granda M., Menéndez R. (2020). J. Organomet. Chem..

[cit1552] Nieto J., Victoria Jimenez M., Alvarez P., Perez-Mas A. M., Gonzalez Z., Pereira R., Sanchez-Page B., Perez-Torrente J. J., Blasco J., Menendez R. (2019). ACS Appl. Energy Mater..

[cit1553] Singh A., Spiccia L. (2013). Coord. Chem. Rev..

[cit1554] Limburg J., Vrettos J. S., Chen H., de Paula J. C., Crabtree R. H., Brudvig G. W. (2001). J. Am. Chem. Soc..

[cit1555] Limburg J., Brudvig G. W., Crabtree R. H. (1997). J. Am. Chem. Soc..

[cit1556] Karlsson E. A., Lee B.-L., Åkermark T., Johnston E. V., Kärkäs M. D., Sun J., Hansson Ö., Bäckvall J.-E., Åkermark B. (2011). Angew. Chem., Int. Ed..

[cit1557] Ruettinger W., Yagi M., Wolf K., Bernasek S., Dismukes G. C. (2000). J. Am. Chem. Soc..

[cit1558] Brimblecombe R., Swiegers G. F., Dismukes G. C., Spiccia L. (2008). Angew. Chem., Int. Ed..

[cit1559] Dunand-Sauthier M. N.-C., Deronzier A., Piron A., Pradon X., Menage S. (1998). J. Am. Chem. Soc..

[cit1560] Gorun S. M., Stibrany R. T., Lillo A. (1998). Inorg. Chem..

[cit1561] Codolà Z., Garcia-Bosch I., Acuña-Parés F., Prat I., Luis J. M., Costas M., Lloret-Fillol J. (2013). Chem. – Eur. J..

[cit1562] Das B., Orthaber A., Ott S., Thappe A. (2016). ChemSusChem.

[cit1563] Kottrup K. G., Hetterscheid D. G.-H. (2016). Chem. Commun..

[cit1564] Wang Z.-Q., Wang Z.-C., Zhan S., Ye J.-S. (2014). Appl. Catal., A.

[cit1565] Wickramasinghe L. D., Zhou R., Zong R., Vo P., Gagnon K. J., Thummel R. P. (2015). J. Am. Chem. Soc..

[cit1566] Liu T., Zhang B., Sun L. (2019). Chem. – Asian J..

[cit1567] Hong D., Mandal S., Yamada Y., Lee Y.-M., Nam W., Llobet A., Fukuzumi S. (2013). Inorg. Chem..

[cit1568] Fillol J. L., Codola Z., Garcia-Bosch I., Gomez L., Pla J. J., Costas M. (2011). Nat. Chem..

[cit1569] Zheng M., Ding Y., Cao X., Tian T., Lin J. (2018). Appl. Catal., B.

[cit1570] Zhang B., Li F., Yu F., Cui H., Zhou X., Li H., Wang Y., Sun L. (2014). Chem. – Asian J..

[cit1571] Chen G., Chen L., Ng S.-M., Man W.-L., Lau T.-C. (2013). Angew. Chem., Int. Ed..

[cit1572] Najafpour M. M., Safdari R., Ebrahimi F., Rafighi P., Bagheri R. (2016). Dalton Trans..

[cit1573] Ellis W. C., McDaniel N. D., Bernhard S., Collins T. J. (2010). J. Am. Chem. Soc..

[cit1574] Okamura M., Kondo M., Kuga R., Kurashige Y., Yanai T., Hayami S., Praneeth V. K.-K., Yoshida M., Yoneda K., Kawata S., Masaoka S. (2016). Nature.

[cit1575] Praneeth V. K.-K., Kondo M., Okamura M., Akai T., Izu H., Masaoka S. (2019). Chem. Sci..

[cit1576] Karim S., Chakraborty A., Samanta D., Zangrando E., Ghosh T., Das D. (2020). Catal. Sci. Technol..

[cit1577] Sinha W., Mahammed A., Fridman N., Gross Z. (2020). ACS Catal..

[cit1578] Shafirovich V. Y., Khannanov N. K., Strelets V. V. (1980). Nouv. J. Chim..

[cit1579] Elizarova G. L., Matvienko L. G., Lozhkina N. V., Parmon V. N., Zamaraev K. I. (1981). React. Kinet. Catal. Lett..

[cit1580] Brunschwig B. S., Chou M. H., Creutz C., Ghosh P., Sutin N. (1983). J. Am. Chem. Soc..

[cit1581] Gerasimov O. V., Elizarova G. L., Parmon V. N. (1992). J. Photochem. Photobiol., B.

[cit1582] Elizarova G. L., Zhidomirov G. M., Parmon V. N. (2000). Catal. Today.

[cit1583] Yin Q., Miles Tan J., Besson C., Geletii Y. V., Musaev D. G., Kuznetsov A. E., Luo Z., Hardcastle K. I., Hill C. L. (2010). Science.

[cit1584] Wasylenko D. J., Ganesamoorthy C., Koivisto B. D., Henderson M. A., Berlinguette C. P. (2010). Inorg. Chem..

[cit1585] Wasylenko D. J., Ganesamoorthy C., Borau-Garcia J., Berlinguette C. P. (2011). Chem. Commun..

[cit1586] Dogutan D. K., McGuire R., Nocera D. G. (2011). J. Am. Chem. Soc..

[cit1587] Lei H., Han A., Li F., Zhang M., Han Y., Du P., Lai W., Cao R. (2014). Phys. Chem. Chem. Phys..

[cit1588] Xu L., Lei H., Zhang Z., Yao Z., Li J., Yu Z., Cao R. (2017). Phys. Chem. Chem. Phys..

[cit1589] Barraza Alvarez I., Wu Y., Sanchez J., Ge Y., Ramos-Garcés M. V., Chu T., Jaramillo T. F., Colón J. L., Villagrán D. (2021). Sustainable Energy Fuels.

[cit1590] Mondal B., Chattopadhyay S., Dey S., Mahammed A., Mittra K., Rana A., Gross Z., Dey A. (2020). J. Am. Chem. Soc..

[cit1591] Neuman N. I., Albold U., Ferretti E., Chandra S., Steinhauer S., Rößner P., Meyer F., Doctorovich F., Vaillard S. E., Sarkar B. (2020). Inorg. Chem..

[cit1592] Lv J., Guan X., Yu M., Li X., Yu Y., Chen D. (2020). Phys. Chem. Chem. Phys..

[cit1593] Biswas S., Bose S., Debgupta J., Das P., Biswas A. N. (2020). Dalton Trans..

[cit1594] Dey A., Kumar V., Pal S., Guha A., Bawari S., Narayanan T. N., Chandrasekhar V. (2020). Dalton Trans..

[cit1595] Su P., Ma S., Huang W., Boyjoo Y., Bai S., Liu J. (2019). J. Mater. Chem. A.

[cit1596] Haider A., Bassil B. S., Soriano-Lopez J., Qasim H. M., Saenz de Pipaon C., Ibrahim M., Dutta D., Koo Y.-S., Carbo J. J., Poblet J. M., Ramon Galan-Mascaros J., Kortz U. (2019). Inorg. Chem..

[cit1597] Pires B. M., dos Santos P. L., Katic V., Strohauer S., Landers R., Formiga A. L.-B., Bonacin J. A. (2019). Dalton Trans..

[cit1598] Lin J., Meng X., Zheng M., Ma B., Ding Y. (2019). Appl. Catal., B.

[cit1599] Luo Z., Zhou M., Wang X. (2018). Appl. Catal., B.

[cit1600] Car P.-E., Guttentag M., Baldridge K. K., Alberto R., Patzke G. R. (2012). Green Chem..

[cit1601] Zhang M., Zhang M.-T., Hou C., Ke Z.-F., Lu T.-B. (2014). Angew. Chem., Int. Ed..

[cit1602] Luo G.-Y., Huang H.-H., Wang J.-W., Lu T.-B. (2016). ChemSusChem.

[cit1603] Wang J.-W., Zhang X.-Q., Huang H.-H., Lu T.-B. (2016). ChemCatChem.

[cit1604] Wang J.-W., Hou C., Huang H.-H., Liu W.-J., Ke Z.-F., Lu T.-B. (2017). Catal. Sci. Technol..

[cit1605] Han Y., Wu Y., Lai W., Cao R. (2015). Inorg. Chem..

[cit1606] Wang L., Duan L., Ambre R. B., Daniel Q., Chen H., Sun J., Das B., Thapper A., Uhlig J., Diner P., Sun L. (2016). J. Catal..

[cit1607] Lin J., Kang P., Liang X., Ma B., Ding Y. (2017). Electrochim. Acta.

[cit1608] Wang D., Bruner C. O. (2017). Inorg. Chem..

[cit1609] Shen J., Wang M., He T., Jiang J., Hu M. (2018). Chem. Commun..

[cit1610] Zhang L.-H., Yu F., Shi Y., Li F., Li H. (2019). Chem. Commun..

[cit1611] Azadi G., Zand Z., Mousazade Y., Bagheri R., Cui J., Song Z., Bikas R., Wozniak K., Allakhverdiev S. I., Najafpour M. M. (2019). Int. J. Hydrogen Energy.

[cit1612] Garrido-Barros P., Grau S., Drouet S., Benet-Buchholz J., Gimbert-Surinach C., Llobet A. (2019). ACS Catal..

[cit1613] Hessels J., Masferrer-Rius E., Yu F., Detz R. J., Klein Gebbink R. J.-M., Reek J. N.-H. (2020). ChemSusChem.

[cit1614] Yoshida M., Onishi S., Mitsutomi Y., Yamamoto F., Nagasaka M., Yuzawa H., Kosugi N., Kondoh H. (2017). J. Phys. Chem. C.

[cit1615] Singh C., Mukhopadhyay S., Das S. K. (2018). Inorg. Chem..

[cit1616] Aligholivand M., Shaghaghi Z., Bikas R., Kozakiewicz A. (2019). RSC Adv..

[cit1617] Shahadat H. M., Younus H. A., Ahmad N., Abdur Rahaman M., Khattak Z. A.-K., Zhuiykov S., Verpoort F. (2019). Catal. Sci. Technol..

[cit1618] Li Q.-J., Ren Y.-J., Xie Q., Wu M., Feng H.-X., Zheng L.-M., Zhang H.-X., Long J.-Q., Wang T.-S. (2020). Appl. Organomet. Chem..

[cit1619] Kalantarifard S., Allakhverdiev S. I., Najafpour M. M. (2020). Int. J. Hydrogen Energy.

[cit1620] Barnett S. M., Goldberg K. I., Mayer J. M. (2012). Nat. Chem..

[cit1621] Garribba E., Micera G., Sanna D., Strinna-Erre L. (2000). Inorg. Chim. Acta..

[cit1622] Fabian I. (1989). Inorg. Chem..

[cit1623] Chen Z., Meyer T. J. (2013). Angew. Chem., Int. Ed..

[cit1624] Su X. J., Zheng C., Hu Q. Q., Du H. Y., Liao R. Z., Zhang M. T. (2018). Dalton Trans..

[cit1625] Coggins M. K., Zhang M. T., Chen Z., Song N., Meyer T. J. (2014). Angew. Chem., Int. Ed..

[cit1626] Su X. J., Gao M., Jiao L., Liao R. Z., Siegbahn P. E., Cheng J. P., Zhang M. T. (2015). Angew. Chem., Int. Ed..

[cit1627] Chen F., Wang N., Lei H., Guo D., Liu H., Zhang Z., Zhang W., Lai W., Cao R. (2017). Inorg. Chem..

[cit1628] Liu Y., Han Y., Zhang Z., Zhang W., Lai W., Wang Y., Cao R. (2019). Chem. Sci..

[cit1629] Shaghaghi Z., Sallakh Kouhsangini P., Mohammad-Rezaei R. (2021). Appl. Organomet. Chem..

[cit1630] Kölle U. (1992). New. J. Chem..

[cit1631] Artero V., Fontecave M. (2013). Chem. Soc. Rev..

[cit1632] Artero V., Fontecave M. (2005). Coord. Chem. Rev..

[cit1633] Du P., Eisenberg R. (2012). Energy Environ. Sci..

[cit1634] Losse S., Voss J. G., Rau S. (2010). Coord. Chem. Rev..

[cit1635] Thoi V. S., Sun Y., Long J. R., Chang C. J. (2013). Chem. Soc. Rev..

[cit1636] Rakowski Dubois M., Dubois D. L. (2009). Acc. Chem. Res..

[cit1637] Drosou M., Kamatsos F., Mitsopoulou C. A. (2020). Inorg. Chem. Front..

[cit1638] Chao T. H., Espenson J. H. (1978). J. Am. Chem. Soc..

[cit1639] Dempsey J. L., Winkler J. R., Gray H. B. (2010). J. Am. Chem. Soc..

[cit1640] Schrauzer G. N., Holland R. J. (1971). J. Am. Chem. Soc..

[cit1641] Solis B. H., Hammes-Schiffer S. (2011). J. Am. Chem. Soc..

[cit1642] Solis B. H., Hammes-Schiffer S. (2011). Inorg. Chem..

[cit1643] Muckerman J. T., Fujita E. (2011). Chem. Commun..

[cit1644] Solis B. H., Yu Y., Hammes-Schiffer S. (2013). Inorg. Chem..

[cit1645] Frey M. (2002). ChemBioChem.

[cit1646] Fontecilla-Camps J. C., Volbeda A., Cavazza C., Nicolet Y. (2007). Chem. Rev..

[cit1647] Rajakumar M., Manickam M., Gandhi N. N., Muthukumar K. (2020). Int. J. Hydrogen.

[cit1648] Abdullahi I. M., Masud J., Ioannou P.-C., Ferentinos E., Kyritsis P., Nath M. (2021). Molecules.

[cit1649] Wang Z.-Q., Tang L.-Z., Zhang Y.-X., Zhan S.-Z., Ye J.-S. (2015). J. Power Sources.

[cit1650] Volbeda A., Charon M. H., Piras C., Hatchikian E. C., Frey M., Fontecilla-Camps J. C. (1995). Nature.

[cit1651] Higuchi Y., Yagi T., Yasuoka N. (1997). Structure.

[cit1652] Higuchi Y., Ogata H., Miki K., Yasuoka N., Yagi T. (1999). Structure.

[cit1653] Gloaguen F. D.-R., Lawrence J. D., Rauchfuss T. B. (2001). J. Am. Chem. Soc..

[cit1654] Darensbourg M. Y., Lyon E. J., Zhao X., Georgakaki I. P. (2003). Proc. Natl. Acad. Sci. U. S. A..

[cit1655] Sun L., Åkermark B., Ott S. (2005). Coord. Chem. Rev..

[cit1656] Tard C., Liu X., Ibrahim S. K., Bruschi M., Gioia L. D., Davies S. C., Yang X., Wang L.-S., Sawers G., Pickett C. J. (2005). Nature.

[cit1657] Gloaguen F., Rauchfuss T. B. (2009). Chem. Soc. Rev..

[cit1658] Fisher B. F., Eisenberg R. (1980). J. Am. Chem. Soc..

[cit1659] Collin J.-P., Abdelaziz J., Sauvage J.-P. (1988). Inorg. Chem..

[cit1660] Bernhardt P. V., Jones L. A. (1999). Inorg. Chem..

[cit1661] Houlding V., Geiger T., Kölle U., Grätzel M. (1982). J. Chem. Soc., Chem. Commun..

[cit1662] Connolly P., Espenson J. H. (1986). Inorg. Chem..

[cit1663] Razavet M., Artero V., Fontecave M. (2005). Inorg. Chem..

[cit1664] Hu X., Cossairt B. M., Brunschwig B. S., Lewis N. S., Peters J. C. (2005). Chem. Commun..

[cit1665] Hu X., Brunschwig B. S., Peters J. C. (2007). J. Am. Chem. Soc..

[cit1666] Baffert C., Artero V., Fontecave M. (2007). Inorg. Chem..

[cit1667] Pantini O., Naskar S., Guillot R., Millet P., Anxolabehere-Mallart E., Aukauloo A. (2008). Angew. Chem., Int. Ed..

[cit1668] Jacques P.-A., Artero V., Pecaut J., Fontecave M. (2009). Proc. Natl. Acad. Sci. U. S. A..

[cit1669] Anxolabehere-Mallart E., Costentin C., Fournier M., Nowak S., Robert M., Saveant J.-M. (2012). J. Am. Chem. Soc..

[cit1670] McCrogy C. C.-L., Uyeda C., Peters J. C. (2012). J. Am. Chem. Soc..

[cit1671] Lay P. A., Mau A., Sasse W. H.-F., Creaser I. I., Gahan L. R., Sargeson A. M. (1983). Inorg. Chem..

[cit1672] Kellet R. M., Spiro T. G. (1985). Inorg. Chem..

[cit1673] Kellet R. M., Spiro T. G. (1985). Inorg. Chem..

[cit1674] Wilson A. D., Newell R. H., McNevin M. J., Muckerman J. T., Rakowski DuBois M., DuBois D. L. (2005). J. Am. Chem. Soc..

[cit1675] Pool D. H., DuBois D. L. (2009). J. Organomet. Chem..

[cit1676] Rakowski DuBois M., DuBois D. L. (2009). Chem. Soc. Rev..

[cit1677] Le Goff A., Artero V., Jousselme B., Tran P. D., Guillet N., Metaye R., Fihri A., Palacin S., Fontecave M. (2009). Science.

[cit1678] Parkin G., Bercaw J. E. (1989). J. Am. Chem. Soc..

[cit1679] Karunadasa H. I., Chang C. J., Long J. R. (2010). Nature.

[cit1680] Sellmann D., Geck M., Moll M. (1991). J. Am. Chem. Soc..

[cit1681] Sellmann D., Fursattel A. (1999). Angew. Chem., Int. Ed..

[cit1682] Sellmann D., Geipel F., Moll M. (2000). Angew. Chem., Int. Ed..

[cit1683] Sellmann D., Geipel F., Lauderbach F., Heinemann F. W. (2000). Angew. Chem., Int. Ed..

[cit1684] Lyaskovskyy V., de Bruin B. (2012). ACS Catal..

[cit1685] Denny J. A., Darensbourg M. Y. (2015). Chem. Rev..

[cit1686] Kaeffer N., Morozan A., Fize J., Martinez E., Guetaz L., Artero V. (2016). ACS Catal..

[cit1687] Wang X.-L., Tian Y., Chang Z.-H., Lin H. (2020). ACS Sustainable Chem. Eng..

[cit1688] Chen Z., Cui Y., Ye C., Liu L., Wu X., Sun Y., Xu W., Zhu D. (2020). Chem. – Eur. J..

[cit1689] Wang P., Liang G., Smith N., Hill K., Donnadieu B., Webster C. E., Zhao X. (2020). Angew. Chem., Int. Ed..

[cit1690] Dolganov A. V., Tanaseichuk B. S., Yurova V. Y., Chernyaeva O. Y., Okina E. V., Balandina A. V., Portnova E. A., Kozlov A. S., Solovyova E. O., Yudina A. D., Akhmatova A. A., Lyukshina Y. I. (2019). Int. J. Hydrogen..

[cit1691] Queyriaux N., Sun D., Fize J., Pecaut J., Field M. J., Chavarot-Kerlidou M., Artero V. (2020). J. Am. Chem. Soc..

[cit1692] Tang H., Brothers E. N., Grapperhaus C. A., Hall M. B. (2020). ACS Catal..

[cit1693] Popescu C. V., Ding S., Ghosh P., Hall M. B., Cohara M. (2019). Inorg. Chem..

[cit1694] Tsay C., Ceballos B. M., Yang J. Y. (2019). Organometallics.

[cit1695] Ho X. L., Das S. P., Kia-Sheun Ng L., Yun Ru Ng A., Ganguly R., Sen Soo H. (2019). Organometallics.

[cit1696] Zhang Y.-Q., Liao R. Z. (2017). Phys. Chem. Chem. Phys..

[cit1697] Ghosh P., Ding S., Chupik R. B., Quiroz M., Hsieh C.-H., Bhuvanesh N., Hall M. B., Darensbourg M. Y. (2017). Chem. Sci..

[cit1698] Haddad A. Z., Cronin S. P., Mashuta M. S., Buchanan R. M., Grapperhaus C. A. (2017). Inorg. Chem..

[cit1699] Harshan A. K., Solis B. H., Winkler J. R., Gray H. B., Hammes-Schiffer S. (2016). Inorg. Chem..

[cit1700] Li X., Zhang Y., Wang W., Meng J., Li K., Lin W., Peng Z., Wan J., Hu Z. (2019). Int. J. Hydrogen Energy.

[cit1701] Chen J., Wang H., Gong Y., Wang Y. (2019). J. Mater. Chem. A.

[cit1702] Sun Z., Yang M., Wang Y., Hang Hu Y. (2019). ACS Appl. Energy Mater..

[cit1703] Jaramillo T. F., Jorgensen K. P., Bonde J., Nielsen J. H., Horch S., Chorkendorff I. (2007). Science.

[cit1704] Voiry D., Yamaguchi H., Li J., Silva R., Alves D. C.-B., Fujita T., Chen M., Asefa T., Shenoy V. B., Eda G., Chhowalla M. (2013). Nat. Mater..

[cit1705] Kwak I. H., Kwon I. S., Abbas H. G., Jung G., Lee Y., Debela T. T., Yoo S. J., Kim J. G., Park J., Kang H. S. (2018). Nanoscale.

[cit1706] Maitra U., Gupta U., De M., Datta R., Govindaraj A., Rao C. N.-R. (2013). Angew. Chem., Int. Ed..

[cit1707] Presolski S., Wang L., Loo A. H., Ambrosi A., Lazar P., Ranc V., Otyepka M., Zboril R., Tomanec O., Ugolotti J., Sofer Z., Pumera M. (2017). Chem. Mater..

[cit1708] Kwak I. H., Kwon I. S., Abbas H. G., Seo J., Jung G., Lee Y., Kim D., Ahn J.-P., Park J., Kang H. S. (2019). J. Mater Chem. A.

[cit1709] Vincent I., Bessarabov D. (2018). Renewable Sustainable Energy Rev..

[cit1710] LeRoy R. L. (1983). J. Electrochem. Soc..

[cit1711] Millet P., Water Electrolysis P. E. M. (2015). Hydrogen Prod..

[cit1712] Verdin B., Fouda-Onana F., Germe S., Serre G., Jacques P. A., Millet P. (2017). Int. J. Hydrogen Energy.

[cit1713] Babic U., Suermann M., Büchi F. N., Gubler L., Schmidt T. J. (2017). J. Electrochem. Soc..

[cit1714] Bernt M., Siebel A., Gasteiger H. A. (2018). J. Electrochem. Soc..

[cit1715] Makharia R., Mathias M. F., Baker D. R. (2005). J. Electrochem. Soc..

[cit1716] Pang X., Davis J. T., Harvey A. D., Esposito D. V. (2020). Energy Environ. Sci..

[cit1717] Wang M., Wang Z., Gong X., Guo Z. (2014). Renewable Sustainable Energy Rev..

[cit1718] Pivovar B. S., Kim Y. S. (2007). J. Electrochem. Soc..

[cit1719] Zhao S., Yu H., Maric R., Danilovic N., Capuano C. B., Ayers K. E., Mustaina W. E. (2015). J. Electrochem. Soc..

[cit1720] Neyerlin K. C., Gu W., Jorne J., Clark A., Gasteiger H. A. (2007). J. Electrochem. Soc..

[cit1721] Millet P. , in PEM Electrolysis for Hydrogen Production: Principles and Applications, ed. D. Bessarabov, H. Wang, H. Li and N. Zhao, CRC press, Boca Raton, London, 2016, pp. 401

[cit1722] Lopata J., Kang Z., Young J., Bender G., Weidner J. W., Shimpalee S. (2020). J. Electrochem. Soc..

[cit1723] David M., Ocampo-Martínez C., Sánchez-Peña R. (2019). J. Energy Storage.

[cit1724] Trinke P., Bensmann B., Hanke-Rauschenbach R. (2017). Int. J. Hydrogen Energy.

[cit1725] Tong W., Forster M., Dionigi F., Dresp S., Sadeghi Erami R., Strasser P., Cowan A. J., Farràs P. (2020). Nat. Energy.

[cit1726] Inaba M., Kinumoto T., Kiriake M., Umebayashi R., Tasaka A., Ogumi Z. (2006). Electrochim. Acta.

[cit1727] Bensmann B., Hanke-Rauschenbach R., Sundmacher K. (2014). Int. J. Hydrogen Energy.

[cit1728] Gasteiger H. A., Panels J. E., Yan S. G. (2004). J. Power Sources.

[cit1729] Schalenbach M. (2016). Int. J. Hydrogen Energy.

[cit1730] Schalenbach M., Carmo M., Fritz D. L., Mergel J., Stolten D. (2013). Int. J. Hydrogen Energy.

[cit1731] Immerz C., Bensmann B., Trinke P., Suermann M., Hanke-Rauschenbach R. (2018). J. Electrochem. Soc..

[cit1732] Parra-Restrepo J., Bligny R., Dillet J., Didierjean S., Stemmelen D., Moyne C., Degiovanni A., Maranzana G. (2020). Int. J. Hydrogen Energy.

[cit1733] Maranzana G., Moyne C., Dillet J., Didierjean S., Lottin O. (2010). J. Power Sources.

[cit1734] Durst J., Lamibrac A., Charlot F., Dillet J., Castanheira L. F., Maranzana G., Dubau L., Maillard F., Chatenet M., Lottin O. (2013). Appl. Catal., B.

[cit1735] Dubau L., Castanheira L., Chatenet M., Maillard F., Dillet J., Maranzana G., Abbou S., Lottin O., De Moor G., El Kaddouri A., Bas C., Flandin L., Rossinot E., Caqué N. (2014). Int. J. Hydrogen Energy.

[cit1736] Dubau L., Castanheira L., Maillard F., Chatenet M., Lottin O., Maranzana G., Dillet J., Lamibrac A., Perrin J.-C., Moukheiber E., ElKaddouri A., De Moor G., Bas C., Flandin L., Caqué N. (2014). WIRES Energy Environ..

[cit1737] Abbou S., Dillet J., Maranzana G., Didierjean S., Lottin O. (2017). J. Power Sources.

[cit1738] Nandjou F., Poirot-Crouvezier J. P., Chandesris M., Blachot J. F., Bonnaud C., Bultel Y. (2016). J. Power Sources.

[cit1739] Nandjou F., Poirot-Crouvezier J. P., Chandesris M., Rosini S., Hussey D. S., Jacobson D. L., LaManna J. M., Morin A., Bultel Y. (2016). Int. J. Hydrogen Energy.

[cit1740] Biswas I., Sánchez D. G., Schulze M., Mitzel J., Kimmel B., Gago A. S., Gazdzicki P., Friedrich K. A. (2020). Energies.

[cit1741] Frensch S. H., Olesen A. C., Araya S. S., Kær S. K. (2018). Electrochim. Acta.

[cit1742] Job N., Chatenet M., Berthon-Fabry S., Hermans S., Maillard F. (2013). J. Power Sources.

[cit1743] Tian M., Cousins C., Beauchemin D., Furuya Y., Ohma A., Jerkiewicz G. (2016). ACS Catal..

[cit1744] Chen J. G., Jones C. W., Linic S., Stamenkovic V. R. (2017). ACS Catal..

[cit1745] Chatenet M., Benziger J., Inaba M., Kjelstrup S., Zawodzinski T., Raccichini R. (2020). J. Power Sources.

[cit1746] Benck J. D., Hellstern T. R., Kibsgaard J., Chakthranont P., Jaramillo T. F. (2014). ACS Catal..

[cit1747] Schmidt T. J., Gasteiger H. A., Stab G. D., Urban P. M., Kolb D. M., Behm R. J. (1998). J. Electrochem. Soc..

[cit1748] Green C. L., Kucernak A. (2002). J. Phys. Chem. B.

[cit1749] Green C. L., Kucernak A. (2002). J. Phys. Chem. B.

[cit1750] Schulenburg H., Durst J., Muller E., Wokaun A., Scherer G. G. (2010). J. Electroanal. Chem..

[cit1751] Grdeń M., Alsabet M., Jerkiewicz G. (2012). ACS Appl. Mater. Interfaces.

[cit1752] Bates M. K., Jia Q., Doan H., Liang W., Mukerjee S. (2016). ACS Catal..

[cit1753] Bergmann A., Jones T. E., Martinez Moreno E., Teschner D., Chernev P., Gliech M., Reier T., Dau H., Strasser P. (2018). Nat. Catal..

[cit1754] Audichon T., Mayousse E., Morisset S., Morais C., Comminges C., Napporn T. W., Kokoh K. B. (2014). Int. J. Hydrogen Energy.

[cit1755] Shang X., Hu W. H., Li X., Dong B., Liu Y. R., Han G. Q., Chai Y. M., Liu C. G. (2017). Electrochim. Acta.

[cit1756] Dubouis N., Yang C., Beer R., Ries L., Voiry D., Grimaud A. (2018). ACS Catal..

[cit1757] Hong W. T., Risch M., Stoerzinger K. A., Grimaud A., Suntivich J., Shao-Horn Y. (2015). Energy Environ. Sci..

[cit1758] McCrory C. C.-L., Jung S., Ferrer I. M., Chatman S. M., Peters J. C., Jaramillo T. F. (2015). J. Am. Chem. Soc..

[cit1759] Geiger S., Kasian O., Ledendecker M., Pizzutilo E., Mingers A. M., Fu W. T., Diaz-Morales O., Li Z., Oellers T., Fruchter L., Ludwig A., Mayrhofer K. J.-J., Koper M. T.-M., Cherevko S. (2018). Nat. Catal..

[cit1760] El-Sayed H. A., Weiß A., Olbrich L. F., Putro G. P., Gasteiger H. A. (2019). J. Electrochem. Soc..

[cit1761] Hartig-Weiss A., Tovini M. F., Gasteiger H. A., El-Sayed H. A. (2020). ACS Appl. Energy Mater..

[cit1762] Li T., Kasian O., Cherevko S., Zhang S., Geiger S., Scheu C., Felfer P., Raabe D., Gault B., Mayrhofer K. J.-J. (2018). Nat. Catal..

[cit1763] Hong W. T., Stoerzinger K. A., Lee Y. L., Giordano L., Grimaud A., Johnson A. M., Hwang J., Crumlin E. J., Yang W., Shao-Horn Y. (2017). Energy Environ. Sci..

[cit1764] Grigoriev S. A., Millet P., Volobuev S. A., Fateev V. N. (2009). Int. J. Hydrogen Energy.

[cit1765] Wang J., Gao Y., Kong H., Kim J., Choi S., Ciucci F., Hao Y., Yang S., Shao Z., Lim J. (2020). Chem. Soc. Rev..

[cit1766] Yang Y., Xiong Y., Zeng R., Lu X., Krumov M., Huang X., Xu W., Wang H., Disalvo F. J., Brock J. D., Muller D. A., Abrunã H. D. (2021). ACS Catal..

[cit1767] O'Neil G. D., Christian C. D., Brown D. E., Esposito D. V. (2016). J. Electrochem. Soc..

[cit1768] Dastafkan K., Meyer Q., Chen X., Zhao C. (2020). Small.

[cit1769] Schillings J. , Doche O. and Deseure J., Proc. 21st International Congress of Chemical and Process Engineering, CHISA 2014 and 17th Conference on Process Integration, Modelling and Optimisation for Energy Saving and Pollution Reduction, PRES 2014, 2014

[cit1770] Bernt M., Schröter J., Möckl M., Gasteiger H. A. (2020). J. Electrochem. Soc..

[cit1771] Görlin M., Chernev P., De Araújo J. F., Reier T., Dresp S., Paul B., Krähnert R., Dau H., Strasser P. (2016). J. Am. Chem. Soc..

[cit1772] Han B., Grimaud A., Giordano L., Hong W. T., Diaz-Morales O., Yueh-Lin L., Hwang J., Charles N., Stoerzinger K. A., Yang W., Koper M. T. M., Shao-Horn Y. (2018). J. Phys. Chem. C.

[cit1773] Frydendal R., Seitz L. C., Sokaras D., Weng T. C., Nordlund D., Chorkendorff I., Stephens I. E. L., Jaramillo T. F. (2017). Electrochim. Acta.

[cit1774] Risch M., Grimaud A., May K. J., Stoerzinger K. A., Chen T. J., Mansour A. N., Shao-Horn Y. (2013). J. Phys. Chem. C.

[cit1775] Dionigi F., Zeng Z., Sinev I., Merzdorf T., Deshpande S., Lopez M. B., Kunze S., Zegkinoglou I., Sarodnik H., Fan D. (2020). et al.. Nat. Commun..

[cit1776] Nong H. N., Reier T., Oh H. S., Gliech M., Paciok P., Vu T. H. T., Teschner D., Heggen M., Petkov V., Schlögl R., Jones T., Strasser P. (2018). Nat. Catal..

[cit1777] Arrigo R., Hävecker M., Schuster M. E., Ranjan C., Stotz E., Knop-Gericke A., Schlögl R. (2013). Angew. Chem., Int. Ed..

[cit1778] Opitz A. K., Nenning A., Rameshan C., Rameshan R., Blume R., Hävecker M., Knop-Gericke A., Rupprechter G., Fleig J., Klötzer B. (2015). Angew. Chem., Int. Ed..

[cit1779] Opitz A. K., Nenning A., Rameshan C., Kubicek M., Götsch T., Blume R., Hävecker M., Knop-Gericke A., Rupprechter G., Klötzer B., Fleig J. (2017). ACS Appl. Mater. Interfaces.

[cit1780] Stoerzinger K. A., Favaro M., Ross P. N., Hussain Z., Liu Z., Yano J., Crumlin E. J. (2018). Top. Catal..

[cit1781] Streibel V., Hävecker M., Yi Y., Velasco Vélez J. J., Skorupska K., Stotz E., Knop-Gericke A., Schlögl R., Arrigo R. (2018). Top. Catal..

[cit1782] May K. J., Carlton C. E., Stoerzinger K. A., Risch M., Suntivich J., Lee Y. L., Grimaud A., Shao-Horn Y. (2012). J. Phys. Chem. Lett..

[cit1783] Kornienko N., Resasco J., Becknell N., Jiang C. M., Liu Y. S., Nie K., Sun X., Guo J., Leone S. R., Yang P. (2015). J. Am. Chem. Soc..

[cit1784] Deng Y., Yeo B. S. (2017). ACS Catal..

[cit1785] Kornienko N., Heidary N., Cibin G., Reisner E. (2018). Chem. Sci..

[cit1786] Elliott W., Salemmilani R., Mubeen S., Meinhart C. D., Stucky G. D., Moskovits M. (2019). J. Catal..

[cit1787] Bo X., Li Y., Chen X., Zhao C. (2020). Chem. Mater..

[cit1788] Wang M., Dong C. L., Huang Y. C., Shen S. (2020). ACS Catal..

[cit1789] Kuang Z., Liu S., Li X., Wang M., Ren X., Ding J., Ge R., Zhou W., Rykov A. I., Sougrati M. T., Lippens P. E., Huang Y., Wang J. (2021). J. Energy Chemistry.

[cit1790] Xu Q., Jiang H., Duan X., Jiang Z., Hu Y., Boettcher S. W., Zhang W., Guo S., Li C. (2021). Nano Lett..

[cit1791] Yamamoto N., Ohsaka T., Terashima T., Oyama N. (1990). J. Electroanal. Chem..

[cit1792] Trotochaud L., Ranney J. K., Williams K. N., Boettcher S. W. (2012). J. Am. Chem. Soc..

[cit1793] Enman L. J., Burke M. S., Batchellor A. S., Boettcher S. W. (2016). ACS Catal..

[cit1794] Morales-Guio C. G., Liardet L., Hu X. (2016). J. Am. Chem. Soc..

[cit1795] Cherevko S., Reier T., Zeradjanin A. R., Pawolek Z., Strasser P., Mayrhofer K. J. J. (2014). Electrochem. Commun..

[cit1796] Cherevko S., Geiger S., Kasian O., Kulyk N., Grote J. P., Savan A., Shrestha B. R., Merzlikin S., Breitbach B., Ludwig A., Mayrhofer K. J. J. (2016). Catal. Today.

[cit1797] Geiger S., Kasian O., Mingers A. M., Mayrhofer K. J. J., Cherevko S. (2017). Sci. Rep..

[cit1798] Cherevko S. (2018). Curr. Opin. Electrochem..

[cit1799] Kasian O., Geiger S., Li T., Grote J. P., Schweinar K., Zhang S., Scheu C., Raabe D., Cherevko S., Gault B., Mayrhofer K. J. J. (2019). Energy Environ. Sci..

[cit1800] Lee J. K., Lee C., Fahy K. F., Kim P. J., LaManna J. M., Baltic E., Jacobson D. L., Hussey D. S., Stiber S., Gago A. S., Friedrich K. A., Bazylak A. (2020). Energy Conv. Manag..

[cit1801] Suermann M., Takanohashi K., Lamibrac A., Schmidt T. J., Büchi F. N. (2017). J. Electrochem. Soc..

[cit1802] Morin A., Peng Z., Jestin J., Detrez M., Gebel G. (2013). Solid State ion.

[cit1803] Babcock E., Szekely N., Konovalova A., Lin Y., Appavou M. S., Mangiapia G., Revay Z., Stieghorst C., Holderer O., Henkensmeier D., Lehnert W., Carmo M. (2019). J. Membr. Sci..

[cit1804] Seweryn J., Biesdorf J., Schmidt T. J., Boillat P. (2016). J. Electrochem. Soc..

[cit1805] Panchenko O., Borgardt E., Zwaygardt W., Hackemüller F. J., Bram M., Kardjilov N., Arlt T., Manke I., Müller M., Stolten D., Lehnert W. (2018). J. Power Sources.

[cit1806] Bessarabov D. and Millet P., in PEM Water Electrolysis, ed. B. G. Pollet, Hydrogen Energy and Fuel Cells Primers, Elsevier Academic Press, ISBN: 9780128111451, 2018, vol. 1

[cit1807] Bessarabov D. and Millet P., PEM Water Electrolysis, ed. B. G. Pollet, Hydrogen Energy and Fuel Cells Primers, Elsevier Academic Press, ISBN: 9780081028308, 2018, vol. 2

[cit1808] Angulo A., van der Linde P., Gardeniers H., Modestino M., Rivas D. F. (2020). Joule.

[cit1809] Lin M. Y., Hourng L. W., Kuo C. W. (2012). Int. J. Hydrogen Energy.

[cit1810] Vogt H. (1983). J. Appl. Electrochem..

[cit1811] Vogt H., Balzer R. J. (2005). Electrochim. Acta.

[cit1812] Lao L., Ramshaw C., Yeung H. (2011). J. Appl. Electrochem..

[cit1813] Eigeldinger J., Vogt H. (2000). Electrochim. Acta.

[cit1814] Scott K. (2018). Renewable Sustainable Energy Rev..

[cit1815] Gatard V., Deseure J., Chatenet M. (2020). Curr. Opin. Electrochem..

[cit1816] Garcés-Pineda F. A., Blasco-Ahicart M., Nieto-Castro D., López N., Galán-Mascarós J. R. (2019). Nat. Energy.

[cit1817] Iida T., Matsushima H., Fukunaka Y. (2007). J. Electrochem. Soc..

[cit1818] Matsushima H., Iida T., Fukunaka Y. (2012). J. Solid State Electrochem..

[cit1819] Matsushima H., Iida T., Fukunaka Y. (2013). Electrochim Acta.

[cit1820] Lin M. Y., Hourng L. W. (2014). Int. J. Energy Res..

[cit1821] Islam M. H., Burheim O. S., Pollet B. G. (2019). Ultrason. Sonochem..

[cit1822] Rashwan S. S., Dincer I., Mohany A., Pollet B. G. (2019). Int. J. Hydrogen Energy.

[cit1823] Rashwan S. S., Dincer I., Mohany A. (2019). Int. J. Energy Res..

[cit1824] Cataldo F. (1992). J. Electroanal. Chem..

[cit1825] Walton D. J., Burke L. D., Murphy M. M. (1996). Electrochim. Acta.

[cit1826] McMurray H. N., Worsley D. A., Wilson B. P. (1998). Chem. Commun..

[cit1827] Pollet B., Lorimer J. P., Phull S. S., Mason T. J., Walton D. J., Hihn J.-Y., Ligier V., Wéry M. (1999). J. Appl. Electrochem..

[cit1828] Budischak C. , Honsberg C. and Opila R. L., Electroanalytic effects of ultrasound on a hydrogen evolution reaction in KOH, Conf. Rec. IEEE Photovolt. Spec. Conf., 2008

[cit1829] Li S.-D., Wang C. C., Chen C. Y. (2009). Electrochim. Acta.

[cit1830] Li J. , Xue J., Tan Z., Zheng Y., Zhang L. and Beijing T., Ultrasound-Assisted Electrolysis in NaOH Solution, EPD Congress, 2011, pp. 919–926

[cit1831] Lepesant M. , Sonoelectrochemical production of hydrogen for PEM fuel cell applications, Internship report, ENSICAEN: Caen, France, 2011

[cit1832] Symes D. , *Sonoelectrochemical (20 kHz) Production of hydrogen from aqueous solutions*, MSc thesis, University of Birmingham, Birmingham, UK, 2011. https://core.ac.uk/download/pdf/75322.pdf

[cit1833] Zadeh S. H. , *Sonoelectrochemical production of hydrogen via alkaline water electrolysis*, MSc thesis, University of Birmingham, Birmingham, UK, 2012. https://etheses.bham.ac.uk/id/eprint/4014/1/Zadeh13MRes.pdf

[cit1834] Zadeh S. H. (2014). J. Autom. Control. Eng..

[cit1835] Lin M.-Y., Hourng L.-W. (2014). J. Chinese Inst. Eng..

[cit1836] Pollet B. G., Foroughi F., Faid A. Y., Emberson D. R., Islam M. H. (2020). Ultrason. Sonochem..

[cit1837] Kaya M. F., Demir N., Albawabiji M. S., Taş M. (2017). Int. J. Hydrogen Energy.

[cit1838] Kaya M. F., Demira N., Rees N. V., El-Kharouf A. (2020). Applied Energy.

[cit1839] Spin-polarized Catalysts for Energy-Efficient AEM Water Electrolysis – SpinCat, https://cordis.europa.eu/project/id/964972, website visited on 22.03.21

[cit1840] Pollet B. G. and Ashokkumar M., Introduction to Ultrasound, Sonochemistry and Sonoelectrochemistry, Springer Nature Switzerland AG: Cham, Switzerland, 2019

[cit1841] Moriguchi N. (1934). J. Chem. Soc. Jpn..

[cit1842] Pollet B. G. , Power Ultrasound in Electrochemistry: From Versatile Laboratory Tool to Engineering Solution, Wiley, 2012

[cit1843] Mason T. J. , Sonochemistry: The uses of ultrasound in chemistry, in Ultrasound Angioplasty, Royal Society of Chemistry, Cambridge, 1990, pp. 25–54

[cit1844] Suslick K. S. (1990). Science.

[cit1845] Yasui K. , Acoustic Cavitation and Bubble Dynamics, in SpringerBriefs in Molecular Science: Ultrasound and Sonochemistry, ed. B. G. Pollet and M. Ashokkumar, Springer, 2018

[cit1846] Sasikala R., Jayakumar O. D., Kulshreshtha S. K. (2007). Ultrason. Sonochem..

[cit1847] Merouani S., Hamdaoui O., Rezgui Y., Guemini M. (2015). Int. J. Hydrogen Energy.

[cit1848] Shiklomanov I. , Water in crisis: A guide to the world's fresh water resources, Oxford University Press, 1993, ch. 2

[cit1849] Da B., Yu H., Ma H., Wu Z. (2018). Anti-Corros. Methods Mater..

[cit1850] Cristiani P., Perboni G., Debenedetti A. (2008). Electrochim. Acta.

[cit1851] Izumiya K., Akiyama E., Habazaki H., Kumagai N., Kawashima A., Hashimoto K. (1998). Electrochim. Acta.

[cit1852] Fujimura F., Izumiya K., Kawashima A., Habazaki H., Akiyama E., Kumagai N., Hashimoto K. (1999). J. Appl. Electrochem..

[cit1853] Fujimura F., Matsui T., Izumiya K., Kumagai N., Akiyama E., Habazaki H., Kawashima A., Asami K., Hashimoto K. (1999). Mater. Sci. Eng..

[cit1854] Habazaki H., Matsui T., Kawashima A., Asami K., Kumagai N., Hashimoto K. (2001). Scr. Mater..

[cit1855] Ghany N. A.-A., Kumagai N., Meguro S., Asami K., Hashimoto K. (2002). Electrochim. Acta.

[cit1856] Kato Z., Sato M., Sasaki Y., Izumiya K. (2014). Electrochim. Acta.

[cit1857] Kuang Y., Kenney M. J., Meng Y., Hung W.-H., Liu Y., Haung J. E., Prasanna R., Li P., Li Y., Wang L., Lin M.-C., McGehee M. D., Sun X., Dai H. (2019). PNAS.

[cit1858] Song H. J., Yoon H., Ju B., Lee D.-Y., Kim D.-W. (2020). ACS Catal..

[cit1859] Yu L., Wu L., McElhenny B., Song S., Luo D., Zhang F., Yu Y., Chen S., Ren Z. (2020). Energy Environ. Sci..

[cit1860] Ko J. S., Johnson J. K., Johnson P. I., Xia Z. (2020). ChemCatChem.

[cit1861] Huang W.-H., Lin C.-Y. (2019). Faraday Discuss..

[cit1862] Exner K. S., Anton J., Jacob T., Over H. (2014). Angew. Chem., Int. Ed..

[cit1863] Mills A. (1989). Chem. Soc. Rev..

[cit1864] Dionigi F., Reier T., Pawolek Z., Gliech M., Strasser P. (2016). ChemSusChem.

[cit1865] Crittenden J. C. , Rhodes Trussell R., Hand D. W., Howe K. J. and Tchobanoglous G., MWH's Water Treatment: Principles and Design, 3rd edn, 2012

[cit1866] Trasatti S. (1984). Electrochim. Acta.

[cit1867] Hansen H. A., Man I. C., Studt F., Abild-Pedersen F., Bligaard T., Rossmeisl J. (2010). Phys. Chem. Chem. Phys..

[cit1868] Surendranath Y., Dinca M., Nocera D. G. (2009). J. Am. Chem. Soc..

[cit1869] De Gusseme B., Hennebel T., Vanhaecke L., Soetaert M., Desloover J., Wille K., Verbeken K., Verstraete W., Boon N. (2011). Environ. Sci. Technol..

[cit1870] Call D., Logan B. E. (2008). Environ. Sci. Technol..

[cit1871] Logen B. E., Call D., Cheng S., Hamelers H. V.-M., Sleutels T. H.-J. A., Jeremiasse A. W., Rozendal R. A. (2008). Environ. Sci. Technol..

[cit1872] Liu H., Grot S., Logan B. E. (2005). Environ. Sci. Technol..

[cit1873] Rozendal R. A., Hamelers H. V.-M., Euverink G. J.-W., Metz S. J., Buisman C. J.-N. (2006). Int. J. Hydrogen Energy.

[cit1874] Logan B. E., Aelterman P., Hamelers B., Rozendal R., Schröder U., Keller J., Freguiac S., Verstraete W., Rabaey K. (2006). Environ. Sci. Technol..

[cit1875] Degaki A. H., Pereira G. F., Rocha-Filho R. C., Bocchi N., Biaggio S. R. (2014). Electrocatalysis.

[cit1876] Sires I., Garrido J. A., Brillas E. (2013). J. Electrochem..

[cit1877] Madsen H. T., Søgaard E. G., Muff J. (2015). Chemosphere.

[cit1878] Wu M., Zhao G., Li M., Liu L., Li D. (2009). J. hazardous materials.

[cit1879] Lacasa E., Tsolaki E., Sbokou Z., Rodrigo M. A., Mantzavinos D., Diamadopoulos E. (2013). Chem. Eng. J..

[cit1880] Panizza M., Brillas E., Comninellis C. (2008). J. Environ. Eng. Manage..

[cit1881] Meddemann T., Bulan A., Sivers M., Kunz U. (2018). J. Electrochem. Soc..

[cit1882] d’Amore-Domenech R., Santiago O., Leo T. J. (2020). Renewable Sustainable Energy Rev..

[cit1883] Shadle T. and Daley T.. “U.S. Navy Submarine Life Support Systems”. 1991. 10.4271/911329

[cit1884] Cembalest M. , “J.P. Morgan Tenth Annual Energy Paper”, 2020. https://tinyurl.com/jpmorgan-10aep

[cit1885] Deloitte, “Investing in hydrogen: Ready, set, net-zero”, 2020. https://tinyurl.com/deloitte-investing-in-hydrogen

[cit1886] HSBC Centre of Sustainable Finance, “Hydrogen for the future: Delivering zero-carbon in heavy industry”, 2020. https://tinyurl.com/hsbc-hydrogen-for-the-future

[cit1887] L. Collins, “Green Hydrogen: ITM Power's New Gigafactory will Cut Costs of Electrolysers by Almost 40%”, 2021. World-Energy. https://www.world-energy.org/article/15430.html

[cit1888] N. Bullard, “A Gigafactory for Hydrogen Could Be a Game-Changer”, 2021. Bloomberg. https://www.bloomberg.com/news/articles/2021-07-01/a-gigafactory-for-hydrogen-could-be-a-game-changer

[cit1889] IEA, “The Future of Hydrogen: Seizing today's opportunities”. 2019, International Energy Agency

[cit1890] Aurora Energy Research, “Hydrogen Market Attractiveness Report (HyMAR)”, 2021

[cit1891] IEA, “Hydrogen Projects Database”, 2020

[cit1892] ETC, “Making the Hydrogen Economy Possible: Accelerating Clean Hydrogen in an Electrified Economy”, 2021. Energy Transitions Commission

[cit1893] Gregory D. P. , Ng D. Y.-C. and Long G. M., The Hydrogen Economy, in Electrochemistry of Cleaner Environments, ed. J. O. Bockris, Springer, 1972

[cit1894] Momirlan M., Veziroglu T. N. (2002). Renewable Sustainable Energy Rev..

[cit1895] Sanderson H. , 2020. *Fuel-cell producers jump on new hydrogen ‘hype cycle’*. Financial Times

[cit1896] Bakker S. (2010). Energy Policy.

[cit1897] Dodds P. E., Staffell I., Hawkes A. D., Li F., Grünewald P., McDowall W., Ekins P. (2014). Int. J. Hydrogen Energy.

[cit1898] IEA, “Global EV Outlook”, 2021

[cit1899] Staffell I., Brett D., Brandon N., Hawkes A. (2012). Energy Environ. Sci..

[cit1900] Liebreich M. , “Hydrogen: The Use Case Ladder”, 2021. https://twitter.com/MLiebreich/status/1397210398252732433

[cit1901] Hydrogen Council, “Path to hydrogen competitiveness: A cost perspective”, 2020

[cit1902] Fuel Cells Bulletin, 2019, **10**, 10 10.1016/S1464-2859(19)30425-0

[cit1903] IRENA, “Green hydrogen cost reduction: Scaling up electrolysers to meet the 1.5 °C climate goal”, 2020, International Renewable Energy Agency

[cit1904] Smolinka T. , Wiebe N. and Thomassen M.. “Cost Break Down and Cost Reduction Strategies for PEM Water Electrolysis Systems”, 2017, 6th EUROPEAN PEFC & Electrolyser Forum

[cit1905] Glenk G., Reichelstein S. (2019). Nat. Energy.

[cit1906] Bertuccioli L. et al. *Fuel cells and hydrogen Joint undertaking Development of Water Electrolysis in the European Union*. (E4Tech/ Element Energy, 2014)

[cit1907] BNEF, “Hydrogen Economy Outlook”, 2020

[cit1908] Mayyas A. and Mann M., *Manufacturing Competitiveness Analysis for Hydrogen Refueling Stations and Electrolyzers* 2018, NREL: DOE Hydrogen and Fuel Cells Program 2018 Annual Merit Review and Peer Evaluation Meeting

[cit1909] Mayyas A., Mann M. (2019). Procedia Manuf..

[cit1910] Böhm H., Goers S., Zauner A. (2019). Int. J. Hydrogen Energy.

[cit1911] James J. , Towards sustainable ammonia production, 2019, TNO

[cit1912] IRENA, “Renewable Power Generation Costs in 2020”, 2021, International Renewable Energy Agency

[cit1913] Jansen M., Staffell I., Kitzing L., Quoilin S., Wiggelinkhuizen E., Bulder B., Riepin I., Müsgens F. (2020). Nat. Energy.

[cit1914] Schmidt O., Hawkes A., Gambhir A., Staffell I. (2017). Nat. Energy.

[cit1915] Staffell I., Green R. (2013). Int. J. Hydrogen Energy.

[cit1916] BCG, “Perspectives on experience”, 1970, Boston Consulting Group

[cit1917] Junginger M. , van Sark W. and Faaij A.. Technological learning in the energy sector: Lessons for policy, industry and science, Edward Elgar Publishing, 2010

[cit1918] Wright T. P. (1936). J. Aeronaut. Sci..

[cit1919] ITRPV. “Maturity report, 11th edition”, 2021

[cit1920] McDonald A., Schrattenholzer L. (2001). Energy Policy.

[cit1921] Neij L. (2008). Energy Policy.

[cit1922] Malhotra A., Schmidt T. S. (2020). Joule.

[cit1923] Saba S. M., Müller M., Robinius M., Stolten D. (2018). Int. J. Hydrogen Energy.

[cit1924] Staffell I., Green R. (2009). Int. J. Hydrogen Energy.

[cit1925] Schoots K., Kramer G. J., van der Zwaan B. C.-C. (2010). Energy Policy.

[cit1926] Wei M., Smith S. J., Sohn M. D. (2017). Appl. Energy.

[cit1927] McDowall W. , “Endogenous technology learning for hydrogen and fuel cell technology” in “UKSHEC II: Literature review, research questions and data”, 2012

[cit1928] Thomas C. E. and Kuhn Jr. I. F. “Electrolytic hydrogen production infrastructure options evaluation”. NREL, 1995. 10.2172/125028

[cit1929] Schoots K. (2008). Int. J. Hydrogen Energy.

[cit1930] Schmidt O., Gambhir A., Staffell I., Hawkes A., Nelson J., Few S. (2017). Int. J. Hydrogen Energy.

[cit1931] Grigoriev S. A., Fateev V. N., Bessarabov D. G., Millet P. (2020). Int. J. Hydrogen Energy.

[cit1932] Phillips R., Dunnill C. W. (2016). RSC Adv..

[cit1933] Staffell I. , Brett D. J.-L., Brandon N. P. and Hawkes A. D., Domestic Microgeneration: Renewable and Distributed Energy Technologies, Policies and Economics, Routledge, London, 2015

[cit1934] BloombergNEF, “Hydrogen Economy Outlook”, 2020

[cit1935] Deutsch M. and Graf A., “EU-wide innovation support is key to the success of electrolysis manufacturing in Europe”, 2019, Agora Energiewende

[cit1936] Schmidt O., Melchior S., Hawkes A., Staffell I. (2019). Joule.

[cit1937] Girish G. P., Vijayalakshmi S. (2013). Int. J. Bus. Manage..

[cit1938] Gissey G. C. , Grubb M., Staffell I., Agnolucci P. and Ekins P.. “Wholesale cost reflectivity of GB and European electricity prices”, 2018, Ofgem

[cit1939] Staffell I. (2017). Energy Policy.

[cit1940] Ward K. R., Green R., Staffell I. (2019). Energy Policy.

[cit1941] Ketterer J. C. (2014). Energy Economics.

[cit1942] Staffell I., Pfenninger S. (2016). Energy.

[cit1943] Pfenninger S., Staffell I. (2016). Energy.

[cit1944] Staffell I. , 2011. The Energy and Fuel Data Sheet. https://tinyurl.com/energy-fuel-data-sheet

[cit1945] McDonagh S., Ahmed S., Desmond C., Murphy J. D. (2020). Appl. Energy.

[cit1946] Guo X., Li X., Xu Z., He G., Miao P. (2020). Energy Storage Sci. Technol..

[cit1947] Lichner C. , “Electrolyzer overview: Lowering the cost of hydrogen and distributing its production”, 2020. PV Magazine

[cit1948] Speirs J., Balcombe P., Johnson E., Martin J., Brandon N., Hawkes A. (2018). Energy Policy.

[cit1949] Parkinson B., Balcombe P., Speirs J. F., Hawkes A. D., Hellgardt K. (2019). Energy Environ. Sci..

