# Supplementary material for: Water electrolysis: from textbook knowledge to the latest scientific strategies and industrial developments
Source: Chem Soc Rev. 2022 May 16;51(11):4583–762. doi: 10.1039/d0cs01079k (PMC9332215; doi:10.1039/d0cs01079k)
Supplement: CS-051-D0CS01079K-s001 [file CS-051-D0CS01079K-s001.pdf]

Electronic Supplementary Material (ESI) for Chemical Society Reviews.
This journal is © The Royal Society of Chemistry 2022

Supplemental Information for: Water Electrolysis: From textbook knowledge to the latest scientific strategies and industrial developments

Marian Chatenet[a], Bruno G. Pollet[b, c], Dario R. Dekel[d, e], Fabio Dionigi[f], Jonathan Deseure[a], Pierre Millet[g], ,

Richard D. Braatz[h], Martin Z. Bazant[h, i], Michael Eikerling[j,k], Iain Staffell[l],

Paul Balcombe[m], Yang Shao-Horn[n], and Helmut Schäfer[o*]

Address:

a: University Grenoble Alpes, CNRS, Grenoble-INP (Institute of Engineering, Univ. Grenoble Alpes), LEPMI 38000 Grenoble, France. Orcid.org/0000-0002-9673-4775 (Marian Chatenet). Orcid.org/0000-0002-2055-7653 (Jonathan Deseure).

b: Hydrogen Energy and Sonochemistry Research group, Department of Energy and Process Engineering, Faculty of Engineering, Norwegian University of Science and Technology (NTNU) NO-7491, Trondheim, Norway. Orcid.org/0000-0002-4928-7378 (Bruno G. Pollet).

C:Green Hydrogen Lab, Hydrogen Research Institute (HRI), Université Du Québec à Trois-Rivières (UQTR), 3351 Boulevard des Forges, Trois-Rivières, Québec G9A 5H7, Canada.

d: The Wolfson Department of Chemical Engineering, Technion – Israel Institute of Technology, Haifa, 3200003, Israel. Orcid.org/0000-0002-8610-0808 (Dario Dekel).

e: The Nancy & Stephen Grand Technion Energy Program (GTEP), Technion - Israel Institute of Technology, Haifa 3200003, Israel

f: Department of Chemistry, Chemical Engineering Division, Technical University Berlin,10623, Berlin, Germany. Orcid.org/0000-0002-0576-024X (Fabio Dionigi)

g: Institut de Chimie Moléculaire et des Matériaux d'Orsay (UMR 8182), University of Paris-Saclay, rue du Doyen Georges Poitou, bâtiment 410, 91400, Orsay, France. Orcid.org/0000-0002-0224-9868 (Pierre Millet).

h: Department of Chemical Engineering, Massachusetts Institute of Technology, Cambridge, Massachusetts 02139, United States. Orcid.org/0000-0003-4304-3484 (Richard D. Braatz). Orcid.org/0000-0002-8200-4501 (Martin Z. Bazant).

i: Department of Mathematics, Massachusetts Institute of Technology, 77 Massachusetts Avenue, Cambridge, Massachusetts 02139, United States.

j: Chair of Theory and Computation of Energy Materials, Division of Materials Science and Engineering, RWTH Aachen University, Intzestraße 5, 52072 Aachen, Germany Orcid.org/0000-0002-0764-8948 (Michael Eikerling).

k: Institute of Energy and Climate Research, IEK-13: Modelling and Simulation of Materials in Energy Technology, Forschungszentrum Jülich GmbH, 52425 Jülich, Germany.

l: Centre for Environmental Policy, Imperial College London, London, UK. Orcid.org/0000-0003-1012-7075 (Iain Staffell).

m: Division of Chemical Engineering and Renewable Energy, School of Engineering and Material Science, Queen Mary University of London, London, UK. Orcid.org/0000-0002-3490-0707 (Paul Balcombe).

n: Research Laboratory of Electronics and Department of Mechanical Engineering, Massachusetts Institute of Technology, Cambridge, Massachusetts 02139, United States. Orcid.org/0000-0001-8714-2121 (Yang Shao-Horn).

o: Institute of Chemistry of New Materials, The Electrochemical Energy and Catalysis Group, University of Osnabrück, Barbarastrasse 7, 49076 Osnabrück, Germany. Orcid.org/0000-0001-5906-3354 (Helmut Schäfer).

Email: helmut.schaefer@uos.de

Table S1. Chemical and mechanical properties of selected AEMs.

Membrane	Polymeric backbone	Functional group	Properties								Conduct-ivity meas-urement temp. (ºC)	Applied in EW	Refs.
			IEC[a]	Conduc-tivity (mS/cm)	Water uptake (wt%)	Swelling ratio (%)	Thickness (µm)	Tensile strength (Mpa)	Elonga-tion (%)	Young's modulus (MPa)			
PTP-75	[(terphenyl piperidinium)-co-(oxindole terphenylylene)]	Piperidinium	2.10	48.6	31.4	4.9[b]	45	29.2	6.8	466.7	20	+	1
PTP-85	[(terphenyl piperidinium)-co-(oxindole terphenylylene)]	Piperidinium	2.38	59.6	37.0	5.9[b]	45	34.2	24.2	620.2	20	+	1
PTP-90	[(terphenyl piperidinium)-co-(oxindole terphenylylene)]	Piperidinium	2.52	64.4	39.7	7.4	45	36.5	25.2	777.6	20	+	1
FAA3-30 (OH-) (FumaTech)[b]	N/A	N/A	1.7-2.1	40	N/A	N/A	25-35	25-40	20-40	N/A	N/A	-	2
FAA3-50 (OH-) (FumaTech) [b]	N/A	N/A	1.59, 1.85	40	17	9	47–53	25-40	20-40	N/A	N/A	+	2, 3
QMSV-0.16	poly(ST-co-VBC	TMA	1.04	0.264	16.7	10.9	60	N/A	N/A	N/A	25	+	4
QMSV-0.33	poly(ST-co-VBC	TMA	2.14	6.80	127.4	15.9	60	N/A	N/A	N/A	25	+	4
PFOTFPh-TMA-C6	(fluorene-alt-tetrafluorophenylene	trimethylammonium (TMA)	3.2	156	122	97	20-30	26.8	8	N/A	70	+	5, 6
PFOTFPh-TMA-C8	(fluorene-alt-tetrafluorophenylene	trimethylammonium (TMA)	2.9	117	74[d]	39	20-30	28.5	8	N/A	70	+	5, 6
PFOTFPh-TMA-C10	(fluorene-alt-tetrafluorophenylene	trimethylammonium (TMA)	2.7	101	67[d]	33	20-30	40.9	10	N/A	70	+	5, 6
GT69[c]	poly (norbornene) (PNB)	N,N,N′,N′- tetramethyl-1,6-hexanediamine (TMHDA),	3.38	178	115	N/A	N/A	N/A	N/A	N/A	80	+	7
GT72-5[c]	poly (norbornene) (PNB)	N,N,N′,N′- tetramethyl-1,6-hexanediamine (TMHDA),	3.50	175	96	N/A	N/A	N/A	N/A	N/A	80	+	7
GT74[c]	poly (norbornene) (PNB)	N,N,N′,N′- tetramethyl-1,6-hexanediamine (TMHDA),	3.56	160	103	N/A	35<br>50	N/A	N/A	N/A	80	+	7
PAP-TP-85	poly(aryl piperidinium) hydroxide	piperidinium	2.37	193	65	12	10-25	67	117	N/A	95	+	8, 9, 10
PAP-TP-85-MQN	poly(aryl piperidinium) hydroxide	piperidinium	3.2	150[d]	N/A	N/A	20	N/A	N/A	N/A	RT	+	8
PBI1-PVBC1-NMPD/OH	poly(vinylbenzyl chloride) (PVBC) cross-linked by	N-methylpiperidine	2.31	83	48	11	50	37.5[e]	16.2[e]	N/A	80	+	11

Membrane	Polymeric backbone	Functional group	Properties								Conduct-ivity meas-urement temp. (ºC)	Applied in EW	Refs.
			IEC[a]	Conduc-tivity (mS/cm)	Water uptake (wt%)	Swelling ratio (%)	Thickness (µm)	Tensile strength (Mpa)	Elonga-tion (%)	Young's modulus (MPa)			
	polybenzimidazole (PBI) and quaternized by N-methylpiperidine (NMPD) i												
PAni-0.87	Polyaniline	3,30 - iminobis(N,Ndimethylpropylamine	0.87	54.9	13.8	10.3		N/A	N/A	N/A	80	-	12
PAni-0.92	Polyaniline	3,30 - iminobis(N,Ndimethylpropylamine	0.92	90.1	18.1	11.7		N/A	N/A	N/A	80	-	12
PAni-1.03	Polyaniline	3,30 - iminobis(N,Ndimethylpropylamine	1.03	105	22.3	18.7	N/A	N/A	N/A	N/A	80	+	12
IPA [b]	N/A	N/A	1.27	32.4	23.1	15.6	N/A	N/A	N/A	N/A	80	+	12
AMB [b]	N/A	N/A	1.06	3.3	24.3	N/A	N/A	N/A	N/A	N/A	80	+	12
Tokuyama- A201 [b]	N/A	N/A	1.8	42	30	N/A	28[e]	96.4 [g]	61.7[g]	1123 [g]	(26,41)	+	2, 10, 13, 14
AEMION™-AF1-HNN8-50-X [b]	N/A	N/A	2.1-2.5	102	N/A	N/A	50	60 (dry I)	85-110 [j]	N/A	50	+	2, 15
AEMION™-AF1-HNN8-25-X [b]	N/A	N/A	2.1-2.5	131	N/A	N/A	25	60 (dry I)	85-110 [g]	N/A	50	+	2, 15
AEMION™-AF1-HNN5-50-X [b]	N/A	N/A	1.4-1.7	40	N/A	N/A	50	60 (dry I)	85-110 [g]	N/A	50	+	2
AF1-HNN5-25-X [b]	N/A	N/A	1.4-1.7	56	N/A	N/A	25	60 (dry I)	85[g]	N/A	50	+	2, 15
SUSTAINION® Sustainion 37–50 [b]	Copolymer of styrene and vinyl benzyl chloride	Tetramethyl Imidazole	N/A	80 [f]	N/A	cracks when dry	50	cracks when dry	cracks when dry	N/A	30	+	2, 19, 16, 17, 18, 19, 20, 21, 22, 23
HDPE-AEM	High density polyethylene	TMA	2.44	214 [g, i]	155 [g]	38	29	35	283	N/A	80	-	24, 25
LDPE-AEM	Low density polyethylene	TMA	2.54	290<br>201<br>304	149	27	28	23	69	N/A	110<br>105<br>120	-	25 , 24, 26, 27, 28, 29
C4-AEM	ETFE	pyrrolidinium	1.51	30	85	59	N/A	N/A	N/A	N/A	60	-	25, 30
SEBS-BTMA	polystyrene-b-poly(ethylene-co-butylene)-b-polystyrene (SEBS)	TMA	1.04	32	40	25.1	60	2.0	180.1	N/A	80	-	25, 31

Membrane	Polymeric backbone	Functional group	Properties								Conduct-ivity meas-urement temp. (ºC)	Applied in EW	Refs.
			IEC[a]	Conduc-tivity (mS/cm)	Water uptake (wt%)	Swelling ratio (%)	Thickness (µm)	Tensile strength (Mpa)	Elonga-tion (%)	Young's modulus (MPa)			
SEBS-CH2-QA-1.5	SEBS	Long flexible alkyl spacer	1.35	56	48	29.6	60	3.3	345.7	N/A	80	-	25, 31
XL100-SEBS-C5-TMA-0.8	SEBS	TMA with different degree of functionalization and cation tether length	1.50	65	28	10	60	7.0	400	N/A	80	-	25, 32
1:1 DCPD:1	a tetraalkylammonium-functionalized norbornene with dicyclopentadiene (DCPD)	tetraalkylammonium	1.40	28	N/A	N/A	N/A	2.3	26	N/A	50	-	25, 33
P1.25-OH	poly(norbornene)	pendant trimethylammonium cations	1.25	177	82	N/A	N/A	N/A	N/A	N/A	80		25, 34
XL35-rPNB-X60-Y40	Poly(bromopropyl norbornene)-blockpoly(butyl norbornene) diblock copolymers	TMHDA	2.20	109	100	28	N/A	2.5	52	N/A	80	-	25, 35
HC-$[1]_{498}$ $[2]_{200}$	PE	Imidazolium	1.69	134	115	17	N/A	N/A	N/A	N/A	80	-	25, 36
PNB-X62-Y38	The tetrablock copolymer, consisting of alternating butyl norbornene (BuNB) and bromopropyl norbornene (BPNB) blocks (two blocks each)	norbornene	2.21	102	71	N/A	50	N/A	N/A	N/A	80	-	25, 37
H22C9N	poly(olefin)s	trimethylamine with the alkyl group	1.43	70 [l]	177	40	N/A	N/A	N/A	N/A	80	-	25, 38
F20C9N	poly(olefin)s	trimethylamine with the alkyl group	1.21	91 [l]	109	26	70	N/A	N/A	N/A	80	-	25, 38
ATMPP	poly(phenylene)s	benzylic cations	2.39	18	156	N/A	N/A	N/A	N/A	N/A	22	-	25, 39
HTMA-DAPP	polyphenylene	hexamethyl trimethyl ammonium	2.6	120	58	N/A	26	>20	N/A	N/A	80	+	25, 40
QPAF (C6)-2	perfluoroalkylene and phenylene groups	TMA	1.14	96	45	N/A	50	24	218	N/A	80	-	25, 41
QP-QAF3	quinquephenylene and fluorene groups	pendant hexyltrimethylammonium	2.21	134	85	N/A	22	35	28	6	80	-	25, 42
PAImEE (12)	poly(arylimidazoliums)	Ethyl as alkyl chains	2.65	46 [g]	28j	26.1	25	64	28.7	1075	80	+	25, 43
BPN1	biphenyl	trifluoromethyl	2.61	122	130	40	25	35	140	N/A	80	-	25, 44, 45
p-TPN1	Para- terphenyl	trifluoromethyl	2.15	81	43	6	25	24	20	N/A	80	-	25, 44, 45
m-TPN1	meta-terphenyl	trifluoromethyl	2.15	127	45	10	25	30	38	N/A	80	-	25, 44, 45
FLN-55	Quaternized poly(fluorene)s	TMA	2.50	120	180	60	30	N/A	N/A	N/A	80	-	25

Membrane	Polymeric backbone	Functional group	Properties								Conduct-ivity meas-urement temp. (ºC)	Applied in EW	Refs.
			IEC[a]	Conduc-tivity (mS/cm)	Water uptake (wt%)	Swelling ratio (%)	Thickness (μm)	Tensile strength (Mpa)	Elonga-tion (%)	Young's modulus (MPa)			
PTPipQ6	poly(arylene)	piperidinium	2.04	111	44	N/A	60	N/A	N/A	N/A	80	-	25, 46
PTPipQ1	poly(arylene)	piperidinium	2.38	89	145	N/A	60	N/A	N/A	N/A	80	-	46
PVBC-MPy/15%PEK-cardo	poly(vinyl benzyl chloride) (PVBC-MPy) and poly(ether ketone-cardo)	methylpyrrolidinium	3.47	50.3	132	110	60	4.5	31.3	20.7	80	-	47
PVBC-MPy/25%PEK-cardo	(PVBC-MPy) and poly(ether ketone-cardo)	methylpyrrolidinium	3.10	37.7	59	61	60	9.5	33.3	30.9	80	-	47
PVBC-MPy/35%PEK-cardo	(PVBC-MPy) and poly(ether ketone-cardo)	methylpyrrolidinium	2.65	28.5	32	38	60	15.1	23.9	67.6	80	+	47
PVBC-MPy/45%PEK-cardo	(PVBC-MPy) and poly(ether ketone-cardo)	methylpyrrolidinium	2.34	15.4	24	26	60	22.1	19.3	110	80	-	47
PSF-TMA$^+$	polysulfone (PSF)	trimethylammonium	2.05	30.5	N/A	N/A	40-80	N/A	N/A	N/A	50	+	48, 49
PSF-DMP$^+$	polysulfone (PSF)	1,4-dimethylpiperazinium	1.51	14.4	230	N/A	40-80	N/A	N/A	N/A	50	-	49
xQAPS	PSF	Trimethylammonium Dimethyl diethyl ammonium as cross linker	1.34	60.5	N/A	4	N/A	N/A	N/A	N/A	80	+	50, 51, 52
PPO24-BIM (bromide form)	oly-(2,6-dimethyl-1,4-phenylene oxide)	mesityl-benzimidazole (BIM)	1.9	12	27	N/A	23	45.9	6.0	951	60	+	13
FAA3-PK-75 (bromide form) [b]	N/A	N/A	N/A	N/A	N/A	N/A	87	26.9	12.1	983	60	+	13, 53
PSEBS-CM-DABCO	polystyrene-block-poly(ethylene-ran-butylene)-block-polystyrene (PSEBS	1,4-diazabicyclo[2.2.2]octane (DABCO)	0.76	75	N/A	N/A	100	N/A	N/A	N/A	30	+	54
PAEK-APMP75	Poly (arylene ether) ketone	1-(3-aminopropyl)-4-methylpiperazin	1.10	6.82	38	N/A	55	12.6	30.4	N/A	60	+	55
PAEK-APMP100	Poly (arylene ether) ketone	1-(3-aminopropyl)-4-methylpiperazin	1.32	9.94	48	N/A	55	9.7	41	N/A	60	+	55
QPDTB	three monomers of methacrylate:2-dimethylaminoethyl methacrylate (DMAEMA), 2,2,2,-tri-fluoroethyl methacrylate (TFEMA), and butyl methacrylate (BMA)	Trimethyl amine	1.275	59	N/A	N/A	300	7.629	45.8	226	50	+	56, 57

Membrane	Polymeric backbone	Functional group	Properties								Conduct-ivity meas-urement temp. (ºC)	Applied in EW	Refs.
			IEC[a]	Conduc-tivity (mS/cm)	Water uptake (wt%)	Swelling ratio (%)	Thickness (µm)	Tensile strength (Mpa)	Elonga-tion (%)	Young's modulus (MPa)			
LDPE-g-VBC-Dab	Low density polyethylene (LDPE) grafted vinylbenzyl chloride (VBC	,4-diazabicyclo(2.2.2)octane (Dabco)	1.5	25	81	N/A	N/A	N/A	N/A	N/A	60	+	58
SEBS-Pi-73%	Polystyrene-block-poly (ethylene-ran-butylene)-block-polystyrene	N-Methylpipridine	1.19	10.09	59.13	25.03	N/A	4.2	600	N/A	30	+	59
PBP-ASU-PPO	poly(biphenyl piperidinium) (PBP)/6-azaspiro[5.5]undecane (ASU)-functionalized polyphenyl ether (ASU-PP	Piperidinium and ASU	2.61	128	120	21	50	N/A	N/A	N/A	80	+	60
Tokuyama- A901 [b]	N/A	N/A	1.8	N/A	N/A	N/A	9	N/A	N/A	N/A	N/A	+	61
FAA-3-PP-75	N/A	N/A	N/A	38	140	13-15	80	N/A	N/A	N/A	60	+	62
SEBS	polystyrene-b-poly (ethylene/butylene)-b-polystyrene	Trimethyl amine	1.9	140	N/A	N/A	120	N/A	N/A	N/A	50	+	63
PTFE+qPDTB-OH^-	Polytetrafluoroethylene (PTFE)	Quaternary ammonium Poly(DMAEMA-co-TFEMA-co-BMA)<br><br>(quaternary ammonium polymethacrylate)	1.02	34	N/A	146	30	10	10	406	50	+	64
mm-qPVBz/OH^-	Methylated melamine grafted poly vinyl benzylchloride (mm-qPVBz/Cl^-)	Amination with methylated melamine	N/A	27	N/A	N/A	70	12.1	14	142	60	+	65, 66
BPN1-100	poly(arylene)	N,N,N-trimethylpentan-1-ammonium	2.61	122	124	66	N/A	N/A	N/A	N/A	80	+	67
TPN1-100	poly(arylene)	N,N,N-trimethylpentan-1-ammonium	2.15	112	70	23	N/A	N/A	N/A	N/A	80	+	67
PAImEE(12)	poly(aryl)	Imidazolium	2.65	21.3	28.1	26.1	25	64.0	28.7	1075	22	+	43
PAImBB(14)	poly(aryl)	Imidazolium	2.3	8.5	12.2	14.0	20	65.2	20.4	1095	22	+	43
QPC-TMA	poly(carbazole)	polymer (poly(9-(6-(trimethylammonium bromide)hexyl)-9H-carbazole-co-1,1,1-trifluoroisopropane)	2.08<br>2.00	125	76	15	50	N/A	N/A	N/A	70	+	68
PVBC-MPy M2	poly(vinyl benzyl)	methylpyrrolidinium	2.02	32.8	28.5	23.8	80	9.4	16.5	N/A	80	+	69
PVBC-MPy M4	poly(vinyl benzyl)	methylpyrrolidinium	2.01	29.8	19.8	20.4	80	30.9	11.1	N/A	80	+	69

Membrane	Polymeric backbone	Functional group	Properties								Conductivity measurement temp. (ºC)	Applied in EW	Refs.
			IEC[a]	Conductivity (mS/cm)	Water uptake (wt%)	Swelling ratio (%)	Thickness (µm)	Tensile strength (Mpa)	Elongation (%)	Young's modulus (MPa)			
PVBC-MPy M6	poly(vinyl benzyl)	methylpyrrolidinium	1.97	30.5	21.5	17.0	80	20.6	8.9	N/A	80	+	69
MES-PBI (25 wt% KOH)	polybenzimidazole	poly(2,2' -(m-mesitylene)-5,5' -bibenzimidazole)	N/A	100	N/A	54	60	N/A	N/A	N/A	80	+	70
PPO-TMA	polyphenylene oxide (PPO)	trimethylamine (TMA)	2.1	52	104	N/A	N/A	6.3	1.9	N/A	70	+	71, 72
PPO-ABCO	polyphenylene oxide (PPO)	1-Azabicyclo[2.2.2]octane (ABCO)	1.9	39	147	N/A	N/A	10.3	2	N/A	70	+	71, 72
PISPVA46	poly(1-vinyl-3-imidazole-co-styrene) (PIS) co-poly(vinyl alcohol) (PVA)	imidazolium	1.65	90	101.1	18.6	55	15.5	316.5	N/A	60	+	73
PISPVA37	poly(1-vinyl-3-imidazole-co-styrene) (PIS) co-poly(vinyl alcohol) (PVA)	imidazolium	1.41	82	78.9	19.7	55	27.6	295.1	N/A	60	+	73
PISPVA28	poly(1-vinyl-3-imidazole-co-styrene) (PIS) co-poly(vinyl alcohol) (PVA)	imidazolium	1.13	74	49.7	18.7	55	30.8	216.9	N/A	60	+	73
QMter-co-Mpi-60%	ether-free polyarylene	piperidinium	1.65	8	37.15	11.89	N/A	N/A	N/A	N/A	30	-	74
QMter-co-Mpi-80%	ether-free polyarylene	piperidinium	2.10	21	49.39	17.77	N/A	N/A	N/A	N/A	30	-	74
QMter-co-Mpi-100%	ether-free polyarylene	piperidinium	2.42	37	78.92	29.00	N/A	11	24	N/A	30	+	74
BPi	PPO	piperidinium	1.94	18	29.0	9.5	50	32.7	2.7	N/A	20	-	75
SCPi	PPO	piperidinium	1.67	25	41.7	11	50	48.4	3.5	N/A	20	+	75
LSCPi	PPO	piperidinium	1.57	29	39.6	10.3	50	35.6	2.0	N/A	20	+	75
BTMA	PPO	Benzyltrimethyl ammonium	2.04	26	65.7	12	50	39.4	4.4	N/A	20	-	75
SCQA	PPO	side-chain-type	1.80	41	79.0	15	50	40.5	8.0	N/A	20	-	75
LSCQA	PPO	long side-chain-type	1.67	39	55.7	12	50	39.0	2.1	N/A	20	+	75
PSU-PVP75%	poly(arylene ether sulfone) and poly(vinylpyrrolidone)	pyrrolidone	N/A	N/A	N/A	N/A	A wide range	5	75	130	N/A	+	76
BPI-c-PVBC/OH 1:2	poly[2-2'-(m-phenylene)-5-5'-bibenzimidazole] co-poly(vinylbenzyl chloride) (PVBC)	DABCO	1.74	30	45	23	N/A	N/A	N/A	N/A	80	+	77

Membrane	Polymeric backbone	Functional group	Properties								Conduct-ivity meas-urement temp. (ºC)	Applied in EW	Refs.
			IEC[a]	Conduc-tivity (mS/cm)	Water uptake (wt%)	Swelling ratio (%)	Thickness (μm)	Tensile strength (Mpa)	Elonga-tion (%)	Young's modulus (MPa)			
BPI-c-PVBC/OH 1:3	poly[2-2'-(m-phenylene)-5-5'-bibenzimidazole] co-poly(vinylbenzyl chloride) (PVBC)	DABCO	1.97	44	52	24	N/A	N/A	N/A	N/A	80	+	77
ABPBI-c-PVBC/OH 1:2	Poly(2,5-benzimidazole) (ABPBI) co -poly(vinylbenzyl chloride) (PVBC)	DABCO	1.70	56	51	90	N/A	N/A	N/A	N/A	90	+	77
C-PVAf·ABPBI	Polyvinyl alcohol (PVA) nanofiber/ABPBI	polyvinyl alcohol (PVA) nanofibers crosslinked with glutaraldehyde (GA)	N/A	41	55	17	30-45	5.0	6	1033	80	+	78
C-PVA·ABPBI	Polyvinyl alcohol (PVA) /ABPBI	polyvinyl alcohol (PVA) nanofibers crosslinked with glutaraldehyde (GA)	N/A	48	67	14	30-45	2.8	16	64	80	-	78
QPAF-4	perfluoroalkylene and fuorene	hexyltrimethylammonium groups	1.47	86.2	105	N/A	50	22.7	269	N/A	80	-	79
PP-BTMA	Aryl-Ether	Polyaromatics	2	10	116	N/A	20-40	N/A	N/A	N/A	30	-	80
PP-HTMA	Aryl-Ether	Polyaromatics	2.4	14	109	N/A	20-40	N/A	N/A	N/A	30	-	80
PPA-HTMA	Aryl-Ether	Polyaromatics	2.1 Polyaromatics	15	453	N/A	20-40	N/A	N/A	N/A	30	-	80
PSBFP-TMA	poly(2,2' -spirobifluorene-alt-1,3-phenylene)	Trimethyl ammonium	1.2	23.1	11.1	17	N/A	N/A	N/A	N/A	70	-	81
PSBFBP-TMA	poly(2,20 -spirobifluorene-alt-4,4' -biphenylene)	Trimethyl ammonium	2.3	86.2	24.5	38	N/A	N/A	N/A	N/A	70	-	81
F-PAE	polyaromatics	BTMA	2.7	46	99	N/A	N/A	30	9.8	N/A	80	+	82
ATM-PP	polyaromatics	BTMA	1.7	37	70	N/A	N/A	42	27	N/A	80	+	82
PBI linear	Polybenzimidazol	Polybenzimidazol	N/A	50	N/A	19	N/A	94	74	2554	22	+	83
CL PBI (crosslinked)	Polybenzimidazol	Polybenzimidazol	N/A	50	N/A	N/A	N/A	36	1.7	2907	22	+	83
Thermally cured PBI	Polybenzimidazol	Polybenzimidazol	N/A	50	N/A	47	N/A	139	59	2914	22	+	83

Membrane	Polymeric backbone	Functional group	Properties								Conduct-ivity meas-urement temp. (ºC)	Applied in EW	Refs.
			IEC[a]	Conduc-tivity (mS/cm)	Water uptake (wt%)	Swelling ratio (%)	Thickness (μm)	Tensile strength (Mpa)	Elonga-tion (%)	Young's modulus (MPa)			
L-ABPBI (C_{KOH}= 1.9 M)	ABPBI	ABPBI	N/A	7	37.8	50	90-120	N/A	N/A	N/A	22	+	84
LC-ABPBI (C_{KOH}= 1.9 M	ABPBI	ABPBI	N/A	8	33.8	50	90-120	N/A	N/A	N/A	22	+	84
PQDP-1	poly (quinquephenylene-co-diphenylene	piperidinium	1.77	67.2	33	4.2	20-30	43	9.8	880	80	-	85
PQDP-3	poly (quinquephenylene-co-diphenylene	piperidinium	2.44	140.5	90	36.5	20-30	41	16.6	980	80	-	85
SDQEO	PPO	alkoxyl- extender-containing dual quaternary ammonium	1.13	87.3	120	30	50	10.5	27.3	N/A	80	-	86
PFTP	poly(fluorenyl aryl piperidinium)	piperidinium	2.81	208	45	16	20	84.5	25.6	1580	80	-	87
m-PTP-20Q	oly(aryl piperidinium) (PAP	piperidinium	3.06	144.2	51	6.25	60-70	N/A	N/A	N/A	80	-	88
O-PDQA-3	aryl-ether free poly(aryl piperidinium) (PBP)	piperidinium cations and ethylene oxide spacers	1.93	106	46.1	12.5	N/A	N/A	N/A	N/A	80	-	89
PP80N20	tetrakis(bromomethyl) monomers	spirocyclic QA	3.2	51.3	266	11	N/A	N/A	N/A	N/A	80	-	90
Q-CLP1	poly(benzimidazolium-imide)-	triazolium	1.36	84	58	17	N/A	15.37	7.80	N/A	80	-	91
BiPyBPEEK-50%	polyetheretherketone (PEEK)	pyridine	3.51	36.99	16	4.4	N/A	66.0	4.5	2400	80	-	92
QPAES/QBGO-3.0	poly (arylene ether sulfone) (QPAES)	1,4-diazabicyclo [2,2,2]octane and 1,6-dibromohexane, and subsequently used to preparemulti-cationic oligomer brushes-decorated graphene oxide	1.68	58.7	103	13.6	60	32.4	9.1	1360	80	-	93
PAEK-HQACz-0.7	poly(arylene ether ketone) copolymers	Long alkyl densely quaternized carbazole derivative pendant	1.88	98.1	46.4	13.5	N/A	32.5	43.5	N/A	80	-	94
PBP-BOP-ASU 8%	poly(4-((1,1′-biphenyl)-4-yl)piperidine)(PBP)	long-chain 3-(3-(1-(8-bromooctyl) piperidin-4-yl) propyl)- 6-azaspiro[5.5] undecan-6-ium bromide(BOP-ASU)	2.65	117	140	32	N/A	N/A	N/A	N/A	80	-	95
PBP-ASU	poly(4-((1,1′-biphenyl)-4-yl)piperidine)(PBP)	ASU	2.82	91	132.98	26	N/A	N/A	N/A	N/A	80	-	95

**Membrane**	**Polymeric backbone**	**Functional group**	**Properties**								**Conduct-ivity meas-urement temp. (ºC)**	**Applied in EW**	**Refs.**
			**IEC[a]**	**Conduc-tivity (mS/cm)**	**Water uptake (wt%)**	**Swelling ratio (%)**	**Thickness (μm)**	**Tensile strength (Mpa)**	**Elonga-tion (%)**	**Young's modulus (MPa)**			
CP2	poly(styrene-b-(ethylene-co-butylene)-b-styrene) (SEBS poly(styrene-b-(ethylene-co-butylene)-b-styrene) (SEBS poly(styrene-b-(ethylene-co-butylene)-b-styrene) (SEBS) Poly(styrene-b-(ethylene-co-butylene)-b-styrene) (SEBS)	branch polyethyleneimine (BPEI)	3.34	66.63	18.16	5.5	N/A	26.17	443	N/A	80	-	96
CP3	SEBS	BPEI	2.47	19.87	15.67	4.89	N/A	28.78	511	N/A	80	-	96
PPEEK:PEG 80:20	poly(ether ether ketone) (QPPEEK) and poly (ethylene glycol) (PEG) as the crosslinke	quaternary phosphonium	1.01	102	139	20	100	13.7	48	338	80	-	97
MPyPPO	PPO	N-methylpyrrolidinium	1.73	84	92.9	23.9	N/A	15.52	25.45	N/A	80	-	98
qPBPTT-5	Poly(biphenyl N-methyl-4- piperidone 1,1,1-Trifluoroacetone 1,3,5-Triphenylbenzene)	piperidine	1.93	116.92	61.71	19.41	30	10.89	8.26	N/A	80	-	99
m-TPNPiQA	poly(terphenylene) backbone	tethered with piperidinium groups	2.66	68.7	2.54	52.25	50	N/A	N/A	N/A	80	-	100
HyAEM-MP-180	polychloromethylstyrene-b-polyethylene-b-polychloromethylstyrene (PCMS-b-PE-b-PCMS)	benzylmethylpiperidinium	2.1	179	33	16.5	N/A	23.9	29.4	39.0	80	-	101
PPO-DMP	PPO	six-membered dimethyl piperidinium	1.98	71.8	125.7	29.6	50	43.7	2.7	N/A	80	-	102
PPO-ASU	PPO	ASU	1.85	76.5	148.6	42.0	50	20.4	1.3	N/A	80	-	102
NPPO-2QA-1.85	Azide-modified PPO (NPPO)	Alkyne side chain precursor containing terminal doubleQA groups (TABB)	1.85	47.22	33.70	8.93	N/A	N/A	N/A	N/A	30	-	103
AEM-9.09	benzonorbornadiene derivative (BenzoNBD-Bis(ImþBr?-ImþI?)) grafted	multi- imidazolium cations side-chains combined the rigid alkyl spacer and flexible alkoxy spacer	1.41	100.74	52	35.3	58	24.7	53.8	318.1	80	-	104
NAPAEK-Q-100	poly(arylene ether ketone)s	Naphthalene	1.46	74	24.3	4.5	N/A	44.43	6.39	2009	100	-	105

Membrane	Polymeric backbone	Functional group	Properties								Conduct-ivity meas-urement temp. (ºC)	Applied in EW	Refs.
			IEC[a]	Conduc-tivity (mS/cm)	Water uptake (wt%)	Swelling ratio (%)	Thickness (µm)	Tensile strength (Mpa)	Elonga-tion (%)	Young's modulus (MPa)			
VIB5/PMS2/PBI 0.5	N,N-butylvinylimidazolium with p-methylstyrene and polybenzimidazole,	Imidazolium	2.06	147	320.19 [j]	10.77 [j]	70	N/A	N/A	N/A	100	-	106
QPAE/GO-(APTS-c-PTMA) 0.7 wt%	quaternary ammonium functionalized graphene oxide (Q-GO) into quaternized poly(arylene ether) (QPAE) random copolymer containing 3-aminopropyl)trie-thoxysilane (APTS) and (3-bromopropyl)trimethyl ammonium bromide (PTMA)	PTMA	1.45	114.2	31.2	13.2	27	14.1	5.3	N/A	90	-	107
PDPF-DMP	PDPF	DMP	2.15	124	111	30	N/A	N/A	N/A	N/A	80	-	108
Q-PAES/PPO-55	Quaternized poly(arylene ether sulfone)/PPO belnded	triethylamine (TEA)	1.79	90.9	45.4	25.7		N/A	N/A	N/A	90	-	109
A-PEI-8	Alkalized poly(ether imide)	imidazolium	1.23	44.2	40.3	19.2	31	30.2	9.4	1400	90	-	110
PPO-22-3QA8F	a tri-quaternary ammonium side chain to the poly(phenylene oxide) (PPO) backbone	Terminal alkyne-containing 3-[(2-perfluorooctyl)ethoxy]prop-1- yne	1.58	83	10.2	3.7	20-30	N/A	N/A	N/A	90	-	111
QN-PAEK/rGO5.0wt %	poly(arylene ether ketone) (PAEK)/ reduced graphene oxide (rGO)	bearing fluorenyl group	1.32	116.8	79	26	30	40.8	4.9	400	90	-	112
QPAEK-CN-0.5	PAEK with various g-C3N4nanosheetsconten	alkaline quaternary ammonium groups and amine/imine groups	1.31	38.6	30.2	6.1	N/A	35.3	86	1370	90	-	113
GT82-5	poly (norbornene) (PNB)	N,N,N′,N′- tetramethyl-1,6-hexanediamine (TMHDA),	3.84	212	122	N/A	N/A	N/A	N/A	N/A	80	-	114
PPO5-4QPip-2.6	PPO	piperidinium	2.6	221	115	N/A	50	N/A	N/A	N/A	80	-	115
QPAF-DMBA	quaternaized aromatic/perfluoroaklyl copolymer (QPAF)	dimethylbutylamine (DMBA)	1.33	152	53	N/A	45	N/A	N/A	N/A	80	-	116
T20NC6NC5N	PPO	hexyl and pentyl spacers	2.52	176	135	18	100	N/A	N/A	N/A	80	-	
PBP-20Q4	PBP	Piperidinium	3.64	155	242	42	60	35	45	N/A	80	-	117
BeC30%-P	polyvinyl alcohol	grafted bis-crown ether	3.51	235	133						80	-	118

Table S2. *Ex-situ* alkaline stability data of selected AEMs.

Membrane	Temperature [◦C]	OH^- conc. [M]	Durability or half life time [h]	%degradation	Measurement Method	Applied in EW	Ref.
PTP-75	80	1	934	41	Conductivity	+	1
	80	1	934	47	NMR		
PTP-85	80	1	934	40	Conductivity	+	1
	80	1	934	36	NMR		
PTP-90	80	1	934	73	Conductivity	+	1
	80	1	934	62	NMR		
PAni-0.87	80	1	48	4	Dry weight ionic, conductivity, IEC	-	12
PAni-0.92	80	1	48	4		-	12
PAni-1.03	80	1	48	4		+	12
PFOTFPh-TMA-C6	80	8	168	13.8	conductivity	+	6
HDPE-AEM	80	RH ¼ 100% N_2 atmosphere	500	8	conductivity	-	25, 24
LDPE-AEM	80	RH ¼ 100% N_2 atmosphere	500	6.2	conductivity	-	25, 24, 26
C4-AEM	80	1	672	13	IEC		25
SEBS-BTMA	60	1	360	13.6	conductivity		25
SEBS-CH2-QA-1.5	60	1	360	7.7	conductivity		25
XL100-SEBS-C5-TMA-0.8	80	1	500	2.4	conductivity		25
P1.25-OH	80	0.1	239	53	conductivity		25
XL35-rPNB-X60-Y40	80	1	576	1.4	conductivity		25
HC-[1]498 [2]200	80	1	720	4.2	conductivity		25
PNB-X62-Y38	80	1	1200	0.8	conductivity		25
H22C9N	80	1	500	9.6	conductivity		25
F20C9N	80	1	500	8.9	conductivity		25
ATMPP	22	4	336	33	conductivity		25
HTMA-DAPP	80	4	336	4.6	conductivity		40, 25
	80	4	336	0	IEC		40, 25
	80	4	3000	39	conductivity		25
	80	4	3000	8	IEC		25
	80	0.5	11,160	72	conductivity		25

Membrane	Temperature [◦C]	OH^- conc. [M]	Durability or half life time [h]	%degradation	Measurement Method	Applied in EW	Ref.
QPAF (C6)-2	60	1	400	90	conductivity		25
QP-QAF3	80	1	1000	15	conductivity		25
PAImEE (12)	80	10	240	6.1	NMR		43, 25
BPN1	95	1	1440	8	IEC		25
m-TPN1	95	1	1440	2	Conductivity		25, 119
FLN-55	80	1	500	2	IEC		25
PTPipQ6	90	2	720	64	NMR		25
PAP-TP-85	100	1	2000	3	IEC		9, 25
PTPipQ1	90	2	360	5	IEC		46
PVBC-MPy/15%PEK-cardo	60	1	432	15	Conductivity	-	47
	80	1	432	9			
	60	6	432	16			
PVBC-MPy/25%PEK-cardo	60	1	432	5	Conductivity	-	47
	80	1	432	15			
	60	6	432	20			
PVBC-MPy/35%PEK-cardo	60	1	432	9	Conductivity	+	47
	80	1	432	15			
	60	6	432	19			
PVBC-MPy/40%PEK-cardo	60	1	432	7	Conductivity	-	47
	80	1	432	11			
	60	6	432	25			
$PST-TMA^+$	60	1	168	10	NMR	+	49
	60	1	168	37	Conductivity		
	60	2	168	44	Conductivity		
	60	6	168	13	NMR		
$PST-DMP^+$	60	1	168	25	NMR	+	49
	60	1	168	49	Conductivity		
	60	2	168	67	Conductivity		
	60	6	168	38	NMR		
PPO24-BIM (bromide form)	80	1	336	65	Conductivity	+	13
FAA-30	80	1	336	8	Conductivity	+	13
Tokuyama A201	80	1	336	0	Conductivity	+	13
PSEBS-CM-DABCO	50	3.8	168	13	Conductivity	+	54

Membrane	Temperature [◦C]	OH^- conc. [M]	Durability or half life time [h]	%degradation	Measurement Method	Applied in EW	Ref.
	50	3.8	168	8	IEC		
	60	3.8	168	47	Conductivity		
	60	3.8	168	33	IEC		
PAEK-APMP75	60	5.3	672	15	IEC	+	55
PAEK-APMP100	60	5.3	672	25	IEC	+	55
SEBS-Pi-73%	80	1	576	25	Conductivity	+	59
				22	IEC		
PBP-ASU-PPO	80	1	2000	13.6	IEC	+	60
BPN1-100	95	1	1440	11	IEC	+	25, 67
	95	1	1440	8	NMR		
TPN1-100	95	1	1440	9	IEC	+	25, 44
	95	1	1440	2.3	NMR		
PAImBB(14)	80	10	240	2.3	NMR	+	43
QPC-TMA	80	1	1000	0	NMR<br>IEC	+	68
PVBC-MPy M2	80	1	600	13	Conductivity	+	69
PVBC-MPy M4	80	1	600	22	Conductivity	+	69
PVBC-MPy M6	80	1	600	18	Conductivity	+	69
PISPVA46	*60*	0.5	240	56.2	Conductivity	+	73
PISPVA37	60	0.5	240	44.9	Conductivity	+	73
PISPVA28	60	0.5	240	39.7	Conductivity	+	73
QMter-co-Mpi- 100%	60	1	43	6	IEC	+	74
	60	1	43	8	Conductivity		
BPi	80	1	560	48	Conductivity	-	75
SCPi	80	1	560	57	Conductivity	+	75
LSCPi	80	1	560	2	Conductivity	+	75
SCQA	80	1	560	71	Conductivity	-	75
LSCQA	80	1	560	16	Conductivity	+	75
BPI-c-PVBC/OH 1:2	60	1	480	10	IEC	+	77
BPI-c-PVBC/OH 1:3	60	1	480	16	IEC	+	77
ABPBI-c-PVBC/OH 1:2	60	1	480	7	IEC	+	77
QPAF-4	80	1	1000	0	Conductivity	-	78
PSBFP-TMA	80	1	168	0	Conductivity	-	81
PSBFBP-TMA	80	1	168	0	Conductivity	-	81
F-PAE	80	0.5	2	61% backbone	NMR	+	82

Membrane	Temperature [◦C]	OH^- conc. [M]	Durability or half life time [h]	%degradation	Measurement Method	Applied in EW	Ref.
				12% benzylic position			
ATM-PP	80	0.5	2	0	NMR	+	82
PPO-TMA	60	1	720	70	IEC	+	71, 72
	60	6	720	83	IEC		
PPO-ABCO	60	1	720	74	IEC	+	71, 72
PPO-ABCO	60	6	720	92	IEC		
PBI linear	85	6	4224	100	Conductivity	+	83
CL PBI (crosslinked)	85	6	4224	50	Conductivity	+	83
Thermally cured PBI	85	6	4224	0	Conductivity	+	83
MES-PBI (25 wt% KOH)	88	9.5	4968	3	Relative mass	+	70
PQDP-1	80	1	720	3	NMR	-	85
SDQEO	60	1	192	25	Conductivity	-	86
PFTP	80	1	2000	4	NMR	-	120
PFTP	80	5	2000	20	NMR	-	120
m-PTP-20Q	80	2	1600	10.55	Conductivity	-	88
O-PDQA-3	80	2	1080	4	Conductivity	-	89
O-PDQA-3	80	2	1080	2.8	IEC	-	89
PP80N20	80	1	1000	30.8	Conductivity	-	90
PP80N20	80	5	1000	41.1	Conductivity	-	90
Q-CLP1	80	2	300	46	Conductivity	-	121
BiPyBPEEK-50%	22	1	750	11	Conductivity	-	92
QPAES/QBGO-3.0	60	1	240	27	Conductivity	-	93
PAEK-HQACz-0.7	22	4	168	3.9	Conductivity	-	94
PBP-BOP-ASU 8%	80	2	1400	15.59	NMR	-	95
PBP-ASU	80	2	1400	11.37	NMR	-	95
CP3	60	2	480	20	NMR	-	96
PPEEK:PEG 80:20	80	1	400	15	Conductivity	-	97
MPyPPO	60	1	720	42	Conductivity	-	98
PPO-TMA	22	0.6 (λ=4)	646	8	NMR	-	122
qPBPTT-5	80	1	480	13.29	Conductivity	-	99
m-TPNPiQA	80	5	240	6	IEC	-	100
HyAEM-MP-180	22	9	168	50	IEC	-	101
NPPO-2QA-1.85	60	1	168	28	NMR	-	103
AEM-9.09	60	1	504	50	Conductivity	-	104

Membrane	Temperature [◦C]	OH^- conc. [M]	Durability or half life time [h]	%degradation	Measurement Method	Applied in EW	Ref.
ETFE-AEM	80	1	168	12	IEC	-	123
NAPAEK-Q-100	22	4	168	0.7	IEC	-	105
VIB5/PMS2/PBI0.5	25	2	204	25	Conductivity	-	106
QPAE/GO-(APTS-c-PTMA) 0.7 wt%	90	2	480	20	IEC	-	107
PDPF-DMP	90	2	2400	8.4	NMR	-	108
Q-PAES/PPO-55	50	2	1000	14.7	IEC	-	109
A-PEI-8	90	1	200	72.3	Conductivity	-	110
PPO-22-3QA8F	80	1	504	49	Conductivity	-	111
QN-PAEK/rGO5.0wt%	70	2	600	25	Conductivity	-	112
QPAEK-CN-0.5	60	1	240	11	Conductivity	-	113
GT82-15	80	1	1000	1.43	Conductivity	-	114
PPO5-4QPip-2.1	90	1	240	14	Conductivity, IEC	-	115
QPAF-DMBA	60	1	1000	42	Conductivity	-	116
T20NC6NC5N	80	1	500	10	Conductivity	-	
PBP-20Q4	80	2	1800	8.2	Conductivity	-	117

Table S3. AEMs and their performance in AEMWE cells using liquid electrolyte.

Membrane electrode assembly (MEA) components				Ionomer/ binder	Feed type	Cell Voltage (V)	Current density (A/cm^2)	Cell temperature (°C)	Ref.
Anode catalyst	Membrane type	Membrane thickness, μm	Cathode catalyst						
$CuCoO_3$	Tokuyama A201	28	Ni/CeO_2–La_2O_3/C	PTFE	1% $K_2CO_3/KHCO_3$	1.9	0.47	50	10
IrO_2	Fumatech FAA-3-PK-75	75	Pt/C	PTFE	0.5 M KOH	1.8	0.48	50	53
IrO_2	Fumatech FAA-3-PK-75	75	Pt/C	PTFE	0.5 M KOH	1.8	0.61	60	53
IrO_2	Fumatech FAA-3-PK-75	75	Pt/C	PTFE	0.5 M KOH	1.8	0.625	70	53
IrO_2	Fumatech FAA-3-PK-75	75	Pt/C	PTFE	0.5 M KOH	1.8	0.87	80	53
IrO_2	Fumatech FAA-3-PK-75	75	Pt/C	PTFE	0.5 M KOH	1.8	0.98	90	53
$Li_{0.21}Co_{2.79}O_4$	QPDTB	50	Ni	QPDTB	0.2 M KOH	2.2	0.3	20	56, 124
$Li_{0.21}Co_{2.79}O_4$	QPDTB	50	Ni	QPDTB	0.2 M KOH	2.05	0.3	40	56, 124
Ni/CPs	Tokuyama A201	28	Ni/CPs	—	1 M KOH	1.9	0.15	50	125
$NiFe_2O_4$	Tokuyama A201	28	NiFeCo	5% Nafion	1 M KOH	2.21	2.13	60	126
$NiFe_2O_4$	AEMION	38	NiFeCo	5% Nafion	1 M KOH	2.26	2.13	60	126
$NiFe_2O_4$	Sustanion	50	NiFeCo	5% Nafion	1 M KOH	2.13	2.13	60	126
$CuCoO_3$	LDPE-*g*-VBC	60	Ni/CeO_2–La_2O_3/C	PTFE	1% $K_2CO_3/KHCO_3$	2.1	0.46	50	58
IrO_2	SEBS-Pi	60	Pt/C		1 M KOH	2.0	0.4	50	31, 59
IrO_2	SEBS-Pi	60	Pt/C		1 M KOH	2.0	0.5	80	31, 59
Pd/TNTA web	Tokuyama A201	28	Pt/C	PTFE	2 M NaOH	2	2	80	127
$CuCoO_x$ (on Ni foam) Acta's 3030	Tokuyama A201	28	$Ni/(CeO_2$–$La_2O_3)/C$ on carbon paper Acta's 4030	I2	1% K_2CO_3	1.91	0.4	60	62

Membrane electrode assembly (MEA) components				Ionomer/ binder	Feed type	Cell Voltage (V)	Current density (A/cm^2)	Cell temperature (°C)	Ref.
**Anode catalyst**	**Membrane type**	**Membrane thickness, µm**	**Cathode catalyst**						
$CuCoO_x$ (on Ni foam)	Fumatech FAA-3	40	Ni/(CeO_2–La_2O_3)/C on carbon paper	I2	1% K_2CO_3	1.91	0.4	60	62
$CuCoO_x$ (on Ni foam)	Fumatech FAA-3-PP-75	80	Ni/(CeO_2–La_2O_3)/C on carbon paper	I2	1% K_2CO_3	1.99	0.4	60	62
$CuCoO_x$ (on Ni foam)	Tokuyama A901	10	Ni/(CeO_2–La_2O_3)/C on carbon paper	I2	1% K_2CO_3	2.1	0.5	50	61
$NiCo_2O_4$	Polyethylene based radiation grafted		Pt	Polystyrene-*b*-poly (ethylene/butylene) -*b*-polystyrene	0.1 M KOH	1.65	0.1	60	63
NiAl	HMT-PMBI	34	NiAlMo	—	1 M KOH	2.1	2	60	128
IrO_2	PTP-90	45	Pt/C		1 M NaOH	2.2	0.91	55	129
IrO_2	PTP-90	45	Pt/C		1 M NaOH	2.2	1	75	129
IrO_2	PTP-85	45	Pt/C		1 M NaOH	2.2	0.83	55	129
IrO_2	PTP-75	45	Pt/C		1 M NaOH	2.2	0.76	55	129
IrO_2	Sustainion 37-50	50	Pt/C		1 M KOH	1.63	1	60	16
NiFe	Sustainion 37-50	50	NiFeCo		1 M KOH	1.9	1	60	16
RANEYs-type-Ni	m-PBI	40	RANEYs-type-Ni Mo		24 wt% KOH	1.8	1.7	80	130
PtRu	PAImEE	13	Pt/C	PAI	6 M KOH	2.3	0.4	80	43
IrO_2	HTMA-DAPP	26	PtRu/C	9 wt%TMA	0.1 M NaOH	1.8	0.95	60	40
NiFe	HTMA-DAPP	26	PtRu/C	20 wt% TMA	0.1 M NaOH	1.8	3.2	60	40
NiFe	HTMA-DAPP	26	PtRu/C	20 wt%TMA	1 M NaOH	1.8	5.3	60	40
IrO_2	QPC-TMA	50	Pt/C	QPC-TMA	1 M KOH	1.9	3.5	70	68
IrO_2	GT74	50	Pt/C	GT-18	3 wt% KOH	1.59	0.1	50	7
IrO_2	GT74	50	Pt/C	GT-32	3 wt% KOH	1.62	0.1	50	7
IrO_2	GT74	50	Pt/C	GT-0	3 wt% KOH	1.69	0.1	50	7
IrO_2	GT74	50	Pt/C	GT-100	3 wt% KOH	1.83	0.1	50	7
IrO_2	GT74	50	Pt/C	GT-75	3 wt% KOH	2.08	0.1	50	7

Membrane electrode assembly (MEA) components				Ionomer/ binder	Feed type	Cell Voltage (V)	Current density (A/cm^2)	Cell temperature (°C)	Ref.
**Anode catalyst**	**Membrane type**	**Membrane thickness, µm**	**Cathode catalyst**						
IrO_2	GT74	50	Pt/C	GT-82	3 wt% KOH	2.11	0.1	50	7
NiFe	PVBC- MPy M2	80	NiMo	--------	1 M KOH	1.9	0.5	80	69
NiFe	PVBC- MPy M4	80	NiMo	--------	1 M KOH	1.9	0.4	80	69
NiFe	PVBC- MPy M6	80	NiMo	--------	1 M KOH	1.9	0.6	80	69
$NiFe_2O_4$	Sustainion 37–50		NiFeCo	PFOTFPh-TMA C6	1 M KOH	2.09	0.94	50	17
IrO_2	PFOTFPh-TMA C6	20-30	Pt/C	PFOTFPh-TMA C8	1 M KOH	1.77	1	80	5, 6
IrO_2	PFOTFPh-TMA C8	20-30	Pt/C	PFOTFPh-TMA C10	1 M KOH	1.79	1	80	5, 6
IrO_2	PFOTFPh-TMA C10	20-30	Pt/C	PFOTFPh-TMA C6	1 M KOH	1.84	1	80	5, 6
NiFe2O4	Sustainion™ X37-50	50	NiFeCo		1 M KOH	1.9	1	60	18
IrO_2	Sustainion™ X37-50	50	Pt/C		1 M KOH	1.63	1	60	18
$CuCoO_x$	Tokuyama A201	28	Pt/C	AS-4	0.1 wt% K_2CO_3	2.22	0.8	50	14
$CuCoO_x$	Tokuyama A201	28	Pt/C	AS-4	1 wt% K_2CO_3	2.05	0.8	50	14
$CuCoO_x$	Tokuyama A201	28	Pt/C	AS-4	10 wt% K_2CO_3	1.87	0.8	50	14
$CuCoO_x$	Tokuyama A201	28	Pt/C	AS-4	0.01 M KOH	2.06	0.8	50	14
Ir black	AF1-HNN8-25 Aemion™	25	Pt/C	Aemion™ AP1-HNN8	1 M KOH	1.75	1	50	15
Ir black	AF1-HNN8-50 Aemion™	50	Pt/C	Aemion™ AP1-HNN8	1 M KOH	1.8	1	50	15
Ir black	AF1-HNN5-25 Aemion™	25	Pt/C	Aemion™ AP1-HNN8	1 M KOH	1.81	1	50	15
Ir black	AF1-HNN5-50 Aemion™	50	Pt/C	Aemion™ AP1-HNN8	1 M KOH	1.92	1	50	15
Ir black	AF1-HNN8-25 Aemion™	25	Pt/C	Aemion™ AP1-HNN8	0.1 M KOH	1.85	1	50	15
Ir black	AF1-HNN8-50 Aemion™	50	Pt/C	Aemion™ AP1-HNN8	0.1 M KOH	1.9	1	50	15
Ir black	AF1-HNN5-25 Aemion™	25	Pt/C	Aemion™ AP1-HNN8	0.11 M KOH	1.98	1	50	15
Ir black	AF1-HNN5-50 Aemion™	50	Pt/C	Aemion™ AP1-HNN8	0.1 M KOH	2.13	1	50	15

Membrane electrode assembly (MEA) components				Ionomer/ binder	Feed type	Cell Voltage (V)	Current density (A/cm^2)	Cell temperature (°C)	Ref.
**Anode catalyst**	**Membrane type**	**Membrane thickness, µm**	**Cathode catalyst**						
Ir black	Fumatech FAA-3-PE-30	20-30	NiMo	Fumion FAA-3-solute-10	1 M KOH	1.9	1	50	131
Ir black	Fumatech FAA-3-PE-30	20-30	Pt/C	Fumion FAA-3-solute-10	1 M KOH	1.8	1	50	131
CuCoOx	Mg-Al LDH	300	Ni/(CeO_2-La_2O_3)/C	PTFE	0.1 M NaOH	2.1	0.16	60	132
CuCoOx	Mg-Al LDH	300	Ni/(CeO_2-La_2O_3)/C	PTFE	0.1 Na_2CO_3	2.1	0.1	60	132
$NiFe_2O_4$	Sustainion®37-50	50	NiFeCo	Nafion	1 M KOH	1.9	1	60	19
$NiFe_2O_4$	Fumatech FAS-50	50	NiFeCo	Nafion	1 M KOH	1.9	0.5	60	19
$NiFe_2O_4$	Fumatech FAPQ	68-82	NiFeCo	Nafion	1 M KOH	1.9	0.17	60	19
$NiFe_2O_4$	Neosepta ACM	110	NiFeCo	Nafion	1 M KOH	1.9	0.05	60	19
$NiFe_2O_4$	AMI 7001	18000	NiFeCo	Nafion	1 M KOH	1.9	0.15	60	19
$NiFe_2O_4$	Celazole®PBI		NiFeCo	Nafion	1 M KOH	1.9	0.07	60	19
IrO_2	Tokuyama A201	28	Pt/C	PTFE	0.5 M KOH	2	1.31	50	133
IrO_2	Sustainion™		Pt	PTFE	1 M KOH	1.9	4.6	60	134
Plain nickel foam	mes-PBI	64	Plain nickel foam		25 wt% KOH	2.3	0.7	80	70
Plain nickel foam	mes-PBI	60	Plain nickel foam		15 wt% KOH	2.3	0.42	80	70
Plain nickel foam	mes-PBI	62	Plain nickel foam		5 wt% KOH	2.3	0.01	80	70
Plain nickel foam	m-PBI	40	Plain nickel foam		20 wt% KOH	2.3	0.9	80	70
Ni foam	L-ABPBI (linear)	90-120	Ni foam		1.9 M KOH	2	0.155	50	84
Ni foam	C-ABPBI (cross-linked)	90-120	Ni foam		1.9 M KOH	2	0.18	50	84
Ni foam	L-ABPBI	90-120	Ni foam	PTFE	1.9 M KOH	2	0.18	70	84
Ni foam	C-ABPBI	90-120	Ni foam	PTFE	1.9 M KOH	2	0.22	70	84
Ni	L-PBI	90-120	Ni	PTFE	30 wt% KOH	2	0.15	80	83
Ni	C-PBI	90-120	Ni	PTFE	30 wt% KOH	2	0.12	80	83

Membrane electrode assembly (MEA) components				Ionomer/ binder	Feed type	Cell Voltage (V)	Current density (A/cm^2)	Cell temperature (°C)	Ref.
**Anode catalyst**	**Membrane type**	**Membrane thickness, µm**	**Cathode catalyst**						
CE-CCO, CuCo-oxide on nickel foam	X37-50 Grade T	50	Pt/C	PTFE	1 M KOH	1.8	1.39	45	22
IrO_2	Sustainion X37-50 Grade T	50	Pt/C	PTFE	1 M KOH	1.8	0.9	45	22
Cu0.5Co2.5O4	Sustainion X37-50 Grade T	50	Pt/C	PTFE	1 M KOH	1.8	1.3	45	23
IrO_2	Sustainion X37-50 Grade T	50	Pt/C	PTFE	1 M KOH	1.8	1.03	45	23
Ni foam	Sustainion X37-50 Grade T	50	Pt/C	PTFE	1 M KOH	1.8	0.35	45	23
IrO_2	PISPVA46	55	Pt/C	PTFE	0.5 M KOH	2	0.55	60	135
IrO_2	PISPVA37	55	Pt/C	PTFE	0.5 M KOH	2	0.41	60	135
IrO_2	PISPVA28	55	Pt/C	PTFE	0.5 M KOH	2	0.15	60	135
IrO_2	QMter-co-Mpi		Pt/C		0.6 M KOH	2	0.25	50	74
IrO_2	QMter-co-Mpi		Pt/C		1 M KOH	2	0.31	50	74
$NiCo_2O_4$	PSEBS-CM-DABCO	100	$NiFe_2O_4$	PSEBS-CM-DABCO	1 wt% KOH	2	0.065	40	54
$NiCo_2O_4$	PSEBS-CM-DABCO	100	$NiFe_2O_4$	PSEBS-CM-DABCO	5 wt% KOH	2	0.105	40	54
$NiCo_2O_4$	PSEBS-CM-DABCO	100	$NiFe_2O_4$	PSEBS-CM-DABCO	10 wt% KOH	2	0.128	40	54
$NiCo_2O_4$	PSEBS-CM-DABCO	100	$NiFe_2O_4$	PSEBS-CM-DABCO	15 wt% KOH	2	0.15	40	54
$NiFe_2O_4$	Sustainion® 37–50	50	NiFeCo		1 M KOH	1.9	1	60	19
$NiFe_2O_4$	Fumatech FAS-50	50	NiFeCo		1 M KOH	1.9	0.5	60	19
$NiFe_2O_4$	Fumatech FAPQ	75	NiFeCo		1 M KOH	1.9	0.16	60	19
$NiFe_2O_4$	AMI-7001	450	NiFeCo		1 M KOH	1.9	0.11	60	19
NiFe-LDH/NF	PVBC-MPy/35%PEK-cardo	60	MoNi/NF	PTFE	1 M KOH	2	0.5	60	47
IrO_2	Fumatech FAA-3-50	50	Pt/C		1 M KOH	2	0.63	60	136

Membrane electrode assembly (MEA) components				Ionomer/ binder	Feed type	Cell Voltage (V)	Current density (A/cm^2)	Cell temperature (°C)	Ref.
**Anode catalyst**	**Membrane type**	**Membrane thickness, µm**	**Cathode catalyst**						
g-CN-CNF-800	Fumatech FAA-3-50	50	Pt/C		1 M KOH	2	0.98	60	136
FeNiMo-based	Sustainion X37-50 Grade T	50	NiMo-based	Nafion	1 M KOH	1.57	1	80	20
FeNiMo-based	Sustainion X37-50 Grade T	50	NiMo-based	Nafion	1 M KOH	1.62	1	60	20
FeNiMo-based	Sustainion X37-50 Grade T	50	NiMo-based	Nafion	1 M KOH	1.68	1	40	20
FeNiMo-based	Sustainion X37-50 Grade T	50	NiMo-based	Nafion	1 M KOH	1.69	0.6	20	20
APS NiAl-AA	NEOSEPTA (Astom)	220	APS NiAlMo-AA		1 M KOH	1.88	0.5	65	137
APS NiAl-AA	NEOSEPTA (Astom)	220	APS NiAlMo-AA		0.7 M KOH	1.93	0.5	65	137
APS NiAl-AA	NEOSEPTA (Astom)	220	APS NiAlMo-AA		0.5 M KOH	1.95	0.5	65	137
APS NiAl-AA	NEOSEPTA (Astom)	220	APS NiAlMo-AA		0.3 M KOH	2	0.5	65	137
APS NiAl-AA	NEOSEPTA (Astom)	220	APS NiAlMo-AA		0.1 M KOH	2.14	0.5	65	137
NiFe2O4	Sustainion Grade T, PTFE reinforced	50	Raney nickel		1 M KOH	1.8	0.84	60	21
NiFe2O4	Sustainion X37-50	50	Raney nickel		1 M KOH	1.8	0.74	60	21
NiFe	PSU-PVP	120	NiMo		20 wt% KOH	1.9	0.5	80	76
Ni-doped FeOOH	Sustainion X37-50 Grade T	50	Pt/C	Nafion	1 M KOH	1.7	0.92	50	138
Ni-doped FeOOH	Sustainion X37-50 Grade T	50	Pt/C	Nafion	1 M KOH + 0.5 M NaCl	1.7	0.74	50	138
Ni-doped FeOOH	Sustainion X37-50 Grade T	50	Pt/C	Nafion	1 M KOH + seawater	1.7	0.73	50	138
IrO_2	Sustainion X37-50 Grade T	50	Pt/C	PTFE	1 M KOH + seawater	1.7	0.47	50	138
Ni foam	PBI-c-PVBC/OH (1:3)	24	Ni foam		1 M KOH	1.98	0.38	50	77

**Membrane electrode assembly (MEA) components**				**Ionomer/ binder**	**Feed type**	**Cell Voltage (V)**	**Current density (A/cm^2)**	**Cell temperature (°C)**	**Ref.**
**Anode catalyst**	**Membrane type**	**Membrane thickness, μm**	**Cathode catalyst**						
Ni foam	ABPBI-c-PVBC/OH (1:2)	90	Ni foam		1 M KOH	1.98	0.38	50	77
Ni foam	C-PVAf·ABPBI	30-45	Ni foam		15 wt% KOH	2	0.24	50	139
Ni foam	C-PVAf·ABPBI	30-45	Ni foam		15 wt% KOH	2	0.27	70	139
Graphene	Selemion AMV	120	Graphene		Water	2	0.09	30	140
$NiCo_2O_4$	AMB-commercial (now Pure Water Technologies)		$NiCo_2O_4$		1 M KOH	2	0.25	50	12
$NiCo_2O_4$	IPA- commercial (CSIR-CSMCRI)		$NiCo_2O_4$		1 M KOH	2	0.16	50	12
$NiCo_2O_4$	PAni-1.03	100	$NiCo_2O_4$		1 M KOH	2	0.4	50	12
PGM	BPN1-100		PGM	AS4	0.5 M NaOH	2.1	0.4	50	67
PGM	TPN1-100		PGM	AS4	0.5 M NaOH	2.35	0.36	50	67
$NiMn_2O_4$	Fumatech FAA3-50	50	Pt/KB		1 M KOH	2	0.24	40	3
$NiMn_2O_4$	Fumatech FAA3-50	50	Pt/KB		1 M KOH	2	0.37	50	3
$NiMn_2O_4$	Fumatech FAA3-50	50	Pt/KB		1 M KOH	2	0.47	60	3
$NiMn_2O_4$	Fumatech FAA3-50	50	Pt/KB		1 M KOH	2	0.53	80	3
IrO_2	HTMA-DAPP	50	PtRu/C	HTMA-DAPP	0.05 M KOH	2	0.7	60	141
IrO_2	HTMA-DAPP	50	PtRu/C	HTMA-DAPP	0.1 M KOH	2	1.22	60	141
IrO_2	HTMA-DAPP	50	PtRu/C	HTMA-DAPP	0.5 M KOH	2	1.4	60	141
IrO_2	HTMA-DAPP	50	PtRu/C	HTMA-DAPP	1 M KOH	2	1.74	60	141

Table S4. AEMs and their performance in AEMWE cells fed with pure water (no liquid electrolyte).

Membrane electrode assembly (MEA) components				Ionomer/binder	Feed type	Cell Voltage (V)	Current density A cm^{-2}	Cell temp. °C	Ref.
Anode catalyst	Membrane type	Membrane thickness, μm	Cathode catalyst						
$Pb_2Ru_2O_{6.5}$	PSF-TMA	50	Pt black	PSF-TMA^+Cl^-	Water	1.8	0.4	50	48
Ni–Fe	xQAPS	70	Ni–Mo	xQAPS	Water	1.85	0.4	70	52
$NiCoO_x$:Fe	Fumatech FAA-3	130	Pt/C	FAA-3	Water	2.0	0.37	50	142
IrO_2	Tokuyama A201	28	Pt black	As-4	Water	1.6	0.1	50	148
$Cu_{0.7}CO_{2.3}O_4$	QPDTB	50	Nano Ni	Poly(DMAEMA-*co*-TFEMA-*co*-BM)	Water	1.9	0.1	22	57
IrO_2	SEBS-Pi	60	Pt/C		Water	2	0.1	50	31, 59
$Ce_{0.2}MnFe_{1.8}O_4$	Fumatech FAA-3-PK-130	110-130	Pt on Ti	—	Water	1.8	0.3	—	143
$NiCo_2O_4$ on steel mesh	Homemade, not specified		Acta 4030	Homemade, not specified	Water	1.86	0.4	60	144
$Cu_xCo_{3-x}O_4$	PTFE-qPDTB-OH-	25	Pt/C	q-ammonium polymethacrylate	Water	2.0	0.4	22	64
IrO_2	HTMA-DAPP	26	PtRu/C	9 wt% TMA	Water	1.8	0.48	60	40
IrO_2	HTMA-DAPP	26	PtRu/C	9 wt% TMA	Water	1.8	0.79	85	40
NiFe	HTMA-DAPP	26	NiMo/C	20 wt% TMA	Water	1.8	0.9	85	40
NiFe	HTMA-DAPP	26	PtRu/C	20 wt% TMA	Water	1.8	2.7	85	40
NiFe-BTC-GNPs MOF	FAA-3-PK-130	130	MoNi4/MoO2		Water	1.85	0.54	70	145
IrO_2	Fumatech FAA-3-50	50	Pt/C	FAA-3-B	Water	1.85	0.12	50	146
IrO_2	QPC-TMA	50	Pt/C	QPC-TMA	Water	1.9	0.3	70	68
IrO_2	PFOTFPh-TMA C6	20-30	Pt/C	PFOTFPh-TMA C8	Water	1.94	1	80	5, 6
IrO_2	PFOTFPh-TMA C8	20-30	Pt/C	PFOTFPh-TMA C10	Water	1.95	1	80	5
IrO_2	PFOTFPh-TMA C10	20-30	Pt/C	PFOTFPh-TMA C6	Water	2.14	1	80	5, 6
$CuCoO_x$	Tokuyama A201	28	Pt/C	AS-4	Water	2.2	0.1	50	14

Membrane electrode assembly (MEA) components				Ionomer/binder	Feed type	Cell Voltage (V)	Current density A cm^{-2}	Cell temp. °C	Ref.
**Anode catalyst**	**Membrane type**	**Membrane thickness, µm**	**Cathode catalyst**						
$Cu_{0.7}Co_{2.3}O_4$	mm-qPVBz/Cl-	70	Ni nano powder	qPVB/Cl-	Water	2.19	0.1	25	65
$Cu_{0.7}Co_{2.3}O_4$	mm-qPVBz/Cl-	70	Ni nano powder	qPVB/Cl-	Water	2.05	0.1	40	65
$Cu_{0.7}Co_{2.3}O_4$	mm-qPVBz/Cl-	70	Ni nano powder	qPVB/Cl-	Water	1.99	0.1	55	65
CuCoOx	Mg-Al LDH	300	Ni/(CeO_2-La_2O_3)/C	PTFE	Water	2.1	0.02	60	132
IrO_2	PPO-TMA	40	Pt/C		Water	1.8	0.24	50	72
IrO_2	PPO-ABCO	40	Pt/C		Water	1.8	0.075	50	72
IrO_2	PISPVA46	55	Pt/C	PTFE	Water	2	0.06	60	135
IrO_2	QMter-co-Mpi		Pt/C		Water	2	0.06	50	74
IrO_2	LSCPi	50	Pt/C	LSCPi	Water	1.8	0.3	50	75
IrO_2	SCPi	50	Pt/C	SCPi	Water	1.8	0.2	50	75
Graphene	Selemion AMV	120	Graphene		Water	2	0.09	30	140
NiMPL-PTL	Sustainion X37-50 Grade T	50	NiMPL-PTL		Water	1.9	0.5	60	147
IrO_2	HTMA-DAPP	50	PtRu/C	HTMA-DAPP	Water	2	0.44	60	141

Table S5. AEMs and their performance stability in operando AEMWE cells.

Membrane	Anode catalyst	Cathode catalyst	Electrolyte	Temperature (°C)	Current density (A cm^{-2})	Durability (h)	Ref.
QMter-co-Mpi	IrO_2	Pt/C	5.6 wt% KOH	50	0.2	500 (2.1 V)	74
Tokuyama A201	IrO_2	Pt	Water	50	0.2	535 (2.25 V)	148
Tokuyama A201	Acta's 3030	Acta's 4030	1 wt% K_2CO_3/$KHCO_3$	43	0.47	1000 (2.05 V)	10
PSF-TMA	$Pb_2Ru_2O_{6.5}$	Pt	Pure water	50	0.2	6 (133.3 mV h^{-1})	48
xQAPS	Ni–Fe	Ni–Mo	Water	70	0.4	8 (1.8 V)	52
mes-PBI	Ni foam	Ni foam	25 wt% KOH	80	0.2	72 (1.95 V)	70
PSEBS-CM-DABCO	$NiCo_2O_4$	$NiFe_2O_4$	10 wt% KOH	50	0.3	150 (2.267 V)	54
Sustainion® 37–50	$NiFe_2O_4$	NiFeCo	5.6 wt% KOH	60	1	2000 (1.9 V)	19
Fumatech FAS-50	$NiFe_2O_4$	NiFeCo	5.6 wt% KOH	60	1	200 (400 μV h^{-1})	19
PAImEE(12)	Pt/C	Pt/C	33.6 wt% KOH	60	0.4	48 (2.6 V)	43
LSCPi	IrO_2	Pt/C	Water	50	0.2	35 (16.3 μV h^{-1})	75
PVBC-MPy/35%PEK-cardo	NiFe-LDH/NF	MoNi/NF	5.6 wt% KOH	60	0.5	46 (2.0 V)	47
SEBS-Pi	IrO_2	Pt/C	5.6 wt% KOH	50	0.4	100 (2.08 V)	59
Fumatech FAA-3	Acta's 3030	Acta's 4030	1 wt% K_2CO_3	60	0.5	31 (2.04 V)	62
Fumatech FAA-3-PP-75	Acta's 3030	Acta's 4030	1 wt% K_2CO_3	60	0.5	200 (2.38 μV h^{-1})	62
NEOSEPTA	APS NiAl-AA	APS NiAlMo-AA	1 M KOH	65	0.5	112 (350 μV h^{-1})	137
Sustainion Grade T	NiFe2O4	Raney nickel	1 M KOH	60	1	12180 (0.7 μV h^{-1})	21
Sustainion X37-50	NiFe2O4	Raney nickel	1 M KOH	60	1	10100 (0.7 μV h^{-1})	21
Fumatech FAS-50	NiFe2O4	Raney nickel	1 M KOH	60	1	140 (655 μV h^{-1})	21
AF1-HNN8-25X	IrO_2	Pt/C	1 M KOH	50	0.5	17 (2390 μV h^{-1})	15
ATM-PP	IrO_2	Pt/C		50	0.2	2100	82
PSU-PVP	NiFe	NiMo	20 wt% KOH	80	0.5	700	76
PTP-85	IrO_2	Pt/C	1 M NaOH	55	0.4	120 (1.61 mV h-1)	129
Sustainion X37–50	$NiFe_2O_4$	NiFeCo	1 M KOH	50	0.2	1000	17
X37-50 Grade T	Ni-doped FeOOH	Pt/C	1 M KOH + seawater	50	0.5	15 (1.72 V)	138
HTMA-DAPP	NiFe	PtRu/C	Water	60	0.2	170 (2.1 V)	40
PFOTFPh-TMA C8	IrO_2	Pt/C	Water	80	0.2	130	5
PFOTFPh-TMA C10	IrO_2	Pt/C	1 M KOH	80	0.2	150	5
Sustainion X37-50 Grade T	CCO-11	Pt/C	1 M KOH	45	0.4	100	23

References:

[1] X. Hu, Y. Huang, L. Liu, Q. Ju, X. Zhou, X. Qiao, Z. Zheng, N. Li, Piperidinium functionalized aryl ether-free polyaromatics as anion exchange membrane for water electrolysers: Performance and durability, J. Memb. Sci. 621 (2021) 118964. https://doi.org/10.1016/j.memsci.2020.118964.

[2] D. Henkensmeier, M. Najibah, C. Harms, J. Zitka, J. Hnat, K. Bouzek, Overview: State-of-the Art Commercial Membranes for Anion Exchange Membrane Water Electrolysis, J. Electrochem. Energy Convers. Storage. 18 (2021). https://doi.org/10.1115/1.4047963.

[3] A. Carbone, S.C. Zignani, I. Gatto, S. Trocino, A.S. Arico, Assessment of the FAA3-50 polymer electrolyte in combination with a NiMn2O4 anode catalyst for anion exchange membrane water electrolysis, Int. J. Hydrogen Energy. 45 (2020) 9285–9292. https://doi.org/10.1016/j.ijhydene.2020.01.150.

[4] S. Vengatesan, S. Santhi, S. Jeevanantham, G. Sozhan, Quaternized poly (styrene-co-vinylbenzyl chloride) anion exchange membranes for alkaline water electrolysers, J. Power Sources. 284 (2015) 361–368. https://doi.org/10.1016/j.jpowsour.2015.02.118.

[5] R. Soni, S. Miyanishi, H. Kuroki, T. Yamaguchi, Pure Water Solid Alkaline Water Electrolyzer Using Fully Aromatic and High-Molecular-Weight Poly(fluorene- alt-tetrafluorophenylene)-trimethyl Ammonium Anion Exchange Membranes and Ionomers, ACS Appl. Energy Mater. 4 (2021) 1053–1058. https://doi.org/10.1021/acsaem.0c01938.

[6] S. Miyanishi, T. Yamaguchi, Highly conductive mechanically robust high M-w polyfluorene anion exchange membrane for alkaline fuel cell and water electrolysis application, Polym. Chem. 11 (2020) 3812–3820. https://doi.org/10.1039/d0py00334d.

[7] G. Huang, M. Mandal, N.U. Hassan, K. Groenhout, A. Dobbs, W.E. Mustain, P.A. Kohl, Ionomer Optimization for Water Uptake and Swelling in Anion Exchange Membrane Electrolyzer: Oxygen Evolution Electrode, J. Electrochem. Soc. 167 (2020) 164514. https://doi.org/10.1149/1945-7111/abcde3.

[8] J.W. Xiao, A.M. Oliveira, L. Wang, Y. Zhao, T. Wang, J.H. Wang, B.P. Setzler, Y.S. Yan, Water-Fed Hydroxide Exchange Membrane Electrolyzer Enabled by a Fluoride-Incorporated Nickel-Iron Oxyhydroxide Oxygen Evolution Electrode, ACS Catal. 11 (2021) 264–270. https://doi.org/10.1021/acscatal.0c04200.

[9] J. Wang, Y. Zhao, B.P. Setzler, S. Rojas-Carbonell, C. Ben Yehuda, A. Amel, M. Page, L. Wang, K. Hu, L. Shi, S. Gottesfeld, B. Xu, Y. Yan, Poly(aryl piperidinium) membranes and ionomers for hydroxide exchange membrane fuel cells, Nat. Energy. 4 (2019) 392–398. https://doi.org/10.1038/s41560-019-0372-8.

[10] C.C. Pavel, F. Cecconi, C. Emiliani, S. Santiccioli, A. Scaffidi, S. Catanorchi, M. Comotti, Highly Efficient Platinum Group Metal Free Based Membrane-Electrode Assembly for Anion Exchange Membrane Water Electrolysis, Angew. CHEMIE-INTERNATIONAL Ed. 53 (2014) 1378–1381. https://doi.org/10.1002/anie.201308099.

[11] W.T. Lu, Z.Z. Yang, H. Huang, F. Wei, W.H. Li, Y.H. Yu, Y.G. Gao, Y.H. Zhou, G. Zhang, Piperidinium-Functionalized Poly(Vinylbenzyl Chloride) Cross-linked by Polybenzimidazole as an Anion Exchange Membrane with a Continuous Ionic Transport Pathway, Ind. Eng. Chem. Res. 59 (2020) 21077–21087. https://doi.org/10.1021/acs.iecr.0c04548.

[12] M. Bhushan, M. Mani, A.K. Singh, A.B. Panda, V.K. Shahi, Self-standing polyaniline membrane containing quaternary ammonium groups loaded with hollow spherical NiCo2O4electrocatalyst for alkaline water electrolyser, J. Mater. Chem. A. 8 (2020) 17089–17097. https://doi.org/10.1039/d0ta05373b.

[13] A. Marinkas, I. Strużyńska-Piron, Y. Lee, A. Lim, H.S. Park, J.H. Jang, H.J. Kim, J. Kim, A. Maljusch, O. Conradi, D. Henkensmeier, Anion-conductive membranes based on 2-mesityl-benzimidazolium functionalised poly(2,6-dimethyl-1,4-phenylene oxide) and their use in alkaline water electrolysis, Polymer (Guildf). 145 (2018) 242–251. https://doi.org/10.1016/j.polymer.2018.05.008.

[14] H. Ito, N. Kawaguchi, S. Someya, T. Munakata, N. Miyazaki, M. Ishida, A. Nakano, Experimental investigation of electrolytic solution for anion exchange membrane water electrolysis, Int. J. Hydrogen Energy. 43 (2018) 17030–17039. https://doi.org/10.1016/j.ijhydene.2018.07.143.

[15] P. Fortin, T. Khoza, X.Z. Cao, S.Y. Martinsen, A.O. Barnett, S. Holdcroft, High-performance alkaline water electrolysis using Aemion (TM) anion exchange membranes, J. Power Sources. 451 (2020) 227814. https://doi.org/10.1016/j.jpowsour.2020.227814.

[16] J.J. Kaczur, H.Z. Yang, Z.C. Liu, S.A. Sajjad, R.I. Masel, Carbon Dioxide and Water Electrolysis Using New Alkaline Stable Anion Membranes, Front. Chem. 6 (2018). https://doi.org/10.3389/fchem.2018.00263.

[17] A.K. Niaz, A. Akhtar, J.Y. Park, H.T. Lim, Effects of the operation mode on the degradation behavior of anion exchange membrane water electrolyzers, J. Power Sources. 481 (2021) 229093. https://doi.org/10.1016/j.jpowsour.2020.229093.

[18] Z. Liu, S.D. Sajjad, Y. Gao, J. Kaczur, R. Masel, An Alkaline Water Electrolyzer with Sustainion^{TM} Membranes: 1 A/cm^2 at 1.9V with Base Metal Catalysts, ECS Trans. 77 (2017) 71–73. https://doi.org/10.1149/07709.0071ecst.

[19] Z. Liu, S.D. Sajjad, Y. Gao, H. Yang, J.J. Kaczur, R.I. Masel, The effect of membrane on an alkaline water electrolyzer, Int. J. Hydrogen Energy. 42 (2017) 29661–29665. https://doi.org/10.1016/j.ijhydene.2017.10.050.

[20] P.Z. Chen, X.L. Hu, High-Efficiency Anion Exchange Membrane Water Electrolysis Employing Non-Noble Metal Catalysts, Adv. Energy Mater. 10 (2020) 2002285. https://doi.org/10.1002/aenm.202002285.

[21] B. Motealleh, Z. Liu, R.I. Masel, J.P. Sculley, Z. Richard Ni, L. Meroueh, Next-generation anion exchange membrane water electrolyzers operating for commercially relevant lifetimes, Int. J. Hydrogen Energy. 46 (2021) 3379–3386. https://doi.org/10.1016/j.ijhydene.2020.10.244.

[22] Y.S. Park, J. Yang, J. Lee, M.J. Jang, J. Jeong, W.S. Choi, Y. Kim, Y. Yin, M.H. Seo, Z. Chen, S.M. Choi, Superior performance of anion exchange membrane water electrolyzer: Ensemble of producing oxygen vacancies and controlling mass transfer resistance, Appl. Catal. B Environ. 278 (2020) 119276. https://doi.org/10.1016/j.apcatb.2020.119276.

[23] M.J. Jang, J. Yang, J. Lee, Y.S. Park, J. Jeong, S.M. Park, J.Y. Jeong, Y. Yin, M.H. Seo, S.M. Choi, K.H. Lee, Superior performance and stability of anion exchange membrane water electrolysis: PH-controlled copper cobalt oxide nanoparticles for the oxygen evolution reaction, J. Mater. Chem. A. 8 (2020) 4290–4299. https://doi.org/10.1039/c9ta13137j.

[24] L. Wang, X. Peng, W.E. Mustain, J.R. Varcoe, Radiation-grafted anion-exchange membranes: The switch from low- to high-density polyethylene leads to remarkably enhanced fuel cell performance, Energy Environ. Sci. 12 (2019) 1575–1579. https://doi.org/10.1039/c9ee00331b.

[25] S. Adhikari, M.K. Pagels, J.Y. Jeon, C. Bae, Ionomers for electrochemical energy conversion & storage technologies, Polymer. 211 (2020) 123080. https://doi.org/10.1016/j.polymer.2020.123080.

[26] L. Wang, M. Bellini, H.A. Miller, J.R. Varcoe, A high conductivity ultrathin anion-exchange membrane with 500+ h alkali stability for use in alkaline membrane fuel cells that can achieve 2 W cm-2 at 80 °c, J. Mater. Chem. A. 6 (2018) 15404–15412. https://doi.org/10.1039/c8ta04783a.

[27] A. Zhegur-Khais, F. Kubannek, U. Krewer, D.R. Dekel, Measuring the true hydroxide conductivity of anion exchange membranes, J. Memb. Sci. 612 (2020) 118461. https://doi.org/10.1016/j.memsci.2020.118461.

[28] J.C. Douglin, J.R. Varcoe, D.R. Dekel, A high-temperature anion-exchange membrane fuel

cell, J. Power Sources Adv. 5 (2020) 100023. https://doi.org/10.1016/j.powera.2020.100023.

29 J.C. Douglin, R.K. Singh, S. Haj, S. Li, J. Biemolt, N. Yan, J.R. Varcoe, G. Rothenberg, D. Dekel, A High-Temperature Anion-Exchange Membrane Fuel Cell with a Critical Raw Material-free Cathode, Chem. Eng. J. Adv. 8 (2021) 100153. https://doi.org/10.1016/j.ceja.2021.100153.

30 J. Ponce-González, I. Ouachan, J.R. Varcoe, D.K. Whelligan, Radiation-induced grafting of a butyl-spacer styrenic monomer onto ETFE: The synthesis of the most alkali stable radiation-grafted anion-exchange membrane to date, J. Mater. Chem. A. 6 (2018) 823–827. https://doi.org/10.1039/c7ta10222d.

31 C. Xiao Lin, X. Qin Wang, E. Ning Hu, Q. Yang, Q. Gen Zhang, A. Mei Zhu, Q. Lin Liu, Quaternized triblock polymer anion exchange membranes with enhanced alkaline stability, J. Memb. Sci. 541 (2017) 358–366. https://doi.org/10.1016/j.memsci.2017.07.032.

32 J.Y. Jeon, S. Park, J. Han, S. Maurya, A.D. Mohanty, D. Tian, N. Saikia, M.A. Hickner, C.Y. Ryu, M.E. Tuckerman, S.J. Paddison, Y.S. Kim, C. Bae, Synthesis of Aromatic Anion Exchange Membranes by Friedel-Crafts Bromoalkylation and Cross-Linking of Polystyrene Block Copolymers, Macromolecules. 52 (2019) 2139–2147. https://doi.org/10.1021/acs.macromol.8b02355.

33 T.J. Clark, N.J. Robertson, H.A. Kostalik IV, E.B. Lobkovsky, P.F. Mutolo, H.D. Abruña, G.W. Coates, A ring-opening metathesis polymerization route to alkaline anion exchange membranes: Development of hydroxide-conducting thin films from an ammonium-functionalized monomer, J. Am. Chem. Soc. 131 (2009) 12888–12889. https://doi.org/10.1021/ja905242r.

34 S.C. Price, X. Ren, A.M. Savage, F.L. Beyer, Synthesis and characterization of anion-exchange membranes based on hydrogenated poly(norbornene), Polym. Chem. 8 (2017) 5708–5717. https://doi.org/10.1039/c7py01084b.

35 W. Chen, M. Mandal, G. Huang, X. Wu, G. He, P.A. Kohl, Highly Conducting Anion-Exchange Membranes Based on Cross-Linked Poly(norbornene): Ring Opening Metathesis Polymerization, ACS Appl. Energy Mater. 2 (2019) 2458–2468. https://doi.org/10.1021/acsaem.8b02052.

36 W. You, E. Padgett, S.N. MacMillan, D.A. Muller, G.W. Coates, Highly conductive and chemically stable alkaline anion exchange membranes via ROMP of trans-cyclooctene derivatives, Proc. Natl. Acad. Sci. U. S. A. 116 (2019) 9729–9734. https://doi.org/10.1073/pnas.1900988116.

37 M. Mandal, G. Huang, P.A. Kohl, Anionic multiblock copolymer membrane based on vinyl

addition polymerization of norbornenes: Applications in anion-exchange membrane fuel cells, J. Memb. Sci. 570–571 (2019) 394–402. https://doi.org/10.1016/j.memsci.2018.10.041.

[38] L. Zhu, X. Peng, S.L. Shang, M.T. Kwasny, T.J. Zimudzi, X. Yu, N. Saikia, J. Pan, Z.K. Liu, G.N. Tew, W.E. Mustain, M. Yandrasits, M.A. Hickner, High Performance Anion Exchange Membrane Fuel Cells Enabled by Fluoropoly(olefin) Membranes, Adv. Funct. Mater. 29 (2019). https://doi.org/10.1002/adfm.201902059.

[39] M.R. Hibbs, Alkaline stability of poly(phenylene)-based anion exchange membranes with various cations, J. Polym. Sci. Part B Polym. Phys. 51 (2013) 1736–1742. https://doi.org/10.1002/polb.23149.

[40] D. Li, E.J. Park, W. Zhu, Q. Shi, Y. Zhou, H. Tian, Y. Lin, A. Serov, B. Zulevi, E.D. Baca, C. Fujimoto, H.T. Chung, Y.S. Kim, Highly quaternized polystyrene ionomers for high performance anion exchange membrane water electrolysers, Nat. Energy. 5 (2020) 378–385. https://doi.org/10.1038/s41560-020-0577-x.

[41] H. Ono, J. Miyake, S. Shimada, M. Uchida, K. Miyatake, Anion exchange membranes composed of perfluoroalkylene chains and ammonium-functionalized oligophenylenes, J. Mater. Chem. A. 3 (2015) 21779–21788. https://doi.org/10.1039/c5ta06454f.

[42] R. Akiyama, N. Yokota, K. Miyatake, Chemically Stable, Highly Anion Conductive Polymers Composed of Quinquephenylene and Pendant Ammonium Groups, Macromolecules. 52 (2019) 2131–2138. https://doi.org/10.1021/acs.macromol.8b02199.

[43] J. Fan, S. Willdorf-Cohen, E.M. Schibli, Z. Paula, W. Li, T.J.G. Skalski, A.T. Sergeenko, A. Hohenadel, B.J. Frisken, E. Magliocca, W.E. Mustain, C.E. Diesendruck, D.R. Dekel, S. Holdcroft, Poly(bis-arylimidazoliums) possessing high hydroxide ion exchange capacity and high alkaline stability, Nat. Commun. 10 (2019) 2306. https://doi.org/10.1038/s41467-019-10292-z.

[44] S. Noh, J.Y. Jeon, S. Adhikari, Y.S. Kim, C. Bae, Molecular Engineering of Hydroxide Conducting Polymers for Anion Exchange Membranes in Electrochemical Energy Conversion Technology, Acc. Chem. Res. 52 (2019) 2745–2755. https://doi.org/10.1021/acs.accounts.9b00355.

[45] W.-H. Lee, E.J. Park, J. Han, D.W. Shin, Y.S. Kim, C. Bae, Poly(terphenylene) Anion Exchange Membranes: The Effect of Backbone Structure on Morphology and Membrane Property, ACS Macro Lett. 6 (2017) 566–570. https://doi.org/10.1021/acsmacrolett.7b00148.

[46] J.S. Olsson, T.H. Pham, P. Jannasch, Poly(arylene piperidinium) Hydroxide Ion Exchange

Membranes: Synthesis, Alkaline Stability, and Conductivity, Adv. Funct. Mater. 28 (2018) 1702758. https://doi.org/10.1002/adfm.201702758.

47 H. Li, M.R. Kraglund, A.K. Reumert, X. Ren, D. Aili, J. Yang, Poly(vinyl benzyl methylpyrrolidinium) hydroxide derived anion exchange membranes for water electrolysis, J. Mater. Chem. A. 7 (2019) 17914–17922. https://doi.org/10.1039/c9ta04868e.

48 J. Parrondo, C.G. Arges, M. Niedzwiecki, E.B. Anderson, K.E. Ayers, V. Ramani, Degradation of anion exchange membranes used for hydrogen production by ultrapure water electrolysis., RSC Adv. 4 (2014) 9875–9879. https://doi.org/10.1039/c3ra46630b.

49 C.G. Arges, J. Parrondo, G. Johnson, A. Nadhan, V. Ramani, Assessing the influence of different cation chemistries on ionic conductivity and alkaline stability of anion exchange membranes, J. Mater. Chem. 22 (2012) 3733. https://doi.org/10.1039/c2jm14898f.

50 J. Pan, Y. Li, L. Zhuang, J. Lu, Self-crosslinked alkaline polymer electrolyte exceptionally stable at 90 °C, Chem. Commun. 46 (2010) 8597–8599. https://doi.org/10.1039/c0cc03618h.

51 J. Pan, C. Chen, L. Zhuang, J. Lu, Designing Advanced Alkaline Polymer Electrolytes for Fuel Cell Applications, Acc. Chem. Res. 45 (2012) 473–481.

52 L. Xiao, S. Zhang, J. Pan, C. Yang, M. He, L. Zhuang, J. Lu, First implementation of alkaline polymer electrolyte water electrolysis working only with pure water, Energy Environ. Sci. 5 (2012) 7869–7871. https://doi.org/10.1039/c2ee22146b.

53 A. Lim, H. juhn Kim, D. Henkensmeier, S. Jong Yoo, J. Young Kim, S. Young Lee, Y.E. Sung, J.H. Jang, H.S. Park, A study on electrode fabrication and operation variables affecting the performance of anion exchange membrane water electrolysis, J. Ind. Eng. Chem. 76 (2019) 410–418. https://doi.org/10.1016/j.jiec.2019.04.007.

54 J. Hnát, M. Plevová, J. Žitka, M. Paidar, K. Bouzek, Anion-selective materials with 1,4-diazabicyclo[2.2.2]octane functional groups for advanced alkaline water electrolysis, Electrochim. Acta. 248 (2017) 547–555. https://doi.org/10.1016/j.electacta.2017.07.165.

55 N. Lee, D.T. Duong, D. Kim, Cyclic ammonium grafted poly (arylene ether ketone) hydroxide ion exchange membranes for alkaline water electrolysis with high chemical stability and cell efficiency, Electrochim. Acta. 271 (2018) 150–157. https://doi.org/10.1016/j.electacta.2018.03.117.

56 X. Wu, K. Scott, A Li-doped Co3O4 oxygen evolution catalyst for non-precious metal alkaline anion exchange membrane water electrolysers, Int. J. Hydrogen Energy. 38 (2013) 3123–3129. https://doi.org/10.1016/j.ijhydene.2012.12.087.

[57] X. Wu, K. Scott, A polymethacrylate-based quaternary ammonium OH - ionomer binder for non-precious metal alkaline anion exchange membrane water electrolysers, J. Power Sources. 214 (2012) 124–129. https://doi.org/10.1016/j.jpowsour.2012.03.069.

[58] M. Faraj, M. Boccia, H. Miller, F. Martini, S. Borsacchi, M. Geppi, A. Pucci, New LDPE based anion-exchange membranes for alkaline solid polymeric electrolyte water electrolysis, Int. J. Hydrogen Energy. 37 (2012) 14992–15002. https://doi.org/10.1016/j.ijhydene.2012.08.012.

[59] X.D. Su, L. Gao, L. Hu, N.A. Qaisrani, X.M. Yan, W.J. Zhang, X.B. Jiang, X.H. Ruan, G.H. He, Novel piperidinium functionalized anionic membrane for alkaline polymer electrolysis with excellent electrochemical properties, J. Memb. Sci. 581 (2019) 283–292. https://doi.org/10.1016/j.memsci.2019.03.072.

[60] N. Chen, C. Lu, Y. Li, C. Long, H. Zhu, Robust poly(aryl piperidinium)/N-spirocyclic poly(2,6-dimethyl-1,4-phenyl) for hydroxide-exchange membranes, J. Memb. Sci. 572 (2019) 246–254. https://doi.org/10.1016/j.memsci.2018.10.067.

[61] I. Vincent, A. Kruger, D. Bessarabov, Hydrogen Production by water Electrolysis with an Ultrathin Anion-exchange membrane (AEM), Int. J. Electrochem. Sci. 13 (2018) 11347–11358. https://doi.org/10.20964/2018.12.84.

[62] I. Vincent, A. Kruger, D. Bessarabov, Development of efficient membrane electrode assembly for low cost hydrogen production by anion exchange membrane electrolysis, Int. J. Hydrogen Energy. 42 (2017) 10752–10761. https://doi.org/10.1016/j.ijhydene.2017.03.069.

[63] G. Gupta, K. Scott, M. Mamlouk, Performance of polyethylene based radiation grafted anion exchange membrane with polystyrene-b-poly (ethylene/butylene)-b-polystyrene based ionomer using NiCo2O4 catalyst for water electrolysis, J. Power Sources. 375 (2018) 387–396. https://doi.org/10.1016/j.jpowsour.2017.07.026.

[64] X. Wu, K. Scott, F. Xie, N. Alford, A reversible water electrolyser with porous PTFE based OH- conductive membrane as energy storage cells, J. Power Sources. 246 (2014) 225–231. https://doi.org/10.1016/j.jpowsour.2013.07.081.

[65] Y.C. Cao, X. Wu, K. Scott, A quaternary ammonium grafted poly vinyl benzyl chloride membrane for alkaline anion exchange membrane water electrolysers with no-noble-metal catalysts, Int. J. Hydrogen Energy. 37 (2012) 9524–9528. https://doi.org/10.1016/j.ijhydene.2012.03.116.

[66] Y.C. Cao, X. Wang, M. Mamlouk, K. Scott, Preparation of alkaline anion exchange polymer membrane from methylated melamine grafted poly(vinylbenzyl chloride) and its fuel cell

performance, J. Mater. Chem. 21 (2011) 12910–12916. https://doi.org/10.1039/c1jm12068a.

67 E.J. Park, C.B. Capuano, K.E. Ayers, C. Bae, Chemically durable polymer electrolytes for solid-state alkaline water electrolysis, J. Power Sources. 375 (2018) 367–372. https://doi.org/10.1016/j.jpowsour.2017.07.090.

68 M.S. Cha, J.E. Park, S. Kim, S.-H. Han, S.-H. Shin, S.H. Yang, T.-H. Kim, D.M. Yu, S. So, Y.T. Hong, S.J. Yoon, S.-G. Oh, S.Y. Kang, O.-H. Kim, H.S. Park, B. Bae, Y.-E. Sung, Y.-H. Cho, J.Y. Lee, Poly(carbazole)-based anion-conducting materials with high performance and durability for energy conversion devices, Energy Environ. Sci. 13 (2020) 3633–3645. https://doi.org/10.1039/d0ee01842b.

69 H. Li, N. Yu, F. Gellrich, A.K. Reumert, M.R. Kraglund, J. Dong, D. Aili, J. Yang, Diamine crosslinked anion exchange membranes based on poly(vinyl benzyl methylpyrrolidinium) for alkaline water electrolysis, J. Memb. Sci. 633 (2021). https://doi.org/10.1016/j.memsci.2021.119418.

70 D. Aili, A.G. Wright, M.R. Kraglund, K. Jankova, S. Holdcroft, J.O. Jensen, Towards a stable ion-solvating polymer electrolyte for advanced alkaline water electrolysis, J. Mater. Chem. A. 5 (2017) 5055–5066. https://doi.org/10.1039/c6ta10680c.

71 C.G. Arges, L. Wang, J. Parrondo, V. Ramani, Best Practices for Investigating Anion Exchange Membrane Suitability for Alkaline Electrochemical Devices: Case Study Using Quaternary Ammonium Poly(2,6-dimethyl 1,4-phenylene)oxide Anion Exchange Membranes, J. Electrochem. Soc. 160 (2013) F1258–F1274. https://doi.org/10.1149/2.049311jes.

72 J. Parrondo, V. Ramani, Stability of Poly(2,6-dimethyl 1,4-phenylene)Oxide-Based Anion Exchange Membrane Separator and Solubilized Electrode Binder in Solid-State Alkaline Water Electrolyzers, J. Electrochem. Soc. 161 (2014) F1015–F1020. https://doi.org/10.1149/2.0601410jes.

73 H.J. Park, S.Y. Lee, T.K. Lee, H.-J. Kim, Y.M. Lee, N3-butyl imidazolium-based anion exchange membranes blended with Poly(vinyl alcohol) for alkaline water electrolysis, J. Memb. Sci. 611 (2020) 118355. https://doi.org/10.1016/j.memsci.2020.118355.

74 X.M. Yan, X. Yang, X.D. Su, L. Gao, J. Zhao, L. Hu, M.T. Di, T.T. Li, X.H. Ruan, G.H. He, Twisted ether-free polymer based alkaline membrane for high-performance water electrolysis, J. Power Sources. 480 (2020) 228805. https://doi.org/10.1016/j.jpowsour.2020.228805.

75 X.M. Chu, Y. Shi, L. Liu, Y.D. Huang, N.W. Li, Piperidinium-functionalized anion exchange membranes and their application in alkaline fuel cells and water electrolysis, J.

Mater. Chem. A. 7 (2019) 7717–7727. https://doi.org/10.1039/c9ta01167f.

76 D. Aili, M.R. Kraglund, J. Tavacoli, C. Chatzichristodoulou, J.O. Jensen, Polysulfone-polyvinylpyrrolidone blend membranes as electrolytes in alkaline water electrolysis, J. Memb. Sci. 598 (2020) 117674. https://doi.org/10.1016/j.memsci.2019.117674.

77 R.E. Coppola, D. Herranz, R. Escudero-Cid, N. Ming, N.B. D'Accorso, P. Ocon, G.C. Abuin, Polybenzimidazole-crosslinked-poly(vinyl benzyl chloride) as anion exchange membrane for alkaline electrolyzers, Renew. Energy. 157 (2020) 71–82. https://doi.org/10.1016/j.renene.2020.04.140.

78 R.E. Coppola, F.N. Molinari, N.B. D'Accorso, G.C. Abuin, Polyvinyl alcohol nanofibers reinforced with polybenzimidazole: Facile preparation and properties of an anion exchange membrane, Polym. Adv. Technol. 32 (2021) 3505–3514. https://doi.org/10.1002/pat.5361.

79 H. Ono, T. Kimura, A. Takano, K. Asazawa, J. Miyake, J. Inukai, K. Miyatake, Robust anion conductive polymers containing perfluoroalkylene and pendant ammonium groups for high performance fuel cells, J. Mater. Chem. A. 5 (2017) 24804–24812. https://doi.org/10.1039/c7ta09409d.

80 S. Maurya, C.H. Fujimoto, M.R. Hibbs, C. Narvaez Villarrubia, Y.S. Kim, Toward Improved Alkaline Membrane Fuel Cell Performance Using Quaternized Aryl-Ether Free Polyaromatics, Chem. Mater. 30 (2018) 2188–2192. https://doi.org/10.1021/acs.chemmater.8b00358.

81 S. Miyanishi, T. Yamaguchi, Highly durable spirobifluorene-based aromatic anion conducting polymer for a solid ionomer of alkaline fuel cells and water electrolysis cells, J. Mater. Chem. A. 7 (2019) 2219–2224. https://doi.org/10.1039/c8ta08400a.

82 Y.K. Choe, C. Fujimoto, K.S. Lee, L.T. Dalton, K. Ayers, N.J. Henson, Y.S. Kim, Alkaline stability of benzyl trimethyl ammonium functionalized polyaromatics: A computational and experimental study, Chem. Mater. 26 (2014) 5675–5682. https://doi.org/10.1021/cm502422h.

83 D. Aili, M.K. Hansen, R.F. Renzaho, Q.F. Li, E. Christensen, J.O. Jensen, N.J. Bjerrum, Heterogeneous anion conducting membranes based on linear and crosslinked KOH doped polybenzimidazole for alkaline water electrolysis, J. Memb. Sci. 447 (2013) 424–432. https://doi.org/10.1016/j.memsci.2013.07.054.

84 L.A. Diaz, J. Hnát, N. Heredia, M.M. Bruno, F.A. Viva, M. Paidar, H.R. Corti, K. Bouzek, G.C. Abuin, Alkali doped poly (2,5-benzimidazole) membrane for alkaline water electrolysis: Characterization and performance, J. Power Sources. 312 (2016) 128–136. https://doi.org/10.1016/j.jpowsour.2016.02.032.

[85] Y. Yang, T. Jiang, L. Li, S. Zhou, H. Fang, X. Li, H. Wei, Y. Ding, Chemo-stable poly(quinquephenylene-co-diphenylene piperidinium) ionomers for anion exchange membrane fuel cells, J. Power Sources. 506 (2021) 230184. https://doi.org/10.1016/j.jpowsour.2021.230184.

[86] L. Li, J. Wang, M. Hussain, L. Ma, N.A. Qaisrani, S. Ma, L. Bai, X. Yan, X. Deng, G. He, F. Zhang, Side-chain manipulation of poly (phenylene oxide) based anion exchange membrane: Alkoxyl extender integrated with flexible spacer, J. Memb. Sci. 624 (2021) 119088. https://doi.org/10.1016/j.memsci.2021.119088.

[87] N. Chen, H.H. Wang, S.P. Kim, H.M. Kim, W.H. Lee, C. Hu, J.Y. Bae, E.S. Sim, Y.C. Chung, J.H. Jang, S.J. Yoo, Y. Zhuang, Y.M. Lee, Poly(fluorenyl aryl piperidinium) membranes and ionomers for anion exchange membrane fuel cells, Nat. Commun. 12 (2021) 2367. https://doi.org/10.1038/s41467-021-22612-3.

[88] C. Long, Z. Wang, H. Zhu, High chemical stability anion exchange membrane based on poly(aryl piperidinium): Effect of monomer configuration on membrane properties, Int. J. Hydrogen Energy. 46 (2021) 18524–18533. https://doi.org/10.1016/j.ijhydene.2021.02.209.

[89] J. Zhang, K. Zhang, X. Liang, W. Yu, X. Ge, M.A. Shehzad, Z. Ge, Z. Yang, L. Wu, T. Xu, Self-aggregating cationic-chains enable alkaline stable ion-conducting channels for anion-exchange membrane fuel cells, J. Mater. Chem. A. 9 (2021) 327–337. https://doi.org/10.1039/d0ta11011f.

[90] X. Qiao, X. Wang, S. Liu, Y. Shen, N. Li, The alkaline stability and fuel cell performance of poly(N-spirocyclic quaternary ammonium) ionenes as anion exchange membrane, J. Memb. Sci. 630 (2021) 119325. https://doi.org/10.1016/j.memsci.2021.119325.

[91] K. Firouz Tadavani, A. Abdolmaleki, M.R. Molavian, M. Zhiani, New Strategy Based on Click Reaction for Preparation of Cross-Linked Poly(Benzimidazolium-Imide) as an Anion-Exchange Membrane with Improved Alkaline Stability, Ind. Eng. Chem. Res. 60 (2021) 7097–7110. https://doi.org/10.1021/acs.iecr.1c00071.

[92] Y. Yuan, T. Zhang, Z. Wang, Preparation of an Anion Exchange Membrane by Pyridine-Functionalized Polyether Ether Ketone to Improve Alkali Resistance Stability for an Alkali Fuel Cell, Energy and Fuels. 35 (2021) 3360–3367. https://doi.org/10.1021/acs.energyfuels.0c03428.

[93] Y. Lu, L. Liu, Y. Pu, Y. Liu, N. Li, Z. Hu, S. Chen, Towards performance improved anion exchange membrane: Cross-linking with multi-cations oligomer modified graphene oxide, Int. J. Hydrogen Energy. 46 (2021) 23855–23867. https://doi.org/10.1016/j.ijhydene.2021.04.167.

[94] D. Liu, L. Lin, Y. Xie, J. Pang, Z. Jiang, Anion exchange membrane based on poly(arylene ether ketone) containing long alkyl densely quaternized carbazole derivative pendant, J. Memb. Sci. 623 (2021) 119079. https://doi.org/10.1016/j.memsci.2021.119079.

[95] F. Wang, Y. Li, C.H. Li, H. Zhu, Preparation and study of spirocyclic cationic side chain functionalized polybiphenyl piperidine anion exchange membrane, J. Memb. Sci. 620 (2021) 118919. https://doi.org/10.1016/j.memsci.2020.118919.

[96] Y. Xiao, M. Zhang, D. Dong, Z. Yang, Y. Cao, K. Wang, M. Fan, Preparation of Branch Polyethyleneimine (BPEI) Crosslinked Anion Exchange Membrane Based on Poly(styrene-b-(ethylene-co-butylene)-b-styrene) (SEBS), Macromol. Mater. Eng. 306 (2021) 2000693. https://doi.org/10.1002/mame.202000693.

[97] M. Kumari, J.C. Douglin, D.R. Dekel, Crosslinked quaternary phosphonium-functionalized poly(ether ether ketone) polymer-based anion-exchange membranes, J. Memb. Sci. 626 (2021) 119167. https://doi.org/10.1016/j.memsci.2021.119167.

[98] M.I. Khan, X. Li, J. Fernandez-Garcia, M.H. Lashari, A. Ur Rehman, N. Elboughdiri, L. Kolsi, D. Ghernaout, Effect of Different Quaternary Ammonium Groups on the Hydroxide Conductivity and Stability of Anion Exchange Membranes, ACS Omega. 6 (2021) 7994–8001. https://doi.org/10.1021/acsomega.0c05134.

[99] L. Bai, L. Ma, L. Li, A. Zhang, X. Yan, F. Zhang, G. He, Branched, Side-Chain Grafted Polyarylpiperidine Anion Exchange Membranes for Fuel Cell Application, ACS Appl. Energy Mater. 4 (2021) 6957–6967. https://doi.org/10.1021/acsaem.1c01037.

[100] X. Wang, C. Lin, Y. Gao, R.G.H. Lammertink, Anion exchange membranes with twisted poly(terphenylene) backbone: Effect of the N-cyclic cations, J. Memb. Sci. 635 (2021) 119525. https://doi.org/10.1016/j.memsci.2021.119525.

[101] N.C. Buggy, Y.F. Du, M.C. Kuo, K.A. Ahrens, J.S. Wilkinson, S. Seifert, E.B. Coughlin, A.M. Herring, A Polyethylene-Based Triblock Copolymer Anion Exchange Membrane with High Conductivity and Practical Mechanical Properties, ACS Appl. Polym. Mater. 2 (2020) 1294–1303. https://doi.org/10.1021/acsapm.9b01182.

[102] H.J. Park, X. Chu, S.P. Kim, D. Choi, J.W. Jung, J. Woo, S.Y. Baek, S.J. Yoo, Y.C. Chung, J.G. Seong, S.Y. Lee, N. Li, Y.M. Lee, Effect of N-cyclic cationic groups in poly(phenylene oxide)-based catalyst ionomer membranes for anion exchange membrane fuel cells, J. Memb. Sci. 608 (2020) 118183. https://doi.org/10.1016/j.memsci.2020.118183.

[103] Y. Wang, D. Zhang, X. Liang, M.A. Shehzad, X. Xiao, Y. Zhu, X. Ge, J. Zhang, Z. Ge, L. Wu, T. Xu, Improving fuel cell performance of an anion exchange membrane by terminal

pending bis-cations on a flexible side chain, J. Memb. Sci. 595 (2020) 117483. https://doi.org/10.1016/j.memsci.2019.117483.

[104] C. Cheng, X. He, S. Huang, F. Zhang, Y. Guo, Y. Wen, B. Wu, D. Chen, Novel self-cross-linked multi-imidazolium cations long flexible side chains triblock copolymer anion exchange membrane based on ROMP-type polybenzonorbornadiene, Int. J. Hydrogen Energy. 45 (2020) 19676–19690. https://doi.org/10.1016/j.ijhydene.2020.04.276.

[105] Z. Liu, X. Li, K. Shen, P. Feng, Y. Zhang, X. Xu, W. Hu, Z. Jiang, B. Liu, M.D. Guiver, Naphthalene-based poly(arylene ether ketone) anion exchange membranes, J. Mater. Chem. A. 1 (2013) 6481–6488. https://doi.org/10.1039/c3ta10355b.

[106] A. Ouadah, H. Xu, T. Luo, S. Gao, Z. Zhang, Z. Li, C. Zhu, Synthesis of novel copolymers based on: P -methylstyrene, N, N -butylvinylimidazolium and polybenzimidazole as highly conductive anion exchange membranes for fuel cell application, RSC Adv. 7 (2017) 47806–47817. https://doi.org/10.1039/c7ra06394f.

[107] J.Y. Chu, K.H. Lee, A.R. Kim, D.J. Yoo, Improved electrochemical performance of composite anion exchange membranes for fuel cells through cross linking of the polymer chain with functionalized graphene oxide, J. Memb. Sci. 611 (2020) 118385. https://doi.org/10.1016/j.memsci.2020.118385.

[108] T.H. Pham, A. Allushi, J.S. Olsson, P. Jannasch, Rational molecular design of anion exchange membranes functionalized with alicyclic quaternary ammonium cations, Polym. Chem. 11 (2020) 6953–6963. https://doi.org/10.1039/d0py01291b.

[109] S.H. Kim, K.H. Lee, J.Y. Chu, A.R. Kim, D.J. Yoo, Enhanced hydroxide conductivity and dimensional stability with blended membranes containing hyperbranched paes/linear ppo as anion exchange membranes, Polymers (Basel). 12 (2020) 3011. https://doi.org/10.3390/polym12123011.

[110] B.H. Oh, A.R. Kim, D.J. Yoo, Profile of extended chemical stability and mechanical integrity and high hydroxide ion conductivity of poly(ether imide) based membranes for anion exchange membrane fuel cells, Int. J. Hydrogen Energy. 44 (2019) 4281–4292. https://doi.org/10.1016/j.ijhydene.2018.12.177.

[111] Y. Li, J. Zhang, H. Yang, S. Yang, S. Lu, H. Wei, Y. Ding, Boosting the performance of an anion exchange membrane by the formation of well-connected ion conducting channels, Polym. Chem. 10 (2019) 2822–2831. https://doi.org/10.1039/c9py00011a.

[112] J.Y. Chu, K.H. Lee, A.R. Kim, D.J. Yoo, Graphene-mediated organic-inorganic composites with improved hydroxide conductivity and outstanding alkaline stability for anion exchange membranes, Compos. Part B Eng. 164 (2019) 324–332.

https://doi.org/10.1016/j.compositesb.2018.11.084.

[113] Y. Lu, X. Pan, N. Li, Z. Hu, S. Chen, Improved performance of quaternized poly(arylene ether ketone)s/graphitic carbon nitride nanosheets composite anion exchange membrane for fuel cell applications, Appl. Surf. Sci. 503 (2020) 144071. https://doi.org/10.1016/j.apsusc.2019.144071.

[114] M. Mandal, G. Huang, N.U. Hassan, X. Peng, T. Gu, A.H. Brooks-Starks, B. Bahar, W.E. Mustain, P.A. Kohl, The Importance of Water Transport in High Conductivity and High-Power Alkaline Fuel Cells, J. Electrochem. Soc. 167 (2020) 054501. https://doi.org/10.1149/2.0022005jes.

[115] H.S. Dang, P. Jannasch, High-Performing Hydroxide Exchange Membranes with Flexible Tetra-Piperidinium Side Chains Linked by Alkyl Spacers, ACS Appl. Energy Mater. 1 (2018) 2222–2231. https://doi.org/10.1021/acsaem.8b00294.

[116] A.M.A. Mahmoud, A.M.M. Elsaghier, K. Otsuji, K. Miyatake, High Hydroxide Ion Conductivity with Enhanced Alkaline Stability of Partially Fluorinated and Quaternized Aromatic Copolymers as Anion Exchange Membranes, Macromolecules. 50 (2017) 4256–4266. https://doi.org/10.1021/acs.macromol.7b00401.

[117] N. Chen, C. Lu, Y. Li, C. Long, Z. Li, H. Zhu, Tunable multi-cations-crosslinked poly(arylene piperidinium)-based alkaline membranes with high ion conductivity and durability, J. Memb. Sci. 588 (2019) 117120. https://doi.org/10.1016/j.memsci.2019.05.044.

[118] C. Shang, Z. Wang, L. Wang, J. Wang, Preparation and characterization of a polyvinyl alcohol grafted bis-crown ether anion exchange membrane with high conductivity and strong alkali stability, Int. J. Hydrogen Energy. 45 (2020) 16738–16750. https://doi.org/10.1016/j.ijhydene.2020.04.134.

[119] H.A.K. Iv, T.J. Clark, N.J. Robertson, P.F. Mutolo, J.M. Longo, G.W. Coates, Solvent Processable Tetraalkylammonium-Functionalized Polyethylene for Use as an Alkaline Anion Exchange Membrane, 43 (2010) 7147–7150. https://doi.org/10.1021/ma101172a.

[120] Y. Liu, J. Wang, Y. Yang, T.M. Brenner, S. Seifert, Y. Yan, M.W. Liberatore, A.M. Herring, Anion transport in a chemically stable, sterically bulky α-C modified imidazolium functionalized anion exchange membrane, J. Phys. Chem. C. 118 (2014) 15136–15145. https://doi.org/10.1021/jp5027674.

[121] K. Firouz Tadavani, A. Abdolmaleki, M.R. Molavian, M. Zhiani, New Strategy Based on Click Reaction for Preparation of Cross-Linked Poly(Benzimidazolium-Imide) as an Anion-Exchange Membrane with Improved Alkaline Stability, Ind. Eng. Chem. Res. 60 (2021) 7097–7110. https://doi.org/10.1021/acs.iecr.1c00071.

[122] S. Willdorf-Cohen, A.N. Mondal, D.R. Dekel, C.E. Diesendruck, Chemical stability of poly(phenylene oxide)-based ionomers in an anion exchange-membrane fuel cell environment, J. Mater. Chem. A. 6 (2018) 22234–22239. https://doi.org/10.1039/C8TA05785K.

[123] L. Wang, J.J. Brink, Y. Liu, A.M. Herring, J. Ponce-González, D.K. Whelligan, J.R. Varcoe, Non-fluorinated pre-irradiation-grafted (peroxidated) LDPE-based anion-exchange membranes with high performance and stability, Energy Environ. Sci. 10 (2017) 2154–2167. https://doi.org/10.1039/c7ee02053h.

[124] X. Wu, K. Scott, CuxCo3-xO4 ($0 \leq x < 1$) nanoparticles for oxygen evolution in high performance alkaline exchange membrane water electrolysers, J. Mater. Chem. 21 (2011) 12344–12351. https://doi.org/10.1039/c1jm11312g.

[125] S.H. Ahn, B.S. Lee, I. Choi, S.J. Yoo, H.J. Kim, E.A. Cho, D. Henkensmeier, S.W. Nam, S.K. Kim, J.H. Jang, Development of a membrane electrode assembly for alkaline water electrolysis by direct electrodeposition of nickel on carbon papers, Appl. Catal. B Environ. 154–155 (2014) 197–205. https://doi.org/10.1016/j.apcatb.2014.02.021.

[126] I. V Pushkareva, A.S. Pushkarev, S.A. Grigoriev, P. Modisha, D.G. Bessarabov, Comparative study of anion exchange membranes for low-cost water electrolysis, Int. J. Hydrogen Energy. 45 (2020) 26070–26079. https://doi.org/10.1016/j.ijhydene.2019.11.011.

[127] Y.X. Chen, A. Lavacchi, H.A. Miller, M. Bevilacqua, J. Filippi, M. Innocenti, A. Marchionni, W. Oberhauser, L. Wang, F. Vizza, Nanotechnology makes biomass electrolysis more energy efficient than water electrolysis, Nat. Commun. 5 (2014) 4036. https://doi.org/10.1038/ncomms5036.

[128] L. Wang, T. Weissbach, R. Reissner, A. Ansar, A.S. Gago, S. Holdcroft, K.A. Friedrich, High Performance Anion Exchange Membrane Electrolysis Using Plasma-Sprayed, Non-Precious-Metal Electrodes, ACS Appl. Energy Mater. 2 (2019) 7903–7912. https://doi.org/10.1021/acsaem.9b01392.

[129] X. Hu, Y.D. Huang, L. Liu, Q. Ju, X.X. Zhou, X.Q. Qiao, Z.F. Zheng, N.W. Li, Piperidinium functionalized aryl ether-free polyaromatics as anion exchange membrane for water electrolysers: Performance and durability, J. Memb. Sci. 621 (2021) 118964. https://doi.org/10.1016/j.memsci.2020.118964.

[130] M.R. Kraglund, M. Carmo, G. Schiller, S.A. Ansar, D. Aili, E. Christensen, J.O. Jensen, Ion-solvating membranes as a new approach towards high rate alkaline electrolyzers, Energy Environ. Sci. 12 (2019) 3313–3318. https://doi.org/10.1039/c9ee00832b.

[131] A.Y. Faid, A.O. Barnett, F. Seland, S. Sunde, Highly Active Nickel-Based Catalyst for Hydrogen Evolution in Anion Exchange Membrane Electrolysis, Catalysts. 8 (2018). https://doi.org/10.3390/catal8120614.

[132] L. Zeng, T.S. Zhao, Integrated inorganic membrane electrode assembly with layered double hydroxides as ionic conductors for anion exchange membrane water electrolysis, Nano Energy. 11 (2015) 110–118. https://doi.org/10.1016/j.nanoen.2014.10.019.

[133] M.K. Cho, H.Y. Park, H.J. Lee, H.J. Kim, A. Lim, D. Henkensmeier, S.J. Yoo, J.Y. Kim, S.Y. Lee, H.S. Park, J.H. Jang, Alkaline anion exchange membrane water electrolysis: Effects of electrolyte feed method and electrode binder content, J. Power Sources. 382 (2018) 22–29. https://doi.org/10.1016/j.jpowsour.2018.02.025.

[134] R.I. Masel, Z. Liu, S.D. Sajjad, Anion Exchange Membrane Electrolyzers Showing 1 A/cm(2) at Less Than 2 V, ECS Trans. 75 (2016) 1143–1146. https://doi.org/10.1149/07514.1143ecst.

[135] H.J. Park, S.Y. Lee, T.K. Lee, H.J. Kim, Y.M. Lee, N3-butyl imidazolium-based anion exchange membranes blended with Poly alcohol) for alkaline water, J. Memb. Sci. 611 (2020) 118355. https://doi.org/10.1016/j.memsci.2020.118355.

[136] J.E. Park, M.J. Kim, M.S. Lim, S.Y. Kang, J.K. Kim, S.H. Oh, M. Her, Y.H. Cho, Y.E. Sung, Graphitic carbon nitride-carbon nanofiber as oxygen catalyst in anion-exchange membrane water electrolyzer and rechargeable metal-air cells, Appl. Catal. B-Environmental. 237 (2018) 140–148. https://doi.org/10.1016/j.apcatb.2018.05.073.

[137] F. Razmjooei, A. Farooqui, R. Reissner, A.S. Gago, S.A. Ansar, K.A. Friedrich, Elucidating the Performance Limitations of Alkaline Electrolyte Membrane Electrolysis: Dominance of Anion Concentration in Membrane Electrode Assembly, ChemElectroChem. 7 (2020) 3951–3960. https://doi.org/10.1002/celc.202000605.

[138] Y.S. Park, J. Lee, M.J. Jang, J. Yang, J. Jeong, J. Park, Y. Kim, M.H. Seo, Z. Chen, S.M. Choi, High-performance anion exchange membrane alkaline seawater electrolysis, J. Mater. Chem. A. 9 (2021) 9586–9592. https://doi.org/10.1039/d0ta12336f.

[139] R.E. Coppola, F.N. Molinari, N.B. D’Accorso, G.C. Abuin, Polyvinyl alcohol nanofibers reinforced with polybenzimidazole: Facile preparation and properties of an anion exchange membrane, Polym. Adv. Technol. 32 (2021) 3505–3514. https://doi.org/10.1002/pat.5361.

[140] J.D. Joe, D.B.S. Kumar, P. Sivakumar, Production Of Hydrogen By Anion Exchange Membrane Using AWE, Int. J. Sci. Technol. Res. 3 (2014) 38–42.

[141] J. Liu, Z. Kang, D. Li, M. Pak, S.M. Alia, C. Fujimoto, G. Bender, Y.S. Kim, A.Z. Weber, Elucidating the Role of Hydroxide Electrolyte on Anion-Exchange-Membrane Water Electrolyzer Performance, J. Electrochem. Soc. 168 (2021) 054522. https://doi.org/10.1149/1945-7111/ac0019.

[142] D. Xu, M.B. Stevens, M.R. Cosby, S.Z. Oener, A.M. Smith, L.J. Enman, K.E. Ayers, C.B. Capuano, J.N. Renner, N. Danilovic, Y. Li, H. Wang, Q. Zhang, S.W. Boettcher, Earth-Abundant Oxygen Electrocatalysts for Alkaline Anion-Exchange-Membrane Water Electrolysis: Effects of Catalyst Conductivity and Comparison with Performance in Three-Electrode Cells, ACS Catal. 9 (2019) 7–15. https://doi.org/10.1021/acscatal.8b04001.

[143] T. Pandiarajan, L.J. Berchmans, S. Ravichandran, Fabrication of spinel ferrite based alkaline anion exchange membrane water electrolysers for hydrogen production, RSC Adv. 5 (2015) 34100–34108. https://doi.org/10.1039/c5ra01123j.

[144] L. Zeng, T.S. Zhao, R.H. Zhang, J.B. Xu, NiCo2O4 nanowires@MnOx nanoflakes supported on stainless steel mesh with superior electrocatalytic performance for anion exchange membrane water splitting, Electrochem. Commun. 87 (2018) 66–70. https://doi.org/10.1016/j.elecom.2018.01.002.

[145] P. Thangavel, M. Ha, S. Kumaraguru, A. Meena, A.N. Singh, A.M. Harzandi, K.S. Kim, Graphene-nanoplatelets-supported NiFe-MOF: High-efficiency and ultra-stable oxygen electrodes for sustained alkaline anion exchange membrane water electrolysis, Energy Environ. Sci. 13 (2020) 3447–3458. https://doi.org/10.1039/d0ee00877j.

[146] J.E. Park, S.Y. Kang, S.H. Oh, J.K. Kim, M.S. Lim, C.Y. Ahn, Y.H. Cho, Y.E. Sung, High-performance anion-exchange membrane water electrolysis, Electrochim. Acta. 295 (2019) 99–106. https://doi.org/10.1016/j.electacta.2018.10.143.

[147] F. Razmjooei, T. Morawietz, E. Taghizadeh, E. Hadjixenophontos, L. Mues, M. Gerle, B.D. Wood, C. Harms, A.S. Gago, S.A. Ansar, K.A. Friedrich, Increasing the performance of an anion-exchange membrane electrolyzer operating in pure water with a nickel-based microporous layer, Joule 5 (2021) 1776–1799. https://doi.org/10.1016/j.joule.2021.05.006.

[148] Y. Leng, G. Chen, A.J. Mendoza, T.B. Tighe, M.A. Hickner, C.Y. Wang, Solid-state water electrolysis with an alkaline membrane, J. Am. Chem. Soc. 134 (2012) 9054–9057. https://doi.org/10.1021/ja302439z.
